# Comparison of time and dose dependent gene expression and affected pathways in primary human fibroblasts after exposure to ionizing radiation

**DOI:** 10.1186/s10020-020-00203-0

**Published:** 2020-09-09

**Authors:** Lara Kim Brackmann, Alicia Poplawski, Caine Lucas Grandt, Heike Schwarz, Thomas Hankeln, Steffen Rapp, Sebastian Zahnreich, Danuta Galetzka, Iris Schmitt, Christian Grad, Lukas Eckhard, Johanna Mirsch, Maria Blettner, Peter Scholz-Kreisel, Moritz Hess, Harald Binder, Heinz Schmidberger, Manuela Marron

**Affiliations:** 1 Leibniz Institute for Prevention Research and Epidemiology – BIPS, Achterstr. 30, 28359 Bremen, Germany; 2 Institute of Medical Biostatistics, Epidemiology and Informatics, University Medical Center of the Johannes Gutenberg University Mainz, Mainz, Germany; 3 Institute of Organismal and Molecular Evolution, Molecular Genetics and Genome Analysis, Johannes Gutenberg University Mainz, Mainz, Germany; 4 Department of Radiation Oncology and Radiation Therapy, University Medical Center of the Johannes Gutenberg University Mainz, Mainz, Germany; 5 Department of Orthopaedics and Traumatology, University Medical Center of the Johannes Gutenberg University Mainz, Mainz, Germany; 6 Radiation Biology and DNA Repair, Darmstadt University of Technology, Darmstadt, Germany; 7 Institute of Medical Biometry and Statistics, Faculty of Medicine and Medical Center Freiburg, University of Freiburg, Freiburg, Germany

**Keywords:** Childhood cancer, Fibroblasts, Gene-radiation interaction, High dose, Ionizing radiation, IPA, Low dose, RNA sequencing, Second primary neoplasm

## Abstract

**Background:**

Exposure to ionizing radiation induces complex stress responses in cells, which can lead to adverse health effects such as cancer. Although a variety of studies investigated gene expression and affected pathways in human fibroblasts after exposure to ionizing radiation, the understanding of underlying mechanisms and biological effects is still incomplete due to different experimental settings and small sample sizes. Therefore, this study aims to identify the time point with the highest number of differentially expressed genes and corresponding pathways in primary human fibroblasts after irradiation at two preselected time points.

**Methods:**

Fibroblasts from skin biopsies of 15 cell donors were exposed to a high (2Gy) and a low (0.05Gy) dose of X-rays. RNA was extracted and sequenced 2 h and 4 h after exposure. Differentially expressed genes with an adjusted *p*-value < 0.05 were flagged and used for pathway analyses including prediction of upstream and downstream effects. Principal component analyses were used to examine the effect of two different sequencing runs on quality metrics and variation in expression and alignment and for explorative analysis of the radiation dose and time point of analysis.

**Results:**

More genes were differentially expressed 4 h after exposure to low and high doses of radiation than after 2 h. In experiments with high dose irradiation and RNA sequencing after 4 h, inactivation of the *FAT10 cancer signaling pathway* and activation of *gluconeogenesis I*, *glycolysis I,* and *prostanoid biosynthesis* was observed taking *p*-value (< 0.05) and (in) activating z-score (≥2.00 or ≤ − 2.00) into account. Two hours after high dose irradiation, inactivation of *small cell lung cancer signaling* was observed. For low dose irradiation experiments, we did not detect any significant (*p* < 0.05 and z-score ≥ 2.00 or ≤ − 2.00) activated or inactivated pathways for both time points.

**Conclusions:**

Compared to 2 h after irradiation, a higher number of differentially expressed genes were found 4 h after exposure to low and high dose ionizing radiation. Differences in gene expression were related to signal transduction pathways of the DNA damage response after 2 h and to metabolic pathways, that might implicate cellular senescence, after 4 h. The time point 4 h will be used to conduct further irradiation experiments in a larger sample.

## Background

Exposure to ionizing radiation induces complex stress responses in cells (Albrecht et al. 2012) and can lead to genomic instability (Kadhim and Hill 2015). These effects are not only limited to the irradiated cells but also observed in adjacent, untreated bystander cells (Mavragani et al. 2016). Such radiation-induced changes in human cells can lead to long-term health outcomes such as cancer (Brooks et al. 2016; Hwang et al. 2008; Cardis et al. 2007; Ronckers et al. 2008; Goodhead 2009; Richardson et al. 2015; Leuraud et al. 2015) as well as cardiovascular (Baselet et al. 2016; Stewart 2012; Menezes et al. 2018; Adams et al. 2003; van der Pal et al. 2005), and other chronic diseases (Vrijheid et al. 2007). Several research groups investigated various types of skin cells to identify differences in gene expression after exposure to ionizing radiation (Sokolov and Neumann 2015). Studies comparing different doses of radiation and time points of analyses reported on more differentially expressed genes (DEGs) in fibroblasts after exposure to a high (HDIR) than to a low dose (LDIR) of ionizing radiation (Hou et al. 2015) and only little overlap of expressed genes between LDIR and HDIR (Velegzhaninov et al. 2015; Mezentsev and Amundson 2011). Moreover, the time point with the highest numbers of DEGs differed from 4 h (Ding et al. 2005) over 16 h (Mezentsev and Amundson 2011) and 24 h (Hou et al. 2015; Mezentsev and Amundson 2011) to 30 h (Albrecht et al. 2012) in a dose-dependent manner. Besides these quantitative differences of gene expression in primary human skin fibroblasts, qualitative divergences, like different expression profiles of genes included in *p53*-associated pathways, have been shown 1 h, 2 h, 4 h and 24 h after exposure to LDIR (0.02 Gray (Gy)) and HDIR (4Gy) (Ding et al. 2005).

Despite the available studies on changes in gene expression and affected pathways in human fibroblasts after exposure to ionizing radiation, the understanding of underlying mechanisms and biological effects is still incomplete for this cell type, especially for low doses (Albrecht et al. 2012; Sokolov and Neumann 2015). The results of the conducted studies are difficult to compare since a variety of different experimental setups were used: Gene expression was measured at different time points, after exposure to different radiation doses and in different cell types (Sokolov and Neumann 2015; Ding et al. 2005; Ray et al. 2012; Yunis et al. 2012; Warters et al. 2009; Stecca and Gerber 1998). Most of the studies were conducted with only a small number of cell donors (Albrecht et al. 2012; Warters et al. 2009; Berglund et al. 2008; Goldberg et al. 2004). Others used skin models (Mezentsev and Amundson 2011; Ray et al. 2012; Yunis et al. 2012), which are not an exact copy of the skin in living humans (De Wever et al. 2015) or established cell lines (Hou et al. 2015; Velegzhaninov et al. 2015; Ding et al. 2005; Kalanxhi and Dahle 2012), whose genotype and phenotype might have changed over time (Kaur and Dufour 2012).

In this study we aim to establish the experimental settings and setup the analysis to identify DEGs and corresponding pathways for further irradiation experiments. Primary human fibroblasts from a subsample of 15 selected cell donors will be irradiated with a high and a low radiation dose, and experiments will be ended at two predefined time points from the literature and preliminary experiments. Comparing these time points, we aim to identify the time point with the highest number of DEGs. The identified time point should then be used in a further project to identify differences in gene expression of former childhood cancer patients with and without a second primary neoplasm (SPN) and cancer-free controls in a study sample of 153 participants. In addition to the descriptive analysis of DEGs, gene expression patterns and affected pathways will be analyzed and compared as well as upstream and downstream effects will be predicted.

## Design, subjects and methods

### Study design and participants

All donors were participants of the ongoing population-based nested case-control study KiKme (Marron et al. 2020). The KiKme project aims to identify differences in genetic predispositions and gene-radiation interactions between former childhood cancer patients and cancer-free controls (*N* = 591). Since radiation-induced changes in human cells can lead to long-term health outcomes such as cancer (Brooks et al. 2016; Hwang et al. 2008; Cardis et al. 2007; Ronckers et al. 2008; Goodhead 2009; Richardson et al. 2015; Leuraud et al. 2015), the identified time point from this work should be used as guidance in further research projects of the study to analyze differences in gene expression patterns between the different groups of study participants. Since differential gene expression might differ between cancer patients and cancer-free controls, we choose to analyze samples from all three patient groups in this work. The recruitment for the KiKme study started in 2013 and includes 591 participants until now. Recruiting strategies and development as well as information on data collection are described in detail elsewhere (Marron et al. 2020). Briefly, the study population consists of former childhood cancer patients with a first primary neoplasm (FPN) only or a subsequent SPN registered at the German Childhood Cancer Registry (Scholz-Kreisel et al. 2018). FPN patients were matched as cancer controls by age, sex, cancer site, year of diagnosis, and age at diagnosis to available SPN cases using an incidence density sampling approach. Cancer-free controls for each matching group were recruited from the Department of Orthopaedics and Traumatology at the Johannes Gutenberg-University in Mainz (Germany) and matched by sex and age within a maximal 5-year age range to the participating SPN cases and FPN controls. They were included if they were hospitalized for an elective surgery unrelated to cancer. Patients with severe diseases were excluded from participation (e.g. cancer, hemophilia, HIV, hepatitis, diabetes). For this work, skin biopsies were taken from 15 participants by punch biopsy with a diameter of 3 mm on the inside of the cubital region for cases and near the surgery region for cancer-free controls. Fibroblasts were isolated, cultivated, and cryopreserved until further usage. Moreover, saliva collection with subsequent DNA extraction took place, and each study participant answered a self-completion questionnaire to assess socio-economical and anthropometric factors as well as information on lifestyle, medical history, and health.

### Irradiation of fibroblasts with subsequent ribonucleic acid (RNA) isolation

For radiation experiments, fibroblasts were cultivated and synchronized in the G_0_/G_1_ phase of the cell cycle by contact inhibition to exclude cell cycle-dependent effects on gene-expression profiles. To this end, cells were seeded at a density of 9000 cells per cm^2^ and cultured for 14 to 15 days. G_0_/G_1_ arrest was confirmed by flow cytometry when the experiment was performed (Web Figure 1). Radiation experiments were conducted using the D3150 X-ray therapy system (Gulmay Medical Ltd., Byfleet, UK). Fibroblasts were exposed to a HDIR of 2Gy, comparable to an average single tumor-dose of fractionated radiation therapy (Seidlitz et al. 2017), and a LDIR of 0.05Gy, comparable to an organ dose of a computed tomography scan (Pearce et al. 2012) or were sham-irradiated (0Gy). Cells from matched triplets, consisting of an SPN, an FPN, and a corresponding cancer-free donor, were cultivated and treated simultaneously to prevent batch effects within groups. For HDIR with 2Gy, fibroblasts were exposed to 140 kV X-rays at a dose rate of 3.62Gy per minute. To apply LDIR of 0.05Gy with the same X-irradiation system, a dose rate of 0.34Gy per minute was achieved by increasing the distance from the source to target by 30 cm and via reduction of the voltage to 50 kV. Cells were exposed at room temperature and sham-irradiated cells for each time point of analysis were kept at the same conditions in the radiation device control room.

To identify the time points post-radiation with the highest numbers of DEGs, we conducted preliminary experiments with several time points with fibroblasts from 3 cancer-free controls (Web Figure 2). From these experiments the time point 2 h was chosen due to the largest number of DEGs after radiation exposure for both, the LDIR and HDIR. We selected the time point of analysis after 4 h for LDIR from the literature (Ding et al. 2005). Thus, the final experimental settings for fibroblasts from 5 SPN cases, 5 FPN controls and 5 cancer-free controls were defined as follows: irradiation with 2Gy and RNA extraction after 2 h (2Gy–2h), irradiation with 2Gy and RNA extraction after 4 h (2Gy–4h), irradiation with 0.05Gy and RNA extraction after 2 h (0.05Gy–2h), irradiation with 0.05Gy and RNA extraction after 4 h (0.05Gy–4h), no radiation and RNA extraction after 2 h (0Gy–2h), no radiation and RNA extraction after 4 h (0Gy–4h).

RNA was isolated using the NucleoSpin RNA Plus (MACHEREY-NAGEL GmbH & Co. KG, Düren, Germany). RNA integrity was assessed using a Bioanalyzer 2100 (Agilent RNA 6000 Nano Kit, Agilent Technologies, Santa Clara, California, USA). Sequencing library construction was done using 1 μg of total RNA (as quantified by QuBit, Thermo Fisher Scientific, Waltham, Massachusetts, USA) with an RNA integrity number greater or equal to 8 with the TruSeq RNA Sample Prep Kit v2 (Set A and B, Illumina, San Diego, California, USA) following the manufacturer’s instruction. RNA-Sequencing libraries were pooled, cBot-clustered, and sequenced on a HiSeq2500 instrument (Illumina, San Diego, California, USA) in high-output mode. Single-end reads with a length of 50 base pairs were generated using TruSeq Single Read Cluster Kit v3 (Illumina, San Diego, California, USA) and TruSeq SBS Kit v3 (Illumina, San Diego, California, USA). Data was generated by Real Time Analysis Version 1.8.4 and converted into FASTQ format using bcl2fastq Version 1.8.4 (Illumina, San Diego, California, USA).

We chose *CDKN1A* (*Cyclin-Dependent Kinase Inhibitor 1A*) and *MDM2* (*Mouse double minute 2 homolog*) as marker genes to validate the RNA-sequencing experiments via Real-Time Quantitative Polymerase-Chain-Reaction (qPCR) in 6 participants (2 SPN, 2 FPN, and 2 cancer-free controls). They consist of two matched groups, each including an SPN, an FPN, and a cancer-free control. The first diagnosis of the SPN and FPN was leukemia or lymphoma, respectively. The site of the SPN was chosen to be potentially radiation-associated (thyroid cancer or leukemia). The methods for this validation were described elsewhere (Galetzka et al. 2020).

### Bioinformatical and statistical analyses

To identify the time point with the largest number of DEGs after radiation exposure, RNA sequencing data had to be processed first. Raw reads were cleaned for adapter sequences using Trimmomatic (Bolger et al. 2014): Bases with a quality less than 3 were removed and reads were trimmed if the average quality over 4 bases was less than 15. Processed reads were aligned to the human reference genome (GRCh38) using STAR (STAR-2.6.0c) (Dobin et al. 2013). Expression per gene, given as the number of aligned reads per gene, was quantified using FeatureCounts (Rsubread v1.30.9) (Liao et al. 2014). Only genes with a minimum of 10 counts in at least 4 samples were analyzed. Data were normalized for sequencing depth using the DESeq package (v1.28.0) (Anders and Huber 2010). Reads were aggregated (summed) on the level of UCSC gene annotations. To address intra-patient correlation, random effect models fitted with lme4 (Bates et al. 2015) were used to estimate the among-patient variation, and the resulting residuals were further inspected. Afterwards, a principal component analysis was conducted with the standardized residuals using the R package stats (R-3.4.4). Correlation of the first three principal components and RNA quality parameters as well as the number of aligned raw reads and normalized number of aligned reads were inspected visually.

For the analysis of differential expression, data was transformed via the Voom (Law et al. 2014) method implemented in the limma package (v3.34.9) (Ritchie et al. 2015). DEGs dependent on radiation dose were detected for defined time points using linear models implemented in the limma package (Ritchie et al. 2015) with blocking on the patient. For each time point and radiation dose the gene expression was compared to the same time point post-radiation after sham-irradiation not taking the disease status into account. To account for expressional variability, we used variance modeling and borrowing information across genes (Ritchie et al. 2015). Additionally, our limma model included the patient identifiers accounting for a random variance. DEGs with a *p*-value smaller than 0.05 after adjustment for false discovery rate (Benjamini-Hochberg procedure) were flagged as significant and used for pathway analyses. Since also small coordinated changes in gene expression might lead to important physiological changes (Christmann and Kaina 2013), there was no restriction set regarding the log fold change.

Finally, pathway analyses were conducted via Ingenuity Pathway Analysis (IPA, Version 1.13, QIAGEN Inc., 2018). As input, lists of DEGs containing previously generated gene-wise *p*-values for each combination of time point and radiation dose, as well as log2-fold changes were used. Settings for comparison analyses in IPA were selected for experimental data in human fibroblasts or alike cells, molecule types, and data sources. The complete setting list can be found in the Supplement file 1. Negative log (−log10) *p*-values of at least 1.30 (≙ p-value = 0.05) were defined as significant. Activating z-score threshold was chosen as greater or equal than 2 or less than or equal minus 2 (Krämer et al. 2014). The z-score indicates pathway (de-)activation by comparing given expressional directions of pathway components with information from the data set entered for analysis (e.g. log-fold change). In addition, we used the comparison analysis in IPA to display and compare pathways across all experiments. Moreover, we included an overview of predicted downstream outcomes and upstream regulators. Analyses were conducted on March 3, 2020, and based on the IPA December 2019 Update.

## Results

A sample of 15 participants was selected from the KiKme study (*N* = 591). They were grouped into 5 matched triplets, each consisting of 1 SPN, 1 FPN, and 1 cancer-free control. Cells originated from 9 male and 6 female participants with a mean age of 28.27 years (age at recruitment: 21–40 years). FPN diagnoses were lymphoma (*n* = 6) or leukemia (*n* = 4) and they were diagnosed at a mean age of 8.10 years (age at FPN diagnosis: 4–14 years). SPN diagnoses were thyroid (*n* = 2) or skin cancer (n = 2) or leukemia (*n* = 1) and occurred at a mean age of 20.00 years (age at SPN diagnosis: 10–36 years).

Primary fibroblasts of the 15 participants were irradiated with a high and a low radiation dose. RNA was isolated 2 h and 4 h after the exposure and used to identify differential gene expression via RNA-sequencing. After normalizing for sequencing depth and removing inter-patient variation, no obvious correlation of RNA quality or sequencing depth with expression variation was observed (Web Figure 3). The validation of the RNA-sequencing experiments was successfully done using *CDKN1A* and *MDM2* as marker genes (Web Figure 4–5). The qPCR furthermore showed that all cells reacted similarly.

### Differential gene expression in reaction to LDIR and HDIR

We compared the gene expression of irradiated and sham-irradiated cells ignoring the tumor status because the sample size of 15 participants is too small to compare different groups of patients. The gene expression 2 h after irradiation differed markedly from the response 4 h after irradiation compared to unirradiated cells. This is indicated by separation of both time points along the first two principal components. The first and fifth principal variance components additionally showed variability of the radiation doses. HDIR samples showed a higher separation from the non-irradiated samples compared to the LDIR samples (Web Figure 6).

**Fig. 1:**
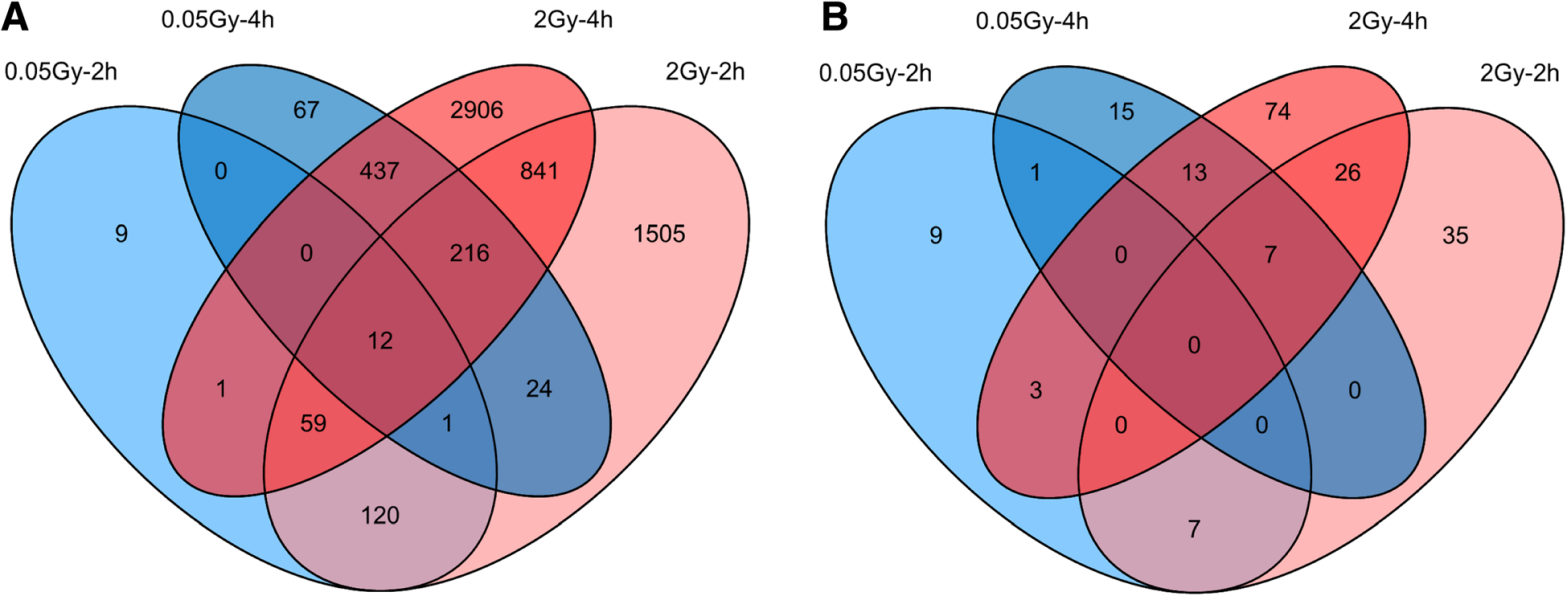
Differentially expressed genes (DEGs) (**a**) and pathways affected by DEGs (fisher’s exact test, *p* ≤ 0.05) (**b**) in human fibroblasts after exposure to ionizing radiation. **a** DEGs 2 and 4 h after low (0.05 Gray (Gy)) and high dose (2Gy) radiation exposure (adjusted for false discovery rate (< 0.05)). **b** Number of identified pathways in Ingenuity Pathway analysis, where fisher’s exact test showed a significant overlap of genes in pathway subset and DEGs (−log(*p*-value) ≤1.3) but not significant activational prediction (z-score: - 2 ≤ z ≤ 2)

Compared to unexposed cells, a larger number of DEGs was found at 4 h after exposure to LDIR (*N* = 757 genes, Web Table 1B) and to HDIR (*N* = 4472 genes, Web Table 1D) than after 2 h for both radiation doses (LDIR: *N* = 202 genes, Web Table 1A; HDIR: *N* = 2778 genes, Web Table 1C). For the LDIR treatment, differential expression of 9 and 67 genes was found in the 0.05Gy–2h and 0.05Gy–4h sample only, respectively (Fig. 1a, Web Table 1A, 1B, 1C, 1D). An increase in DEGs was also present for the HDIR treatment. Considering genes that were only differentially expressed in the experiment with 2Gy irradiation, about twice as many genes (N = 2906) were found to be differentially expressed exclusively after 4 h compared to 2 h (N = 1505; Fig. 1a, Web Table 1C). Additional 841 DEGs were identified at both time points after exposure to HDIR. Twelve genes were found to be differentially expressed in all 4 experimental settings.

### Pathway analysis

**Fig. 2:**
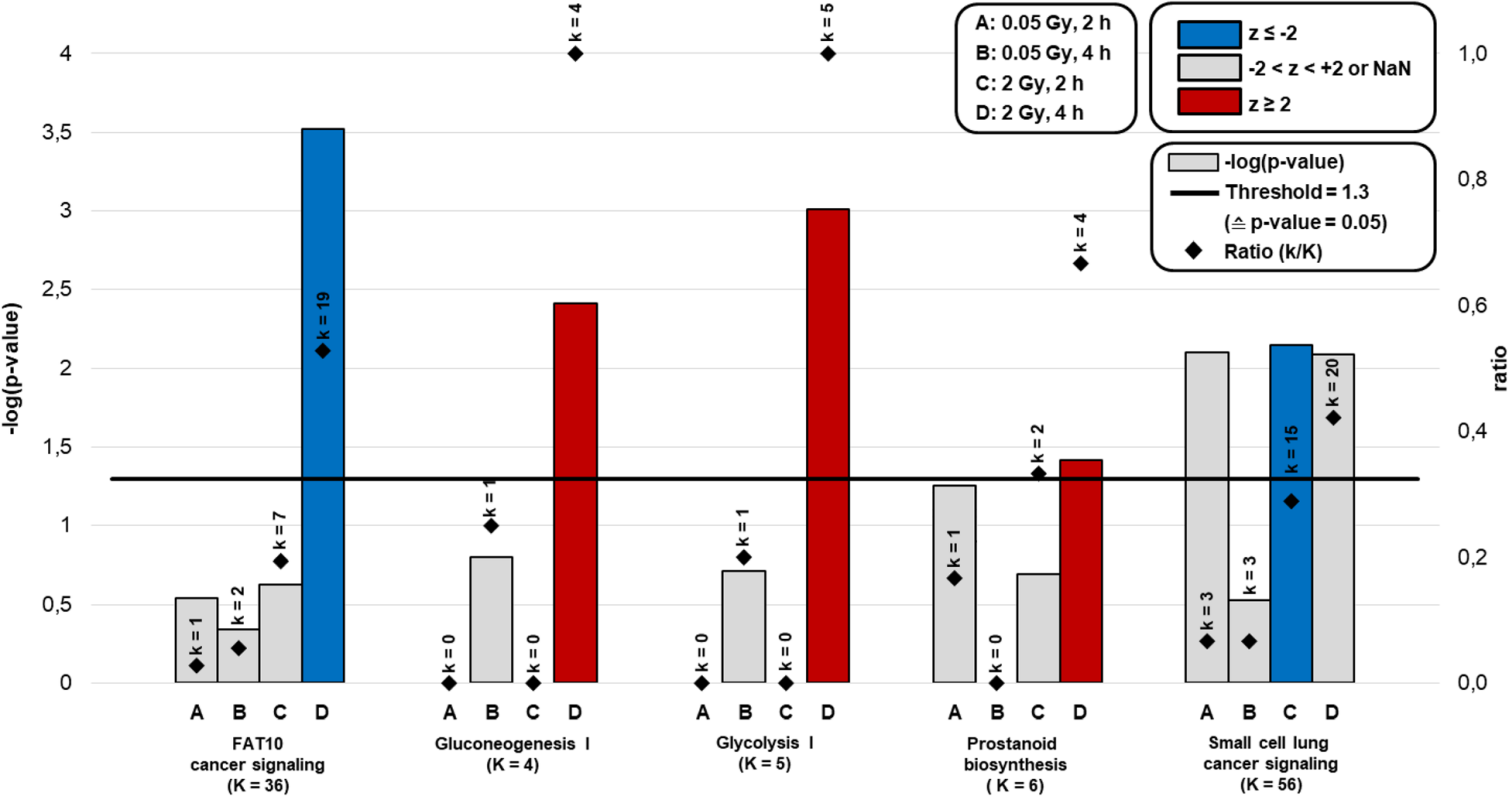
Pathways affected by differentially expressed genes after high or low dose irradiation of primary human fibroblasts. Abbreviations: not a number (NaN), Gray (Gy), hours (h), *Gq* *protein alpha subunit* (*Gαq*), *phosphoinositide 3-kinase* (*PI3K*), *v-akt murine thymoma viral oncogene* (*AKT*), z-score (z), number of differential expressed genes (k), number of total genes in pathway (K)

Using the Qiagen Knowledge Base in IPA, we identified 5 cellular pathways related to the DEGs. In these pathways, differential expression of genes exceeded a significant *p*-value in at least one experimental setting and the activating z-score threshold was surpassed to determine an activation or inhibition of pathways (Fig. 2, Web Figure 7–8, Web Table 2). For each pathway, a ratio of DEGs divided by the number of total genes in the pathway (k/K) is given as an indication of the enrichment.

For the 2Gy-2h samples, *small cell lung cancer signaling* pathway (z = − 2.12, k/K = 15/56) was predicted to be inactivated. For 2Gy-4h samples, 4 pathways with significant *p*-values and z-score were detected (*FAT10 cancer signaling pathway, gluconeogenesis I, glycolysis I,* and *prostanoid biosynthesis*). Three of them were predicted to be activated, indicated by a positive z-score (*gluconeogenesis I*: z = 2.00, k/K = 4/4; *glycolysis I*: z = 2.24, k/K = 5/5; *prostanoid biosynthesis*: z = 2.00, k/K = 4/6). *FAT10 cancer signaling pathway* was predicted to be inactivated (z = − 2.07, k/K = 19/39). In addition, 2 pathways based on liver and T-cells (*hepatic fibrosis signaling* (z = − 2.29, k/K = 70/214) and *T-cell exhaustion* (z = − 2.68, k/K = 26/72)) were predicted to be inactivated. However, these 2 pathways were excluded concerning content for discussion. None of the mentioned pathways for HDIR were significantly altered at both time points (Web Table 2). Based on the applied criteria, no pathways were significantly altered in any of the LDIR samples.

We further identified 190 additional cellular pathways, where differential expression activity in genes reached a significant *p*-value but were not predicted to be activated or inactivated via z-score (Fig. 1b, Web Figure 7–8, Web Table 2). However, none of the pathways were found to be activated or inactivated in more than one experimental setting (Web Figure 7–8). In 0.05Gy-4h samples, mainly metabolic pathways exceeded a –log(p-value) of 1.30. Signaling pathways with only significant p-value were identified for both time points after exposure to LDIR. A stronger time-dependent increment of significant pathways (only in p-value, Fig. 1b) was observed after LDIR (20 pathways after 2 h compared to 36 pathways after 4 h, an increase of 80%) than after HDIR (75 pathways after 2 h compared to 123 pathways after 4 h, increase of 64%). Two hours after exposure to HDIR, differences in gene expression were related to signal transduction pathways of the DNA damage response. Four hours after exposure to HDIR, the pattern had changed to metabolic pathways (Fig. 2, Web Table 2, Web Figure 7–8).

When considering resulting diseases and functions in a downstream prediction, LDIR experiments again showed only a few results for activity patterns (Web Figure 9). Two hours after exposure to LDIR, only *cell proliferation of fibroblasts,* which can be grouped as a function of cell cycle progression, was predicted to be inactivated (z = − 0.07). Likewise, only functions of cell cycle progression were found to be inactivated after 4 h after LDIR. However, cell cycle progression was indicated as activated at this time point and radiation dose. After exposure to HDIR, processes of cell cycle progression were found to be inactivated at both time points (Web Figure 9). While 2 h after exposure to HDIR additionally functions related to senescence and cell transformation were predicted to be inactivated, functions of senescence, apoptosis, metabolism, and repair mechanisms were mainly predicted to be activated in IPA. In the prediction of upstream regulators especially *p53* was found to be activated after exposure to HDIR after 2 h (z = 1.77) and after 4 h (z = 1.72, Web Figure 10). Moreover, Interleukins and *mechanistic Target of Rapamycin* (*mTOR*) were predicted to be downregulated after 2 h with a significant z-score > |2| (Web Figure 10).

## Discussion

To identify the time point with the highest number of DEGs in primary human fibroblasts after exposure to LDIR or HDIR for the usage in later study projects, we compare gene expression profiles and associated cellular pathways at 2 h and 4 h post radiation. More DEGs were detected 4 h after exposure to both LDIR and HDIR. In 2Gy-2h samples, *small cell lung cancer signaling* was predicted to be inactivated. In 2Gy-4h samples, we observed inactivation of *FAT10 cancer signaling,* and activation of *gluconeogenesis I***,** *glycolysis I,* and *prostanoid biosynthesis.* Exposure to LDIR did not cause a significant difference in pathway activation prediction via z-score for both time points of analysis.

### Differentially expressed genes after irradiation

As reported by previous studies (Albrecht et al. 2012; Hou et al. 2015; Mezentsev and Amundson 2011), the number of DEGs differed largely across our 4 experimental settings. In total, more genes were differentially expressed after exposure to HDIR than to LDIR at both time points. The increase of DEGs from 2 h to 4 h was much more pronounced in LDIR compared to HDIR. Following HDIR, a fast cellular response is expected according to the strong genotoxic impact inducing a high count of DEGs already after 2 h. Therefore, the increase of DEGs from 2 h to 4 h after exposure to HDIR might be rather minor compared to LDIR since the stimuli of the lower energetic nature in LDIR may cause a more delayed response and rise of DEGs. In line with our assumptions, Ding et al. (Ding et al. 2005) reported on a maximum of DEGs 2 h after exposure to 4Gy and 4 h after exposure to 0.02Gy. In our study, also the number of significant pathways (only in *p*-value) showed a time-dependent increase for low and high doses, corresponding with this hypothesis of delayed gene expression patterns post-radiation. Only 12 genes were found to be differentially expressed under all experimental conditions. This finding is in line with results from several other groups indicating only a little overlap of DEGs and activated pathways for different time points and radiation doses (Sokolov and Neumann 2015; Velegzhaninov et al. 2015; Mezentsev and Amundson 2011). In addition, we compared the DEGs of our experiments with genes listed in the RadAtlas (Xu et al. 2020), which is a recently published database for radiation-associated genes. In the 2Gy-4h experiment, 244 (29%) of our DEGs were found in the 844 genes described in the database. In the other experiments, 15% (2Gy-2h), 5% (0.05Gy-4h) and 1% (0.05Gy-2h) of our DEGs were listed in the RadAtlas, respectively (data not shown).

We furthermore compared our results on affected pathways to this database (Xu et al. 2020) and other existing datasets (Ghandhi et al. 2015). Therefore, we choose all available single-fraction datasets with existing sham-irradiated (0Gy) control cells, manually calculated their log-fold changes, and included them to our IPA analysis. We identified similar patterns of activation and inactivation of pathways (Web Figure 11). Likewise, our results on downstream diseases and functions (Web Figure 12) and on upstream regulators (Web Figure 13) were also comparable to those from available datasets (Xu et al. 2020; Ghandhi et al. 2015), especially when considering other human samples. However, predicted downstream effects from gene expression in mouse blood cells tend to differ from available human samples. In particular, cell death of lymphocytes was predicted to be inactivated in mice, whereas lymphocytes in human samples are known to activate processes of cell death after radiation exposure (Miszczyk et al. 2018). This was also observed in human samples in our comparison analysis (Web Figure 11). Interestingly, *Interleukins 1A, 1B*, and *17A* were predicted to be inactivated as upstream regulators in our 2Gy-2h experiments, whereas they were predicted to be activated in human blood samples 4 h after exposure to 1.25Gy of ionizing radiation (Web Figure 13). Interleukins are important factors for cell signaling and cancer progression (Mantovani et al. 2018), and usually described to increase after exposure to ionizing radiation (Liu et al. 2006; Liao et al. 2017; Li et al. 2015). However, we observed inactivation of *mTOR* in the same experiment, which was previously described to suppress the translation of *Interleukin 1A* (Laberge et al. 2015).

### Affected pathways following HDIR

Corresponding to the identified genes from RNA sequencing and subsequent processing, the *small cell lung cancer signaling* pathway was found to be inhibited in 2Gy-2h samples compared to sham-irradiation. The *small cell lung cancer signaling* pathway includes the two key players *Phosphoinositide* *3-kinase* (*PI3K*) and *nuclear factor* *kappa-light-chain-enhancer of activated B cells* (*NF-κB*). *PI3K* showed decreased gene expression in our 2Gy-2h experiments. Lack of *PI3K* leads to activation of *NF-κB*, which is usually linked to stress response (e.g. exposure to ionizing radiation) (QIAGEN 2018) and has been previously reported as a potential radiation biomarker (Stecca and Gerber 1998; Park et al. 2002) as well as a key player in inducing transcription of anti-apoptotic genes after exposure to ionizing radiation (QIAGEN 2018; Maier et al. 2016). *PI3K* and *NF-κB* also play important roles in other pathways, that were found to be significant in the 2Gy-2h experiment, but failed to exceed a z-score > |2| (Web Figure 7). As an example, the *lymphotoxin-β receptor signaling* pathway (*p* = 0.01; z = − 1.89) activates several signaling pathways, including *NF-κB* and cell death. In addition, *PI3K* is closely associated with the *prolactin signaling* pathway, which was also significant via *p*-value in our analysis (p = 0.01; z = − 1.94). When comparing our analysis data to available datasets from other study groups (Xu et al. 2020; Ghandhi et al. 2015), the *small cell lung cancer signaling* pathway was also be found as significantly affected via p-value in human blood cells 4 h after exposure to 1.25Gy irradiation (Xu et al. 2020) and to all available datasets from human coronary artery endothelium cells and mouse tissues (Xu et al. 2020) (Web Figure 11). However, for none of these samples, a significant activity prediction could be calculated.

In addition, in 2Gy-2h samples, the *p53 signaling* pathway was found to be significant in *p*-value (*p* = 0.02; z = 1.94). *P53* is a very well-known mediator of the response to genotoxic stress and several other studies reported on *p53* stabilization and activation of its downstream signaling pathways as a response to HDIR (Albrecht et al. 2012; Hou et al. 2015; Mezentsev and Amundson 2011; Warters et al. 2009; Jen and Cheung 2005). We furthermore found *p53* as predicted to be activated as an upstream regulator in our IPA analysis 2 h after exposure to HDIR (Web Figure 10). This finding was also pronounced in 2Gy-4h samples, but with a smaller –log(p-value).

While we observed changes in the activity of pathways associated with intracellular signaling at 2 h after irradiation, cellular metabolic pathways were affected after 4 h. This shows a chronological trend in response to ionizing radiation. Immediately after irradiation, a complex signaling network of the DNA damage and cell cycle response is activated (2Gy-2h) causing a transient cell cycle arrest or its manifestation as premature senescence (2Gy-4h, Web Figure 9). The frequent induction of premature differentiation and senescence in fibroblasts after irradiation is in line with the significant activation of the *glycolysis I* pathway in 2Gy-4h samples since senescent fibroblasts show an increased rate of glucose metabolism through glycolysis (James et al. 2015). Likewise, the *gluconeogenesis I* pathway shows a significant activation in the 2Gy-4h samples. Since gluconeogenesis represents the reverse process of glycolysis, there is a large redundancy regarding the involved processes and enzymatic reactions and a concurrent activity of both pathways seems likely. Neither *glycolysis I* nor *gluconeogenesis I* was found to be affected in available data from other studies (Xu et al. 2020; Ghandhi et al. 2015) (Web Figure 11).

The activation of the *prostanoid biosynthesis* pathway comprising only 6 genes is driven by activation of 4 prostaglandin-E Synthase genes (Web Table 2). Their expression can be induced by *p53* and may be involved in *p53* mediated apoptosis (Polyak et al. 1997). Since the *p53 signaling pathway* in the 2Gy-4h samples also shows a significant activation via *p*-value (*p* < 0.01), the activation of the pathway seems plausible, although the z-score with 0.82 was not significant. The *prostanoid biosynthesis* pathway was also affected, when analyzing available data from radiation experiments with human blood cells (1.25Gy-4h) (Xu et al. 2020). However, the activity prediction showed no significant results for these samples (Web Figure 11).

Furthermore, we observed an enhanced expression of the *FAT10 cancer signaling pathway* in our 2Gy-4h experiment. The enhanced expression of this pathway was expected as a reaction to DNA damage according to a recent study (Chen et al. 2018) and can lead to prolonged survival and proliferation (Aichem and Groettrup 2016). When comparing our analysis data to available datasets from other studies (Xu et al. 2020; Ghandhi et al. 2015), the *FAT10 cancer signaling pathway* was also be found as significantly affected via *p*-value in human blood samples 4 h after exposure to a radiation dose of 1.25Gy (Web Figure 11). Likewise, samples from mouse blood showed this pathway to be affected 24 h after exposure to 1Gy irradiation (Web Figure 11). However, for both of these samples, the activity prediction did not exceed a z-score > |2|.

Some pathways were significant in p-value but received a z-score of “Not a Number”. For these pathways activity prediction is not possible, as data in the IPA-database was not sufficient for calculation of the z-score at the time of analysis. Hence, there is not enough information to date to predict the effect of our DEGs and calculate a reliable z-score. Nevertheless, results with this informational gap are also important, as some known radiation- and stress response-related pathways can be observed in this category. Significant pathways that had z = “Not a Number” were examined concerning content (Web Table 2, Web Figure 14–28).

In 2Gy-4h samples, the *base excision repair (BER) system* pathway was given as “Not a Number” via activating z-score (*p* = 0.04, Web Table 2, Web Figure 14). *BER* is one of the most prominent DNA repair pathways which is activated after exposure to genotoxic stressors including ionizing radiation (QIAGEN 2018; Chaudhry 2007; Krokan and Bjørås 2013). The gene expression of several members of *BER* repair was affected including proliferation cell nuclear antigen, *DNA polymerase beta (POLB), DNA ligase I (LIG1)*, and *DNA-(apurinic or apyrimidinic site) lyase (APEX1)*, highlighting the important role of this DNA repair pathway to maintain genomic integrity.

Furthermore, in both of our HDIR experiments, the *molecular mechanisms of cancer* pathway was flagged as *p*-value significant (2Gy-2h: *p* = 0.03; 2Gy-4h: *p* < 0.01, Web Table 2, Web Figure 15). This pathway fosters tumor progression and generation of mutations in onco- or tumor suppressor-genes as well as activation of related signaling pathways (QIAGEN 2018). Our data suggest a high radiation-related expression of key players of cell cycle regulation and death, e.g. of *CDKN1A*, *PUMA*, and *MDM2* as well as of the proto-oncogene *c-Fos*.

Comparable to our results, published data from other studies (Hou et al. 2015; Mezentsev and Amundson 2011; Ding et al. 2005; Warters et al. 2009; Kalanxhi and Dahle 2012) identified pathways related to signal transduction of the DNA damage response and senescence in a time-dependent manner: In one of the first conducted studies by Ding and colleagues (Ding et al. 2005), exposure to HDIR (4 Gy) resulted in apoptosis and cell proliferation in the human skin fibroblast cell line HSF42. Similar results for HDIR were found by a recent study using another human skin fibroblast cell line (AG01522) (Hou et al. 2015). In this study, 6 h after exposure to a high dose of 2Gy, cells responded to DNA damage by activation of the *p53* signaling network, apoptosis, and control of cell cycle. At the earlier time point (3 h) DEGs were mostly involved in *G-protein-coupled receptor downstream signaling*. They stated that cellular response started at 3 h to 6 h after irradiation, which was also reported by another study (Kalanxhi and Dahle 2012), and that cellular defense mechanisms occurred earlier after exposure to HDIR than to LDIR. Activation of *p53*-related pathways (Mezentsev and Amundson 2011; Warters et al. 2009) and cell cycle control (Mezentsev and Amundson 2011) after exposure to different high doses of ionizing radiation was also reported by other studies for the time points 4 h (Mezentsev and Amundson 2011; Warters et al. 2009), 16 h (Mezentsev and Amundson 2011) and 24 h (Mezentsev and Amundson 2011).

The time dependency of pathways related to different processes in the cell could be found in our data in the prediction of downstream diseases and functions in IPA (Web Figure 9). Comparable to the results from the study groups mentioned above (Hou et al. 2015; Mezentsev and Amundson 2011; Ding et al. 2005; Warters et al. 2009; Kalanxhi and Dahle 2012), functions related to senescence, apoptosis, metabolism, and repair mechanisms were predicted to be affected 4 h after exposure to HDIR in our experiments. None of them were found to be predicted as activated 2 h after exposure.

### Affected pathways following LDIR

For LDIR, no pathways surpassed our thresholds for *p*-value and activating z-score thresholds. This observation can either correspond to the hypothesis of delayed gene expression patterns in LDIR or can be caused by a high inter-individual variation in the response to LDIR (Wilson et al. 2010), which hinders the detection of significant differences. However, we identified several pathways that are related to DEGs after LDIR and were significant only in *p*-value, but not in activating z-score. Like after HDIR, the *molecular mechanisms of cancer* pathway was also found to be p-value significant in the 0.05Gy-4h experiment (*p* < 0.01, Web Table 2, Web Figure 15). However, given the result “Not a Number”, activity prediction for this pathway is not possible.

Similar to our LDIR pathway analysis, a study investigating the transcriptional response to LDIR in skin biopsies was also not able to identify a significant activation or inactivation of pathways previously identified after in vitro LDIR of normal human skin fibroblasts (AG01522) (Berglund et al. 2008). They conducted their experiments with skin biopsies obtained from five prostate cancer patients after in vivo exposure during radiation therapy. Even if we could not identify significant pathways via p-value and z-score in our LDIR experiments, other studies reported on changes in gene expression related to several mechanisms in the cell after exposure to LDIR. A recent study in normal human skin fibroblasts (AG01522) identified biological processes responding to stress induced by ionizing radiation shortly after exposure (Hou et al. 2015). Amongst others, these processes included activation and signaling amplification of G proteins, apoptotic pathways, DNA and RNA metabolic processes, kinase activity, DNA repair, and replication as well as cell cycle arrest (Hou et al. 2015). Another study from Ding et al. (Ding et al. 2005) identified 16 genes responding only to a low dose of 0.02Gy in normal human skin fibroblasts (HSF42). These genes were found to be involved in cell-cell signaling, cell proliferation, signal transduction, and transcriptional regulations.

When not only considering affected pathways but also predicted downstream diseases and functions in our data, we were also able to identify pathways related to functions of cell cycle progression (Web Figure 9), likewise the study groups from Ding (Ding et al. 2005) and Hou (Hou et al. 2015). Two hours after exposure to LDIR, cell proliferation of fibroblasts was predicted to be inactivated in our results. However, the amount of inactivation was only minor (z = − 0.07). Similar results were found 4 h after exposure to LDIR. Here, DNA synthesis and cell proliferation were predicted to be inactivated. Cell cycle progression was indicated as activated at this time point. However, with a z-score of 0.56, this predicted activation is also not significant.

Due to the low number of DEGs after LDIR and therefore only limited information input, prediction of upstream regulators only showed inactivation of the tumor necrosis factor (TNF) as a predictable result (Web Figure 10). Despite that the threshold of z > |2| could not be reached here either, it appears to be a reaction that occurs shortly after the stimulus in a dose-dependent manner.

To sum up, previously conducted studies comparing different doses of radiation and time points of analyses reported on more DEGs in fibroblasts after exposure to a high than to a low dose of ionizing radiation (Hou et al. 2015) and only little overlap of expressed genes between low and high dose (Velegzhaninov et al. 2015; Mezentsev and Amundson 2011). This also applies to our study. Since the time point with the largest number of DEGs differs in published studies from 4 h (Ding et al. 2005) over 16 h (Mezentsev and Amundson 2011) to 24 h (Hou et al. 2015; Mezentsev and Amundson 2011) for different radiation doses and in different cell types, we identified 4 h after irradiation as the best point for our analysis in primary human fibroblasts. At this time point, the largest number of DEGs could be observed for both LDIR and HDIR.

Despite the conducted studies on changes in gene expression and triggered pathways in human fibroblasts after exposure to ionizing radiation, the understanding of underlying mechanisms and biological effects is still incomplete for this cell type, especially for low doses (Albrecht et al. 2012; Sokolov and Neumann 2015). Using RNA sequencing data of 15 participants to analyze underlying pathways, we were able to guide further research on radiation-related changes in gene expression. Gained results can be used to conduct radiation experiments in a larger extend and to differentiate between patient groups.

### Strengths and limitations

The present study has several strengths: Unlike previous studies using commercialy available cells (Hou et al. 2015; Velegzhaninov et al. 2015; Mezentsev and Amundson 2011; Ding et al. 2005; Jen and Cheung 2005; Ghandhi et al. 2008) or only a limited number of donors (Albrecht et al. 2012; Warters et al. 2009; Berglund et al. 2008; Goldberg et al. 2004), we used fibroblasts from skin biopsies from a total of 15 donors. All samples were cultivated for the first time and synchronized in the G_0_/G_1_ phase of the cell cycle by contact inhibition to exclude cell cycle-dependent effects on gene-expression profiles. G_0_/G_1_ arrest was confirmed by flow cytometry for all samples. To guarantee the same conditions for all of our samples, non-irradiated samples were kept and analyzed under identical conditions as irradiated ones. Pathway analysis via IPA allows analyses of complex RNA data and gives insight beyond single expressional patterns. This expands the investigational frame and adds knowledge to the overall picture of radiation biology.

Besides the mentioned strengths, the main constrains of our study are a limited number of radiation doses and time points of analysis. To identify two potent time points for our analysis, we conducted preliminary experiments with smaller sample sizes and literature research. Longer post-irradiation time points might also be interesting for subsequent pathological changes such as cancer. However, genes and pathways affected directly after exposure to ionizing radiation (immediate early genes) are also assumed to affect long term radiation-induced outcomes (Averbeck et al. 2020). Regarding dose, a high and a low radiation dose with clinical relevance (Seidlitz et al. 2017; Pearce et al. 2012; Averbeck et al. 2020) were chosen to mimic characteristic exposures to ionizing radiation used in medical diagnostics and radiation therapy. In addition, we choose to analyze samples from all 3 patient groups (SPN, FPN, cancer-free controls) of the KiKme study. This might increase the heterogeneity of gene expression levels. However, expressional variability that may be introduced to the analysis by gender, age, and FPN diagnosis was accounted for in matching for these factors. Moreover, regarding the long-term goal of the KiKme study, it was important to include samples of all 3 patient groups into the analysis of this work, since differential gene expression might differ between the groups. A comparison between groups will be conducted in a subsequent study with an increased sample size and therefore more statistical power. Here, the preliminary analysis indicated no relevant differences between unadjusted and adjusted models.

## Conclusions and outlook

In this work, we detected different patterns of DEGs after exposure to LDIR and HDIR in radiation experiments with primary human fibroblasts from 15 participants from the KiKme study. Besides changes in expression patterns of single genes, expression patterns of related pathways were altered as well. We observed a shift from DNA damage-associated towards metabolism-related genes and associated pathways. The choice of the time point with the best fit for the expressional analysis of irradiation was a key task of this study. While several time points have been used in the literature our results suggest that measurement of gene expression is best done at 4 h after irradiation. At this time point, the largest effect on differential gene expression has been observed. Therefore, all subsequent experiments of the large molecular-epidemiological study KiKme will use the time point 4 h to identify differences in genetic predispositions and gene-radiation interactions between former childhood cancer patients and cancer-free controls.

## Supplementary information

**Additional file 1: Web Figure 1.** Representative measurements of the cell cycle distribution of HOECHST33258-stained fibroblasts by flow cytometry during (A) log-phase growth or (B) after G0/1 synchronization over 14 days for radiation experiments. **Web Figure 2.** Total number of differentially expressed genes in human fibroblasts from cancer free-controls at 0.25 h, 2 h and 24 h after exposure to low (0.05 Gray (Gy)) or high dose (2Gy) of X-rays compared to unirradiated fibroblasts (*N* = 3). **Web Figure 3.** Correlation of RNA quality metrics (RIN, Qbit RNA-concentration), expression variation (PC1–3) and number of aligned reads (aligned reads, aligned reads normalized) for all experiments. The color indicates the sequencing run (red = run 1, blue = run 2). **Web Figure 4.** Relative expression of *Cyclin-Dependent Kinase Inhibitor 1A* (*CDKN1A*) in Real-Time Quantitative Polymerase-Chain-Reaction (qPCR) analyzing the expression of *CDKN1A* in fibroblasts of 6 participants 2 h and 4 h after exposure to 0.05 Gray (Gy) or 2Gy ionizing radiation compared to sham-irradiated samples (0Gy, reference). *** *p* < 0.001. **Web Figure 5.** Relative expression of *Mouse double minute 2 homolog* (*MDM2*) in Real-Time Quantitative Polymerase-Chain-Reaction (qPCR) analyzing the expression of *MDM2* in fibroblasts of 6 participants 2 h and 4 h after exposure to 0.05 Gray (Gy) or 2Gy ionizing radiation compared to sham-irradiated samples (0Gy, reference). *** *p* < 0.001. **Web Figure 6.** Expression variation in fibroblasts summarized for all experiments and attributed to time point post irradiation (circle = 2 h, cross = 4 h) and dose (orange = 0 Gray (Gy), blue = 0.05Gy, green = 2Gy).

**Additional file 2: Web Figure 7.** Shared pathways from low and high dose ionizing radiation experiments. Gy = Gray. **Web Figure 8.** Pathways only affected in high dose ionizing radiation experiments. Gy = Gray.

**Additional file 3: Web Figure 9.** Predicted downsteam diseases and functions. **Web Figure 10.** Predicted upstream regulators. LDIR = Low dose of ionizing radiation (0.05 Gray), HDIR = High dose of ionizing radiation (2 Gray).

**Additional file 4: Web Figure 11.** Comparison of affected pathways in different data sets.

**Additional file 5: Web Figure 12.** Comparison of predicted downstream diseases and functions in different data sets.

**Additional file 6: Web Figure 13.** Comparison of predicted upstream regulators in different data sets.

**Additional file 7:** Gene expression in the "Not a Number" pathways (blue = downregulation, red = upregulation). **Web Figure 14.** *Base excision repair (*BER*) system*. **Web Fig. 15.** *Molecular mechanisms of cancer*. **Web Fig. 16.** *Assembly of RNA polymerase III complex*. **Web Fig. 17.** *DNA double-strand break repair by homologous recombination*. **Web Fig. 18.** *Interleukin 4 (IL-4) signaling*. **Web Fig. 19.** *Interleukin 17 (IL-17) signaling*. **Web Fig. 20.** *Interleukin 17A (IL-17A) signaling in fibroblasts*. **Web Fig. 21.** *Mitochondrial dysfunction*. **Web Fig. 22.** *Myc mediated apoptosis signaling*. **Web Fig. 23.***Nucleotide excision repair*. **Web Fig. 24.** *Protein ubiquitination*. **Web Fig. 25.** *Retinoic acid receptor (RAR) activation*. **Web Fig. 26.** *Role of Janus kinase 2 (JAK2) in hormone-like cytokine signaling*. **Web Fig. 27.** *Role of Janus kinase (JAK) family kinases in Interleukin 6 (IL-6) type cytokine signaling*. **Web Fig. 28.** *Tight junction signaling*.

**Additional file 8: Web Table 1A.** Differentially expressed genes 2 h after exposure to low dose ionizing radiation (0.05 Gray).

**Additional file 9: Web Table 1B.** Differentially expressed genes 4 h after exposure to low dose ionizing radiation (0.05 Gray).

**Additional file 10: Web Table 1C.** Differentially expressed genes 2 h after exposure to high dose ionizing radiation (2 Gray).

**Additional file 11: Web Table 1D.** Differentially expressed genes 4 h after exposure to high dose ionizing radiation (2 Gray).

**Additional file 12: Web Table 2.** Differential expression activity in cellular pathways and involved molecules

**Additional file 13: Supplement file 1.** Settings for comparison analyses in IPA.

## Data Availability

The datasets generated and analyzed during the current study are not publicly available due to ethic and data protection reasons but are available from the corresponding author on reasonable request.

## Abbreviations

*BER*: *Base excision repair*
*CDKN1A*: *Cyclin-Dependent Kinase Inhibitor 1A*
DEG: Differentially expressed gene
DNA: Deoxyribonucleic acid
FPN: First primary neoplasm
*Gαq*: *Gq protein alpha subunit*
Gy: Gray
H: Hour
HDIR: High dose ionizing radiation
IPA: Ingenuity Pathway Analysis
LDIR: Low dose ionizing radiation
*MDM2*: *Mouse double minute 2 homolog*
*mTOR*: *Mechanistic Target of Rapamycin*
*NF-κB*: *Nuclear factor* *kappa-light-chain**-enhancer of activated B cells*
qPCR: Real-Time Quantitative Polymerase-Chain-Reaction
*PI3K*: *Phosphoinositide* *3-kinase*
*POLB*: *DNA polymerase beta*
*PUMA*: *P53 upregulated modulator of apoptosis*
RIN: RNA integrity number
RNA: Ribonucleic acid
SPN: Second primary neoplasm
