# Supplementary material for: Comparison of time and dose dependent gene expression and affected pathways in primary human fibroblasts after exposure to ionizing radiation
Source: Mol Med. 2020 Sep 9;26:85. doi: 10.1186/s10020-020-00203-0 (PMC7488023; doi:10.1186/s10020-020-00203-0)
Supplement: Supplementary file 1 — Additional file 1: Web Figure 1. Representative measurements of the cell cycle distribution of HOECHST33258-stained fibroblasts by flow cytometry during (A) log-phase growth or (B) after G0/1 synchronization over 14 days for radiation experiments. Web Figure 2. Total number of differentially expressed genes in human fibroblasts from cancer free-controls at 0.25 h, 2 h and 24 h after exposure to low (0.05 Gray (Gy)) or high dose (2Gy) of X-rays compared to unirradiated fibroblasts (N = 3). Web Figure 3. Correlation of RNA quality metrics (RIN, Qbit RNA-concentration), expression variation (PC1–3) and number of aligned reads (aligned reads, aligned reads normalized) for all experiments. The color indicates the sequencing run (red = run 1, blue = run 2). Web Figure 4. Relative expression of Cyclin-Dependent Kinase Inhibitor 1A (CDKN1A) in Real-Time Quantitative Polymerase-Chain-Reaction (qPCR) analyzing the expression of CDKN1A in fibroblasts of 6 participants 2 h and 4 h after exposure to 0.05 Gray (Gy) or 2Gy ionizing radiation compared to sham-irradiated samples (0Gy, reference). *** p < 0.001. Web Figure 5. Relative expression of Mouse double minute 2 homolog (MDM2) in Real-Time Quantitative Polymerase-Chain-Reaction (qPCR) analyzing the expression of MDM2 in fibroblasts of 6 participants 2 h and 4 h after exposure to 0.05 Gray (Gy) or 2Gy ionizing radiation compared to sham-irradiated samples (0Gy, reference). *** p < 0.001. Web Figure 6. Expression variation in fibroblasts summarized for all experiments and attributed to time point post irradiation (circle = 2 h, cross = 4 h) and dose (orange = 0 Gray (Gy), blue = 0.05Gy, green = 2Gy). [file 10020_2020_203_MOESM1_ESM.docx]

**
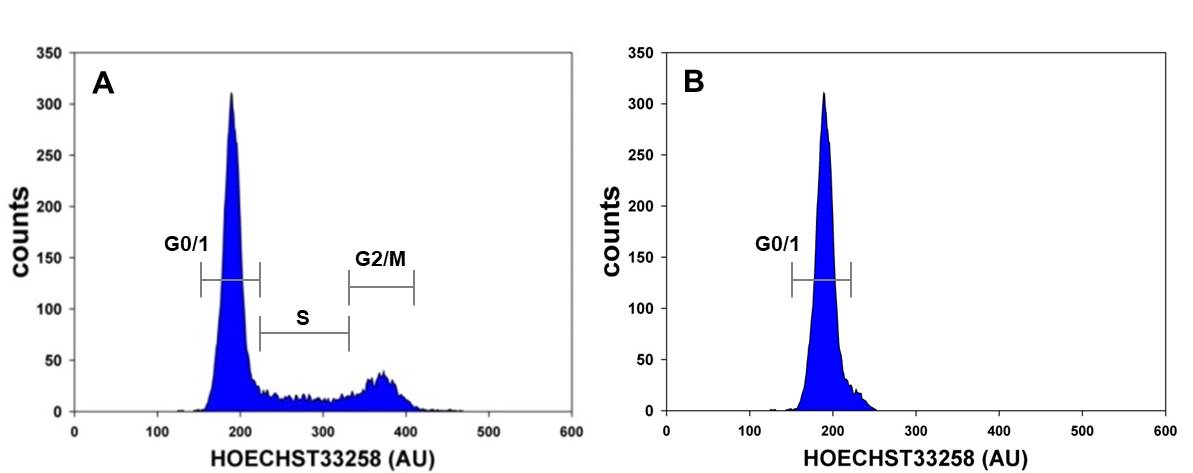
**

**Web Figure 1:** Representative measurements of the cell cycle distribution of HOECHST33258-stained fibroblasts by flow cytometry during (A) log-phase growth or (B) after G0/1 synchronization over 14 days for radiation experiments.

**
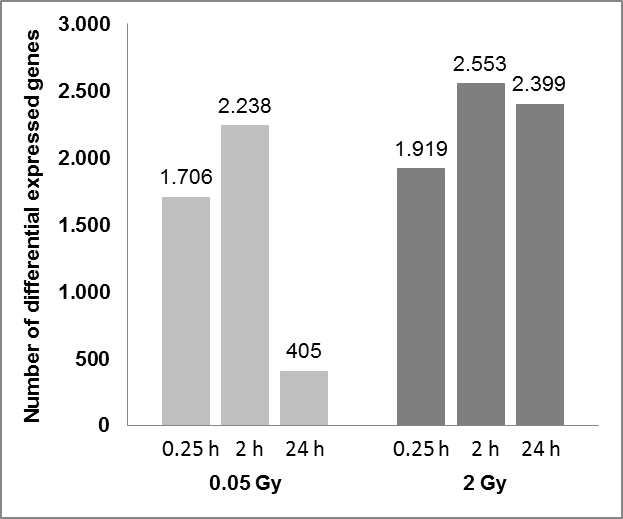
**

**Web Figure 2:** Total number of differentially expressed genes in human fibroblasts from cancer free-controls at 0.25h, 2h and 24h after exposure to low (0.05 Gray (Gy)) or high dose (2Gy) of X-rays compared to unirradiated fibroblasts (N = 3).

**
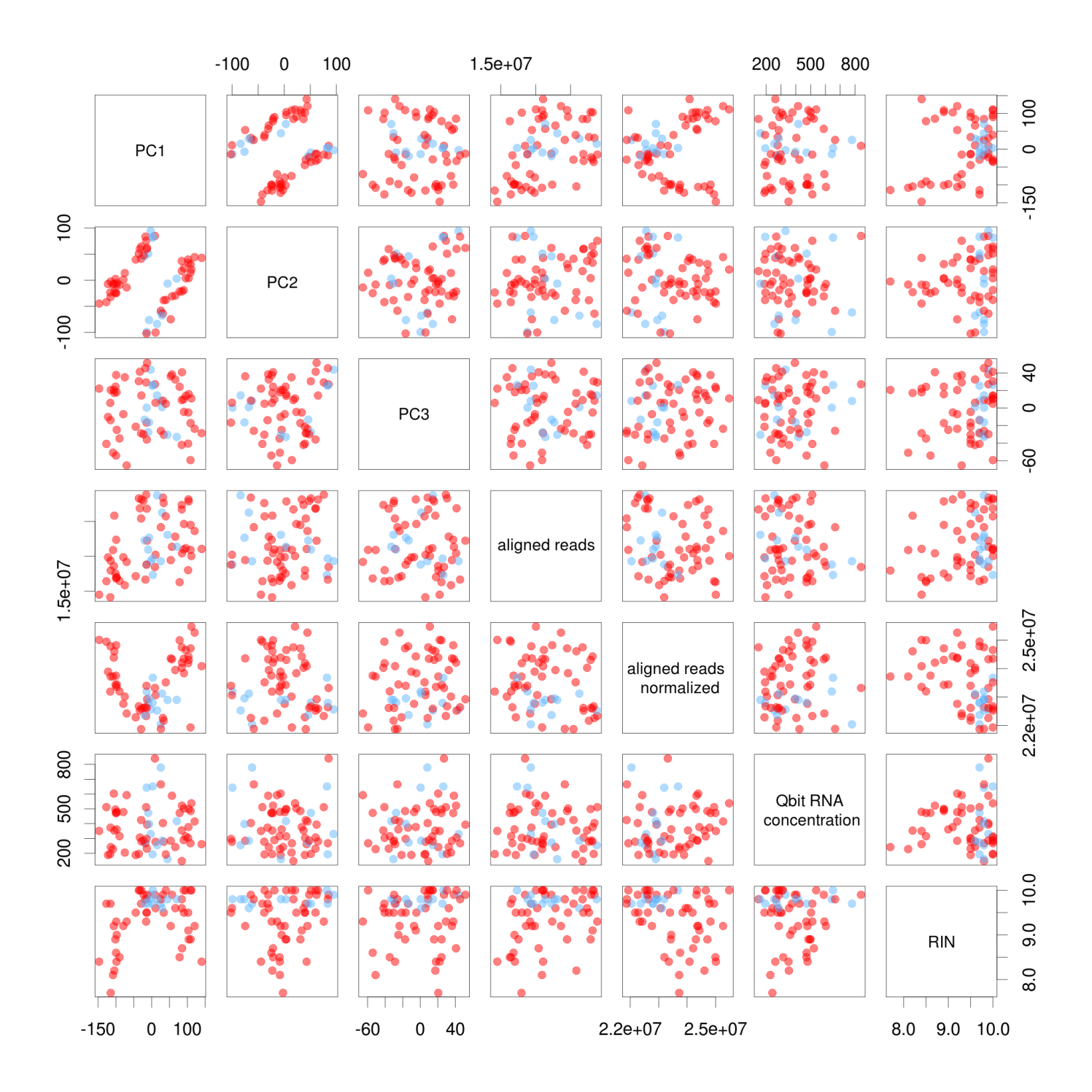
**

**Web Figure 3:** Correlation of RNA quality metrics (RIN, Qbit RNA-concentration), expression variation (PC1-3) and number of aligned reads (aligned reads, aligned reads normalized) for all experiments. The color indicates the sequencing run (red = run 1, blue = run 2).

**
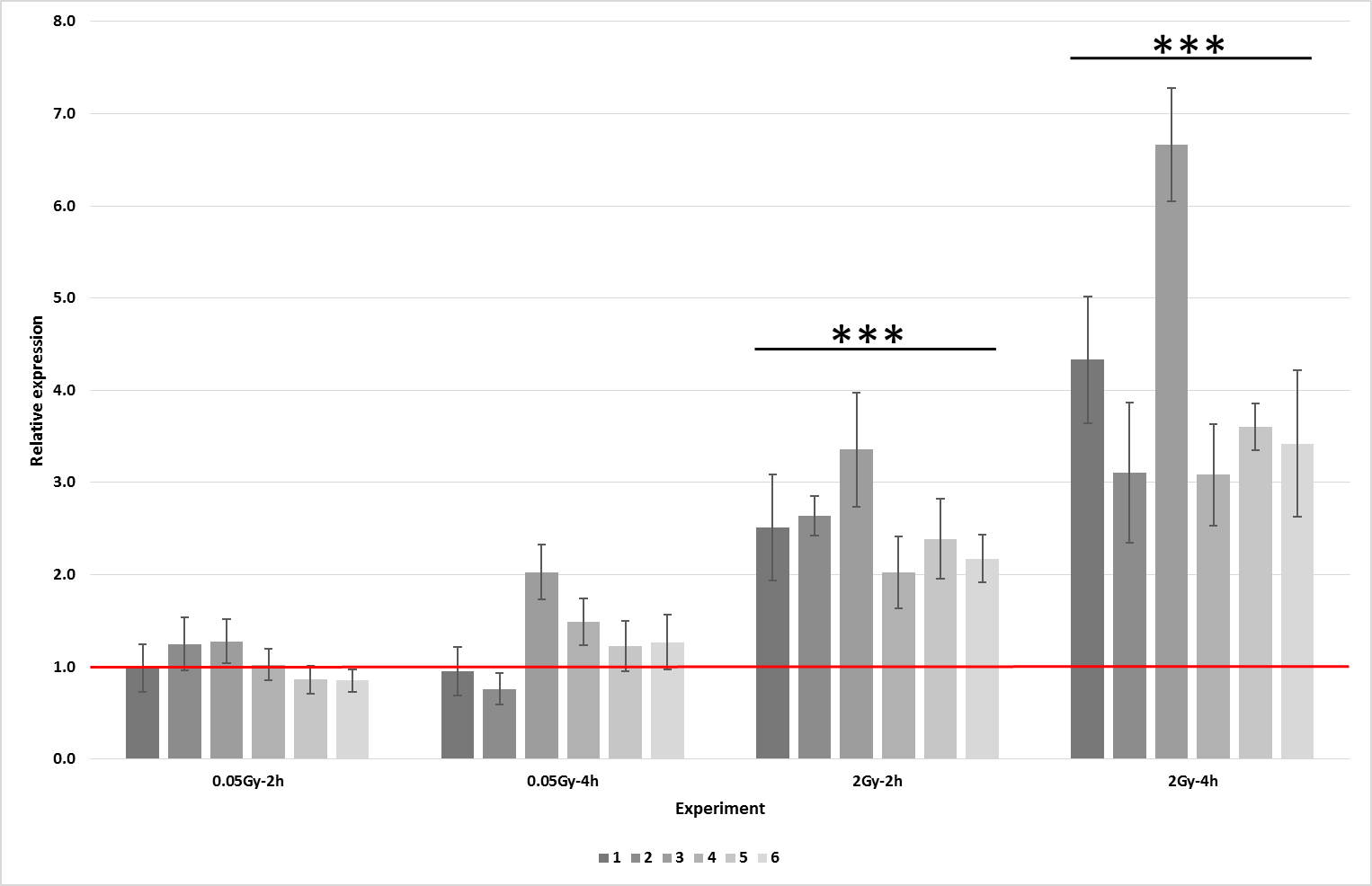
Web Figure 4:** Relative expression of *Cyclin-Dependent Kinase Inhibitor 1A* (*CDKN1A*) in Real-Time Quantitative Polymerase-Chain-Reaction (qPCR) analyzing the expression of *CDKN1A* in fibroblasts of 6 participants 2h and 4h after exposure to 0.05 Gray (Gy) or 2Gy ionizing radiation compared to sham-irradiated samples (0Gy, reference). *** p < 0.001.

**
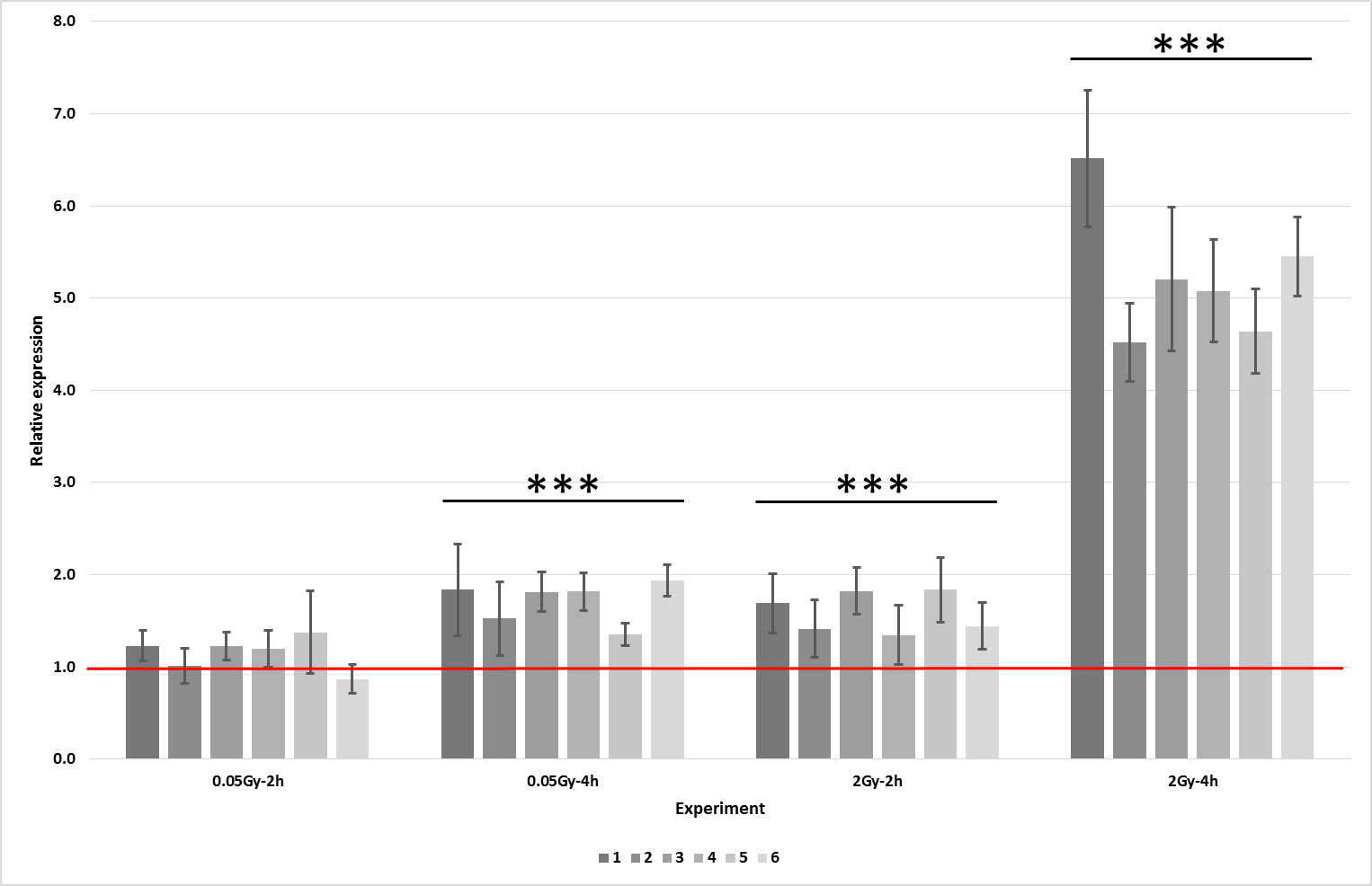
Web Figure 5:** Relative expression of *Mouse double minute 2 homolog* (*MDM2*) in Real-Time Quantitative Polymerase-Chain-Reaction (qPCR) analyzing the expression of *MDM2* in fibroblasts of 6 participants 2h and 4h after exposure to 0.05 Gray (Gy) or 2Gy ionizing radiation compared to sham-irradiated samples (0Gy, reference). *** p < 0.001.


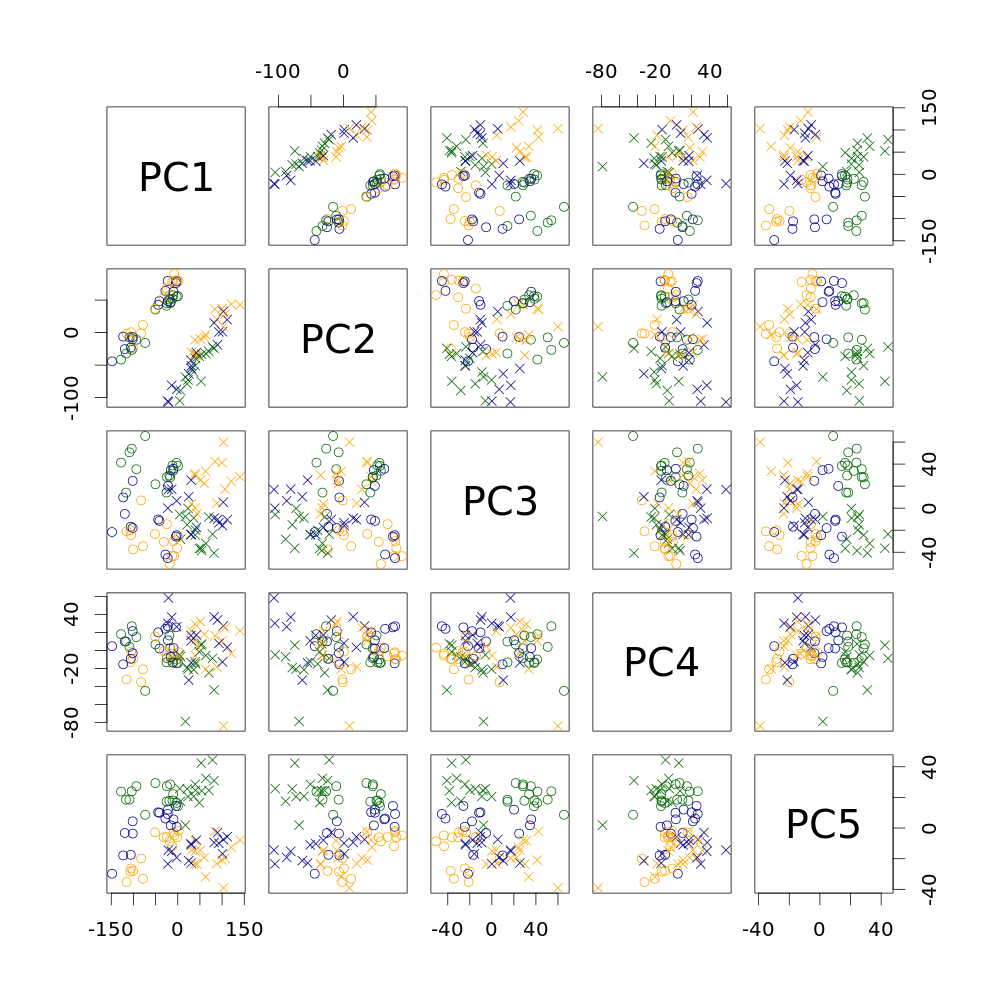


Web Figure 6: Expression variation in fibroblasts summarized for all experiments and attributed to time point post irradiation (circle = 2h, cross= 4h) and dose (orange = 0 Gray (Gy), blue = 0.05Gy, green = 2Gy).
