# Supplementary material for: Comparison of time and dose dependent gene expression and affected pathways in primary human fibroblasts after exposure to ionizing radiation
Source: Mol Med. 2020 Sep 9;26:85. doi: 10.1186/s10020-020-00203-0 (PMC7488023; doi:10.1186/s10020-020-00203-0)
Supplement: Supplementary file 2 — Additional file 2: Web Figure 7. Shared pathways from low and high dose ionizing radiation experiments. Gy = Gray. Web Figure 8. Pathways only affected in high dose ionizing radiation experiments. Gy = Gray. [file 10020_2020_203_MOESM2_ESM.pdf]

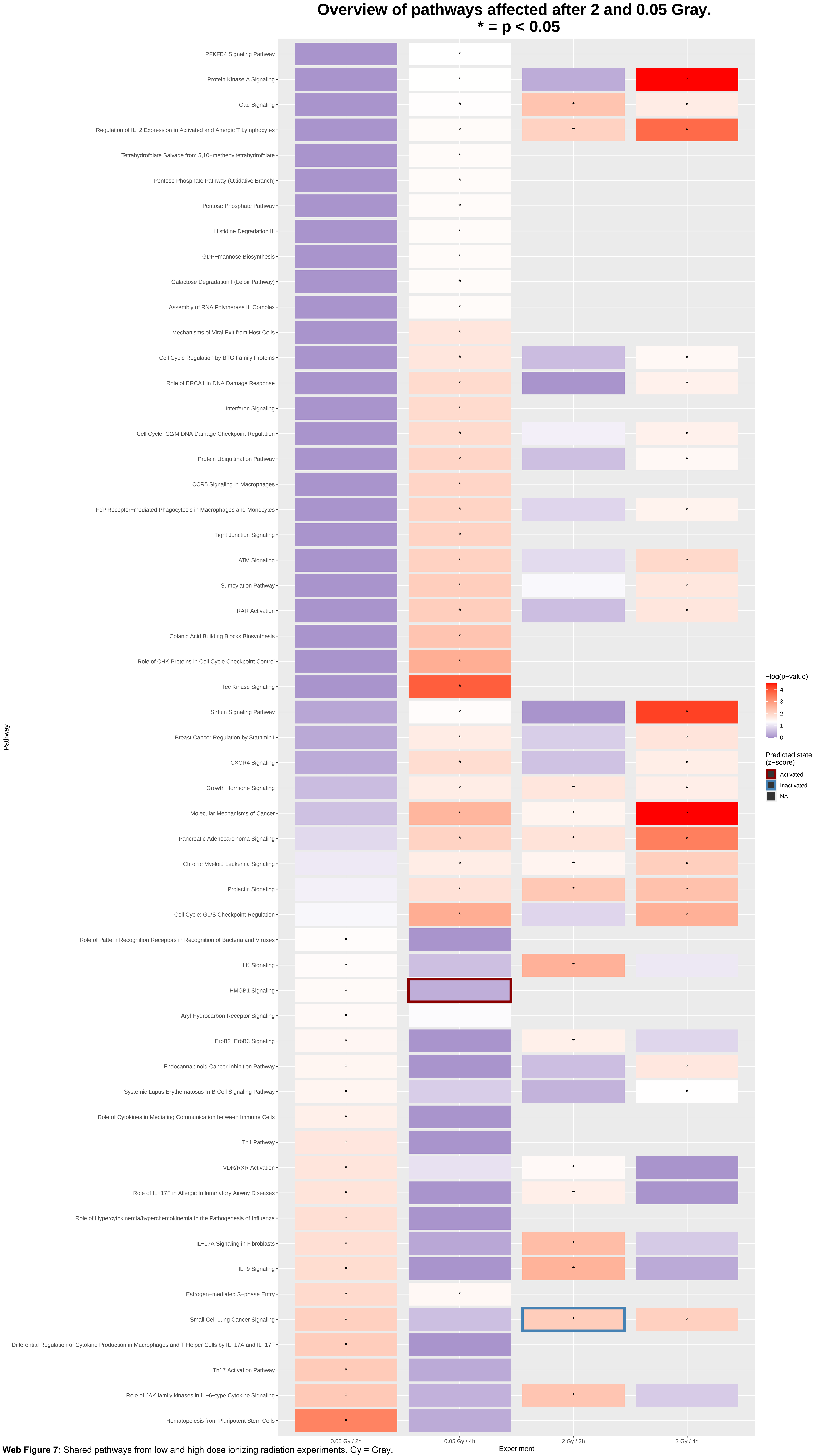

Web Figure 7: Shared pathways from low and high dose ionizing radiation experiments. Gy = Gray.

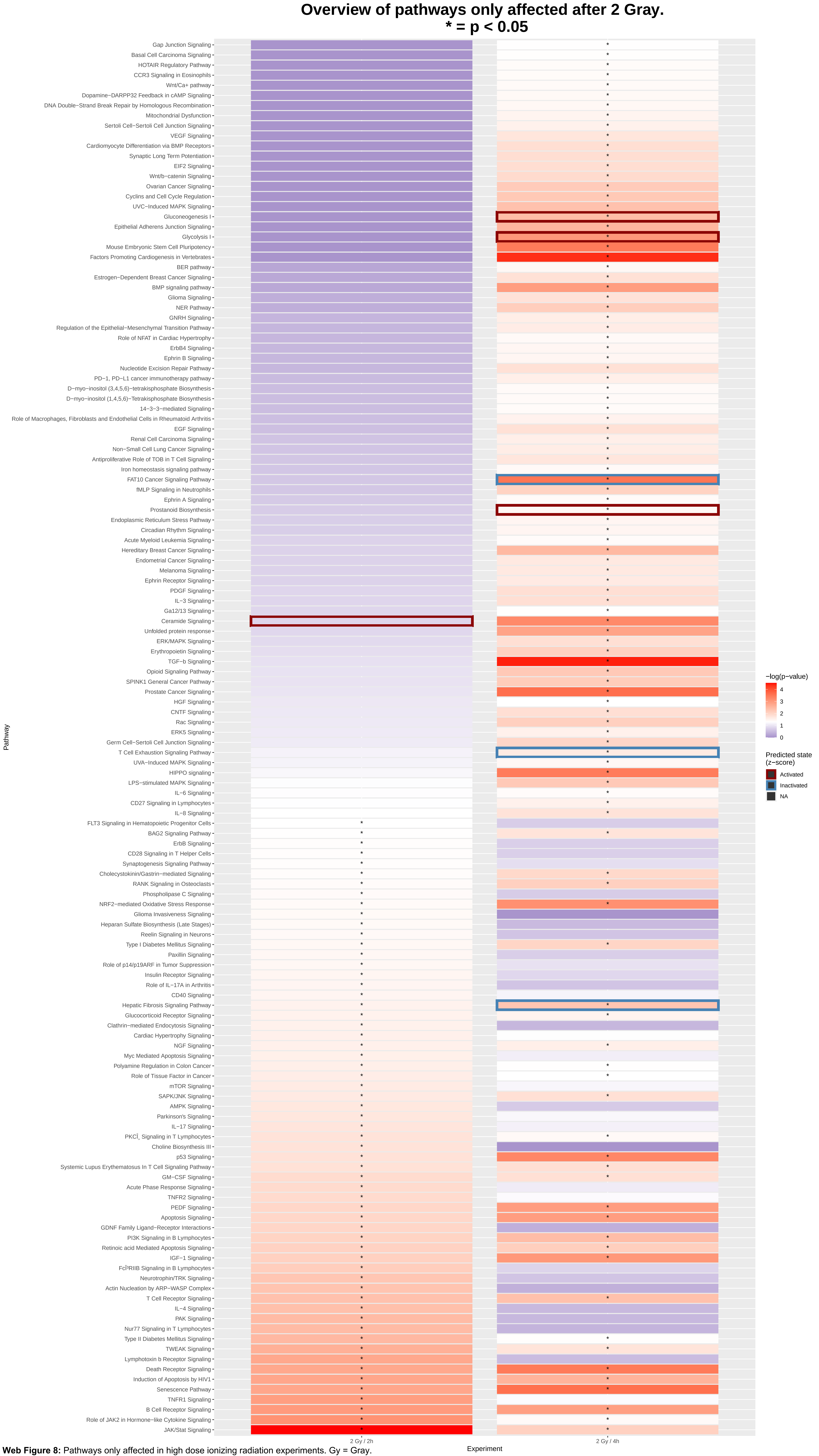

Web Figure 8: Pathways only affected in high dose ionizing radiation experiments. Gy = Gray.
