# Supplementary material for: Comparison of time and dose dependent gene expression and affected pathways in primary human fibroblasts after exposure to ionizing radiation
Source: Mol Med. 2020 Sep 9;26:85. doi: 10.1186/s10020-020-00203-0 (PMC7488023; doi:10.1186/s10020-020-00203-0)
Supplement: Supplementary file 3 — Additional file 3: Web Figure 9. Predicted downsteam diseases and functions. Web Figure 10. Predicted upstream regulators. LDIR = Low dose of ionizing radiation (0.05 Gray), HDIR = High dose of ionizing radiation (2 Gray). [file 10020_2020_203_MOESM3_ESM.docx]

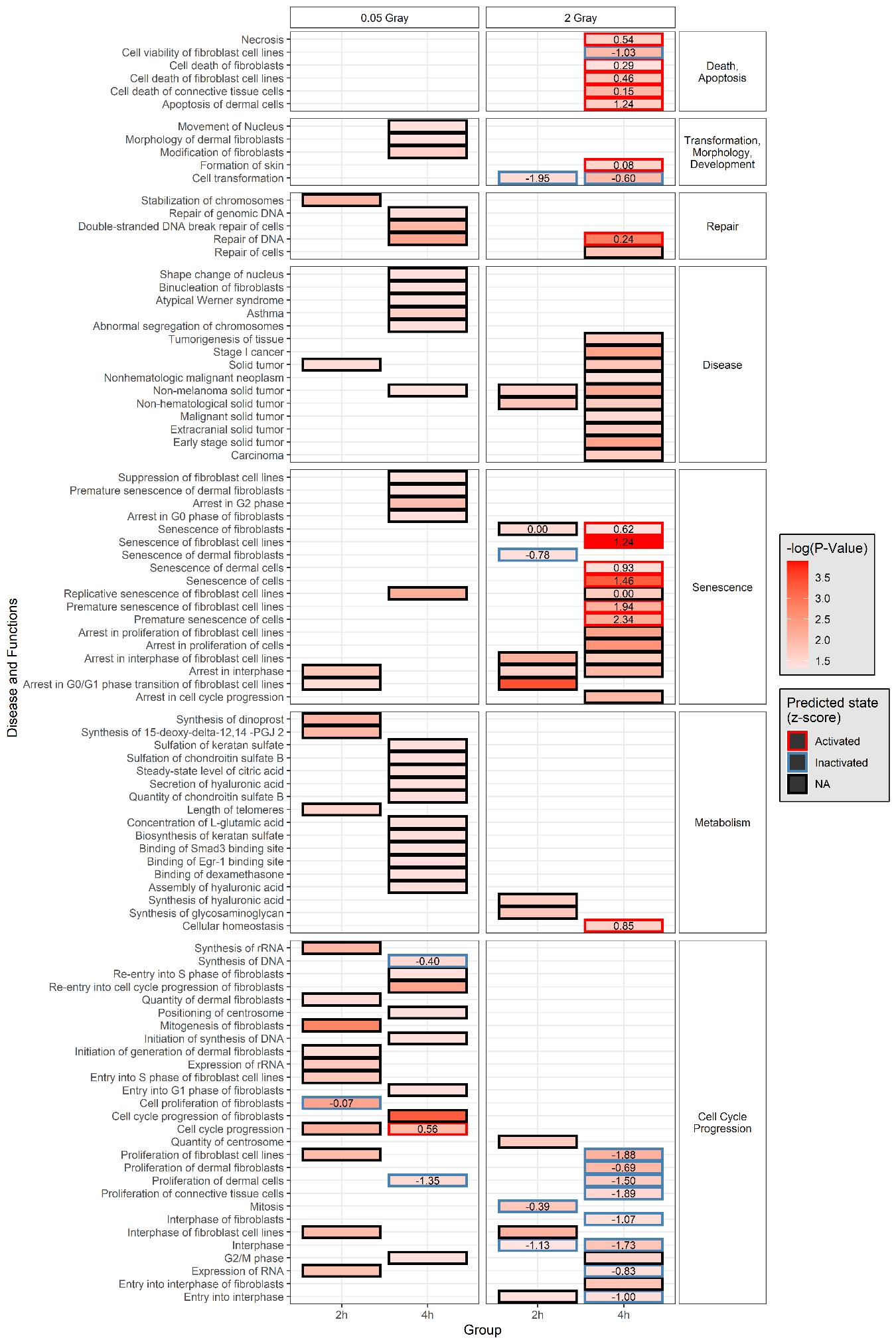


**Web Figure 9:** Predicted downsteam diseases and functions.


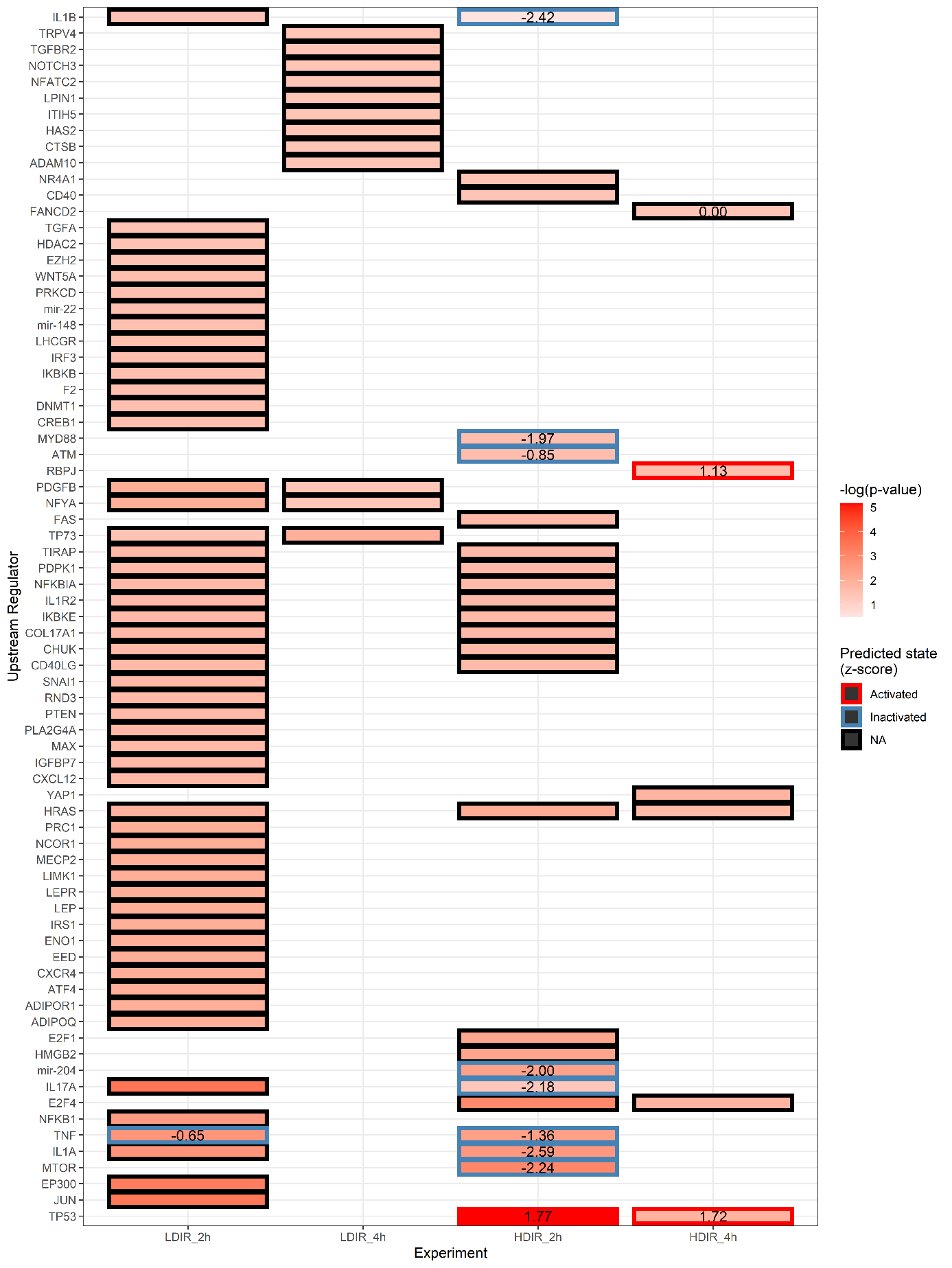


Web Figure 10: Predicted upstream regulators. LDIR = Low dose of ionizing radiation (0.05 Gray), HDIR = High dose of ionizing radiation (2 Gray).
