# Supplementary figures and images for: Comparison of time and dose dependent gene expression and affected pathways in primary human fibroblasts after exposure to ionizing radiation
Source: Mol Med. 2020 Sep 9;26:85. doi: 10.1186/s10020-020-00203-0 (PMC7488023; doi:10.1186/s10020-020-00203-0)

Web Figure 11: Comparison of affected pathways in different data sets.

p < 0.05

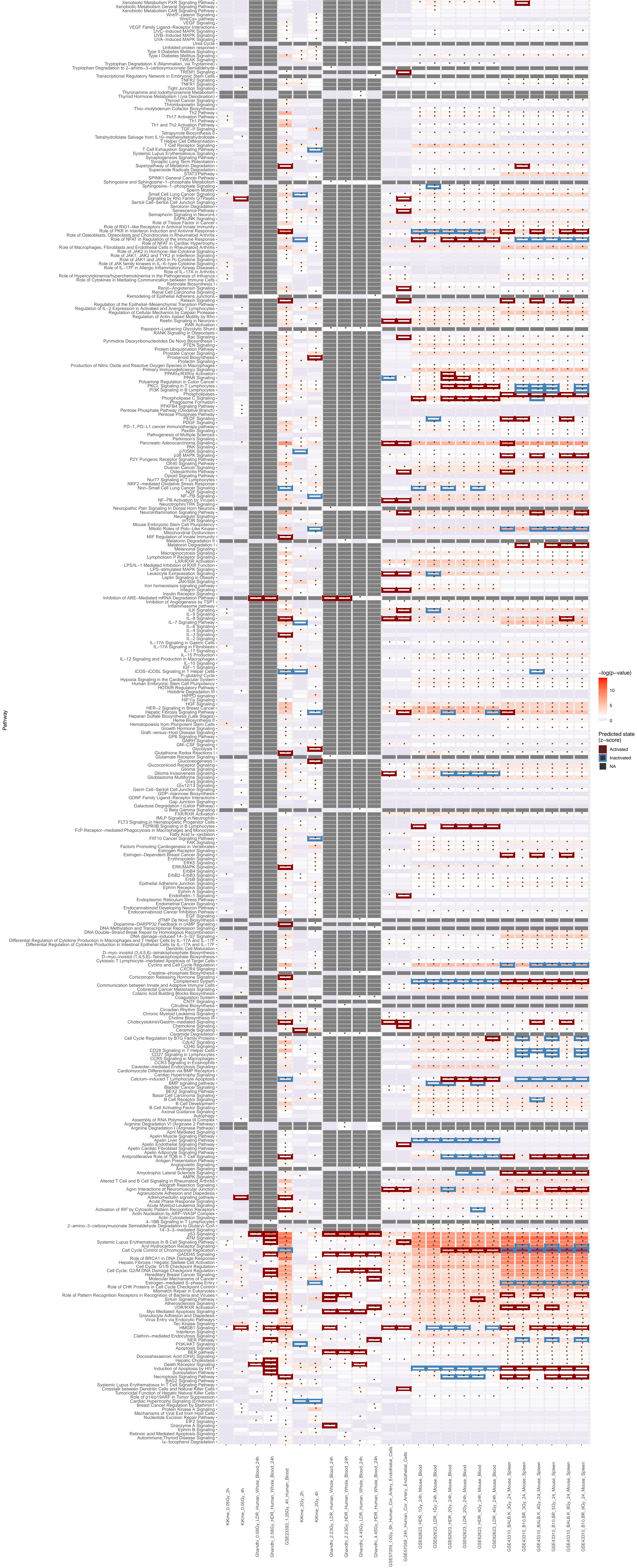

Supplement: Supplementary file 4 — Additional file 4: Web Figure 11. Comparison of affected pathways in different data sets. [file 10020_2020_203_MOESM4_ESM.pdf]
