# Supplementary material for: Comparison of time and dose dependent gene expression and affected pathways in primary human fibroblasts after exposure to ionizing radiation
Source: Mol Med. 2020 Sep 9;26:85. doi: 10.1186/s10020-020-00203-0 (PMC7488023; doi:10.1186/s10020-020-00203-0)
Supplement: Supplementary file 7 — Additional file 7: Gene expression in the "Not a Number" pathways (blue = downregulation, red = upregulation). Web Figure 14. Base excision repair (BER) system. Web Fig. 15. Molecular mechanisms of cancer. Web Fig. 16. Assembly of RNA polymerase III complex. Web Fig. 17. DNA double-strand break repair by homologous recombination. Web Fig. 18. Interleukin 4 (IL-4) signaling. Web Fig. 19. Interleukin 17 (IL-17) signaling. Web Fig. 20. Interleukin 17A (IL-17A) signaling in fibroblasts. Web Fig. 21. Mitochondrial dysfunction. Web Fig. 22. Myc mediated apoptosis signaling. Web Fig. 23.Nucleotide excision repair. Web Fig. 24. Protein ubiquitination. Web Fig. 25. Retinoic acid receptor (RAR) activation. Web Fig. 26. Role of Janus kinase 2 (JAK2) in hormone-like cytokine signaling. Web Fig. 27. Role of Janus kinase (JAK) family kinases in Interleukin 6 (IL-6) type cytokine signaling. Web Fig. 28. Tight junction signaling. [file 10020_2020_203_MOESM7_ESM.docx]

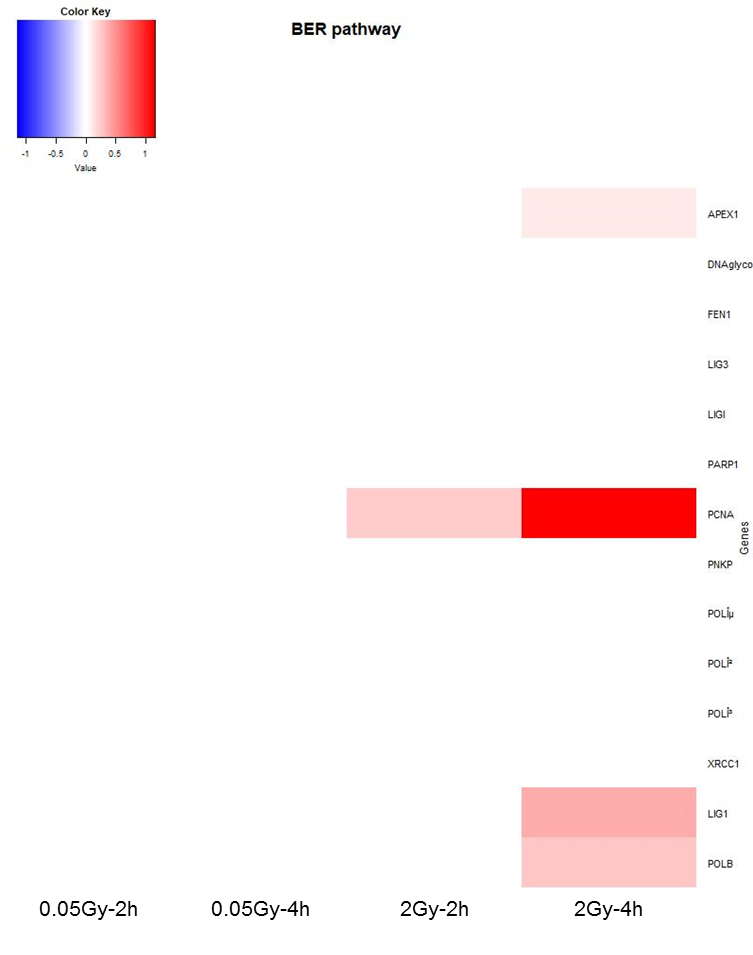


Web Figure 14: Gene expression in the “Not a Number” pathway *base excision repair (BER) system* (blue = downregulation, red: upregulation), Gy = Gray.


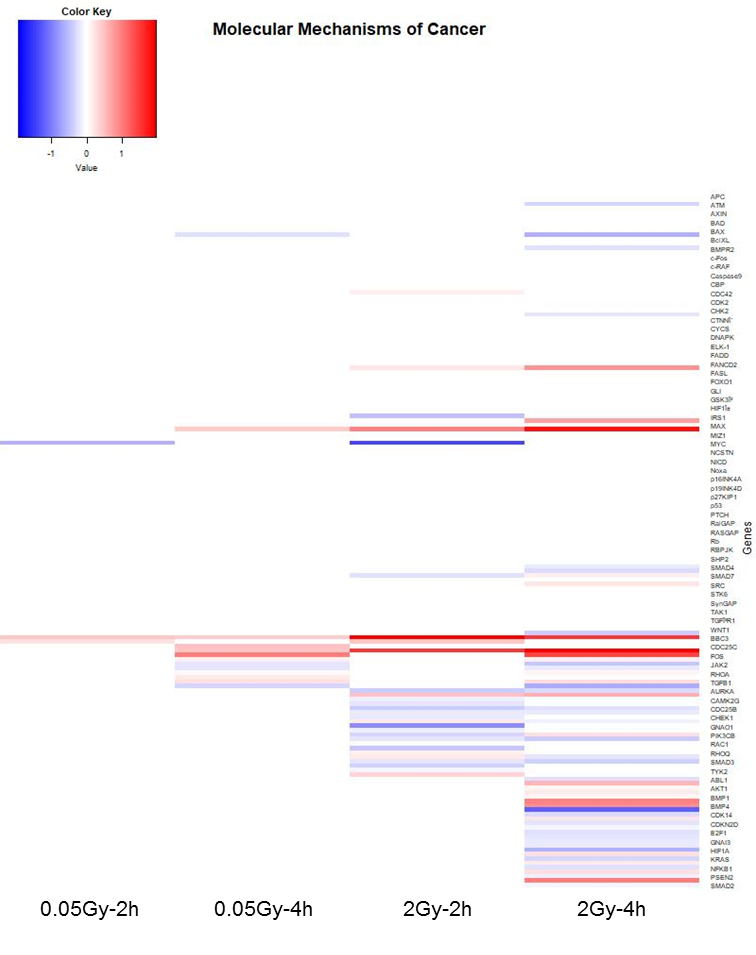


Web Figure 15: Gene expression in the “Not a Number” pathway *molecular mechanisms of cancer* (blue = downregulation, red: upregulation), Gy = Gray.


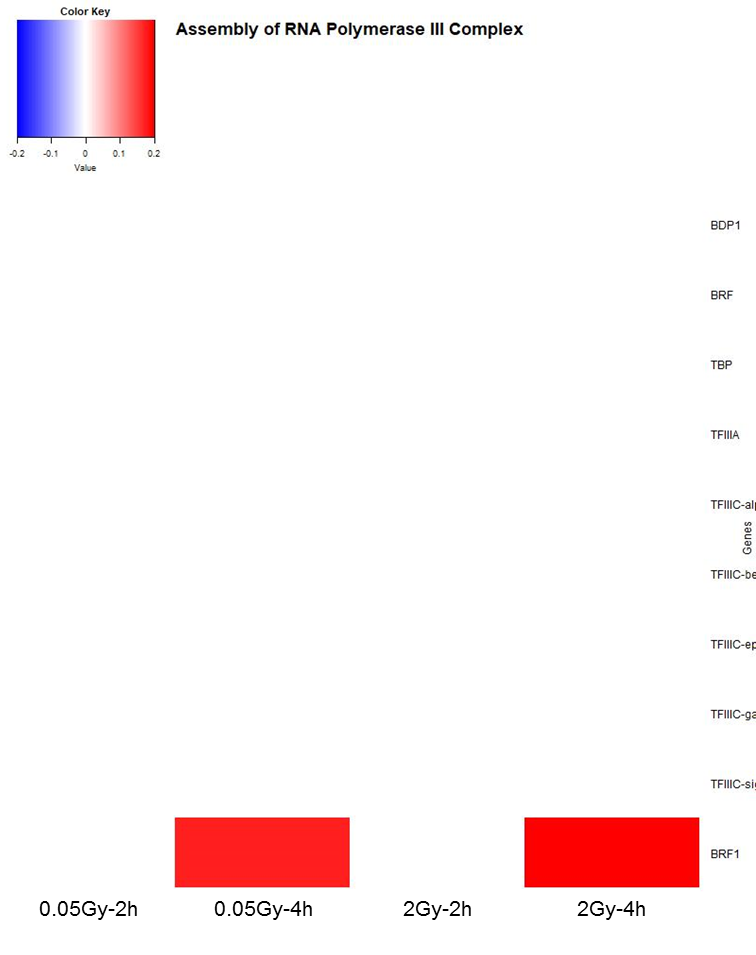


Web Figure 16: Gene expression in the “Not a Number” pathway *assembly of RNA polymerase III complex* (blue = downregulation, red: upregulation), Gy = Gray.


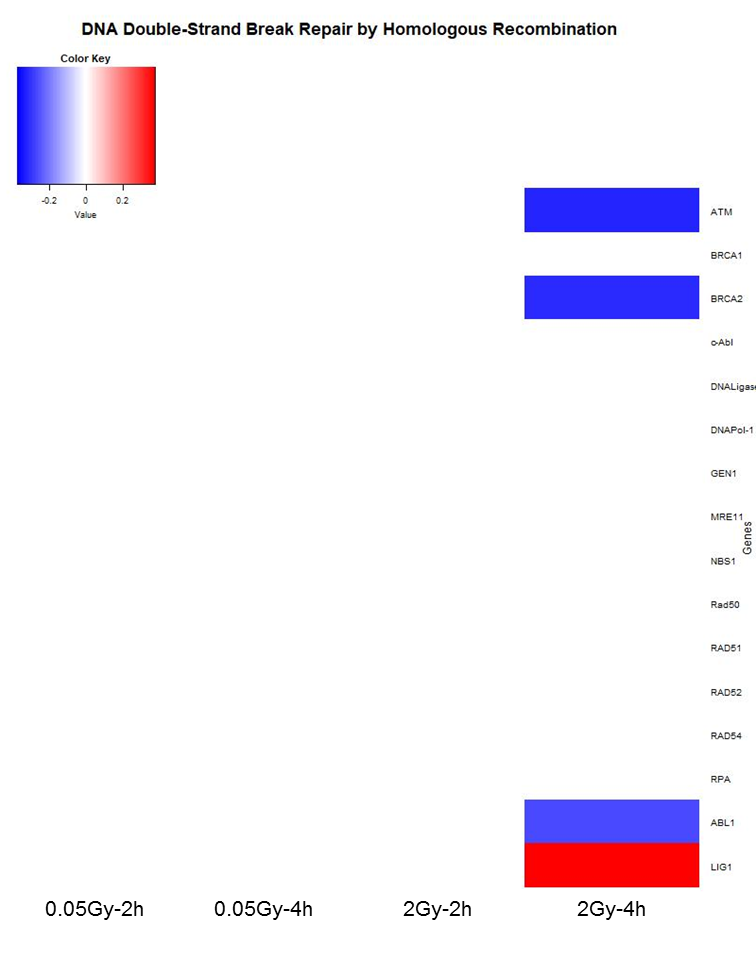


Web Figure 17: Gene expression in the “Not a Number” pathway *DNA double-strand break repair by homologous recombination* (blue = downregulation, red: upregulation), Gy = Gray.


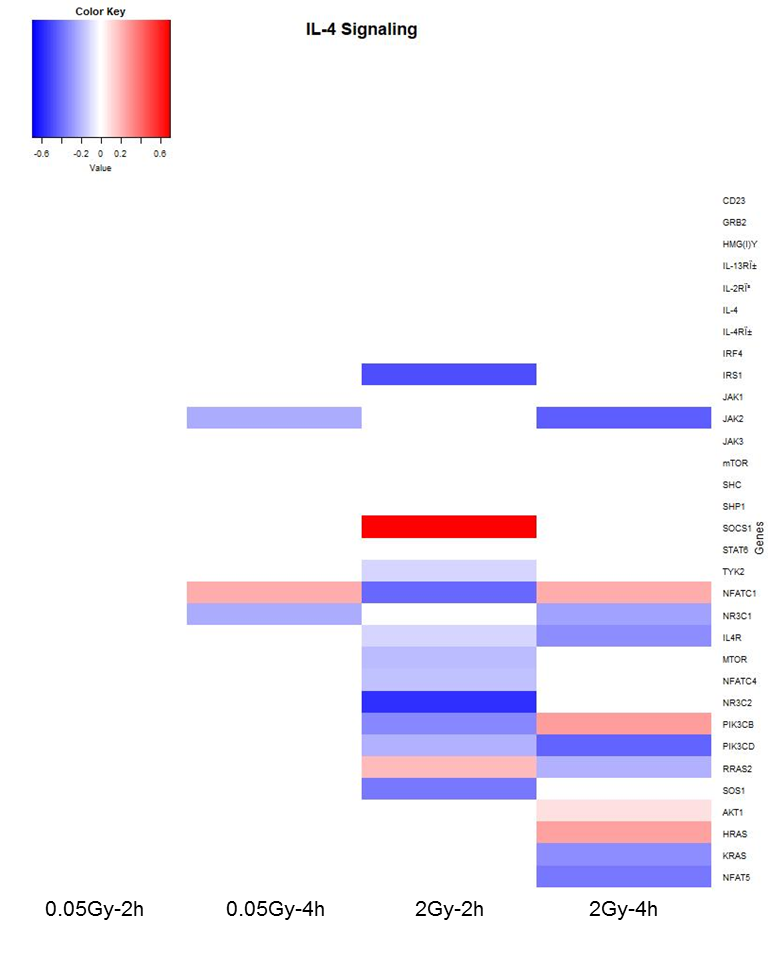


Web Figure 18: Gene expression in the “Not a Number” pathway *Interleukin 4 (IL-4) signaling* (blue = downregulation, red: upregulation), Gy = Gray.


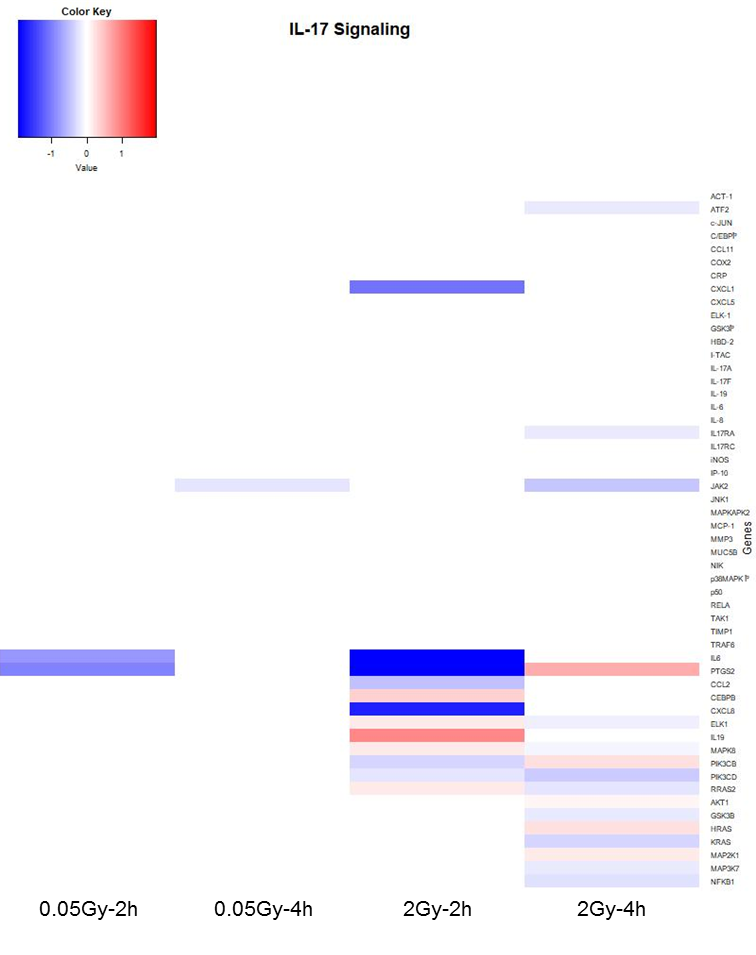


Web Figure 19: Gene expression in the “Not a Number” pathway *Interleukin 17 (IL-17) signaling* (blue = downregulation, red: upregulation), Gy = Gray.


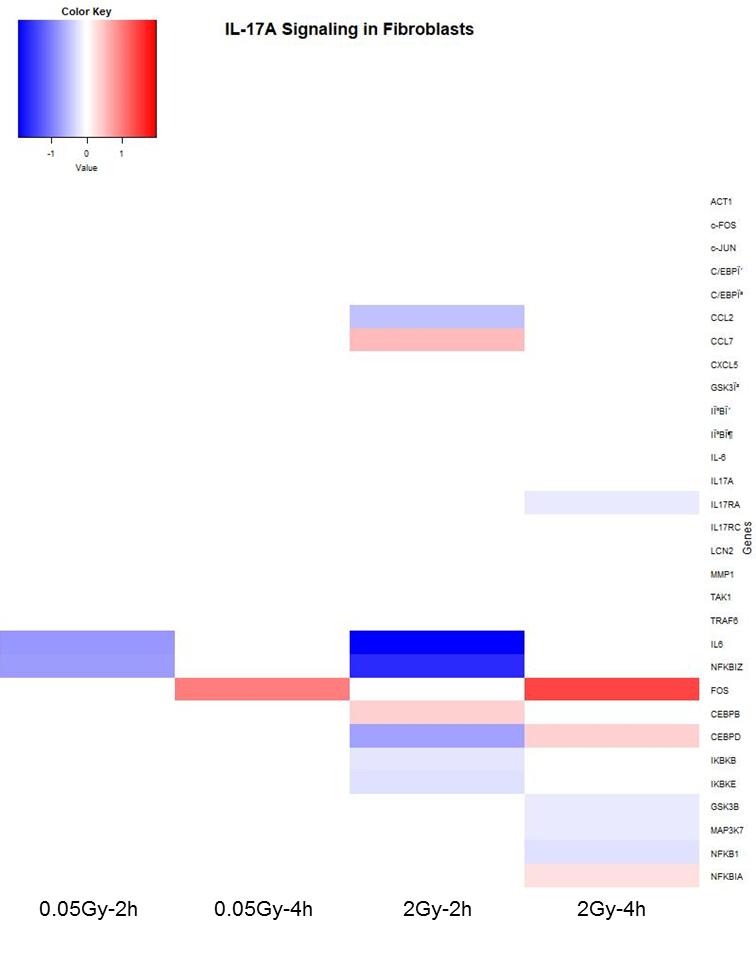


Web Figure 20: Gene expression in the “Not a Number” pathway *Interleukin 17A (IL-17A) signaling in fibroblasts* (blue = downregulation, red: upregulation), Gy = Gray.


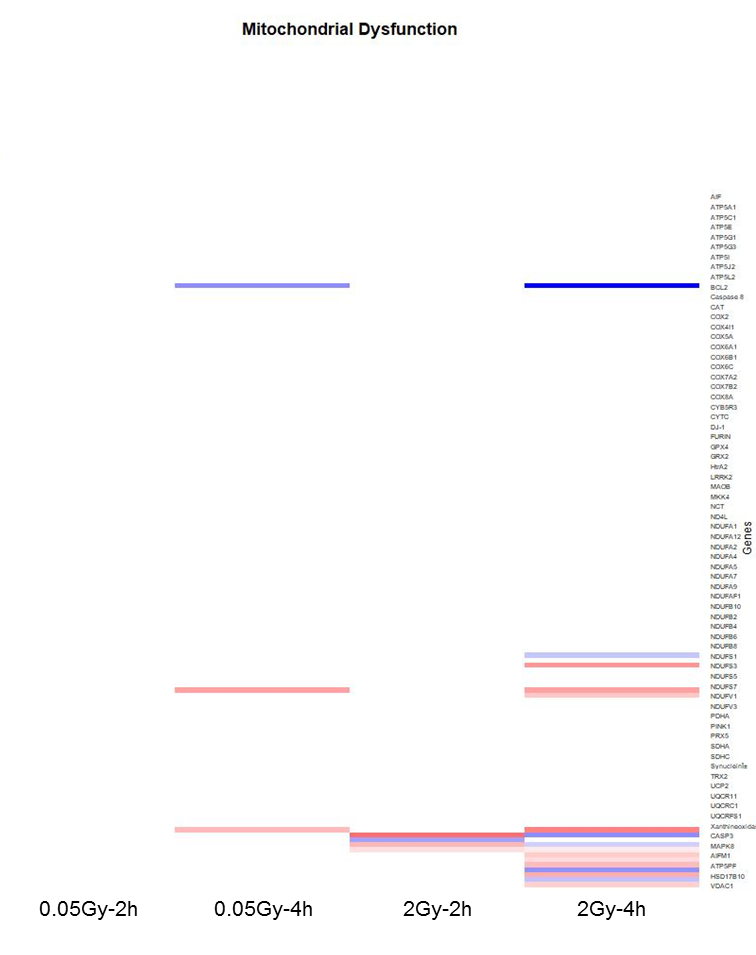


Web Figure 21: Gene expression in the “Not a Number” pathway *mitochondrial dysfunction* (blue = downregulation, red: upregulation), Gy = Gray.


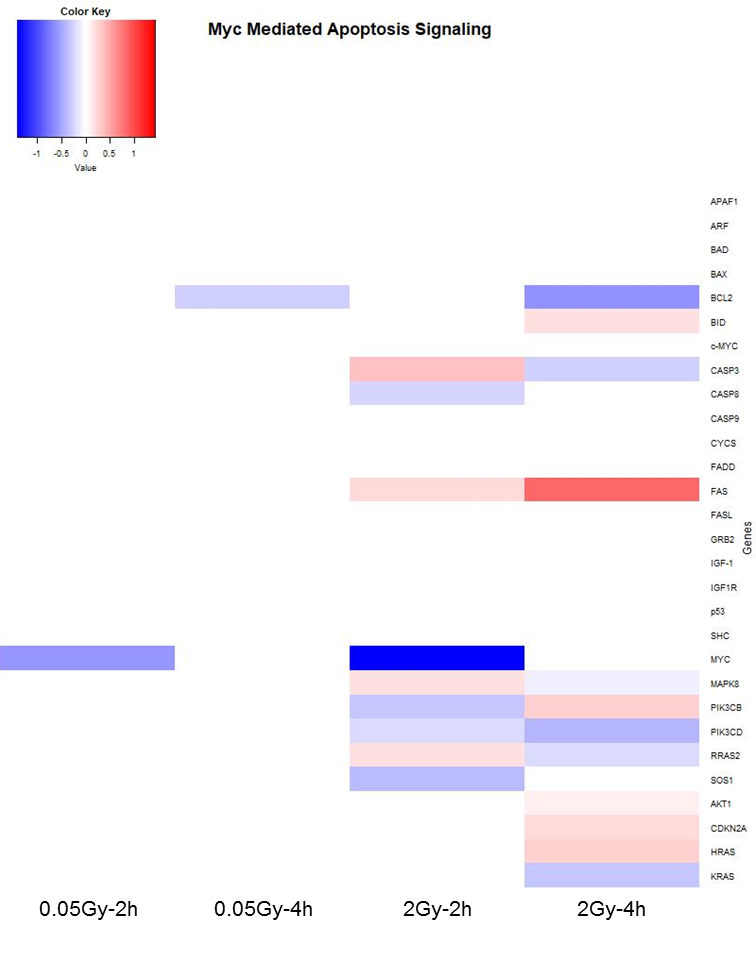


Web Figure 22: Gene expression in the “Not a Number” pathway *Myc mediated apoptosis signaling* (blue = downregulation, red: upregulation), Gy = Gray.


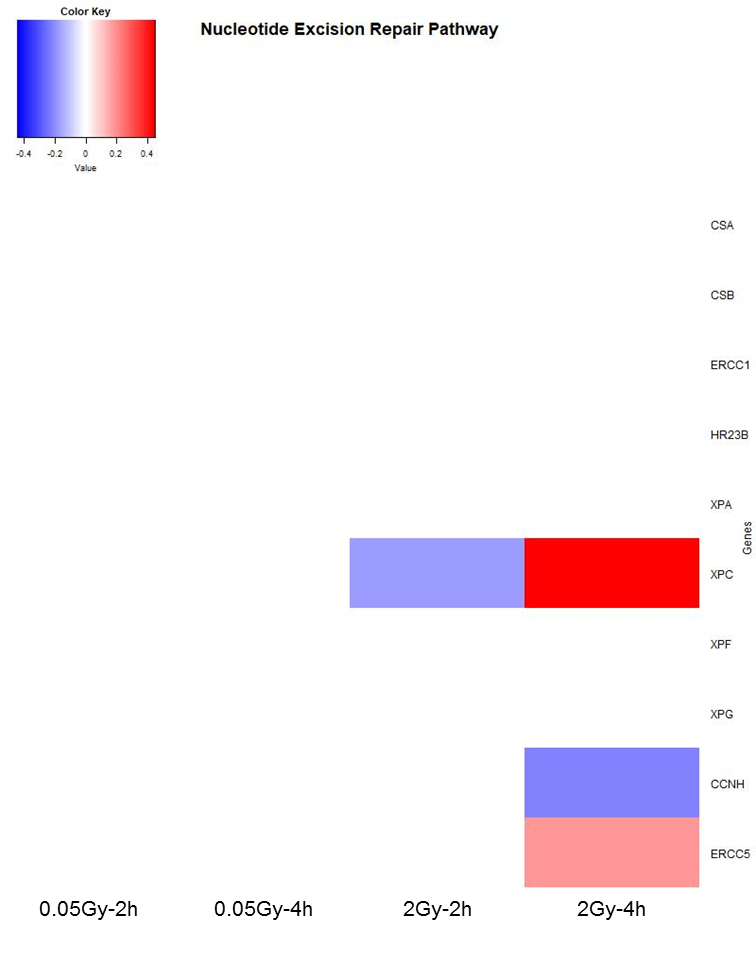


Web Figure 23: Gene expression in the “Not a Number” pathway *nucleotide excision repair* (blue = downregulation, red: upregulation), Gy = Gray.


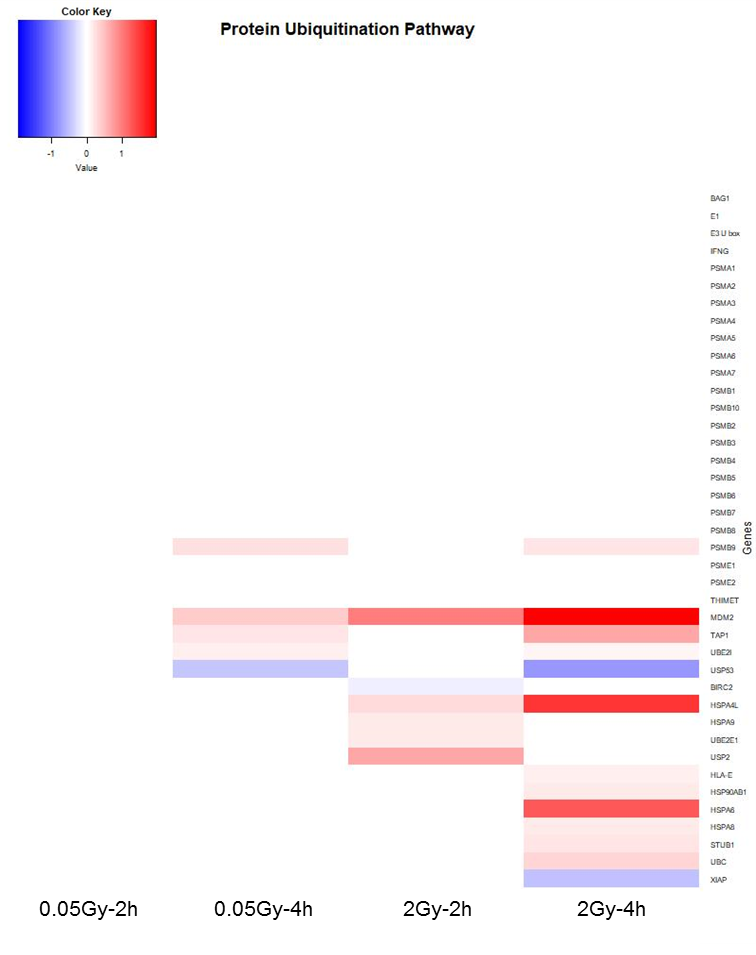


Web Figure 24: Gene expression in the “Not a Number” pathway *protein ubiquitination* (blue = downregulation, red: upregulation), Gy = Gray.


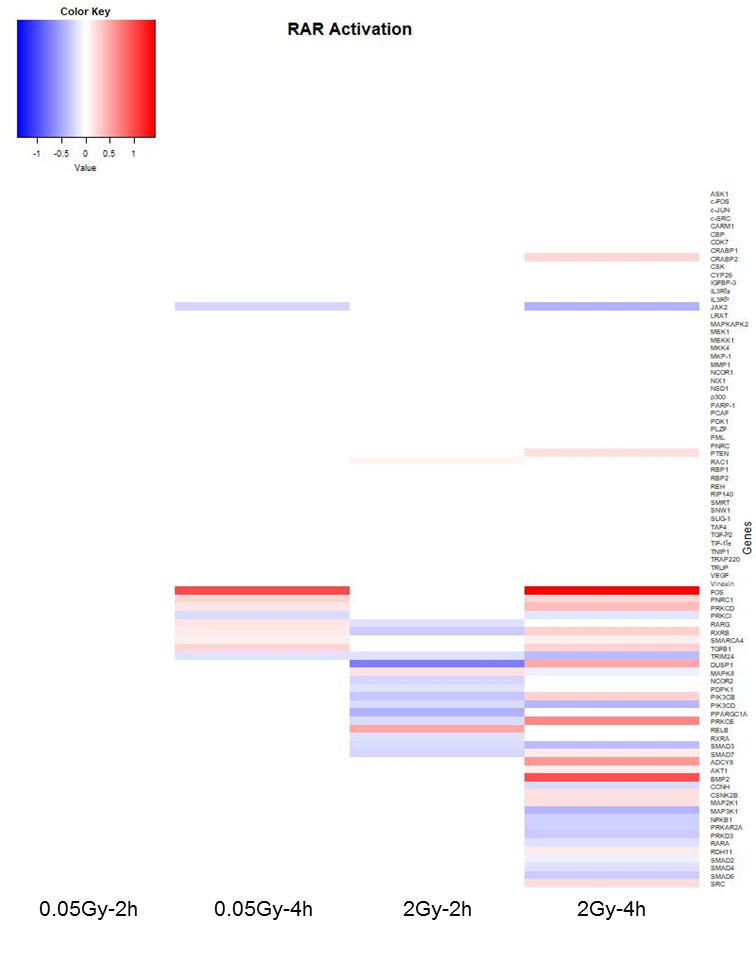


Web Figure 25: Gene expression in the “Not a Number” pathway *retinoic acid receptor (RAR) activation* (blue = downregulation, red: upregulation), Gy = Gray.


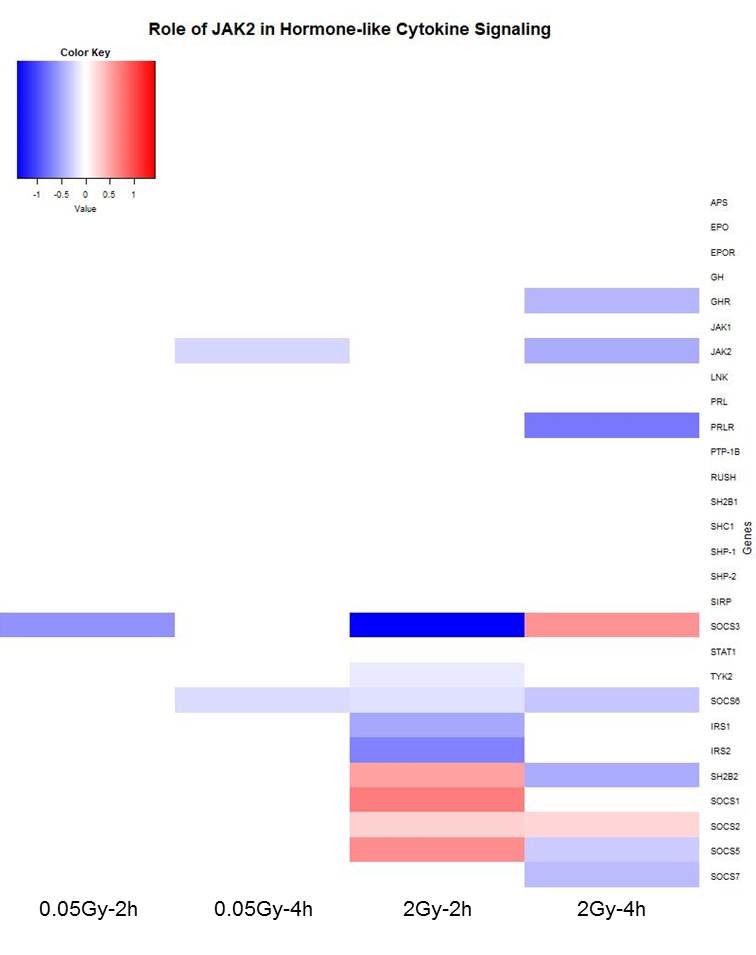


Web Figure 26: Gene expression in the “Not a Number” pathway *role of Janus kinase 2 (JAK2) in hormone-like cytokine signaling* (blue = downregulation, red: upregulation), Gy = Gray.


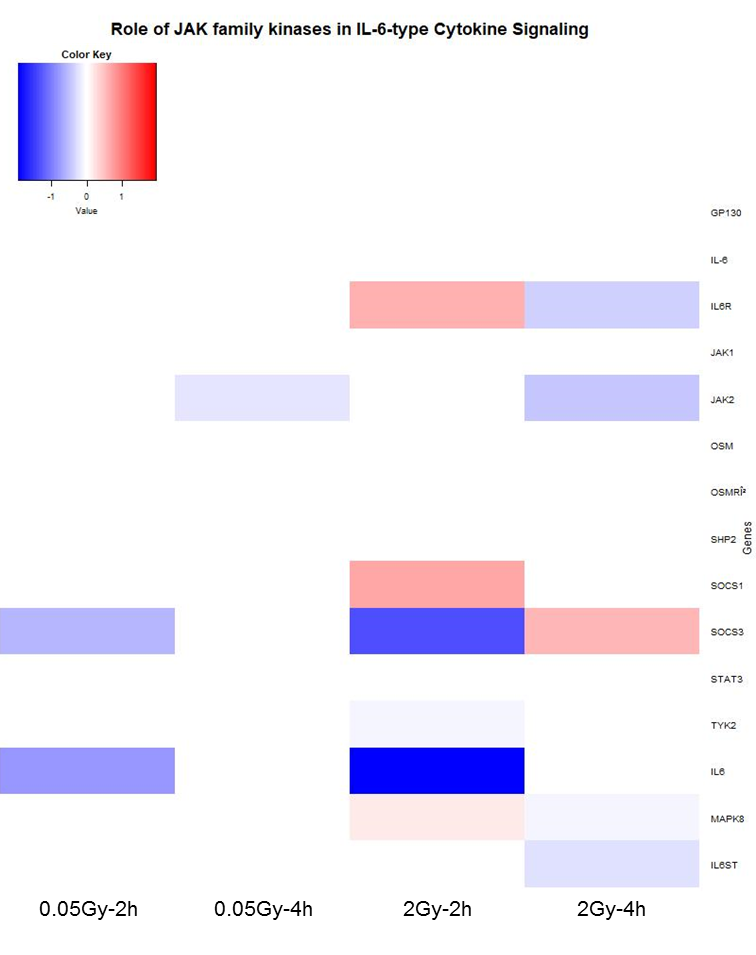


Web Figure 27: Gene expression in the “Not a Number” pathway *role of Janus kinase (JAK) family kinases in Interleukin 6 (IL-6) type cytokine signaling* (blue = downregulation, red: upregulation), Gy = Gray.


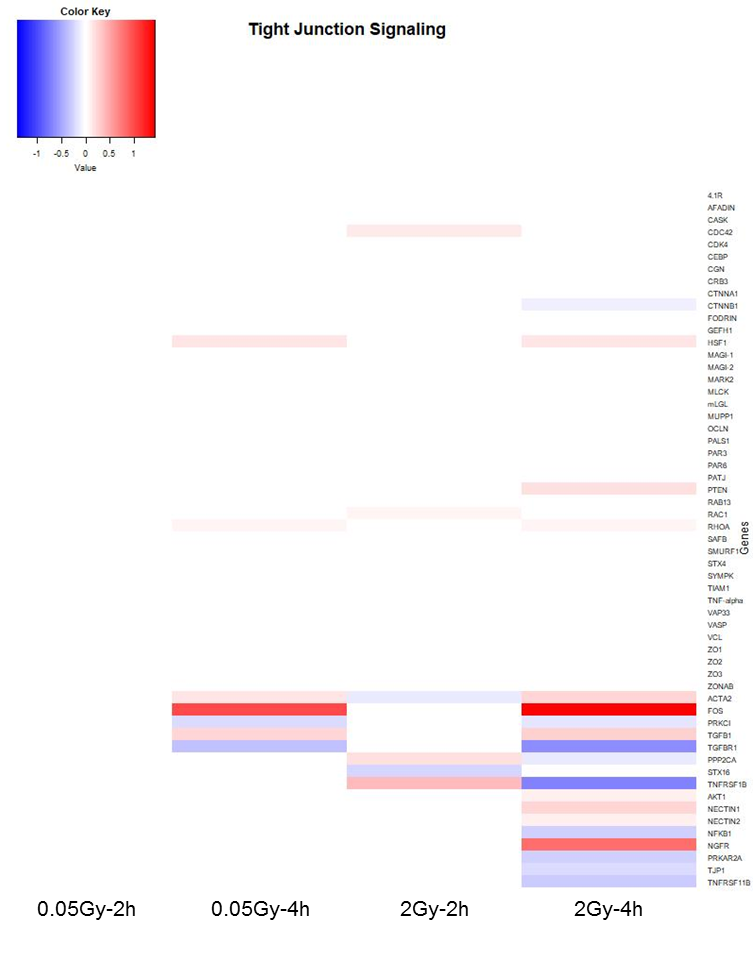


Web Figure 28: Gene expression in the “Not a Number” pathway *tight junction signaling* (blue = downregulation, red: upregulation), Gy = Gray
