# Supplementary material for: Comparison of time and dose dependent gene expression and affected pathways in primary human fibroblasts after exposure to ionizing radiation
Source: Mol Med. 2020 Sep 9;26:85. doi: 10.1186/s10020-020-00203-0 (PMC7488023; doi:10.1186/s10020-020-00203-0)
Supplement: Supplementary file 8 — Additional file 8: Web Table 1A. Differentially expressed genes 2 h after exposure to low dose ionizing radiation (0.05 Gray). [file 10020_2020_203_MOESM8_ESM.pdf]

Web Table 1A: Differentially expressed genes 2 hours after exposure to low dose ionizing radiation (0.05 Gray).

Gene	Log fold change	Average Expression	t	P-value	Adjusted P-value	B
PPP1R10	-0.507617105	5.684207763	-7.574473015	6.77E-11	1.02E-06	14.50914081
CITED2	-0.494064384	7.284362326	-5.838179731	1.19E-07	0.000299766	7.424618124
PLK3	0.422295983	4.367813593	5.866430393	1.06E-07	0.000299766	7.530413694
SLC25A25	-0.446987779	3.769731825	-5.881151425	9.99E-08	0.000299766	7.588570396
ZNF441	0.445566593	3.029300148	5.887386555	9.73E-08	0.000299766	7.425940119
ZNF792	0.714762149	0.668462933	5.908734083	8.90E-08	0.000299766	6.160192785
ZNF57	0.586465343	1.250619665	5.783416628	1.50E-07	0.000322456	6.612207354
SNAI2	0.409298509	6.800166099	5.721392236	1.94E-07	0.000364592	6.967229472
EGR2	-0.660292465	3.221774446	-5.54911146	3.93E-07	0.000578124	6.303394221
RNF214	-0.197523194	3.427979864	-5.573737924	3.55E-07	0.000578124	6.374277009
SIN3B	-0.192300572	5.12749706	-5.531300694	4.22E-07	0.000578124	6.234235742
BBC3	0.449804196	4.023738717	5.439860668	6.12E-07	0.000658571	5.865071961
PTGS2	-0.96888866	2.583827388	-5.454843676	5.76E-07	0.000658571	5.916743691
ZNF93	-0.485833678	1.484655632	-5.450647367	5.86E-07	0.000658571	5.697066696
HYLS1	0.344071757	2.854120555	5.375998189	7.92E-07	0.000745862	5.570969833
TMCC1	-0.172218576	4.187774405	-5.38214177	7.73E-07	0.000745862	5.66697741
ITPKC	0.301254668	4.639786348	5.338102815	9.23E-07	0.000817657	5.502336477
MAST3	-0.375744081	2.696501265	-5.185866831	1.70E-06	0.001403462	4.846735269
SPRY2	-0.453926634	4.381484668	-5.174993483	1.77E-06	0.001403462	4.883402608
HOXA9	0.446444943	2.911502299	5.138387954	2.05E-06	0.00154141	4.699477721
CSRNP1	-0.55130458	3.571844999	-5.086400257	2.51E-06	0.001802461	4.554302004
PYGO2	-0.173313805	4.882586005	-5.074348078	2.63E-06	0.001804143	4.5185434
TRIB1	-0.368931218	3.62736882	-5.045984801	2.95E-06	0.00192922	4.415025117
HILPDA	0.39007184	2.29563701	5.010800887	3.38E-06	0.002122228	4.15277638
BICRA	-0.287802786	3.558719552	-4.959436434	4.13E-06	0.002347401	4.106894219
CDKN1B	0.250169426	4.570327322	4.954799644	4.21E-06	0.002347401	4.088461923
CEP164	-0.199602282	4.464904601	-4.95783559	4.16E-06	0.002347401	4.094045672
SMYD4	-0.257243314	2.515640738	-4.93877756	4.48E-06	0.002409183	3.991364015
TSC22D2	-0.398083096	4.592108337	-4.922807713	4.76E-06	0.002475023	3.959157165
MYC	-0.590352181	4.969945963	-4.904206936	5.12E-06	0.002490574	3.878852704
PCF11	-0.323159636	4.194386172	-4.904002133	5.13E-06	0.002490574	3.905658167
MAU2	-0.186128246	4.89619244	-4.845420463	6.43E-06	0.002995408	3.67986403
ZNF404	-0.420122785	1.283114933	-4.837933927	6.62E-06	0.002995408	3.588704242
ZNF555	0.333746337	2.23977075	4.832312784	6.76E-06	0.002995408	3.545892035
EPC1	-0.249835117	4.143940781	-4.785221556	8.10E-06	0.003486784	3.477304867
IRF2BP2	0.203581207	6.155245929	4.730427028	9.99E-06	0.004179683	3.251646297
ATXN2L	-0.215050097	6.643587415	-4.701996357	1.11E-05	0.004412224	3.146921654
C19orf44	-0.316414284	1.993211342	-4.702807697	1.11E-05	0.004412224	3.112470271
KIF3A	-0.29064727	2.517266485	-4.666386097	1.27E-05	0.004907707	3.057970472
RNF19B	0.268085858	3.164707185	4.649141418	1.36E-05	0.004907707	2.997604603
USP27X	0.308967414	2.537054077	4.647507925	1.37E-05	0.004907707	2.986725518
ZNF230	0.420211903	1.889952638	4.655085189	1.33E-05	0.004907707	2.863913576
EMSY	-0.392017503	3.812486004	-4.637977585	1.42E-05	0.004947382	2.971515792
KLF6	-0.391505594	6.23785017	-4.627100197	1.48E-05	0.004947382	2.881104916
RSRC2	0.344768567	5.112437589	4.63054041	1.46E-05	0.004947382	2.921374232
LINC00921	-0.751309583	-1.34946933	-4.610114169	1.58E-05	0.005149751	1.459873656
REPIN1	-0.146741739	5.134372614	-4.604912889	1.61E-05	0.005149751	2.817810681
IL6	-0.798699595	1.982741516	-4.587983153	1.71E-05	0.005272998	2.80095433
MADD	-0.238136417	4.961093935	-4.587534299	1.72E-05	0.005272998	2.768273052
TXNDC9	0.167263939	4.550973891	4.576673118	1.79E-05	0.005382539	2.737309933
MTCL1	-0.245445568	6.909153688	-4.557474377	1.92E-05	0.005456622	2.635852723
ZNF281	-0.31538113	5.148538314	-4.557850802	1.92E-05	0.005456622	2.643888659
ZNF34	0.337420577	1.528761665	4.565357818	1.86E-05	0.005456622	2.518327964
NOCT	-0.521458746	1.541791622	-4.53811415	2.06E-05	0.00575765	2.607378471
ANAPC5	0.109296495	6.452380026	4.499848769	2.38E-05	0.006519567	2.434446437
BCL3	0.392794935	5.381712417	4.480175127	2.56E-05	0.006888703	2.378473963
DEDD2	0.300887568	4.046853599	4.456729902	2.79E-05	0.007382241	2.352133805
IL12A	-0.733063583	-1.299688675	-4.450844601	2.85E-05	0.007414678	1.319870178
GRK2	-0.130864734	5.242496109	-4.393254869	3.53E-05	0.009013066	2.083762572
CYTH1	-0.238865371	3.982642941	-4.357368727	4.03E-05	0.010109168	2.001111004
CDK7	0.222880236	4.160941339	4.328319601	4.48E-05	0.011056393	1.890570396
ZNF786	0.387760245	1.961840646	4.320122452	4.61E-05	0.011207906	1.823010318
NFRKB	-0.229122011	3.892256458	-4.307218376	4.83E-05	0.011560364	1.832921753
FST	0.231338805	6.619768423	4.271730452	5.50E-05	0.012943675	1.653048785
LRCH1	-0.216619478	4.130630219	-4.252058703	5.90E-05	0.013684198	1.644431017
RPRD2	-0.184809604	5.607265704	-4.235100088	6.28E-05	0.014327218	1.542285961
KANSL2	0.24809426	3.845354325	4.199550993	7.13E-05	0.016037984	1.485882749
BRSK1	-0.47156278	0.551035531	-4.194294519	7.27E-05	0.016102883	1.223025015
ZFPM2	-0.371156955	2.845053828	-4.17823708	7.70E-05	0.016808995	1.437982044

Gene	Log fold change	Average Expression	t	P-value	Adjusted P-value	B
RAB40C	-0.176581132	3.991638463	-4.167254248	8.01E-05	0.017232465	1.376182414
UHRF2	-0.305566958	4.163104614	-4.14981593	8.52E-05	0.018080736	1.317497618
ABL2	-0.432535935	6.051190918	-4.143570465	8.71E-05	0.018230866	1.228508303
COIL	0.176109094	3.932233374	4.126959459	9.24E-05	0.019075908	1.235523935
KIAA1614	-0.198581512	3.608454138	-4.113046863	9.71E-05	0.019771133	1.203746709
HPS4	-0.152562642	4.425754514	-4.094103636	0.000103866	0.020175154	1.099012672
KCNE4	-0.389313908	2.743789532	-4.10171893	0.000101103	0.020175154	1.189823712
LOC103344931	-0.321654308	1.718752912	-4.092477476	0.000104465	0.020175154	1.155609119
PNRC2	0.186125639	5.262119797	4.09332288	0.000104153	0.020175154	1.076254535
MRM3	0.203940262	2.800009944	4.088538486	0.000105931	0.020199238	1.152386557
BORCS6	0.304875397	2.190199816	4.083413379	0.000107867	0.020311401	1.113568555
RCOR3	-0.17486846	4.667935028	-4.076215822	0.000110644	0.020577138	1.043930012
PLEKHF1	0.326147189	5.026845982	4.066894948	0.000114343	0.021005702	1.002129094
EGR1	-1.00691369	6.810030351	-4.042674822	0.000124519	0.022337832	0.91440145
MARCH7	-0.204171067	5.848780365	-4.042579985	0.00012456	0.022337832	0.891730163
MAP3K10	-0.28972931	2.729229699	-4.030824638	0.000129809	0.023005248	0.967301401
CDAN1	-0.21330382	3.340838434	-4.021578433	0.000134086	0.023486938	0.930651232
ELL	0.222413642	4.299972796	4.011110174	0.000139092	0.024083725	0.824884916
ARL4D	0.287546564	3.596746301	4.000181216	0.00014451	0.024604115	0.85538145
DCAF5	-0.104378249	5.784441832	-3.998493034	0.000145364	0.024604115	0.753107972
EVC2	-0.166273378	4.110388829	-3.992477982	0.000148449	0.02484707	0.799460455
RNF216P1	0.17955027	3.292139692	3.983037436	0.000153418	0.025396531	0.80248366
CTC1	-0.199430693	2.69420865	-3.968387276	0.000161446	0.026150711	0.765927877
RUSC2	-0.190202148	5.867802485	-3.968915033	0.00016115	0.026150711	0.653235889
PHF13	0.40351287	5.251495747	3.954469146	0.000169444	0.026314553	0.625137043
RNF44	-0.192045622	3.467183352	-3.95916733	0.000166703	0.026314553	0.712913061
YTHDC1	-0.148101285	5.058275286	-3.955663592	0.000168743	0.026314553	0.63053985
ZNF766	0.190504908	3.520778069	3.956685225	0.000168146	0.026314553	0.719495047
HES1	0.407884074	4.343279933	3.951166838	0.000171397	0.026346229	0.625040309
BRPF1	-0.16709174	4.330299577	-3.947806575	0.000173407	0.026385849	0.630884592
RANBP10	-0.234257388	3.912218445	-3.937392049	0.000179779	0.027081875	0.618283738
DNTTIP2	0.150698288	6.084594931	3.932214806	0.000183029	0.027298557	0.535767691
AMOTL2	-0.726463878	6.437816275	-3.923530556	0.000188609	0.027854918	0.527777389
DENND2C	0.471567488	2.317854819	3.90751579	0.000199328	0.028223686	0.592473333
MARK2	-0.160702695	4.210483805	-3.905853019	0.000200474	0.028223686	0.515906796
POLR1C	0.323121971	3.869990066	3.91214177	0.000196173	0.028223686	0.558573773
SOCS3	-0.582021809	5.479201848	-3.906592856	0.000199963	0.028223686	0.455112574
SPRY1	-0.616988242	2.503585826	-3.909320388	0.000198091	0.028223686	0.587662752
RAB3A	0.463606154	1.450154373	3.89212802	0.000210175	0.028782544	0.476492309
ST3GAL6-AS1	1.000903977	-3.917254941	3.894617642	0.000208383	0.028782544	-1.061430237
ZKSCAN4	0.320710015	2.107599583	3.894571178	0.000208416	0.028782544	0.525876423
TMEM68	-0.224842509	2.976632077	-3.886890632	0.000213995	0.029041565	0.515870561
ERF	0.447729153	6.219476716	3.860782389	0.000234046	0.030742842	0.310897918
KATNAL1	-0.23011996	4.26854648	-3.859974937	0.000234694	0.030742842	0.359842249
KCNJ2	-0.502107407	2.402750033	-3.863802377	0.000231638	0.030742842	0.458878616
NFKBIZ	-0.774442759	1.118680004	-3.867040248	0.000229082	0.030742842	0.469801968
KLF9	0.248060748	4.966092688	3.85472465	0.000238949	0.03103038	0.313205938
ENO2	-0.22217787	4.872701056	-3.847871909	0.000244614	0.031249472	0.292664377
TIGD2	-0.242602439	2.481001479	-3.84766714	0.000244785	0.031249472	0.399412974
PPP1R15B	-0.254880895	4.861127733	-3.843799311	0.000248041	0.031399038	0.271877867
ORC2	-0.193962587	3.078329138	-3.835333529	0.000255312	0.031998131	0.34525682
PIM3	0.320194201	3.957166409	3.833377092	0.000257022	0.031998131	0.292899039
S100A2	0.467734428	0.100839542	3.830775092	0.000259312	0.032018633	0.157488877
ARID3A	0.277669375	3.536743634	3.826205729	0.000263381	0.032256664	0.323797626
TCF7L2	-0.242075014	5.198042049	-3.823769492	0.000265575	0.03226311	0.205488924
EZH1	-0.168482567	4.500825299	-3.812745032	0.000275725	0.032964513	0.211642141
OTUD1	-0.264869111	3.192914073	-3.812772568	0.0002757	0.032964513	0.263615922
SPTB	-1.228331294	-4.750620944	-3.808073708	0.000280137	0.032968653	-1.656782404
ZNF451	-0.214777013	4.417657652	-3.80880346	0.000279444	0.032968653	0.199249366
FBXO42	-0.165317959	4.369333738	-3.79629371	0.000291564	0.034047459	0.17039475
PIM2	-0.360736994	0.23449648	-3.79071948	0.000297125	0.034087669	0.033934631
TRIM68	-0.271196531	2.875231273	-3.792875124	0.000294963	0.034087669	0.240932905
WDCP	0.412509273	2.569546029	3.789161917	0.000298697	0.034087669	0.231921149
ZNF616	0.344351868	3.501483294	3.785531996	0.000302391	0.034249784	0.208332564
FAM214A	-0.2780167	3.816023579	-3.777522424	0.000310697	0.03482223	0.122634876
ZSCAN26	-0.200974569	3.204013778	-3.776219977	0.000312069	0.03482223	0.177203194
DCUN1D3	-0.259327405	4.725073927	-3.766229594	0.000322779	0.035270087	0.028827308
FAM161B	-0.180212779	2.68457292	-3.769951782	0.000318748	0.035270087	0.170142964
MOB4	0.174462347	4.220203624	3.765929709	0.000323106	0.035270087	0.073640415
CCDC120	-0.248090133	2.229933113	-3.757966646	0.000331902	0.035687636	0.143695638

Gene	Log fold change	Average Expression	t	P-value	Adjusted P-value	B
KCTD21	0.266213079	3.727474248	3.754474823	0.000335831	0.035687636	0.089588233
SYT3	-0.721774501	-1.899823605	-3.753965598	0.000336408	0.035687636	-0.624606317
ZNF649	0.252099538	2.65928391	3.758991773	0.000330757	0.035687636	0.141887119
ZNF829	-0.265182782	2.270884654	-3.750397103	0.000340475	0.035866547	0.120344393
DNAJC16	-0.251177834	3.686301898	-3.740326711	0.000352208	0.036141596	0.03088946
GOLGA6L17P	1.177266635	-4.628614519	3.745649048	0.00034596	0.036141596	-1.746277436
IER5L	0.327308585	5.5983292	3.734403507	0.000359288	0.036141596	-0.085210835
IL11	0.620527044	0.213437919	3.73391295	0.00035988	0.036141596	-0.044002186
INPP5E	-0.202108536	3.158091339	-3.737096093	0.000356053	0.036141596	0.052716686
KIAA0753	-0.238835918	3.141176367	-3.741935567	0.000350308	0.036141596	0.068116013
UQCRC2	0.096342052	6.751268197	3.736579687	0.000356671	0.036141596	-0.082852667
AHRR	-0.230966363	5.529719084	-3.726102967	0.000369438	0.036855764	-0.094667119
DISP1	-0.287560264	2.757101556	-3.715479659	0.000382828	0.037459348	0.003521334
E2F6	-0.191845051	2.67381161	-3.71696071	0.000380934	0.037459348	-0.012507497
TSC1	-0.192867906	5.003091299	-3.715385768	0.000382949	0.037459348	-0.104656588
AIMP1	0.163063442	5.445630436	3.7095727	0.000390472	0.037705616	-0.151022918
LARP7	0.135987801	4.916535587	3.710565336	0.000389178	0.037705616	-0.128110403
HPS6	0.206798619	4.726919039	3.702255481	0.000400143	0.037910416	-0.133466184
PHF21A	-0.192361159	5.12586995	-3.702418945	0.000399925	0.037910416	-0.164692621
ZFHX2	-0.476127493	0.566619232	-3.702333575	0.000400039	0.037910416	-0.16551128
FBRS	-0.117368279	5.970577001	-3.697731712	0.000406236	0.038247081	-0.200359837
ZNF248	-0.279494226	2.50186373	-3.683048164	0.000426625	0.039917297	-0.077418428
PISD	0.187369629	4.044412885	3.673763305	0.000440017	0.040916178	-0.18231351
CRYZL1	0.138455307	3.901238723	3.67181366	0.00044288	0.040929696	-0.191097684
N4BP2L1	-0.253859104	2.49685235	-3.664365829	0.000453978	0.041699582	-0.150865665
FAXDC2	-0.172124575	4.699607346	-3.652445515	0.000472294	0.042942598	-0.305649931
ZC3H4	-0.181122365	4.74804751	-3.651110598	0.000474389	0.042942598	-0.299168877
ZNF516	-0.223506946	3.906706921	-3.650047509	0.000476063	0.042942598	-0.263721019
IFFO2	0.308221353	3.495481602	3.640040745	0.0004921	0.043415139	-0.24944441
KLF7	-0.394290716	4.8151864	-3.639707236	0.000492643	0.043415139	-0.364048178
PRPF40B	-0.184311546	2.000630495	-3.637829792	0.000495712	0.043415139	-0.208800546
WHRN	-0.3216441	2.534640824	-3.638583231	0.000494478	0.043415139	-0.235750326
ZNF250	0.2288873	2.723961288	3.64303152	0.000487254	0.043415139	-0.19938351
ULK1	-0.167597347	5.493694662	-3.633396209	0.000503031	0.043801487	-0.389577966
DGKD	-0.265018939	2.708718347	-3.605110547	0.000552199	0.046201402	-0.320983464
FAM13B	-0.274013594	5.065322747	-3.611523845	0.000540666	0.046201402	-0.438701258
FAM212A	0.426894279	2.452264684	3.605650848	0.000551218	0.046201402	-0.299791461
MORC2	-0.1459457	4.966363712	-3.605867288	0.000550826	0.046201402	-0.445956782
RASD1	0.600067322	3.897393683	3.603501694	0.000555128	0.046201402	-0.335862022
SHROOM3	-0.317018208	4.119030279	-3.606590025	0.000549518	0.046201402	-0.43836946
TTC33	-0.287993286	2.747521737	-3.61542136	0.00053377	0.046201402	-0.27961544
ZNRD1ASP	-0.424633236	0.092261738	-3.603702076	0.000554763	0.046201402	-0.43933758
TMCC2	-0.338186618	1.883116238	-3.585876456	0.000588204	0.048685162	-0.354550503
ARID5B	-0.302289535	7.10762133	-3.579957111	0.000599725	0.048833846	-0.549959435
CRAMP1	-0.253283288	2.60678758	-3.581886852	0.000595946	0.048833846	-0.372153124
KIAA1107	-0.510682569	1.165641879	-3.581010065	0.00059766	0.048833846	-0.379261116
TOMM20	0.087778962	7.382595431	3.572935423	0.00061367	0.049700693	-0.576087711
