# Supplementary material for: Comparison of time and dose dependent gene expression and affected pathways in primary human fibroblasts after exposure to ionizing radiation
Source: Mol Med. 2020 Sep 9;26:85. doi: 10.1186/s10020-020-00203-0 (PMC7488023; doi:10.1186/s10020-020-00203-0)
Supplement: Supplementary file 9 — Additional file 9: Web Table 1B. Differentially expressed genes 4 h after exposure to low dose ionizing radiation (0.05 Gray). [file 10020_2020_203_MOESM9_ESM.pdf]

Web Table 1B: Differentially expressed genes 4 hours after exposure to low dose ionizing radiation (0.05 Gray).

Gene	Log fold change	Average Expression	t	P-value	Adjusted P-value	B
CDKN1A	0.432843772	9.578941307	8.592280753	7.46E-13	1.12E-08	18.45294046
SESN1	0.377724505	4.358072382	7.255830766	2.75E-10	2.07E-06	12.99102452
PPM1D	0.301682881	4.428273031	6.34317319	1.43E-08	7.16E-05	9.343067703
PIAS3	0.214282135	5.014677333	6.113953025	3.76E-08	0.000141784	8.480558511
PHLDB3	0.468393766	3.007928833	5.855704199	1.11E-07	0.000334452	7.203553078
MBNL2	-0.398759763	4.966848271	-5.782920262	1.50E-07	0.000376973	7.180888615
MTA3	0.152253748	4.715769842	5.683028064	2.27E-07	0.000488033	6.795100017
EHBP1	-0.233554344	6.601495233	-5.639609065	2.71E-07	0.000510451	6.647121528
APOL3	0.278570802	5.091969825	5.571441436	3.58E-07	0.000599841	6.379874611
NECAB3	0.329384083	3.046172099	5.525758002	4.32E-07	0.000650461	6.056547187
MDM2	0.396213602	6.788792003	5.45693695	5.71E-07	0.000658113	5.950355355
HR	0.367044286	3.229848901	5.478908066	5.22E-07	0.000658113	5.945279971
SAMD4A	-0.42097292	5.653654879	-5.449860986	5.88E-07	0.000658113	5.923138281
ARL15	-0.271105921	3.427625651	-5.440032531	6.12E-07	0.000658113	5.807819727
RANBP9	-0.199477375	6.11643876	-5.392186656	7.42E-07	0.000739991	5.705537442
PHLDA3	0.263949023	7.237706873	5.377958774	7.86E-07	0.000739991	5.651704401
USP21	0.277661312	3.607283331	5.295827279	1.09E-06	0.000968829	5.306681421
BBC3	0.409923678	4.023738717	5.244402218	1.34E-06	0.001123891	5.118207869
TANK	-0.193021374	4.913049683	-5.214115268	1.52E-06	0.00115607	5.037026412
TAB1	0.24947671	4.765249553	5.210880631	1.53E-06	0.00115607	5.024255899
ATF1	-0.173329521	4.947189102	-5.177949065	1.75E-06	0.001254991	4.904965038
ZNF219	0.223698433	6.050325957	5.147079354	1.98E-06	0.001351495	4.791397747
RBMS1	-0.225366254	6.997915219	-5.136296088	2.06E-06	0.001351495	4.751547288
GPI	0.180602348	7.898670936	5.101007711	2.37E-06	0.001430846	4.622568664
SLC39A7	0.123020517	7.498341312	5.092184745	2.46E-06	0.001430846	4.590235583
BLOC1S2	0.188341462	5.890622378	5.090794277	2.47E-06	0.001430846	4.584645319
PPP1R10	0.33782037	5.684207763	5.04417391	2.97E-06	0.001645005	4.413998405
RAP2C	-0.197236304	4.570085976	-5.036469747	3.06E-06	0.001645005	4.386402838
SPG20	-0.129773671	7.214586345	-5.021093377	3.25E-06	0.001678443	4.329647227
ZFPM1	0.413893723	2.984503149	5.013727717	3.34E-06	0.001678443	4.211386557
CDV3	-0.234343443	7.585817109	-5.004761754	3.46E-06	0.0016823	4.271134381
LAMTOR3	-0.151904665	5.231015097	-4.94282127	4.41E-06	0.002075142	4.047074648
PROKR1	-0.288643687	2.010559965	-4.92740422	4.68E-06	0.002136538	3.744415326
RPAP1	0.225225201	4.342937164	4.893858664	5.33E-06	0.002361833	3.872631957
POLRMT	0.21603156	4.041310195	4.880483161	5.61E-06	0.002416243	3.824603067
NAV3	-0.458940258	4.020636292	-4.86415663	5.98E-06	0.00250212	3.765309911
STAU2	-0.238971907	3.94009305	-4.852220988	6.26E-06	0.002513378	3.717908225
MVB12B	0.228056353	3.617885268	4.845135795	6.43E-06	0.002513378	3.684970227
FGD1	0.217240846	5.443223203	4.842243663	6.51E-06	0.002513378	3.684474194
SPRED2	-0.257744255	5.967506875	-4.834551282	6.70E-06	0.002524246	3.655898493
RHOBTB3	-0.250641713	6.8082914	-4.812147899	7.31E-06	0.00268432	3.575155861
KIAA1614	0.227096378	3.608454138	4.784309893	8.13E-06	0.002833638	3.477027994
SLC20A1	-0.594276109	7.015763557	-4.779630024	8.28E-06	0.002833638	3.460103963
PRIMPOL	-0.307748877	2.844398578	-4.785112874	8.10E-06	0.002833638	3.405528991
TGFB1	0.251149066	7.251656543	4.762665242	8.83E-06	0.002953226	3.400407545
GSE1	-0.240273799	5.83869265	-4.757207817	9.02E-06	0.002953226	3.379318774
GAS2L3	-0.482732583	3.350529159	-4.734137156	9.85E-06	0.003050554	3.30499913
SH2B3	-0.333073157	5.366836049	-4.73226628	9.92E-06	0.003050554	3.292957044
HSPA1L	0.510793415	1.072949412	4.732161259	9.92E-06	0.003050554	2.866127914
FBXO22	0.204152226	4.733788456	4.723438931	1.03E-05	0.003090577	3.269057059
SLC10A3	0.154741153	5.656145013	4.714502496	1.06E-05	0.003134839	3.229170925
TLCD1	0.26093056	2.17816021	4.694606101	1.14E-05	0.00331613	3.10009792
GTF2IRD1	0.190342221	4.135950413	4.688163238	1.17E-05	0.003334126	3.146966108
DCP1B	0.195069849	4.061718856	4.658954065	1.31E-05	0.003655409	3.045330726
KLHL42	-0.274081234	4.488815774	-4.653183994	1.34E-05	0.003666277	3.023769393
FERMT2	-0.297009111	7.023179374	-4.643868582	1.39E-05	0.003666277	2.980621042
WDR91	0.269425922	2.691396339	4.645119566	1.38E-05	0.003666277	2.954435079
VAV2	0.212410737	4.729795476	4.636235537	1.43E-05	0.003708436	2.962686081
SUOX	0.19823093	3.940767992	4.617207304	1.53E-05	0.003885726	2.899681102
TBL3	0.198013289	5.362563944	4.614859275	1.55E-05	0.003885726	2.881689228
SCAF1	0.18292039	6.637841298	4.569450978	1.84E-05	0.004533021	2.719083838
APEX2	0.187294199	4.288411392	4.563042731	1.88E-05	0.004544623	2.712701834
DUS2	0.227241061	2.788009318	4.555949904	1.93E-05	0.004544623	2.673191005
KY	0.625703535	0.221966586	4.556429267	1.93E-05	0.004544623	2.086469717
PLEKHA4	0.221880652	6.905160502	4.550653412	1.97E-05	0.004564263	2.655058609
PNRC1	0.237279033	6.351458449	4.544756387	2.01E-05	0.004595325	2.633089607
MIER1	-0.163246347	5.456200614	-4.505720552	2.33E-05	0.00523623	2.504054173
PRKD2	0.182248786	4.668302801	4.493129136	2.44E-05	0.005395704	2.466706715
PPME1	0.160522641	5.100861017	4.48974237	2.47E-05	0.005395704	2.451101111

Gene	Log fold change	Average Expression	t	P-value	Adjusted P-value	B
FLJ20021	0.415509322	1.914310567	4.46795276	2.68E-05	0.005766512	2.253405213
MOSPD2	-0.293415846	4.18114598	-4.460261701	2.76E-05	0.005849603	2.360822985
BNIP2	-0.217572632	5.977886095	-4.438856234	2.98E-05	0.006243563	2.26972567
USP11	0.171944755	6.414960399	4.429052446	3.09E-05	0.006303373	2.235072302
METAP1D	-0.247460614	2.506577814	-4.428851704	3.10E-05	0.006303373	2.209471564
APOL2	0.229024129	5.502636902	4.424234739	3.15E-05	0.006326148	2.220888576
ZBTB44	-0.159038893	5.6187454	-4.419621019	3.20E-05	0.006350001	2.206983306
C19orf68	0.290011466	3.024283823	4.414911471	3.26E-05	0.00637724	2.209072517
RIMKLB	-0.276387951	3.552132045	-4.404601631	3.39E-05	0.006482193	2.180512003
ZBTB45	0.18881402	4.443433278	4.40009095	3.44E-05	0.006482193	2.158011503
TNS2	0.212933447	7.209638618	4.402519332	3.41E-05	0.006482193	2.147678709
ING5	-0.251956584	2.91927999	-4.377633694	3.74E-05	0.006933881	2.083840546
UMAD1	-0.196433402	3.73117527	-4.375020211	3.77E-05	0.006933881	2.081821691
MED25	0.198099537	4.946566717	4.367101999	3.89E-05	0.00705203	2.039368243
NXPH4	0.323861596	2.88423744	4.359898955	3.99E-05	0.00707012	2.018192656
GPSM1	0.182599877	5.830682005	4.36154952	3.97E-05	0.00707012	2.007746778
ARHGAP18	-0.277617509	4.711536226	-4.352585753	4.10E-05	0.007175494	1.993127841
TAP1	0.189040143	5.266718745	4.346667758	4.19E-05	0.007175494	1.965100257
TAOK3	-0.179878274	5.237977076	-4.346368021	4.19E-05	0.007175494	1.96185892
SPRED1	-0.305921377	6.028243405	-4.337733088	4.33E-05	0.007322166	1.927144512
THEM6	0.227271919	3.370717777	4.321165819	4.60E-05	0.007691714	1.903006895
FGF5	-0.401790561	5.634833103	-4.31136771	4.76E-05	0.007766408	1.846045929
STAT5A	0.16190553	4.999452527	4.309494419	4.79E-05	0.007766408	1.842810965
HTR7P1	-0.243126947	2.700114598	-4.305919364	4.86E-05	0.007766408	1.829640317
OSBPL5	0.182099203	5.922964281	4.303651693	4.90E-05	0.007766408	1.812302154
HTR7	-1.161475245	-2.64235738	-4.306025605	4.86E-05	0.007766408	-0.517642063
DAB2	-0.224973146	9.017649532	-4.298920321	4.98E-05	0.007818791	1.822110857
ANKZF1	0.266377013	3.044247036	4.295934172	5.04E-05	0.007822572	1.820250796
SLC29A3	0.220067276	3.722881265	4.279187608	5.35E-05	0.008180528	1.765213532
PRELID3B	-0.16606138	5.026968209	-4.277973709	5.38E-05	0.008180528	1.737758052
MRPL2	0.240149281	4.176568333	4.270783206	5.52E-05	0.008224653	1.729513653
VIPAS39	0.235344668	4.662199395	4.263221957	5.67E-05	0.008224653	1.694617107
CARM1	0.123813747	6.667150074	4.265850294	5.62E-05	0.008224653	1.684462949
RAP2B	0.177746068	6.391043181	4.262877427	5.68E-05	0.008224653	1.67458384
TOX	-0.5339685	1.658053922	-4.265392956	5.63E-05	0.008224653	1.577679909
TCP1	0.125280262	7.349444151	4.255593523	5.83E-05	0.00836368	1.65588431
PUSL1	0.255157877	3.472066732	4.246444852	6.03E-05	0.008511107	1.658610832
SLC35A5	-0.185005419	5.087413857	-4.244394296	6.07E-05	0.008511107	1.624816289
PTPN12	-0.160259002	5.891333803	-4.242951717	6.10E-05	0.008511107	1.610958026
TRIQK	-0.187076743	4.673065596	-4.234910091	6.28E-05	0.008605279	1.605034483
UBL3	-0.162176774	6.968783265	-4.234811422	6.28E-05	0.008605279	1.583337897
VDR	-0.308811039	5.957651041	-4.227297077	6.46E-05	0.008761746	1.558921983
REEP4	0.255655772	3.368425212	4.217538006	6.69E-05	0.00891428	1.564458034
ETFBKMT	0.370359071	0.872196008	4.218140605	6.67E-05	0.00891428	1.298724462
LRP3	0.170675793	7.069257127	4.213577045	6.78E-05	0.008962817	1.512851173
RPS6KA4	0.157120338	6.213833542	4.201077537	7.09E-05	0.009292827	1.468693351
CGGBP1	-0.188226215	6.292001153	-4.188932796	7.41E-05	0.00960414	1.428687129
YIPF4	-0.152052798	5.556530055	-4.187078205	7.46E-05	0.00960414	1.428497428
DNM2	0.114014574	6.598203373	4.180873507	7.63E-05	0.009736688	1.401960707
ZNF367	-0.487469074	2.277310962	-4.177311982	7.72E-05	0.009778705	1.413898621
SRSF6	-0.212855494	6.611087388	-4.167739147	7.99E-05	0.01003487	1.3596519
SEMA6C	0.265983109	3.207102384	4.159632822	8.23E-05	0.010234908	1.378371565
TRAK1	-0.182069117	6.431975182	-4.157582674	8.29E-05	0.010234908	1.325057354
NAPG	-0.19648043	3.779086621	-4.149683206	8.53E-05	0.010274718	1.343137284
REST	-0.341895234	4.514536397	-4.153179107	8.42E-05	0.010274718	1.334677276
UBE2I	0.128183763	6.740166105	4.150543124	8.50E-05	0.010274718	1.302963933
MIR22HG	-0.309119854	4.512902585	-4.144448591	8.69E-05	0.010385102	1.31326148
CLDND1	-0.137961016	4.360996225	-4.137821784	8.89E-05	0.010543906	1.295129772
RFX1	0.21109009	3.721508768	4.135762891	8.96E-05	0.010543906	1.291643969
NBEA	-0.454445617	2.515692331	-4.131829365	9.09E-05	0.010600454	1.279720387
WBP4	-0.162557875	3.816927692	-4.126669945	9.25E-05	0.010600454	1.270728295
TCF25	0.138328863	6.966272345	4.128693893	9.19E-05	0.010600454	1.232776921
WWC2	-0.285799328	4.914778638	-4.12348098	9.36E-05	0.010600454	1.228221774
TMEM214	0.111644535	7.131425908	4.125496123	9.29E-05	0.010600454	1.224530917
KLHDC2	-0.121827167	4.782172718	-4.115327124	9.63E-05	0.010830405	1.211238802
IMPDH1	0.159916808	5.744370048	4.105763553	9.97E-05	0.011121045	1.158486279
ZBTB14	-0.164017534	3.540121875	-4.095756202	0.00010326	0.011437569	1.168656793
SS18	-0.196355017	5.341592048	-4.089872735	0.000105432	0.011575525	1.109334814
SLTM	-0.160849277	5.472935436	-4.088240434	0.000106042	0.011575525	1.105052726
LYRM7	-0.179270771	3.78862417	-4.085144634	0.000107209	0.011618711	1.137003265

Gene	Log fold change	Average Expression	t	P-value	Adjusted P-value	B
NFATC1	0.228176646	3.954438508	4.082764971	0.000108115	0.011633137	1.120940321
RTKN	0.214824664	3.428007467	4.078985766	0.000109568	0.011705862	1.114853377
PDSS2	-0.206998895	3.87787076	-4.073111545	0.000111863	0.011783985	1.096384407
ZNF213	0.205631996	4.585731165	4.074545992	0.000111299	0.011783985	1.076413174
SLC7A1	-0.373884947	6.361562501	-4.06473381	0.000115218	0.012053056	1.022945606
ZNF865	0.210190976	4.565038375	4.058882615	0.000117618	0.012219256	1.031739773
TRAF5	-0.259826247	3.504169606	-4.046737338	0.000122753	0.012665462	1.016719769
MBNL1	-0.230409628	7.043340342	-4.043486471	0.000124164	0.012723877	0.959023017
GMPPB	0.166883209	4.103838916	4.040878449	0.000125307	0.012754253	0.983495465
TTC14	-0.279044061	3.373045466	-4.027448608	0.000131355	0.012761172	0.96046304
HDAC9	-0.322034205	2.93685236	-4.026795217	0.000131657	0.012761172	0.958582092
NR4A3	-0.455045196	5.204685634	-4.032498395	0.000129049	0.012761172	0.948926862
SENP2	-0.170003705	4.945437393	-4.03200353	0.000129273	0.012761172	0.937723346
STX5	0.169502196	5.806787924	4.035747393	0.000127586	0.012761172	0.931464466
KRIT1	-0.198287361	4.207953914	-4.022766995	0.000133529	0.012761172	0.920395076
HK2	-0.330031327	5.150617754	-4.026290451	0.00013189	0.012761172	0.911110966
GFPT2	0.129589302	6.503074872	4.026159482	0.000131951	0.012761172	0.897220082
SLC30A9	-0.154620534	5.681041488	-4.02236389	0.000133718	0.012761172	0.889847976
ATP8B1	-0.41129837	5.899632055	-4.022089154	0.000133847	0.012761172	0.884263942
RPF2	-0.137718532	5.311659101	-4.017970509	0.000135792	0.012865201	0.879763735
VPS51	0.188324137	6.821742012	4.015990464	0.000136737	0.012873743	0.865551189
STYX	-0.220185171	4.33725848	-4.00900625	0.00014012	0.013082917	0.874335912
FBRSL1	0.169165634	4.821798694	4.007834197	0.000140695	0.013082917	0.860278927
SRM	0.215336055	7.019175171	4.001288099	0.000143952	0.013303616	0.820950049
DGKI	-0.387627493	4.003025498	-3.997979727	0.000145625	0.013333581	0.850920754
SPTLC3	-0.237344842	2.509336805	-3.99715261	0.000146046	0.013333581	0.849563242
RUSC1	0.167719697	5.049675164	3.989355876	0.000150075	0.013618845	0.796409898
DCAF16	-0.215882646	3.749407762	-3.98641814	0.00015162	0.013676699	0.815032394
PTPN13	-0.25838587	6.833740535	-3.980420622	0.000154823	0.01388246	0.752216564
AP4B1	0.223886478	2.725771101	3.974972651	0.000157788	0.013899525	0.799026434
SGSH	0.115083093	6.229337668	3.977535605	0.000156387	0.013899525	0.74129419
REEP3	-0.218678013	5.559262405	-3.974786691	0.000157891	0.013899525	0.73714401
ERBIN	-0.258495008	6.036175868	-3.972866263	0.000158949	0.013899525	0.728303584
PPP2R5D	0.156308925	5.904852937	3.971644174	0.000159627	0.013899525	0.726503316
PTPA	0.123525104	6.919767174	3.956631947	0.000168177	0.014559878	0.676756174
ZDHHC8	0.11974546	5.922642323	3.953479318	0.000170028	0.014635975	0.666772706
CACNB3	0.156539274	4.381478004	3.94848767	0.000172998	0.014807025	0.687678445
GABPA	-0.175638637	4.605709174	-3.943310267	0.000176131	0.01499002	0.659879617
GPATCH3	0.168232093	3.628358312	3.933460971	0.000182242	0.015314005	0.660479594
PELI3	0.190763069	4.250853172	3.931195023	0.000183676	0.015314005	0.631933139
MOB2	0.203835588	4.081970548	3.929086839	0.000185021	0.015314005	0.631925803
HM13	0.145536927	8.152803381	3.932307089	0.000182971	0.015314005	0.618916341
KLHL35	0.548466834	-0.067311872	3.929542402	0.000184729	0.015314005	0.122490329
NIPA1	-0.28386922	3.143569113	-3.920850781	0.000190363	0.015447405	0.62873857
NEDD4	-0.288380236	4.385058781	-3.920287117	0.000190734	0.015447405	0.597490486
BICD1	-0.277007238	4.067378472	-3.922543631	0.000189253	0.015447405	0.594730936
SMURF2	-0.302976939	5.204137854	-3.922678818	0.000189165	0.015447405	0.58595407
MTHFD2	-0.222728045	5.931491427	-3.918487723	0.000191923	0.015460576	0.559998528
KLF9	-0.256426023	4.966092688	-3.915473359	0.000193931	0.015539209	0.570029142
RPL28	0.294344137	9.710130806	3.913659851	0.000195148	0.015554034	0.588292397
RNFT1	-0.184845456	2.307757228	-3.908601431	0.000198583	0.015625147	0.585199443
SELENOO	0.232189261	3.877890603	3.908819307	0.000198434	0.015625147	0.573342587
MED15	0.123587887	6.511314176	3.907771508	0.000199152	0.015625147	0.519862497
CNOT10	0.132208954	4.394555074	3.905960636	0.000200399	0.015641526	0.553387038
NUDT18	0.24152704	3.070249745	3.903356877	0.000202206	0.015701154	0.578029559
VARS	0.126145109	6.49368658	3.90060234	0.000204133	0.015769562	0.496687253
TRPM7	-0.267775534	5.827134773	-3.898070659	0.000205921	0.015826476	0.490478713
PKNOX1	-0.170602743	4.683564458	-3.895028075	0.000208089	0.015831547	0.503415792
ALPK2	-0.334940289	5.400052428	-3.895674928	0.000207626	0.015831547	0.487761227
TMEM184C	-0.143397651	5.540114535	-3.89185277	0.000210374	0.01592502	0.47628073
LONRF1	-0.260976635	3.465584677	-3.88007619	0.000219063	0.016332871	0.496936615
ORC5	0.190404383	3.347553121	3.879128619	0.000219777	0.016332871	0.4932039
PINK1	0.151467544	4.875322563	3.880618045	0.000218656	0.016332871	0.45503961
C17orf97	0.3738605	1.192324686	3.878702442	0.000220099	0.016332871	0.421535665
ANKRD13B	0.224564476	4.322104598	3.867850592	0.000228447	0.016605867	0.436333939
OPLAH	0.16964467	4.170025649	3.865376217	0.000230392	0.016605867	0.432001207
BAG6	0.108733604	7.873821161	3.871493182	0.000225612	0.016605867	0.421789484
BRF1	0.172694249	4.718201846	3.865930587	0.000229955	0.016605867	0.41652925
FPGS	0.15431901	5.455366822	3.868309444	0.000228088	0.016605867	0.403146842
PTPN23	0.135505879	6.982300781	3.867721654	0.000228548	0.016605867	0.396848777

Gene	Log fold change	Average Expression	t	P-value	Adjusted P-value	B
SGPP1	-0.173112232	4.699774945	-3.862048369	0.000233033	0.016637041	0.3996477
ALG3	0.180441177	5.236578846	3.862353943	0.00023279	0.016637041	0.388056567
LPIN2	-0.203210443	5.651252628	-3.858662369	0.000235751	0.016751641	0.370045217
PHAX	-0.177201412	5.877160807	-3.848535999	0.000244059	0.017260586	0.335888726
GBP1	-0.360077801	4.986768263	-3.842498734	0.000249145	0.017475471	0.340429929
EIF2B4	0.194737843	4.922494569	3.842178323	0.000249418	0.017475471	0.333667763
TRIM68	0.237511119	2.875231273	3.837594411	0.000253351	0.017668867	0.374222879
C3orf58	-0.226126635	4.484756733	-3.834478937	0.000256058	0.017775358	0.333878996
ZNF674	-0.363803389	0.878530875	-3.82789256	0.000261872	0.018095574	0.174718644
CCDC28B	0.317524916	2.225801655	3.817000787	0.000271764	0.018638763	0.306360439
KITLG	-0.336091569	6.688324879	-3.81652187	0.000272207	0.018638763	0.233713322
MLLT3	0.296576131	3.68347239	3.809776866	0.000278521	0.018762629	0.282652257
AP2A1	0.118487986	7.937477936	3.809273002	0.000278998	0.018762629	0.229246451
STIP1	0.142974737	7.143542889	3.812398117	0.000276051	0.018762629	0.226163986
MAP2K3	-0.302587667	6.536569544	-3.809973075	0.000278335	0.018762629	0.21337423
ISYNA1	0.24101549	3.69154865	3.804815366	0.000283254	0.018809987	0.264113309
RASA2	-0.366329278	3.925605774	-3.806717183	0.000281431	0.018809987	0.257415059
USP12	-0.227991172	4.182484426	-3.801221319	0.000286731	0.018809987	0.232129186
SUDS3	-0.14693021	4.456171382	-3.801289205	0.000286665	0.018809987	0.223400887
ARHGDIA	0.135712239	8.147761136	3.800429151	0.000287503	0.018809987	0.207906705
PAK4	0.186139355	4.955729258	3.799348832	0.000288559	0.018809987	0.201014089
PLCD1	0.183421317	5.186049927	3.798255862	0.00028963	0.018809987	0.192088238
MBOAT7	0.135450094	6.725798185	3.798193378	0.000289692	0.018809987	0.177063583
ZNF385A	0.307827138	5.010030805	3.791762534	0.000296077	0.019142073	0.178501805
CCDC102A	0.254471063	3.690003772	3.787054609	0.000300836	0.019284252	0.210243584
C1orf94	1.217045694	-4.325427959	3.787558798	0.000300323	0.019284252	-1.800731774
UBTF	0.10945776	7.103248139	3.782403924	0.00030561	0.019507217	0.133452056
AP1AR	-0.202039562	3.820026589	-3.778524749	0.000309646	0.019681479	0.175902746
MARCKS	-0.112648561	8.796832226	-3.769232419	0.000319524	0.020055435	0.125672025
ARHGAP22	0.256238643	5.005111097	3.770129615	0.000318557	0.020055435	0.115322873
MAGI2-AS3	-0.13035725	5.659663206	-3.769948151	0.000318752	0.020055435	0.093512125
UCK1	0.158979958	4.799372464	3.762421114	0.000326954	0.020352215	0.09382649
EGR1	0.902168994	6.810030351	3.763047756	0.000326264	0.020352215	0.071922969
FIZ1	0.180274275	3.938184887	3.761110295	0.000328403	0.020358272	0.122627294
GPKOW	0.198978766	4.351074194	3.755496367	0.000334677	0.020662193	0.088710992
PRKCI	-0.213188489	4.603567934	-3.752951564	0.000337559	0.020670667	0.066945909
RALGPS2	-0.285576451	5.476268076	-3.753811397	0.000336582	0.020670667	0.045973636
ADO	-0.123933648	5.077587352	-3.749107353	0.000341957	0.020771108	0.042947764
RYK	-0.099468182	6.29346394	-3.749681251	0.000341297	0.020771108	0.026579681
VEZT	-0.14524929	5.377023165	-3.742675629	0.000349437	0.021055693	0.017143004
EHMT2	0.177692375	5.895375297	3.743814323	0.000348102	0.021055693	0.011161201
RARG	0.142326293	7.413476339	3.739996763	0.000352599	0.021161549	0.009177484
OSGEPL1	0.255111912	1.747334524	3.738355433	0.000354549	0.021194173	0.040667057
OCEL1	0.234706983	3.555348854	3.733463404	0.000360424	0.02125079	0.055234776
LMNA	0.142748103	10.63727751	3.731711927	0.00036255	0.02125079	0.049133473
USP53	-0.436086626	5.689863853	-3.733732233	0.000360099	0.02125079	-0.011316766
MSL1	-0.169213001	6.004124613	-3.732487051	0.000361608	0.02125079	-0.026083632
SART1	0.211672395	6.448224005	3.732252668	0.000361892	0.02125079	-0.027251969
USP20	0.143332488	4.269455515	3.72604247	0.000369513	0.021574999	-0.000879371
A4GALT	0.178144371	6.119492771	3.72386619	0.00037222	0.021649125	-0.051902981
SIX5	0.234204481	3.348596153	3.710391082	0.000389405	0.02171094	-0.01092018
CPSF4	0.23074923	4.182432959	3.720340778	0.000376645	0.02171094	-0.012519347
HIP1R	0.211216815	3.429358216	3.709748381	0.000390243	0.02171094	-0.013637801
CRY1	-0.21265703	3.768772653	-3.715976348	0.000382192	0.02171094	-0.023715041
NEK1	-0.295590716	4.777719625	-3.716172887	0.000381941	0.02171094	-0.048432051
FAM102B	-0.274801359	4.725748207	-3.711718986	0.000387678	0.02171094	-0.057969848
DNAJB2	0.141736003	5.873914115	3.720538861	0.000376395	0.02171094	-0.059744082
PPP1R37	0.183530205	5.806505945	3.714357868	0.000384269	0.02171094	-0.078040139
WIPI2	0.106414109	6.344099156	3.714516827	0.000384065	0.02171094	-0.081508706
ZMAT2	0.10440008	6.306969379	3.710912484	0.000388726	0.02171094	-0.092388759
RHOG	0.159031368	6.275164448	3.710648438	0.00038907	0.02171094	-0.093115527
PBRM1	-0.273835781	5.943743675	-3.709491987	0.000390578	0.02171094	-0.09415671
ZFHX2	0.398011475	0.566619232	3.706883015	0.000394001	0.021820704	-0.114979159
FAM83G	-0.297959306	3.792874725	-3.704510847	0.000397138	0.021913887	-0.03107779
CUL5	-0.269918689	4.752082652	-3.702429123	0.000399911	0.021923371	-0.092253273
PPP1R9B	0.116661686	7.069330625	3.70219735	0.000400221	0.021923371	-0.112423216
GJC1	-0.460911341	4.233130727	-3.698360038	0.000405384	0.022045872	-0.087204848
APBB1	0.124813337	5.818094618	3.698432911	0.000405285	0.022045872	-0.125919812
PAPOLA	-0.158778695	7.031438212	-3.695219244	0.000409657	0.022198123	-0.135929407
KHSRP	0.096673605	7.868583538	3.691103974	0.000415321	0.022424375	-0.132114798

Gene	Log fold change	Average Expression	t	P-value	Adjusted P-value	B
USP5	0.098145782	6.585597176	3.688724846	0.00041863	0.022522281	-0.159935938
MACF1	-0.335919852	7.50681922	-3.687610843	0.000420187	0.022525638	-0.151305028
SLC22A15	-0.326142794	2.009207023	-3.678382664	0.000433305	0.022609663	-0.096436723
ZSCAN12	-0.380943633	1.517281409	-3.678066169	0.000433762	0.022609663	-0.111479218
MOB1B	-0.232478825	4.458923374	-3.682180474	0.00042786	0.022609663	-0.143407842
VGLL3	-0.279089949	8.705675528	-3.680938959	0.000429633	0.022609663	-0.149981915
UCK2	-0.241743311	5.05915692	-3.682264057	0.000427741	0.022609663	-0.164954528
SLC38A1	-0.320906135	6.776361395	-3.684141655	0.000425074	0.022609663	-0.171855425
PRKACB	-0.136423382	5.353874169	-3.680621767	0.000430087	0.022609663	-0.173126363
MEN1	0.198145848	5.350119782	3.678433975	0.000433231	0.022609663	-0.17704145
ARMC7	0.167744945	2.825082243	3.67551028	0.000437467	0.02261507	-0.110909969
PHLPP2	-0.335706291	3.846106915	-3.674891038	0.00043837	0.02261507	-0.137803858
DDX54	0.114384662	5.817737169	3.676004877	0.000436748	0.02261507	-0.196574323
TTLL7	-0.392554793	1.52854077	-3.673742677	0.000440048	0.022624149	-0.146170729
UBXN2A	-0.134145208	4.673106186	-3.670153688	0.000445331	0.022817904	-0.184021461
TCHP	0.177719536	3.852572961	3.66892584	0.000447152	0.022833559	-0.152249565
SGTB	-0.184889491	4.091010876	-3.665822208	0.000451788	0.022847333	-0.178119546
SERPINB8	-0.228329516	4.746140507	-3.667342771	0.000449511	0.022847333	-0.202295166
INF2	0.145965869	7.286861847	3.665699393	0.000451972	0.022847333	-0.222760471
PSMB10	0.247528107	3.224473978	3.661128641	0.000458885	0.023119187	-0.151671742
CDC42BPA	-0.235534248	6.238744506	-3.659937614	0.000460702	0.023133396	-0.246543775
ARHGAP39	0.199822645	2.85354167	3.648513086	0.000478489	0.023459227	-0.190809149
C1D	-0.204443655	2.341325739	-3.640632348	0.000491138	0.023459227	-0.197515254
SAC3D1	0.242420152	3.502055546	3.647434069	0.000480203	0.023459227	-0.201817335
LIMD2	0.227641432	2.924597232	3.64336106	0.000486723	0.023459227	-0.203274219
KLHL2	-0.205856608	3.258042699	-3.641876107	0.000489121	0.023459227	-0.214538654
HDAC11	0.173731681	3.684941353	3.641092302	0.000490391	0.023459227	-0.227612869
TRIM28	0.155808776	8.119257323	3.650719195	0.000475005	0.023459227	-0.250434174
LRFN3	0.145962	5.117812102	3.651796171	0.000473312	0.023459227	-0.254503699
NFKB2	0.185498701	5.123431713	3.648710359	0.000478177	0.023459227	-0.262233223
ASMTL	0.167828571	5.051194808	3.647599915	0.000479939	0.023459227	-0.263912689
TELO2	0.190660396	4.576867282	3.640035783	0.000492108	0.023459227	-0.268137803
MARK4	0.156952449	5.94190108	3.65172228	0.000473428	0.023459227	-0.269252487
IDH3G	0.225734068	5.597020857	3.645972884	0.000482532	0.023459227	-0.281223028
EPS15L1	0.133894082	5.364930811	3.643335834	0.000486763	0.023459227	-0.284521216
MYBBP1A	0.1179643	6.157068329	3.644347474	0.000485136	0.023459227	-0.294642343
ZCCHC14	-0.216749117	6.731432286	-3.64015321	0.000491917	0.023459227	-0.302554941
ZFPL1	0.201580967	2.518879796	3.635752843	0.000499128	0.023710938	-0.211422386
RBM15	-0.249246447	2.428941939	-3.633770309	0.000502409	0.023710938	-0.219657628
COQ7	-0.14763268	3.161389543	-3.633101643	0.000503521	0.023710938	-0.231566431
MAK16	-0.223146029	3.875494573	-3.632059076	0.000505258	0.023710938	-0.265546963
ATIC	0.125269348	6.460109309	3.632917403	0.000503827	0.023710938	-0.329012366
SH2D4A	-0.209109594	5.219020967	-3.630282408	0.000508232	0.023776439	-0.317954949
KIAA1109	-0.369556492	5.430957883	-3.628860904	0.000510624	0.023814365	-0.329797916
RHBDD2	0.164021213	5.932854676	3.626671788	0.000514328	0.023913073	-0.345535505
FTH1	0.266283376	9.6883212	3.621798643	0.000522665	0.024209826	-0.299673337
RADIL	0.389638124	1.702855776	3.62026192	0.000525321	0.024209826	-0.322466034
TMEM102	0.432072874	-0.124321669	3.620139951	0.000525532	0.024209826	-0.478648763
SERINC3	-0.150912189	7.352570452	-3.619100706	0.000527336	0.024218861	-0.360038522
C16orf52	-0.23992122	3.663838903	-3.616789236	0.000531369	0.02425619	-0.286060041
WASL	-0.196368407	6.017048397	-3.617245642	0.00053057	0.02425619	-0.374702175
RNASEK	0.374919892	1.024010551	3.615245677	0.000534079	0.024306224	-0.354880356
LRRK2	-0.310745774	3.697537029	-3.612157289	0.00053954	0.024480793	-0.30091279
CSNK1G2	0.134863866	6.492699089	3.610871899	0.000541828	0.024510799	-0.395213934
SORBS3	0.245946889	7.625547937	3.608445261	0.000546173	0.024633398	-0.390668861
RNF138	-0.181723251	3.544573481	-3.60702385	0.000548734	0.024675011	-0.319967275
ZFC3H1	-0.310650834	4.053920773	-3.598265969	0.000564765	0.025208995	-0.405516356
COG1	0.110367612	4.727977434	3.597800284	0.000565629	0.025208995	-0.40641611
SLC25A1	0.206323858	6.55098851	3.598966431	0.000563466	0.025208995	-0.430719647
RNF24	-0.206868197	6.04313865	-3.59633981	0.000568349	0.025255505	-0.438244392
MAGED1	0.100771135	8.072603753	3.593863169	0.000572991	0.025274657	-0.418373383
SOCS6	-0.205043775	4.845977168	-3.592419205	0.000575713	0.025274657	-0.423428251
SASH1	-0.228145504	7.215031413	-3.593364717	0.000573929	0.025274657	-0.441093502
LNPK	-0.168307841	5.462098598	-3.591773174	0.000576935	0.025274657	-0.443044546
CTDSP1	0.150676876	7.342935895	3.591649376	0.00057717	0.025274657	-0.444498715
ZSWIM6	-0.323526265	3.109707887	-3.585517607	0.000588896	0.025713422	-0.359794748
TBC1D31	0.256681317	1.815630738	3.581518419	0.000596666	0.025754073	-0.375271498
EIF4H	0.078836754	7.236233068	3.582356742	0.000595029	0.025754073	-0.471564359
ST6GALNAC4	0.174736462	5.547283024	3.581767369	0.000596179	0.025754073	-0.474316764
PITPNB	-0.161019638	5.925321791	-3.582852271	0.000594064	0.025754073	-0.477178924

Gene	Log fold change	Average Expression	t	P-value	Adjusted P-value	B
LARP4B	-0.203814444	5.082449967	-3.577730427	0.000604115	0.02600109	-0.479157611
CBL	-0.315692694	4.166663103	-3.576101888	0.000607344	0.026028479	-0.460715091
KIAA1211	-0.47613036	0.379276119	-3.575668307	0.000608207	0.026028479	-0.595818961
NUP85	0.148923504	4.06753551	3.571994141	0.000615563	0.026264147	-0.45171713
PNPLA6	0.127434695	5.93706266	3.571181886	0.0006172	0.026264147	-0.510096098
TMUB1	0.231119748	4.547735852	3.568335719	0.000622971	0.026350122	-0.477623723
MALT1	-0.274973234	4.654921077	-3.567910895	0.000623837	0.026350122	-0.481023278
PURB	-0.234240457	5.964151163	-3.567601435	0.000624469	0.026350122	-0.520261414
ZNF480	0.182634987	3.823526492	3.564351061	0.000631137	0.026409567	-0.462597575
BTG2	0.42417629	4.197394101	3.565840755	0.000628072	0.026409567	-0.4633905
CCDC85B	0.328753394	6.94206842	3.565089152	0.000629617	0.026409567	-0.530997883
CLMP	-0.135232007	8.371915616	-3.561234141	0.000637595	0.026421389	-0.507420613
U2SURP	-0.16049339	5.642251186	-3.56316621	0.000633584	0.026421389	-0.528875977
TSPAN17	0.114074691	5.426908594	3.560935988	0.000638216	0.026421389	-0.533118767
FUT8	-0.142941264	6.102206878	-3.560830697	0.000638435	0.026421389	-0.54224543
INPP4A	-0.196042935	4.234555589	-3.559107885	0.000642036	0.026497609	-0.490601274
IL4I1	0.296562349	1.592665219	3.557193484	0.00064606	0.026590824	-0.454572252
FRMD5	-0.45275182	1.257888259	-3.553322432	0.000654269	0.026671466	-0.46846697
CREBZF	-0.305970055	3.287571038	-3.553870321	0.000653101	0.026671466	-0.481177127
SPCS2	0.146830683	3.832361087	3.554229958	0.000652336	0.026671466	-0.493832479
FAM91A1	-0.197860212	4.691385197	-3.551279186	0.000658642	0.026671466	-0.542723386
SLC6A8	0.13053586	5.39886838	3.552801111	0.000655382	0.026671466	-0.557868305
RNF224	1.147663631	-4.229898317	3.551397955	0.000658387	0.026671466	-2.176596781
ADAL	-0.16939053	2.854314826	-3.548717841	0.000664163	0.026822932	-0.46354569
MON1A	0.197101777	3.87618841	3.546315669	0.000669381	0.026880902	-0.518710801
FSCN1	0.181976038	8.71078901	3.547188751	0.00066748	0.026880902	-0.538041388
TWF2	0.168757238	5.613795479	3.545595873	0.000670952	0.026880902	-0.5811157
IFT88	-0.183222925	3.299475648	-3.544081638	0.000674268	0.026942113	-0.494693185
STRADA	0.284696383	0.898819051	3.542174735	0.000678467	0.026966813	-0.5517944
RABEP1	-0.138000425	6.593265251	-3.542254319	0.000678291	0.026966813	-0.598295151
ZBED6	-0.681782407	1.422917967	-3.54046501	0.000682252	0.027039357	-0.490912014
C19orf48	0.139948196	4.472322093	3.538925923	0.000685677	0.027039357	-0.566629463
MED16	0.144877961	6.164177486	3.539196749	0.000685073	0.027039357	-0.606957967
ATAD3A	0.186557812	4.426195674	3.537976303	0.000687798	0.027052187	-0.57225297
HINT3	-0.174692536	4.153468796	-3.536083903	0.000692043	0.027133291	-0.554315656
TTLL12	0.1246159	5.556258207	3.535453917	0.000693462	0.027133291	-0.613120533
PNMAL2	0.208000009	2.994612164	3.529368267	0.000707311	0.027320352	-0.534224037
ZNF710-AS1	0.380525253	0.877530417	3.531715778	0.000701938	0.027320352	-0.596195238
PTBP1	0.098021714	7.729849333	3.531700099	0.000701974	0.027320352	-0.608244421
CCAR1	-0.152498229	5.45224619	-3.530066408	0.000705709	0.027320352	-0.625185836
FHOD1	0.226606546	5.55138205	3.529491761	0.000707028	0.027320352	-0.634414149
SLC25A11	0.172139851	5.103066906	3.52758354	0.000711422	0.027368709	-0.617692482
MID1IP1	-0.141867374	5.248961084	-3.527248285	0.000712197	0.027368709	-0.626792485
LPGAT1	-0.230043455	5.554739039	-3.524091716	0.000719531	0.027580177	-0.646137456
WDR11	-0.164209476	5.157580324	-3.52295449	0.00072219	0.027611864	-0.638642061
THOP1	0.148060902	4.967169799	3.519753778	0.000729726	0.027829342	-0.640956095
PLCL1	-0.308359992	2.611429262	-3.513825721	0.00074388	0.028155283	-0.555931076
FLRT2	-0.250107595	6.931790448	-3.515075981	0.000740873	0.028155283	-0.675818827
CRAT	0.119831681	6.594063384	3.514038826	0.000743366	0.028155283	-0.682433569
ALDOA	0.196304651	10.01998868	3.511697199	0.000749025	0.028278971	-0.611680484
DNAAF2	-0.15770924	3.127355714	-3.508777056	0.000756138	0.028362901	-0.60373466
KCTD9	-0.149699329	4.508153883	-3.508467433	0.000756896	0.028362901	-0.661172313
TMEM250	0.095628768	6.368877724	3.508579036	0.000756623	0.028362901	-0.698730173
STX3	-0.216742811	3.958574505	-3.506054557	0.000762828	0.028375166	-0.635803851
TRIM24	-0.191887432	4.159617874	-3.506035679	0.000762875	0.028375166	-0.657738818
REXO4	0.121326194	4.968767575	3.507438112	0.000759421	0.028375166	-0.6798107
CCDC112	-0.238761775	2.439224781	-3.502097227	0.000772652	0.028597607	-0.593088355
JOSD1	-0.13710911	6.21433537	-3.502342015	0.000772041	0.028597607	-0.715977156
MRPL49	0.107147567	5.15600004	3.499875927	0.000778218	0.028606932	-0.703814523
HSPA1B	0.227913083	4.993090733	3.499352441	0.000779536	0.028606932	-0.704379804
SCAMP4	0.15015019	6.918833341	3.50044737	0.000776783	0.028606932	-0.720031274
ARF3	0.080787298	7.000143237	3.49896987	0.0007805	0.028606932	-0.722123285
TUT1	0.196600148	2.333310399	3.497838015	0.000783359	0.02862571	-0.609263551
LRIF1	0.165687714	3.627153635	3.49726388	0.000784813	0.02862571	-0.649299722
SNX30	-0.244535195	5.460957192	-3.496103237	0.00078776	0.028663799	-0.728625949
POLR2E	0.193585301	7.122543734	3.495247628	0.000789939	0.028673834	-0.733058953
ATF7	0.113710194	4.946653077	3.493418427	0.000794617	0.028774309	-0.715583196
TACO1	0.174387936	4.176246346	3.492379253	0.000797286	0.028801735	-0.689335288
E2F5	-0.267681205	1.062135124	-3.489618807	0.000804418	0.028920669	-0.670055365
GNG5	0.145804069	5.884153976	3.490047854	0.000803306	0.028920669	-0.749850851

Gene	Log fold change	Average Expression	t	P-value	Adjusted P-value	B
RPLP2	0.265542139	9.431971875	3.487048795	0.000811113	0.029015718	-0.700278816
TAF13	-0.22805042	4.724611693	-3.486388645	0.000812841	0.029015718	-0.728630403
RAVER1	0.162897469	4.732799426	3.48724581	0.000810598	0.029015718	-0.732688808
ZDHHC17	-0.30120139	4.007282439	-3.482832152	0.00082221	0.029211736	-0.706384375
DRG1	0.124168965	5.537865608	3.482896903	0.000822039	0.029211736	-0.765959403
FMN1	-0.371732035	2.862040545	-3.480227142	0.000829138	0.029339376	-0.668027
TNIP1	0.120140596	6.770837637	3.480017334	0.000829698	0.029339376	-0.781587501
TRMT2A	0.175109039	3.844730701	3.478357669	0.000834143	0.029350378	-0.719728442
NRBP1	0.08896822	7.558028906	3.478276494	0.000834361	0.029350378	-0.771501855
TCOF1	0.128495127	5.330968238	3.477721047	0.000835855	0.029350378	-0.77487027
CHST14	0.11947798	5.909258369	3.476235618	0.00083986	0.02942244	-0.789780889
PER2	-0.263281962	3.070457678	-3.475037887	0.000843103	0.029429856	-0.669473817
SNRPG	0.242993346	3.387187411	3.472700477	0.000849465	0.029429856	-0.706449972
AHCTF1	-0.227999902	4.034760832	-3.473886834	0.00084623	0.029429856	-0.740918709
HS6ST1	-0.21439709	4.628138271	-3.4725634	0.00084984	0.029429856	-0.761239989
GNB2	0.179603317	7.466947938	3.474154358	0.000845502	0.029429856	-0.789130494
TMEM170B	-0.301909745	2.7584743	-3.470864072	0.000854496	0.029523227	-0.689928627
KIAA1841	-0.199268224	2.714012615	-3.468191665	0.000861867	0.029660275	-0.689569437
FAM122B	-0.150135131	3.634101005	-3.467289351	0.000864369	0.029660275	-0.734294962
C5orf51	-0.185909895	5.562360364	-3.467297115	0.000864348	0.029660275	-0.809677637
C1GALT1	-0.224360468	5.544368065	-3.466563983	0.000866386	0.029661906	-0.80869787
USP31	-0.203218013	2.433152368	-3.463934386	0.000873734	0.029693664	-0.701546083
DTWD2	-0.394545503	1.27694599	-3.465312675	0.000869875	0.029693664	-0.723909095
MIS12	-0.168338262	3.776767353	-3.464521498	0.000872088	0.029693664	-0.750288254
ZNF580	0.260485765	4.463951641	3.462669126	0.00087729	0.029693664	-0.783492419
SMARCA4	0.094322568	6.6391332	3.46246802	0.000877857	0.029693664	-0.831842393
ADRM1	0.197119232	5.999633909	3.462012803	0.000879141	0.029693664	-0.833035606
SOBP	0.245897217	3.895379749	3.461293787	0.000881172	0.029695687	-0.774850291
FUT11	-0.166741236	4.945652816	-3.457266392	0.000892631	0.029907223	-0.828584475
NR3C1	-0.229405031	7.480725544	-3.456063584	0.000896081	0.029907223	-0.839765208
WSB2	-0.185831007	5.702922819	-3.457789965	0.000891134	0.029907223	-0.840918484
TMED5	-0.206058053	5.854946392	-3.456281951	0.000895454	0.029907223	-0.848495385
EEF1D	0.176417868	6.371070631	3.455613336	0.000897376	0.029907223	-0.85323037
CCSER2	-0.170654134	5.722479411	-3.45174387	0.000908574	0.030213599	-0.859136323
MOB4	-0.159322178	4.220203624	-3.449383833	0.000915469	0.030366828	-0.811385905
SESN2	0.395637511	5.719769829	3.448789308	0.000917214	0.030366828	-0.856950824
NR2F6	0.187641415	4.563286301	3.446563874	0.000923772	0.030450123	-0.840657661
SDHA	0.09748891	6.127631499	3.446580209	0.000923724	0.030450123	-0.878516498
LSM14A	-0.085545987	6.600632423	-3.44531542	0.000927471	0.030505292	-0.882810069
CST3	0.207252523	8.492572375	3.441697054	0.00093827	0.03079325	-0.857525974
NAPA	0.127624891	6.13400156	3.438646825	0.000947466	0.031027446	-0.901684335
PAXIP1	-0.221323028	2.953098513	-3.436654408	0.000953518	0.031090477	-0.798847337
VAPA	-0.083433267	6.910609197	-3.436804727	0.00095306	0.031090477	-0.904670118
LARP1	-0.184371843	7.448734189	-3.433814581	0.000962208	0.031306057	-0.900518069
ZBTB1	-0.191035651	5.146071725	-3.432617911	0.000965892	0.031358195	-0.895698196
NDUFV3	0.166476059	4.988188979	3.431045358	0.000970754	0.031448255	-0.898008171
SLC7A11	-0.356300419	4.790378446	-3.429215437	0.00097644	0.031564588	-0.900358897
BCL2	-0.27111198	3.944966011	-3.428370388	0.000979077	0.031582045	-0.856432789
TIGAR	0.207591033	4.312602112	3.427014323	0.000983322	0.031643358	-0.865521297
ZADH2	-0.161042202	4.528999418	-3.426422845	0.000985179	0.031643358	-0.907272198
AASDHPPT	-0.099331932	4.728699671	-3.424700901	0.000990604	0.031682498	-0.912264198
HSF1	0.150412469	6.387354282	3.425110911	0.00098931	0.031682498	-0.941688975
IQSEC2	0.142677209	3.699378763	3.419694004	0.001006539	0.031807415	-0.867385474
SLC43A2	0.230551923	4.160040614	3.419942449	0.001005742	0.031807415	-0.902691227
HNRNPUL1	0.076738312	8.256637085	3.420724142	0.001003241	0.031807415	-0.921474945
MT2A	0.248209618	7.278274126	3.420113226	0.001005195	0.031807415	-0.947049203
ATG12	-0.104154667	5.833501327	-3.419494546	0.001007179	0.031807415	-0.953873386
SYDE2	-0.446272152	0.822354332	-3.422408464	0.00099787	0.031807415	-0.954788719
SCRIB	0.117085301	5.840180126	3.417176578	0.001014642	0.031909318	-0.958199111
ANKRD28	-0.208111332	6.333690819	-3.417750301	0.00101279	0.031909318	-0.962783304
MIR137HG	-0.350063037	0.657027117	-3.415035493	0.001021582	0.032060643	-0.9197988
CHML	-0.265696437	3.373068988	-3.414021162	0.001024885	0.032097446	-0.875675513
JARID2	-0.251359992	3.250508947	-3.411223284	0.00103405	0.032312925	-0.881970465
FAM180A	-0.238713076	5.276057543	-3.410613958	0.001036056	0.032312925	-0.958214693
GRIN2D	0.225279984	3.994546724	3.409099996	0.001041056	0.032319382	-0.915102137
NOC2L	0.122009782	5.55895829	3.408603967	0.001042699	0.032319382	-0.981364732
LINC00704	-1.227306787	-3.506058157	-3.409561332	0.00103953	0.032319382	-2.223889354
KRCC1	0.191231614	4.695084235	3.405791032	0.001052064	0.032417482	-0.961853703
REPIN1	0.107498587	5.134372614	3.40571447	0.00105232	0.032417482	-0.978515985
SRGAP1	-0.3039384	6.088752548	-3.406252287	0.001050523	0.032417482	-0.995921761

Gene	Log fold change	Average Expression	t	P-value	Adjusted P-value	B
LDLRAP1	0.144265179	4.813476688	3.404377188	0.001056801	0.032489079	-0.965979657
AEN	0.279893016	4.984272254	3.400549773	0.001069725	0.032686291	-0.964734853
SSH3	0.151333004	4.657992923	3.401003183	0.001068186	0.032686291	-0.96916844
THOC2	-0.216677098	5.051311387	-3.400558591	0.001069695	0.032686291	-0.988516455
ARFGAP3	-0.255012725	5.557324152	-3.397976313	0.001078499	0.032887669	-1.015923526
GTF2F1	0.125635454	7.076808228	3.39675898	0.001082673	0.032948249	-1.018244962
USP16	-0.132248844	5.647831324	-3.391856791	0.001099636	0.033396996	-1.031619941
CDK16	0.087212562	6.650804306	3.389719367	0.00110711	0.033556339	-1.042994678
LSM11	-0.279140795	2.549601979	-3.387649828	0.001114392	0.033574405	-0.922612736
GOPC	-0.175624162	5.188147742	-3.388145596	0.001112643	0.033574405	-1.026310232
ABCF3	0.10804285	5.711367533	3.388431889	0.001111635	0.033574405	-1.043394469
ZNF414	0.255210049	3.271756761	3.383285078	0.001129899	0.033973658	-0.946819878
NAT10	0.114498884	5.644662629	3.381145366	0.001137575	0.034136325	-1.064390081
TMEM160	0.385279832	1.813599221	3.379031946	0.001145205	0.034296965	-0.93217762
UBA1	0.102017569	8.558920749	3.377883069	0.001149373	0.034353491	-1.034554164
DYRK2	-0.162804942	5.512439508	-3.375432835	0.00115831	0.034552049	-1.076002851
DUSP3	-0.126881295	6.272464369	-3.372330759	0.001169718	0.034823398	-1.09312359
PRKCSH	0.130604783	9.039245585	3.371692057	0.001172081	0.034824893	-1.039605085
CYB561	0.109129542	5.571384957	3.370083307	0.00117805	0.034933352	-1.092618053
PARP12	0.18167509	3.028443045	3.366265588	0.00119233	0.034960111	-0.984336755
CMTM7	0.227378011	3.297640628	3.365881035	0.001193778	0.034960111	-0.998022364
RPL13	0.198726635	9.914367865	3.366714395	0.001190643	0.034960111	-1.028839657
AAAS	0.16829222	4.750213602	3.367918683	0.001186127	0.034960111	-1.066124119
SAMD8	-0.222563773	4.744776342	-3.36749204	0.001187725	0.034960111	-1.066497376
TRAF7	0.099928325	7.24958236	3.367167269	0.001188943	0.034960111	-1.100266123
TCF12	-0.223016789	6.687911214	-3.365504276	0.001195198	0.034960111	-1.111898848
ZNF33B	-0.179978185	2.62799406	-3.361450994	0.001210573	0.035028715	-0.979011164
TUBB	0.117229823	9.807866447	3.361209341	0.001211495	0.035028715	-1.043842349
TRAPPC6B	-0.141769168	4.345233114	-3.36293628	0.001204917	0.035028715	-1.057924207
GNAI2	0.143996022	8.617416378	3.362116148	0.001208037	0.035028715	-1.079178163
PPP2R1A	0.114602285	8.146183873	3.362388771	0.001206999	0.035028715	-1.090401255
ARHGAP1	0.11244736	7.889616429	3.361579678	0.001210082	0.035028715	-1.099594682
NUCB1	0.118766391	9.170904291	3.358597436	0.001221508	0.035250571	-1.072295253
HOMER1	-0.338722194	2.313763299	-3.356654327	0.001229008	0.035346397	-0.999887935
UBTD2	-0.127260662	6.235896702	-3.356521627	0.001229522	0.035346397	-1.138396145
BCKDK	0.146580296	5.512009376	3.354985528	0.001235483	0.035450134	-1.130965837
MED19	0.176642954	3.80663775	3.353507255	0.001241246	0.035480332	-1.062710487
FAM160B2	0.08357867	5.935836743	3.354087434	0.001238981	0.035480332	-1.141254409
KIT	-0.673830843	3.397774175	-3.352502303	0.001245179	0.035525322	-1.013612641
SYN1	0.369920227	0.138262675	3.351695606	0.001248344	0.035548293	-1.140974765
BRMS1L	-0.125402824	3.461705376	-3.348540923	0.001260793	0.03577258	-1.059351786
PRKAG2	-0.168530142	3.425174674	-3.348496591	0.001260969	0.03577258	-1.063394519
KAT2B	-0.211842747	4.178412729	-3.347710213	0.001264091	0.035793739	-1.095833704
MAP4K3	-0.240631458	4.112261692	-3.346745046	0.001267933	0.035835161	-1.101934002
NME2	0.220731726	4.256950776	3.3443204	0.001277632	0.035854331	-1.108590479
STAM2	-0.189595944	4.997047517	-3.34544267	0.001273134	0.035854331	-1.142210545
G6PD	0.1831213	7.225634283	3.344196062	0.001278132	0.035854331	-1.165433642
LIMS1	-0.17406281	6.51713682	-3.344471554	0.001277026	0.035854331	-1.171056345
KIF1C	0.119046679	7.700806025	3.34320084	0.001282135	0.035899769	-1.157117747
EFNA4	0.194956443	1.957626822	3.341346308	0.001289625	0.035907729	-1.031312162
GFER	0.200304627	3.237679104	3.339106742	0.001298726	0.035907729	-1.072422418
DCUN1D1	-0.155759649	4.313576664	-3.337846933	0.001303872	0.035907729	-1.133648822
DST	-0.280954623	7.942679674	-3.341891725	0.001287418	0.035907729	-1.154895173
EXOC6B	-0.19109544	5.194681598	-3.340083627	0.001294749	0.035907729	-1.168344721
PPDPF	0.276939813	7.479238945	3.339871823	0.00129561	0.035907729	-1.174816007
ZMIZ2	0.115814512	5.812745499	3.339892813	0.001295525	0.035907729	-1.18004141
MOB1A	-0.149419019	6.916802211	-3.338231709	0.001302298	0.035907729	-1.185914521
LCTL	-0.99744792	-3.015420429	-3.338004348	0.001303228	0.035907729	-2.119382683
ZNF771	0.234002479	3.258897348	3.330843858	0.001332829	0.036384214	-1.095475988
MPV17L2	0.171880707	3.168823823	3.33160136	0.001329668	0.036384214	-1.103214007
ACOT2	0.145452113	3.497236462	3.330515383	0.001334202	0.036384214	-1.114699836
BTBD2	0.190339129	7.752510379	3.331148917	0.001331555	0.036384214	-1.192814522
EHD4	-0.191977059	5.474408355	-3.331458949	0.001330261	0.036384214	-1.19931915
PYHIN1	1.154908686	-3.438538938	3.330165458	0.001335666	0.036384214	-2.38310836
RTP4	0.27735815	1.140072407	3.327720583	0.001345937	0.036531891	-1.079176557
C20orf27	0.184478064	5.668260833	3.327902967	0.001345169	0.036531891	-1.211625434
CDCA4	-0.148719665	3.00130011	-3.322807824	0.001366802	0.036679681	-1.110569675
LIMD1	-0.249726443	3.674017102	-3.324871282	0.001358002	0.036679681	-1.165064041
CARD6	0.183501306	4.86330035	3.323016648	0.001365909	0.036679681	-1.204041579
FNDC3B	-0.286045956	7.321398866	-3.325047705	0.001357252	0.036679681	-1.212706598

Gene	Log fold change	Average Expression	t	P-value	Adjusted P-value	B
SHOC2	-0.152108145	6.195389656	-3.323399216	0.001364274	0.036679681	-1.229713343
HDGFRP2	0.199959874	6.318456763	3.324034632	0.001361563	0.036679681	-1.230081364
ARPP19	-0.137260382	6.069482637	-3.322428195	0.001368427	0.036679681	-1.234049252
RXRB	0.137754869	4.913670393	3.320497235	0.00137672	0.036836425	-1.210441726
FMNL1	0.213760827	3.488331792	3.318926576	0.0013835	0.036886811	-1.136559388
RAD1	-0.157218541	4.710254585	-3.319007627	0.00138315	0.036886811	-1.201356696
SEC13	0.137162878	7.107981143	3.318081976	0.001387159	0.036919026	-1.24057366
CNPPD1	0.145913139	5.611593759	3.317018068	0.001391781	0.036976713	-1.240645567
FMR1	-0.171279567	4.346495581	-3.316372534	0.001394593	0.036986179	-1.202474986
EIF5A2	-0.215719897	3.784739633	-3.315726961	0.00139741	0.036995757	-1.148752197
HGH1	0.150098359	4.649972896	3.313875178	0.00140552	0.037145192	-1.223326493
YPEL2	-0.238986113	2.984925397	-3.308595941	0.001428886	0.037695349	-1.168715249
ZC3H4	0.160530868	4.74804751	3.30804558	0.001431342	0.037695349	-1.241858152
KDELR1	0.13339378	8.391929352	3.307224794	0.001435013	0.037726079	-1.240531042
SPATA2	0.149397931	4.163039709	3.306209867	0.001439565	0.037741235	-1.218012687
AMD1	-0.244101583	5.714381529	-3.305979408	0.001440601	0.037741235	-1.278449619
DOCK11	-0.194329512	5.139383708	-3.30495146	0.001445228	0.037796725	-1.263013408
COG7	0.15330303	3.509966312	3.301684392	0.001460027	0.038117588	-1.173451256
SLC9A3R1	0.147594118	3.912537081	3.299860619	0.00146835	0.038245542	-1.218986076
IRAK4	-0.118493443	4.095342833	-3.299498988	0.001470006	0.038245542	-1.226112263
TBC1D12	-0.236770324	3.43117724	-3.296651039	0.001483106	0.038513914	-1.205294926
BLOC1S6	-0.08898682	6.245480963	-3.296147342	0.001485434	0.038513914	-1.308487338
PCGF2	0.169315023	5.680560689	3.293341563	0.001498467	0.038785064	-1.308783625
PIP5K1C	0.117305307	7.316712773	3.292305718	0.001503306	0.038843559	-1.308891396
FAM111A	-0.203126334	4.714747735	-3.290888673	0.001509948	0.038902084	-1.277404126
USP25	-0.096583105	5.541046678	-3.290721171	0.001510735	0.038902084	-1.314973793
NEK11	-0.154888879	3.942748173	-3.288319064	0.001522065	0.039126933	-1.2480261
STC1	-0.343544231	3.542155622	-3.287317446	0.001526812	0.039182112	-1.220217839
C5orf24	-0.215100335	5.657242905	-3.284867496	0.001538483	0.039414479	-1.335870552
ZCCHC17	0.152959109	4.470208016	3.283261393	0.00154618	0.039477381	-1.29112155
EIF5	-0.241123864	7.200811647	-3.283614548	0.001544485	0.039477381	-1.329637763
NAT6	0.231201348	2.422255849	3.281696106	0.001553716	0.039535766	-1.19238644
IRF2BP1	0.174183684	4.986963867	3.281909414	0.001552687	0.039535766	-1.320444793
STK11	0.132619967	5.810760581	3.280029901	0.001561775	0.039673831	-1.347553601
TAF3	0.144871748	4.130986198	3.276825619	0.001577385	0.03968439	-1.289625888
CLN6	0.146880216	4.342114266	3.278514501	0.001569139	0.03968439	-1.304933136
XPOT	-0.14193751	7.676060488	-3.278098339	0.001571167	0.03968439	-1.340807351
IER5	0.221257494	6.031800192	3.276700527	0.001577997	0.03968439	-1.346655476
MAX	0.074806077	5.949285316	3.278038818	0.001571458	0.03968439	-1.357316635
PELP1	0.133215071	5.941454846	3.277150285	0.001575797	0.03968439	-1.360021674
UBASH3B	-0.307432924	3.413826286	-3.274809458	0.001587282	0.039851348	-1.281244606
TGFBR1	-0.346577391	6.723783455	-3.274179157	0.001590387	0.039857672	-1.366142515
ARAF	0.114494063	5.751858913	3.273685244	0.001592825	0.039857672	-1.366166058
PARP16	0.172614515	2.694356881	3.271708739	0.001602616	0.040028063	-1.23037535
TRIP10	0.168449603	5.785428556	3.271239386	0.001604949	0.040028063	-1.372800924
SHISA5	0.118408216	7.972097666	3.267025281	0.001626041	0.040143431	-1.364732373
GTF3C5	0.137060607	5.239322757	3.267893603	0.001621674	0.040143431	-1.366388909
UBA3	-0.098071671	5.229684686	-3.266892942	0.001626707	0.040143431	-1.371121289
STX6	-0.131672582	5.570593247	-3.268542792	0.001618416	0.040143431	-1.375131148
ACAP2	-0.202696141	5.786501239	-3.268856283	0.001616845	0.040143431	-1.381430194
ASAP3	0.12646411	6.725121096	3.267365537	0.001624328	0.040143431	-1.389172675
SF3A2	0.240555267	6.511873964	3.266591114	0.001628229	0.040143431	-1.391363817
PDP2	-0.368286053	3.563233644	-3.264973746	0.001636403	0.040234011	-1.305470371
FAM214B	-0.176446326	4.182907705	-3.264281119	0.001639915	0.040234011	-1.347710781
RASEF	-0.565178875	-0.655050916	-3.264479314	0.001638909	0.040234011	-1.52740906
NCDN	0.094862982	5.939578353	3.261233473	0.001655454	0.040549203	-1.403240603
ATP6AP1	0.104054614	6.981062297	3.259935307	0.001662115	0.040646261	-1.406165809
MXRA8	0.167210339	9.374462451	3.258645089	0.00166876	0.040742618	-1.345141328
SDF2L1	0.200688098	3.443700475	3.256905939	0.001677756	0.040895981	-1.308282158
NAA25	-0.273523309	3.741589155	-3.255769388	0.00168366	0.040954776	-1.357288984
AGFG1	-0.188052865	5.999771392	-3.255395632	0.001685605	0.040954776	-1.422235646
PABPC4	0.144555448	8.668545728	3.251054277	0.001708361	0.041440819	-1.387934351
ATP6V1B2	-0.241538413	7.147421766	-3.249502828	0.001716563	0.041543952	-1.429545357
YES1	-0.196243316	6.139752248	-3.248687792	0.001720886	0.041543952	-1.441270059
FSIP1	-0.401055975	0.233365003	-3.248939315	0.001719551	0.041543952	-1.44749516
PHF21A	0.168138067	5.12586995	3.247864174	0.001725265	0.041583033	-1.421122111
ZNRF1	0.152605094	4.04494041	3.246956093	0.001730106	0.041633089	-1.37686411
RAB7B	0.23035193	3.951820994	3.246294443	0.001733641	0.041651617	-1.374037331
VPS33B	0.145774213	3.279246016	3.243095103	0.001750829	0.04193083	-1.344363132
PRKCD	0.164638936	3.383479729	3.243538861	0.001748436	0.04193083	-1.361007089

Gene	Log fold change	Average Expression	t	P-value	Adjusted P-value	B
ZNRF2	-0.20777559	2.612814509	-3.241438668	0.001759791	0.042011876	-1.304447839
SCYL1	0.12072717	6.840004899	3.241911026	0.001757231	0.042011876	-1.456149566
MGRN1	0.087971949	6.273007404	3.240063332	0.001767265	0.04212354	-1.465103175
CYTH1	-0.171267441	3.982642941	-3.238890275	0.001773663	0.042209252	-1.398375187
RBM6	-0.160025936	5.107470628	-3.237673171	0.001780324	0.042245308	-1.438633062
SMARCD2	0.08708772	5.813709675	3.237588782	0.001780787	0.042245308	-1.467653938
LOC100132215	0.831302401	-1.939247579	3.236879582	0.00178468	0.042271097	-1.952626504
GMIP	0.1824205	2.603977769	3.234386603	0.001798429	0.042446998	-1.330742707
CEP131	0.275945505	3.092239795	3.233072304	0.001805717	0.042446998	-1.334306453
PPARGC1B	-0.532472597	-0.028967557	-3.223499284	0.001859643	0.042446998	-1.334622208
SH3BP1	0.226072909	2.185738166	3.230490983	0.001820112	0.042446998	-1.33991139
POLA2	0.176799798	3.397014172	3.233363741	0.001804098	0.042446998	-1.368537556
TARS2	0.217450642	3.502339906	3.225208703	0.001849904	0.042446998	-1.383863307
DZIP3	-0.228172606	3.499124157	-3.226353498	0.001843409	0.042446998	-1.388693609
LRSAM1	0.149662718	4.307206016	3.229321366	0.001826669	0.042446998	-1.420076946
EDEM1	-0.233189349	4.499252887	-3.229959058	0.001823091	0.042446998	-1.449198736
MAFG	-0.16095839	5.073227565	-3.229825106	0.001823842	0.042446998	-1.453680338
MAD2L2	0.20781123	4.65037539	3.224433897	0.001854312	0.042446998	-1.462882202
TRAFD1	0.105429064	4.982243166	3.229011898	0.001828408	0.042446998	-1.468083839
ADA	0.26885043	4.774683029	3.223393161	0.001860249	0.042446998	-1.470763535
RAB11FIP2	-0.220383565	4.861563552	-3.225417151	0.00184872	0.042446998	-1.471461131
SNAP47	0.095599053	4.86463363	3.223434033	0.001860016	0.042446998	-1.475849844
GNA13	-0.262594275	5.840941588	-3.234583589	0.001797339	0.042446998	-1.479146119
MFSD5	0.097069549	6.030987389	3.232039482	0.001811463	0.042446998	-1.487217071
LMF2	0.151368622	6.882325409	3.23097191	0.001817422	0.042446998	-1.488249554
GNL3	-0.183463491	5.852258361	-3.230535173	0.001819864	0.042446998	-1.490536473
KARS	0.134076981	7.080145036	3.226533862	0.001842388	0.042446998	-1.496504029
TTC17	-0.176835815	5.724350638	-3.222990482	0.001862551	0.042446998	-1.504419547
NR1H2	0.155082448	6.305181585	3.225516768	0.001848154	0.042446998	-1.504844184
WFS1	0.137815212	6.041887148	3.224052895	0.001856484	0.042446998	-1.508468974
ATP13A1	0.108952219	6.022900255	3.223754172	0.001858188	0.042446998	-1.508623601
ACSS2	0.200821757	6.101399312	3.223009758	0.001862441	0.042446998	-1.510812563
MDH2	0.097737998	7.233953266	3.221897702	0.001868811	0.042525326	-1.506477805
SLC16A7	-0.361200485	5.038564364	-3.220607493	0.001876227	0.04262969	-1.489819882
RAI14	-0.21501422	6.442750697	-3.219229417	0.001884179	0.042745889	-1.521245511
EMD	0.176564332	5.839487486	3.217289144	0.001895428	0.042936436	-1.523191696
JAK2	-0.232807556	3.950715641	-3.213657297	0.001916654	0.042953677	-1.469083717
FOS	0.996011129	5.295860894	3.212874105	0.00192126	0.042953677	-1.503176731
BCOR	-0.341568133	4.543805943	-3.214410224	0.001912235	0.042953677	-1.508754588
PCIF1	0.118918359	5.111952748	3.215645812	0.001905005	0.042953677	-1.509200535
HSPBP1	0.196997226	5.067467851	3.214489607	0.00191177	0.042953677	-1.509355033
SLC27A4	0.12513797	5.433798151	3.215261915	0.001907249	0.042953677	-1.522231832
C19orf43	0.201482048	6.947133289	3.214599535	0.001911126	0.042953677	-1.532737936
PSMD3	0.138372134	6.717412405	3.21277365	0.001921852	0.042953677	-1.539104534
RRAD	0.596579749	-0.756764208	3.213240921	0.001919102	0.042953677	-1.819158575
HSPB1	0.267175273	7.853606312	3.209652429	0.001940318	0.043302148	-1.527926757
ASXL1	-0.199632662	6.03118855	-3.208133274	0.001949366	0.04343971	-1.550802087
LOC100288846	0.771537301	-2.693934158	3.205798749	0.001963346	0.043686634	-2.135772941
EIF3B	0.091367551	7.872466218	3.205208006	0.001966899	0.04369266	-1.532200305
EPN1	0.168777489	7.833219788	3.204789721	0.001969418	0.04369266	-1.541029834
RILP	0.257778227	3.234898733	3.203195154	0.001979049	0.043841763	-1.439210135
CSRP1	0.149450737	7.230610016	3.200698177	0.001994219	0.044112955	-1.562715772
CTXN1	0.294842462	2.837676887	3.199537098	0.002001311	0.0442049	-1.426992985
GCLM	-0.23684171	5.13316337	-3.197105993	0.002016234	0.044404321	-1.569372304
MAP4K5	-0.148757573	5.920165537	-3.197490871	0.002013865	0.044404321	-1.579773195
TMEM253	0.614027066	-2.232035075	3.194946023	0.002029581	0.04463301	-2.045774717
ZNF281	-0.228324399	5.148538314	-3.193829527	0.002036513	0.044633972	-1.561512359
RAB11B	0.169381005	6.45008476	3.193835449	0.002036476	0.044633972	-1.592597853
PPM1E	-0.788085819	-2.028168137	-3.193507824	0.002038514	0.044633972	-2.03715069
WIPF1	-0.163935628	6.825158531	-3.191534334	0.002050831	0.044838489	-1.597747748
E2F4	0.101167968	5.783921943	3.190840808	0.002055176	0.044868366	-1.598301968
C17orf53	0.249994594	1.180980984	3.189858014	0.002061348	0.044873047	-1.419804154
XRCC6	0.07948971	8.083173543	3.1902856	0.002058661	0.044873047	-1.570500813
KIAA1958	-0.283683497	2.569847404	-3.188828848	0.00206783	0.044884426	-1.441708549
ARNT2	-0.266436981	4.590755291	-3.188851387	0.002067688	0.044884426	-1.564609813
PSMC4	0.148408795	5.951134861	3.188150484	0.002072113	0.044912675	-1.605444155
ABCB6	0.339448729	0.952966416	3.187460084	0.00207648	0.044940909	-1.463807104
PCMTD2	-0.178361285	4.41777094	-3.18688982	0.002080094	0.044940909	-1.555784053
SENP6	-0.118797232	5.745022251	-3.186531881	0.002082366	0.044940909	-1.608866049
C2CD2	-0.194231218	4.662901941	-3.184828411	0.002093207	0.045045815	-1.581166367

Gene	Log fold change	Average Expression	t	P-value	Adjusted P-value	B
TALDO1	0.142305938	6.876372072	3.185079033	0.002091609	0.045045815	-1.613865775
PDE12	-0.189777838	4.810012686	-3.183275799	0.002103134	0.045194886	-1.588806825
THEMIS2	0.229615733	2.038766014	3.182499935	0.002108112	0.0451996	-1.448664365
PPP1R16A	0.205147628	3.403161881	3.182306427	0.002109355	0.0451996	-1.504599536
GALK1	0.224551337	4.584795212	3.180530605	0.002120795	0.045279892	-1.574263802
BOP1	0.189010486	5.170160483	3.178007508	0.002137148	0.045279892	-1.618838154
TBC1D13	0.098356789	5.61479244	3.179430003	0.002127914	0.045279892	-1.624674542
YIF1A	0.176794637	5.928268544	3.180478535	0.002121131	0.045279892	-1.62502126
PRKAA1	-0.272244296	5.663321824	-3.179618457	0.002126693	0.045279892	-1.626652671
SH3D19	-0.112010418	7.199078096	-3.17877284	0.002132175	0.045279892	-1.629113558
WDR46	0.107066324	5.745658002	3.178482404	0.002134061	0.045279892	-1.630610504
ADAM15	0.124603711	6.794138426	3.178181335	0.002136018	0.045279892	-1.633408664
TAF6L	0.192000931	3.110490782	3.176785317	0.002145112	0.045384796	-1.49797544
C8orf82	0.188500786	4.34838493	3.17524605	0.002155182	0.045533891	-1.581634049
EOGT	-0.22350937	4.095278724	-3.174087839	0.002162788	0.045548393	-1.586536827
LZTFL1	-0.100883583	4.597979618	-3.174289292	0.002161463	0.045548393	-1.599652371
ATP13A3	-0.464364115	6.929385497	-3.173760932	0.00216494	0.045548393	-1.628938255
DPM2	0.1804949	4.71933195	3.172617633	0.002172479	0.045605426	-1.609400274
RCBTB1	-0.140241208	4.786217446	-3.17243207	0.002173705	0.045605426	-1.614674994
NANS	0.152377715	5.030189328	3.170845147	0.002184217	0.045698667	-1.632441884
ACTA2	0.14607822	6.35846942	3.171032092	0.002182976	0.045698667	-1.654543687
PRR16	-0.126062045	4.47269225	-3.16930588	0.002194458	0.045849254	-1.613579477
FDFT1	0.158618171	7.369933844	3.168425117	0.002200338	0.045908435	-1.647518677
GTPBP4	-0.251814122	5.73218001	-3.167509476	0.002206466	0.045972629	-1.658751304
ZCCHC2	-0.307142464	3.576307931	-3.164331081	0.002227864	0.045994102	-1.58572858
PEX14	0.18131809	3.954841805	3.164182398	0.00222887	0.045994102	-1.587030703
CIR1	0.142691427	5.297025115	3.166036233	0.002216361	0.045994102	-1.651664258
TECR	0.186968777	5.432087656	3.164381317	0.002227524	0.045994102	-1.658674438
ARF6	-0.132945291	6.576744563	-3.164924236	0.002223856	0.045994102	-1.671045277
RAB8B	-0.190089341	6.316743594	-3.164407726	0.002227346	0.045994102	-1.673030037
ZFP2	0.471516713	-0.631515057	3.166305309	0.00221455	0.045994102	-1.695488055
TNPO3	0.085701847	5.984627984	3.162808108	0.002238186	0.046060148	-1.674719762
TBC1D17	0.147161123	6.492818403	3.163237984	0.002235268	0.046060148	-1.676195149
PSMA6	0.182200139	3.041585888	3.161839684	0.002244772	0.046132669	-1.544766611
RABGEF1	0.203955827	1.459412946	3.161297141	0.00224847	0.046145709	-1.491434617
ARHGEF10L	0.119605703	5.046495641	3.160104804	0.002256617	0.046249895	-1.658463262
FJX1	-0.196915654	3.784608156	-3.159397182	0.002261465	0.046277058	-1.597010676
MVD	0.2631572	6.624514458	3.159015122	0.002264086	0.046277058	-1.68409664
ST6GALNAC6	0.10763454	7.106005849	3.15819744	0.002269706	0.046329069	-1.683275331
CHAF1B	0.20312958	2.3926205	3.157323781	0.002275725	0.046389075	-1.512012882
SLC4A4	-0.323283236	0.476354238	-3.156046567	0.002284552	0.046443304	-1.524921722
SPRYD3	0.103102689	5.81105075	3.156160199	0.002283765	0.046443304	-1.691379368
CCDC50	-0.126469948	6.613315298	-3.154997548	0.002291825	0.046528375	-1.698094546
DDX5	-0.226704298	8.4589716	-3.152205466	0.00231129	0.046860384	-1.667042156
DMTF1	-0.213506001	3.650856751	-3.151117596	0.002318915	0.046887159	-1.587407017
PSMB9	0.219359029	4.013991351	3.150263255	0.00232492	0.046887159	-1.628508428
SOWAHC	-0.225038747	4.613073255	-3.150985384	0.002319843	0.046887159	-1.635104488
HSDL1	-0.141839201	4.146225073	-3.149673077	0.002329077	0.046887159	-1.649268344
WDR17	-0.443948391	-0.360056314	-3.148098943	0.002340198	0.046887159	-1.664872828
HRH1	-0.245150601	4.530205225	-3.148194828	0.002339519	0.046887159	-1.67725057
TMEM65	-0.190155214	5.479330044	-3.148038913	0.002340623	0.046887159	-1.703785942
TAF15	0.119554765	6.857626642	3.149908138	0.00232742	0.046887159	-1.70973891
UAP1	-0.205303269	6.85163187	-3.14853379	0.002337121	0.046887159	-1.710099464
LDOC1	0.214769521	5.584249096	3.146284151	0.00235308	0.04707411	-1.710527722
GPANK1	0.120584937	3.942276909	3.14463555	0.002364841	0.047121642	-1.661819842
THAP12	-0.163024207	4.413666259	-3.144871057	0.002363157	0.047121642	-1.678886595
ATN1	0.134831582	7.903683782	3.145387459	0.00235947	0.047121642	-1.703579222
AQP5	0.906940955	-3.519287679	3.142427531	0.002380678	0.047374542	-2.49330272
PNO1	-0.149258015	4.119826183	-3.141042114	0.002390665	0.047510523	-1.670328191
ENOX2	-0.113028003	4.325429542	-3.139776218	0.002399825	0.047567053	-1.680483526
SNX17	0.114540064	7.333446678	3.139996932	0.002398225	0.047567053	-1.728953904
DDX41	0.100429518	5.643280065	3.137189007	0.002418647	0.047877142	-1.738771751
ZBTB24	-0.193193562	3.745653668	-3.136458206	0.002423989	0.047919911	-1.665351025
RTL8C	0.171286199	7.719794511	3.133808344	0.00244345	0.048241331	-1.736811213
HGS	0.095999424	6.553119866	3.132922184	0.002449991	0.048307152	-1.757860496
IFITM1	0.212515948	5.584891936	3.132269075	0.002454822	0.048339134	-1.748742458
H1FX	0.267759728	4.979876715	3.131630459	0.002459554	0.048369094	-1.72498081
CDC25C	0.469217888	-0.199334532	3.131104206	0.00246346	0.048382747	-1.743484285
KLF11	-0.249394441	4.000951792	-3.12986329	0.002472694	0.048500862	-1.671952091
WDR26	-0.11998893	7.247463343	-3.129419042	0.002476007	0.0485027	-1.759958819

Gene	Log fold change	Average Expression	t	P-value	Adjusted P-value	B
LOXL1	0.165933681	7.685087268	3.127090447	0.002493443	0.048717549	-1.753985208
NDUFS8	0.222718801	5.666703128	3.127309156	0.002491801	0.048717549	-1.763554942
MAPK8IP2	0.36376192	-0.404723792	3.125618333	0.002504525	0.048870683	-1.761806609
USP37	-0.417599462	1.859431593	-3.125090915	0.002508507	0.048885051	-1.599445773
EPHX1	0.18062147	7.56821241	3.124137984	0.002515715	0.04896219	-1.766327954
KDM4D	0.341761179	-0.23677035	3.122371597	0.002529129	0.049159733	-1.676644045
CNTROB	0.10368056	4.247171677	3.121586589	0.002535111	0.049212517	-1.728318369
EXOC8	-0.144317639	3.853847368	-3.12062043	0.002542492	0.04929228	-1.694088618
CCT6A	0.073746865	7.47940173	3.119326652	0.002552408	0.049367872	-1.778125806
CBFB	-0.123505568	5.350373981	-3.119256578	0.002552946	0.049367872	-1.782553917
GABPB2	-0.161662263	3.036022441	-3.118403704	0.002559503	0.049412887	-1.650065368
LAS1L	0.12774909	4.721972991	3.118101027	0.002561834	0.049412887	-1.759283599
ARHGEF17	-0.168847692	5.774788109	-3.117408551	0.002567174	0.049452569	-1.796784233
BSDC1	-0.08802588	5.617307368	-3.116338463	0.002575447	0.049548567	-1.786894486
CYB5R4	-0.153057673	4.086529414	-3.114344701	0.002590926	0.049719381	-1.73057782
EIF4A3	0.118166246	6.169900897	3.114408628	0.002590429	0.049719381	-1.808132713
RHOA	0.074906506	9.484159322	3.113203086	0.002599829	0.049826746	-1.731486284
CD248	0.186493757	10.71254685	3.112668007	0.002604011	0.04984349	-1.693259367
