# Supplementary material for: Comparison of time and dose dependent gene expression and affected pathways in primary human fibroblasts after exposure to ionizing radiation
Source: Mol Med. 2020 Sep 9;26:85. doi: 10.1186/s10020-020-00203-0 (PMC7488023; doi:10.1186/s10020-020-00203-0)
Supplement: Supplementary file 10 — Additional file 10: Web Table 1C. Differentially expressed genes 2 h after exposure to high dose ionizing radiation (2 Gray). [file 10020_2020_203_MOESM10_ESM.pdf]

Web Table 1C: Differentially expressed genes 2 hours after exposure to high dose ionizing radiation (2 Gray).

Gene	Log fold change	Average Expression	t	P-value	Adjusted P-value	B
CDKN1A	1.528362877	9.578941307	28.31804734	2.12E-42	3.19E-38	84.03063643
BBC3	1.949777049	4.023738717	24.84443753	1.85E-38	1.39E-34	75.58248136
RNF19B	1.328400864	3.164707185	24.50784342	4.69E-38	2.36E-34	73.99741243
PPM1D	1.111784618	4.428273031	22.89888567	4.65E-36	1.75E-32	70.71042278
RAP2B	0.743209346	6.391043181	17.79530089	5.68E-29	1.71E-25	55.35260264
ARID3A	1.198085947	3.536743634	17.37594512	2.47E-28	6.21E-25	53.49513599
PPP1R10	-1.177326775	5.684207763	-17.22732293	4.19E-28	9.01E-25	53.37100848
BTG2	1.971750067	4.197394101	15.75564466	8.95E-26	1.69E-22	47.93943305
SESN1	0.832635125	4.358072382	15.51945005	2.17E-25	3.64E-22	47.16276069
TOB1	0.83523574	5.784883154	15.46908913	2.63E-25	3.96E-22	47.10672284
PLK3	1.07330323	4.367813593	15.23240947	6.44E-25	8.82E-22	46.1765927
IRF2BP2	0.650223875	6.155245929	15.17719219	7.95E-25	9.97E-22	46.02467468
CITED2	-1.266489564	7.284362326	-15.12587964	9.66E-25	1.12E-21	45.81409043
DEDD2	0.959448837	4.046853599	14.75857194	3.96E-24	4.26E-21	44.26031467
RSRC2	1.0023542	5.112437589	13.75905355	2.01E-22	2.02E-19	40.56786033
MDM2	0.944877486	6.788792003	12.8995805	6.50E-21	6.12E-18	37.17547379
DCP1B	0.583965784	4.061718856	12.88379557	6.94E-21	6.15E-18	37.02946288
TIGAR	0.748892714	4.312602112	12.72480491	1.33E-20	1.12E-17	36.43504267
EGR2	-1.601619914	3.221774446	-12.488617	3.54E-20	2.81E-17	35.4631009
SPRY2	-1.103282424	4.381484668	-12.22465653	1.06E-19	8.00E-17	34.41767434
ZNF441	0.890544437	3.029300148	12.1458702	1.48E-19	1.06E-16	33.87231325
TRIB1	-0.909607925	3.62736882	-12.03567474	2.34E-19	1.60E-16	33.62486117
ITPKC	0.666307901	4.639786348	12.0036302	2.68E-19	1.76E-16	33.50292295
SLC25A25	-0.898548111	3.769731825	-11.50181685	2.24E-18	1.40E-15	31.41280136
MYC	-1.42019061	4.969945963	-11.48662343	2.39E-18	1.44E-15	31.35891369
ZNF281	-0.800262192	5.148538314	-11.4086083	3.33E-18	1.93E-15	31.03163448
CSRNP1	-1.262313869	3.571844999	-11.25153481	6.51E-18	3.63E-15	30.36985851
KATNBL1	-0.64858136	3.238532637	-11.01838946	1.77E-17	9.53E-15	29.35568046
TIGD2	-0.728808434	2.481001479	-10.94933012	2.39E-17	1.24E-14	28.9665302
TMCC1	-0.350809641	4.187774405	-10.8741126	3.30E-17	1.66E-14	28.76741438
NFKBIE	0.820692151	2.782827442	10.8274594	4.04E-17	1.96E-14	28.4708597
CLP1	0.461821421	3.834240506	10.66987104	7.98E-17	3.76E-14	27.89937849
FST	0.576220662	6.619768423	10.58114146	1.17E-16	5.36E-14	27.51832301
DCAF5	-0.273637541	5.784441832	-10.46617385	1.93E-16	8.43E-14	27.02602782
PHLDA3	0.513248439	7.237706873	10.46322137	1.96E-16	8.43E-14	27.01280405
DYRK3	0.651401112	4.28191998	10.38632797	2.74E-16	1.15E-13	26.69387805
CEP164	-0.41494889	4.464904601	-10.21735299	5.73E-16	2.33E-13	25.96888298
MARCH7	-0.516586875	5.848780365	-10.20179873	6.13E-16	2.43E-13	25.88679283
ARL4D	0.709199227	3.596746301	10.15650454	7.47E-16	2.83E-13	25.70253084
EGR1	-2.513741281	6.810030351	-10.15539898	7.51E-16	2.83E-13	25.69415346
EPHA2	1.086260556	3.036133609	10.12531063	8.56E-16	3.15E-13	25.56659505
BLOC1S2	0.373067778	5.890622378	10.10218725	9.48E-16	3.32E-13	25.4578349
ZNF649	0.654625946	2.65928391	10.11031714	9.14E-16	3.28E-13	25.4561801
AMOTL2	-1.829640284	6.437816275	-9.974240643	1.66E-15	5.68E-13	24.91008969
TP53INP1	0.866186327	5.508580613	9.959651443	1.77E-15	5.81E-13	24.846705
ABL2	-1.037047975	6.051190918	-9.959075508	1.77E-15	5.81E-13	24.83893734
BCL2L1	0.478022365	6.143834118	9.856598714	2.78E-15	8.73E-13	24.39257186
SIN3B	-0.343046361	5.12749706	-9.836093197	3.05E-15	9.33E-13	24.31647338
ZNF93	-0.934824773	1.484655632	-9.858419877	2.76E-15	8.73E-13	24.19832949
HILPDA	0.741653405	2.29563701	9.832386055	3.10E-15	9.33E-13	24.18417819
SMYD4	-0.523176524	2.515640738	-9.786174046	3.79E-15	1.12E-12	24.04751607
KLF7	-1.086050324	4.8151864	-9.753030884	4.39E-15	1.27E-12	23.95742309
ERF	1.128104365	6.219476716	9.73788184	4.69E-15	1.33E-12	23.87935432
IFFO2	0.79500948	3.495481602	9.695889606	5.64E-15	1.57E-12	23.72505856
RNF214	-0.344894358	3.427979864	-9.655369791	6.75E-15	1.82E-12	23.55028301
TMEM217	1.163401918	0.558342338	9.65514363	6.75E-15	1.82E-12	23.03559267
DDIT4	1.131357878	3.769428838	9.468413755	1.54E-14	4.07E-12	22.74428554
SLC38A9	-0.571153941	2.826221988	-9.39882326	2.09E-14	5.34E-12	22.43015645
PFKFB3	0.804546054	5.158914354	9.398841032	2.09E-14	5.34E-12	22.41315684
POLR1C	0.75549306	3.869990066	9.384844611	2.23E-14	5.59E-12	22.38011499
IL6	-1.951535878	1.982741516	-9.364974462	2.43E-14	6.00E-12	22.24380709
PTGS2	-1.930973378	2.583827388	-9.329107729	2.85E-14	6.92E-12	22.10743944
HOXA9	0.780866329	2.911502299	9.229106613	4.43E-14	1.06E-11	21.6925837
PHF21A	-0.479578481	5.12586995	-9.150977684	6.27E-14	1.47E-11	21.33736116
EPC1	-0.480989303	4.143940781	-9.101496713	7.80E-14	1.80E-11	21.14494112
REPIN1	-0.29065185	5.134372614	-9.098989761	7.89E-14	1.80E-11	21.10927784
DUSP7	0.746857876	4.922825719	9.009146854	1.17E-13	2.64E-11	20.71807419
CYR61	-2.515813293	7.172185715	-8.974641261	1.37E-13	3.03E-11	20.58050529
SOCS3	-1.357782309	5.479201848	-8.943032839	1.57E-13	3.44E-11	20.42082882

Gene	Log fold change	Average Expression	t	P-value	Adjusted P-value	B
PISD	0.443618132	4.044412885	8.863978617	2.24E-13	4.81E-11	20.11659619
CLK3	0.497754042	4.206251868	8.777745603	3.28E-13	6.95E-11	19.73335348
KLF6	-0.744898426	6.23785017	-8.77411525	3.33E-13	6.97E-11	19.67437557
RASD1	1.399944023	3.897393683	8.730569582	4.04E-13	8.22E-11	19.55310189
IRS2	-0.67329298	6.069927608	-8.743699067	3.81E-13	7.86E-11	19.54004064
EPHB3	0.990431639	5.116817974	8.720380528	4.23E-13	8.49E-11	19.48630831
CDKN1B	0.434148675	4.570327322	8.710315712	4.42E-13	8.76E-11	19.43846692
FGF2	0.49417361	5.899448548	8.671328832	5.25E-13	1.03E-10	19.22301052
ZNF2	0.666600897	2.414994417	8.637711723	6.10E-13	1.17E-10	19.12075261
EVC2	-0.362801271	4.110388829	-8.628322668	6.36E-13	1.20E-10	19.09249518
ARID5B	-0.726114508	7.10762133	-8.63554957	6.16E-13	1.17E-10	19.07186096
ZNF79	0.62693351	2.889914548	8.604428432	7.07E-13	1.30E-10	19.00629957
TRIM11	0.435038825	3.847052932	8.605431719	7.04E-13	1.30E-10	18.99594615
TRAF4	0.474529635	4.04860766	8.597020593	7.31E-13	1.33E-10	18.93824199
ZNF786	0.742793577	1.961840646	8.554779971	8.81E-13	1.58E-10	18.7148888
ZNF555	0.574592511	2.23977075	8.541915515	9.33E-13	1.65E-10	18.69824095
HPS6	0.467223302	4.726919039	8.481977128	1.22E-12	2.13E-10	18.43009972
TTI1	-0.383308378	4.141844402	-8.444420026	1.44E-12	2.49E-10	18.28908369
ING2	0.556803288	3.252382284	8.432680647	1.52E-12	2.59E-10	18.25876894
RRP8	0.440247384	3.35251613	8.397944839	1.77E-12	2.96E-10	18.11201341
KCTD6	0.571690612	2.256614993	8.400447622	1.75E-12	2.96E-10	18.10791967
UCK2	0.564581408	5.05915692	8.390412723	1.83E-12	3.03E-10	18.01794768
GTF2B	0.368707816	4.085202935	8.351412188	2.17E-12	3.56E-10	17.87995215
PTGER4	-1.223032764	1.419273007	-8.33332983	2.35E-12	3.77E-10	17.8275412
PLEKHF1	0.66066562	5.026845982	8.337198734	2.31E-12	3.75E-10	17.78517274
EDRF1	0.656750905	2.960532807	8.298038186	2.75E-12	4.32E-10	17.68461423
PARP16	-0.456527194	2.694356881	-8.295561546	2.78E-12	4.32E-10	17.67348505
ATXN2L	-0.379414171	6.643587415	-8.300840241	2.72E-12	4.31E-10	17.60215063
SPRY1	-1.491943879	2.503585826	-8.267427938	3.15E-12	4.81E-10	17.54003756
ZUFSP	0.653210926	2.315413151	8.266918367	3.16E-12	4.81E-10	17.53503601
KIAA1614	-0.401549957	3.608454138	-8.220461549	3.88E-12	5.79E-10	17.33214575
ASAP3	-0.319444489	6.725121096	-8.222878606	3.84E-12	5.79E-10	17.26207946
DBF4	0.614080726	2.703949132	8.168258178	4.90E-12	7.16E-10	17.12523835
ZNF775	0.639398895	1.823019546	8.184075569	4.56E-12	6.74E-10	17.10042411
BORCS6	0.589694293	2.190199816	8.131032475	5.77E-12	8.29E-10	16.93859097
CTC1	-0.415532314	2.69420865	-8.124775192	5.94E-12	8.42E-10	16.93590684
SPSB1	0.778272139	5.790773619	8.143125716	5.47E-12	7.93E-10	16.91974406
SNAI2	0.583298412	6.800166099	8.123111504	5.98E-12	8.42E-10	16.83001212
SNPH	-0.602844611	3.916471216	-8.098866909	6.66E-12	9.20E-10	16.79873051
CAMTA2	-0.309657058	5.03568709	-8.099626847	6.64E-12	9.20E-10	16.74651493
SPRY4	-0.574653662	3.754660943	-8.068908451	7.61E-12	1.04E-09	16.63160988
ZNF34	0.580488801	1.528761665	8.056654803	8.03E-12	1.08E-09	16.53840314
IER5	0.529654043	6.031800192	8.056135496	8.05E-12	1.08E-09	16.5328736
SURF6	0.380709839	4.954363541	8.044467872	8.48E-12	1.13E-09	16.5184896
SHROOM3	-0.719098232	4.119030279	-8.017465485	9.55E-12	1.24E-09	16.40807635
SGK1	-0.743988556	6.400361427	-8.023936804	9.28E-12	1.23E-09	16.39450174
ZNF404	-0.723366054	1.283114933	-8.000667614	1.03E-11	1.32E-09	16.37898201
EHMT2	-0.382522423	5.895375297	-8.019533579	9.46E-12	1.24E-09	16.37796174
ZKSCAN4	0.636627589	2.107599583	7.977728553	1.14E-11	1.45E-09	16.26371506
KRCC1	-0.459121511	4.695084235	-7.971843283	1.17E-11	1.48E-09	16.20902027
BYSL	0.387398386	4.018675171	7.951753433	1.28E-11	1.60E-09	16.13710833
TBC1D10A	0.701996055	3.008107458	7.925592836	1.43E-11	1.79E-09	16.07527901
KCTD21	0.547905192	3.727474248	7.905921265	1.57E-11	1.92E-09	15.97168324
ORC2	-0.406729097	3.078329138	-7.900888991	1.60E-11	1.94E-09	15.96577854
CSRNP2	0.47148297	4.803697265	7.912021505	1.52E-11	1.88E-09	15.93884006
TNFSF9	0.745604281	2.0157642	7.832341005	2.17E-11	2.57E-09	15.68219614
KLHL25	0.688962451	1.659633474	7.83453047	2.15E-11	2.57E-09	15.63238629
ATG14	0.591836306	4.354382732	7.824308136	2.25E-11	2.64E-09	15.5996559
TOMM20	0.19298376	7.382595431	7.835973849	2.13E-11	2.57E-09	15.57930264
TSHZ1	-0.397572978	5.02322749	-7.779714114	2.73E-11	3.17E-09	15.36699833
RANBP10	-0.466891455	3.912218445	-7.744661079	3.19E-11	3.67E-09	15.24976002
ZNF792	0.926073434	0.668462933	7.794542158	2.56E-11	2.99E-09	15.09364981
USP16	0.303541371	5.647831324	7.721467017	3.54E-11	4.04E-09	15.08395263
SUPT20H	-0.384892757	3.794146112	-7.696882595	3.94E-11	4.47E-09	15.05659921
N4BP2L1	-0.548921253	2.49685235	-7.682152105	4.21E-11	4.70E-09	15.03796832
ZNF250	0.469411159	2.723961288	7.672086783	4.40E-11	4.84E-09	14.99574685
AHDC1	0.8607082	5.300008251	7.676557628	4.31E-11	4.78E-09	14.93025586
DNTTIP2	0.294127535	6.084594931	7.687819749	4.10E-11	4.61E-09	14.92939321
NOCT	-0.929379485	1.541791622	-7.650857901	4.83E-11	5.13E-09	14.90259411
TNFRSF10D	0.635108236	4.215595526	7.66386474	4.56E-11	4.94E-09	14.87295417

Gene	Log fold change	Average Expression	t	P-value	Adjusted P-value	B
GAB2	-0.413791761	4.954672794	-7.665163892	4.54E-11	4.94E-09	14.861663
ORC5	-0.412663672	3.347553121	-7.64346109	4.99E-11	5.26E-09	14.85731052
PHLDA1	-0.771506667	5.933007737	-7.660021131	4.64E-11	4.99E-09	14.81290517
NIP7	0.330690135	5.116719747	7.653619038	4.77E-11	5.10E-09	14.79894046
EMSY	-0.659252079	3.812486004	-7.621240659	5.51E-11	5.72E-09	14.7488981
ELL	0.419295476	4.299972796	7.63794128	5.11E-11	5.35E-09	14.74601068
MRPS31	-0.558519951	3.071299946	-7.591291705	6.28E-11	6.48E-09	14.62936019
ZNF48	0.628343677	2.688541279	7.568367718	6.95E-11	7.07E-09	14.55253305
TAF5	0.601304071	2.126702262	7.566530139	7.01E-11	7.08E-09	14.54244906
TNS2	-0.367877743	7.209638618	-7.584463404	6.47E-11	6.64E-09	14.48649807
SEMA4C	0.415107105	4.049055391	7.563123579	7.11E-11	7.14E-09	14.46723459
MPHOSPH10	0.446875857	5.150511353	7.556714116	7.32E-11	7.30E-09	14.38386805
GCH1	0.807579272	2.292021016	7.511209733	8.94E-11	8.76E-09	14.30959343
GAS1	-0.555798588	6.346857465	-7.521747777	8.54E-11	8.46E-09	14.210661
ZMYM3	-0.41994782	4.933911627	-7.510761425	8.96E-11	8.76E-09	14.18573988
PDE4B	-1.135846791	2.471099876	-7.480335002	1.02E-10	9.77E-09	14.17254463
RAB3A	0.861537553	1.450154373	7.491314131	9.76E-11	9.49E-09	14.17057584
RCOR3	-0.322702245	4.667935028	-7.488452017	9.88E-11	9.54E-09	14.11550231
BCL3	0.652055218	5.381712417	7.485753874	1.00E-10	9.60E-09	14.06646863
NFIC	-0.264137948	7.901152932	-7.474265449	1.05E-10	9.97E-09	14.01958892
RNF168	0.377755739	4.640752936	7.457943235	1.13E-10	1.06E-08	13.97878567
KCNE4	-0.728285686	2.743789532	-7.433809825	1.26E-10	1.17E-08	13.96977585
GTPBP4	0.595641153	5.73218001	7.461607112	1.11E-10	1.05E-08	13.95431088
ENO2	-0.431747466	4.872701056	-7.432763086	1.26E-10	1.17E-08	13.85429892
BTG3	0.401089582	4.032592312	7.410559356	1.39E-10	1.26E-08	13.8037941
KANSL2	0.430709435	3.845354325	7.406421825	1.42E-10	1.28E-08	13.80356491
PHF13	0.750917597	5.251495747	7.423821094	1.31E-10	1.21E-08	13.80062334
FILIP1L	-0.733497593	5.429270258	-7.411399139	1.39E-10	1.26E-08	13.73119594
CDC25B	-0.39679197	7.025777452	-7.403179044	1.44E-10	1.29E-08	13.69556821
USP19	-0.284617487	5.527758812	-7.40029875	1.46E-10	1.29E-08	13.69505451
RGS3	0.715625678	5.908003305	7.400074834	1.46E-10	1.29E-08	13.68319951
TRIM68	-0.541530255	2.875231273	-7.353365584	1.79E-10	1.56E-08	13.63872587
SLC3A2	-0.564680926	7.418706419	-7.3781512	1.61E-10	1.41E-08	13.60751186
RSAD1	-0.408364958	4.360944924	-7.364605268	1.70E-10	1.49E-08	13.57900249
ZNF615	0.497444261	2.913860868	7.338783076	1.91E-10	1.64E-08	13.57280354
LRCH1	-0.376719844	4.130630219	-7.342498456	1.88E-10	1.63E-08	13.51559272
PCF11	-0.487083213	4.194386172	-7.33516802	1.94E-10	1.66E-08	13.48165026
NFRKB	-0.392622268	3.892256458	-7.319134985	2.08E-10	1.76E-08	13.42258425
CDK9	0.247210181	5.209376899	7.323185192	2.04E-10	1.74E-08	13.37111598
TSC22D2	-0.594508339	4.592108337	-7.310205168	2.16E-10	1.82E-08	13.329786
WDCP	0.768297246	2.569546029	7.278818571	2.48E-10	2.07E-08	13.32494017
TBC1D13	-0.22847132	5.61479244	-7.303904583	2.22E-10	1.86E-08	13.2788402
SERPINB8	0.467623553	4.746140507	7.272355756	2.55E-10	2.11E-08	13.17543614
DCP2	0.548693024	3.506217014	7.243688692	2.90E-10	2.36E-08	13.14294379
ZNF654	0.50182243	3.798547372	7.243542677	2.90E-10	2.36E-08	13.10403886
ELMSAN1	-0.66520468	4.077134731	-7.248053493	2.84E-10	2.34E-08	13.09407113
USP21	-0.392190287	3.607283331	-7.234911652	3.01E-10	2.41E-08	13.08189717
PLK2	0.624873376	4.854332864	7.238148452	2.97E-10	2.39E-08	13.03074254
DENND2C	0.843870664	2.317854819	7.199476363	3.51E-10	2.73E-08	12.98718021
KLF2	-1.01333332	3.463363547	-7.212921435	3.31E-10	2.61E-08	12.95901899
RELB	0.502451185	4.513188695	7.21915625	3.22E-10	2.57E-08	12.95854597
ZNF563	0.784473698	0.77161294	7.242703529	2.91E-10	2.36E-08	12.94306664
E2F7	0.906016891	1.951261267	7.187650203	3.70E-10	2.84E-08	12.93230206
IER3IP1	0.26075161	4.980832651	7.218352206	3.24E-10	2.57E-08	12.93228977
CCDC174	0.271739617	4.016640839	7.205065219	3.43E-10	2.69E-08	12.92827034
PHF23	0.708592067	5.726053083	7.20142035	3.48E-10	2.72E-08	12.8407603
TRERF1	-0.710847184	4.789533086	-7.187776562	3.70E-10	2.84E-08	12.83758726
KAT14	-0.417861766	3.726469447	-7.172969382	3.95E-10	2.99E-08	12.82541499
TBK1	0.303994242	4.594493836	7.182760637	3.78E-10	2.89E-08	12.79039582
CDK7	0.365674606	4.160941339	7.176169926	3.89E-10	2.96E-08	12.77851261
JUNB	-0.923106851	6.337617529	-7.157705866	4.22E-10	3.18E-08	12.6406422
ZNF57	0.711473666	1.250619665	7.123594842	4.90E-10	3.65E-08	12.63841273
PEX11A	-0.532386055	1.944064876	-7.111313623	5.17E-10	3.83E-08	12.61787564
ZNF207	0.216783108	6.46216393	7.151791657	4.33E-10	3.24E-08	12.61129127
PPP2R5B	-0.341324099	4.13071913	-7.103622561	5.34E-10	3.93E-08	12.48088167
HAS2	-0.745596792	3.817740075	-7.100162292	5.42E-10	3.97E-08	12.47562303
FRAT2	0.637462823	2.39554511	7.077271072	5.99E-10	4.32E-08	12.47356602
ING1	0.459584418	3.884499285	7.085949265	5.77E-10	4.18E-08	12.41471732
SRSF3	0.251110488	7.14741694	7.10454037	5.32E-10	3.93E-08	12.41262436
CDCA4	0.32770387	3.00130011	7.066962159	6.27E-10	4.48E-08	12.39717936

Gene	Log fold change	Average Expression	t	P-value	Adjusted P-value	B
KIAA0753	-0.458828304	3.141176367	-7.062096907	6.41E-10	4.53E-08	12.38056376
WDR43	0.486089346	5.634297041	7.088304442	5.71E-10	4.16E-08	12.34347191
USP35	-0.515013004	2.676570771	-7.045750886	6.88E-10	4.78E-08	12.33639329
CSNK1G1	-0.577432976	4.141590378	-7.063246983	6.37E-10	4.53E-08	12.33591242
ZNF195	0.323897922	3.50271428	7.053754401	6.64E-10	4.65E-08	12.3044585
RXRB	-0.296682079	4.913670393	-7.06700597	6.27E-10	4.48E-08	12.28585536
ZFPM2	-0.641754373	2.845053828	-7.029200978	7.39E-10	5.02E-08	12.2528927
KIAA0907	-0.604014283	3.340576907	-7.029415643	7.39E-10	5.02E-08	12.23483229
MAP3K10	-0.514631891	2.729229699	-7.021047437	7.66E-10	5.18E-08	12.21640708
MAP2K3	0.561347242	6.536569544	7.058931548	6.49E-10	4.57E-08	12.21348246
SELENOS	0.367629556	5.3078796	7.047575064	6.82E-10	4.76E-08	12.18294082
NFKBIZ	-1.638933335	1.118680004	-7.007000953	8.14E-10	5.43E-08	12.17498097
NKRF	0.487086459	3.010242763	7.011502727	7.99E-10	5.35E-08	12.16076957
FHL2	0.347033643	7.000128448	7.04357947	6.94E-10	4.80E-08	12.15667204
ATN1	-0.304538547	7.903683782	-7.029547724	7.38E-10	5.02E-08	12.11371469
SBNO2	0.382545248	6.1177573	7.032831718	7.28E-10	5.01E-08	12.10110793
EGR3	-0.731794501	3.819849279	-7.015605797	7.84E-10	5.28E-08	12.03089962
OTUD1	-0.493350062	3.192914073	-6.977821721	9.25E-10	6.03E-08	11.99931046
CDC37L1	0.337661102	4.1200745	6.980242716	9.15E-10	6.02E-08	11.95749187
MORC2	-0.283357353	4.966363712	-6.980597339	9.14E-10	6.02E-08	11.91731116
CRY2	0.377295739	4.576926977	6.976918334	9.28E-10	6.03E-08	11.91075307
EDN1	-1.467065411	-0.460296738	-6.99606692	8.54E-10	5.67E-08	11.9031225
KLF9	0.445707628	4.966092688	6.979169917	9.19E-10	6.02E-08	11.89636801
ZNF667	0.699047896	3.156848324	6.934511172	1.12E-09	7.10E-08	11.85792119
ZFP64	-0.329466536	4.026706894	-6.952847026	1.03E-09	6.67E-08	11.85416328
USP27X	0.452449944	2.537054077	6.923025539	1.17E-09	7.43E-08	11.81227657
MOB4	0.31823962	4.220203624	6.942645047	1.08E-09	6.94E-08	11.7824371
MAU2	-0.266678486	4.89619244	-6.938389124	1.10E-09	7.04E-08	11.73552159
NFATC1	-0.406895153	3.954438508	-6.919028419	1.19E-09	7.53E-08	11.71935653
NIFK	0.337653807	5.308837163	6.935180608	1.11E-09	7.10E-08	11.70321616
DISP1	-0.546402976	2.757101556	-6.878887173	1.42E-09	8.88E-08	11.62598213
MAD2L1BP	0.326880683	4.026908892	6.878112083	1.43E-09	8.88E-08	11.52234076
TFB2M	0.323642919	3.598560964	6.855600998	1.57E-09	9.75E-08	11.45019148
MTCL1	-0.370288961	6.909153688	-6.879177055	1.42E-09	8.88E-08	11.44503609
MEX3A	0.865032788	1.797947981	6.830105728	1.76E-09	1.07E-07	11.441494
PARS2	0.497733367	2.515983215	6.82902987	1.77E-09	1.07E-07	11.43524338
C2CD2L	0.570453507	2.2345947	6.808386416	1.93E-09	1.15E-07	11.35378622
UAP1	0.443559073	6.85163187	6.847905861	1.63E-09	1.00E-07	11.31215107
FAM212A	0.77938665	2.452264684	6.792589142	2.07E-09	1.20E-07	11.27790655
ARC	1.139958727	0.284636044	6.798788408	2.01E-09	1.19E-07	11.26145721
ZNF324B	0.65352605	1.883066701	6.785008048	2.14E-09	1.23E-07	11.2507727
IRS1	-0.479309502	5.998819712	-6.832533166	1.74E-09	1.06E-07	11.24742404
ZNF584	0.37839947	3.594916629	6.802239854	1.98E-09	1.18E-07	11.24310772
ZNF230	0.600563318	1.889952638	6.781424086	2.17E-09	1.24E-07	11.23627153
ZBTB7B	0.376461018	4.437794707	6.809449197	1.92E-09	1.15E-07	11.2226922
HPS4	-0.254247654	4.425754514	-6.813217016	1.89E-09	1.14E-07	11.21657968
HSPA14	0.263809315	4.328006453	6.808609414	1.93E-09	1.15E-07	11.21391984
ZNF248	-0.528505766	2.50186373	-6.775537013	2.23E-09	1.25E-07	11.21250499
MAK16	0.420373467	3.875494573	6.795216854	2.04E-09	1.19E-07	11.18255925
THSD1	0.653998323	3.121218096	6.783996675	2.15E-09	1.23E-07	11.177533
FOSL2	-0.697008925	7.248480997	-6.810881736	1.91E-09	1.15E-07	11.17542009
EEPD1	-0.409821489	4.88099409	-6.797159008	2.03E-09	1.19E-07	11.15968878
RAB40C	-0.288223827	3.991638463	-6.780992022	2.17E-09	1.24E-07	11.13852777
TEAD4	0.484497603	3.580425489	6.776944612	2.21E-09	1.25E-07	11.12899407
PYGO2	-0.2318735	4.882586005	-6.792318945	2.07E-09	1.20E-07	11.1263357
LMCD1	0.695557894	6.67097804	6.798505463	2.01E-09	1.19E-07	11.10682809
COG8	-0.24907217	3.958078499	-6.776916546	2.21E-09	1.25E-07	11.10301986
PSTPIP2	0.494267174	2.24355641	6.750818509	2.48E-09	1.37E-07	11.10256082
PNRC2	0.30707952	5.262119797	6.791198359	2.08E-09	1.20E-07	11.09151862
SUFU	-0.397079024	4.925692768	-6.758039311	2.40E-09	1.34E-07	10.98820251
FAM117B	-0.544694558	2.356899243	-6.721482868	2.81E-09	1.53E-07	10.98612649
HYLS1	0.425202359	2.854120555	6.726677421	2.75E-09	1.50E-07	10.98069403
CASP3	0.34772592	4.970203619	6.759542155	2.38E-09	1.34E-07	10.97377739
CXCL8	-1.68008117	-1.419650404	-6.837863546	1.70E-09	1.04E-07	10.96593134
CCDC9	0.291172936	4.557667371	6.756193383	2.42E-09	1.34E-07	10.96499939
PPP1R3B	-0.409664659	4.911958339	-6.756071812	2.42E-09	1.34E-07	10.9611266
PDGFA	0.772210619	2.054681483	6.707223742	2.99E-09	1.60E-07	10.93377578
ZNF131	0.295069417	4.41857802	6.740589863	2.59E-09	1.42E-07	10.92338533
POLRMT	-0.325463211	4.041310195	-6.720714451	2.82E-09	1.53E-07	10.88371012
LACTB	0.276267332	4.600366481	6.732399112	2.68E-09	1.47E-07	10.86897215

Gene	Log fold change	Average Expression	t	P-value	Adjusted P-value	B
CBX4	0.328664361	3.612345311	6.713633071	2.91E-09	1.56E-07	10.8661724
TCHP	-0.325730986	3.852572961	-6.714618167	2.90E-09	1.56E-07	10.84517608
HUNK	-0.81325212	3.908125227	-6.707709186	2.98E-09	1.60E-07	10.84198217
C19orf44	-0.45438058	1.993211342	-6.675089641	3.43E-09	1.80E-07	10.80127605
SLC25A32	0.423203545	4.825436785	6.702741502	3.05E-09	1.62E-07	10.72246236
TUBB2A	0.315385797	4.516978986	6.689333553	3.23E-09	1.70E-07	10.7130083
EZH1	-0.296622141	4.500825299	-6.689250197	3.23E-09	1.70E-07	10.70752261
BAK1	0.497093598	3.210743888	6.659200553	3.68E-09	1.90E-07	10.68507581
DUSP14	0.456324913	6.059343135	6.69637524	3.13E-09	1.66E-07	10.66855028
FAM43A	0.64212213	4.466314543	6.680092035	3.36E-09	1.76E-07	10.65095448
DPH1	-0.275380814	4.589505294	-6.674451582	3.44E-09	1.80E-07	10.61715311
TBC1D31	-0.503826097	1.815630738	-6.627024522	4.23E-09	2.16E-07	10.60352349
ANAPC5	0.161771778	6.452380026	6.666498252	3.56E-09	1.85E-07	10.54158889
GLI2	-0.60520684	4.109991595	-6.630602152	4.16E-09	2.14E-07	10.52624976
NUMA1	-0.234181545	7.466547827	-6.635326066	4.08E-09	2.10E-07	10.42363283
PUM3	0.330103276	4.790576627	6.622985547	4.30E-09	2.20E-07	10.41557949
PAFAH2	-0.376074256	3.022976839	-6.591222901	4.93E-09	2.48E-07	10.38597871
DNAJC2	0.323501746	4.647989673	6.61250336	4.50E-09	2.29E-07	10.37860828
CLK2	0.692295468	3.206244649	6.570666258	5.38E-09	2.66E-07	10.32334077
RBBP6	-0.311850936	5.579448775	-6.609667508	4.55E-09	2.31E-07	10.30436566
NUDT12	-0.493271244	2.76159686	-6.562662919	5.57E-09	2.74E-07	10.30021285
ADAMTS1	-0.813988689	6.247424984	-6.595135048	4.85E-09	2.45E-07	10.24298897
AHRR	-0.409778474	5.529719084	-6.585293439	5.06E-09	2.54E-07	10.2246949
BRIX1	0.329954227	4.265850565	6.579102838	5.19E-09	2.58E-07	10.22100483
DLG4	-0.292069318	4.278473108	-6.576071391	5.26E-09	2.61E-07	10.21985329
PTP4A1	0.322556571	7.322408638	6.582139126	5.13E-09	2.56E-07	10.1960601
SOCS5	0.601903757	7.134241271	6.581656169	5.14E-09	2.56E-07	10.18694879
ERI2	-0.497363399	1.802953416	-6.526127173	6.52E-09	3.12E-07	10.17881355
FAM214A	-0.488155125	3.816023579	-6.550912881	5.86E-09	2.87E-07	10.15581841
C11orf84	0.385097402	3.922550741	6.545388991	6.00E-09	2.90E-07	10.1401672
KATNAL1	-0.392558928	4.26854648	-6.543791529	6.04E-09	2.91E-07	10.09754664
CLEC16A	-0.333138429	4.464108646	-6.547368401	5.95E-09	2.90E-07	10.08547213
HNRNPDL	0.256380134	7.306849616	6.556244953	5.73E-09	2.81E-07	10.07844259
NOP2	0.415076341	5.140181867	6.546515517	5.97E-09	2.90E-07	10.06895464
MSANTD2	0.429206735	2.584112078	6.503657146	7.18E-09	3.41E-07	10.06836562
RBM26	-0.226931744	4.777061123	-6.539902035	6.15E-09	2.95E-07	10.05842629
AIMP1	0.28643124	5.445630436	6.545997078	5.99E-09	2.90E-07	10.04742907
HSF2	0.374083912	3.404001352	6.49404011	7.48E-09	3.53E-07	9.96345706
HES1	0.66507725	4.343279933	6.502936674	7.20E-09	3.41E-07	9.884876897
RUSC2	-0.311624781	5.867802485	-6.502712237	7.21E-09	3.41E-07	9.852756304
GABPB1	0.263552891	4.623964466	6.487296886	7.70E-09	3.62E-07	9.841106471
ZNF16	-0.30452594	3.333962554	-6.456382648	8.79E-09	4.10E-07	9.799055077
MEX3B	0.66626231	2.657906876	6.440631196	9.40E-09	4.34E-07	9.794193164
USP30	-0.339144763	3.301901702	-6.449318089	9.06E-09	4.21E-07	9.78621475
FBXL20	-0.468013012	4.065768714	-6.460379334	8.64E-09	4.04E-07	9.775001033
LFNG	1.292800086	2.359853396	6.424995801	1.01E-08	4.60E-07	9.757330374
SIRT7	0.433745778	2.938815993	6.43398404	9.67E-09	4.46E-07	9.756909279
KDM4B	-0.310065918	5.396896254	-6.467488082	8.38E-09	3.93E-07	9.729866454
ZCCHC2	0.67539543	3.576307931	6.419170424	1.03E-08	4.69E-07	9.645069284
PPP1R15B	-0.428758483	4.861127733	-6.442418457	9.33E-09	4.33E-07	9.62421992
DMTF1	0.401588796	3.650856751	6.422276432	1.02E-08	4.64E-07	9.610314495
PRICKLE1	-0.632691375	3.080605501	-6.405811198	1.09E-08	4.95E-07	9.607275855
NSD3	-0.304267716	5.634840997	-6.431535733	9.78E-09	4.49E-07	9.563351838
NXT1	0.410493541	3.317768375	6.39247011	1.16E-08	5.21E-07	9.533337031
PTPDC1	-0.516264553	1.85219879	-6.363090936	1.31E-08	5.85E-07	9.51471722
CPEB1	-0.506791302	2.935544899	-6.369571173	1.27E-08	5.71E-07	9.506163658
RHOB	-0.466317121	5.586634923	-6.404274997	1.10E-08	4.97E-07	9.445759133
AHCTF1	0.433009922	4.034760832	6.370651164	1.27E-08	5.70E-07	9.397876972
PIM3	0.524069707	3.957166409	6.359987649	1.33E-08	5.90E-07	9.339424023
LRRC27	-0.301509129	3.472464359	-6.35018584	1.38E-08	6.10E-07	9.337881928
FOXC1	-0.5099667	3.785569714	-6.359910256	1.33E-08	5.90E-07	9.335403889
ZNF335	0.311114055	4.047217173	6.352902119	1.37E-08	6.06E-07	9.30817695
EIF1AD	0.296238608	4.363042681	6.350095083	1.38E-08	6.10E-07	9.283627022
ZNF548	-0.304727219	2.839872652	-6.313758231	1.62E-08	6.99E-07	9.249710587
UHRF2	-0.471197883	4.163104614	-6.328572903	1.52E-08	6.62E-07	9.245493323
MADD	-0.329493911	4.961093935	-6.339130258	1.45E-08	6.37E-07	9.220149657
PPP2R2A	0.259499932	4.876669053	6.327279729	1.53E-08	6.64E-07	9.1613901
FBXO45	0.443079412	3.83216195	6.309681678	1.64E-08	7.09E-07	9.151919417
BET1L	0.223003871	5.874189111	6.331254695	1.50E-08	6.57E-07	9.141792625
WHRN	-0.567977174	2.534640824	-6.285575805	1.82E-08	7.79E-07	9.139809453

Gene	Log fold change	Average Expression	t	P-value	Adjusted P-value	B
KCTD11	0.360976391	5.022761533	6.324175494	1.55E-08	6.71E-07	9.138506767
BANP	-0.401349498	2.642754272	-6.268066144	1.96E-08	8.32E-07	9.088820564
ZNF343	0.441013282	2.585851869	6.260464381	2.03E-08	8.48E-07	9.073411985
PER1	-0.379549532	3.927861469	-6.300731647	1.71E-08	7.35E-07	9.046116779
ZMYM6	0.377061199	2.695579169	6.261809721	2.01E-08	8.45E-07	9.043135062
CDKN2AIP	0.314639663	3.65047322	6.276923469	1.89E-08	8.04E-07	9.035048351
CXXC1	-0.323293503	4.920979352	-6.291465644	1.78E-08	7.62E-07	8.995733765
FBXW7	0.44264693	3.679572079	6.264774824	1.99E-08	8.39E-07	8.983700939
GRK2	-0.186997036	5.242496109	-6.282440221	1.85E-08	7.88E-07	8.959931537
PGBD1	-0.263642331	2.648871964	-6.229297646	2.31E-08	9.49E-07	8.933812548
PDCD7	0.251324083	4.735357925	6.265614598	1.98E-08	8.39E-07	8.913175403
LANCL2	0.327022542	4.704874196	6.262177631	2.01E-08	8.45E-07	8.89565898
COIL	0.26400843	3.932233374	6.246258639	2.15E-08	8.95E-07	8.879018359
NUAK2	-1.013101895	0.782516435	-6.201367601	2.60E-08	1.05E-06	8.869110319
ZFHX2	-0.844767234	0.566619232	-6.22533604	2.35E-08	9.63E-07	8.867342192
RGS12	-0.28408438	4.014798202	-6.239370258	2.22E-08	9.19E-07	8.845191483
PXN	-0.219942286	7.064224426	-6.259802262	2.03E-08	8.48E-07	8.838649562
CCDC120	-0.414277953	2.229933113	-6.198937593	2.63E-08	1.06E-06	8.834135604
KCTD1	-0.341725461	2.988684013	-6.205226486	2.56E-08	1.05E-06	8.810560805
KSR1	-0.589460579	3.080993261	-6.202430515	2.59E-08	1.05E-06	8.77751761
SH2B1	-0.274950182	4.913429194	-6.23680183	2.24E-08	9.27E-07	8.773317494
NDEL1	0.278149366	5.607532935	6.236218837	2.25E-08	9.27E-07	8.745451719
UBE3B	-0.213320537	5.304263561	-6.231479684	2.29E-08	9.43E-07	8.734428214
NRDE2	0.402359433	3.492533117	6.198683508	2.63E-08	1.06E-06	8.719101782
ZNF558	-0.275991946	3.259449283	-6.18626214	2.77E-08	1.11E-06	8.69551542
NDOR1	-0.279410423	3.036041894	-6.170166399	2.97E-08	1.18E-06	8.641799695
RPRD2	-0.270791913	5.607265704	-6.203471839	2.58E-08	1.05E-06	8.623935324
SMARCAL1	-0.224481899	4.950116576	-6.195991693	2.66E-08	1.07E-06	8.60682667
ATXN7L1	-0.602903391	2.932665118	-6.148956518	3.25E-08	1.27E-06	8.582663541
ALKBH4	0.354861468	3.186809218	6.150608191	3.23E-08	1.27E-06	8.571810598
RPAP2	-0.328922109	4.091635692	-6.167227014	3.01E-08	1.20E-06	8.550438228
INPP5E	-0.334842441	3.158091339	-6.143497723	3.32E-08	1.30E-06	8.541230107
TMEM200B	-0.258360731	4.009529533	-6.161371194	3.08E-08	1.22E-06	8.530151664
TOM1L2	-0.328128818	5.033688495	-6.173468716	2.93E-08	1.17E-06	8.503340432
KIAA1107	-0.923146861	1.165641879	-6.110304797	3.82E-08	1.48E-06	8.501190293
C7orf26	-0.284287499	4.501984284	-6.163904136	3.05E-08	1.21E-06	8.497696378
TBC1D22B	0.26262019	3.409827416	6.134729891	3.45E-08	1.34E-06	8.461773478
FOXL1	0.401074278	3.398806479	6.131839978	3.49E-08	1.35E-06	8.461603107
NCOR2	-0.227598606	8.212976303	-6.156917979	3.14E-08	1.24E-06	8.44367065
ZCCHC7	-0.277973985	3.980274554	-6.135207255	3.44E-08	1.34E-06	8.429310134
ATG101	0.279533855	4.779394231	6.132417236	3.48E-08	1.35E-06	8.354074363
STX3	0.373800535	3.958574505	6.099489446	4.00E-08	1.54E-06	8.268938866
TEX30	0.309982855	2.920081479	6.073764221	4.46E-08	1.70E-06	8.265962171
MED9	0.311235719	3.623477448	6.084961067	4.25E-08	1.63E-06	8.262062024
ZNF823	0.568597863	1.005880837	6.052736371	4.87E-08	1.83E-06	8.244423009
ZNF628	-0.465363669	3.163344876	-6.080078022	4.34E-08	1.66E-06	8.240119514
CENPJ	-0.521889002	1.91982366	-6.045711472	5.02E-08	1.87E-06	8.219047674
GNL2	0.353371838	6.088498473	6.105764785	3.90E-08	1.50E-06	8.203554429
BICRA	-0.352637499	3.558719552	-6.065841814	4.61E-08	1.75E-06	8.185758685
PLAUR	0.346483313	4.844504989	6.091033472	4.15E-08	1.59E-06	8.17263447
RCL1	0.28416367	2.936356001	6.049015023	4.95E-08	1.85E-06	8.14233559
ZNF22	-0.328622346	3.016641806	-6.048711727	4.95E-08	1.85E-06	8.124928409
SECISBP2	0.367404823	3.906961133	6.052990485	4.87E-08	1.83E-06	8.098400482
CCP110	-0.488761031	3.333904423	-6.033702624	5.28E-08	1.96E-06	8.085567413
ANKRD34A	0.824130034	1.725401346	5.998708331	6.11E-08	2.21E-06	8.058497433
RFXAP	-0.370502583	2.021121426	-6.009468108	5.84E-08	2.12E-06	8.051142395
NUPL2	-0.289339323	3.160990433	-6.03008569	5.36E-08	1.98E-06	8.048791609
H3F3B	0.251411475	7.944467256	6.056991142	4.78E-08	1.81E-06	8.034189137
NR2C1	-0.267997582	3.717051062	-6.028273984	5.40E-08	1.99E-06	8.01410662
CEBPD	-0.719277638	5.811572083	-6.059092948	4.74E-08	1.80E-06	8.012448331
TRMT1L	-0.3965664	3.999700328	-6.020722337	5.57E-08	2.04E-06	7.997720793
SETDB2	-0.379580778	4.11860496	-6.033785067	5.27E-08	1.96E-06	7.997022026
NOD1	0.438359319	2.621129408	5.993005288	6.26E-08	2.26E-06	7.941784523
USP20	-0.236330191	4.269455515	-6.019047996	5.61E-08	2.05E-06	7.929548415
HS6ST1	0.36552456	4.628138271	6.027493549	5.42E-08	1.99E-06	7.923501853
TUBB6	0.30692253	8.062682417	6.029192323	5.38E-08	1.99E-06	7.909421565
SUDS3	-0.234077222	4.456171382	-6.018716956	5.62E-08	2.05E-06	7.90856586
EN1	-0.415249775	4.135486645	-6.014282099	5.72E-08	2.08E-06	7.907184494
GFOD2	0.233395998	4.193608722	5.994851007	6.21E-08	2.24E-06	7.842762376
RELT	0.719277898	0.575787618	5.945524018	7.63E-08	2.69E-06	7.841881083

Gene	Log fold change	Average Expression	t	P-value	Adjusted P-value	B
OSBPL7	-0.510320716	1.54037571	-5.930903346	8.11E-08	2.86E-06	7.783701558
ZNF526	0.373650071	4.429586494	5.984035012	6.50E-08	2.34E-06	7.774862073
TCEANC2	-0.408922454	2.99316318	-5.94708791	7.58E-08	2.69E-06	7.772604981
ZNF618	-0.533252969	4.983832803	-5.970794441	6.87E-08	2.46E-06	7.694617614
ELMO2	-0.178711929	5.313864093	-5.96570437	7.02E-08	2.51E-06	7.652907029
SH3PXD2B	-0.457324693	8.009816068	-5.961714646	7.13E-08	2.55E-06	7.63042928
REEP4	-0.402931102	3.368425212	-5.91817623	8.56E-08	2.97E-06	7.625447488
FANCL	-0.424254356	1.968525052	-5.90095689	9.19E-08	3.16E-06	7.623062755
SMAP2	-0.246474342	4.984677174	-5.95575544	7.31E-08	2.60E-06	7.621406491
RIOX1	0.281885698	3.384309151	5.926431237	8.27E-08	2.89E-06	7.621328745
FGFR1OP2	-0.224999503	4.627467101	-5.952463168	7.42E-08	2.63E-06	7.615629258
TNFAIP3	-0.730747056	2.552029645	-5.902500075	9.14E-08	3.14E-06	7.613381997
ZNF318	-0.403864337	5.026761088	-5.946439507	7.60E-08	2.69E-06	7.591716953
ANKRD26	-0.408568961	3.005540625	-5.897741249	9.32E-08	3.19E-06	7.543751342
DLX3	1.089160926	0.578335009	5.868983915	1.05E-07	3.56E-06	7.534854927
KDM4A	-0.270556855	5.247918199	-5.926787801	8.26E-08	2.89E-06	7.496570299
ZBTB17	0.324389464	4.323365836	5.914421845	8.69E-08	3.01E-06	7.492892858
ZNF274	-0.420486741	2.771523423	-5.880855648	1.00E-07	3.40E-06	7.478705098
CSTF2T	-0.242598185	5.57503419	-5.925149353	8.31E-08	2.90E-06	7.475176316
ARHGAP12	-0.367175404	5.939040469	-5.928203993	8.21E-08	2.88E-06	7.474908304
ATF7IP	-0.42964193	5.766297775	-5.922491472	8.40E-08	2.92E-06	7.46021199
PPP2CA	0.187203126	6.51490808	5.913399727	8.73E-08	3.02E-06	7.410442583
TRPS1	-0.535096654	5.598755458	-5.904728613	9.05E-08	3.12E-06	7.395892471
KIAA0355	-0.516550282	4.217981293	-5.880903041	1.00E-07	3.40E-06	7.350422083
ZSCAN22	0.430255755	2.004884147	5.818182847	1.30E-07	4.34E-06	7.314485559
RHOQ	0.132155576	6.61766176	5.886686972	9.76E-08	3.33E-06	7.301768814
DGCR11	0.502737329	0.903559522	5.806791089	1.36E-07	4.49E-06	7.294597082
GATA2	0.626335996	3.218539868	5.8342394	1.21E-07	4.09E-06	7.252680556
ZNF438	-0.369433172	2.952847858	-5.807012355	1.36E-07	4.49E-06	7.170495059
ZNF616	0.518444065	3.501483294	5.802818443	1.38E-07	4.56E-06	7.166020338
MTPAP	0.317492104	3.844427183	5.817228242	1.30E-07	4.34E-06	7.140244166
CREBZF	0.546057853	3.287571038	5.799570433	1.40E-07	4.61E-06	7.137305235
RBM47	-0.796092405	1.076591594	-5.758568376	1.66E-07	5.33E-06	7.111646305
IER5L	0.510493448	5.5983292	5.836816048	1.20E-07	4.06E-06	7.103649807
ZNF630	-0.488690232	0.455592206	-5.755897282	1.68E-07	5.38E-06	7.096890017
TARS2	-0.372910596	3.502339906	-5.799000567	1.40E-07	4.61E-06	7.083468986
SNX33	-0.177770116	6.669118481	-5.828408635	1.24E-07	4.18E-06	7.065590264
PANK1	0.664605987	2.497608233	5.752173694	1.71E-07	5.45E-06	7.063474259
MLLT10	-0.477857368	3.506491821	-5.781766424	1.51E-07	4.93E-06	7.061821546
ETF1	0.261856263	7.215437237	5.825182419	1.26E-07	4.23E-06	7.056853028
CDYL	-0.237649251	4.688611142	-5.808374567	1.35E-07	4.48E-06	7.039761566
CDAN1	-0.307448076	3.340838434	-5.772686622	1.57E-07	5.07E-06	7.032714244
BCL6	-0.512119289	5.938922774	-5.81753641	1.30E-07	4.34E-06	7.022388936
PPIL2	-0.204911951	4.938324037	-5.808580775	1.35E-07	4.48E-06	7.021929731
MAFK	0.340281374	3.740487341	5.782036234	1.51E-07	4.93E-06	6.973056424
ZC3H4	-0.287133643	4.74804751	-5.77868339	1.53E-07	4.98E-06	6.924357708
FAM60A	0.861402526	0.758019895	5.709706072	2.03E-07	6.34E-06	6.915650613
ZNF24	-0.322209008	5.368994903	-5.776757885	1.54E-07	5.01E-06	6.874299677
SLC12A4	-0.186255964	6.265771077	-5.774860023	1.55E-07	5.04E-06	6.849368445
MED12	-0.309560664	5.456873407	-5.767313187	1.60E-07	5.17E-06	6.834273891
UBTD2	0.219727446	6.235896702	5.767495005	1.60E-07	5.17E-06	6.82167928
WTAP	0.249201304	6.414560728	5.763170369	1.63E-07	5.24E-06	6.802019923
ZKSCAN2	-0.491079865	3.02263463	-5.715058907	1.99E-07	6.22E-06	6.800921654
E2F6	-0.296436875	2.67381161	-5.71456876	1.99E-07	6.22E-06	6.798289505
FZD2	-0.353173735	4.40415785	-5.731131826	1.86E-07	5.90E-06	6.767151915
SERPINB9	0.603585944	1.529848247	5.672318697	2.37E-07	7.18E-06	6.762286854
TRAFD1	-0.188787816	4.982243166	-5.739585502	1.80E-07	5.73E-06	6.746365232
ZNF516	-0.352226814	3.906706921	-5.720731321	1.94E-07	6.11E-06	6.746140838
MRM3	0.280837648	2.800009944	5.69374109	2.17E-07	6.71E-06	6.745057913
DEPDC7	0.505915658	1.905784148	5.677010456	2.32E-07	7.07E-06	6.744330636
CYCS	0.234992717	5.255552236	5.733726104	1.84E-07	5.86E-06	6.716115665
RGS4	-0.628038207	4.915745894	-5.733161815	1.84E-07	5.86E-06	6.710642774
IL6R	0.597689073	3.176895308	5.691544967	2.19E-07	6.75E-06	6.707208216
SLC35G2	0.297291676	3.103047295	5.692475776	2.18E-07	6.73E-06	6.706472332
ZNF114	1.506941501	-1.686357682	5.840385802	1.18E-07	4.00E-06	6.674783893
PAPD7	0.470727475	4.09578995	5.702987073	2.09E-07	6.49E-06	6.669302803
AMBRA1	-0.269048991	5.154805277	-5.71932954	1.95E-07	6.13E-06	6.664120809
SMURF2	0.429857602	5.204137854	5.722622216	1.93E-07	6.08E-06	6.652972136
HDGFRP3	0.163809805	5.629198588	5.720867836	1.94E-07	6.11E-06	6.647103641
MTM1	-0.34641657	2.869952761	-5.672338128	2.37E-07	7.18E-06	6.643252056

Gene	Log fold change	Average Expression	t	P-value	Adjusted P-value	B
UBE2G2	0.172632546	6.180514168	5.72317482	1.92E-07	6.08E-06	6.642760599
RFX1	-0.324184191	3.721508768	-5.679470188	2.30E-07	7.02E-06	6.633765595
GPBP1L1	-0.227972473	5.273572114	-5.706805368	2.06E-07	6.40E-06	6.59990904
C14orf79	-0.447642823	2.453702151	-5.648363043	2.62E-07	7.86E-06	6.585966855
ABHD6	-0.380624128	2.249233049	-5.637064378	2.74E-07	8.20E-06	6.576767954
LRRC59	0.182695357	7.573385441	5.694873673	2.16E-07	6.69E-06	6.536954486
POLD3	-0.314308107	3.910892564	-5.66574609	2.43E-07	7.35E-06	6.533054859
MYSM1	0.737914675	2.533777879	5.615912475	2.99E-07	8.82E-06	6.502662198
SLC20A1	0.708127608	7.015763557	5.68616883	2.24E-07	6.88E-06	6.501057865
PSMD6	0.213777301	5.801221867	5.683152927	2.27E-07	6.95E-06	6.490883529
PPIF	0.422892023	5.894195507	5.682641048	2.27E-07	6.95E-06	6.481669504
KLF4	0.257231615	6.567953487	5.681902103	2.28E-07	6.96E-06	6.475082294
ZNF26	0.364064009	2.644310353	5.617000536	2.97E-07	8.80E-06	6.445448915
NKX3-1	0.481710748	1.984296258	5.594667226	3.26E-07	9.46E-06	6.438144503
INPPL1	-0.219896613	6.102900878	-5.670163352	2.39E-07	7.23E-06	6.428883966
TXNDC9	0.205439333	4.550973891	5.652136523	2.57E-07	7.76E-06	6.427315798
MLLT3	-0.429956197	3.68347239	-5.631836691	2.80E-07	8.35E-06	6.40627204
ZNF71	0.357316809	2.979323064	5.607083754	3.10E-07	9.09E-06	6.401516963
ULK3	0.325022197	3.063418268	5.603619563	3.14E-07	9.17E-06	6.386187144
SIX1	0.606568189	3.822617765	5.62083034	2.93E-07	8.68E-06	6.383791725
VIPAS39	-0.319845367	4.662199395	-5.636612982	2.74E-07	8.20E-06	6.362767706
RMDN2	0.291900072	3.372427097	5.608453074	3.08E-07	9.08E-06	6.360746603
ZNF518A	-0.580114823	2.499165623	-5.582568745	3.42E-07	9.86E-06	6.340974868
TCF7L2	-0.357569917	5.198042049	-5.641669662	2.69E-07	8.07E-06	6.336447803
DOT1L	0.460325356	4.476610279	5.624830096	2.88E-07	8.57E-06	6.308836185
MAST3	-0.400816107	2.696501265	-5.558757258	3.77E-07	1.07E-05	6.25623737
GDI1	-0.186581045	6.299324926	-5.62102425	2.93E-07	8.68E-06	6.231057772
SLC26A11	-0.310634797	3.368765225	-5.585025324	3.39E-07	9.78E-06	6.227413458
MINDY1	-0.260464948	3.508709087	-5.586091061	3.38E-07	9.76E-06	6.220546114
SERTAD2	0.396219325	7.328783091	5.606808403	3.10E-07	9.09E-06	6.192800063
DUSP1	-0.721846087	5.323536731	-5.606676211	3.10E-07	9.09E-06	6.185063359
PRDM2	-0.465475162	5.27253688	-5.602116039	3.16E-07	9.21E-06	6.176007449
ZFP36	-0.700723221	6.422503888	-5.604941161	3.12E-07	9.14E-06	6.170290896
DUSP2	-0.880493726	-0.26838837	-5.513732941	4.53E-07	1.27E-05	6.167583087
TBX5	0.631876371	4.166187214	5.565746618	3.67E-07	1.05E-05	6.157835114
EIF5	0.403840317	7.200811647	5.600320973	3.18E-07	9.26E-06	6.152633393
RHOT1	-0.246985331	4.183217683	-5.571876045	3.58E-07	1.03E-05	6.126211646
SFSWAP	0.303124541	4.597467121	5.572516182	3.57E-07	1.03E-05	6.125859484
FAS	0.209381052	5.41049368	5.58737645	3.36E-07	9.73E-06	6.111488162
RRN3	0.391354532	4.325944307	5.568527296	3.63E-07	1.04E-05	6.108467087
YEATS2	-0.420885586	4.362742991	-5.54265505	4.03E-07	1.15E-05	6.042081858
TBX2	0.467933737	3.538872063	5.53101497	4.23E-07	1.20E-05	6.028036089
SUB1	0.134271199	7.05204874	5.568715175	3.62E-07	1.04E-05	6.025082129
HINFP	0.361370495	2.570932666	5.489838113	5.00E-07	1.39E-05	5.989590008
METTL1	0.275908096	3.599979171	5.516185263	4.49E-07	1.26E-05	5.971334203
GPATCH2	0.375856246	3.261007123	5.503808776	4.72E-07	1.32E-05	5.940229033
TCF20	-0.329788786	5.287808616	-5.538810196	4.09E-07	1.16E-05	5.940136196
RNF34	-0.219860439	4.164322973	-5.512684377	4.55E-07	1.28E-05	5.920386214
WBP4	0.207077718	3.816927692	5.517041694	4.47E-07	1.26E-05	5.919375198
LINS1	0.448968066	1.947173532	5.447781031	5.93E-07	1.61E-05	5.87346747
SOX8	1.297042623	-0.510047906	5.461131343	5.62E-07	1.53E-05	5.87043448
THRA	-0.173582431	6.263442811	-5.527817823	4.28E-07	1.21E-05	5.861991683
GAN	0.369617378	2.961718492	5.473349706	5.34E-07	1.47E-05	5.855237426
PLD6	-0.428305193	0.803772793	-5.435216442	6.24E-07	1.68E-05	5.838394362
CCDC117	0.265667717	4.966505348	5.506935077	4.66E-07	1.30E-05	5.834930912
RNF216P1	0.244313965	3.292139692	5.475793638	5.29E-07	1.46E-05	5.830826868
BLOC1S4	0.248166587	3.532735878	5.481693657	5.17E-07	1.43E-05	5.822244539
ARHGAP32	-0.391365835	4.382400406	-5.491350809	4.97E-07	1.38E-05	5.798281738
FAM46A	-0.511526196	6.475562028	-5.509697939	4.61E-07	1.29E-05	5.789664326
DLC1	-0.560649953	6.82414642	-5.503133208	4.73E-07	1.32E-05	5.7876375
PIP5K1A	0.415559172	5.240849492	5.485690093	5.08E-07	1.41E-05	5.706830426
MIR22HG	0.392828199	4.512902585	5.472776971	5.36E-07	1.47E-05	5.691157617
CHD1	0.507828735	4.706783403	5.468597289	5.45E-07	1.49E-05	5.671611339
ZNF624	0.495426941	2.053607115	5.390611524	7.47E-07	1.98E-05	5.667981908
ZNF512	-0.256852356	3.549047362	-5.437193455	6.19E-07	1.67E-05	5.657693396
HMOX1	0.56407324	9.028612763	5.462481681	5.58E-07	1.53E-05	5.656255237
TMUB2	-0.17842168	5.206427672	-5.462638203	5.58E-07	1.53E-05	5.624306438
CEP104	-0.264353277	4.299529609	-5.441578875	6.08E-07	1.65E-05	5.607663872
ZNF442	0.920243874	-0.303613108	5.397689006	7.26E-07	1.94E-05	5.588786596
FLYWCH1	-0.324237787	5.64940981	-5.44501673	5.99E-07	1.63E-05	5.542617455

Gene	Log fold change	Average Expression	t	P-value	Adjusted P-value	B
ULK1	-0.251041086	5.493694662	-5.443597133	6.03E-07	1.64E-05	5.540006887
RWDD2A	0.334176199	2.96371086	5.389446018	7.50E-07	1.98E-05	5.534900881
ANKRD46	0.458458882	2.306768314	5.356035293	8.59E-07	2.25E-05	5.511055822
CEP152	-0.720902485	0.430563388	-5.341175103	9.11E-07	2.36E-05	5.502021578
PPP4R2	0.272418297	5.013655155	5.427170701	6.44E-07	1.74E-05	5.500291712
CCDC51	0.346468018	3.648749669	5.395150928	7.33E-07	1.95E-05	5.489937476
POLR3E	-0.214266335	4.343546975	-5.40382846	7.08E-07	1.90E-05	5.463408785
UTP11	0.258038012	5.252457424	5.419973307	6.63E-07	1.78E-05	5.461743781
ASTE1	0.406853177	2.723857705	5.353112346	8.69E-07	2.27E-05	5.450092838
TRIM47	0.484773215	4.790896977	5.402720384	7.11E-07	1.90E-05	5.437232763
HOMEZ	0.442324601	3.145431146	5.346769484	8.91E-07	2.32E-05	5.419993167
STXBP1	-0.194900549	5.733201176	-5.409986504	6.91E-07	1.85E-05	5.405729125
PAIP1	0.177329846	4.803875652	5.395932508	7.31E-07	1.95E-05	5.391395338
KAT6B	-0.331289857	4.998370542	-5.395112329	7.33E-07	1.95E-05	5.377717539
GORASP2	0.144720406	7.336600742	5.393144877	7.39E-07	1.96E-05	5.336038387
SYNRG	-0.420971291	4.89074306	-5.378427232	7.84E-07	2.07E-05	5.334259136
CNOT10	-0.180758154	4.394555074	-5.374899966	7.96E-07	2.09E-05	5.332655148
GTF2E1	0.399560603	2.726426225	5.314463651	1.01E-06	2.60E-05	5.310335699
TMEM206	-0.357487803	1.768168692	-5.296477834	1.09E-06	2.77E-05	5.3075952
IRX2	0.392159412	3.976060363	5.35659354	8.57E-07	2.25E-05	5.294476288
STX6	0.216721407	5.570593247	5.374967828	7.96E-07	2.09E-05	5.272031337
AXIN2	-0.411176828	3.655024932	-5.339401898	9.18E-07	2.37E-05	5.259729927
SHOX2	-0.340799575	3.978008705	-5.344132796	9.01E-07	2.34E-05	5.250133715
RCOR2	-0.538157899	1.695706982	-5.270410659	1.21E-06	3.04E-05	5.245253047
KMT5C	0.756000244	0.721125176	5.274663923	1.19E-06	3.00E-05	5.240457726
F3	-0.868383083	2.902619591	-5.339831955	9.16E-07	2.37E-05	5.225497328
DDX20	0.253294439	4.389039194	5.345397792	8.96E-07	2.33E-05	5.215395973
CD274	-0.793480867	-0.227743443	-5.262287739	1.25E-06	3.10E-05	5.211587782
CHTF8	-0.275796485	5.575713826	-5.354862046	8.63E-07	2.26E-05	5.196809017
TTC33	-0.424953603	2.747521737	-5.279874663	1.17E-06	2.95E-05	5.173102814
PRIM1	-0.404641281	1.735738425	-5.264120718	1.24E-06	3.08E-05	5.164462456
PQLC2	0.30047963	4.348662282	5.324969289	9.73E-07	2.50E-05	5.158647628
TIAM2	-0.47939323	2.192357253	-5.26185016	1.25E-06	3.10E-05	5.123277535
TTC31	-0.262619403	3.697092878	-5.308739272	1.04E-06	2.66E-05	5.119939855
PHRF1	0.20974471	5.575125458	5.333367623	9.40E-07	2.43E-05	5.109617672
RBM5	-0.353932016	4.582961735	-5.324657834	9.74E-07	2.50E-05	5.109305481
SLC19A2	0.447830546	2.550570471	5.275763792	1.18E-06	2.99E-05	5.083958168
GPSM1	-0.224180876	5.830682005	-5.324171193	9.76E-07	2.50E-05	5.068379468
MTHFSD	-0.279416507	3.04363448	-5.259719219	1.26E-06	3.12E-05	5.035849997
ZFP41	-0.293675967	2.720366862	-5.254021993	1.29E-06	3.19E-05	5.034473556
PPHLN1	0.167194829	5.102630819	5.307184749	1.04E-06	2.67E-05	5.032315085
TRMT44	-0.301202493	2.18657261	-5.22728291	1.44E-06	3.49E-05	5.014011195
EED	0.228389146	3.678165261	5.271790688	1.20E-06	3.03E-05	5.012421568
PMS2	-0.410238272	2.802871586	-5.242047731	1.36E-06	3.31E-05	4.997606273
SIX4	0.472558484	3.898773199	5.269551955	1.21E-06	3.05E-05	4.989172703
CALHM2	-0.212258724	6.17092771	-5.297354989	1.09E-06	2.77E-05	4.958641297
FAM53B	-0.199439255	4.256243005	-5.268458846	1.22E-06	3.05E-05	4.957474415
CBFA2T2	-0.378017356	4.134847051	-5.265029216	1.24E-06	3.08E-05	4.95652246
ZCCHC4	0.434295029	2.225882084	5.209132677	1.55E-06	3.73E-05	4.945077281
MAVS	-0.325317099	6.665097431	-5.293241547	1.10E-06	2.81E-05	4.93899269
TRIAP1	0.222058977	4.994139952	5.283175733	1.15E-06	2.92E-05	4.937101278
FLRT3	-1.073647451	1.155685431	-5.185487651	1.70E-06	4.07E-05	4.916941696
AUTS2	-0.37366907	5.056171247	-5.269145102	1.22E-06	3.05E-05	4.902349377
DPM1	0.270795532	4.725993392	5.264830923	1.24E-06	3.08E-05	4.895828595
NET1	0.355157046	3.897992017	5.248279883	1.32E-06	3.25E-05	4.894229033
PPP3CC	-0.215389284	3.681963541	-5.242501832	1.35E-06	3.31E-05	4.885000354
MAP2K5	-0.198622719	4.7442092	-5.265309148	1.24E-06	3.08E-05	4.871775715
CBLB	0.484623581	4.995698016	5.267719481	1.22E-06	3.06E-05	4.870031215
PCTP	0.269891596	2.682131371	5.191570789	1.66E-06	3.98E-05	4.849978295
ETV3	0.26744528	4.496259908	5.247879702	1.32E-06	3.25E-05	4.821539511
TMEM39A	0.301330001	5.1887841	5.249889657	1.31E-06	3.23E-05	4.799159127
ECHDC1	0.117012781	5.840165235	5.245808827	1.34E-06	3.27E-05	4.763996928
ZFP62	0.322013448	3.301138805	5.186631812	1.69E-06	4.05E-05	4.75671036
UBE4B	-0.183249909	6.423532279	-5.238300801	1.38E-06	3.35E-05	4.725215088
SOS1	-0.373561148	6.210473769	-5.235680124	1.39E-06	3.38E-05	4.716508681
RARG	-0.196086546	7.413476339	-5.233541854	1.40E-06	3.41E-05	4.715500375
PNO1	0.252388054	4.119826183	5.208110986	1.55E-06	3.74E-05	4.710545209
PNPLA8	0.254354027	5.183917698	5.220301892	1.48E-06	3.58E-05	4.693038565
SWT1	-0.38105595	2.268514656	-5.140365515	2.03E-06	4.73E-05	4.676867396
EFS	-0.242008153	5.314929253	-5.217497439	1.49E-06	3.61E-05	4.671161952

Gene	Log fold change	Average Expression	t	P-value	Adjusted P-value	B
WDR89	0.34773037	2.797425055	5.14591519	1.99E-06	4.65E-05	4.632719945
ZNF480	-0.269660207	3.823526492	-5.173486705	1.78E-06	4.25E-05	4.609508617
ZNF10	0.412142109	1.287591707	5.103843193	2.35E-06	5.34E-05	4.608627729
ALG2	0.136152249	5.933632885	5.205010479	1.57E-06	3.78E-05	4.602903995
BBS12	0.344601355	2.684669683	5.124537794	2.16E-06	4.97E-05	4.599849601
CXCL2	-1.638130104	-1.964615805	-5.162246621	1.86E-06	4.41E-05	4.587474938
CARD6	-0.292567068	4.86330035	-5.17154494	1.79E-06	4.27E-05	4.52664858
COQ8B	-0.219748251	3.701211442	-5.146813839	1.98E-06	4.64E-05	4.501706578
PHF12	0.274831743	4.858206189	5.157758185	1.90E-06	4.48E-05	4.498967195
ZSCAN2	0.323413761	1.922694579	5.08423992	2.53E-06	5.68E-05	4.498714348
DUSP3	0.195023088	6.272464369	5.176151768	1.76E-06	4.21E-05	4.48586099
TAX1BP1	0.110922789	7.188616512	5.172232014	1.79E-06	4.27E-05	4.476697047
UBA3	0.15501904	5.229684686	5.162822394	1.86E-06	4.41E-05	4.460186774
TXNIP	0.508807579	7.24926452	5.16679351	1.83E-06	4.35E-05	4.452150833
SPICE1	0.368050106	2.016897998	5.08496402	2.53E-06	5.67E-05	4.451544948
ACTRT3	-0.330302338	1.971308034	-5.081028856	2.57E-06	5.74E-05	4.434386178
CRTC1	-0.23670643	4.795920447	-5.147638931	1.97E-06	4.64E-05	4.422356652
CEP95	0.290115804	3.670280348	5.124892888	2.16E-06	4.97E-05	4.419621984
PPFIBP2	-0.275122429	4.100342424	-5.131480803	2.10E-06	4.86E-05	4.41299936
FAM193B	0.461595045	3.265370847	5.10039305	2.38E-06	5.39E-05	4.404655927
SNAPC4	-0.225753158	3.720390271	-5.106751364	2.32E-06	5.28E-05	4.399156888
VAPA	0.124849718	6.910609197	5.151454495	1.94E-06	4.59E-05	4.393059312
CNOT2	0.177464482	5.741944057	5.149731384	1.96E-06	4.61E-05	4.393044392
ELK1	0.175860724	5.884369928	5.147462284	1.97E-06	4.64E-05	4.382815028
OSBPL2	-0.167127154	4.771924155	-5.134906903	2.07E-06	4.82E-05	4.3815716
PNN	0.380377216	5.141509249	5.136440884	2.06E-06	4.79E-05	4.3734952
FAM102A	-0.18477412	6.609379449	-5.145951739	1.99E-06	4.65E-05	4.369283849
CHAC1	-1.597327079	1.1046177	-5.038229566	3.04E-06	6.62E-05	4.367980519
DDX23	-0.150596412	6.095195324	-5.142688595	2.01E-06	4.69E-05	4.360258079
XPO7	-0.182545741	6.188878674	-5.142598982	2.01E-06	4.69E-05	4.357293951
AEBP2	0.242824248	4.326962876	5.123273678	2.17E-06	4.99E-05	4.353015828
CPEB4	-0.448941919	3.64603606	-5.094683051	2.43E-06	5.50E-05	4.340687659
PSPC1	0.24739015	4.557363479	5.114411394	2.25E-06	5.14E-05	4.335601979
TRMO	0.305296739	3.071209632	5.085176733	2.52E-06	5.67E-05	4.330619327
RSL1D1	0.161204714	7.906585487	5.128194766	2.13E-06	4.92E-05	4.32789282
TULP3	-0.154325365	5.728374342	-5.131682453	2.10E-06	4.86E-05	4.323740743
EPHB4	-0.202141181	6.292327357	-5.132027908	2.10E-06	4.86E-05	4.317617699
KLHDC2	0.148996152	4.782172718	5.120775472	2.19E-06	5.03E-05	4.317520901
AGPAT4	-0.326546475	4.109336602	-5.099828667	2.38E-06	5.40E-05	4.31190311
AASDH	-0.428455663	2.623290751	-5.054934482	2.84E-06	6.28E-05	4.305304307
UFM1	0.201152992	6.118725012	5.117282565	2.22E-06	5.09E-05	4.264653977
MPHOSPH8	-0.335023751	5.147377378	-5.107391045	2.31E-06	5.28E-05	4.253836467
ZMYND8	-0.297367273	4.160123733	-5.086329423	2.51E-06	5.66E-05	4.251776792
METTL13	-0.200575369	5.27074638	-5.102396811	2.36E-06	5.36E-05	4.236133273
ZCCHC10	0.30995295	3.085304856	5.052233736	2.87E-06	6.34E-05	4.23578228
MSX2	0.62819331	2.439421403	5.029855772	3.14E-06	6.81E-05	4.213690014
ANKRA2	-0.332913405	2.486144544	-5.041782359	2.99E-06	6.55E-05	4.209558565
MIER2	0.273257354	4.209717532	5.078013282	2.60E-06	5.80E-05	4.206950324
TSC22D4	-0.29441929	5.511434944	-5.094172095	2.44E-06	5.50E-05	4.19775739
PLEKHF2	0.375216859	3.218555619	5.04245778	2.99E-06	6.54E-05	4.181764974
TTBK2	-0.394681417	3.080830876	-5.045293095	2.95E-06	6.48E-05	4.180366372
PCNA	0.233691253	5.947349807	5.091911399	2.46E-06	5.54E-05	4.172298835
CDCA8	-0.569231183	1.85808182	-4.988634183	3.69E-06	7.82E-05	4.157287392
HMGCL	-0.257486868	4.744770578	-5.068586119	2.70E-06	6.01E-05	4.121841516
FAM161B	-0.239543724	2.68457292	-5.006575732	3.44E-06	7.39E-05	4.108847406
TECPR1	-0.206035222	4.130324621	-5.049232707	2.91E-06	6.40E-05	4.095814561
ZNF100	-0.569707142	1.680822552	-4.969285326	3.98E-06	8.34E-05	4.087859263
PLCG1	-0.174585427	5.692114975	-5.067781031	2.70E-06	6.03E-05	4.081572785
TIMM17A	0.214991038	5.23301039	5.058339124	2.81E-06	6.22E-05	4.072771818
PIP5K1C	-0.179284638	7.316712773	-5.064537388	2.74E-06	6.08E-05	4.066823156
BNIP2	0.248747866	5.977886095	5.065570644	2.73E-06	6.07E-05	4.065969987
SMARCD2	-0.136998886	5.813709675	-5.060579675	2.78E-06	6.17E-05	4.053182562
SEH1L	0.291663725	5.243595622	5.051186615	2.89E-06	6.36E-05	4.051128576
SYNJ1	-0.467408882	3.098938354	-4.995800395	3.59E-06	7.64E-05	4.04740586
BRSK1	-0.556292641	0.551035531	-4.940220227	4.45E-06	9.25E-05	4.038652713
PHC2	0.234423732	6.700155936	5.057452208	2.82E-06	6.23E-05	4.033428749
DBP	-0.363892961	2.99976443	-4.99618371	3.58E-06	7.64E-05	4.029133754
RPUSD2	0.252448845	3.381368965	5.00292013	3.49E-06	7.47E-05	4.010359042
ZCCHC3	0.201722441	4.431519196	5.029882847	3.14E-06	6.81E-05	4.002238192
DDAH1	0.22967834	7.17706387	5.045637191	2.95E-06	6.48E-05	3.987309095

Gene	Log fold change	Average Expression	t	P-value	Adjusted P-value	B
C17orf80	-0.309727859	3.378771202	-4.988517758	3.69E-06	7.82E-05	3.985312783
ZSWIM4	-0.321319583	2.884271624	-5.002371448	3.49E-06	7.47E-05	3.980029991
RNF115	0.158469084	6.04159249	5.040994088	3.00E-06	6.56E-05	3.972280741
COPS4	0.117042435	5.587945508	5.036415022	3.06E-06	6.66E-05	3.970191605
ZNF330	0.193652781	4.792549649	5.018428071	3.28E-06	7.11E-05	3.931627624
FRMD6	0.609288744	8.116846773	5.014597228	3.33E-06	7.21E-05	3.912814103
RRS1	0.322475525	4.733177432	5.011947252	3.37E-06	7.27E-05	3.908888907
NANP	0.443165386	3.714574901	4.990831545	3.66E-06	7.78E-05	3.907808003
RECQL5	-0.256114776	3.005568504	-4.968656094	3.99E-06	8.35E-05	3.898476706
DUSP6	-0.494242646	4.819377349	-5.010055935	3.39E-06	7.31E-05	3.878674935
COPS2	0.10976785	7.147510258	5.01185038	3.37E-06	7.27E-05	3.864188205
RBM3	0.217272115	7.082893927	5.008501506	3.41E-06	7.34E-05	3.846349983
CBWD1	0.367990553	1.573951505	4.902745691	5.15E-06	0.000104844	3.845441148
SLC25A42	-0.318661047	2.120043608	-4.924355065	4.74E-06	9.73E-05	3.845089425
SDC4	0.320550149	7.390911002	5.004937934	3.46E-06	7.42E-05	3.836613279
STPG1	-0.504136145	2.089099174	-4.920612679	4.81E-06	9.85E-05	3.825816691
LSM14A	0.124248185	6.600632423	5.002761337	3.49E-06	7.47E-05	3.823505204
CBARP	0.432955274	2.767527913	4.942725291	4.41E-06	9.19E-05	3.813561153
TM2D3	0.199515833	4.605651352	4.979898308	3.82E-06	8.05E-05	3.800780496
ZFP36L2	0.431990108	8.211334793	4.983942056	3.76E-06	7.95E-05	3.795397942
ZBTB8A	0.254669811	3.372687839	4.940483164	4.45E-06	9.25E-05	3.792689787
UBE2N	0.166546169	5.730685474	4.988362985	3.69E-06	7.82E-05	3.785272816
SLC2A1	0.437298194	4.850747086	4.973772576	3.91E-06	8.22E-05	3.781688625
GPAT3	0.912039731	-0.90799554	4.895655369	5.29E-06	0.000107619	3.777451675
SPG20	0.12927797	7.214586345	4.982697467	3.77E-06	7.97E-05	3.758547076
NOP58	0.243141578	5.420869146	4.974399923	3.90E-06	8.21E-05	3.746398259
FASTKD2	0.235151634	4.684228162	4.958604922	4.15E-06	8.65E-05	3.729038933
MMACHC	-0.329528219	2.601503701	-4.890783015	5.39E-06	0.000109409	3.717547574
TRIM32	0.291721719	5.178829829	4.964165829	4.06E-06	8.48E-05	3.71381938
CIRBP	0.299022408	6.942349626	4.97138216	3.94E-06	8.29E-05	3.707657536
NELFA	-0.200754735	4.168108146	-4.939795445	4.46E-06	9.25E-05	3.705364895
ZNF33A	0.2493561	3.864810401	4.934832321	4.55E-06	9.41E-05	3.702544268
TUT1	0.331113122	2.333310399	4.873664156	5.76E-06	0.000115616	3.69398367
ZMIZ2	-0.172416606	5.812745499	-4.96621998	4.02E-06	8.42E-05	3.693167313
CASP10	-0.376678552	2.302150551	-4.88263316	5.57E-06	0.000112575	3.670165249
YRDC	-0.384660036	2.282296876	-4.879562876	5.63E-06	0.000113614	3.629545109
PIGW	0.357246097	3.010101968	4.876461927	5.70E-06	0.000114831	3.620403211
WDR5	-0.147273875	5.2201505	-4.937973916	4.49E-06	9.31E-05	3.616805735
DNAJA3	-0.222059552	4.771610831	-4.926839622	4.69E-06	9.66E-05	3.601885164
ZNF567	0.335605794	2.241035735	4.848208257	6.36E-06	0.000124894	3.580225457
ZFP2	-0.800185291	-0.631515057	-4.82456936	6.97E-06	0.000135038	3.572976323
HOXC4	0.514914797	2.046962249	4.836569707	6.65E-06	0.000129609	3.564346226
AMPD2	-0.198850105	5.663248499	-4.928168052	4.67E-06	9.64E-05	3.55837742
BRD8	-0.221361491	4.753636132	-4.911387049	4.98E-06	0.000101664	3.55223843
HP1BP3	0.27626509	7.15547024	4.926537633	4.70E-06	9.66E-05	3.53756064
ZKSCAN1	-0.387819886	6.429767393	-4.926654378	4.69E-06	9.66E-05	3.537102004
TMCC2	-0.45961657	1.883116238	-4.820293928	7.08E-06	0.000137101	3.531453034
RND3	-0.396890667	7.878790764	-4.916101092	4.89E-06	1.00E-04	3.528548988
RAB11A	0.105515554	6.564792938	4.922960077	4.76E-06	9.77E-05	3.522322695
GRAMD1B	-0.463424144	3.200630889	-4.872341508	5.79E-06	0.000116054	3.521568976
PXMP4	-0.278299578	3.190676768	-4.869332918	5.86E-06	0.000116945	3.50880649
TNFRSF12A	0.51114587	6.06408896	4.918397853	4.85E-06	9.92E-05	3.506587102
BDNF	-0.57709853	2.966599867	-4.883167518	5.56E-06	0.000112493	3.497744615
OXNAD1	-0.339399971	2.268654858	-4.831579974	6.78E-06	0.000131783	3.483182343
CXCL1	-1.081361395	0.319690729	-4.789440608	7.97E-06	0.000151234	3.478635674
WDTC1	-0.159936908	6.328783586	-4.910531642	5.00E-06	0.000101864	3.475971054
RUNDC1	0.272876502	3.734394251	4.867771252	5.90E-06	0.000117342	3.470516491
ZNF211	0.390831991	1.860854904	4.800730034	7.63E-06	0.000145927	3.462310463
ALG13	0.197523332	4.065535617	4.874835804	5.74E-06	0.000115247	3.461200679
ZFYVE28	-0.309634511	2.71854907	-4.832836984	6.75E-06	0.000131316	3.448819369
MIIP	-0.348369982	3.386265065	-4.855168034	6.19E-06	0.000122223	3.448549852
ANO8	-0.357208207	2.579205456	-4.840894767	6.54E-06	0.000128298	3.43079908
ZNF143	0.195942814	3.808283012	4.859026124	6.10E-06	0.000121211	3.422872503
ZSCAN30	-0.246919983	3.271318211	-4.840439708	6.55E-06	0.000128356	3.418446962
PPP1R15A	-0.398725674	6.371601883	-4.890699369	5.40E-06	0.000109409	3.402767955
ABCC10	-0.243215434	4.031343186	-4.858680002	6.11E-06	0.000121214	3.392374494
NCKIPSD	-0.232854745	3.784186763	-4.850290577	6.31E-06	0.000124329	3.390153676
SMAD7	-0.239067336	4.36427296	-4.870530699	5.83E-06	0.000116581	3.376008042
TBC1D10B	-0.169803223	6.10598866	-4.882182344	5.58E-06	0.00011262	3.374197049
CCDC127	-0.191074642	5.073980381	-4.870481245	5.84E-06	0.000116581	3.373872068

Gene	Log fold change	Average Expression	t	P-value	Adjusted P-value	B
PDK2	-0.209210205	4.648772209	-4.868442698	5.88E-06	0.000117192	3.370138431
IKBKB	-0.201874155	4.138388389	-4.856680983	6.15E-06	0.00012178	3.366546688
C5orf30	0.397019592	3.22123045	4.829238751	6.84E-06	0.000132804	3.363064185
CEP68	-0.28326379	4.418249454	-4.850061082	6.31E-06	0.000124329	3.356446198
RNF114	-0.144647035	6.07224011	-4.87606023	5.71E-06	0.000114856	3.349831556
BCL9	-0.318804622	4.094703329	-4.838064597	6.61E-06	0.000129199	3.335795161
LSM11	0.453697706	2.549601979	4.784861239	8.11E-06	0.000153333	3.327757292
ZNF226	0.261013155	3.280650477	4.814743754	7.23E-06	0.000139163	3.326031363
KIF3C	-0.324634842	4.98082714	-4.856450523	6.16E-06	0.00012178	3.322115748
IER2	-0.500784105	4.53734825	-4.858193634	6.12E-06	0.000121282	3.317052861
RBM33	-0.287094874	4.452201039	-4.837225771	6.63E-06	0.00012945	3.277353266
WDR91	-0.287319692	2.691396339	-4.781430306	8.22E-06	0.00015483	3.274462657
BORCS5	-0.300120457	3.271240499	-4.801084985	7.62E-06	0.000145913	3.264813417
LEMD2	-0.171254187	4.858318767	-4.839683351	6.57E-06	0.000128563	3.257439358
ZBTB7A	0.221836738	6.778198068	4.848402866	6.35E-06	0.000124894	3.244576357
ZNF284	0.704427317	0.303397103	4.713372249	1.07E-05	0.000192294	3.233913163
ZSWIM6	0.389000703	3.109707887	4.795814736	7.78E-06	0.000148211	3.220468093
USP2	0.634809592	0.081906327	4.706348604	1.09E-05	0.000196794	3.209145933
TMEM63B	-0.240855648	4.487301324	-4.816741405	7.18E-06	0.00013863	3.201603178
TLCD1	0.314311815	2.17816021	4.731880908	9.93E-06	0.000181819	3.19886717
C22orf29	0.257821948	4.402367796	4.811834381	7.31E-06	0.000140548	3.17808788
DCUN1D3	-0.33181779	4.725073927	-4.819881852	7.09E-06	0.000137142	3.165914664
DGKD	-0.350603401	2.708718347	-4.752440035	9.18E-06	0.00016975	3.162816565
SOCS1	0.697106496	1.54448754	4.703351224	1.11E-05	0.000198812	3.153256094
KLHL12	-0.161135635	4.599746151	-4.80274738	7.57E-06	0.00014517	3.138405299
KDM6A	-0.544400157	3.557018223	-4.765777672	8.73E-06	0.000163113	3.137867304
CDC5L	0.137243068	5.669054364	4.816315995	7.19E-06	0.000138679	3.135619277
GDF15	0.662635426	5.277656728	4.815694296	7.21E-06	0.000138833	3.125676305
ZNF687	-0.216763714	4.713311978	-4.794036453	7.83E-06	0.000148779	3.109852337
NAMPT	0.369243014	6.491049589	4.811334444	7.33E-06	0.000140639	3.109055568
CH25H	-0.54551413	4.342699748	-4.782651842	8.18E-06	0.000154442	3.08845939
VARS2	-0.200988792	3.763764119	-4.770243876	8.58E-06	0.000160749	3.085307288
SENP5	0.25391743	4.539675576	4.7859186	8.08E-06	0.000152904	3.082140024
BCL2L13	-0.144396488	5.734815929	-4.799727228	7.66E-06	0.000146304	3.073224604
DZIP1L	0.379116106	1.967074763	4.694808246	1.14E-05	0.000204397	3.060015766
PFKFB4	-0.504858323	1.803417212	-4.674475295	1.24E-05	0.000218456	3.058317952
DAZAP2	0.129125612	7.425819358	4.794750104	7.81E-06	0.00014856	3.054374156
TNPO3	-0.130337411	5.984627984	-4.795693041	7.78E-06	0.000148211	3.052987339
ZSCAN25	-0.291287645	3.384068857	-4.746739756	9.39E-06	0.000173273	3.046238171
OGT	-0.345247593	5.176867683	-4.774068232	8.45E-06	0.00015861	3.022136981
SLC38A7	-0.205041946	5.510646665	-4.781343713	8.22E-06	0.00015483	3.015597705
CABIN1	-0.155400573	6.393439527	-4.786499744	8.06E-06	0.000152756	3.014158329
FLI1	-0.281307128	3.071453523	-4.715581241	1.06E-05	0.000190913	3.002866984
HMGCR	0.709970782	6.877863802	4.779256689	8.29E-06	0.000155878	2.989334745
SLC4A2	-0.186635777	6.193424101	-4.776444267	8.38E-06	0.00015737	2.977439358
ZNF766	0.225480442	3.520778069	4.722019803	1.03E-05	0.000186963	2.972461291
CCDC14	-0.273821762	3.205299515	-4.736279609	9.77E-06	0.000179449	2.968542258
TXNDC11	0.169057178	5.054866074	4.761524544	8.87E-06	0.00016488	2.967377341
LRIF1	-0.23062213	3.627153635	-4.728546401	1.01E-05	0.000183254	2.9668302
DENND1A	-0.201727989	5.088579226	-4.765139072	8.75E-06	0.00016331	2.964679231
TPST1	0.16368381	5.469395438	4.769590221	8.60E-06	0.000160951	2.96378952
LTV1	0.313865997	4.09052415	4.742602612	9.54E-06	0.0001756	2.955692017
PIK3C2B	-0.555752623	1.576888022	-4.647776216	1.37E-05	0.00023888	2.952536479
IL11	0.759292995	0.213437919	4.633259534	1.44E-05	0.000250022	2.943788128
ARHGEF7	-0.218403214	5.415269136	-4.761343236	8.88E-06	0.00016488	2.938598378
WDR81	-0.191232792	6.027287479	-4.763370749	8.81E-06	0.000164011	2.933846846
EHMT1	-0.18060286	5.1063869	-4.752493604	9.18E-06	0.00016975	2.933125551
BLOC1S3	0.21879014	3.998903187	4.728942963	1.00E-05	0.000183199	2.930773688
MIDN	-0.29074108	6.055505755	-4.763543413	8.80E-06	0.000164011	2.929398624
EPOP	0.431080032	1.43303426	4.641304591	1.40E-05	0.000244226	2.919346657
MSANTD3	0.146929178	5.643247655	4.753284051	9.15E-06	0.000169619	2.905476664
BRD2	0.20952668	6.857142591	4.756056411	9.06E-06	0.000168039	2.904261016
TRIM35	0.212394467	4.745762943	4.736614844	9.76E-06	0.000179439	2.896828719
TMEM70	0.206558848	4.549988096	4.733719916	9.86E-06	0.00018084	2.893509171
SALL1	0.589435922	3.364114991	4.684505343	1.19E-05	0.000211295	2.89183676
DZANK1	-0.551935981	-0.091092223	-4.613316669	1.56E-05	0.000265294	2.887503687
FGD1	-0.215696651	5.443223203	-4.744514965	9.47E-06	0.000174537	2.884767352
NUFIP1	0.284009916	2.95549316	4.691287256	1.16E-05	0.000206657	2.880274268
KBTBD8	-0.598594006	-0.887965282	-4.613296394	1.56E-05	0.000265294	2.879523174
STX16	-0.234287406	4.25782021	-4.730233677	1.00E-05	0.000182521	2.87030833

Gene	Log fold change	Average Expression	t	P-value	Adjusted P-value	B
ZNF398	0.365206166	4.836544897	4.731203462	9.96E-06	0.000182068	2.867142325
ELMOD3	-0.255752859	2.856801377	-4.681687144	1.20E-05	0.000213315	2.861697818
BBS10	-0.271560078	3.723600921	-4.700670419	1.12E-05	0.00020037	2.861461813
SLF1	-0.465632315	1.421623919	-4.616033193	1.54E-05	0.000263468	2.849707873
STRIP1	-0.249109519	4.804768653	-4.72330395	1.03E-05	0.000186275	2.844592987
NDE1	-0.229940929	4.78411516	-4.720676219	1.04E-05	0.000187696	2.843568236
OSGEPL1	-0.297126605	1.747334524	-4.638004202	1.42E-05	0.000246738	2.841651837
LONRF1	0.323049777	3.465584677	4.690573919	1.16E-05	0.000206973	2.837422033
TRIM66	-0.529373009	1.334224702	-4.621496611	1.51E-05	0.000259573	2.832500093
EFNB1	0.212862939	6.236242073	4.733616495	9.87E-06	0.00018084	2.823541128
SNRPB2	0.249605057	5.497274187	4.725000912	1.02E-05	0.000185438	2.808828514
PBDC1	0.241676035	4.693931979	4.710578404	1.08E-05	0.000193885	2.803063397
PRPF40B	-0.234530882	2.000630495	-4.635236963	1.43E-05	0.000248734	2.794418608
DIDO1	-0.417734442	5.996408093	-4.724802207	1.02E-05	0.000185438	2.791314071
NR4A2	-0.632261845	0.472193928	-4.593341805	1.68E-05	0.000281203	2.775565673
TXNRD1	0.348959635	8.063923037	4.712520935	1.07E-05	0.000192688	2.773537416
TIMM29	0.207248916	3.419324617	4.667657868	1.27E-05	0.000223124	2.769411616
SMIM15	0.119694034	5.731025582	4.716434374	1.05E-05	0.000190523	2.766198937
SNAI1	0.454667341	3.442302807	4.688519127	1.17E-05	0.000208347	2.757442149
ZNF547	0.494583891	0.614890244	4.575747104	1.79E-05	0.000296447	2.750814701
USP54	-0.332310675	2.422499853	-4.624836098	1.49E-05	0.000256662	2.732268449
AKNA	-0.315982086	4.643417091	-4.69262388	1.15E-05	0.000205855	2.729993663
GRAMD1A	-0.223853128	5.456938896	-4.70087693	1.12E-05	0.00020037	2.715522799
MFSD2A	0.54883388	1.234575099	4.606744416	1.60E-05	0.000270085	2.710586849
PLEKHM1	-0.150386152	5.4942437	-4.698156571	1.13E-05	0.000202053	2.7087716
SHROOM1	-0.354095504	1.894744503	-4.592087237	1.69E-05	0.000282089	2.687643949
BLOC1S5	-0.233409221	3.343203097	-4.637982636	1.42E-05	0.000246738	2.684444444
SZT2	-0.380980166	4.669763821	-4.670483687	1.25E-05	0.000221007	2.658490505
RGP1	-0.342352905	4.831183225	-4.673919914	1.24E-05	0.00021866	2.656545941
SPATS2	-0.153773532	4.290155797	-4.664368384	1.28E-05	0.000225657	2.656383889
MBTD1	-0.368884258	2.461789828	-4.600809203	1.63E-05	0.000274948	2.651261952
MORF4L2	0.137749899	7.707411731	4.676018065	1.23E-05	0.000217693	2.627351751
NUDT4	0.243329437	4.009754181	4.647044314	1.37E-05	0.000239264	2.624966402
SH3BP2	-0.198494489	4.365007446	-4.652534832	1.34E-05	0.000234894	2.616229898
USP10	0.283954211	5.554497513	4.670800787	1.25E-05	0.000221	2.616080581
TOR1A	0.127444741	5.902208806	4.675671743	1.23E-05	0.000217723	2.612988536
RUBCNL	0.416369884	3.343656875	4.610931837	1.57E-05	0.000266854	2.587019326
USP42	0.28304728	3.788652755	4.627676096	1.47E-05	0.000254758	2.576291414
HNRNPA3	0.155481282	6.324227563	4.661596235	1.30E-05	0.000227772	2.557211606
MORF4L1	0.100743916	6.980087633	4.659432387	1.31E-05	0.000229377	2.548972583
S100PBP	-0.265261297	4.048570883	-4.619275669	1.52E-05	0.000261158	2.54812914
AGAP3	-0.206094906	6.052614064	-4.654564626	1.33E-05	0.000233369	2.537013512
TAF3	-0.204941686	4.130986198	-4.624790848	1.49E-05	0.000256662	2.533855088
CDC14A	-0.635509522	0.478102799	-4.508163132	2.31E-05	0.000370631	2.522565896
LINC00847	-0.334548478	2.836237334	-4.564668623	1.87E-05	0.00030733	2.493853648
SEMA6C	-0.278813153	3.207102384	-4.59191307	1.69E-05	0.000282089	2.493037424
LARP7	0.169047631	4.916535587	4.631379174	1.45E-05	0.000251513	2.492987091
ARL8A	0.221588077	4.566547735	4.626056188	1.48E-05	0.000256026	2.488857276
MXD1	0.327899213	2.909062878	4.576069326	1.79E-05	0.000296414	2.478022116
CCT2	0.141603357	6.986108937	4.635331825	1.43E-05	0.000248734	2.460480224
ZNF346	-0.19632306	3.178793408	-4.575185093	1.80E-05	0.000296747	2.459125231
FAM160B2	-0.115405896	5.935836743	-4.633862052	1.44E-05	0.000249741	2.458517477
BCL7A	0.338514634	3.164140339	4.57253224	1.81E-05	0.000299385	2.443908103
POU2F2	0.47843724	3.80042286	4.584358275	1.74E-05	0.000288605	2.443745076
ZNF420	0.399862458	2.533401702	4.531407137	2.12E-05	0.000344274	2.438284485
ZHX2	-0.233859293	4.825603493	-4.616168665	1.54E-05	0.000263468	2.436704372
PPP6R2	-0.126310143	5.229718416	-4.620729698	1.51E-05	0.000260027	2.431442404
CRTC2	-0.212546624	4.59983535	-4.605891358	1.60E-05	0.000270472	2.427653947
GOLPH3L	-0.257442981	4.941956626	-4.610839258	1.57E-05	0.000266854	2.426556543
SRSF12	0.835543743	-0.99403412	4.500048944	2.38E-05	0.000379163	2.421259926
KDM6B	-0.378797776	4.51221032	-4.607734591	1.59E-05	0.000269687	2.418204105
ZNF280D	-0.152311293	4.60532195	-4.603721255	1.61E-05	0.000272257	2.414543854
POLR1E	0.169650256	5.354264436	4.612455629	1.56E-05	0.000265834	2.399466037
CXXC5	0.240346959	6.988256951	4.618270534	1.53E-05	0.000261851	2.397793316
TMEM86A	-0.332240819	2.002648475	-4.516568296	2.24E-05	0.000360432	2.39611469
MMS19	-0.166405535	5.77489551	-4.614468366	1.55E-05	0.000264725	2.393368668
ZNF502	-0.426332619	1.775961075	-4.503699684	2.35E-05	0.000375433	2.393252311
SH2B2	0.493772772	1.939205095	4.502706928	2.36E-05	0.000375955	2.391351082
DNAJC16	-0.306765456	3.686301898	-4.570951129	1.83E-05	0.000300836	2.390611046
SPTSSA	0.164621975	4.848344126	4.597776925	1.65E-05	0.000276862	2.385363919

Gene	Log fold change	Average Expression	t	P-value	Adjusted P-value	B
ZNF202	0.240380626	3.027302651	4.546408677	2.00E-05	0.000327279	2.375850379
TTLL11	-0.281089814	2.453445363	-4.521815607	2.19E-05	0.000355663	2.374753557
RCHY1	-0.262388929	3.27837869	-4.563430077	1.88E-05	0.000308422	2.372528481
DAZAP1	0.144009078	5.808602917	4.607343462	1.59E-05	0.00026978	2.369140014
PPIG	-0.144723951	5.660416213	-4.605767442	1.60E-05	0.000270472	2.366131403
FASTKD5	-0.188831065	3.997832803	-4.581690118	1.75E-05	0.000290868	2.360871027
RSF1	-0.353107326	5.387946444	-4.599050519	1.64E-05	0.000275847	2.359695733
MAGEH1	0.157938092	4.888739397	4.590935843	1.69E-05	0.000282499	2.354341585
BOLA3-AS1	-0.38118889	1.389448032	-4.478302637	2.58E-05	0.000405065	2.347823053
ERRFI1	-0.488421273	5.02512228	-4.599231621	1.64E-05	0.000275847	2.336779043
GLI3	-0.377810228	6.411526831	-4.599509372	1.64E-05	0.000275847	2.3302008
TMEM97	0.371447051	5.282706806	4.584925792	1.73E-05	0.000288309	2.323324079
NEPRO	0.178147323	5.422436443	4.589634073	1.70E-05	0.00028357	2.316197743
C21orf58	-0.352167081	0.914141676	-4.460677238	2.75E-05	0.000429724	2.312855394
FAM200A	0.350883201	2.545182441	4.495179801	2.42E-05	0.000384869	2.310775098
ZNF638	-0.193327471	5.905801067	-4.591428891	1.69E-05	0.000282289	2.306744878
DTWD1	-0.158834312	5.053434572	-4.583172599	1.74E-05	0.000289573	2.306214782
RPAP1	-0.219259869	4.342937164	-4.559419956	1.91E-05	0.000312748	2.293910482
VCPIP1	0.20752407	4.707243851	4.569664854	1.83E-05	0.000301959	2.287840353
KIZ	-0.294497769	5.067634796	-4.578159293	1.78E-05	0.000294423	2.275019634
ZNF879	0.376870704	1.20535359	4.441870401	2.95E-05	0.000455595	2.267726675
ZNF443	0.668979677	-0.555496334	4.43702849	3.00E-05	0.000462762	2.265668674
NEMP1	-0.391099411	3.490535429	-4.517608905	2.23E-05	0.000359989	2.20585111
TWNK	0.317319674	3.423486625	4.516228147	2.24E-05	0.000360432	2.204312478
CDK20	-0.294815905	2.575891556	-4.483207447	2.53E-05	0.000399424	2.182455658
NRBF2	-0.17031028	4.731662457	-4.539586611	2.05E-05	0.000335003	2.171608305
REPS1	-0.123307048	4.849108226	-4.540212077	2.05E-05	0.000334583	2.168487498
KPNA4	0.194001082	6.625467506	4.554770565	1.94E-05	0.000317892	2.168087974
SUMO1	0.123824292	5.928596607	4.549486242	1.98E-05	0.000323886	2.155579254
ATF7	-0.148284268	4.946653077	-4.530387233	2.12E-05	0.000345214	2.128774917
NSUN6	-0.432746117	1.301557964	-4.412710246	3.29E-05	0.000496974	2.121051141
ARF4	0.134680237	8.532967593	4.526669853	2.15E-05	0.000349659	2.115723609
DCLRE1A	-0.256396198	2.444125205	-4.456886831	2.79E-05	0.000434446	2.110986536
UBE2J1	0.137227602	6.515118364	4.536873787	2.07E-05	0.000338049	2.103631759
PLEKHO2	0.329220554	5.865265539	4.531493688	2.12E-05	0.000344274	2.095899725
C15orf41	-0.203125614	3.481173758	-4.48935268	2.48E-05	0.000392054	2.094325265
TNRC6B	-0.276960583	5.035963896	-4.519660817	2.21E-05	0.000357762	2.093197495
ARAP3	-0.283871753	3.374320256	-4.479925727	2.56E-05	0.000403474	2.084529447
CNNM2	-0.194640047	3.962263945	-4.49671514	2.41E-05	0.000383083	2.077808639
SMARCAD1	-0.290377189	4.638080866	-4.506970642	2.32E-05	0.000371488	2.069941165
LRRC4	0.452654275	0.856459792	4.379052145	3.72E-05	0.00054871	2.058406225
MNT	-0.237750362	5.318655716	-4.520248945	2.21E-05	0.000357362	2.052306146
C11orf63	-0.280932695	2.432765876	-4.444752663	2.92E-05	0.00045159	2.051378477
ETFBKMT	-0.396238865	0.872196008	-4.38031948	3.70E-05	0.00054689	2.050181201
VPS37B	0.190286073	5.133458168	4.515072088	2.25E-05	0.000361602	2.049781281
TRIM65	-0.227206595	4.249146655	-4.483302487	2.53E-05	0.000399424	2.045978321
RGCC	0.592086739	3.982165762	4.481335836	2.55E-05	0.000401788	2.043509483
SNHG15	0.405201415	2.333527588	4.423544818	3.16E-05	0.000482325	2.040972135
GGNBP2	-0.174572983	5.878955322	-4.517419562	2.23E-05	0.000359989	2.036874223
METTL15	-0.289976819	2.16709197	-4.423325269	3.16E-05	0.000482325	2.036646092
RPP38	0.256577911	2.404980268	4.427907961	3.11E-05	0.000476656	2.035325127
MIS18BP1	-0.34186166	4.083837049	-4.478804642	2.57E-05	0.000404733	2.031779263
ZNF703	0.275321271	6.374036371	4.516415644	2.24E-05	0.000360432	2.031201233
RAD1	0.209546368	4.710254585	4.502617498	2.36E-05	0.000375955	2.031027345
ZBTB12	-0.355508685	2.617421381	-4.432773422	3.05E-05	0.00046913	2.022394201
CCDC12	0.340022492	4.16000263	4.487669569	2.49E-05	0.000394098	2.018385172
MRPL47	0.231280675	4.775175895	4.492038552	2.45E-05	0.000388979	2.005031234
SYMPK	-0.144254566	6.20929931	-4.507232662	2.32E-05	0.000371488	1.998079458
ADNP	-0.209306422	6.338062392	-4.503560909	2.35E-05	0.000375433	1.984742207
FANCF	0.257919352	2.894467919	4.414629456	3.26E-05	0.000495023	1.979868903
ZNF594	0.595081393	0.436413872	4.343431714	4.24E-05	0.000616075	1.974025332
ZNF136	-0.216604858	2.64367285	-4.422344721	3.17E-05	0.000483582	1.971964898
FBRS	-0.142589988	5.970577001	-4.496795352	2.41E-05	0.000383083	1.963004057
RXRA	-0.1824304	7.177806356	-4.489898327	2.47E-05	0.000391671	1.941259411
ACVR2A	-0.391737431	5.348943487	-4.486574358	2.50E-05	0.000395289	1.939046072
CYP4V2	0.192413587	4.624791229	4.468280156	2.68E-05	0.000419092	1.929308814
IKBKE	-0.268377852	3.281560659	-4.423585275	3.16E-05	0.000482325	1.92927653
DIMT1	0.136155648	5.127065329	4.472289394	2.64E-05	0.000413769	1.919464807
NAIF1	-0.238130506	2.903628999	-4.412518799	3.29E-05	0.000496974	1.910206822
MGC12916	0.546266817	0.649175589	4.337883773	4.32E-05	0.000626264	1.90890845

Gene	Log fold change	Average Expression	t	P-value	Adjusted P-value	B
ING4	-0.228152693	4.103207248	-4.456583598	2.79E-05	0.000434485	1.908033858
STAT5A	-0.170413471	4.999452527	-4.46219626	2.74E-05	0.000427756	1.886415727
PALB2	0.336936646	3.333149147	4.408654637	3.34E-05	0.000502482	1.883228185
FAXDC2	-0.209781666	4.699607346	-4.45838469	2.78E-05	0.00043249	1.873757263
C16orf91	0.27216794	2.554055955	4.388706363	3.59E-05	0.000534847	1.872413323
ZSCAN26	-0.232832796	3.204013778	-4.401062733	3.43E-05	0.000513644	1.869237051
KAT5	0.155902747	4.366403128	4.445918653	2.91E-05	0.000450573	1.86292644
GBP1	0.404168144	4.986768263	4.462222447	2.74E-05	0.000427756	1.860645626
MOB3A	-0.150934509	6.454264974	-4.468570819	2.67E-05	0.000419076	1.859532471
SIX5	-0.280249774	3.348596153	-4.41246991	3.29E-05	0.000496974	1.852791302
AJUBA	0.373025709	4.579324189	4.450243687	2.86E-05	0.000444338	1.848328243
WEE2-AS1	-0.752294698	-1.139139686	-4.306850522	4.84E-05	0.000689951	1.845660951
TAF5L	0.284065477	4.046197342	4.430409006	3.08E-05	0.000472759	1.83431892
LOC100505715	-0.622116666	-0.986525773	-4.297776024	5.00E-05	0.000708536	1.826262212
RBM12	0.249202386	6.456127925	4.458816593	2.77E-05	0.000432246	1.82535023
TMEM55B	0.306203688	4.712674201	4.44501185	2.92E-05	0.00045159	1.823435617
ZNF613	0.318735311	1.740498447	4.326669615	4.50E-05	0.000646815	1.82311007
SUPT7L	-0.127287724	4.529829642	-4.440480997	2.97E-05	0.000457366	1.816353413
ANKRD27	-0.287212825	4.439683229	-4.424474201	3.15E-05	0.00048175	1.815975592
TMEM41B	0.40776055	3.894395529	4.419496839	3.21E-05	0.000486715	1.814270798
RPP14	0.232811686	3.622769942	4.408388233	3.34E-05	0.000502482	1.812868573
POU3F1	1.259605901	-3.664674794	4.382968234	3.67E-05	0.000545154	1.808556284
SELENOF	0.110029925	7.369374199	4.449235787	2.87E-05	0.000445538	1.801073965
GPATCH11	-0.321919158	3.238936096	-4.38044274	3.70E-05	0.00054689	1.799109169
NARFL	-0.297215671	3.878020926	-4.408965539	3.33E-05	0.00050242	1.786282868
DPH6	0.29065752	1.795588996	4.329959302	4.45E-05	0.000639713	1.774438371
PSMD12	0.18598682	5.718504508	4.4418083	2.95E-05	0.000455595	1.773351515
ARPC5L	0.152144234	4.698215697	4.419792749	3.20E-05	0.000486715	1.761131846
RBM4B	-0.200018464	3.84995906	-4.404311545	3.39E-05	0.000508553	1.760899172
MBLAC2	-0.266909294	2.671706852	-4.351426916	4.11E-05	0.000601237	1.755500946
ADM2	-0.410122636	3.973933106	-4.420932002	3.19E-05	0.000485616	1.752660083
ZMAT3	0.228897898	7.3940892	4.433375283	3.05E-05	0.000468567	1.747462361
ZNF217	-0.347485326	5.689264931	-4.427150425	3.12E-05	0.000477504	1.730718881
KLHDC1	-0.40489539	0.626942896	-4.28678419	5.21E-05	0.000734468	1.725159706
ALMS1-IT1	1.07218242	-2.423715252	4.31314193	4.73E-05	0.000676259	1.721738384
SLC29A3	-0.226744821	3.722881265	-4.386277233	3.62E-05	0.000539105	1.719555661
PARL	0.186427392	4.789447649	4.410361087	3.31E-05	0.000500346	1.716118561
RIOK1	0.223545772	4.498889724	4.405478126	3.37E-05	0.00050688	1.716056017
CENPV	0.373829634	0.78881615	4.278124284	5.37E-05	0.000752959	1.715260193
VRK3	-0.188586486	4.243580353	-4.396046807	3.49E-05	0.000522684	1.711169464
FAM212B	0.555560977	2.843669715	4.342865519	4.25E-05	0.000616755	1.710519297
PGBD4	-0.521875537	0.980735888	-4.276854504	5.40E-05	0.000755026	1.707771271
PHF8	-0.285394401	4.010786908	-4.381499541	3.69E-05	0.000546482	1.693013029
COX11	0.15785533	4.395684981	4.39414896	3.52E-05	0.00052582	1.692968804
C10orf10	-0.552443942	4.323428172	-4.382678372	3.67E-05	0.000545161	1.688719755
ARHGAP24	-0.248743954	5.977796573	-4.419590896	3.20E-05	0.000486715	1.687709903
GPSM2	-0.32326613	4.111563199	-4.382427322	3.67E-05	0.000545161	1.672399671
PWP1	0.108165134	5.971727565	4.41362825	3.28E-05	0.000496352	1.670547256
PRAG1	0.645031392	2.134616295	4.317178449	4.66E-05	0.000667662	1.651630839
ZNF689	-0.20162084	3.648618044	-4.3550308	4.06E-05	0.000593942	1.644302822
POLG	-0.194629251	4.915980749	-4.390008453	3.57E-05	0.000533351	1.642564599
RAB18	0.13209541	6.441823098	4.406924293	3.36E-05	0.000504693	1.640920284
B4GALT4	0.204719315	4.083511217	4.369521515	3.85E-05	0.000566556	1.637300099
USP6NL	-0.202702332	3.925009497	-4.366722338	3.89E-05	0.000571837	1.636299058
UVSSA	-0.482112375	1.531044884	-4.290053453	5.15E-05	0.000726488	1.635572466
CCDC121	-0.43312003	0.445252795	-4.247076429	6.01E-05	0.000825541	1.632245875
TRIM16	-0.243309131	2.859031562	-4.331246342	4.43E-05	0.000637326	1.62745613
PNPLA6	-0.157081972	5.93706266	-4.401462584	3.43E-05	0.000513399	1.626767766
ZBED4	0.327706394	4.285177119	4.3726535	3.81E-05	0.000561187	1.622853196
GDF5	0.457629005	3.56404684	4.333424151	4.39E-05	0.000633394	1.604031142
NAE1	0.175029598	5.43173361	4.389378543	3.58E-05	0.000534057	1.598404378
MICALL1	0.219399217	5.598030238	4.380225151	3.70E-05	0.00054689	1.56417236
TMEM171	-0.518360747	0.622417593	-4.234113629	6.30E-05	0.000860348	1.553555276
IDI1	0.374274544	6.579023238	4.380911288	3.69E-05	0.00054689	1.551196851
SEMA7A	0.439237437	4.732776758	4.350974221	4.12E-05	0.000601649	1.541790362
NOL11	0.238015234	4.867351777	4.360570274	3.98E-05	0.000583157	1.540007254
ZBED8	0.549960253	1.013492394	4.211365352	6.84E-05	0.000920389	1.535806255
TMEM87A	0.172657884	5.537191058	4.371209877	3.83E-05	0.000563612	1.531449575
MUM1	-0.157681287	5.182907381	-4.361976262	3.96E-05	0.000581295	1.516556306
BCL2L11	-0.41292593	3.272226296	-4.299335916	4.98E-05	0.000705717	1.48722357

Gene	Log fold change	Average Expression	t	P-value	Adjusted P-value	B
THAP11	-0.17091818	4.348596728	-4.333439718	4.39E-05	0.000633394	1.487018779
NOP56	0.21938683	6.216329045	4.361585684	3.96E-05	0.000581559	1.483546683
VASN	0.206913538	8.661280365	4.345346179	4.21E-05	0.000612965	1.481583421
ZNF37A	-0.187755883	4.646957081	-4.338914767	4.31E-05	0.000625114	1.475518699
COPS8	0.128938317	6.46311088	4.356439221	4.04E-05	0.000591464	1.463670233
UPF3A	0.210022643	3.362905363	4.30028095	4.96E-05	0.000703961	1.462664132
ADPGK	0.140277885	5.115049289	4.343710233	4.23E-05	0.000616043	1.462028221
FAM46B	-0.741539381	0.234708813	-4.200377476	7.11E-05	0.000951477	1.459698345
PGM3	0.186621434	5.90699653	4.348948694	4.15E-05	0.000605534	1.450134005
LRRC20	-0.229120528	3.017603277	-4.293652888	5.08E-05	0.000718405	1.44734608
CNBP	0.181855772	8.118427518	4.33607946	4.35E-05	0.000629796	1.429311122
PTPN1	0.268110721	4.853898859	4.333204749	4.40E-05	0.000633394	1.423151832
CBX2	0.680801085	0.174233429	4.171214365	7.90E-05	0.001029714	1.417047352
THAP2	-0.353169369	2.043780181	-4.217765586	6.68E-05	0.000904188	1.413299931
SMIM13	0.271387152	3.944552274	4.304501877	4.88E-05	0.000694553	1.409132933
ZBTB4	-0.166775827	7.704250994	-4.335319588	4.36E-05	0.000630334	1.408411597
FEZ2	0.12043073	5.889278104	4.338207691	4.32E-05	0.000626126	1.40803192
ADPRHL2	0.170574428	4.630986313	4.321060146	4.60E-05	0.000658916	1.402914389
IKBIP	0.107828828	6.354412931	4.335745807	4.36E-05	0.000629958	1.392245624
PITPNM2	-0.261782047	3.715021363	-4.282920405	5.28E-05	0.000741361	1.382982863
INVS	-0.248912688	3.617190691	-4.285311123	5.24E-05	0.000736345	1.368424238
PI4KAP1	0.687777799	-1.355300831	4.161137618	8.18E-05	0.001060116	1.361116763
TMEM263	0.232199868	6.860543097	4.326387222	4.51E-05	0.000646865	1.359138608
BBOF1	0.208595197	2.862484687	4.249559655	5.96E-05	0.000820417	1.358746122
CREBRF	-0.405425197	4.720975666	-4.308128822	4.82E-05	0.000687401	1.34858323
ZNF829	-0.297727676	2.270884654	-4.22266012	6.56E-05	0.00089253	1.345052907
ADM	-0.33152395	8.095080254	-4.306301064	4.85E-05	0.000690677	1.335583234
AARS2	-0.243741858	3.605816219	-4.269696937	5.54E-05	0.000773416	1.332329245
GFPT2	-0.139081455	6.503074872	-4.316708948	4.67E-05	0.000668169	1.324381182
MAP1LC3B	0.119365246	7.202556774	4.311708749	4.76E-05	0.00067915	1.312718104
BAG4	0.209030292	3.976315167	4.270725807	5.52E-05	0.000771256	1.310762789
TLE1	0.259880138	4.338020448	4.285089473	5.24E-05	0.000736345	1.310331196
NACAD	-0.279331079	3.537341547	-4.257029554	5.80E-05	0.00080322	1.305152884
PLEKHG1	-0.507053864	4.577222543	-4.292157508	5.11E-05	0.000721638	1.30514807
TSPYL5	-0.2061537	4.215347758	-4.282403344	5.29E-05	0.000742061	1.291828207
SPOP	-0.116076939	5.599192332	-4.301012913	4.95E-05	0.000702753	1.287395202
PUS10	-0.43895644	0.509303164	-4.14966955	8.53E-05	0.001098719	1.286399142
FKRP	0.166583047	3.861484264	4.267214821	5.59E-05	0.000779673	1.285652807
CEP57L1	-0.279514997	2.365152228	-4.207600818	6.93E-05	0.000930426	1.285092786
HMGN3	0.268434036	3.496645585	4.248660791	5.98E-05	0.000822332	1.27836035
SETD3	0.113295097	5.592433951	4.297720416	5.00E-05	0.000708536	1.277549359
AURKA	-0.421903724	2.123851608	-4.188369606	7.42E-05	0.000985461	1.260109132
ZNF605	-0.30260745	3.502106665	-4.239523933	6.18E-05	0.000846032	1.248244235
ZNF629	0.237106981	5.579172169	4.285049896	5.24E-05	0.000736345	1.240676642
FJX1	0.26510497	3.784608156	4.251510621	5.92E-05	0.00081615	1.232986736
RFX2	-0.201654654	3.669379807	-4.244123506	6.08E-05	0.000833626	1.220902492
CUL7	-0.157970513	6.204979144	-4.286243562	5.22E-05	0.000735221	1.218915257
C16orf87	0.261388177	2.07931515	4.183062479	7.57E-05	0.000995599	1.21192096
TFAM	0.221902259	4.728123597	4.265481567	5.62E-05	0.000783128	1.20936445
NCL	0.134593538	9.27532227	4.25806574	5.78E-05	0.00080145	1.207067401
GAS2L1	-0.21371478	4.764370587	-4.264004232	5.66E-05	0.000786597	1.201830332
TRMT10B	-0.250770729	1.787123339	-4.168341063	7.98E-05	0.001038545	1.192054855
GPR157	0.562394999	0.70436718	4.118650434	9.52E-05	0.001204245	1.190082285
SBDS	0.161150507	6.427652966	4.277313742	5.39E-05	0.000754471	1.188342808
TTC23	-0.208138734	4.056229276	-4.237794779	6.22E-05	0.000849778	1.184992927
FOPNL	0.123315655	5.009981582	4.266036733	5.61E-05	0.000782279	1.184538806
TVP23B	0.254208294	2.903783655	4.2017133	7.08E-05	0.000948612	1.180745134
USP28	-0.244924344	4.548020771	-4.254382222	5.86E-05	0.000809953	1.179311338
RSBN1L	0.324007387	3.75563	4.221643	6.59E-05	0.000894188	1.178779242
PPAN	0.393138947	1.187058158	4.137237561	8.91E-05	0.001139691	1.178619897
PCGF3	-0.18416924	4.412280723	-4.251883258	5.91E-05	0.000815799	1.177305503
NUP35	0.277393004	2.40407387	4.180301625	7.64E-05	0.001002112	1.168930777
PTHLH	-0.331631051	2.207504892	-4.18146618	7.61E-05	0.001000431	1.166872462
ZNF682	0.537209366	0.1026498	4.093006297	0.00010427	0.001297044	1.163490036
ZNF500	0.231912466	2.975792572	4.187323741	7.45E-05	0.000986552	1.160184019
VBP1	0.136329273	5.222620627	4.258945861	5.76E-05	0.000799641	1.15789609
CEP295	-0.53738882	1.657124274	-4.118287739	9.53E-05	0.001204785	1.148599436
SPHK1	0.31235031	6.247033246	4.263130751	5.67E-05	0.000788358	1.144229607
PPME1	-0.155948323	5.100861017	-4.247593078	6.00E-05	0.000824755	1.134388701
PTP4A2	0.093936314	8.142175113	4.250110356	5.95E-05	0.000819537	1.131020778

Gene	Log fold change	Average Expression	t	P-value	Adjusted P-value	B
ZNF252P	-0.191221835	3.772279088	-4.218578819	6.66E-05	0.000902473	1.130855348
PPP6C	0.102480797	5.832980132	4.256946408	5.80E-05	0.00080322	1.123063459
SLC25A33	0.29506866	2.085052537	4.16986417	7.93E-05	0.001033801	1.117184427
RRAD	0.668220021	-0.756764208	4.075900325	0.000110768	0.001369955	1.114256323
KDM8	0.292198264	1.484284716	4.120850041	9.45E-05	0.00119671	1.113540837
STK3	-0.251102999	4.469067644	-4.23385533	6.31E-05	0.000860369	1.108657791
TSKU	0.191025426	6.163184052	4.252479638	5.90E-05	0.00081479	1.107011533
DNAL4	-0.247483035	3.246953215	-4.201434775	7.09E-05	0.000948716	1.099553306
TRIM23	0.379023701	3.778724297	4.195750237	7.23E-05	0.000963978	1.09562244
NASP	0.227240931	4.889233952	4.231028722	6.37E-05	0.000866821	1.089511675
PIK3CB	-0.321994614	3.44721703	-4.196881415	7.20E-05	0.000960925	1.088804634
NR4A1	-0.455891228	3.849953621	-4.240297237	6.16E-05	0.000844443	1.085303329
ZNF485	0.632289357	0.318735159	4.066028847	0.000114693	0.001410396	1.075488154
BCOR	0.483749486	4.543805943	4.213531301	6.78E-05	0.000914069	1.070944179
SAR1A	0.154908104	7.544992381	4.237965939	6.21E-05	0.000849778	1.064793474
CBFB	0.170189852	5.350373981	4.231650049	6.36E-05	0.000865665	1.059398266
RIMKLB	0.270792613	3.552132045	4.184358628	7.53E-05	0.000993593	1.041356218
STK17A	0.248052379	5.560302075	4.232844843	6.33E-05	0.000862726	1.041334878
ZNF701	-0.2952545	1.697593877	-4.107555526	9.90E-05	0.001243196	1.038351467
THAP1	-0.219363324	3.211325032	-4.180856497	7.63E-05	0.001001743	1.035923913
DLGAP1	-0.785985154	-1.327195089	-4.04654374	0.000122837	0.001486278	1.01478037
NAA15	0.338097379	5.347745619	4.217551409	6.69E-05	0.000904188	1.012892006
PRKRA	0.105750719	5.018782723	4.209755702	6.88E-05	0.000924076	1.000206902
COPS7B	-0.118395726	4.122414883	-4.187797681	7.44E-05	0.000985745	0.998823107
PXDC1	0.293388974	5.887370061	4.222360767	6.57E-05	0.000892686	0.998452762
SFPQ	0.213295218	7.289650633	4.21900763	6.65E-05	0.000901893	0.99297773
PATZ1	-0.138954524	4.269901727	-4.187797615	7.44E-05	0.000985745	0.992084124
TSPAN3	0.1088279	7.662338896	4.213892954	6.78E-05	0.000913699	0.98863134
CPEB2	0.458243151	3.635671408	4.173539316	7.83E-05	0.001022962	0.985566224
RUNX1T1	-0.341155924	5.028120381	-4.198555973	7.16E-05	0.000956866	0.978413912
CREG1	0.124615745	7.576877663	4.210914702	6.85E-05	0.000921057	0.975432731
DDX1	0.136805535	6.900306509	4.215228847	6.74E-05	0.000910953	0.974577676
ARHGEF40	-0.118749725	6.673246667	-4.214257999	6.77E-05	0.000913318	0.96980362
FAM222A	0.498397751	-0.085923135	4.032262318	0.000129156	0.001553999	0.962156143
SH3BGRL	0.102574665	7.337259703	4.205447574	6.98E-05	0.000936814	0.949683514
NCK1	-0.129883117	5.021513417	-4.193110159	7.30E-05	0.000972286	0.937629114
ZNF609	-0.322432346	5.171044782	-4.189219622	7.40E-05	0.00098333	0.936572867
CNNM3	-0.196355749	3.676907502	-4.158446756	8.26E-05	0.001069428	0.930256026
MBNL1-AS1	0.210478563	2.690370695	4.115501416	9.63E-05	0.001212381	0.92801395
TAB1	-0.199454385	4.765249553	-4.183537233	7.55E-05	0.000995599	0.922529436
TFCP2	-0.126322317	4.721485911	-4.180266747	7.64E-05	0.001002112	0.922439962
KNOP1	-0.192927625	3.037104652	-4.12275639	9.38E-05	0.001190812	0.917646686
NAB2	0.211533689	6.052512216	4.196898723	7.20E-05	0.000960925	0.914755059
LIN9	-0.365566772	1.435188246	-4.048657206	0.000121927	0.001481222	0.912020404
MBIP	0.140741373	3.89975868	4.156698697	8.32E-05	0.001075196	0.905463965
RNF139	0.170943468	5.456104894	4.190948314	7.36E-05	0.00097812	0.904360875
TLE4	0.350963672	3.461626758	4.145126081	8.67E-05	0.001113796	0.902932951
ATG2A	-0.145137026	5.314992275	-4.184558827	7.53E-05	0.000993593	0.901205556
SP4	0.408379392	2.365578077	4.106045866	9.96E-05	0.001248826	0.900500601
DUSP4	0.52123353	3.263555843	4.134218602	9.01E-05	0.001149074	0.898096643
THAP3	0.272420621	3.063095242	4.114739953	9.65E-05	0.001213941	0.896813772
HNRNPH2	0.123357224	6.339460511	4.191678103	7.34E-05	0.000976427	0.893418654
DOK1	0.187117819	4.87261833	4.17573884	7.77E-05	0.001015827	0.892504156
RIN1	0.219309077	5.453184069	4.179213188	7.67E-05	0.001005023	0.880420606
IER3	-0.430964956	4.822627109	-4.183215098	7.56E-05	0.000995599	0.880171512
NOP16	0.381064093	3.354261287	4.120870719	9.45E-05	0.00119671	0.879983904
PDP1	-0.332683935	4.644967231	-4.177081569	7.73E-05	0.001011838	0.879767122
PTPN23	-0.146000187	6.982300781	-4.186753424	7.47E-05	0.000987702	0.877740164
PPP2R5D	-0.164048392	5.904852937	-4.185528384	7.50E-05	0.000991175	0.875590665
CENPA	-0.574188756	0.452114647	-4.010280101	0.000139497	0.001653326	0.866117383
FAM177A1	0.161913236	5.059616403	4.172417735	7.86E-05	0.001026182	0.86539058
C6orf47	0.156740853	4.195859606	4.146174341	8.63E-05	0.001110591	0.861692952
GMIP	-0.24740319	2.603977769	-4.087065347	0.000106484	0.001321312	0.861448736
EBLN3P	-0.233136095	5.018992113	-4.166003255	8.04E-05	0.00104634	0.848943524
SPRTN	-0.189671957	3.293156206	-4.125050509	9.31E-05	0.001182142	0.846603134
PPARGC1A	-0.434629919	1.7009906	-4.054418019	0.000119481	0.001459743	0.832659457
DYNC1LI1	0.152421946	5.299706318	4.165764889	8.05E-05	0.00104634	0.83153211
SLC38A6	0.188279566	4.524387464	4.151207248	8.48E-05	0.001093647	0.831089884
ADO	0.138286901	5.077587352	4.162026254	8.16E-05	0.001057667	0.82925067
GBA2	0.199451741	4.09546794	4.133451512	9.03E-05	0.001150266	0.827993299

Gene	Log fold change	Average Expression	t	P-value	Adjusted P-value	B
WBP11	0.110737031	5.637132537	4.164539397	8.09E-05	0.001050025	0.815507596
MSH6	0.197402833	5.917154835	4.162322772	8.15E-05	0.001057458	0.800363724
RNF123	-0.219767786	4.458751982	-4.141844563	8.77E-05	0.001123058	0.794479523
SAP130	-0.134719488	5.391350741	-4.152753252	8.43E-05	0.001088567	0.788932325
HIC1	0.271204858	5.6249989	4.154569052	8.38E-05	0.001082467	0.786537563
HNRNPK	0.098659093	8.373261462	4.147573736	8.59E-05	0.001106011	0.78531734
ANKRD42	-0.187302281	3.27466394	-4.104877396	1.00E-04	0.001252965	0.785249209
ACKR3	0.264523926	4.969560615	4.14456129	8.68E-05	0.001114138	0.771765021
PRELID3B	0.161368045	5.026968209	4.143371368	8.72E-05	0.001117918	0.767658093
YTHDC1	-0.15476611	5.058275286	-4.144720275	8.68E-05	0.001114138	0.765097317
HDHD3	0.296613872	2.276762491	4.034435811	0.000128175	0.001545895	0.753985647
ZBTB49	0.354602254	1.109591812	4.000067816	0.000144567	0.001700042	0.753180729
C22orf23	0.537006556	-0.0494505	3.966457918	0.000162532	0.001878608	0.753082525
RPTN	1.389936651	-4.033151148	4.053168068	0.000120008	0.001462816	0.752648842
WDR20	0.164002369	3.934189779	4.111937983	9.75E-05	0.001225046	0.745941408
GNPDA2	0.162600223	4.360247769	4.115337738	9.63E-05	0.001212381	0.740139966
RPUSD3	-0.287690318	3.567499787	-4.096374647	0.000103034	0.001284858	0.740095226
ALAS1	0.164469768	5.06290247	4.133975736	9.02E-05	0.001149094	0.736374034
INO80C	0.376572233	1.284913989	4.009999963	0.000139633	0.001653646	0.73283998
ATP6V0A1	-0.182631351	5.56764939	-4.135150824	8.98E-05	0.001146239	0.721800625
DGKQ	-0.193130796	3.948917271	-4.104484103	0.000100117	0.001253669	0.721681135
C1orf198	0.261452731	6.987827441	4.140044399	8.82E-05	0.001129319	0.720236911
RBM8A	0.128656466	4.631645554	4.11760909	9.56E-05	0.001206422	0.715199655
TDG	-0.246492999	2.785048788	-4.066305307	0.000114581	0.001410173	0.711377271
ZNF107	0.390141946	1.723255996	3.99267322	0.000148348	0.001735026	0.702263469
CBX3	0.141840076	6.754446907	4.13521305	8.98E-05	0.001146239	0.70005577
GATAD1	-0.171656783	4.702116658	-4.117432864	9.56E-05	0.001206422	0.696968143
LRIG2	0.336097698	3.033187719	4.059245845	0.000117467	0.001437065	0.692217183
DDX24	0.13410328	7.550753877	4.126188578	9.27E-05	0.001178364	0.682284997
NMT1	-0.166878592	6.888994636	-4.129159542	9.17E-05	0.001166971	0.681607112
CEP112	0.159290254	3.406949595	4.072700732	0.000112026	0.001383241	0.673457054
GRWD1	0.143698145	5.45606528	4.120654607	9.45E-05	0.00119671	0.672308676
TMC7	-0.526644682	-0.248817321	-3.932900177	0.000182596	0.002080653	0.663262676
ZNF8	0.398067377	1.886571448	3.994628615	0.000147339	0.001725905	0.65004661
ZNF589	-0.300688031	3.225688255	-4.048664518	0.000121924	0.001481222	0.647330739
SMARCE1	0.168589388	6.017742933	4.116513022	9.59E-05	0.001209355	0.640608874
N6AMT1	-0.266836855	2.686010582	-4.035233736	0.000127816	0.001542806	0.637711757
CCNF	0.400409585	2.641123585	4.002829156	0.000143179	0.001687671	0.614703909
AIFM2	0.180006554	4.673544803	4.090723324	0.000105115	0.001305406	0.612722079
PROB1	0.386698567	1.816631984	3.984278645	0.000152755	0.001782423	0.60739222
PLEKHA6	-0.321245072	2.86151841	-4.031926511	0.000129308	0.00155459	0.594053236
RBM4	0.275746223	1.632967201	3.975076705	0.000157731	0.001831968	0.59249215
NME6	-0.236373928	2.982731548	-4.026561041	0.000131765	0.001576574	0.585972121
ZNF496	-0.192318686	5.271470839	-4.092555607	0.000104436	0.001298042	0.585599725
FUS	0.213404999	7.407780241	4.099274177	0.000101982	0.001274901	0.584890896
UQCRC2	0.105718655	6.751268197	4.100983101	0.000101367	0.001268261	0.58377472
ZNF781	-0.424285611	-0.665360562	-3.913354944	0.000195354	0.002206487	0.583532969
ASNSD1	0.112249526	5.824365045	4.098189008	0.000102375	0.001278748	0.581076128
GTF2IRD1	-0.171735042	4.135950413	-4.062260453	0.000116226	0.001424601	0.581074803
WDR45B	0.123129429	6.261247444	4.097057248	0.000102786	0.001282819	0.572455836
MYLIP	-0.335695767	3.825252144	-4.053129896	0.000120024	0.001462816	0.570893786
CEBPB	0.360878821	6.510615381	4.095743581	0.000103265	0.001286666	0.568823776
TMEM68	-0.231458543	2.976632077	-4.018773475	0.00013541	0.001612508	0.568136404
C18orf25	0.239979257	2.948372535	4.022789658	0.000133518	0.001595021	0.565785697
SMAD3	-0.202534122	7.130400699	-4.093857915	0.000103956	0.00129421	0.56284928
RNF25	0.272538975	4.5286974	4.067751337	0.000113999	0.001405298	0.546301111
ARMC5	-0.224779934	3.825049993	-4.060163502	0.000117088	0.001433997	0.540808792
BORA	-0.555339104	0.701083216	-3.90323999	0.000202287	0.002274068	0.53144975
CTNS	-0.145291261	5.17499151	-4.076216311	0.000110644	0.001369552	0.527487379
MAPK8	0.173547315	3.963004281	4.047785186	0.000122302	0.001483855	0.525777442
ACER2	1.036003617	-1.493927051	3.893493518	0.00020919	0.002339452	0.523546499
PPIL4	0.152908557	4.805443642	4.064890526	0.000115154	0.001413758	0.522484751
UFSP1	0.36336799	0.697733699	3.908979228	0.000198325	0.002237874	0.517084915
C12orf65	0.214501888	3.794820134	4.028299955	0.000130964	0.001569483	0.510249968
CDC25A	0.399070128	0.880466049	3.920216156	0.000190781	0.002165728	0.50972216
RYBP	0.233029611	6.500205438	4.078547794	0.000109737	0.001360561	0.50873784
DTX3	-0.325241848	3.36981797	-4.033405931	0.000128639	0.001550251	0.504148826
SHOC2	0.186034073	6.195389656	4.077139759	0.000110284	0.001366217	0.503216404
RIPK1	0.26056912	5.697796634	4.073727158	0.000111621	0.001379371	0.502884966
ZNF302	-0.211481874	3.694286859	-4.02522349	0.000132384	0.001582728	0.494829397

Gene	Log fold change	Average Expression	t	P-value	Adjusted P-value	B
PRDM1	0.370635265	2.905438371	3.97778571	0.00015625	0.001817573	0.494447111
HSPA9	0.169638172	7.963854464	4.065041919	0.000115093	0.001413758	0.488394144
MDFI	-0.919969525	-1.067412876	-3.875273767	0.000222704	0.002470413	0.486768275
SP2	0.156762421	4.278478648	4.049301198	0.000121652	0.001481222	0.483713748
MYO18A	-0.302373758	5.548280822	-4.0666714	0.000114433	0.001409506	0.481833652
ACBD3	0.253616144	6.316487821	4.070277881	0.000112987	0.001393973	0.480535142
CEP97	-0.415262866	2.411152724	-3.95014219	0.000172008	0.001976449	0.47021093
FUBP1	0.17836626	4.945938243	4.047693928	0.000122341	0.001483855	0.470140009
ELOC	0.204357054	4.837466015	4.04696457	0.000122655	0.001486278	0.464749987
FIP1L1	-0.189316297	4.10237616	-4.031317509	0.000129585	0.001556673	0.455762792
GTF3C1	-0.207263641	6.791653748	-4.06259398	0.00011609	0.001424086	0.454914522
ZNF200	0.226174326	2.57223761	3.962332531	0.00016488	0.001903256	0.454887292
MED8	0.179355195	4.879836598	4.045956122	0.000123091	0.001488157	0.450318723
ZFP1	0.241930511	3.641875227	4.00069707	0.000144249	0.001697634	0.447572406
ABHD17C	0.262054765	4.770395607	4.046609762	0.000122808	0.001486278	0.447139582
UBA2	0.117574772	6.268977779	4.059095529	0.000117529	0.001437065	0.444280788
PDHX	0.199140927	3.900564038	4.016033428	0.000136716	0.001625485	0.444120687
ERCC6L	-0.55011009	0.655590292	-3.87697856	0.000221405	0.002457812	0.435172592
SRSF6	-0.209276138	6.611087388	-4.053372362	0.000119922	0.001462816	0.434864393
BUB3	0.121388493	5.913684245	4.048658315	0.000121927	0.001481222	0.414054115
PQLC3	0.140857874	5.046220158	4.036449022	0.000127272	0.001537469	0.412260969
RADIL	-0.35101614	1.702855776	-3.93488366	0.000181347	0.00207112	0.411460271
HOXA3	0.923448425	-1.167107374	3.85413113	0.000239434	0.002638507	0.409676842
CASS4	-0.718562284	-0.116655205	-3.881917514	0.000217683	0.002422323	0.404473274
ZNF570	0.257913966	2.759312047	3.946545583	0.000174167	0.001995168	0.400491013
A1BG-AS1	-0.358377377	1.004890103	-3.88185929	0.000217726	0.002422323	0.383451865
MLPH	-0.327125592	3.889289554	-4.003880283	0.000142654	0.001684118	0.374438466
C19orf54	-0.227283464	3.665688171	-3.992075126	0.000148658	0.001735955	0.371565613
UBLCP1	0.122303505	5.446547923	4.028545162	0.000130851	0.001569382	0.367847168
PRPS1	0.201499729	4.782255121	4.008619328	0.000140309	0.001659043	0.364868548
ZNF197	0.149280141	4.242434337	3.997245265	0.000145999	0.001712873	0.361026267
CCT6A	0.094029672	7.47940173	4.029818658	0.000130268	0.001563633	0.356042935
ITPKB	-0.326730498	5.139715522	-4.015768792	0.000136843	0.001625708	0.355758365
ZNF267	0.229385918	2.918769342	3.947604335	0.000173528	0.001990885	0.355423133
ATP6V1G1	0.188780715	6.297530766	4.032650361	0.00012898	0.001553125	0.355367617
HERC1	-0.382842575	5.352645226	-4.022105897	0.000133839	0.001597581	0.342882925
CCNL2	0.462053931	3.707936549	3.978823057	0.000155687	0.001812418	0.341838838
BLOC1S6	0.109012873	6.245480963	4.026947726	0.000131586	0.00157569	0.336892324
PPP1R26-AS1	-0.792741388	-1.961822702	-3.826348659	0.000263253	0.002855031	0.332385113
ZFX	-0.2881339	3.978591808	-3.992158822	0.000148614	0.001735955	0.327655359
YWHAZ	0.091830961	7.800624245	4.017439265	0.000136045	0.00161878	0.327445266
SNX18	-0.135215171	7.138421275	-4.018873138	0.000135363	0.001612508	0.322833559
SLC45A3	-0.832263762	-1.00566008	-3.820402027	0.000268637	0.002894671	0.32250728
FKBP15	-0.104781415	5.791505548	-4.02040434	0.000134639	0.001605861	0.321536126
TBX18	-0.291641454	5.079114307	-4.008967936	0.000140138	0.001658323	0.314416413
PCGF6	0.169218674	3.161304496	3.946903093	0.000173951	0.001994213	0.312246051
IPO13	-0.173560604	5.390031406	-4.010643165	0.00013932	0.001652528	0.311041087
NR3C2	-0.569708758	0.149523791	-3.840475787	0.000250872	0.002754467	0.304372243
HMGB1	0.117904443	5.538671305	4.008256097	0.000140488	0.001659849	0.29411341
DEGS1	0.112597001	6.850436318	4.01158864	0.000138859	0.001648368	0.283934515
CFLAR	-0.175601857	5.308854358	-4.003298465	0.000142944	0.001686225	0.280734853
CEP44	-0.280082833	2.032430252	-3.904114592	0.000201678	0.002268918	0.278363331
PGRMC2	0.110555781	7.7403914	4.002122772	0.000143533	0.001690519	0.273881942
RREB1	-0.286524401	4.55708321	-3.979224401	0.000155469	0.001811285	0.273644123
BIRC2	-0.132020383	5.093170556	-3.996069594	0.0001466	0.001718581	0.264195819
KBTBD3	-0.397756575	0.913111859	-3.831496833	0.000258675	0.002818163	0.264076314
RAB1A	0.107049876	7.590318183	3.998729158	0.000145244	0.001706678	0.259840862
NECAP1	0.190436679	3.99896151	3.968070077	0.000161624	0.00186997	0.259189132
ZC3H10	0.218079806	2.723872693	3.905196648	0.000200928	0.002262163	0.251337093
SZRD1	0.089300824	7.330369903	3.99778598	0.000145724	0.001710974	0.246862033
PACS1	-0.143842982	7.657991954	-3.993920189	0.000147704	0.001728834	0.246853928
WDR31	-0.468652724	2.020068549	-3.885489059	0.000215028	0.002397616	0.239754143
PTPN2	0.206180409	4.175902352	3.955419237	0.000168887	0.00194355	0.219222061
PTCD2	-0.204464434	3.562691802	-3.934267647	0.000181734	0.002073968	0.217215381
YAF2	0.198490761	3.825695567	3.942127741	0.000176854	0.002024413	0.214438276
AP4E1	-0.246892409	3.461251882	-3.936116756	0.000180574	0.002063863	0.208385575
COPS3	0.120763596	5.777447848	3.980846148	0.000154594	0.001802475	0.193709494
NRAV	-0.264733687	2.256918932	-3.873279791	0.000224233	0.00248554	0.191499948
LOC101927751	-0.281170725	1.771930318	-3.843983821	0.000247884	0.002723655	0.179343623
TMEM33	0.123928672	5.441532648	3.972598225	0.000159098	0.001844995	0.178036274

Gene	Log fold change	Average Expression	t	P-value	Adjusted P-value	B
NHLRC2	-0.219715059	3.491415516	-3.927798301	0.000185847	0.002112902	0.174996328
SYNCRIP	0.217584082	7.560640125	3.973196234	0.000158767	0.001842578	0.171711298
PDE4A	-0.15592307	5.300197869	-3.969331465	0.000160916	0.001863215	0.169105148
BCLAF1	0.144857073	6.371033063	3.975581573	0.000157454	0.001830162	0.165314625
SERPINE1	0.337376476	8.499264123	3.956071385	0.000168505	0.001940638	0.151020699
TLN2	-0.261170708	5.974625603	-3.969665	0.00016073	0.001862486	0.149101906
TUBGCP3	-0.236212181	3.934832066	-3.927066551	0.000186318	0.002116657	0.146211222
UBIAD1	0.145826473	3.913843371	3.92846817	0.000185417	0.002109606	0.143086422
POLR3F	0.194980811	3.145935946	3.899075945	0.000205209	0.002300053	0.142202687
TSC2	-0.138265393	6.742546647	-3.96630316	0.00016262	0.001878608	0.134652452
ELOA	0.211511558	5.499537878	3.960652026	0.000165845	0.001911472	0.131674931
STRAP	0.094939841	7.104749927	3.961888823	0.000165134	0.001904733	0.122737258
GDPD1	-0.672907703	-1.539302529	-3.750651674	0.000340183	0.003500356	0.120745868
EIF5A2	0.233711332	3.784739633	3.928906155	0.000185136	0.002108005	0.118666944
TBX15	-0.250267732	5.532146501	-3.951402211	0.000171257	0.00196933	0.112893401
MRPL50	0.159900026	3.916816211	3.917497408	0.00019258	0.002182867	0.109896763
CAMK2G	-0.153143081	4.859074044	-3.941477838	0.000177253	0.002027436	0.102026398
TAPT1	-0.18170052	3.457280306	-3.902997182	0.000202456	0.002274274	0.099831979
ZC3H14	-0.143963191	5.455031886	-3.948798884	0.000172811	0.001984166	0.098999538
IQSEC2	-0.160654597	3.699378763	-3.905369976	0.000200808	0.002262163	0.084855689
FAM220A	-0.14659148	4.490923474	-3.915330879	0.000194026	0.0021976	0.066487037
SLC5A6	-0.140232161	4.130653384	-3.913960078	0.000194946	0.002206362	0.066277944
ZNF761	0.241475734	2.969359885	3.85502691	0.000238702	0.00263236	0.055284334
PLEKHH3	-0.269491442	3.977505756	-3.90560842	0.000200643	0.002262163	0.054788027
ZNF830	0.19034085	3.70060427	3.89053565	0.000211329	0.002358124	0.042781973
ZBTB11	0.352503675	4.296085565	3.902115676	0.000203072	0.002279491	0.03736626
KHDRBS1	0.101145777	7.529479393	3.933147154	0.00018244	0.00208045	0.036934275
TGFBR3L	-0.672553004	-1.317975754	-3.71762097	0.000380093	0.003835041	0.025654522
DEPDC1	-0.477465228	1.154009176	-3.771063227	0.000317554	0.003296785	0.02242833
ZNF285	0.394303753	0.59023196	3.736002188	0.000357364	0.00364455	0.021269942
ARHGAP19	-0.342734184	1.8188705	-3.800190036	0.000287736	0.003054587	0.01386181
UPF2	-0.280942945	4.398463199	-3.894479652	0.000208482	0.00233326	0.006864371
FBXO38	0.25912153	4.555176949	3.894823739	0.000208235	0.002332231	0.004152276
SH3RF1	0.235572303	4.827775463	3.918224722	0.000192097	0.002179032	-0.003419996
NUDT15	0.132706701	4.03002796	3.882845802	0.00021699	0.0024177	-0.006540656
METTL4	-0.268809909	2.409535228	-3.834443583	0.000256089	0.002801538	-0.009063965
CASP8AP2	-0.315576776	3.146121476	-3.834074844	0.000256411	0.002802061	-0.017666843
LIMK2	-0.31983246	2.994397414	-3.830024382	0.000259976	0.002829683	-0.018562583
IL16	-0.410403862	2.26459542	-3.804627478	0.000283435	0.003024778	-0.02197732
PPM1F	-0.132503108	5.294571103	-3.913291256	0.000195397	0.002206487	-0.023009387
MBD4	0.199264173	4.572653923	3.892600604	0.000209834	0.00234462	-0.026030582
ANGEL2	-0.206932453	4.008376412	-3.880091671	0.000219052	0.002435274	-0.026772185
RTL3	0.850848488	-1.789153179	3.705804895	0.000395424	0.003963184	-0.029546155
MTERF1	-0.238621059	2.364149649	-3.802665455	0.000285329	0.003035452	-0.034876355
FPGT	-0.247421654	2.509175138	-3.80996095	0.000278347	0.002982231	-0.036315519
GIMAP2	-0.319155044	1.522767657	-3.770015476	0.00031868	0.003306193	-0.037299564
SCAP	-0.121065936	6.47084008	-3.913403679	0.000195321	0.002206487	-0.040914227
RASSF9	-0.751414634	0.232516365	-3.731918202	0.000362299	0.003685126	-0.043486079
CISH	-0.423588708	1.286193563	-3.761183101	0.000328322	0.003389887	-0.048303174
USPL1	0.332481864	3.324164627	3.831670463	0.000258522	0.002818163	-0.056949451
CYYR1-AS1	-0.895102158	-2.384247535	-3.696808353	0.00040749	0.004062494	-0.060710742
NOL4L	0.260054507	3.283901799	3.817383181	0.000271411	0.002914135	-0.073323828
EFCAB7	-0.29935502	2.478421091	-3.809736537	0.000278559	0.002982384	-0.073643643
GINM1	0.088959187	6.495492765	3.901877088	0.000203239	0.002279667	-0.078466221
MTOR	-0.185954371	5.343318742	-3.892420817	0.000209964	0.00234462	-0.079315858
MON1A	-0.219441722	3.87618841	-3.853127786	0.000240257	0.002645642	-0.086247559
HMBOX1	-0.330499192	1.733916959	-3.769085652	0.000319682	0.003314309	-0.092804412
ZNF691	0.317131901	1.942852402	3.744390637	0.000347427	0.003556142	-0.094900582
ZNF135	-0.242156988	2.199776004	-3.785887725	0.000302027	0.003182685	-0.095830426
ZNF213	-0.202850947	4.585731165	-3.868378537	0.000228034	0.002523953	-0.098318567
INCENP	-0.361329421	3.345591548	-3.818266267	0.000270597	0.002909541	-0.102753663
UBE3D	-0.210154169	1.504822569	-3.748873414	0.000342226	0.003516571	-0.105822759
DDX5	0.283170611	8.4589716	3.871416271	0.000225671	0.002499638	-0.106382352
SPOUT1	-0.141452638	3.969904111	-3.851597251	0.000241518	0.002657583	-0.119365522
PLK1	-0.419813366	2.51621726	-3.781940961	0.000306089	0.003215426	-0.121909695
TDP1	-0.210639007	2.973059945	-3.804119532	0.000283924	0.003024778	-0.124001838
SLC25A29	-0.27296289	3.189613988	-3.836070313	0.000254672	0.002792119	-0.125603392
MPP2	-0.333858916	1.533555205	-3.723579968	0.000372577	0.003769313	-0.134183576
HNRNPA0	0.085899599	7.626674593	3.878048947	0.000220593	0.002450604	-0.137171209
FAM171A2	-0.211244575	3.426736673	-3.820963253	0.000268125	0.002891216	-0.151114367

Gene	Log fold change	Average Expression	t	P-value	Adjusted P-value	B
DCAF13	0.163364079	5.212285439	3.862804771	0.000232431	0.002568843	-0.158223252
RGS17	-0.356327733	2.180347559	-3.75069887	0.000340129	0.003500356	-0.159310785
RHBDF2	-0.26833978	2.078017259	-3.749930585	0.00034101	0.00350647	-0.169394943
FAM76A	0.279121302	3.280498601	3.799178216	0.000288726	0.003062932	-0.189571699
MEDAG	0.16906175	7.366190119	3.865100609	0.00023061	0.002550591	-0.189940279
CCDC184	-0.613697874	-0.070633309	-3.6529624	0.000471486	0.004585193	-0.191495171
TCEAL1	0.211445372	3.932958184	3.825050886	0.000264419	0.002861499	-0.197097363
LOC100507437	-0.295917368	1.334951749	-3.714758554	0.000383754	0.003866801	-0.199096001
AP4M1	-0.172980325	4.05444503	-3.827871125	0.000261891	0.002844357	-0.199758861
SHANK1	-0.33592839	1.732844128	-3.72276428	0.000373598	0.003774565	-0.200739372
EVI5	0.241739166	3.507121191	3.807565909	0.000280621	0.003000194	-0.204035728
PDPK1	-0.197825687	3.444171589	-3.803818276	0.000284215	0.003025733	-0.204381006
SDHAF4	-0.275280669	1.40331821	-3.711760663	0.000387624	0.003895378	-0.205778659
PSRC1	-0.369254623	1.280042803	-3.683739959	0.000425643	0.004207277	-0.209432551
ESF1	0.198617574	4.342996176	3.833963253	0.000256508	0.002802061	-0.21074803
MLYCD	-0.194782326	2.690383319	-3.755595939	0.000334565	0.003449612	-0.215526982
CARM1	-0.11190802	6.667150074	-3.859457658	0.00023511	0.002596549	-0.216715942
PDXDC1	-0.167984199	6.10472065	-3.856206206	0.000237741	0.002623681	-0.218011345
CLUAP1	-0.132795718	4.459714704	-3.8348481	0.000255735	0.002801443	-0.219750482
ZNF540	0.517354739	-0.293585751	3.634521959	0.000501163	0.004833237	-0.223063928
GPATCH4	0.196106699	4.540608158	3.831433183	0.000258731	0.002818163	-0.225177054
LINC01730	-1.028478599	-2.914403468	-3.649989715	0.000476154	0.004624621	-0.22964855
GPCPD1	-0.268794948	3.889916878	-3.82594935	0.000263611	0.00285686	-0.230721248
ZNF222	0.273577822	1.28902492	3.685815894	0.000422709	0.00418751	-0.239955522
ZNF175	-0.265663788	3.186162105	-3.768258764	0.000320576	0.003321289	-0.242647925
LPAR6	-0.32705267	2.225548412	-3.734925093	0.000358659	0.003650569	-0.245843215
CCDC96	0.392298775	0.65396698	3.646853359	0.000481127	0.004660901	-0.246904056
TNFAIP8	0.236021845	4.474333226	3.822586452	0.000266647	0.002877344	-0.249757678
TMEM165	0.127214198	5.92977197	3.845743031	0.000246399	0.002709314	-0.250962719
AKAP8L	-0.221906024	4.601314544	-3.818708976	0.000270189	0.002907237	-0.262486694
PHF14	-0.145816214	4.745889535	-3.824463943	0.000264948	0.002863111	-0.268097514
SPEG	-0.267887041	3.758287511	-3.796341446	0.000291517	0.003088194	-0.268632202
FERMT2	0.245274498	7.023179374	3.840212697	0.000251097	0.002754934	-0.272172306
ZNHIT6	0.166200669	5.00331552	3.826581712	0.000263044	0.002854821	-0.279629724
SPAG16	0.189118428	3.205763733	3.77595894	0.000312344	0.003265199	-0.281243601
TNFRSF1B	0.386462146	4.680945544	3.802376359	0.000285609	0.003036287	-0.290502055
CERS5	0.109954838	5.292585804	3.825121669	0.000264355	0.002861499	-0.293286341
CDC42	0.123964348	7.037468203	3.834666524	0.000255894	0.002801443	-0.293487465
ZNF550	0.497587947	0.584740516	3.632943934	0.000503783	0.004846099	-0.30129265
TMCO1	0.110348859	6.341437171	3.832851812	0.000257482	0.002810663	-0.302065053
LINC00654	-0.563041573	2.361113941	-3.707388421	0.000393336	0.003947507	-0.30512627
DROSHA	-0.144718217	5.288969464	-3.824541906	0.000264878	0.002863111	-0.306495459
PEX13	-0.173087471	3.853787195	-3.784912351	0.000303026	0.0031877	-0.308897822
FEM1B	0.202966859	6.501327436	3.829764299	0.000260207	0.002830147	-0.31278959
TBPL1	0.140872784	3.625621181	3.772625535	0.000315883	0.003290771	-0.314105915
MOAP1	0.187034531	3.319403167	3.772199654	0.000316337	0.003290957	-0.31452386
NYNRIN	-0.35093686	6.251609185	-3.827987047	0.000261787	0.002844357	-0.316473904
PRDM4	0.18571847	5.248625318	3.818724555	0.000270175	0.002907237	-0.32213399
UTP15	0.171619321	3.664211041	3.777006946	0.000311239	0.003258173	-0.328004664
TRIM62	0.210186023	2.484961514	3.722473238	0.000373962	0.003775717	-0.328446922
EIF2A	0.129850097	6.832751595	3.824201932	0.000265184	0.002863612	-0.32894892
NFATC4	-0.168436086	7.401243121	-3.818040535	0.000270804	0.0029097	-0.333432134
CASP8	-0.231839294	3.26070826	-3.764564102	0.000324599	0.003356043	-0.335217022
THNSL1	-0.284924212	2.300010293	-3.686224492	0.000422134	0.004186322	-0.341696074
RAB3D	-0.287066053	2.009851896	-3.677679904	0.00043432	0.004276209	-0.341724148
OSBPL5	-0.162119184	5.922964281	-3.815625617	0.000273038	0.00292952	-0.350362478
MUS81	-0.185614286	3.853137396	-3.77524173	0.000313102	0.003268587	-0.351541105
PDP2	0.441407108	3.563233644	3.759636379	0.000330039	0.003405281	-0.353865003
LMF1-AS1	1.066586083	-1.941958955	3.599584141	0.000562324	0.005327575	-0.355465191
POP1	0.36075831	3.291312245	3.735327812	0.000358174	0.003648099	-0.365657487
ZRANB2	-0.179055178	4.78909718	-3.797291315	0.000290579	0.003080428	-0.366566556
EIF2S1	0.104730059	6.274098243	3.810621411	0.000277723	0.002977663	-0.372365413
FTH1	-0.278177067	9.6883212	-3.771197675	0.00031741	0.003296785	-0.373439342
UTP3	0.139194076	5.568817662	3.807086344	0.000281078	0.003002953	-0.374886172
RBBP8	0.189273194	4.704956907	3.788559788	0.000299307	0.003161822	-0.375120215
ATMIN	0.116474886	6.299980029	3.809300782	0.000278972	0.002984682	-0.377147863
ZNF19	-0.343277245	0.976767761	-3.618841544	0.000527787	0.005040924	-0.38187246
PCDH18	-0.310208145	8.785863844	-3.785080924	0.000302853	0.0031877	-0.384470119
COASY	-0.18470004	5.563466061	-3.801420019	0.000286538	0.00304401	-0.388610443
UBR4	-0.352642533	7.160532738	-3.804335015	0.000283717	0.003024778	-0.388747454

Gene	Log fold change	Average Expression	t	P-value	Adjusted P-value	B
EID1	0.12047026	8.43620879	3.788584889	0.000299281	0.003161822	-0.390278862
ATP5S	0.178648301	3.133947629	3.724196706	0.000371808	0.003766586	-0.392350005
ATG9A	-0.121469514	6.66005752	-3.804270319	0.000283779	0.003024778	-0.394925679
SAT1	0.196182245	4.677453332	3.777386281	0.000310841	0.00325626	-0.402245725
NCBP3	-0.169573178	4.854833033	-3.78856108	0.000299306	0.003161822	-0.4031786
FRMD8	0.21441221	5.402060639	3.786834746	0.00030106	0.003175892	-0.403189436
MED28	0.114352018	5.153477237	3.785790321	0.000302127	0.003182685	-0.40650394
GNL3	0.218955327	5.852258361	3.794733141	0.000293111	0.003102897	-0.410859562
PAX9	0.847734106	-0.896166936	3.565457921	0.000628858	0.005862081	-0.411471126
FMNL3	-0.15260954	5.019458685	-3.780067468	0.000308035	0.003231363	-0.411611974
FAM83D	-0.407698209	1.944522417	-3.66211752	0.000457381	0.00447111	-0.412099354
MTG2	0.190644998	3.618546554	3.744132337	0.000347729	0.003556142	-0.413126911
KIF3A	-0.227256462	2.517266485	-3.701322474	0.000401393	0.004009978	-0.41732418
CEP41	-0.161368389	3.512522471	-3.746788904	0.000344635	0.003534097	-0.418375428
MB21D2	0.213518128	2.723524154	3.723722779	0.000372399	0.003769313	-0.426155661
TYK2	-0.113980076	5.418678453	-3.78769837	0.000300181	0.003168837	-0.426252153
NEURL4	-0.24630109	2.746374945	-3.680529133	0.00043022	0.004241382	-0.426944709
KCNE3	-0.408895319	0.588583217	-3.589433202	0.000581382	0.005471531	-0.433574649
WDR24	-0.253256545	3.314288337	-3.727034772	0.000368285	0.003738445	-0.43395748
ZNF367	0.429106336	2.277310962	3.686692999	0.000421475	0.004182543	-0.435839659
CCDC122	-0.312275979	1.005425036	-3.628910483	0.00051054	0.004898586	-0.437453683
MAT2B	-0.115124251	5.557644376	-3.782830351	0.000305169	0.003208001	-0.43983758
CKAP2L	-0.443165678	1.948047499	-3.648546213	0.000478437	0.004637821	-0.444006441
RNF149	0.139408115	5.195629643	3.775626016	0.000312696	0.003266608	-0.44763086
SUSD6	0.234737396	4.48969887	3.764632272	0.000324524	0.003356043	-0.451032535
LIG4	-0.307301376	2.907945246	-3.715677172	0.000382575	0.003857506	-0.457044087
FBXO42	-0.162478187	4.369333738	-3.748328453	0.000342854	0.003520626	-0.464598819
LOC100287015	-0.400579678	0.229358543	-3.578429116	0.000602734	0.005647308	-0.464784362
SAMD4A	0.293821638	5.653654879	3.776595901	0.000311672	0.003260438	-0.471037124
CAPZA2	0.099802878	6.722896675	3.7801425	0.000307957	0.003231363	-0.472396107
LARP4B	0.219873041	5.082449967	3.764228622	0.000324966	0.00335754	-0.475574002
DIRAS1	0.185459175	5.900995055	3.772324891	0.000316204	0.003290957	-0.479007184
GMPS	0.116307894	6.378701753	3.777500129	0.000310721	0.00325626	-0.479196189
ZFAND5	0.16851542	7.200329575	3.771962331	0.000316591	0.003291324	-0.482964967
ZXDA	-0.380812716	1.458473694	-3.624676922	0.000517725	0.004958052	-0.488120548
HNRNPAB	0.154142695	6.946159884	3.773424552	0.000315031	0.003284566	-0.492421562
MED16	-0.154353252	6.164177486	-3.773388841	0.000315069	0.003284566	-0.49280418
GZF1	-0.188134662	4.086421595	-3.73581894	0.000357584	0.00364455	-0.497024323
VAV3	0.609702976	0.19115105	3.548744763	0.000664105	0.006133707	-0.502532819
BAZ2B	-0.332628489	3.533817478	-3.725354836	0.000370367	0.003757039	-0.509392058
TNFRSF10B	0.209355002	6.324581296	3.767133562	0.000321796	0.003331638	-0.513827833
RUNX2	-0.335898558	2.692656264	-3.672996254	0.000441141	0.004337698	-0.514270435
PELI3	-0.187445173	4.250853172	-3.724808875	0.000371045	0.003761391	-0.525922289
AAED1	0.183341235	4.805347277	3.747992638	0.000343242	0.003522207	-0.527934786
KIAA1586	0.177698323	3.400579002	3.704192579	0.000397561	0.003979308	-0.52796596
CCNE1	0.342919998	1.649013882	3.589591371	0.00058108	0.005471531	-0.541966206
CKAP2	-0.281528206	4.35669508	-3.723261096	0.000372976	0.003770812	-0.541980816
HDAC11	-0.177219915	3.684941353	-3.705600564	0.000395694	0.003963256	-0.543295012
XIRP1	0.799050554	-0.855075934	3.519275032	0.000730859	0.006632329	-0.548758807
ISCA1	0.184300254	3.962800401	3.712379908	0.000386822	0.003889908	-0.554026835
STK38	-0.178894863	6.380604827	-3.753092363	0.000337399	0.003476452	-0.558666135
VASH1	-0.326576306	3.348798551	-3.67661249	0.000435866	0.004288624	-0.560274545
ELAC1	-0.251021014	2.007886057	-3.621365481	0.000523412	0.005002971	-0.560963928
HNRNPF	0.083932855	7.818134759	3.744204393	0.000347645	0.003556142	-0.561394637
TCOF1	-0.139592563	5.330968238	-3.740086917	0.000352492	0.003602401	-0.568327801
HECTD3	-0.190605029	5.712393707	-3.746011203	0.000345538	0.003540947	-0.570333214
DUSP19	-0.419963335	0.057430373	-3.531555226	0.000702305	0.006431317	-0.584663157
ZNF844	0.34049315	3.017300864	3.630121194	0.000508503	0.004884021	-0.593727797
CEP19	-0.238373208	2.70982281	-3.648593433	0.000478362	0.004637821	-0.595239077
FAM135A	-0.326633952	2.893839154	-3.648961606	0.000477779	0.004637412	-0.595536894
STEAP1	0.176109009	5.151362386	3.727900725	0.000367217	0.003730114	-0.600449358
ACTL10	0.481501428	0.555872042	3.52862767	0.000709014	0.006473087	-0.60291389
HMGA2	0.328247537	4.712937559	3.713142112	0.000385836	0.003883373	-0.603860807
CUL3	0.138815823	6.104881029	3.737557301	0.000355502	0.003630695	-0.60489239
GMNN	0.244930467	2.78491668	3.637244228	0.000496673	0.004796075	-0.605648955
MEF2D	-0.171914632	6.216844812	-3.737201079	0.000355927	0.003632581	-0.608473518
NLRX1	-0.162503032	4.660294321	-3.713081763	0.000385914	0.003883373	-0.612164034
KCTD18	-0.128315689	4.381883283	-3.706512647	0.000394489	0.003956449	-0.617041649
DHX30	0.12229914	5.803751738	3.731153824	0.00036323	0.003692102	-0.618817708
C17orf49	0.764604093	-2.360228619	3.496080177	0.000787818	0.007064105	-0.619339201

Gene	Log fold change	Average Expression	t	P-value	Adjusted P-value	B
GSDMC	1.086252037	-2.620711979	3.510817569	0.000751161	0.006783865	-0.622954526
ZNF778	0.328845611	1.995276026	3.598290614	0.000564719	0.005343546	-0.62460599
SNN	0.238542723	4.604633079	3.700691946	0.000402239	0.0040128	-0.627384712
STK35	0.154621291	4.38443772	3.700859252	0.000402014	0.0040128	-0.632328055
PLD1	-0.227013266	3.943127277	-3.692280882	0.000413694	0.004116173	-0.633498136
CENPC	0.201256661	3.020053265	3.660768513	0.000459433	0.004488265	-0.635400381
KIAA0513	-0.248135922	4.716627643	-3.701647783	0.000400956	0.004009978	-0.642563547
ORC4	-0.143764143	3.984242962	-3.685744545	0.00042281	0.00418751	-0.643316793
KCNMB2	1.055773047	-3.806498254	3.528994154	0.000708171	0.006469309	-0.643857591
SOBP	-0.280226937	3.895379749	-3.670739305	0.000444465	0.004364678	-0.646786195
POLM	-0.178531206	3.084189166	-3.642613288	0.000487929	0.004717689	-0.666694026
METTL3	-0.23462878	3.394746689	-3.660380994	0.000460025	0.00449113	-0.667263109
NANOS1	0.340696659	1.056963896	3.536476059	0.000691162	0.006344704	-0.667595713
TIGD3	0.749091409	-2.532390607	3.473641183	0.000846899	0.007464911	-0.672360852
TUBB4B	0.157290233	7.717777536	3.709204784	0.000390953	0.003926213	-0.677358289
ZNF865	-0.191694017	4.565038375	-3.690267639	0.000416481	0.004135713	-0.681070777
C20orf24	-0.302508369	2.143175913	-3.588742555	0.000582701	0.005478067	-0.682668945
CD58	0.24005558	2.784277552	3.617007958	0.000530986	0.005059314	-0.682950782
CLK1	-0.433864109	3.847084894	-3.685510541	0.000423139	0.004188024	-0.683942679
TMEM41A	0.19013422	4.098393238	3.671581066	0.000443222	0.004355318	-0.692759135
LENG8	0.316436081	4.98386071	3.693135972	0.000412515	0.004109875	-0.698441416
PPP1R18	0.150029353	7.447912329	3.701299186	0.000401424	0.004009978	-0.702815202
CYTH1	-0.199196539	3.982642941	-3.664145126	0.000454311	0.004446879	-0.703919244
PRRT3	-0.195739978	3.141446977	-3.632565948	0.000504413	0.004849059	-0.710887791
JADE2	-0.253007853	5.012797156	-3.681288358	0.000429133	0.004233442	-0.717060334
DOLPP1	-0.175348416	3.067049769	-3.625626476	0.000516106	0.004945683	-0.717455655
TMEM102	-0.491758262	-0.124321669	-3.466811251	0.000865698	0.007590731	-0.722437405
FAM206A	-0.167600448	4.070189316	-3.666085481	0.000451393	0.004426939	-0.722909627
DDX56	0.154233714	5.666117214	3.692717895	0.000413091	0.004112892	-0.73334337
TROAP	-0.505403137	0.365299328	-3.479363577	0.000831447	0.007358937	-0.737948365
NAXD	-0.131629906	4.455908544	-3.665847898	0.000451749	0.004427552	-0.740591811
SCOC	0.105323868	5.734836411	3.6910046	0.000415459	0.004128282	-0.743470879
AAK1	-0.182995851	5.207841955	-3.682378026	0.000427579	0.00422087	-0.74548064
NFX1	0.198715389	5.489925388	3.684649112	0.000424356	0.004197306	-0.748924185
ZNF146	-0.131230085	6.125853466	-3.691417619	0.000414887	0.004125319	-0.749880977
ALG10B	-0.35016712	1.2437188	-3.523297695	0.000721387	0.006558219	-0.753059671
PBX2	-0.167449916	4.079631485	-3.655089475	0.000468172	0.004561803	-0.754832617
PPARGC1B	1.078404593	-0.028967557	3.437706234	0.000950319	0.008152391	-0.767589185
FRS3	0.376804141	0.837418872	3.481501157	0.000825743	0.007321361	-0.772580308
PIGV	-0.141196622	3.860513859	-3.64383527	0.000485959	0.004704686	-0.778834825
CCDC71L	-0.206207567	5.848646176	-3.683134117	0.000426503	0.004213013	-0.780287369
CRIPAK	-0.262791065	1.610560491	-3.541070368	0.00068091	0.006277369	-0.780813676
NKX6-1	1.27454655	-3.175666375	3.45664152	0.000894422	0.007790909	-0.791804209
TRPC4AP	-0.08705982	6.708240617	-3.679074678	0.000432308	0.004259184	-0.792606943
ZC3H18	-0.127982057	5.702953738	-3.667324533	0.000449538	0.004411623	-0.81240509
TCEANC	-0.314239575	1.088634756	-3.481031286	0.000826994	0.007328136	-0.814972356
DBT	-0.257047447	4.169513683	-3.630005096	0.000508698	0.004884021	-0.815885374
YPEL4	-0.419650728	0.818629285	-3.472064256	0.000851205	0.007494186	-0.82363052
GUCA1B	0.763682692	-2.404410582	3.426116309	0.000986143	0.008402292	-0.82546195
TMEM99	-0.210996027	3.055567953	-3.5924907	0.000575578	0.005432648	-0.826706989
C19orf47	-0.178466887	3.132017227	-3.590262963	0.000579801	0.005465662	-0.829093749
SMG5	-0.134180208	6.349584286	-3.665536362	0.000452217	0.004429255	-0.834209187
E4F1	0.216178449	3.94422207	3.615329247	0.000533932	0.005084163	-0.835746203
HK2	0.300739672	5.150617754	3.654127676	0.000469668	0.004573202	-0.836586159
LOC100507487	-1.062248581	-2.74999914	-3.428674582	0.000978127	0.008338713	-0.837116517
UGDH	0.255979456	6.882504833	3.663765135	0.000454885	0.004449603	-0.840149195
IRF2	-0.210278097	4.386041064	-3.618664617	0.000528094	0.005040924	-0.841916575
CNTROB	-0.124024864	4.247171677	-3.624056502	0.000518786	0.004965056	-0.848098035
OGFOD1	0.083286127	5.492906174	3.651988679	0.00047301	0.004597049	-0.848331878
RRAGC	0.112739557	5.70905231	3.655902234	0.000466912	0.004552467	-0.850815571
METTL9	0.099312607	7.60645305	3.653946957	0.000469949	0.004573202	-0.852583354
PIM2	-0.322511606	0.23449648	-3.437261476	0.00095167	0.008159341	-0.856833602
SSB	0.113876273	6.178630201	3.65672454	0.00046564	0.004543008	-0.858984605
PSMA3-AS1	-0.218605264	3.384152676	-3.598762862	0.000563843	0.005338614	-0.862887435
RDH10	0.367171495	5.265234993	3.643620088	0.000486306	0.004705014	-0.863604957
RRAGB	0.119424859	4.177897338	3.618159774	0.000528974	0.005043333	-0.867990564
CHST11	-0.249994338	4.821501503	-3.633434505	0.000502967	0.004844436	-0.871986466
NGDN	0.200991055	3.945085642	3.61375327	0.000536711	0.005104175	-0.875263486
CEP89	-0.105662309	4.945028248	-3.633859958	0.000502261	0.004840725	-0.8901607
RNF13	0.132009746	5.3361542	3.635922467	0.000498848	0.004813995	-0.898926986

Gene	Log fold change	Average Expression	t	P-value	Adjusted P-value	B
ZNRD1ASP	-0.401129753	0.092261738	-3.423535877	0.00099429	0.008457362	-0.901449877
PIWIL4	0.260881492	0.880955881	3.451572894	0.000909072	0.007870599	-0.907457472
OAT	0.114797531	6.839568747	3.641979474	0.000488953	0.004724563	-0.908657043
SYNGAP1	-0.206274622	4.851559453	-3.623568393	0.000519623	0.004969901	-0.91176011
FAM43B	0.614933576	0.57853369	3.408869014	0.001041821	0.008759724	-0.913534193
PDSS1	0.277590978	0.98684054	3.448681515	0.00091753	0.007925273	-0.914812206
TMEM186	0.244127466	2.006473378	3.49584325	0.000788421	0.007065306	-0.916014983
SLC7A6OS	-0.227079636	1.925921067	-3.500163001	0.000777497	0.006979864	-0.916101409
HSPA1L	-0.376707595	1.072949412	-3.452849418	0.000905361	0.007857738	-0.916200801
LGALSL	0.318931886	1.11872247	3.454496583	0.000900594	0.00783288	-0.922689942
SSR4P1	-0.285990014	0.804414384	-3.453926628	0.000902241	0.007838153	-0.931208895
SLC30A7	0.287838965	4.844415108	3.612482516	0.000538962	0.005122349	-0.933420075
DESI2	0.113543426	6.288614033	3.63318232	0.000503387	0.004845377	-0.93484706
CSPP1	-0.314532797	2.465144389	-3.511842547	0.000748672	0.006765447	-0.939188302
LRCH4	-0.243091457	1.902587643	-3.474802449	0.000843742	0.007454617	-0.943709064
BMP2K	0.290823678	4.45940182	3.594497637	0.000571798	0.005400356	-0.947051994
LARP6	-0.187267379	6.023138944	-3.627505412	0.000512914	0.004918232	-0.949121938
ZNF684	0.282113938	0.944421946	3.424090878	0.000992533	0.008451956	-0.949914714
MICB	0.205083008	3.198606848	3.551934661	0.000657236	0.006085192	-0.950867001
PLEKHM3	-0.765173263	1.354540286	-3.456542698	0.000894705	0.007790909	-0.951294415
FCHSD1	-0.152472886	3.354522758	-3.563545569	0.0006328	0.005895173	-0.951616784
INTS12	-0.186456884	3.302353584	-3.562877278	0.000634182	0.005900756	-0.95379171
LOC339803	-0.213782501	2.880300681	-3.537503483	0.000688856	0.006331258	-0.955558901
CA13	0.411455875	-0.074054749	3.402021805	0.001064737	0.008915619	-0.956987707
HSPA4L	0.282787271	3.94770276	3.578385862	0.000602819	0.005647308	-0.95908913
MKI67	-0.460409792	4.849061925	-3.601196377	0.000559352	0.005306093	-0.966795245
SLC10A7	-0.316484636	2.498672128	-3.512522334	0.000747026	0.006754624	-0.969002434
RNPS1	0.186332723	4.740413378	3.600461991	0.000560704	0.00531557	-0.969192925
AP3D1	-0.081104891	7.646589936	-3.614957211	0.000534586	0.005087183	-0.97319315
SPHK2	-0.163193635	3.92285909	-3.574176829	0.000611183	0.005711449	-0.980194414
TRMT6	0.15086646	3.625830464	3.570338523	0.000618905	0.005772872	-0.980840284
SIMC1	-0.170843182	2.980542985	-3.516032137	0.000738582	0.006690314	-0.98132684
CAD	-0.215043715	6.645161738	-3.618496742	0.000528387	0.005040924	-0.981941337
THUMPD2	0.20139434	2.230181835	3.491692819	0.000799054	0.007147835	-0.983186467
SLF2	0.363645894	4.12250508	3.555066257	0.000650559	0.006027071	-0.987613539
LMCD1-AS1	0.550308058	-1.909489508	3.354483567	0.001237437	0.01010887	-0.98995841
LRRC75A	-0.344848271	0.680528764	-3.400283947	0.001070628	0.008954995	-0.990363581
TXLNA	0.183243186	6.442660258	3.611406405	0.000540875	0.005137291	-1.004109731
LDLRAP1	-0.151995419	4.813476688	-3.591609291	0.000577245	0.005444976	-1.007959539
ARFIP2	0.156197741	5.647709604	3.602581372	0.000556811	0.005285317	-1.009320323
RAET1E-AS1	-0.79847162	-1.126627725	-3.347748727	0.001263938	0.010297438	-1.014197068
RAD18	-0.196560281	3.169758225	-3.536517013	0.00069107	0.006344704	-1.018874528
FXR2	0.187055768	4.713731346	3.581576229	0.000596553	0.005599046	-1.018875368
ZNF439	-0.290214338	1.203985104	-3.423141786	0.00099554	0.008463215	-1.020143867
FAM222B	0.157436252	4.066181935	3.557893452	0.000644586	0.005979087	-1.021437715
SPECC1L	-0.100625125	4.939726071	-3.588619704	0.000582936	0.005478067	-1.023765749
RRAS2	0.185714236	5.5924253	3.596933275	0.000567243	0.005360692	-1.025581442
BRD1	0.162640029	4.58020464	3.579424862	0.000600772	0.005635134	-1.026782556
TSPY26P	-0.225129388	2.697019713	-3.489999099	0.000803432	0.007174218	-1.030690619
NSD1	-0.289432449	6.039946602	-3.597067729	0.000566992	0.005360692	-1.037651579
GRPEL1	0.146467199	4.72489272	3.577631916	0.000604309	0.005654234	-1.049620876
RAD52	-0.427916318	1.081085652	-3.405080366	0.001054442	0.008849093	-1.052548635
SERPINB1	0.176483377	4.824533827	3.575037248	0.000609464	0.005698925	-1.056024015
ZNF862	-0.192319071	2.54370972	-3.479482395	0.000831129	0.007358937	-1.057052264
ING3	-0.196815172	3.296731032	-3.533194251	0.000698574	0.006401049	-1.060614304
C11orf96	0.492000077	2.907265129	3.504008208	0.000767893	0.006897758	-1.063353751
TSC22D1	-0.158182603	6.756704903	-3.589364212	0.000581514	0.005471531	-1.071658471
VRK1	0.206590032	2.639781926	3.485549791	0.000815042	0.007252089	-1.071953363
CDKL5	-0.413617625	2.079311293	-3.470782465	0.00085472	0.007520738	-1.076621821
BLZF1	0.152854087	4.392075497	3.561320148	0.000637416	0.005923521	-1.078836126
PON2	0.126755318	4.188794262	3.547559941	0.000666673	0.006149888	-1.080460691
TEX10	0.249799977	5.196076443	3.571073451	0.000617419	0.005762581	-1.084329731
COMMD10	0.153341764	3.913162469	3.538628284	0.000686341	0.00631199	-1.085993372
ZFHX3	-0.273063494	4.626410919	-3.55855728	0.000643191	0.005969824	-1.089850696
FLJ31104	0.876851771	-2.937266503	3.319891709	0.00137933	0.011123249	-1.09094755
UBXN7	-0.368717623	5.592430704	-3.572246145	0.000615056	0.005744078	-1.091069288
SEPT7	0.080289296	7.540776204	3.577814929	0.000603947	0.005654234	-1.091965014
ARF6	0.150781071	6.576744563	3.581600144	0.000596506	0.005599046	-1.094414225
NECAB3	-0.197643732	3.046172099	-3.504331013	0.000767092	0.006894673	-1.098731774
INSIG1	0.333217563	8.714058717	3.56081973	0.000638458	0.00592955	-1.108170423

Gene	Log fold change	Average Expression	t	P-value	Adjusted P-value	B
ZNF341	-0.233048905	1.667850405	-3.415662602	0.001019545	0.00862338	-1.108343097
ACBD5	-0.259664121	4.073175166	-3.529608443	0.00070676	0.006464256	-1.115325593
ZMYM1	-0.35293998	2.544237297	-3.449014303	0.000916553	0.007921374	-1.119157494
ZNF672	0.134066391	4.351264027	3.540315975	0.000682583	0.006286852	-1.120885864
ZNF30	0.335170162	1.12247296	3.363148318	0.001204112	0.009884871	-1.124682609
ZFP90	-0.219666305	4.836887464	-3.547804668	0.000666142	0.006148753	-1.126697648
UBOX5	0.190623818	3.005105491	3.476641588	0.000838763	0.007414984	-1.132840988
FAM196A	0.540809137	0.187746065	3.316819651	0.001392645	0.011206627	-1.142046935
SIPA1L3	-0.217309533	4.251695845	-3.526869562	0.000713073	0.006499355	-1.143294396
JUND	0.25218986	7.55623253	3.556187458	0.000648184	0.006008762	-1.150603542
DCAF6	0.093582398	6.634705752	3.563287033	0.000633334	0.005896506	-1.152644019
RIPOR1	-0.138859269	6.619388177	-3.561414425	0.000637219	0.005923521	-1.158377468
ZNF384	-0.188860776	4.066678136	-3.518228516	0.000733343	0.00665086	-1.164459391
TONSL	-0.212748934	2.922356872	-3.474572901	0.000844365	0.007455751	-1.1647516
SGK3	0.285323895	1.650582015	3.403881666	0.001058466	0.008872971	-1.166930121
LRRK1	-0.187580295	5.359091257	-3.549505	0.000662462	0.006123561	-1.169059857
RNF113A	0.215201942	3.388432011	3.482570538	0.000822904	0.007303571	-1.172206189
KTI12	0.162278338	3.77299	3.509817194	0.000753597	0.006801791	-1.176907946
MTAP	-0.130940653	4.571617439	-3.52720278	0.000712302	0.006499164	-1.179141218
SRP19	0.164794035	2.96270532	3.473551029	0.000847145	0.007464911	-1.183046363
KMT2E	-0.257689023	5.885080405	-3.549441031	0.0006626	0.006123561	-1.183612343
ST3GAL5	-0.214225243	4.973827767	-3.534495284	0.000695627	0.006377919	-1.183651338
EIF4H	0.077398071	7.236233068	3.550799422	0.000659673	0.006104001	-1.184674617
COX15	-0.129994527	4.343680027	-3.520551851	0.00072784	0.00660891	-1.18592442
LLPH	0.114938638	3.847898578	3.505355818	0.000764554	0.006880072	-1.188921894
BSN	-0.583738859	-0.67694082	-3.288276748	0.001522265	0.011987141	-1.190007561
UNKL	-0.348395035	3.195818427	-3.461565975	0.000880402	0.007687208	-1.192650944
BIVM	0.132448524	4.074847019	3.515499478	0.000739857	0.006696001	-1.193654016
POLD1	-0.200762418	3.051519118	-3.473463123	0.000847384	0.007464911	-1.202149727
FAM57A	0.152949867	5.672719077	3.535377111	0.000693636	0.006363536	-1.2100414
TPRA1	-0.154762159	5.038765604	-3.52910624	0.000707913	0.006469309	-1.211260054
MIEF2	-0.193468014	2.934980279	-3.43903528	0.00094629	0.008139735	-1.21312569
MED25	-0.159563902	4.946566717	-3.526448241	0.000714049	0.006499355	-1.217600544
MED20	-0.128421428	3.960890266	-3.49541131	0.000789522	0.00707096	-1.217976522
RNF20	-0.164463451	6.005749965	-3.54023048	0.000682773	0.006286852	-1.21810348
LARP1B	0.223374466	3.519557152	3.467335149	0.000864242	0.007583351	-1.224410721
OCIAD1	0.111568508	6.333449809	3.539164616	0.000685145	0.006304837	-1.225351859
GTPBP10	-0.166836447	3.97726251	-3.490121848	0.000803114	0.007174218	-1.228438232
ABT1	0.141688234	4.192474802	3.50633765	0.00076213	0.006862357	-1.229464792
FNIP2	0.36059668	4.606094739	3.50836667	0.000757143	0.006825616	-1.232510473
SLC25A16	0.160102769	3.449086321	3.469926235	0.000857076	0.007537064	-1.237040077
ZNF397	-0.228095985	3.327134538	-3.455398951	0.000897993	0.007814766	-1.244455233
SLU7	0.095282007	5.71957031	3.526555102	0.000713801	0.006499355	-1.249958083
MAPK14	-0.231964526	5.807045227	-3.526695083	0.000713477	0.006499355	-1.251909179
PLCD4	-0.248298139	1.632737545	-3.367421128	0.001187991	0.009773837	-1.254236897
RERE	-0.158834769	6.509021196	-3.529993808	0.000705876	0.006460093	-1.254429906
STOM	0.15984276	8.150947027	3.516672773	0.00073705	0.006680457	-1.2584608
FAM117A	-0.233459274	2.06751341	-3.390086804	0.001105821	0.009178013	-1.261177861
PLEKHA2	-0.346702431	2.4352914	-3.402541843	0.00106298	0.008905857	-1.265931747
REEP5	0.085972997	6.823210857	3.524974785	0.000717472	0.006526567	-1.268913581
MARF1	-0.209001098	6.012582346	-3.522784768	0.000722588	0.006565178	-1.27243784
CRY1	0.216494737	3.768772653	3.460181026	0.000884324	0.007713641	-1.275343567
MAPKBP1	-0.204105399	3.806661219	-3.467295249	0.000864353	0.007583351	-1.276927478
MITF	-0.324470597	5.007193853	-3.5051419	0.000765083	0.006880723	-1.284885633
FAN1	-0.3121978	3.144398709	-3.438727283	0.000947222	0.008139735	-1.292496685
ZNF84	-0.178574206	4.125316642	-3.474210718	0.000845349	0.00746007	-1.295157819
RAB3IL1	-0.232821645	6.353346769	-3.515398893	0.000740098	0.006696001	-1.298733118
ZBTB34	0.287295728	3.31421272	3.436605396	0.000953668	0.008167169	-1.304408233
CCDC43	0.162669601	3.967051059	3.467330187	0.000864256	0.007583351	-1.304818896
RBM25	0.262771344	5.931469036	3.50774858	0.000758659	0.00683519	-1.306501348
PACSIN2	-0.123309747	5.329703887	-3.499244576	0.000779807	0.006996438	-1.313042951
RAB11FIP3	0.108313951	6.257405832	3.509076668	0.000755406	0.006814029	-1.316399533
GPR19	-1.059707126	-3.250733345	-3.246564375	0.001732198	0.013340402	-1.317013657
RALGAPA1	-0.361384661	1.56473606	-3.35102789	0.001250969	0.010202688	-1.321583763
RCE1	0.191490164	2.76371528	3.41120421	0.001034112	0.008723975	-1.331922731
ALG5	0.13894575	4.890507061	3.483973446	0.000819192	0.007280422	-1.334071326
PHOSPHO2	-0.365392592	-0.160544988	-3.263575497	0.001643501	0.01276828	-1.336490974
AFG3L2	0.13648372	4.933753665	3.484172244	0.000818668	0.007280055	-1.338127967
PARP2	-0.214508891	3.136684638	-3.417512863	0.001013556	0.008586182	-1.343003263
RAB22A	0.18074065	5.727348109	3.495193933	0.000790076	0.00707172	-1.344089937

Gene	Log fold change	Average Expression	t	P-value	Adjusted P-value	B
ABHD8	-0.199897132	3.514860958	-3.446974652	0.000922558	0.007964137	-1.347292231
ZFPL1	-0.180852654	2.518879796	-3.392910941	0.001095967	0.009126393	-1.349284545
FOXK1	-0.209316605	4.84221966	-3.477793279	0.00083566	0.007391888	-1.349358808
CDK17	-0.170854484	5.104212977	-3.483606916	0.000820161	0.007284728	-1.354802756
LIPE	-0.391051778	-0.087888788	-3.255828079	0.001683354	0.013044265	-1.356691944
LETM1	0.167523821	5.232678098	3.48243921	0.000823252	0.007303571	-1.357114394
CASKIN2	-0.164352954	4.181000156	-3.446768982	0.000923166	0.007964818	-1.358873558
PLEKHJ1	-0.207199109	4.541051096	-3.466527861	0.000866487	0.007593225	-1.3606816
MTA2	0.141629868	6.256402891	3.491274048	0.000800135	0.007153252	-1.368362219
PPP1R37	-0.173303401	5.806505945	-3.488647175	0.000806943	0.007192776	-1.368598041
SET	0.131445802	7.31456681	3.489482336	0.000804773	0.007181928	-1.369572341
CHMP2B	0.120804198	5.872034279	3.487698446	0.000809416	0.007206286	-1.3725914
NMNAT1	-0.235085556	3.275356048	-3.410850947	0.001035275	0.008723975	-1.375655488
CCT5	0.103881119	7.105139399	3.489129146	0.00080569	0.007185857	-1.37574641
PAPOLG	0.251424816	3.32640138	3.401647649	0.001066003	0.008921259	-1.378138605
KRBA1	-0.143530012	3.374041412	-3.417376888	0.001013995	0.008586182	-1.381456416
DDX21	0.447365332	6.723208104	3.487867842	0.000808974	0.007206286	-1.381733818
TMEM140	-0.155075098	4.197240787	-3.45035388	0.000912629	0.007896521	-1.391564028
FBXO36	0.317181138	1.00004023	3.290971792	0.001509558	0.01189952	-1.392357882
ZNF473	0.207651826	2.753484992	3.360402475	0.00121458	0.00994912	-1.399658527
PRIMPOL	0.191262117	2.844398578	3.410612976	0.001036059	0.008723975	-1.400147521
ZNF518B	-0.169882536	3.987994628	-3.436927656	0.000952686	0.008163404	-1.404729307
C1orf35	-0.233962244	2.810993147	-3.36722733	0.001188718	0.009774477	-1.406505783
RAP2C	0.137257139	4.570085976	3.452590207	0.000906114	0.0078582	-1.408742
PPP1R2	0.158617181	5.165773092	3.465021798	0.000870688	0.007625609	-1.41191191
BCCIP	0.175773368	5.101670218	3.461545792	0.000880459	0.007687208	-1.414227334
SIAH2	0.145058186	4.965326035	3.461578489	0.000880367	0.007687208	-1.414705052
MTA3	-0.091685385	4.715769842	-3.456532721	0.000894734	0.007790909	-1.415397392
IFIT2	-0.308095705	3.651648148	-3.391063964	0.001102402	0.009162275	-1.420343391
ZNF816	0.237594158	1.813040892	3.299505125	0.001469978	0.011679191	-1.423739943
CABYR	-0.290932868	1.147853121	-3.278129513	0.001571015	0.012292333	-1.434823864
B4GALT3	0.153765901	4.234855032	3.432546493	0.000966113	0.00825966	-1.440326205
DENND4B	-0.156297566	4.613380725	-3.444075273	0.000931159	0.008024588	-1.44040682
NECTIN4	1.19928078	-3.371736808	3.20290086	0.001980832	0.014924694	-1.441425075
TMEM184B	-0.122953872	6.747069504	-3.467750324	0.00086309	0.007583351	-1.442695378
CNN3	0.132552015	7.589081643	3.463145559	0.00087595	0.007662779	-1.442787546
EXOSC3	0.216668287	3.327195961	3.389143046	0.001109133	0.009200431	-1.444801258
MAML2	-0.351031395	5.027235201	-3.451559636	0.000909111	0.007870599	-1.448659205
ZNF680	0.311878118	1.622357902	3.278391493	0.001569738	0.012292333	-1.449473732
SNHG16	0.154911755	3.978617504	3.42156122	0.001000568	0.008501161	-1.449774916
PPP1CC	0.081401915	6.780655583	3.463968575	0.000873638	0.007646998	-1.454103108
JADE1	-0.291318185	4.768914087	-3.442611876	0.000935529	0.008057635	-1.455699214
NDP	-0.434218087	0.079561081	-3.203225259	0.001978867	0.014924694	-1.456738397
ZNF256	-0.205981444	1.523130701	-3.292043353	0.001504533	0.011878559	-1.458323311
TTK	-0.385941723	1.632302667	-3.290548018	0.001511549	0.011908986	-1.458440614
KBTBD7	-0.283253766	2.494260473	-3.326606768	0.001350641	0.010932864	-1.458487273
RPP25L	-0.201057594	3.253551134	-3.386301628	0.00111916	0.009278499	-1.458895908
IFT172	-0.127187708	4.487125798	-3.437730293	0.000950246	0.008152391	-1.461764291
HNRNPR	0.137761865	6.779337103	3.461431177	0.000880783	0.007687208	-1.462344499
AP5B1	-0.196781971	4.318040318	-3.429109748	0.00097677	0.008336578	-1.464390344
GUSBP1	0.268644747	2.269807839	3.333286879	0.001322659	0.010740991	-1.464511396
SKA3	-0.371925592	1.586079697	-3.296389022	0.001484317	0.011762098	-1.466484881
ZNF432	0.262260919	2.274554503	3.316913751	0.001392235	0.011206627	-1.472432379
GHITM	0.093717108	7.171926964	3.45398873	0.000902061	0.007838153	-1.478002233
PTCD3	-0.110479629	4.867518629	-3.438788675	0.000947036	0.008139735	-1.478661388
DNAJB2	-0.13207196	5.873914115	-3.452788289	0.000905539	0.007857738	-1.478930211
SLC43A1	-0.198523409	5.143887895	-3.441635191	0.000938456	0.008078228	-1.480442302
ZFAND4	-0.234941545	1.284002316	-3.27266879	0.001597853	0.012477998	-1.481523972
AMD1	0.258111983	5.714381529	3.449203357	0.000915998	0.007921124	-1.483451562
PKP4	-0.157505374	4.927577169	-3.429503298	0.000975544	0.008330834	-1.484479741
DNAJA2	0.120556192	6.260292909	3.452311351	0.000906924	0.0078607	-1.488493167
PRKAG2	0.176120119	3.425174674	3.382982951	0.00113098	0.009361036	-1.488530185
HMGB3	0.1446039	3.956111096	3.395872754	0.001085721	0.009056361	-1.497049962
SRSF8	0.109113574	5.147589372	3.437747736	0.000950193	0.008152391	-1.498082015
ACTR10	0.113606059	5.798976479	3.445368435	0.000927314	0.007996024	-1.49865689
GNAO1	-0.863094903	-3.008630529	-3.165768462	0.002218163	0.016411795	-1.499844904
EIF2B2	-0.122924016	4.178484972	-3.409654071	0.001039223	0.008745732	-1.502184234
PAN3	0.265455356	4.027113539	3.399784314	0.001072328	0.008959261	-1.503479607
ANKRD30B	0.950959183	-4.213295966	3.187404394	0.002076833	0.015472508	-1.505567035
BORCS7	0.15595774	4.63798763	3.419690691	0.001006549	0.008537534	-1.506977863

Gene	Log fold change	Average Expression	t	P-value	Adjusted P-value	B
SMCR8	-0.428688571	5.382714889	-3.434192343	0.000961048	0.008221026	-1.514341013
OGFR	-0.18304749	5.633646422	-3.436198946	0.000954907	0.008173136	-1.516731449
CCNL1	-0.320732518	3.900715831	-3.413776784	0.001025683	0.008665667	-1.516959691
VMA21	0.339011871	4.487561885	3.412926948	0.00102846	0.008684262	-1.517228307
GRIN2D	-0.225384814	3.994546724	-3.397893202	0.001078783	0.009008201	-1.517827265
HIST1H3H	-0.478819271	-0.867889213	-3.165307083	0.002221273	0.016426733	-1.525671354
ORC3	0.115092284	4.526614994	3.410723845	0.001035694	0.008723975	-1.528947932
PIGC	0.135843667	4.423527323	3.408800138	0.001042049	0.008759724	-1.534804356
COG2	-0.149752136	4.128466955	-3.39586337	0.001085753	0.009056361	-1.53900459
SREK1	-0.215191711	4.518734817	-3.404113071	0.001057688	0.008871388	-1.541829608
RNF26	-0.116458714	5.639421617	-3.428854912	0.000977564	0.008338636	-1.543935106
ZNF718	-0.339277769	1.100889069	-3.24564446	0.00173712	0.013364645	-1.544284337
ZSWIM8	-0.130123494	5.786468215	-3.430979057	0.000970959	0.008296389	-1.547956018
ZNF426	-0.156659511	4.210785597	-3.392504144	0.001097381	0.009131589	-1.550586953
TAF6	-0.129200286	5.16132689	-3.420400371	0.001004276	0.008527855	-1.550619626
ST3GAL2	-0.125637615	5.176286533	-3.415919254	0.001018712	0.008621278	-1.550898309
POLR2A	-0.16531503	7.800904758	-3.423684763	0.000993818	0.008457362	-1.55302724
R3HDM2	-0.162009595	4.580648678	-3.410807505	0.001035418	0.008723975	-1.556663591
EMC2	0.18253014	4.283682197	3.39468638	0.001089814	0.009080177	-1.563113775
TMEM216	0.160314851	3.416335662	3.361388585	0.001210811	0.009923641	-1.564099742
SLC12A7	-0.276245395	4.13281493	-3.384541839	0.001125413	0.009325208	-1.565528937
CTU1	0.2682058	1.786189234	3.26683377	0.001627006	0.012659717	-1.567220198
NSMCE4A	0.213055627	3.336308814	3.356217246	0.001230701	0.010059292	-1.569224057
PIK3IP1	-0.203788054	4.790847669	-3.395248833	0.001087872	0.00906901	-1.569296349
LINC00476	-0.370753201	0.787098331	-3.219896324	0.001880327	0.014313141	-1.572856362
ZNF850	0.465485024	-0.549760865	3.152145398	0.00231171	0.016987121	-1.573629539
TOB2	0.299958644	6.217899311	3.419897129	0.001005888	0.008536727	-1.579572788
NDFIP1	0.087022267	7.22654422	3.417708039	0.001012926	0.008586182	-1.5848429
SOCS2	0.257209588	3.663194408	3.373321901	0.001166062	0.009619692	-1.591295366
POMP	0.163412808	6.432507223	3.415489909	0.001020105	0.00862338	-1.599238821
EHD2	-0.152680826	9.578444688	-3.375676221	0.00115742	0.009564108	-1.600510851
CDADC1	-0.186402987	2.738439644	-3.308988416	0.001427136	0.011435307	-1.602124061
PHF19	-0.110574027	4.667771042	-3.392382889	0.001097803	0.009131589	-1.604315099
LINC02154	-0.79240567	-1.789050868	-3.116080667	0.002577443	0.018532986	-1.607439067
SELENOK	0.204025512	4.23003612	3.371445376	0.001172994	0.009666292	-1.608906421
INHBA	-0.538063021	5.264476187	-3.399953687	0.001071752	0.008959261	-1.61431297
FOXL2	0.55183416	1.371314136	3.195029343	0.002029065	0.015202767	-1.61435324
PLCE1	-0.225137976	3.915011839	-3.363902823	0.00120125	0.00987214	-1.617078366
C9orf64	-0.249481682	3.666793248	-3.35085975	0.001251631	0.010202688	-1.61709574
APBB1	-0.114996527	5.818094618	-3.406867724	0.00104847	0.008808786	-1.617214274
MVB12B	-0.157942613	3.617885268	-3.345475202	0.001273004	0.010356402	-1.61779809
FAM193A	-0.131171891	5.061779764	-3.390976284	0.001102709	0.009162275	-1.618547249
RNF216	-0.179167113	6.07979119	-3.405400945	0.001053369	0.008845011	-1.624673389
MYOM1	-0.319475115	0.793481724	-3.20310483	0.001979596	0.014924694	-1.635392247
MAP10	-0.404015212	0.510628169	-3.155976953	0.002285034	0.016832151	-1.650756846
ZFYVE26	-0.242017681	5.078736094	-3.374587849	0.001161407	0.009586542	-1.656939185
MINDY3	-0.124111813	4.385911066	-3.369639884	0.0011797	0.009710931	-1.659289447
SLC27A1	-0.112350188	5.667226714	-3.39039991	0.001104725	0.009173966	-1.662005191
PRDM16	-0.376824212	1.475536908	-3.223222037	0.001861227	0.014206979	-1.662086626
ZC3H6	-0.253847606	2.303811512	-3.255056878	0.001687371	0.013055241	-1.665574974
PITPNM1	-0.130094272	5.305379215	-3.383053852	0.001130726	0.009361036	-1.667629155
MAPK7	0.144904929	5.236043922	3.376102444	0.001155861	0.009556474	-1.670754763
C16orf52	0.200894444	3.663838903	3.346767371	0.001267844	0.010323676	-1.680092854
FUT4	-0.160744644	2.953472036	-3.303676832	0.001450985	0.01155346	-1.681601125
FGF7	-0.170497202	7.869507631	-3.374713512	0.001160946	0.009586542	-1.687534744
CCL2	-0.499479792	3.15333416	-3.34407934	0.001278601	0.010394408	-1.693716551
CLCN2	-0.352183608	0.556052139	-3.155642263	0.002287352	0.016840996	-1.699277096
ANKRD13D	-0.242863976	3.308202807	-3.306358043	0.0014389	0.011498986	-1.70068648
PURG	-0.740885434	-2.249385158	-3.075622814	0.002909307	0.020565835	-1.703427906
SIK3	-0.267975394	4.855741306	-3.36151943	0.001210311	0.009923641	-1.70364404
DARS	0.106111237	6.240718554	3.378226716	0.001148125	0.009497725	-1.708539559
ZNF383	0.237915911	2.763164628	3.258919245	0.001667346	0.012926863	-1.708552331
RAI1	-0.224079817	5.205463477	-3.359864922	0.001216639	0.009960573	-1.708879353
TMEM220	0.196592355	2.67591307	3.270836885	0.001606952	0.012531379	-1.709487859
HNRNPC	0.096919046	7.630635318	3.371550718	0.001172604	0.009666292	-1.709853354
ARRB1	-0.294083702	3.742701127	-3.323094578	0.001365576	0.011035962	-1.714235828
GPN3	0.124919049	3.5916064	3.313548816	0.001406954	0.011297633	-1.717051465
KCNJ2	-0.417369663	2.402750033	-3.253202079	0.001697067	0.013116789	-1.719622957
RGS19	-0.216207995	2.754193029	-3.267430254	0.001624003	0.012642882	-1.72208947
SPDL1	0.249737801	4.175339732	3.310378201	0.001420957	0.011391855	-1.722973795

Gene	Log fold change	Average Expression	t	P-value	Adjusted P-value	B
CDC23	-0.124743338	4.639632315	-3.345416746	0.001273238	0.010356402	-1.724301572
CAND2	-0.270979913	1.874595508	-3.218987175	0.00188558	0.014345644	-1.732180754
CEBPZ	0.136142537	5.291372375	3.358787092	0.001220778	0.009983608	-1.732708069
MTMR1	-0.157294507	3.995062329	-3.320741173	0.00137567	0.01110562	-1.734075791
MINK1	-0.099306833	6.329890778	-3.370085931	0.00117804	0.009702567	-1.734482906
ZNF644	-0.218255687	4.826059528	-3.35210116	0.001246751	0.010173924	-1.735146257
HSPA4	0.110903305	7.972258295	3.359315094	0.001218749	0.009972426	-1.738498237
C1QTNF5	0.65405165	-2.102467064	3.060802554	0.003040521	0.021268551	-1.740740438
DUSP12	0.172824967	3.221834707	3.278503932	0.001569191	0.012292333	-1.74105767
AADAT	0.16432798	2.978891049	3.271022747	0.001606027	0.012531379	-1.746168622
NOLC1	0.161415147	6.32100831	3.363327986	0.00120343	0.009884659	-1.749270349
TCAF2	0.321369567	0.406844043	3.127119839	0.002493223	0.018013384	-1.751041765
MBD2	0.170648208	6.367053915	3.361820472	0.001209163	0.009920935	-1.757798517
TRAPPC5	-0.300074376	0.642809039	-3.140281072	0.002396168	0.017522268	-1.759250348
SC5D	0.219751393	5.701599662	3.352164125	0.001246504	0.010173924	-1.760908127
AVL9	0.276899326	4.248665784	3.319968177	0.001379	0.011123249	-1.763693938
SPACA6	0.325968921	0.862671778	3.14927514	0.002331884	0.017101993	-1.768318287
SHF	-0.25226241	2.182869901	-3.225019654	0.001850979	0.014139526	-1.771504541
DDX52	-0.098207518	4.584066405	-3.331147829	0.00133156	0.010801622	-1.777160252
EGLN3	0.430045561	-0.054739553	3.096724682	0.002731548	0.019436961	-1.779867591
SCAI	-0.261293657	1.939363906	-3.203183712	0.001979119	0.014924694	-1.784670771
RTN4IP1	-0.199047358	1.690084077	-3.192322346	0.002045905	0.015287453	-1.79040091
ZNF286A	0.298858943	1.158038215	3.132646802	0.002452027	0.017826898	-1.790545955
EXO5	-0.189486101	1.803034984	-3.18204966	0.002111005	0.015688299	-1.791963777
TRIM3	-0.163347589	3.978655486	-3.298022253	0.001476785	0.011721874	-1.793837222
BRPF1	-0.139822059	4.330299577	-3.321306369	0.001373239	0.011091943	-1.797198876
NLK	0.262395569	3.053653591	3.250695663	0.001710253	0.013191632	-1.798219751
TMEM151A	0.764998333	-1.57718837	3.038791977	0.003245583	0.02240187	-1.79895855
RFT1	-0.122014273	3.875600684	-3.297061908	0.001481209	0.011743652	-1.80080159
IMP3	0.167997175	4.527337927	3.316608099	0.001393566	0.011206812	-1.809064757
ATP6V0C	-0.983640832	-3.006644284	-3.032859829	0.003303007	0.022688783	-1.814605642
ZNF257	-0.734813571	-1.492002864	-3.031239668	0.003318854	0.022787246	-1.816126016
MIR4534	0.445677295	0.818205472	3.131883444	0.002457679	0.017842154	-1.817490232
CCL7	0.520162206	-0.937317011	3.058603582	0.003060453	0.021393347	-1.818455395
TBCE	0.111262442	4.325117486	3.305438272	0.001443035	0.011525916	-1.818820919
ZNF639	-0.097228714	4.696835495	-3.319029169	0.001383056	0.011147331	-1.823281122
TRANK1	-0.290995034	4.961295186	-3.324489682	0.001359625	0.010993769	-1.824580717
EWSR1	0.158495015	6.691778243	3.339160948	0.001298505	0.010550529	-1.824703523
TRIM24	-0.188696351	4.159617874	-3.294378332	0.001493639	0.011823529	-1.826841557
SNX7	0.10813974	5.566779264	3.33233193	0.001326626	0.010767397	-1.827290846
KCTD9	0.144162968	4.508153883	3.308224353	0.001430544	0.011450431	-1.829036946
PAK1IP1	0.157055219	4.538035139	3.307766763	0.001432588	0.011454624	-1.830316775
RPL22	0.081259207	7.823038638	3.326689802	0.00135029	0.010932864	-1.83320703
TRA2A	0.200571006	4.384637349	3.308755881	0.001428172	0.011437527	-1.838365706
RFC1	-0.195435869	5.476151334	-3.324572194	0.001359274	0.010993769	-1.839166383
SRSF7	0.126500902	5.840160264	3.328837478	0.001341236	0.010874261	-1.84210933
GATA6	-0.32889562	3.580070312	-3.283139886	0.001546764	0.012154642	-1.843501273
LACC1	0.213759182	3.97253754	3.285553262	0.001535208	0.012076436	-1.844495298
TADA2A	-0.206125117	3.1718641	-3.241134095	0.001761444	0.013531049	-1.845251266
ARMC1	-0.10863221	4.724083289	-3.30789189	0.001432029	0.011454624	-1.846643507
CDCA2	-0.243947134	2.474291127	-3.201522828	0.001989197	0.014967667	-1.848739566
EDEM1	0.245093005	4.499252887	3.297995887	0.001476906	0.011721874	-1.850340287
TMEM11	0.196882358	4.599675141	3.304266401	0.001448319	0.011543642	-1.852903575
RTKN	-0.194444725	3.428007467	-3.238829458	0.001773995	0.013599726	-1.853819781
SCCPDH	0.129112835	6.071304911	3.327475986	0.001346969	0.010914869	-1.855013748
BRAP	0.116477316	4.476527364	3.294998503	0.001490758	0.011806927	-1.856367078
RFX7	0.407546229	4.621602837	3.285106449	0.001537341	0.012086905	-1.857944594
GRIPAP1	-0.158256375	4.224814067	-3.297588316	0.001478783	0.011730586	-1.859139879
ZNF337	0.404717654	-0.322842371	3.041764643	0.003217156	0.022307391	-1.8625895
PPP4R3B	-0.159691793	5.764409841	-3.318803687	0.001384032	0.01114923	-1.869525299
TOGARAM1	-0.249587278	4.218389848	-3.277439801	0.001574382	0.012307464	-1.875376678
PDK1	0.223483755	3.129504175	3.220975338	0.00187411	0.014280015	-1.878297432
CHRM3	-1.02823398	-2.995110197	-3.013642442	0.00349558	0.023740943	-1.879024979
BEND7	-0.179138075	2.675097587	-3.199737955	0.002000082	0.01501956	-1.879688801
EPN2	-0.180499923	4.422960578	-3.286727012	0.001529617	0.012038744	-1.881605719
TUBA1C	0.147584543	5.22751852	3.304920305	0.001445368	0.011538436	-1.881833436
INPP5F	-0.214130814	3.376596262	-3.245842468	0.001736059	0.01336331	-1.881923985
SLC8B1	-0.116535848	5.046309432	-3.304692582	0.001446395	0.01154052	-1.881979904
FASTKD1	-0.211003856	2.62135451	-3.216447479	0.001900327	0.014428696	-1.882124145
LRSAM1	-0.144815895	4.307206016	-3.29318159	0.001499213	0.011848977	-1.883357836

Gene	Log fold change	Average Expression	t	P-value	Adjusted P-value	B
WDR92	-0.242144387	1.407645033	-3.136733061	0.002421979	0.01767669	-1.88394007
RASSF1	-0.175191625	4.18009197	-3.278819947	0.001567652	0.012292333	-1.88417368
NAP1L3	0.333048874	3.108138493	3.20986924	0.00193903	0.014656069	-1.887663831
SYCE1L	1.058575631	-2.622021914	3.000130806	0.003637157	0.024536558	-1.889096123
RNF219	0.270757757	3.222851008	3.22761378	0.001836283	0.014048638	-1.88992752
SRSF1	0.075032777	6.552949731	3.3164728	0.001394156	0.011206812	-1.891634268
PUF60	0.130808517	6.968540689	3.315222465	0.001399615	0.011244696	-1.892160257
C18orf54	-0.284667766	2.381912879	-3.168318187	0.002201053	0.016301209	-1.899573064
SLC25A12	0.119123883	4.308761142	3.277002	0.001576522	0.012317803	-1.899729958
ZNF773	-0.252298155	1.93856028	-3.154816904	0.00229308	0.016866677	-1.899814371
LZTR1	-0.095642869	5.929044229	-3.31119978	0.001417316	0.011368716	-1.900974707
LRRFIP1	0.121590526	6.573507517	3.311985986	0.00141384	0.011346877	-1.90346869
BTF3L4	0.157962103	4.714338382	3.291354699	0.001507761	0.011894901	-1.911083652
MAGI2-AS3	0.115147333	5.659663206	3.303570169	0.001451468	0.01155346	-1.913148478
TRIM45	-0.483265383	-0.656911099	-3.01695661	0.003461643	0.023552929	-1.914951156
ZNF454	0.394116932	0.024742621	3.026944968	0.003361203	0.0230465	-1.915071892
CMPK1	0.091769298	7.418029991	3.303485238	0.001451852	0.01155346	-1.916383191
KLHL23	-0.341854721	0.203078344	-3.07307154	0.002931513	0.020693678	-1.916463759
RP9	0.244936401	2.271297772	3.171835945	0.002177648	0.016143747	-1.919706819
U2SURP	0.148083726	5.642251186	3.302561437	0.00145604	0.011580671	-1.921646993
KRTAP2-3	-0.747431736	-2.350383303	-3.002804373	0.003608728	0.024399406	-1.922802351
RALGDS	-0.143427873	5.311682924	-3.293653876	0.001497011	0.011837782	-1.924658623
UBE2Q1	0.096092734	6.170125195	3.304382957	0.001447793	0.011543642	-1.925223871
POLK	-0.259168645	4.016305578	-3.255172342	0.001686769	0.013055241	-1.92838235
LRIG3	-0.534323581	5.240207661	-3.278469276	0.00156936	0.012292333	-1.9301384
ZBTB20	-0.499675429	-0.087222813	-3.050758987	0.003132551	0.02182643	-1.930942392
RRP15	0.148339708	4.392212933	3.270434634	0.001608957	0.012538709	-1.933902854
KY	-0.464577008	0.221966586	-3.03531037	0.003279172	0.022566216	-1.934210693
MESDC2	0.073383014	6.244740162	3.300748232	0.001464294	0.011640171	-1.935374378
MAGI3	-0.252306621	3.287814589	-3.221885617	0.00186888	0.014247374	-1.936149007
ZNF184	0.259427359	2.893925777	3.181275166	0.002115991	0.015717599	-1.937297997
ZNF573	-0.414299962	-0.916269618	-3.00071101	0.00363097	0.024510349	-1.937580053
ENTPD6	-0.112458283	5.570013751	-3.294067395	0.001495085	0.011828764	-1.939793507
NIM1K	-0.383602299	0.38019963	-3.043534461	0.003200343	0.022247328	-1.941399473
COG7	-0.139284277	3.509966312	-3.24362774	0.001747957	0.013441153	-1.944120964
TDP2	-0.105532544	5.081066421	-3.282205706	0.001551259	0.012183607	-1.947797202
IQCC	0.497062228	-0.260008813	2.998690558	0.003652558	0.024585403	-1.950137704
MOCS1	-0.120373399	4.054979883	-3.249106597	0.001718663	0.013249714	-1.95271331
TPX2	-0.278668726	4.630993381	-3.269596208	0.001613142	0.012564828	-1.955029611
CRYZL1	0.121798469	3.901238723	3.241502405	0.001759445	0.013522595	-1.956351422
TTI2	0.158474397	2.609573921	3.167487011	0.002206617	0.016334388	-1.957491424
LMBR1L	-0.144384974	3.495223172	-3.218493749	0.001888437	0.014360127	-1.957869039
RANGAP1	-0.109644159	6.172595635	-3.292665901	0.001501621	0.011861786	-1.958126826
LRP11	0.113909158	6.300631085	3.291264806	0.001508182	0.011894901	-1.963811849
BNIP1	0.225722505	2.678275508	3.182816451	0.00210608	0.01565942	-1.967179769
MRPS22	0.164171131	4.873622982	3.270790293	0.001607184	0.012531379	-1.969305648
EIF3M	0.115629882	7.275374653	3.279692668	0.001563411	0.012272656	-1.98990747
FAM118A	-0.333326514	3.040491252	-3.187115453	0.002078664	0.015478492	-1.990870228
CARF	-0.242368193	1.840824885	-3.130812149	0.002465631	0.017874042	-1.995787135
GGA2	-0.137401742	5.939110342	-3.27800349	0.00157163	0.012292333	-1.997060151
SLC46A1	-0.239757484	0.944624924	-3.063182887	0.003019082	0.021163077	-1.998544126
ARID5A	0.263558103	4.627939506	3.262236631	0.001650324	0.012814682	-1.999677716
BZW1	0.13309998	6.484624259	3.278064474	0.001571332	0.012292333	-2.001909233
MIR34A	0.561846264	0.706988747	3.041673552	0.003218024	0.022307391	-2.002229219
KCNA3	-0.791118401	-2.264447449	-2.970290624	0.003968864	0.026273993	-2.005777581
FGF9	-0.840836363	-2.102482286	-2.952088892	0.004184726	0.027408133	-2.006662398
KIFC1	-0.35835124	2.426011641	-3.147398695	0.002345161	0.017190999	-2.011702664
SNIP1	0.173813834	4.299007609	3.240251741	0.001766239	0.013560972	-2.012451294
TAF6L	-0.186977185	3.110490782	-3.194955099	0.002029525	0.015202767	-2.012633569
MSL3	-0.103870656	4.389343003	-3.239012447	0.001772995	0.013598983	-2.017145033
APOL3	-0.164376354	5.091969825	-3.25516196	0.001686823	0.013055241	-2.023255993
THTPA	-0.206705497	2.226616528	-3.131948961	0.002457193	0.017842154	-2.028316615
THADA	-0.125880023	4.921707282	-3.251957124	0.001703605	0.013153822	-2.032684397
METAP2	0.083155903	7.028230177	3.265186096	0.001635327	0.012711338	-2.033715081
NCOA7	-0.245114453	5.010651053	-3.251055782	0.001708353	0.013183724	-2.03430251
FAM131A	-0.159225104	3.045856895	-3.153915385	0.002299351	0.01690455	-2.036154337
CTIF	-0.148245745	6.615018612	-3.265208546	0.001635214	0.012711338	-2.039266569
LINC00667	0.186637787	4.208052549	3.219890982	0.001880358	0.014313141	-2.041529663
PITPNB	0.147377265	5.925321791	3.261461262	0.001654288	0.012838842	-2.043554205
ALKBH1	0.21748445	2.845911141	3.155040623	0.002291526	0.016863482	-2.053370886

Gene	Log fold change	Average Expression	t	P-value	Adjusted P-value	B
ZEB2	-0.211551221	6.709438454	-3.258932412	0.001667278	0.012926863	-2.057187222
CDV3	0.15268911	7.585817109	3.248776746	0.001720414	0.013256425	-2.061389808
GEN1	0.263245549	2.182790133	3.114298381	0.002591287	0.018614758	-2.061784511
ABCE1	0.12736678	6.066220783	3.254096379	0.001692386	0.013087319	-2.061998074
NR6A1	-0.726277727	-1.266589628	-2.930228276	0.004458316	0.028781943	-2.063658123
GOSR2	0.116729391	3.680019556	3.192045101	0.002047637	0.01529281	-2.064241543
CACNB3	-0.126472311	4.381478004	-3.222733306	0.001864022	0.014217535	-2.0727085
ECD	0.094886102	5.055734617	3.235516397	0.001792186	0.013725207	-2.073196424
CNNM1	-0.40248428	0.521606131	-3.014611204	0.003485628	0.023684031	-2.074549678
SLC35G1	0.173670327	2.709594572	3.134293066	0.00243988	0.017772894	-2.075607157
ZER1	-0.096521191	6.244237907	-3.252244122	0.001702096	0.013148908	-2.076851348
KIAA1522	-0.197058505	4.884438094	-3.225646323	0.001847419	0.01411949	-2.079843093
MEG9	0.790194032	-1.472444278	2.92787431	0.004488742	0.02890911	-2.082466859
TMCC3	-0.601417764	-0.551564232	-2.945812175	0.004261647	0.027791105	-2.083934004
CCL8	-0.974796744	-2.879350242	-2.915823258	0.004647532	0.029640314	-2.084702147
SH3YL1	0.137927047	3.775898675	3.190910773	0.002054737	0.015338238	-2.088543077
ELK4	0.185316929	5.430342609	3.239972535	0.001767759	0.013565729	-2.088553486
RNF152	-0.305606847	2.941534833	-3.157858129	0.002272042	0.016752835	-2.089882617
SERTAD4	-0.535355008	2.177609479	-3.081150092	0.002861733	0.020286658	-2.091645461
GFOD1	0.370980538	0.851941371	3.021596396	0.003414645	0.023317413	-2.091926213
NUP43	-0.146598952	4.046709524	-3.200009557	0.001998422	0.015017107	-2.095218387
SIPA1	-0.171832323	4.279772236	-3.210497432	0.001935302	0.014642589	-2.096493151
PDK4	-0.812635892	-1.409490528	-2.918658798	0.00460971	0.029448969	-2.097982274
TMEM178A	1.071705902	-3.659262025	2.918336689	0.004613992	0.02946383	-2.098814829
FN3K	-0.289710575	3.906090008	-3.19823627	0.002009283	0.015081136	-2.101154167
TUBD1	-0.288786614	1.101703372	-3.020943388	0.003421223	0.023351749	-2.102229076
TAPT1-AS1	-0.385530256	-0.868296388	-2.959518506	0.004095335	0.026986934	-2.103406284
HDX	-0.264648269	1.968873064	-3.076481877	0.002901865	0.020522859	-2.104133788
TBC1D30	-0.432956505	-1.063860166	-2.941274851	0.004318064	0.02806183	-2.106140177
TSC1	-0.166315453	5.003091299	-3.217742082	0.001892797	0.014378763	-2.108741572
ZNF652	0.165386646	4.450076125	3.207527707	0.001952983	0.014754132	-2.10887294
UBAP2	-0.122813709	4.824195194	-3.215959481	0.001903174	0.014443026	-2.110997063
KCND1	-0.260859492	2.400928375	-3.099234992	0.002711089	0.019327895	-2.113530536
EOGT	0.236563294	4.095278724	3.190135417	0.002059604	0.015359346	-2.113698734
C11orf24	0.128031222	6.570157512	3.238536013	0.001775599	0.0136051	-2.116541327
PPA2	0.115897328	4.914961425	3.218055981	0.001890975	0.014372171	-2.118564535
ZNF263	0.141918793	4.67763459	3.211379298	0.001930081	0.014617767	-2.120250263
CDC20	-0.341597685	2.904816015	-3.135653103	0.002429887	0.017717237	-2.120259107
EIF3J	0.134913284	5.716116512	3.230948404	0.001817553	0.013912407	-2.12172452
AMER1	0.393250162	3.437917893	3.144880577	0.002363089	0.017288771	-2.123774371
CACFD1	-0.158846621	4.177251233	-3.202789774	0.001981505	0.014924694	-2.124776508
RNF8	-0.128117819	4.228875717	-3.186820939	0.002080531	0.015484743	-2.130510418
INO80	0.142882361	4.829969558	3.214078151	0.001914183	0.014511955	-2.131766112
IFT80	0.153490158	4.673391737	3.20203671	0.001986074	0.014951633	-2.138294069
IVD	-0.08110705	5.529264325	-3.223140116	0.001861695	0.014206979	-2.141279245
SRP9	0.112108101	5.883623486	3.226416946	0.00184305	0.014093248	-2.143677943
CDC6	0.278614273	2.79318541	3.118847619	0.002556088	0.018423401	-2.144018366
ZNF451	-0.179161181	4.417657652	-3.196254844	0.002021484	0.015157608	-2.144175602
FAM204A	0.141636451	5.137774254	3.214529277	0.001911538	0.014499196	-2.146215492
CCDC77	-0.176343533	2.407474073	-3.096824748	0.00273073	0.019436961	-2.148483338
MCUR1	0.100872137	5.010869439	3.211052294	0.001932016	0.014625066	-2.148961386
BRF2	0.162518764	2.232055947	3.070200962	0.002956687	0.020832334	-2.155441858
STK38L	0.265609646	4.421934173	3.189119048	0.002066	0.015399421	-2.156152747
STAC3	0.501187296	-1.276610557	2.900965568	0.004850438	0.030610387	-2.161603846
CD72	-0.305027387	-0.188798677	-2.947979158	0.004234944	0.027649763	-2.167230164
SKA1	-0.391599812	0.599134303	-2.978787407	0.003871667	0.025795134	-2.167576249
BEST1	-0.161990075	3.149696595	-3.13613883	0.002426327	0.01769985	-2.169075453
RABL2B	0.123167427	3.25235256	3.137534743	0.002416124	0.017642508	-2.171686964
ZNF354C	-0.290985476	2.858823485	-3.098598434	0.002716263	0.019348508	-2.176011739
PMPCA	0.137804127	5.664559062	3.209958219	0.001938502	0.014656069	-2.177473466
MIR3189	0.975331533	-3.122669002	2.873142872	0.005252533	0.03250787	-2.182386525
TMEM2	0.396299525	4.291694662	3.193263741	0.002040034	0.015251149	-2.185856622
NHLRC1	-0.320506315	0.436590327	-2.951663042	0.004189903	0.027418204	-2.186180891
DIS3	0.176048472	5.900230872	3.211863804	0.001927218	0.014603425	-2.18661694
HLF	0.902070755	-3.244457333	2.871618767	0.005275422	0.032619093	-2.187888789
ZNF596	-0.296286285	1.220380087	-2.997785906	0.003662262	0.024629337	-2.188289074
CCNC	0.098998835	5.387026386	3.203881137	0.001974901	0.014912232	-2.188822866
PIGO	-0.199514547	3.813312998	-3.159728409	0.002259194	0.016682598	-2.190653761
PLPP6	-0.243059292	2.170068694	-3.064514508	0.00300715	0.021128593	-2.196806733
VRK2	-0.159731376	3.350487811	-3.135492772	0.002431063	0.017717237	-2.203678122

Gene	Log fold change	Average Expression	t	P-value	Adjusted P-value	B
SAR1B	0.094047761	5.509004466	3.199954514	0.001998759	0.015017107	-2.20606171
DUSP8	-0.686000647	-1.592211541	-2.907166743	0.004764777	0.030216526	-2.210115807
SMG8	-0.222591338	4.132520184	-3.157908612	0.002271694	0.016752835	-2.213627201
SBF1	-0.095314046	7.177782754	-3.200930208	0.001992805	0.014987328	-2.215646223
SGTB	0.163713243	4.091010876	3.156723074	0.002279873	0.016802349	-2.217148674
XPC	-0.1777481	5.48816694	-3.193289043	0.002039876	0.015251149	-2.223724048
C12orf4	-0.162092833	4.435305785	-3.173256528	0.002168263	0.016082084	-2.223900773
CEP126	-0.16634703	2.788414855	-3.104450256	0.002669039	0.019073245	-2.224863723
ABTB1	-0.162568248	3.606540367	-3.146621119	0.002350683	0.017223101	-2.226267698
POLR3H	0.087642399	5.952206543	3.196733352	0.002018531	0.015143005	-2.226306292
SLC10A5	-0.794215369	-2.295885368	-2.85880024	0.005471601	0.033587695	-2.229019544
MRPL35	-0.087101123	4.415729828	-3.164817273	0.002224578	0.016443106	-2.230361025
JAG2	0.470953164	-0.620305338	2.899693578	0.004868183	0.030683812	-2.232932256
ATL3	0.149387101	7.083673224	3.194459502	0.002032599	0.015218226	-2.234909667
RAN	0.093009563	7.307547618	3.193715033	0.002037225	0.015245282	-2.236983277
ZNF610	0.326934526	1.317509166	2.95309104	0.004172564	0.027351342	-2.239701004
TTC21B	-0.196728025	3.481715482	-3.118233147	0.002560816	0.018448653	-2.251862609
ZNF845	0.263933334	2.418996062	3.044753558	0.003188808	0.022177381	-2.252395796
PRDX3	0.074995172	6.452079505	3.19051275	0.002057234	0.015349272	-2.253077515
ZNF697	0.347391001	3.847131254	3.138210717	0.002411198	0.017615075	-2.255805028
PREB	-0.15141721	5.664098689	-3.183587615	0.002101137	0.015630384	-2.255850905
ANKHD1	0.470677157	-0.772401266	2.873859656	0.0052418	0.032468121	-2.257154969
HNRNPUL2-BSCL2	0.540795576	-2.029109338	2.844538132	0.00569775	0.034539598	-2.257803402
FANCG	-0.152150477	3.719013258	-3.133514204	0.00244562	0.017797494	-2.257948746
EFCAB12	-0.657819442	-2.412758245	-2.840883643	0.005757065	0.034857084	-2.266554751
SENP8	-0.212851139	1.293093875	-2.972541788	0.003942895	0.026188609	-2.268413258
HNRNPD	0.128401566	7.421413339	3.180777213	0.002119203	0.015733695	-2.270217526
SMN1	0.244021852	1.207419351	2.979239964	0.003866553	0.025772457	-2.270714383
TNFAIP8L1	-0.261731175	1.544075416	-2.984660859	0.003805774	0.025468758	-2.270970275
PLCL2	-0.250194381	3.926483013	-3.127345668	0.002491527	0.018009769	-2.271476768
EID3	-0.30671768	0.912316852	-2.971353641	0.003956581	0.026231362	-2.271491062
TMEM69	0.141442257	4.007495556	3.131889753	0.002457632	0.017842154	-2.279036445
SEMA6D	-0.491897144	1.263120866	-2.936479325	0.004378442	0.028368536	-2.279062762
LZTS1	0.389113042	0.942703646	2.968477258	0.003989898	0.026384467	-2.280082826
SNX5	0.111889991	6.07940959	3.17840752	0.002134548	0.015839817	-2.28408754
ZNRF2	0.195960394	2.612814509	3.063792114	0.003013617	0.02115298	-2.284466697
RNF39	0.348277011	0.141430056	2.904684234	0.0047989	0.030363913	-2.287374745
RAD17	-0.128313449	3.886079112	-3.120263398	0.002545225	0.018362675	-2.292935248
INPP1	0.113789398	4.694934988	3.149502699	0.002330278	0.017098544	-2.293282897
THAP8	-0.243459826	3.635757337	-3.109136004	0.002631777	0.018849827	-2.297978683
PRNP	0.084995969	8.654079724	3.150906891	0.002320395	0.017042626	-2.299572508
ESCO1	0.330157043	3.539905333	3.100986614	0.002696897	0.019244937	-2.300784315
EXOC3	-0.10814162	5.94568179	-3.169385315	0.002193928	0.016256436	-2.307946281
TRIP11	-0.19870202	5.335222324	-3.158894006	0.002264918	0.016716668	-2.309127384
ZNF514	-0.245998567	1.818517575	-2.979615379	0.003862315	0.025764591	-2.31250215
BEND3	0.317305593	2.673363191	3.036566673	0.003267014	0.022513406	-2.31267001
PLEKHG5	-0.234289303	1.89089943	-3.033683615	0.003294977	0.022664626	-2.313186228
SRPK1	-0.179710099	4.835346578	-3.146164413	0.002353933	0.017230148	-2.314241243
DCAF4	0.23561734	4.122673375	3.110755487	0.002619012	0.018787046	-2.317057002
PLA2G12A	0.114353075	4.37768627	3.133538785	0.002445438	0.017797494	-2.319928362
SERBP1	0.067086606	7.579911186	3.160480506	0.002254047	0.016652751	-2.321656591
CFAP157	-0.43459513	-0.480004425	-2.855389168	0.005524925	0.033825706	-2.327840058
LOC103344931	-0.232691636	1.718752912	-3.006694341	0.003567732	0.02418736	-2.332420101
FXYD6	-0.776686626	-2.141923364	-2.809308682	0.00629369	0.03742919	-2.340422091
EDARADD	-1.023611832	-3.518030199	-2.811807616	0.006249603	0.03719637	-2.341382304
HDAC6	-0.091723515	4.479172687	-3.130466196	0.002468204	0.017884089	-2.342019788
PHLDA2	0.284901795	3.083436193	3.062722776	0.003023215	0.021182193	-2.343860218
IRAK2	0.411314126	1.014013685	2.906598866	0.004772563	0.03024564	-2.347757103
NRIP3	0.232833719	1.931992944	2.987597551	0.003773218	0.025307105	-2.347929235
ASB14	0.485024074	-1.676332365	2.813108435	0.006226766	0.037104843	-2.350105939
ROCK1P1	-1.045334346	-4.480186124	-2.813166229	0.006225753	0.037104843	-2.351460065
ZIC4	-0.23043559	2.035615364	-3.015157661	0.003480026	0.023656637	-2.35161283
POLL	-0.160564669	4.282545823	-3.119768323	0.002549019	0.018381242	-2.356489733
ST3GAL4	-0.169238878	4.456448977	-3.121107992	0.002538765	0.018324846	-2.359185781
CEP131	-0.240310007	3.092239795	-3.072674138	0.002934986	0.020708491	-2.360091488
TUBGCP4	-0.150367329	3.635620817	-3.091066864	0.002778189	0.019750187	-2.360990495
UBE2B	0.105086099	6.000244127	3.150324474	0.002324489	0.01706438	-2.361672193
EHD1	0.152203735	7.202123632	3.146424962	0.002352079	0.017224945	-2.368481044
MERTK	-0.58350719	-1.703056415	-2.802450142	0.006416161	0.037969918	-2.372075732
ANKRD13C	0.146277279	5.02597667	3.131283634	0.002462128	0.017857244	-2.373807284

Gene	Log fold change	Average Expression	t	P-value	Adjusted P-value	B
GCLM	0.239381048	5.13316337	3.129458318	0.002475714	0.017929885	-2.374606936
CIART	-0.254278189	2.017818515	-3.017742187	0.003453643	0.023509121	-2.380221408
SOCS6	-0.179604879	4.845977168	-3.124187863	0.002515337	0.018164451	-2.383254109
RRP1	0.212361647	5.263785488	3.129031075	0.002478905	0.017944362	-2.383857314
KPRP	-0.780599656	-4.821971802	-2.79628221	0.006528163	0.038459226	-2.386049811
KLF12	-0.215028975	4.517058216	-3.113111273	0.002600546	0.018672367	-2.387396274
TP53BP2	0.169647236	4.943234056	3.128021443	0.002486459	0.0179904	-2.388405416
MLLT1	-0.098582516	7.038455564	-3.139855198	0.002399252	0.017536311	-2.389866237
STAMBPL1	-0.282359877	2.64026295	-3.038710216	0.003246368	0.02240187	-2.390054727
LCA5	-0.328855231	1.982248428	-2.943916951	0.004285129	0.027895931	-2.401256356
LINC00954	-0.674573356	-2.697904756	-2.775897843	0.006911192	0.040150479	-2.402304822
B3GALT5	-0.451948686	-0.602422926	-2.826996535	0.005987677	0.036021713	-2.402473306
UBR2	-0.197841176	4.923758912	-3.110270196	0.002622831	0.018805487	-2.406660933
AREG	0.910621566	-4.083079132	2.772924612	0.006968749	0.040406941	-2.409176545
SNRPD1	0.157141735	4.761143201	3.106192765	0.002655125	0.018991835	-2.410969074
ARHGEF10L	-0.1190241	5.046495641	-3.115681414	0.002580538	0.018546389	-2.415563512
HMGCS1	0.329359451	6.92190708	3.132656979	0.002451952	0.017826898	-2.415715578
KIAA0895L	-0.256380855	1.504112755	-2.949077813	0.004221465	0.027588784	-2.416728431
TMEM8B	-0.133139475	5.069469765	-3.117296829	0.002568037	0.018476447	-2.417313474
RSL24D1	0.174286965	6.552135691	3.131426845	0.002461065	0.017857244	-2.419201607
CTBP1-AS	0.641514438	-1.934828657	2.776151115	0.006906309	0.040137591	-2.420383023
ITGAE	0.160781601	3.257028763	3.046967126	0.003167964	0.02206297	-2.42263734
SNX1	0.071475794	6.874640842	3.127601594	0.002489606	0.018004526	-2.427890856
TTC32	0.37013925	-0.06282962	2.858370907	0.005478287	0.033601349	-2.429474605
TCHH	0.560076727	-1.869780875	2.771076986	0.007004737	0.040553173	-2.431632861
ZNF699	-0.678720694	0.268301297	-2.851202018	0.005591034	0.034084719	-2.432662689
WNK4	-0.415447653	-0.015830884	-2.847009495	0.005657957	0.034381387	-2.435511578
NEXN	0.211468879	5.229750969	3.112213479	0.002607569	0.018713874	-2.436445147
ZNF607	0.380369698	1.684454597	2.895219138	0.004931083	0.030976576	-2.436881053
RPH3AL	-0.374570653	-0.373386535	-2.838780967	0.00579145	0.035023042	-2.437403788
TOX2	-0.315357855	2.58800201	-3.017986897	0.003451155	0.023502803	-2.439777219
KIF13B	-0.319360395	3.00349293	-3.035323361	0.003279046	0.022566216	-2.443776436
DERL2	0.117506091	5.109606022	3.107681837	0.002643289	0.018916152	-2.446222072
NUDCD2	0.131955721	4.437031587	3.088945313	0.002795869	0.01985713	-2.4464401
KIAA1755	-0.228690643	3.345570912	-3.039955575	0.003234428	0.022380586	-2.447806404
CRIPT	0.124347325	4.184897106	3.077316278	0.002894653	0.020481473	-2.448864697
MAP4K2	-0.141156886	2.754065847	-3.020588167	0.003424807	0.023365622	-2.449728253
ARHGEF18	-0.232791473	1.659689492	-2.92321158	0.004549576	0.029163751	-2.452050359
IFI16	0.113409002	8.343996348	3.098563333	0.002716549	0.019348508	-2.45246638
ATP13A1	-0.105804995	6.022900255	-3.117461594	0.002566765	0.018476447	-2.452759114
NAP1L4	0.092547522	5.929555609	3.117255748	0.002568354	0.018476447	-2.45332064
DUS2	-0.163306468	2.788009318	-2.994982045	0.003692492	0.024820132	-2.455057535
PAK1	-0.11281409	5.015145655	-3.102291962	0.002686367	0.019178875	-2.455111037
LARS2	-0.131093966	4.479352264	-3.084326737	0.00283472	0.020104627	-2.456136919
FANCM	-0.294823812	1.108440442	-2.89241174	0.004970929	0.031123059	-2.457785718
LYSMD1	0.226320738	1.952687192	2.923539335	0.004545275	0.029160999	-2.45889386
HSDL1	-0.141992715	4.146225073	-3.071530377	0.002945003	0.020759724	-2.46136846
ZNF169	-0.372846184	-0.522529495	-2.787626788	0.006688359	0.039234206	-2.465437655
SNAPC1	0.141484812	3.179940536	3.041534521	0.003219349	0.022307391	-2.466312782
ZNF788	-0.275309066	2.215460572	-2.93243849	0.004429922	0.028652789	-2.466448861
BRD3	-0.232133377	5.062141509	-3.089391455	0.002792143	0.019840017	-2.467804089
LMNB1	-0.295160745	3.308811724	-3.019993011	0.003430819	0.023396041	-2.468353839
MAPK6	0.288232382	5.687698886	3.109010439	0.002632769	0.018849827	-2.469442325
CCDC89	-0.295196881	0.54601349	-2.857064415	0.005498677	0.033698973	-2.47209026
ATP7B	-0.300425437	0.860369576	-2.887240015	0.005045112	0.031430757	-2.472588285
SEPSECS	-0.263231528	1.835738016	-2.918033391	0.004618027	0.029477103	-2.475151133
TRIP4	0.091841839	4.10445108	3.06764185	0.002979299	0.020972035	-2.476688435
TTC14	0.205943656	3.373045466	3.039584062	0.003237985	0.022385045	-2.479249721
PTGES3	0.076206611	7.489607452	3.104122867	0.00267166	0.019082927	-2.479559252
ZNF74	0.15670504	3.197678473	3.004794039	0.003587705	0.02428997	-2.480120494
TAB2	-0.125248099	6.313193869	-3.109359412	0.002630013	0.018848008	-2.480768784
CBR4	-0.17984337	2.93004788	-3.009786113	0.003535457	0.023979342	-2.483279488
FBXO2	0.487566001	-0.803420277	2.789311693	0.006656895	0.039080071	-2.483296995
C2orf68	-0.18093249	3.495585369	-3.039020311	0.003243391	0.02240187	-2.483762079
ATG12	0.094841949	5.833501327	3.105138344	0.002663536	0.019042958	-2.484098365
ZNF608	-0.434848169	3.351491789	-3.040555998	0.003228686	0.022353052	-2.486140246
C8orf48	-0.278098496	0.971417895	-2.83730456	0.005815706	0.035141513	-2.487214116
ZSWIM3	0.255033132	1.786618609	2.903685563	0.004812691	0.030410394	-2.487291448
SLC2A12	-0.303790968	1.320420976	-2.915555367	0.00465112	0.029650644	-2.488103566
ZNF44	0.203029413	1.875556354	2.917264026	0.004628278	0.029530024	-2.494831103

Gene	Log fold change	Average Expression	t	P-value	Adjusted P-value	B
ENC1	0.244712973	3.426775941	3.036274842	0.003269835	0.022522539	-2.497779247
ADPRH	-0.239937111	1.874756938	-2.940340182	0.004329771	0.028113654	-2.498049087
ELP3	-0.094649068	4.924137175	-3.079782194	0.002873439	0.020350485	-2.49900354
RABGGTA	-0.179526885	4.057980442	-3.064544887	0.003006878	0.021128593	-2.501083199
CCNY	0.07133697	6.918240905	3.100642072	0.002699683	0.019255696	-2.502905499
MUM1L1	-0.69888348	-5.016819543	-2.748427236	0.007459949	0.042374313	-2.504916804
TAF13	0.201309115	4.724611693	3.077382363	0.002894083	0.020481473	-2.506829376
CHRAC1	0.099986264	5.128420331	3.080898142	0.002863886	0.020292369	-2.518340084
CACNA1A	-0.185072702	3.584085308	-3.030934873	0.003321843	0.022797379	-2.5195457
POLQ	-0.427699706	0.242942844	-2.820530994	0.006097915	0.036538977	-2.522683837
CNEP1R1	0.157047999	3.110508026	3.003933058	0.003596788	0.024340528	-2.524061722
AP3S1	0.108005905	6.850887257	3.091104316	0.002777878	0.019750187	-2.530162519
NAP1L1	0.083881911	9.258497315	3.052377398	0.003117548	0.021752082	-2.53474302
LYST	-0.316198584	3.985456496	-3.022901244	0.003401535	0.023248966	-2.536680137
CEP83	-0.211080388	2.592401383	-2.956476339	0.004131722	0.027179151	-2.541990651
TBXA2R	-0.246895589	1.533097118	-2.904547128	0.004800792	0.030363913	-2.542404234
KIAA0586	-0.196049342	3.899922135	-3.033162861	0.003300051	0.022678819	-2.543321549
TOMM40	0.200696822	4.541808384	3.053825213	0.003104184	0.021678917	-2.544181762
CCDC112	-0.213189399	2.439224781	-2.949923963	0.004211111	0.02753306	-2.5447484
CLCN7	-0.131694101	6.699298792	-3.085943131	0.002821066	0.020026647	-2.544843728
APBB3	-0.257196184	1.370308825	-2.907778748	0.004756399	0.030194013	-2.547075128
TGFB1I1	-0.14911862	6.378691202	-3.085150082	0.002827758	0.020064693	-2.547781067
NARF	-0.145211759	4.760776728	-3.067210723	0.002983124	0.020989153	-2.548319719
WDR62	-0.306815795	1.318727238	-2.868980627	0.00531526	0.032815195	-2.550089376
KDM4C	-0.166715485	2.243529528	-2.953440361	0.004168332	0.027336419	-2.555910825
TMCC1-AS1	-0.359176396	0.165227197	-2.802382464	0.00641738	0.037969918	-2.557703907
RFLNB	0.140245703	5.864686639	3.074753431	0.002916856	0.020609531	-2.561658685
PSME4	0.27601698	5.892497415	3.07194816	0.002941341	0.020743612	-2.569072195
EMP1	0.195577347	9.382988105	3.041854619	0.0032163	0.022307391	-2.570020169
MLF1	0.152645938	2.878306687	2.971833062	0.003951053	0.026208132	-2.57195891
UBE2E1	0.150201701	6.215892852	3.074468332	0.002919336	0.020617381	-2.576454642
RABGAP1L	0.090954607	4.500055607	3.042110396	0.003213865	0.022307391	-2.577028964
SKIDA1	0.295001478	0.899748966	2.817027954	0.006158417	0.036828263	-2.577597524
LONRF2	-0.555265409	-1.353564573	-2.713529583	0.00821446	0.045898601	-2.577922557
ZNF551	-0.194353877	2.388069497	-2.940778294	0.00432428	0.028090107	-2.581068293
TAF4	-0.174790427	3.287278591	-2.972910609	0.003938655	0.026172855	-2.581598887
TNK2	-0.152372907	4.640871916	-3.045807133	0.003178871	0.022128704	-2.586663757
LCMT2	-0.127529248	3.48520111	-3.000656461	0.003631551	0.024510349	-2.588827423
H2AFV	0.138866862	6.310524485	3.069312329	0.00296452	0.020877763	-2.590044717
XYLT2	-0.157035449	4.736358876	-3.040532973	0.003228906	0.022353052	-2.591616572
MICALCL	-0.633100673	-2.512294911	-2.693985825	0.008666816	0.047823047	-2.593258598
CPSF6	0.08244835	5.621213729	3.061518136	0.003034061	0.02123843	-2.596930963
MTA1	-0.115975554	5.811480681	-3.063958935	0.003012123	0.02115298	-2.599347962
FBXL12	0.155527151	4.266317478	3.025265457	0.0033779	0.023129403	-2.59965798
NOL8	-0.126010582	5.280525483	-3.051960568	0.003121406	0.021768914	-2.599989964
YPEL5	0.071368369	6.658527068	3.065498668	0.002998359	0.021086499	-2.601451124
ARPP19	0.127211132	6.069482637	3.06323268	0.003018635	0.021163077	-2.602890348
UBE2A	0.078727696	6.269728053	3.063656489	0.003014833	0.02115298	-2.604721481
EP400	-0.229729975	5.858011994	-3.060728769	0.003041188	0.021268551	-2.605673651
SDHAF2	0.212432335	2.77992596	2.956108457	0.004136142	0.027194418	-2.60651942
HELZ	-0.244622427	4.877334989	-3.039806527	0.003235855	0.022380586	-2.606609191
NAPEPLD	-0.212612865	3.585139887	-2.984127718	0.003811712	0.025497169	-2.607236341
IMPA2	-0.199571476	3.162220125	-2.986598844	0.00378426	0.02534731	-2.609370117
PTGR2	0.184426094	1.511700204	2.867858491	0.005332289	0.032879901	-2.610164902
PIP4K2B	-0.139562873	6.795557422	-3.061875848	0.003030837	0.021225722	-2.611154455
DYNLT3	0.095693674	5.145471149	3.044804506	0.003188327	0.022177381	-2.617269406
TRAF7	-0.090509383	7.24958236	-3.055260871	0.003090984	0.021596747	-2.619880237
CNPPD1	-0.134536064	5.611593759	-3.051302468	0.003127505	0.021801359	-2.624269683
KYAT3	0.160108904	5.06290442	3.036764372	0.003265105	0.022512904	-2.626656312
TRIM33	0.324107565	4.628776701	3.022157073	0.003409006	0.023289463	-2.628165807
HINT3	-0.145111994	4.153468796	-3.019175395	0.003439094	0.023441858	-2.628922146
ZNF92	-0.183333361	2.660009373	-2.927511797	0.004493445	0.028914673	-2.629663727
PFKM	0.090216669	5.982296008	3.05295139	0.003112243	0.021725132	-2.630939957
NUS1	0.160924371	4.200855689	3.010233945	0.003530804	0.023958574	-2.630994536
GGCT	0.208769247	3.897789744	2.99922175	0.003646871	0.024558096	-2.631782842
IL19	0.925301405	-4.297540065	2.675561158	0.009113939	0.04957669	-2.635562973
RPF2	0.105518845	5.311659101	3.036728949	0.003265447	0.022512904	-2.647515309
MFSD11	0.127774064	3.819250642	2.98690257	0.003780899	0.025336061	-2.647519959
TBKBP1	-0.143876213	4.161902502	-2.999460851	0.003644314	0.024558096	-2.651550252
SWAP70	0.225250948	7.131390782	3.04204194	0.003214517	0.022307391	-2.652597075

Gene	Log fold change	Average Expression	t	P-value	Adjusted P-value	B
DET1	-0.138507666	2.602152071	-2.923212276	0.004549567	0.029163751	-2.655851485
RAF1	0.097147704	6.259120627	3.042212152	0.003212897	0.022307391	-2.664576299
RBM45	0.145556865	2.892165106	2.936324605	0.004380403	0.028369039	-2.665402248
UBA7	-0.116470809	5.796802263	-3.038857045	0.003244958	0.02240187	-2.665695892
CCDC86	0.192119641	4.668601748	3.005315888	0.00358221	0.024263672	-2.677728381
ABCC5	-0.235290393	3.279559744	-2.954374759	0.004157032	0.027274189	-2.677918091
LARP4	0.283415294	5.145740453	3.024761351	0.003382927	0.023153298	-2.67883691
SULT1B1	-0.518108653	-0.890236848	-2.698123213	0.008569201	0.047423379	-2.679001482
RNF6	-0.139691115	5.070535474	-3.024490362	0.003385632	0.023161289	-2.679823871
MID1IP1	0.121669246	5.248961084	3.025692188	0.00337365	0.023110809	-2.681203833
STK11IP	-0.122696583	3.609246095	-2.965192355	0.004028263	0.026614805	-2.68129809
BCAP29	0.095423703	6.008662888	3.033455541	0.003297198	0.022669555	-2.682195838
HAS3	0.546602227	-1.67712824	2.682817736	0.008935391	0.048893109	-2.683150533
XKR8	0.175352236	2.774954883	2.895830989	0.004922437	0.030948079	-2.687119638
JKAMP	0.187477871	4.33498359	3.000647665	0.003631645	0.024510349	-2.687350679
NKAPL	-0.351672335	0.015224228	-2.746342109	0.007503188	0.042571758	-2.689328211
HEATR3	-0.20490328	3.43733902	-2.947868656	0.004236302	0.027649763	-2.691029329
FLAD1	-0.163554702	3.921926493	-2.974576471	0.003919558	0.02606809	-2.692078954
PEX14	-0.16973441	3.954841805	-2.981891184	0.003836716	0.025618922	-2.692727358
INTS1	-0.123914576	6.969495228	-3.03076099	0.00332355	0.022798702	-2.693249575
SUOX	-0.121803397	3.940767992	-2.987247642	0.003777084	0.025321756	-2.694040921
ACVR1C	-0.5218896	-1.251778051	-2.683779098	0.008911977	0.048782711	-2.694671426
HAUS3	0.23308455	2.308327909	2.873632384	0.005245201	0.032475833	-2.694914107
SCAMP1-AS1	0.301179078	-0.001218142	2.737428152	0.007690627	0.043504168	-2.695399918
CENPI	-0.327976244	0.98074533	-2.79822993	0.006492602	0.038309659	-2.697373685
ARL4A	-0.289848336	2.033279854	-2.904363935	0.004803319	0.030363913	-2.701368925
DFFA	-0.095955065	5.329400508	-3.01827453	0.003448232	0.02349352	-2.701715737
DPH2	0.164950595	3.731903902	2.956982192	0.004125651	0.027151071	-2.702735252
CHEK1	-0.165857133	3.678873952	-2.966068483	0.004017997	0.026558627	-2.70381311
DTNBP1	-0.198104202	3.781533889	-2.976284122	0.003900071	0.025949945	-2.705293248
LOC105378753	0.37085174	-1.01622841	2.682264833	0.008948882	0.048922712	-2.705400847
GPAT4	-0.106506556	6.088295047	-3.026018281	0.003370406	0.02309909	-2.705951025
NUP58	0.261971793	4.356560612	2.988775059	0.003760237	0.025231275	-2.707794221
HJURP	-0.361349928	1.620401551	-2.818574703	0.006131635	0.036682664	-2.712069726
SEMA4G	-0.295077834	0.190312044	-2.748811224	0.007452012	0.042345192	-2.71222398
ZWILCH	0.159655346	3.50706922	2.944788615	0.004274314	0.027861649	-2.712617505
STX12	0.116264462	6.341301696	3.023535564	0.003395179	0.023216056	-2.715590521
TMEM229B	-0.201809213	1.304708493	-2.81272009	0.006233575	0.037130319	-2.721311374
KBTBD4	-0.151430572	2.611367335	-2.892506442	0.00496958	0.031123059	-2.72440101
CAPN10	-0.212931092	2.319472304	-2.893764598	0.004951691	0.031054233	-2.726717357
VPS37D	0.230694613	1.784647896	2.822036092	0.006072088	0.036427689	-2.729029833
LIMS1	0.156853102	6.51713682	3.016246026	0.003468893	0.023591606	-2.735524856
TBC1D8	-0.189367646	4.023022301	-2.970762072	0.003963412	0.026255427	-2.738411381
LRRC49	0.126941681	3.42588736	2.938537516	0.004352433	0.028236459	-2.739146177
TSPYL2	0.161546478	4.562315945	2.97289926	0.003938785	0.026172855	-2.740664112
MLH3	-0.172347669	2.875657819	-2.895502667	0.004927075	0.030964311	-2.740953513
C2CD3	-0.179028259	3.703373678	-2.951513063	0.004191728	0.027418236	-2.742231732
DNAJC6	0.433595356	-0.046261575	2.706034292	0.008385335	0.046697484	-2.743129845
SCP2	0.074873236	7.026234992	3.012150046	0.003510962	0.023834671	-2.743150034
FAM3C	0.167320995	4.078546492	2.957057471	0.004124748	0.027151071	-2.7490935
RAD51D	-0.098321394	3.479534448	-2.933809445	0.004412394	0.028551674	-2.751992946
NUDT5	0.089315676	5.583703083	3.003485925	0.003601514	0.024361563	-2.753153414
OPRL1	-0.251976735	0.810471724	-2.791887537	0.006609055	0.0388598	-2.753219914
CHORDC1	-0.181704337	3.79825457	-2.92941363	0.004468824	0.028817794	-2.753876407
MOB2	-0.157102571	4.081970548	-2.958159742	0.00411155	0.027081938	-2.754861479
SRSF2	0.155425212	5.800198373	2.999353084	0.003645466	0.024558096	-2.760647492
PRRC1	0.242381578	6.584779433	3.00631083	0.003571754	0.024203738	-2.762856621
UBALD1	-0.217287485	4.54506132	-2.98472118	0.003805102	0.025468758	-2.763568974
KBTBD6	0.420194908	3.732222741	2.915075821	0.004657549	0.02967907	-2.76439649
SUPT5H	-0.076366267	7.262362968	-3.000825921	0.003629746	0.024510349	-2.76601172
CMTM7	-0.196200321	3.297640628	-2.920797938	0.004581365	0.029307794	-2.769685814
ALPK1	-0.157485131	3.877307704	-2.95555306	0.004142824	0.027216528	-2.773538699
CNOT9	0.11654516	5.654598282	2.994840775	0.003694021	0.024820132	-2.773936552
RNF2	0.112372386	4.637927281	2.972264384	0.003946086	0.026198256	-2.774957939
FAM189B	-0.144928132	5.966650076	-2.999388868	0.003645083	0.024558096	-2.775122681
TDRD7	-0.139944846	3.402196351	-2.925090047	0.004524977	0.029055521	-2.775424833
SPAG1	0.194089596	1.672114102	2.815348967	0.00618761	0.036944179	-2.775490353
MPZL1	0.099352502	7.578806341	2.994394221	0.003698859	0.024841556	-2.778336238
SIRT5	-0.174184159	2.359409703	-2.867407596	0.005339145	0.03289525	-2.778534838
TMEM187	-0.174532459	2.565678547	-2.886314502	0.005058495	0.031501103	-2.778685118

Gene	Log fold change	Average Expression	t	P-value	Adjusted P-value	B
USP36	0.178264834	5.900659722	2.997777279	0.003662355	0.024629337	-2.78177797
MYBL1	-0.34158146	0.815617791	-2.741951329	0.007594987	0.04301161	-2.783936568
ZBTB18	0.210614608	2.312806471	2.852189506	0.005575378	0.034030589	-2.786234948
ZNF316	0.146399529	4.86444271	2.979847156	0.003859701	0.025760979	-2.787083244
ZFHX4	-0.323090986	4.614277019	-2.964117918	0.004040886	0.026686499	-2.78724037
MRM2	0.092339439	4.44812478	2.959753294	0.004092539	0.026980313	-2.787512975
MTF2	-0.171801999	2.718002735	-2.861462638	0.005430311	0.033375033	-2.787871698
RALGAPA2	-0.350486834	1.793719229	-2.852926525	0.005563719	0.033986968	-2.788266288
PIK3CD	-0.214202136	3.171888928	-2.88725733	0.005044862	0.031430757	-2.788866247
RBM38	0.179613317	3.228707059	2.893025376	0.004962194	0.031107157	-2.789645901
SLC25A19	-0.211767459	1.40027883	-2.780254248	0.006827644	0.039803265	-2.790591883
EIF2B4	-0.153455411	4.922494569	-2.970218089	0.003969703	0.026273993	-2.798529819
PNPT1	0.13525395	4.003010283	2.942516749	0.004302554	0.027997269	-2.800270616
NBAS	-0.143637385	6.335277839	-2.992113803	0.003723655	0.024996944	-2.800731405
AK2	0.085517683	5.600294725	2.983730944	0.003816137	0.025515438	-2.806063498
FAM50B	-0.219378265	2.674194203	-2.87143325	0.005278215	0.032619093	-2.80790876
MAP6D1	0.192734457	1.426853227	2.760871469	0.007206603	0.041356294	-2.811367354
ZBTB45	-0.126949915	4.443433278	-2.954440309	0.004156241	0.027274189	-2.813503766
RNASEL	-0.174116687	4.22633604	-2.941582493	0.004314217	0.02804893	-2.815721137
IL4R	-0.125076511	5.491598057	-2.977778999	0.003883086	0.025859776	-2.816363128
CCDC85B	-0.277104299	6.94206842	-2.983353837	0.003820346	0.025520931	-2.81763525
MRPL42	0.136432331	4.912236111	2.963664847	0.00404622	0.026710014	-2.820998272
SUV39H2	0.215874439	2.765080786	2.85220089	0.005575198	0.034030589	-2.824355113
RAB6A	0.090161589	6.573474711	2.98337035	0.003820162	0.025520931	-2.825034643
POLR2K	0.112286031	4.878603514	2.96023373	0.004086824	0.026966235	-2.825463788
FAM219A	-0.132447139	3.77377259	-2.920018642	0.004591672	0.029346183	-2.825567907
KIAA1549	-0.265523639	3.858527508	-2.920766925	0.004581775	0.029307794	-2.825996407
UBAP2L	-0.090888307	7.63042235	-2.977014812	0.003891761	0.02590609	-2.826906095
DIABLO	0.177359115	2.227421616	2.824396307	0.006031789	0.036229215	-2.830682734
TSNAX	0.152148538	4.106706851	2.928230122	0.004484131	0.028904127	-2.831461602
AKTIP	0.153052339	3.049747786	2.892561688	0.004968794	0.031123059	-2.83248998
ZNF720	-0.131944391	2.762949983	-2.871688757	0.005274369	0.032619093	-2.833632068
TFG	0.080724042	6.724681239	2.979495966	0.003863662	0.025764591	-2.835378563
ZNF324	0.151054902	3.065314411	2.878890216	0.005167038	0.032057766	-2.836447541
CHST2	0.147359879	3.762249205	2.904451285	0.004802114	0.030363913	-2.841237735
TMEM120B	-0.215602242	2.675703675	-2.847692013	0.005647012	0.034342621	-2.842003224
ATXN7	-0.219467881	3.938032486	-2.9265986	0.004505311	0.028966284	-2.84539477
TRA2B	0.098349306	6.108099213	2.971943045	0.003949786	0.026208132	-2.85280481
CD55	0.100830226	5.940924662	2.971227398	0.003958038	0.026231362	-2.852814882
RNF31	0.176071543	3.693056092	2.891008957	0.004990951	0.031209499	-2.852845507
ZNF653	-0.234715545	0.736641585	-2.725542793	0.007947203	0.044703761	-2.8546487
PPP1R3G	0.204642654	2.588271953	2.823260051	0.006051159	0.036316597	-2.85770189
SYNE1	-0.199391915	6.000703597	-2.968544989	0.00398911	0.026384467	-2.857746112
NAA25	0.272783276	3.741589155	2.890673655	0.004995747	0.031213579	-2.858179303
ANGEL1	-0.190620674	3.175233398	-2.883248132	0.005103068	0.031713125	-2.859181598
SLC37A1	-0.254543938	0.11899011	-2.696232811	0.008613677	0.04759957	-2.863146548
MAP2K4	0.120383617	4.519402397	2.934637136	0.004401842	0.028495639	-2.868430495
INTS10	0.091351827	5.112659668	2.954899904	0.004150694	0.027256347	-2.868575193
LINC00662	-0.176508605	2.523012358	-2.84557645	0.005681	0.034479686	-2.869979889
RNF11	0.097220187	7.481118996	2.960071954	0.004088748	0.026967117	-2.873743275
CNOT4	0.147728597	4.599041802	2.929992579	0.004461354	0.028781943	-2.879388732
FANCA	-0.290201918	0.966374697	-2.712818291	0.008230539	0.045937322	-2.879499434
KLHL22	-0.122491904	4.595273876	-2.928018706	0.00448687	0.02890911	-2.886986827
FAM214B	-0.167266023	4.182907705	-2.90708018	0.004765963	0.030216526	-2.888256555
WDR41	-0.096423331	5.296737261	-2.948893046	0.004223729	0.02759161	-2.889077425
HARBI1	-0.231205576	1.552577725	-2.736940399	0.007701005	0.043546526	-2.890036218
ZMIZ1	-0.216696359	8.005195612	-2.951838298	0.004187772	0.027416164	-2.890309224
EIF3E	0.115610354	8.932636912	2.925886412	0.004514585	0.02901353	-2.891493094
DDX19B	0.164768623	3.043409225	2.861016579	0.005437209	0.033403798	-2.895272407
ZNF407	-0.219311984	4.171562389	-2.890729127	0.004994954	0.031213579	-2.895326404
SMARCC2	-0.10157008	6.136754647	-2.955982789	0.004137653	0.027194418	-2.896879845
MRTO4	0.128774101	5.17576301	2.937444916	0.004366222	0.02831372	-2.900678528
NKTR	-0.292730836	3.636434421	-2.882622095	0.005112212	0.031753904	-2.901977877
TMEM44	-0.175882275	3.784954949	-2.900784804	0.004852956	0.030613453	-2.902556722
MAPK1IP1L	0.109279122	6.291143158	2.952952833	0.004174239	0.027351342	-2.905807198
RAC1	0.068623811	7.833008801	2.942041898	0.004308479	0.028023714	-2.90792164
CHPT1	0.092240873	5.60030047	2.944599293	0.004276661	0.027864889	-2.908178257
TMEM50A	0.093056625	7.375077388	2.946606644	0.004251839	0.027739153	-2.911946172
ZNF430	0.231368203	2.358766109	2.783625412	0.006763628	0.039518697	-2.916943852
EXOC2	-0.114988721	4.851660866	-2.927545818	0.004493003	0.028914673	-2.917249996

Gene	Log fold change	Average Expression	t	P-value	Adjusted P-value	B
FBXO46	0.151738733	2.581617724	2.807903488	0.006318606	0.037532916	-2.920735368
PGS1	-0.1362853	3.820448266	-2.891174398	0.004988586	0.031209499	-2.924109303
ZNF675	0.235786038	1.479333014	2.718714094	0.008098131	0.045390836	-2.924391404
ZBTB2	0.096129147	5.518951245	2.93720138	0.004369301	0.028321494	-2.92610269
PSMA4	0.120419247	6.54177769	2.944214842	0.00428143	0.027883902	-2.92965327
MIR4458HG	0.1929502	1.967177815	2.789352863	0.006656128	0.039080071	-2.931584503
TRIM4	-0.103313131	5.386791152	-2.930921116	0.004449397	0.028754063	-2.933473529
SLC20A2	0.219981444	5.443241045	2.922583991	0.004557822	0.029191766	-2.934017926
CCDC102A	-0.192348564	3.690003772	-2.877613348	0.00518592	0.032148438	-2.935177253
TNRC6A	0.26139665	5.233742466	2.922422724	0.004559943	0.029192939	-2.936122279
EVA1A	0.220651418	4.040942249	2.908827686	0.004742071	0.030128453	-2.93926888
ASPA	0.183864881	2.585043415	2.803763961	0.006392532	0.03786752	-2.940115507
GLT8D1	0.07585875	6.13484407	2.938695727	0.00435044	0.028235687	-2.942363339
CHKA	0.186656641	3.453655623	2.84312013	0.005720698	0.03466476	-2.944506426
ARRDC1-AS1	-0.191444136	2.304221326	-2.788488316	0.006672254	0.039154978	-2.944902277
C19orf48	0.11651963	4.472322093	2.90283196	0.004824508	0.030472278	-2.945101347
H3F3A	0.291193212	0.730398521	2.675390206	0.009118184	0.04957669	-2.95115739
SRSF10	-0.100830774	5.639692449	-2.930088899	0.004460112	0.028781943	-2.951762198
ZNF160	0.225888843	4.132474462	2.877695375	0.005184705	0.032148438	-2.953802012
DHFR2	-0.140027532	3.266192863	-2.853485678	0.005554889	0.033967893	-2.95493858
ASB8	-0.101461707	4.465932067	-2.894234507	0.004945024	0.031025342	-2.958333068
POMGNT1	-0.120335416	5.241608701	-2.922828077	0.004554613	0.029183623	-2.959238006
GOLGA5	0.07875004	6.028088836	2.930373752	0.004456442	0.028781943	-2.96023453
BAMBI	0.338298623	1.101114496	2.68825186	0.008803776	0.048401491	-2.960571592
ZSCAN31	-0.305936743	0.756172308	-2.677596942	0.009063524	0.049345814	-2.961627975
RASAL2	0.306969632	5.004305336	2.89787203	0.0048937	0.030805972	-2.96174226
ACTA2	-0.13513835	6.35846942	-2.93200723	0.004435449	0.028676226	-2.961785006
PTBP1	0.079317194	7.729849333	2.924905526	0.004527388	0.029058616	-2.962338601
LYRM4-AS1	-0.177896006	2.226678347	-2.779313811	0.006845601	0.039846268	-2.96330642
C18orf32	-0.238497294	1.267396826	-2.701589475	0.008488192	0.047113531	-2.965540631
KIAA1328	-0.211463764	1.627271401	-2.751273443	0.007401297	0.042136484	-2.967507228
PHIP	-0.26265563	4.657955157	-2.898055946	0.004891118	0.030805972	-2.967525854
RETSAT	-0.095242086	5.984234506	-2.926616207	0.004505082	0.028966284	-2.971790139
RPGR	0.172978406	1.789116968	2.758727902	0.007249674	0.041508586	-2.97184841
INTS9	-0.111537733	3.83151495	-2.871370109	0.005279165	0.032619093	-2.973746588
PTRH2	0.167880223	4.238067202	2.884736409	0.005081389	0.031630598	-2.975008416
IRGQ	0.265985156	3.743825883	2.865198945	0.005372849	0.033059776	-2.975891118
NFIL3	-0.174934107	5.801989246	-2.925125897	0.004524509	0.029055521	-2.976510667
SMIM10L1	0.157180574	4.699172725	2.901346338	0.004845137	0.030589751	-2.977754362
PRKD2	-0.123491872	4.668302801	-2.889009021	0.005019624	0.031323783	-2.978818388
ENOPH1	0.114258759	5.071200373	2.907475193	0.004760553	0.030207652	-2.980082769
ARAF	-0.102551077	5.751858913	-2.920145907	0.004589988	0.029346183	-2.981621249
ZNF121	0.436569748	3.244370775	2.844600923	0.005696735	0.034539598	-2.983289186
AKAP10	-0.182698924	3.946106452	-2.862216425	0.005418673	0.033317098	-2.986615517
CNTRL	-0.198471249	2.332803689	-2.781796139	0.006798296	0.039662873	-2.987353253
PDE12	0.1744545	4.810012686	2.899807072	0.004866597	0.030683812	-2.988023186
TRMT10C	0.13187431	4.573374928	2.896964529	0.004906458	0.030873386	-2.99010569
SH2B3	0.207915845	5.366836049	2.910065113	0.00472522	0.030046737	-2.991233844
C17orf97	-0.288916672	1.192324686	-2.672105221	0.009200109	0.049888567	-2.991730174
ABI2	-0.091219466	5.461755579	-2.909603799	0.004731496	0.030073948	-2.996709154
NAA40	-0.18027178	2.827024219	-2.808633182	0.006305656	0.037470769	-2.997857547
RFC5	0.145989095	2.778875903	2.80411625	0.00638621	0.037865509	-2.999755041
ADGRB2	-0.137716391	3.787978763	-2.853396397	0.005556298	0.033967893	-3.002309166
ZDHHC8	-0.088442937	5.922642323	-2.913806461	0.004674607	0.029775173	-3.003398283
DNAJC27	0.254082199	1.988949786	2.720705462	0.00805385	0.045202381	-3.00472239
ZNF597	-0.188334662	2.451860231	-2.779354384	0.006844826	0.039846268	-3.005842442
PLEKHG2	-0.150009631	4.956632951	-2.896207651	0.004917122	0.030927571	-3.008415317
H2AFZ	0.138706772	6.00087707	2.912335532	0.004694446	0.029876272	-3.008956113
DDX18	0.165496652	5.681502694	2.905113313	0.004792986	0.030349536	-3.010336729
SLC25A43	0.09426395	5.142185242	2.897973	0.004892282	0.030805972	-3.010543068
TNPO2	-0.145249554	5.985155998	-2.910392753	0.004720768	0.030031102	-3.011008096
WHAMM	0.200767016	2.558693917	2.779886449	0.006834662	0.039828763	-3.013541431
MDH1	0.090831073	6.580344457	2.912401986	0.004693548	0.029876272	-3.013836874
ZNF133	0.156948009	2.821633821	2.769762213	0.007030449	0.040654703	-3.014576704
FAM161A	-0.20107015	1.333315331	-2.686722773	0.008840631	0.048550954	-3.014770959
TGDS	0.155726646	2.305355821	2.753925256	0.00734703	0.04189037	-3.017862607
MAGOH	0.201415295	4.065120779	2.860023075	0.005452601	0.033484706	-3.022696569
CASC4	0.127930945	6.669405792	2.90852569	0.004746192	0.030141923	-3.023710469
ITM2B	0.064380068	8.548015968	2.882510385	0.005113846	0.031753904	-3.029057822
CKAP5	-0.174340819	6.776980433	-2.906103056	0.00477937	0.030276045	-3.029863591

Gene	Log fold change	Average Expression	t	P-value	Adjusted P-value	B
AP3S2	-0.115501554	4.389465748	-2.868534925	0.005322018	0.032843455	-3.032214665
ARL6IP5	0.082316803	8.315628698	2.883810723	0.005094863	0.031675203	-3.035509723
DIP2A	-0.192619612	4.319463277	-2.868030467	0.005329676	0.032877247	-3.035538249
ARID4A	-0.30127751	3.671345398	-2.82018864	0.006103803	0.036545189	-3.036927901
SQSTM1	-0.10008297	9.052085219	-2.86745161	0.005338476	0.03289525	-3.039590089
MKNK1	0.129360716	3.842752584	2.853953066	0.005547518	0.033943057	-3.039659327
ZFP36L1	0.214485138	8.824299119	2.870115115	0.005298095	0.032722631	-3.043307933
LAMTOR3	0.089296759	5.231015097	2.887609661	0.005039776	0.031423506	-3.045153944
GTF2A2	0.165642873	4.92176147	2.88016436	0.005148259	0.031954416	-3.046765
RPIA	0.127474062	3.82582972	2.835727165	0.005841724	0.035270435	-3.047288765
ORAI3	-0.197258234	3.935113285	-2.840672888	0.005760503	0.034863887	-3.047913775
TMCO6	-0.202027549	1.726678436	-2.706170166	0.008382209	0.046697337	-3.048658847
THAP12	0.153098025	4.413666259	2.858129467	0.00548205	0.033610744	-3.052344373
LOC100129940	-0.196724587	2.07477141	-2.734616619	0.007750628	0.043794247	-3.053519297
CABLES1	-0.162529731	5.350006316	-2.889222338	0.005016558	0.031317626	-3.055888419
PIEZO1	-0.148036471	7.347436478	-2.891142455	0.004989042	0.031209499	-3.058264843
PSMC2	0.108579876	6.331468364	2.894625475	0.004939484	0.031007625	-3.058480236
CAPZA1	0.104644339	6.69996793	2.894579026	0.004940142	0.031007625	-3.06051324
FAM107B	0.242234095	4.682990478	2.865082757	0.005374628	0.033059776	-3.065345988
ZNF444	-0.190194061	4.009107817	-2.839083921	0.005786484	0.035007065	-3.065424158
BDKRB1	0.26822621	4.050191758	2.84941224	0.005619514	0.034216796	-3.066395
RARS2	0.081273202	5.567643218	2.883955323	0.005092756	0.031675177	-3.067348212
ZNF225	0.193649991	2.83235177	2.758029699	0.007263754	0.041543927	-3.06783214
PPIA	0.140121117	6.187815449	2.889506186	0.005012482	0.031305151	-3.070860629
WDR53	0.158349784	2.208256013	2.728232019	0.007888475	0.044456409	-3.073186088
ARHGAP22	-0.195762089	5.005111097	-2.867251041	0.005341528	0.032896475	-3.073484842
GLIS2	0.142959765	6.677582351	2.888809342	0.005022495	0.031328721	-3.074877555
CFAP69	0.178771982	1.854568087	2.7151647	0.008177608	0.04574359	-3.077080632
TMEM241	-0.179647434	2.033658324	-2.727206723	0.007910819	0.044548999	-3.080978048
URB2	0.34661474	3.619618995	2.823724091	0.006043242	0.036283536	-3.081435847
SHPRH	-0.433356853	2.133883004	-2.706632501	0.00837158	0.046655374	-3.082012634
GLE1	-0.123896232	5.053297747	-2.866862063	0.005347452	0.032919501	-3.087290599
ID3	0.322283394	5.613528326	2.884153557	0.005089869	0.031670296	-3.087399791
DENND4C	-0.341473119	4.626380159	-2.844856101	0.005692615	0.034536271	-3.089158811
FITM2	-0.239618016	3.640306377	-2.814465155	0.006203029	0.037021563	-3.094393075
HDDC3	-0.174087123	1.935764062	-2.715426939	0.008171712	0.045737608	-3.095745197
REXO4	-0.100913417	4.968767575	-2.858548478	0.005475521	0.033598064	-3.09657037
ETAA1	-0.176037695	3.007119561	-2.766645191	0.007091754	0.04088411	-3.097037694
TAF1	-0.287214388	4.297093765	-2.833573647	0.005877418	0.035457521	-3.098403978
NT5DC3	0.294657963	1.838438926	2.713175833	0.008222453	0.045912586	-3.100139871
C19orf25	-0.139539541	3.626277342	-2.803909622	0.006389917	0.037866922	-3.10268341
CTDP1	-0.094891326	4.631337486	-2.847549403	0.005649297	0.034342621	-3.10322434
SYF2	0.144163805	5.929244977	2.875057989	0.005223901	0.032370564	-3.104353961
ACTL6A	0.083511807	4.868959842	2.853266947	0.005558342	0.033967893	-3.10967347
DCLRE1B	0.167690408	3.266535632	2.770451336	0.007016962	0.040608341	-3.116078852
TAP2	0.1213191	3.382862339	2.782870411	0.006777917	0.039559297	-3.116181775
NPM1	0.128212587	8.004633134	2.855941634	0.005516256	0.033792958	-3.124898769
RAD54L2	0.228799802	3.904257645	2.800313112	0.006454765	0.03816114	-3.125231027
MOV10	-0.093917241	4.933693466	-2.847615341	0.005648241	0.034342621	-3.132840328
FAM13B	-0.215333279	5.065322747	-2.851975963	0.00557876	0.034032929	-3.133604906
SNRNP27	0.12950304	4.236216915	2.827357365	0.005981579	0.035999405	-3.136634688
STARD9	-0.306291729	2.480107704	-2.701078402	0.008500092	0.047162204	-3.138214093
RFTN2	-0.179221525	3.17546814	-2.780562309	0.006821772	0.039784424	-3.138673019
RNF138	0.135623441	3.544573481	2.801126063	0.006440055	0.038089117	-3.139621139
TIMM8A	0.175418518	2.062067305	2.681521121	0.008967059	0.048995201	-3.140163242
ANKMY2	-0.145911609	4.288890374	-2.825928388	0.006005762	0.036088705	-3.141263972
SLC25A38	0.08461586	5.070807248	2.845743582	0.005678308	0.034477239	-3.142586336
ELAC2	-0.08812187	5.660476503	-2.851880059	0.00558028	0.034032929	-3.15526033
ZBTB25	-0.173159037	2.22335341	-2.692589173	0.008699996	0.047953437	-3.158625219
PSIP1	0.088856367	6.136290999	2.8553148	0.005526093	0.033825706	-3.159988186
UAP1L1	-0.146205922	5.523121159	-2.847558619	0.00564915	0.034342621	-3.160945273
RUNX1	-0.278924202	7.370506412	-2.842196888	0.005735685	0.034741599	-3.163394315
PAQR8	-0.185666667	3.454431885	-2.774194582	0.006944111	0.040279585	-3.165150607
FUT10	-0.244931446	3.377334006	-2.758587054	0.007252512	0.04150906	-3.16627118
GTPBP2	0.208024305	4.262402475	2.813104468	0.006226835	0.037104843	-3.173011827
MIA3	-0.137205024	6.830592882	-2.850778535	0.005597761	0.034111921	-3.173394498
CNST	0.207322842	4.559824355	2.831565441	0.005910885	0.035645142	-3.175159236
SLC2A3	-0.201641408	5.69424123	-2.850036007	0.005609573	0.03417008	-3.176423907
MFSD14B	0.125674884	5.44906755	2.838507934	0.005795928	0.035036061	-3.177455442
ACACB	-0.199508831	2.376808116	-2.709181723	0.00831319	0.046364272	-3.180915816

Gene	Log fold change	Average Expression	t	P-value	Adjusted P-value	B
FAM89A	0.190287538	2.156780494	2.687453942	0.008822991	0.048489431	-3.184694105
SP100	0.08344221	6.251728385	2.846262807	0.005669953	0.034440389	-3.185819141
SMG6	-0.216518655	5.2030081	-2.825914356	0.006006	0.036088705	-3.193790687
C7orf73	0.104205549	5.737698891	2.836799505	0.005824025	0.03517767	-3.197269537
MKS1	-0.170180501	2.206294515	-2.713149032	0.008223059	0.045912586	-3.201931248
PRDM10	0.275390657	3.067861748	2.719230638	0.008086623	0.045352529	-3.202332843
SGMS1	0.131312028	4.69077833	2.819490516	0.006115828	0.036602635	-3.202714843
ZFAT	-0.104134692	3.585641502	-2.764917934	0.007125935	0.041019059	-3.205262979
RABGGTB	0.126554061	4.330407765	2.798710403	0.006483857	0.038277315	-3.207411266
ASB13	0.168736957	3.853794379	2.759358229	0.007236984	0.041499023	-3.207570952
MTMR4	-0.201517789	4.293015998	-2.797060297	0.006513935	0.038405449	-3.208773549
DCAF10	-0.127077266	5.411794681	-2.827426043	0.005980419	0.035999405	-3.20877367
MARCKS	0.083817301	8.796832226	2.804062993	0.006387165	0.037865509	-3.2092558
PEX11B	-0.12913308	4.164759556	-2.772671921	0.006973661	0.040419863	-3.209596464
TSPAN14	-0.153317326	5.552408422	-2.830506012	0.00592861	0.035723433	-3.210514499
TOMM34	0.117049304	5.009159278	2.81381493	0.006214394	0.037074708	-3.211520072
TCEAL9	0.09251577	6.860264701	2.835075769	0.0058525	0.035321338	-3.214486895
LRR1	0.165238621	2.383012946	2.678477823	0.009041788	0.04930696	-3.214749389
MTURN	0.110094373	5.817215434	2.830805538	0.005923594	0.035707489	-3.21630576
RUSC1	-0.119137837	5.049675164	-2.81606984	0.006175061	0.036883868	-3.216914357
EVI5L	-0.122346576	5.2497045	-2.821530664	0.006080749	0.036465131	-3.219657568
TRPV4	-0.181044312	2.061267668	-2.678453721	0.009042382	0.04930696	-3.220249161
EIF4ENIF1	0.168533263	4.348288526	2.78543927	0.006729412	0.039392236	-3.221472386
NOL10	0.095677311	4.997356789	2.808896635	0.006300986	0.037457797	-3.226936947
FDX1	0.130910169	3.872449527	2.777416317	0.006881965	0.040026994	-3.226944998
SORBS3	-0.19319959	7.625547937	-2.820274428	0.006102327	0.036545189	-3.227517135
GRK5	-0.149895135	4.616647061	-2.799270453	0.006473678	0.038257937	-3.228788024
PRKCE	-0.223338839	4.232242803	-2.792954344	0.006589334	0.038758975	-3.230371756
CDK13	0.147736784	5.468153652	2.821246469	0.006085625	0.036479846	-3.230499406
VAC14	-0.093778675	6.429979329	-2.828487586	0.005962516	0.035913371	-3.232118309
PCNT	-0.17376192	4.826850846	-2.804870713	0.00637269	0.037809451	-3.233349077
NFIX	-0.163105111	9.841390167	-2.774216965	0.006943677	0.040279585	-3.235040106
GRAMD3	0.209739122	5.098709264	2.816669065	0.006164647	0.036850887	-3.236359165
NCBP2	0.091981201	6.311691691	2.826182062	0.006001463	0.036088705	-3.237241632
TRAM2	-0.246950704	8.080176026	-2.809482561	0.006290613	0.037425667	-3.242698198
RFXANK	-0.170945277	3.880620562	-2.766882765	0.007087064	0.040872716	-3.251494724
POLR1A	-0.201334679	6.056110757	-2.816121674	0.006174159	0.036883868	-3.252040615
MLX	-0.096018685	4.873077169	-2.798670696	0.00648458	0.038277315	-3.252571434
PDE4D	-0.324149221	2.272025149	-2.736787553	0.00770426	0.043548585	-3.253494807
RPGRIP1L	-0.17418952	4.013653497	-2.767500101	0.007074891	0.040833778	-3.254153138
EIF2AK3	0.323428487	4.190940312	2.754987473	0.007325395	0.041814987	-3.255988607
POLE3	0.090265471	5.232075234	2.802607993	0.006413317	0.037969918	-3.256021236
BAIAP2-AS1	-0.100815388	4.947990367	-2.79686175	0.006517563	0.038411804	-3.257876217
OSBPL6	-0.213730552	3.195223668	-2.704269512	0.008426037	0.046854862	-3.264728525
UPRT	-0.113163228	2.958363393	-2.707465286	0.008352464	0.046566068	-3.267179755
HBP1	-0.11808729	5.95132955	-2.811912961	0.00624775	0.03719637	-3.267478874
CAV2	0.137562365	5.499013489	2.805199617	0.006366804	0.037789414	-3.270807333
ARRDC4	0.338339943	4.172866516	2.752130973	0.007383708	0.042068148	-3.271976072
GPT2	-0.153867605	3.913298251	-2.750248278	0.007422374	0.042208623	-3.278358136
ZNF142	-0.161345549	4.11726683	-2.747846163	0.007471976	0.042410642	-3.279723958
VPS37C	0.096045591	4.539893406	2.784504009	0.006747035	0.039455487	-3.283610411
DHX16	-0.139773938	5.035693197	-2.784978272	0.006738093	0.039418501	-3.284212761
WDR48	-0.11307229	5.220842343	-2.792986817	0.006588735	0.038758975	-3.287321386
CREBBP	-0.24632735	6.552066335	-2.806445446	0.006344554	0.037672198	-3.288633734
ITGA5	-0.133895779	8.743676506	-2.774371587	0.006940683	0.040279585	-3.299482421
VEZF1	-0.236531761	4.949190974	-2.783648206	0.006763197	0.039518697	-3.300183137
RRP9	0.168469802	4.477660116	2.759746849	0.007229171	0.041470005	-3.300348094
AP1B1	-0.083075892	5.995006169	-2.79911933	0.006476423	0.038259152	-3.300920773
PCID2	0.08600199	5.128347447	2.786532044	0.006708875	0.039310077	-3.302116692
MAP2K7	0.082698203	4.705857119	2.772171377	0.0069834	0.040460744	-3.302221547
FAM83G	0.200196405	3.792874725	2.757951167	0.007265339	0.041543927	-3.303288203
AASDHPPT	0.082276681	4.728699671	2.771140721	0.007003493	0.040553173	-3.304065838
TSN	0.090184836	6.157232077	2.797908866	0.006498452	0.038329161	-3.306847734
MAP4K3	0.200069255	4.112261692	2.747997527	0.007468841	0.04240883	-3.312294199
EXTL2	0.1348422	4.69898131	2.764798134	0.007128311	0.041019059	-3.312933916
NPRL3	-0.153361783	5.927387028	-2.794717226	0.006556865	0.038609624	-3.313205077
TMEM248	0.08133921	7.099738069	2.794610967	0.006558818	0.038609624	-3.315044445
RAPGEF6	-0.205109848	3.240990295	-2.710947537	0.008272963	0.046157005	-3.316108787
ADAL	-0.123455608	2.854314826	-2.685524854	0.008869602	0.048621428	-3.316807699
PPP3R1	0.123619778	4.977860866	2.774630588	0.00693567	0.040277154	-3.31793496

Gene	Log fold change	Average Expression	t	P-value	Adjusted P-value	B
DYNC2LI1	0.173522863	3.59506619	2.732372531	0.007798826	0.044033553	-3.318228167
EIF3B	0.077571612	7.872466218	2.785355516	0.006730989	0.039392236	-3.31891343
RHOBTB2	-0.095043977	4.862865882	-2.769628494	0.007033069	0.040654703	-3.320469588
PLXNA2	-0.29816217	3.387141399	-2.70227501	0.008472253	0.047059744	-3.320504742
ANAPC16	0.125786584	5.670623433	2.789679195	0.00665005	0.039070341	-3.320563401
PSMD7	0.083053668	5.283024331	2.776743217	0.006894906	0.040086788	-3.325765607
RHEB	0.141046387	5.002864234	2.768983199	0.007045725	0.040696627	-3.326414989
GCN1	-0.145709774	6.938106523	-2.79077958	0.006629594	0.038965354	-3.32782184
MAPKAPK2	0.096651984	7.425227949	2.786518924	0.006709122	0.039310077	-3.329812987
RCN2	0.091720849	6.026079771	2.785735957	0.006723831	0.039380945	-3.33498049
MTHFD2	0.156294898	5.931491427	2.787294347	0.006694583	0.03925543	-3.336130383
EDC3	-0.110048574	4.797389111	-2.758730267	0.007249626	0.041508586	-3.33752039
FAM111A	-0.166184937	4.714747735	-2.766280655	0.007098955	0.04090997	-3.339289596
EIF2B1	0.130679721	5.445983626	2.77783649	0.006873897	0.039995515	-3.340333446
GIGYF2	-0.260782249	6.325434053	-2.783170177	0.006772241	0.039541486	-3.344325064
RSBN1	0.268871455	3.909299609	2.716125227	0.00815603	0.045673769	-3.344459886
RALBP1	-0.077219347	6.238666056	-2.783515104	0.006765714	0.039518697	-3.346249284
C9orf40	-0.133647926	3.43682433	-2.704789995	0.008414014	0.046805285	-3.346335616
TLN1	-0.113709273	9.865351579	-2.728085126	0.007891672	0.044457797	-3.348744521
ATF4	-0.176214467	7.770699085	-2.76936756	0.007038184	0.040668665	-3.349127757
DENND6A	-0.170282933	3.014455511	-2.675162168	0.00912385	0.049582134	-3.349489874
RNF14	-0.116666067	5.051177022	-2.761731069	0.007189397	0.041273277	-3.351352483
EID2	0.145549979	3.818092351	2.715347283	0.008173502	0.045737608	-3.353300798
THUMPD3	-0.101022261	4.838756984	-2.7566595	0.007291458	0.041652832	-3.354090646
PAIP2	0.077099145	4.596712723	2.745878772	0.007512827	0.042610399	-3.355274763
NEDD9	-0.354342358	2.620600824	-2.730542954	0.007838324	0.044206856	-3.35537664
AP3B1	0.075298258	6.252266956	2.779391273	0.006844121	0.039846268	-3.355662999
DCAF12	-0.137641776	4.67787757	-2.742333329	0.00758696	0.04298231	-3.357102408
CHCHD7	0.13705702	3.499273544	2.700565596	0.008512048	0.047186729	-3.358748789
FOXP4	-0.157623179	5.298893118	-2.764774666	0.007128777	0.041019059	-3.359836862
GOT1	0.158959358	5.087643078	2.762313475	0.007177761	0.041237906	-3.360806993
C1orf52	0.136714799	3.976204896	2.729887886	0.00785251	0.044270289	-3.361724676
EXT1	0.110367729	8.076924861	2.765372644	0.007116922	0.040997826	-3.363968689
C7orf49	-0.127233731	3.44350715	-2.687012572	0.008833635	0.048530227	-3.366033828
S1PR3	0.258466447	5.272747546	2.751362313	0.007399472	0.042136484	-3.369475564
C1QBP	0.15872721	6.028495869	2.770244085	0.007021016	0.040616198	-3.374000672
MAP3K13	0.108021664	4.276704187	2.725704078	0.00794367	0.044700577	-3.375602757
PLEKHA1	-0.167300588	4.146543413	-2.718159622	0.0081105	0.045435688	-3.378433986
SARAF	0.083311225	7.281825937	2.767692014	0.007071111	0.040827602	-3.37878428
NSDHL	0.149418076	5.653501009	2.759033162	0.007243526	0.041508586	-3.378998189
DBR1	-0.114653977	3.651182318	-2.696366238	0.008610531	0.04759957	-3.383447504
SLC35D2	-0.121257995	3.461716936	-2.693167533	0.008686242	0.047895151	-3.385567109
CDK12	0.294626051	5.773545984	2.764065758	0.007142855	0.041053019	-3.385886169
ZPR1	0.116533882	5.169560429	2.754478687	0.007335751	0.041842388	-3.38715751
SRSF4	-0.103717623	6.023767113	-2.764449835	0.007135224	0.041024817	-3.38724052
VDR	0.200415474	5.957651041	2.767097301	0.007082832	0.040863951	-3.388158111
SNRPD3	0.099131786	5.495004741	2.757874955	0.007266878	0.041543927	-3.388529285
MEN1	-0.148575097	5.350119782	-2.754662763	0.007332003	0.041836851	-3.391846585
BAG3	0.1119846	6.595277552	2.764632944	0.007131589	0.041019573	-3.395393355
ANXA7	0.092859839	7.115400192	2.761792587	0.007188167	0.041273277	-3.396392881
ETFRF1	0.112561574	3.463467338	2.686291542	0.00885105	0.048590459	-3.39748052
TMEM129	0.092808991	5.461834079	2.755073926	0.007323637	0.041814987	-3.399339262
ARAP1	-0.079484799	6.960768604	-2.756899313	0.007286602	0.041640885	-3.408660017
PES1	0.103810269	6.676033612	2.75880042	0.007248213	0.041508586	-3.409991502
MIER1	0.099323231	5.456200614	2.749930762	0.007428913	0.042229867	-3.413242531
DTD2	0.14053312	3.513517754	2.679789527	0.009009509	0.04917364	-3.414275467
TP53RK	0.09550906	5.001756232	2.738866171	0.007660102	0.043347775	-3.417320068
GLO1	0.06913193	7.00223483	2.753793175	0.007349724	0.04189037	-3.419078473
FAM8A1	0.105776155	5.909476391	2.75058617	0.007415421	0.042185008	-3.423175181
LTBP3	-0.155855276	8.657054419	-2.725939801	0.007938508	0.044688224	-3.423931029
DOCK6	-0.145119681	4.03369497	-2.699111281	0.008546037	0.047347373	-3.42653633
LURAP1L	-0.168294523	5.060392173	-2.731634311	0.007814741	0.04410688	-3.428143238
VMP1	0.087985375	6.495552964	2.751125276	0.00740434	0.042137882	-3.429120755
APC	-0.29051296	5.028465418	-2.723353763	0.007995303	0.044950586	-3.436092292
BCR	-0.131673118	5.941560264	-2.743020303	0.007572543	0.04293293	-3.440769655
RAB2A	0.079253059	7.392964502	2.742531987	0.007582788	0.042974839	-3.440892085
WDR61	0.099710118	4.359525443	2.702013097	0.00847834	0.047076191	-3.441100672
ZNF512B	-0.112057481	4.961439968	-2.72327446	0.007997051	0.044950586	-3.44395442
PDCD6IP	0.083578481	7.247319922	2.739426684	0.007648234	0.04329688	-3.451163762
KIAA2026	-0.201478386	4.358483464	-2.694066559	0.008664902	0.047823047	-3.45578088

Gene	Log fold change	Average Expression	t	P-value	Adjusted P-value	B
PMP22	0.081938582	8.017996358	2.721664875	0.008032595	0.045116706	-3.463654189
RAVER1	-0.130047997	4.732799426	-2.704887663	0.00841176	0.046805285	-3.467129161
CALCOCO1	0.090815202	6.697765744	2.734414642	0.007754955	0.043802266	-3.469999284
C1GALT1C1	0.092675888	4.673506536	2.702380201	0.00846981	0.047059744	-3.472806817
MIER3	0.238529134	4.136575363	2.67818774	0.00904894	0.04931738	-3.475519908
CFAP20	0.12286183	3.981704401	2.678398005	0.009043755	0.04930696	-3.476328659
POLDIP3	-0.172776313	6.328643382	-2.731031925	0.00782775	0.044163755	-3.477553629
SNX4	0.13547832	4.15356101	2.682196779	0.008950544	0.048922712	-3.47766966
ZBTB40	0.233233785	4.336647058	2.677578275	0.009063985	0.049345814	-3.481073309
MPC1	0.147454878	4.310153314	2.686024237	0.008857514	0.04859053	-3.486228921
ZNF436	0.303064238	5.941534948	2.722312185	0.008018283	0.04505312	-3.492610537
FRYL	-0.313315993	4.310723798	-2.67302382	0.009177133	0.049831387	-3.494066363
MYL12A	0.10837096	7.295952438	2.721380298	0.008038894	0.045135258	-3.495227052
ICE1	0.227594261	5.573652395	2.714327674	0.008196454	0.045814984	-3.496147211
MLXIP	-0.192842014	5.993028657	-2.718653566	0.00809948	0.045390836	-3.50070596
SRSF5	0.15363544	6.58867383	2.719562245	0.008079244	0.045328017	-3.504078206
AARS	-0.088029588	8.052486475	-2.700556935	0.00851225	0.047186729	-3.509181932
UBA5	0.106476066	4.62835296	2.685600719	0.008867765	0.048621428	-3.51022016
DAB2	0.139518484	9.017649532	2.67778148	0.009058966	0.049345814	-3.513079757
ZBED1	-0.093069943	5.862592463	-2.714419328	0.008194388	0.045814984	-3.515058776
CCDC115	0.111032349	4.772941957	2.689688961	0.008769266	0.048246977	-3.518587012
TTC7A	0.11531821	5.419718498	2.697058394	0.008594227	0.047544414	-3.522236989
PPP1R3C	-0.25479429	5.524912932	-2.689927504	0.00876355	0.048233143	-3.528161031
CRBN	-0.080793238	4.561959212	-2.680543176	0.008991011	0.049108262	-3.528832382
ALPK2	0.235233269	5.400052428	2.6987394	0.008554749	0.047362301	-3.533390066
MAX	-0.062097812	5.949285316	-2.705526751	0.008397023	0.046745288	-3.535207686
RIC1	0.320066408	4.883636568	2.684649948	0.008890816	0.048712525	-3.535260902
RPS27L	0.17835269	7.374167856	2.702599891	0.00846471	0.047052541	-3.535472883
SAMD4B	-0.156512675	7.688249713	-2.694541557	0.008653646	0.047785382	-3.539534063
TMEM237	0.090662043	4.842308451	2.68019993	0.008999432	0.049136439	-3.540025122
GATD1	-0.144789506	5.222908309	-2.68910304	0.008783321	0.048306664	-3.541794814
ATP6AP2	0.078015938	8.003759151	2.690854309	0.008741372	0.048128666	-3.542422139
BICD2	-0.153643856	5.770441333	-2.69683892	0.008599394	0.047555532	-3.548541798
EI24	0.073962118	7.140784474	2.700485806	0.008513909	0.047186729	-3.550755333
GYS1	-0.077390465	5.504048977	-2.69195938	0.008714996	0.048018544	-3.551148797
TWF1	0.097175292	6.043081477	2.698727818	0.00855502	0.047362301	-3.553713615
DAXX	-0.117738162	5.834228116	-2.69594859	0.008620382	0.047619154	-3.556947281
CCSER2	-0.133650631	5.722479411	-2.691155868	0.008734167	0.04810658	-3.566004431
EIF2S3	0.09581855	7.143188284	2.693487778	0.008678635	0.047870728	-3.566013817
FAM120B	-0.104147277	5.333834922	-2.682640067	0.008939724	0.048899057	-3.572066109
MAEA	-0.068929701	5.621563409	-2.686089899	0.008855926	0.04859053	-3.574955923
PANK3	0.206472413	5.811925513	2.684572843	0.008892687	0.048712525	-3.584048032
TMEM98	0.080622309	7.082873298	2.683805027	0.008911346	0.048782711	-3.591614995
ADH5	0.064864871	7.726814883	2.672918193	0.009179772	0.049831387	-3.599335623
C9orf78	0.08221029	5.714113873	2.676784019	0.009083625	0.049434872	-3.599752179
VAMP3	0.064602998	7.445697395	2.674242919	0.009146721	0.049688497	-3.606534351
PLXNA3	-0.115687029	5.524921183	-2.672791119	0.009182948	0.049831387	-3.610952096
PRDX1	0.119951663	7.345007249	2.672119332	0.009199756	0.049888567	-3.614021037
TRIM22	0.085371159	6.847950027	2.675334942	0.009119557	0.04957669	-3.616959306
