# Supplementary material for: Comparison of time and dose dependent gene expression and affected pathways in primary human fibroblasts after exposure to ionizing radiation
Source: Mol Med. 2020 Sep 9;26:85. doi: 10.1186/s10020-020-00203-0 (PMC7488023; doi:10.1186/s10020-020-00203-0)
Supplement: Supplementary file 11 — Additional file 11: Web Table 1D. Differentially expressed genes 4 h after exposure to high dose ionizing radiation (2 Gray). [file 10020_2020_203_MOESM11_ESM.pdf]

Web Table 1D: Differentially expressed genes 4 hours after exposure to high dose ionizing radiation (2 Gray).

Gene	Log fold change	Average Expression	t	P-value	Adjusted P-value	B
CDKN1A	1.953793501	9.578941307	36.51032106	2.71E-50	4.09E-46	102.4322623
PPM1D	1.427962733	4.428273031	30.95634703	3.90E-45	2.94E-41	91.68402178
SESN1	1.445791861	4.358072382	28.57029552	1.14E-42	5.71E-39	86.25366503
PCNA	1.150876044	5.947349807	25.21271253	6.74E-39	2.54E-35	78.01418735
MDM2	1.82344373	6.788792003	24.6550448	3.12E-38	9.40E-35	76.50257123
BLOC1S2	0.889099096	5.890622378	24.1232989	1.38E-37	3.46E-34	75.07085155
PHLDB3	1.737078236	3.007928833	22.79721317	6.27E-36	1.35E-32	70.72137511
FBXO22	0.959362261	4.733788456	22.66734672	9.19E-36	1.73E-32	70.90071167
FAS	0.845983693	5.41049368	22.3688394	2.23E-35	3.73E-32	70.0709961
TIGAR	1.244625133	4.312602112	21.31391687	5.48E-34	8.26E-31	66.84689752
TRIAP1	0.882657687	4.994139952	21.00025981	1.45E-33	1.99E-30	65.95706066
BBC3	1.558497276	4.023738717	20.72275287	3.47E-33	4.35E-30	64.99089778
RAP2B	0.846070993	6.391043181	20.24829163	1.57E-32	1.81E-29	63.65593793
MRPL49	0.611788125	5.15600004	20.19227886	1.87E-32	2.02E-29	63.4618788
HSPA4L	1.441938638	3.94770276	18.8704937	1.43E-30	1.44E-27	59.11577582
MVB12B	0.848324525	3.617885268	18.58651553	3.74E-30	3.52E-27	58.13541085
DCP1B	0.757512371	4.061718856	18.47125955	5.53E-30	4.90E-27	57.82354496
WDR63	1.488912077	1.76538814	16.82307189	1.78E-27	1.49E-24	51.4609755
IER5	1.106680548	6.031800192	16.55304248	4.73E-27	3.75E-24	51.18503266
FDXR	0.932735569	5.055756172	16.48694938	6.02E-27	4.53E-24	50.94173935
PHLDA3	0.817741993	7.237706873	16.45768356	6.70E-27	4.80E-24	50.84025071
BTG2	1.883364918	4.197394101	16.4417977	7.10E-27	4.86E-24	50.76137392
GDF5	1.55484675	3.56404684	16.06066091	2.88E-26	1.89E-23	49.34728675
TRAF4	0.930627714	4.04860766	15.87738543	5.68E-26	3.57E-23	48.68831635
TBC1D22B	0.654579926	3.409827416	15.38907407	3.55E-25	2.14E-22	46.86127286
RRAD	2.680726088	-0.756764208	15.23611407	6.35E-25	3.68E-22	43.5066225
KLLN	1.477525918	0.394399179	15.05686391	1.26E-24	7.02E-22	44.70491734
TAP1	0.644425774	5.266718745	14.94385976	1.94E-24	1.04E-21	45.22116399
ARHGAP22	0.99644821	5.005111097	14.89507633	2.34E-24	1.22E-21	45.03570136
ZFPM1	1.180873098	2.984503149	14.84733294	2.81E-24	1.41E-21	44.77736289
RNF19B	0.697811177	3.164707185	14.20797991	3.39E-23	1.65E-20	42.37050257
ARID3A	0.917408872	3.536743634	14.19075157	3.63E-23	1.71E-20	42.31282752
MAST4	1.587837998	3.351853681	14.10045488	5.18E-23	2.37E-20	41.95146115
CCDC51	0.830499921	3.648749669	13.48536023	6.02E-22	2.67E-19	39.53942523
CES2	0.426925167	5.81792216	13.18562218	2.02E-21	8.71E-19	38.31592436
SLC52A1	2.970636303	-1.805607518	13.1710056	2.15E-21	8.99E-19	34.70561704
NYNRIN	1.2099976	6.251609185	13.13507642	2.49E-21	1.01E-18	38.11218618
ATG4A	0.644031487	3.19141132	12.97452059	4.78E-21	1.90E-18	37.48266752
FBXW7	0.875113803	3.679572079	12.84611106	8.10E-21	3.13E-18	36.96897091
PARD6G	0.750369828	2.515779141	12.81539428	9.19E-21	3.46E-18	36.82229641
POU2F2	1.21619882	3.80042286	12.77200412	1.10E-20	4.03E-18	36.66265236
FHL2	0.602049827	7.000128448	12.37444436	5.69E-20	2.04E-17	34.99918802
BCL2L1	0.60244047	6.143834118	12.36574649	5.90E-20	2.07E-17	34.96390191
CABYR	1.047144501	1.147853121	12.23034408	1.04E-19	3.55E-17	34.23868843
TAF3	0.529605445	4.130986198	12.1904626	1.22E-19	4.10E-17	34.27196245
SCRIB	0.412910907	5.840180126	12.08190798	1.93E-19	6.32E-17	33.78588763
ZNF79	0.806088342	2.889914548	12.01685793	2.53E-19	8.12E-17	33.57003996
E2F7	1.263110127	1.951261267	11.82305072	5.73E-19	1.80E-16	32.74320927
PLK3	0.892963157	4.367813593	11.79123037	6.56E-19	2.02E-16	32.61518596
DDB2	0.527978005	5.250345323	11.70506084	9.44E-19	2.84E-16	32.21606484
PGF	1.681959225	2.786905909	11.68070928	1.05E-18	3.09E-16	32.15296731
PACSIN3	0.997062118	4.026428573	11.60517407	1.44E-18	4.15E-16	31.83438054
GDF15	1.756528508	5.277656728	11.60233437	1.46E-18	4.15E-16	31.80882123
ETV7	1.147064956	0.544258867	11.53820934	1.92E-18	5.35E-16	31.25222129
COQ8A	0.657590576	4.316638727	11.43693544	2.95E-18	8.08E-16	31.11523559
IKBIP	0.280096431	6.354412931	11.28068826	5.75E-18	1.55E-15	30.40658962
VAV2	0.506731634	4.729795476	11.15220101	9.97E-18	2.63E-15	29.88408825
DAB2	-0.575426635	9.017649532	-11.10970298	1.20E-17	3.11E-15	29.72125667
SESN2	1.257153916	5.719769829	11.04998318	1.55E-17	3.95E-15	29.43244025
SAC3D1	0.712042487	3.502055546	10.9750202	2.14E-17	5.36E-15	29.17694427
TLCD1	0.594589477	2.17816021	10.94271678	2.45E-17	5.96E-15	29.0648328
TNFRSF10B	0.608963965	6.324581296	10.94350748	2.45E-17	5.96E-15	28.96695656
TNFRSF10D	0.932427545	4.215595526	10.75774788	5.46E-17	1.30E-14	28.2308617
TOB1	0.577439563	5.784883154	10.68996558	7.32E-17	1.72E-14	27.88173793
SAMD4A	-0.833698966	5.653654879	-10.67694393	7.74E-17	1.79E-14	27.82964609
POLH	1.009119625	4.858459809	10.64938667	8.73E-17	1.99E-14	27.7305078
TLE1	0.630412793	4.338020448	10.63128125	9.44E-17	2.12E-14	27.66828833
MAP2K3	-0.839298959	6.536569544	-10.53638128	1.43E-16	3.16E-14	27.21555797
THSD1	1.051558472	3.121218096	10.49044208	1.74E-16	3.80E-14	27.12686054

Gene	Log fold change	Average Expression	t	P-value	Adjusted P-value	B
TMEM68	0.576149734	2.976632077	10.47799798	1.84E-16	3.95E-14	27.06959701
AARS2	0.582335838	3.605816219	10.4488398	2.09E-16	4.43E-14	26.91894147
SRSF6	-0.534530158	6.611087388	-10.39909694	2.59E-16	5.42E-14	26.62442584
MARCKS	-0.306781435	8.796832226	-10.32457615	3.58E-16	7.40E-14	26.34142384
DNAJB2	0.391640175	5.873914115	10.29513457	4.08E-16	8.30E-14	26.17140556
SDHA	0.290623544	6.127631499	10.27558101	4.44E-16	8.92E-14	26.08440247
KLF4	0.464837127	6.567953487	10.24125217	5.16E-16	1.02E-13	25.93656044
ZNF367	-1.347765314	2.277310962	-10.08654197	1.01E-15	1.99E-13	25.39982808
FJX1	-0.653860686	3.784608156	-10.04786978	1.20E-15	2.32E-13	25.19292136
TP53INP1	0.852947577	5.508580613	10.01428838	1.39E-15	2.66E-13	24.95294966
AEN	0.810275464	4.984272254	10.00972957	1.42E-15	2.68E-13	24.96817316
ALPK2	-0.875185015	5.400052428	-10.0004596	1.48E-15	2.75E-13	24.90544679
PIDD1	0.748656132	3.527555897	9.888252466	2.42E-15	4.45E-13	24.50614748
ANKZF1	0.59721447	3.044247036	9.822714126	3.23E-15	5.79E-13	24.22507997
EHBP1	-0.407379038	6.601495233	-9.824409688	3.21E-15	5.79E-13	24.11855682
GPC1	0.377965438	7.649240959	9.792859249	3.68E-15	6.53E-13	24.00162722
MRPL39	0.553853321	3.685957242	9.723802077	4.99E-15	8.74E-13	23.77547528
PTPN12	-0.367377782	5.891333803	-9.677654624	6.12E-15	1.06E-12	23.48527614
THEM6	0.500484718	3.370717777	9.670101905	6.32E-15	1.08E-12	23.56048291
TANK	-0.357563983	4.913049683	-9.572881249	9.71E-15	1.64E-12	23.06105492
GADD45A	1.539106052	4.930651997	9.497940419	1.35E-14	2.26E-12	22.75426937
VWCE	0.820061048	4.044055864	9.418708572	1.92E-14	3.17E-12	22.41363932
PLXNB2	0.348463572	8.671728735	9.413487852	1.96E-14	3.18E-12	22.37407907
RHOBTB3	-0.48982568	6.8082914	-9.414398454	1.95E-14	3.18E-12	22.32508008
LRRC42	-0.310628985	5.109243013	-9.396627143	2.11E-14	3.39E-12	22.26596433
CRIPT	0.371039528	4.184897106	9.361942097	2.46E-14	3.91E-12	22.15899948
PRR16	-0.375915421	4.47269225	-9.321794627	2.94E-14	4.62E-12	21.9712431
ARHGAP18	-0.605609406	4.711536226	-9.316802407	3.01E-14	4.67E-12	21.94645195
WHRN	0.923805161	2.534640824	9.269244399	3.71E-14	5.71E-12	21.87562873
CAVIN2	1.253276035	1.192694398	9.25320266	3.99E-14	6.06E-12	21.78740593
HDAC7	-0.320538112	7.246363863	-9.233499236	4.35E-14	6.55E-12	21.5368213
PTP4A1	0.449316335	7.322408638	9.15485271	6.16E-14	9.19E-12	21.19385325
SLC9A3R1	0.404073182	3.912537081	9.139201101	6.60E-14	9.75E-12	21.19748646
SH2D4A	-0.532204075	5.219020967	-9.107342511	7.60E-14	1.11E-11	21.00658059
PELI3	0.437484888	4.250853172	9.101453798	7.80E-14	1.13E-11	21.00593313
SLC12A4	0.293242376	6.265771077	9.088022986	8.28E-14	1.19E-11	20.88897157
PLK2	0.741965372	4.854332864	9.03915598	1.03E-13	1.46E-11	20.69485829
NUMBL	-0.455882107	6.275816428	-9.015639977	1.14E-13	1.61E-11	20.57072831
MSH6	-0.423200495	5.917154835	-8.971741289	1.39E-13	1.93E-11	20.37950681
FERMT2	-0.568726734	7.023179374	-8.906315885	1.85E-13	2.56E-11	20.09351522
SRGAP3	0.898135145	1.593080831	8.87152537	2.16E-13	2.96E-11	20.1528751
POU3F1	2.979171195	-3.664674794	8.864353418	2.23E-13	3.03E-11	17.72441859
EDA2R	0.479112814	4.690613368	8.784033341	3.19E-13	4.29E-11	19.60020409
CEP164	0.349358451	4.464904601	8.774004087	3.33E-13	4.44E-11	19.56132875
PER2	-0.701767266	3.070457678	-8.771189827	3.37E-13	4.46E-11	19.70168477
TMEM97	0.679803375	5.282706806	8.760539019	3.54E-13	4.63E-11	19.45550387
CPSF4	0.536115973	4.182432959	8.748237493	3.74E-13	4.85E-11	19.46300437
ANKRA2	0.652456389	2.486144544	8.739080885	3.89E-13	4.97E-11	19.57277968
NINJ1	0.58744743	4.814540268	8.74058495	3.86E-13	4.97E-11	19.3984702
PRIMPOL	-0.582394668	2.844398578	-8.736615918	3.93E-13	4.98E-11	19.55340841
MAMLD1	-0.474583879	5.282641386	-8.700128763	4.62E-13	5.80E-11	19.20164062
PLCD1	0.413779193	5.186049927	8.604694913	7.06E-13	8.79E-11	18.78123913
RANBP9	-0.317783438	6.11643876	-8.569250447	8.27E-13	1.02E-10	18.60973633
RHBDF2	0.590384693	2.078017259	8.537637933	9.51E-13	1.16E-10	18.69771146
DCLRE1B	-0.480845159	3.266535632	-8.516028783	1.05E-12	1.27E-10	18.50685889
PPP1R10	0.565924311	5.684207763	8.463638041	1.32E-12	1.59E-10	18.14647629
SLC7A8	0.37298096	6.687456896	8.460003173	1.34E-12	1.60E-10	18.12492651
ST8SIA1	1.208959613	2.383663175	8.44869962	1.41E-12	1.67E-10	18.31207819
TRIM3	0.399557012	3.978655486	8.431136993	1.53E-12	1.80E-10	18.07922017
HIP1R	0.472516275	3.429358216	8.427542189	1.55E-12	1.81E-10	18.11509577
STXBP3	0.34512949	4.643779594	8.396185931	1.78E-12	2.06E-10	17.89478615
NECAB3	0.494858755	3.046172099	8.393015308	1.81E-12	2.08E-10	18.0147175
KCNK2	-0.287077781	8.205197416	-8.304305213	2.68E-12	3.06E-10	17.4727276
GAS2L3	-0.875908925	3.350529159	-8.258855506	3.28E-12	3.71E-10	17.37160231
PIAS3	0.288931927	5.014677333	8.253011486	3.36E-12	3.78E-10	17.23851352
MIR22HG	-0.628997217	4.512902585	-8.237416151	3.60E-12	4.02E-10	17.22990989
PRKCE	0.672761133	4.232242803	8.194490336	4.36E-12	4.83E-10	17.0417336
TCOF1	0.301780613	5.330968238	8.192511908	4.40E-12	4.83E-10	16.96292853
RBM14	0.272460944	4.741305263	8.18359929	4.57E-12	4.99E-10	16.9523561
SLC10A3	0.267700659	5.656145013	8.160928589	5.06E-12	5.48E-10	16.81456042

Gene	Log fold change	Average Expression	t	P-value	Adjusted P-value	B
UHRF1	-1.016905722	1.683118784	-8.153838397	5.22E-12	5.62E-10	17.0587482
SH2B3	-0.578060517	5.366836049	-8.149355186	5.32E-12	5.69E-10	16.77399488
SERPINB8	-0.514102955	4.746140507	-8.140898828	5.53E-12	5.86E-10	16.76336737
TSKU	0.363010871	6.163184052	8.134925129	5.68E-12	5.98E-10	16.69371994
FGF5	-0.769886951	5.634833103	-8.128418233	5.84E-12	6.11E-10	16.69910972
PRDM1	0.633742812	2.905438371	8.120798731	6.04E-12	6.28E-10	16.77566248
GEMIN4	-0.368451364	4.971088831	-8.11669981	6.15E-12	6.35E-10	16.64898882
ABHD17C	-0.542523604	4.770395607	-8.09401204	6.80E-12	6.97E-10	16.57582729
PPRC1	-0.449839884	5.959866947	-8.076210845	7.36E-12	7.49E-10	16.44987885
XPC	0.445663389	5.48816694	8.057685135	7.99E-12	8.08E-10	16.36701276
TMEM217	1.000669273	0.558342338	8.053864958	8.13E-12	8.16E-10	16.60791482
CCDC112	-0.571670082	2.439224781	-8.044960766	8.46E-12	8.44E-10	16.53394401
PPME1	0.28579101	5.100861017	8.011504506	9.81E-12	9.72E-10	16.1733723
ZMAT3	0.415596861	7.3940892	7.982052475	1.12E-11	1.10E-09	16.03985779
SPG20	-0.206070447	7.214586345	-7.978969016	1.13E-11	1.11E-09	16.01558849
CDV3	-0.372220785	7.585817109	-7.967205157	1.19E-11	1.16E-09	15.97368679
RNF24	-0.459528077	6.04313865	-7.965060723	1.20E-11	1.16E-09	15.95009414
NCKIPSD	0.376442478	3.784186763	7.919661955	1.47E-11	1.41E-09	15.86449306
METTL13	0.305118515	5.27074638	7.918549009	1.48E-11	1.41E-09	15.76040682
DUSP3	-0.298300832	6.272464369	-7.916078293	1.50E-11	1.42E-09	15.73368915
RPAP1	0.361803832	4.342937164	7.898396004	1.62E-11	1.52E-09	15.7164818
CGRRF1	0.271533208	3.547801621	7.884750384	1.72E-11	1.61E-09	15.73855968
SLC20A1	-0.976887898	7.015763557	-7.861710485	1.90E-11	1.77E-09	15.50235321
PPP4R3A	0.33034728	5.297316175	7.857405504	1.94E-11	1.79E-09	15.48986057
GBP1	-0.752817136	4.986768263	-7.855835877	1.95E-11	1.79E-09	15.53015141
POLR3H	-0.213458594	5.952206543	-7.848172085	2.02E-11	1.84E-09	15.43729213
TAB1	0.374074259	4.765249553	7.84312554	2.07E-11	1.88E-09	15.4632576
TEF	-0.454648186	4.751686708	-7.832756279	2.16E-11	1.95E-09	15.42834817
HDAC9	-0.64905108	2.93685236	-7.828442338	2.20E-11	1.98E-09	15.53038678
MORC4	0.239827326	5.480581671	7.810963393	2.38E-11	2.12E-09	15.28303148
DNAJA1	0.261107705	7.482722506	7.803730375	2.46E-11	2.18E-09	15.26332749
TMEM92	1.081970778	-0.759268617	7.800734284	2.49E-11	2.20E-09	15.43850246
RAB11FIP5	-0.332750178	6.612689139	-7.755642083	3.04E-11	2.66E-09	15.03133553
PTX3	-0.613193094	8.997051295	-7.748151478	3.14E-11	2.74E-09	15.05554227
STK11	0.312683884	5.810760581	7.745201836	3.19E-11	2.75E-09	14.99121333
MGRN1	0.210381412	6.273007404	7.744222747	3.20E-11	2.75E-09	14.98039463
SPRED1	-0.548927394	6.028243405	-7.742493573	3.22E-11	2.76E-09	14.98116956
OSBPL3	0.601621578	5.779485899	7.726999916	3.45E-11	2.94E-09	14.90975657
RNF138	-0.39693379	3.544573481	-7.725172216	3.48E-11	2.95E-09	15.05851216
SP2	-0.316326993	4.278478648	-7.714431039	3.65E-11	3.07E-09	14.95266943
CASP10	0.609867367	2.302150551	7.696149543	3.96E-11	3.31E-09	15.03817241
EHD4	-0.446753853	5.474408355	-7.693640287	4.00E-11	3.33E-09	14.77757087
PRKCD	0.385229224	3.383479729	7.673417463	4.37E-11	3.62E-09	14.79091843
NEK11	-0.366830918	3.942748173	-7.655818545	4.73E-11	3.89E-09	14.71822944
JOSD1	-0.29967707	6.21433537	-7.639697035	5.08E-11	4.16E-09	14.52607339
REEP4	0.457836507	3.368425212	7.630998666	5.27E-11	4.29E-09	14.61226036
IFIH1	0.504918114	3.826447532	7.593472429	6.22E-11	5.01E-09	14.42968956
DDX5	-0.541876947	8.4589716	-7.593579205	6.22E-11	5.01E-09	14.36507678
IL4R	-0.316395634	5.491598057	-7.581386397	6.56E-11	5.26E-09	14.28366301
VAPA	-0.183884155	6.910609197	-7.578495634	6.65E-11	5.30E-09	14.26012729
GDNF	0.799753729	2.514593446	7.570166464	6.90E-11	5.45E-09	14.49372621
GRB10	0.402328461	5.118165129	7.569803057	6.91E-11	5.45E-09	14.24311027
KIF23	-0.656385587	3.037464213	-7.561971788	7.15E-11	5.59E-09	14.40651318
SLC7A1	-0.699369596	6.361562501	-7.561524745	7.16E-11	5.59E-09	14.18834566
TAOK3	-0.313367271	5.237977076	-7.536531971	8.00E-11	6.21E-09	14.09628691
DNAAF2	-0.343632164	3.127355714	-7.513819525	8.84E-11	6.79E-09	14.1408785
BCL2	-0.611835781	3.944966011	-7.514692394	8.81E-11	6.79E-09	14.11109974
VDR	-0.552329494	5.957651041	-7.511047569	8.95E-11	6.84E-09	13.9749402
CHML	-0.603587101	3.373068988	-7.482534277	1.01E-10	7.72E-09	14.01212849
HIST1H2BG	1.613753925	-1.692329728	7.481370582	1.02E-10	7.72E-09	13.78462932
RIMKLB	-0.477447488	3.552132045	-7.476906147	1.04E-10	7.83E-09	13.95423159
ZBTB24	-0.470319921	3.745653668	-7.471836274	1.06E-10	7.97E-09	13.9139282
DKK1	0.664432364	7.22443257	7.470505435	1.07E-10	7.98E-09	13.79135361
TNFRSF10A	0.992415079	0.916942112	7.467887653	1.08E-10	8.03E-09	14.12664352
C1orf198	-0.472773117	6.987827441	-7.459425977	1.12E-10	8.29E-09	13.74460151
ARHGEF3	0.460068937	5.481086069	7.444107697	1.20E-10	8.83E-09	13.69662492
ORAI3	0.501833956	3.935113285	7.429473979	1.28E-10	9.37E-09	13.70938167
SMAD3	-0.369290749	7.130400699	-7.379067799	1.60E-10	1.16E-08	13.40046683
DCAF4	-0.513489107	4.122673375	-7.340495067	1.89E-10	1.37E-08	13.29390863
NAT6	0.506801158	2.422255849	7.338243262	1.91E-10	1.38E-08	13.45814062

Gene	Log fold change	Average Expression	t	P-value	Adjusted P-value	B
MTA3	0.195810527	4.715769842	7.3160717	2.11E-10	1.51E-08	13.17390718
DEPDC7	0.678406128	1.905784148	7.291883639	2.34E-10	1.67E-08	13.331928
TNFRSF1B	-0.682123025	4.680945544	-7.291360191	2.35E-10	1.67E-08	13.04169394
ARHGAP11A	-0.479047973	3.423041714	-7.272965422	2.55E-10	1.80E-08	13.07788521
H1F0	-0.230086295	6.564068945	-7.254036626	2.77E-10	1.95E-08	12.84639846
UQCC1	0.281997696	4.565094582	7.251837784	2.79E-10	1.96E-08	12.89911374
SUOX	0.30896563	3.940767992	7.238887861	2.96E-10	2.06E-08	12.91126527
SEMA6C	0.457173943	3.207102384	7.236886615	2.98E-10	2.07E-08	12.97035705
AMD1	-0.53545204	5.714381529	-7.212160478	3.32E-10	2.30E-08	12.67220249
PRR7	0.644307361	1.309576208	7.206347327	3.41E-10	2.35E-08	12.99706604
ADA	0.594813666	4.774683029	7.203225929	3.46E-10	2.37E-08	12.67218556
KLHL42	-0.428119001	4.488815774	-7.200163488	3.50E-10	2.39E-08	12.68399649
UBC	0.302483308	9.345611817	7.196173084	3.57E-10	2.41E-08	12.6913372
NEXN	-0.486950738	5.229750969	-7.196923935	3.55E-10	2.41E-08	12.62656373
DTL	-0.838817839	2.637869346	-7.191011932	3.65E-10	2.44E-08	12.85421112
TRAF5	-0.470947264	3.504169606	-7.192024313	3.63E-10	2.44E-08	12.73386204
ATF1	-0.241472151	4.947189102	-7.189098355	3.68E-10	2.45E-08	12.60502775
BCOR	-0.778815319	4.543805943	-7.171643412	3.97E-10	2.63E-08	12.54163678
ACER2	1.655589261	-1.493927051	7.165252181	4.08E-10	2.70E-08	12.68326963
TNFRSF10C	0.641710341	1.422892158	7.163969422	4.10E-10	2.70E-08	12.79463059
EVC2	0.282758315	4.110388829	7.151123711	4.34E-10	2.83E-08	12.48030838
CEP250	0.288406794	5.430074012	7.151799032	4.33E-10	2.83E-08	12.4173889
ING5	-0.418790414	2.91927999	-7.148081282	4.40E-10	2.86E-08	12.60033976
HSPA1B	0.463045877	4.993090733	7.146208394	4.44E-10	2.87E-08	12.40724158
ZBTB14	-0.288777374	3.540121875	-7.141700256	4.52E-10	2.91E-08	12.4910275
NR4A3	-0.827266278	5.204685634	-7.139690835	4.56E-10	2.93E-08	12.44009741
EI24	0.197085016	7.140784474	7.133403795	4.69E-10	2.99E-08	12.33559946
ATP8B1	-0.730460573	5.899632055	-7.122207566	4.93E-10	3.13E-08	12.27826445
KCNJ2	0.90992733	2.402750033	7.12027239	4.97E-10	3.14E-08	12.54095144
LIMS1	-0.370206236	6.51713682	-7.114350627	5.10E-10	3.21E-08	12.24341955
PHLPP2	-0.66886918	3.846106915	-7.111912168	5.15E-10	3.23E-08	12.36867216
TRIM24	-0.394136813	4.159617874	-7.111423209	5.16E-10	3.23E-08	12.31396544
RBMS1	-0.312019423	6.997915219	-7.109826797	5.20E-10	3.24E-08	12.22379367
RNF115	-0.22301324	6.04159249	-7.106629932	5.27E-10	3.27E-08	12.21146828
TCAIM	0.307496102	3.897876906	7.102396673	5.37E-10	3.32E-08	12.31857014
YPEL4	0.81497317	0.818629285	7.09850874	5.46E-10	3.36E-08	12.56187512
TMEM201	-0.324430706	3.877551585	-7.091561678	5.63E-10	3.45E-08	12.26617856
TMEM138	0.41953861	4.645657487	7.089864272	5.67E-10	3.46E-08	12.19663386
TOX	-0.944256568	1.658053922	-7.0859019	5.77E-10	3.51E-08	12.49443804
PIM1	0.862173925	3.858871985	7.080515916	5.91E-10	3.57E-08	12.20165024
SRSF5	-0.398985387	6.58867383	-7.080128954	5.92E-10	3.57E-08	12.0963663
FGF2	0.410270851	5.899448548	7.071570544	6.15E-10	3.69E-08	12.07351124
NAV3	-0.67930984	4.020636292	-7.068063446	6.24E-10	3.73E-08	12.15084419
TUBGCP6	-0.332767595	4.273233729	-7.042822781	6.97E-10	4.15E-08	12.03870256
C1orf216	-0.263085616	5.463367394	-7.029507359	7.38E-10	4.38E-08	11.89051897
BLCAP	0.199542803	5.677898487	7.019412689	7.72E-10	4.56E-08	11.84089144
RBM12	-0.391454053	6.456127925	-7.005865827	8.18E-10	4.82E-08	11.77540355
DBF4	-0.492561533	2.703949132	-6.998681831	8.45E-10	4.95E-08	11.97349478
RALGDS	0.300231273	5.311682924	6.985099465	8.96E-10	5.23E-08	11.70418546
RCC2	0.385062363	5.656850944	6.980162457	9.15E-10	5.32E-08	11.66898709
SRP68	0.167289045	6.383594122	6.971800527	9.49E-10	5.50E-08	11.62851274
CDC25A	-0.755528489	0.880466049	-6.956603725	1.01E-09	5.83E-08	11.96987169
GSE1	-0.352102375	5.83869265	-6.956849211	1.01E-09	5.83E-08	11.56900797
UAP1	-0.452669761	6.85163187	-6.955718763	1.02E-09	5.83E-08	11.5648272
WBP4	-0.274969048	3.816927692	-6.913295423	1.22E-09	6.95E-08	11.52576293
RALGPS2	-0.529471752	5.476268076	-6.912699553	1.23E-09	6.95E-08	11.39146515
ZNF219	0.3005807	6.050325957	6.91347052	1.22E-09	6.95E-08	11.3804329
FAM212B	0.792856286	2.843669715	6.899242611	1.30E-09	7.34E-08	11.51466204
EED	0.282303037	3.678165261	6.896218703	1.32E-09	7.41E-08	11.42369497
LMNA	0.264717757	10.63727751	6.892815712	1.34E-09	7.49E-08	11.45399641
CSPG4	0.612371743	5.96447642	6.891035441	1.35E-09	7.53E-08	11.28445513
NIP7	-0.301224423	5.116719747	-6.880821172	1.41E-09	7.84E-08	11.27649859
TMEM63B	0.341877889	4.487301324	6.869224233	1.48E-09	8.21E-08	11.25841103
FAM43A	0.67024404	4.466314543	6.864604206	1.51E-09	8.35E-08	11.24505453
ZNF37A	0.290674999	4.646957081	6.863754495	1.52E-09	8.35E-08	11.21717071
PIM2	0.582920753	0.23449648	6.85283196	1.59E-09	8.71E-08	11.53834361
BMP2	0.97433606	3.11209234	6.852148444	1.60E-09	8.71E-08	11.29594983
CABLES1	-0.394150981	5.350006316	-6.842921215	1.66E-09	9.04E-08	11.11071939
SPRED2	-0.365777579	5.967506875	-6.83935189	1.69E-09	9.15E-08	11.07118616
LRRTM2	1.095754006	-0.032077673	6.829927748	1.76E-09	9.47E-08	11.44092809

Gene	Log fold change	Average Expression	t	P-value	Adjusted P-value	B
KBTBD11	-0.670497387	3.327482149	-6.829776915	1.76E-09	9.47E-08	11.23311042
DGKI	-0.678312153	4.003025498	-6.82767055	1.78E-09	9.52E-08	11.13287792
NUDT18	0.416203393	3.070249745	6.80500843	1.96E-09	1.04E-07	11.10632568
PNO1	-0.327370836	4.119826183	-6.804693836	1.96E-09	1.04E-07	11.01059329
RUSC1	0.284865286	5.049675164	6.792014166	2.07E-09	1.10E-07	10.891931
PHC2	-0.313819953	6.700155936	-6.790508909	2.09E-09	1.10E-07	10.85191441
ARNT2	-0.577058734	4.590755291	-6.784434476	2.14E-09	1.13E-07	10.89563619
ZNF385A	0.546961424	5.010030805	6.774448618	2.24E-09	1.17E-07	10.81899678
DZIP3	-0.492368906	3.499124157	-6.770094306	2.28E-09	1.19E-07	10.95483106
UBTD2	-0.256294871	6.235896702	-6.755102298	2.43E-09	1.27E-07	10.70033094
ZBTB44	-0.243432834	5.6187454	-6.746826924	2.52E-09	1.31E-07	10.67881575
NADSYN1	0.317760332	5.623903738	6.746214409	2.53E-09	1.31E-07	10.673186
EFCAB7	0.563711534	2.478421091	6.743692337	2.55E-09	1.31E-07	10.95070518
AAED1	-0.337495245	4.805347277	-6.743418398	2.56E-09	1.31E-07	10.71133603
DHX35	-0.291672489	2.581461466	-6.74161708	2.58E-09	1.32E-07	10.90987906
SGPL1	-0.357605693	4.820410557	-6.729664779	2.71E-09	1.39E-07	10.64251111
CMBL	0.292485811	5.822838897	6.725612151	2.76E-09	1.41E-07	10.58374188
EIF4A3	0.255151027	6.169900897	6.720193453	2.83E-09	1.43E-07	10.55180973
DEDD2	0.385970215	4.046853599	6.703806808	3.03E-09	1.53E-07	10.55150466
HIST1H2BD	0.554251899	2.61383852	6.699322346	3.09E-09	1.56E-07	10.73171695
HSPA1L	0.711748299	1.072949412	6.688865785	3.24E-09	1.63E-07	10.83528554
CSTF3	0.39173041	3.755146628	6.668465932	3.53E-09	1.77E-07	10.46100979
LIMD1	-0.510092233	3.674017102	-6.661001994	3.65E-09	1.81E-07	10.40807299
UCK2	-0.440787823	5.05915692	-6.661251202	3.65E-09	1.81E-07	10.33195814
BICC1	-0.437953114	7.71604122	-6.658555714	3.69E-09	1.83E-07	10.31187577
PGBD2	0.445911905	1.739586415	6.657203048	3.71E-09	1.83E-07	10.64159429
TMEM120B	0.474253994	2.675703675	6.635716478	4.07E-09	2.00E-07	10.43055394
IRF2BP2	0.282185886	6.155245929	6.624473765	4.27E-09	2.10E-07	10.14575343
SRGAP1	-0.591858417	6.088752548	-6.613977675	4.47E-09	2.19E-07	10.10221336
TBCK	-0.233066064	5.332314816	-6.610501829	4.54E-09	2.21E-07	10.11113823
DCUN1D3	0.49186622	4.725073927	6.604592252	4.65E-09	2.26E-07	10.15072814
RAF1	-0.210616079	6.259120627	-6.601185166	4.72E-09	2.29E-07	10.04639451
KCTD10	-0.300147237	6.771134448	-6.600112111	4.74E-09	2.29E-07	10.04140571
C4orf46	-0.396209271	3.356308403	-6.59175988	4.92E-09	2.37E-07	10.19569511
GMPPB	0.270948527	4.103838916	6.589754194	4.96E-09	2.38E-07	10.08864397
CXXC5	-0.351073483	6.988256951	-6.572899944	5.33E-09	2.55E-07	9.947885599
SLC7A11	-0.692023748	4.790378446	-6.544389953	6.03E-09	2.87E-07	9.864009075
TYMS	0.433988538	4.179192654	6.516684428	6.79E-09	3.23E-07	9.778162368
ANKLE2	-0.242299659	5.652825807	-6.512211145	6.92E-09	3.28E-07	9.67611225
LIN7A	0.557404879	2.275825574	6.507897635	7.05E-09	3.33E-07	9.970522124
KRCC1	0.363310604	4.695084235	6.503806811	7.17E-09	3.38E-07	9.68350237
HDAC11	0.307485368	3.684941353	6.494083132	7.48E-09	3.51E-07	9.735598802
SLC27A4	0.252042792	5.433798151	6.486123409	7.74E-09	3.62E-07	9.57329015
FAM111B	-0.882115459	1.939021424	-6.482918272	7.85E-09	3.65E-07	9.931354953
LIG1	0.374741149	3.593740631	6.482979267	7.84E-09	3.65E-07	9.683761128
ELFN2	0.703188867	0.946021658	6.482069867	7.87E-09	3.65E-07	9.948531655
HS3ST3B1	-0.368135176	5.373013503	-6.47774773	8.02E-09	3.71E-07	9.554979114
CNOT2	-0.222949614	5.741944057	-6.47134639	8.24E-09	3.80E-07	9.506589527
ZNF423	0.500686597	3.247743785	6.468690419	8.34E-09	3.83E-07	9.681661988
VIPAS39	0.355708254	4.662199395	6.466649548	8.41E-09	3.85E-07	9.528735313
LSM14A	-0.1605731	6.600632423	-6.465302148	8.46E-09	3.86E-07	9.471939226
KCTD1	0.327784658	2.988684013	6.463285028	8.53E-09	3.88E-07	9.661841503
BEND3	-0.59951053	2.673363191	-6.448987435	9.07E-09	4.12E-07	9.631100428
PRKX	0.46319199	2.786967248	6.446192743	9.18E-09	4.14E-07	9.584222139
TBL3	0.276352153	5.362563944	6.445971889	9.19E-09	4.14E-07	9.407399505
RGS16	0.826448184	2.076997515	6.443749557	9.28E-09	4.17E-07	9.773515484
SMURF2	-0.501831985	5.204137854	-6.433712383	9.68E-09	4.34E-07	9.385567056
TNFSF10	0.620755144	2.985993647	6.428631643	9.90E-09	4.42E-07	9.573160156
LMO7	-0.386865973	6.946652049	-6.425971994	1.00E-08	4.46E-07	9.32009968
ABCB9	0.635281331	1.747649787	6.421914899	1.02E-08	4.52E-07	9.624683235
TXNIP	-0.670159722	7.24926452	-6.421780005	1.02E-08	4.52E-07	9.338700112
SYDE1	-0.25750336	6.323239854	-6.414332831	1.05E-08	4.65E-07	9.259251004
SDHAF4	0.485403419	1.40331821	6.410781311	1.07E-08	4.71E-07	9.657634126
PITPNB	-0.288717914	5.925321791	-6.409303104	1.07E-08	4.72E-07	9.240087378
MSL1	-0.290763589	6.004124613	-6.403665057	1.10E-08	4.82E-07	9.213706919
LOC145694	-0.949278469	0.014071486	-6.393553412	1.15E-08	5.02E-07	9.579440595
HR	0.42670798	3.229848901	6.391562828	1.16E-08	5.05E-07	9.352390163
TFEB	-0.446392471	2.25517721	-6.388037741	1.18E-08	5.11E-07	9.408220401
H2AFY2	0.362809296	3.804013247	6.371253393	1.26E-08	5.47E-07	9.223591616
SMOX	0.446569965	3.63812164	6.367810246	1.28E-08	5.54E-07	9.203011123

Gene	Log fold change	Average Expression	t	P-value	Adjusted P-value	B
THRAP3	-0.263608658	6.786861113	-6.365739634	1.29E-08	5.57E-07	9.054053594
ASXL1	-0.396939721	6.03118855	-6.356221564	1.35E-08	5.79E-07	9.018806787
KDM1A	0.159700804	6.147168097	6.353558565	1.36E-08	5.84E-07	9.002574606
MGME1	0.298082319	3.261437173	6.345483674	1.41E-08	5.99E-07	9.150639109
PPP1R37	0.313333118	5.806505945	6.346067317	1.41E-08	5.99E-07	8.976162127
ZHX3	-0.454268615	6.112450758	-6.346634734	1.40E-08	5.99E-07	8.97583803
HIST1H2BJ	0.749330461	-0.651952373	6.342826272	1.43E-08	6.04E-07	9.418798485
RAPGEFL1	0.537544942	0.705363245	6.341298737	1.44E-08	6.06E-07	9.386393679
TBC1D12	-0.466027205	3.43117724	-6.339494948	1.45E-08	6.09E-07	9.122486239
ZNF337	0.725350988	-0.322842371	6.335925838	1.47E-08	6.17E-07	9.413009821
FUT8	-0.254213768	6.102206878	-6.322862642	1.55E-08	6.48E-07	8.877465814
PDE4DIP	-0.230763821	6.560635395	-6.323212193	1.55E-08	6.48E-07	8.875047553
DUSP11	0.200378637	4.508397358	6.317787612	1.59E-08	6.61E-07	8.914074582
UBL3	-0.241818546	6.968783265	-6.317224806	1.59E-08	6.61E-07	8.855934925
FAM83G	-0.518655661	3.792874725	-6.308148483	1.65E-08	6.85E-07	9.001653922
DGKQ	0.297162829	3.948917271	6.306672032	1.66E-08	6.87E-07	8.926627151
ATF7	0.204703336	4.946653077	6.303471078	1.69E-08	6.95E-07	8.833701453
PPP2R5D	0.247830751	5.904852937	6.300025335	1.71E-08	7.03E-07	8.785347469
NDUFV3	0.304485487	4.988188979	6.298452363	1.72E-08	7.06E-07	8.808663735
CALCOCO1	-0.208922207	6.697765744	-6.29368436	1.76E-08	7.18E-07	8.752005147
PTCHD4	0.63159585	1.049987012	6.290480305	1.78E-08	7.26E-07	9.188196448
PRR5	0.470367959	4.006929966	6.289508237	1.79E-08	7.27E-07	8.833382924
RXRB	0.260073756	4.913670393	6.288109508	1.80E-08	7.30E-07	8.768863794
MEX3C	-0.250623642	5.533814869	-6.287406093	1.81E-08	7.30E-07	8.744480786
TNFAIP8L3	-0.347811035	4.544047074	-6.277243459	1.89E-08	7.60E-07	8.741203272
ARHGAP17	-0.225811852	6.305763663	-6.272441469	1.93E-08	7.73E-07	8.663185193
AXIN1	-0.316603854	4.750588649	-6.264259556	1.99E-08	7.99E-07	8.682277957
PIK3CD	-0.421579258	3.171888928	-6.253571632	2.09E-08	8.34E-07	8.762701525
CBL	-0.560760181	4.166663103	-6.250918518	2.11E-08	8.41E-07	8.650414408
FMN1	-0.694349633	2.862040545	-6.247748968	2.14E-08	8.48E-07	8.808142102
ZFC3H1	-0.546136984	4.053920773	-6.24827657	2.13E-08	8.48E-07	8.617596231
BICD1	-0.445536281	4.067378472	-6.246292459	2.15E-08	8.51E-07	8.62170761
KIAA1614	0.295385445	3.608454138	6.241429966	2.20E-08	8.66E-07	8.680470365
LAMC2	0.446165901	1.961865542	6.239699012	2.21E-08	8.70E-07	8.895774397
TTC23	0.29351162	4.056229276	6.236186091	2.25E-08	8.78E-07	8.607537005
ABL1	-0.269408963	8.120764505	-6.23629097	2.24E-08	8.78E-07	8.552184971
TSHZ3	-0.372267403	5.979418649	-6.23585044	2.25E-08	8.78E-07	8.518469822
USP53	-0.736536622	5.689863853	-6.231297454	2.29E-08	8.92E-07	8.520424919
HINT3	-0.308471707	4.153468796	-6.18320549	2.81E-08	1.09E-06	8.409142789
USP21	0.323302632	3.607283331	6.180850288	2.84E-08	1.10E-06	8.429863536
PPP3R1	-0.272527747	4.977860866	-6.177638275	2.88E-08	1.11E-06	8.305748337
RPS6KA5	0.790943001	0.778951517	6.165032756	3.03E-08	1.17E-06	8.6938582
ZCCHC14	-0.366542979	6.731432286	-6.163469147	3.05E-08	1.17E-06	8.214845763
NIFK	-0.298726054	5.308837163	-6.154322031	3.18E-08	1.22E-06	8.195159099
DISP1	0.450084919	2.757101556	6.150931639	3.22E-08	1.23E-06	8.392855256
DCAF16	-0.335745888	3.749407762	-6.145749192	3.29E-08	1.26E-06	8.254114287
LZTFL1	-0.196113213	4.597979618	-6.137915675	3.40E-08	1.29E-06	8.17220045
LRIF1	0.28865042	3.627153635	6.136029978	3.43E-08	1.30E-06	8.236910777
PPP2R5A	0.265881879	5.426380355	6.132487617	3.48E-08	1.32E-06	8.09365223
MALT1	-0.477754547	4.654921077	-6.119126685	3.68E-08	1.39E-06	8.107873339
PLD1	-0.392027094	3.943127277	-6.115194017	3.75E-08	1.41E-06	8.151475372
UMAD1	-0.276627314	3.73117527	-6.111719002	3.80E-08	1.42E-06	8.142940773
TGFBR1	-0.646277408	6.723783455	-6.111718946	3.80E-08	1.42E-06	7.998506023
ZCCHC17	0.283169483	4.470208016	6.10922417	3.84E-08	1.44E-06	8.06002318
IPO13	0.259969695	5.390031406	6.106467978	3.89E-08	1.45E-06	7.988180918
CFL2	-0.228291071	6.698324837	-6.100219931	3.99E-08	1.48E-06	7.946723724
RBM6	-0.30360181	5.107470628	-6.098105914	4.02E-08	1.49E-06	7.991062287
KIAA0922	-0.536196362	3.931763771	-6.095282332	4.07E-08	1.51E-06	8.029806969
ZNF598	-0.231100357	5.010673749	-6.094874349	4.08E-08	1.51E-06	7.960650704
STEAP1	-0.286162025	5.151362386	-6.084763944	4.26E-08	1.57E-06	7.915159426
TEX10	-0.415258259	5.196076443	-6.077654602	4.39E-08	1.61E-06	7.875193369
NANS	0.291128003	5.030189328	6.074701871	4.44E-08	1.63E-06	7.87015331
CGGBP1	-0.272887196	6.292001153	-6.065760646	4.61E-08	1.69E-06	7.805203944
HECTD3	0.308481784	5.712393707	6.061541282	4.69E-08	1.71E-06	7.7972776
CDR2L	0.195944692	5.560481528	6.056771191	4.79E-08	1.74E-06	7.776041131
TRIB3	-0.770058547	6.146656686	-6.052168316	4.88E-08	1.77E-06	7.770117347
MAP6D1	0.360757076	1.426853227	6.045641797	5.02E-08	1.81E-06	8.105212043
PLEKHA4	0.295130324	6.905160502	6.045388894	5.02E-08	1.81E-06	7.725794445
NOL3	0.463627084	2.4623553	6.042213978	5.09E-08	1.83E-06	7.998643187
MAP2K1	0.189020434	5.113702048	6.04215774	5.09E-08	1.83E-06	7.734573818

Gene	Log fold change	Average Expression	t	P-value	Adjusted P-value	B
TLE3	-0.459439808	5.205073148	-6.037778432	5.19E-08	1.86E-06	7.728899155
ATG2A	0.205582161	5.314992275	6.034993921	5.25E-08	1.88E-06	7.694946519
JAK2	-0.443507573	3.950715641	-6.032317582	5.31E-08	1.89E-06	7.780053119
ADRA2A	-0.573513884	5.407615927	-6.03203745	5.31E-08	1.89E-06	7.705509973
ZCCHC7	0.264811815	3.980274554	6.027496455	5.42E-08	1.92E-06	7.755860234
MBNL1	-0.343031626	7.043340342	-6.027188823	5.42E-08	1.92E-06	7.656232945
BNIP2	-0.295861921	5.977886095	-6.026472705	5.44E-08	1.92E-06	7.646185761
TFAP4	-0.41874004	2.85314038	-6.010081825	5.83E-08	2.06E-06	7.74240396
SEH1L	-0.333993387	5.243595622	-6.003798842	5.98E-08	2.11E-06	7.563973654
RRM2B	0.526878507	5.474807387	5.995662186	6.19E-08	2.17E-06	7.52919619
PLEKHG5	0.549694801	1.89089943	5.994739926	6.21E-08	2.18E-06	7.936054118
ATOH8	-0.524036733	4.498553861	-5.991605329	6.30E-08	2.20E-06	7.59282933
FAM111A	-0.373373569	4.714747735	-5.990652038	6.32E-08	2.20E-06	7.571580695
CCNE1	-0.466125486	1.649013882	-5.989208082	6.36E-08	2.21E-06	7.837973539
FST	0.323493063	6.619768423	5.989715481	6.35E-08	2.21E-06	7.491550034
STK40	-0.288498992	4.891972356	-5.988129004	6.39E-08	2.21E-06	7.543352019
RNF2	-0.222290157	4.637927281	-5.980883082	6.58E-08	2.28E-06	7.512691763
SPIN4	-0.595594255	1.98523633	-5.977509005	6.68E-08	2.30E-06	7.807599688
GSK3A	0.232875158	5.255430311	5.976406852	6.71E-08	2.31E-06	7.458290122
CCNE2	-1.231547081	0.026306582	-5.962641226	7.11E-08	2.44E-06	7.915743883
N4BP2L1	0.43635171	2.49685235	5.95169371	7.44E-08	2.55E-06	7.646323518
MGEA5	-0.433297523	6.190428515	-5.949242556	7.52E-08	2.57E-06	7.326099272
MOB2	0.306936973	4.081970548	5.941008711	7.78E-08	2.65E-06	7.392607889
POLA2	0.321644303	3.397014172	5.938871726	7.85E-08	2.67E-06	7.460145392
CDCA4	-0.269064131	3.00130011	-5.936882718	7.91E-08	2.69E-06	7.49954244
RAB35	-0.201130479	6.022381973	-5.93528228	7.97E-08	2.70E-06	7.271690712
HES2	1.771322761	-2.587944919	5.932685498	8.05E-08	2.71E-06	7.57773301
AEBP2	0.287917119	4.326962876	5.933120356	8.04E-08	2.71E-06	7.353454682
TSPYL5	-0.295304546	4.215347758	-5.923449504	8.37E-08	2.81E-06	7.333505169
MCM10	-0.855769323	0.924570408	-5.916333038	8.62E-08	2.89E-06	7.693111596
NXT1	-0.384842762	3.317768375	-5.915185798	8.67E-08	2.89E-06	7.386821805
RAD1	-0.282165191	4.710254585	-5.915414331	8.66E-08	2.89E-06	7.257139449
LINC01679	0.828357414	0.197908324	5.912908227	8.75E-08	2.92E-06	7.705809917
ZNF563	0.46644809	0.77161294	5.910199576	8.85E-08	2.94E-06	7.597390357
TARS2	0.393737414	3.502339906	5.908400276	8.91E-08	2.95E-06	7.347509269
GJC1	-0.751810485	4.233130727	-5.908778998	8.90E-08	2.95E-06	7.258531773
U2SURP	-0.266921202	5.642251186	-5.906717882	8.98E-08	2.97E-06	7.166720089
TWNK	-0.422913217	3.423486625	-5.903544948	9.10E-08	2.99E-06	7.329496467
CDIP1	0.297300087	4.836958235	5.903410372	9.10E-08	2.99E-06	7.18558377
PPARGC1B	-1.07006454	-0.028967557	-5.898507054	9.29E-08	3.05E-06	7.582249157
HIST1H2AG	0.658569314	0.36249596	5.893366152	9.49E-08	3.11E-06	7.626127025
PSTPIP2	0.415391304	2.24355641	5.876075939	1.02E-07	3.33E-06	7.340435406
ST3GAL1	-0.52309094	5.92300042	-5.875679129	1.02E-07	3.33E-06	7.03106985
MBNL2	-0.404473934	4.966848271	-5.866436189	1.06E-07	3.45E-06	7.044311528
HSP90AB1	0.168719061	9.402644555	5.863611102	1.07E-07	3.49E-06	7.085322699
STX5	0.246109944	5.806787924	5.861280137	1.08E-07	3.51E-06	6.971188843
PPAT	-0.572798529	2.894480482	-5.857418999	1.10E-07	3.56E-06	7.12589458
KLF16	-0.369174113	4.802922398	-5.857723061	1.10E-07	3.56E-06	7.044861355
DAB2IP	0.318662792	6.453616113	5.856197895	1.11E-07	3.57E-06	6.946462928
VPS37B	0.251744445	5.133458168	5.852944524	1.12E-07	3.61E-06	6.972763295
NR2F2	-0.35197592	6.329748965	-5.851155124	1.13E-07	3.63E-06	6.923803624
KIT	-1.29199472	3.397774175	-5.849939776	1.14E-07	3.64E-06	7.244415908
BCL6	0.524462941	5.938922774	5.825416607	1.26E-07	4.02E-06	6.83926024
MAPRE3	0.318404603	4.147758246	5.823382543	1.27E-07	4.03E-06	6.93222602
HSPB8	0.364358982	4.728214553	5.823879925	1.27E-07	4.03E-06	6.87221706
PHF13	-0.599526942	5.251495747	-5.822606861	1.27E-07	4.04E-06	6.847506033
SRGAP2	0.359761649	5.247200741	5.819176312	1.29E-07	4.09E-06	6.81894087
PLEKHG3	0.329087831	3.228756138	5.817844962	1.30E-07	4.10E-06	6.946771991
CEP170B	0.216531277	5.366518976	5.815478557	1.31E-07	4.13E-06	6.794275093
RAI14	-0.388364952	6.442750697	-5.814904682	1.32E-07	4.14E-06	6.77731378
INTS3	-0.307315503	5.008540437	-5.813484618	1.32E-07	4.15E-06	6.829081014
ZBTB1	-0.325350473	5.146071725	-5.809967077	1.34E-07	4.20E-06	6.799126036
ARHGEF17	-0.315542778	5.774788109	-5.808207628	1.35E-07	4.23E-06	6.755132245
REST	-0.482298573	4.514536397	-5.807583231	1.36E-07	4.23E-06	6.816974393
STAT5A	0.217680932	4.999452527	5.798971452	1.40E-07	4.37E-06	6.747103713
ZNF672	0.215017247	4.351264027	5.797903021	1.41E-07	4.38E-06	6.786180847
WIPI2	0.166183283	6.344099156	5.797760635	1.41E-07	4.38E-06	6.706080447
CDC6	-0.502800673	2.79318541	-5.791445821	1.45E-07	4.48E-06	6.938347152
FTO	-0.218825358	6.235342744	-5.788637651	1.47E-07	4.53E-06	6.669603052
ERBIN	-0.377160881	6.036175868	-5.784059661	1.49E-07	4.60E-06	6.656265739

Gene	Log fold change	Average Expression	t	P-value	Adjusted P-value	B
MVD	0.483016508	6.624514458	5.780674908	1.52E-07	4.66E-06	6.643661781
USP16	-0.225755621	5.647831324	-5.776108459	1.54E-07	4.74E-06	6.62852173
WDR91	0.333468074	2.691396339	5.774668223	1.55E-07	4.74E-06	6.881551505
MACF1	-0.524469649	7.50681922	-5.774395564	1.56E-07	4.74E-06	6.6301543
CCDC50	-0.231540699	6.613315298	-5.77465299	1.55E-07	4.74E-06	6.612542296
NSMF	0.185416244	6.079412189	5.771729974	1.57E-07	4.79E-06	6.601280791
ARHGAP29	-0.540580017	6.531040245	-5.770185732	1.58E-07	4.81E-06	6.59704782
DYRK2	-0.279084848	5.512439508	-5.765914122	1.61E-07	4.88E-06	6.592080572
C5orf30	-0.487380742	3.22123045	-5.763387957	1.63E-07	4.91E-06	6.790830772
AFAP1	-0.442715303	7.050306755	-5.763640371	1.63E-07	4.91E-06	6.575009038
RNF114	0.170659534	6.07224011	5.75929732	1.66E-07	4.99E-06	6.552523902
SLC38A1	-0.500935418	6.776361395	-5.75243171	1.70E-07	5.12E-06	6.525463897
SH3D19	-0.202462003	7.199078096	-5.749660549	1.72E-07	5.17E-06	6.516456391
FAM13C	1.129419609	-1.008473751	5.749165745	1.73E-07	5.17E-06	7.057573739
CAMKK1	-0.402573637	3.716931635	-5.742303423	1.78E-07	5.31E-06	6.578332645
SHANK3	0.348232168	2.510931501	5.735482252	1.83E-07	5.45E-06	6.758989413
SMIM15	-0.145226928	5.731025582	-5.734672068	1.83E-07	5.46E-06	6.459782522
MKNK1	-0.27538907	3.842752584	-5.732365184	1.85E-07	5.50E-06	6.603701737
TMEM267	-0.399223463	3.332230027	-5.726382551	1.90E-07	5.62E-06	6.636765176
CTCF	-0.304428273	5.825345183	-5.724154226	1.91E-07	5.67E-06	6.4135523
SLC7A5	-0.562677596	5.52721655	-5.721180693	1.94E-07	5.72E-06	6.453883639
YTHDF2	-0.162053134	5.972099124	-5.718761464	1.96E-07	5.77E-06	6.394211404
COPS8	0.168965869	6.46311088	5.711648862	2.02E-07	5.93E-06	6.35771814
KDM4B	0.267005983	5.396896254	5.710660115	2.02E-07	5.93E-06	6.36661892
GTPBP4	-0.456242278	5.73218001	-5.710547619	2.02E-07	5.93E-06	6.364691228
ZC3H4	0.275974445	4.74804751	5.705745819	2.07E-07	6.04E-06	6.381415853
HIST2H2BE	0.287404984	2.85973671	5.702833816	2.09E-07	6.10E-06	6.550013295
UNC5B-AS1	1.028429204	-1.601274719	5.698178644	2.13E-07	6.21E-06	6.835597521
POLR1B	-0.45705734	3.922830257	-5.69438291	2.16E-07	6.29E-06	6.381197811
STAM2	-0.324693118	4.997047517	-5.694008015	2.17E-07	6.29E-06	6.333352797
WDR70	-0.258916858	4.479631438	-5.691205784	2.19E-07	6.35E-06	6.369849869
FTH1	0.420452211	9.6883212	5.689283047	2.21E-07	6.39E-06	6.392016638
STAU2	-0.281126881	3.94009305	-5.684515111	2.25E-07	6.50E-06	6.393062092
GLUL	0.180345377	7.85090607	5.683707052	2.26E-07	6.50E-06	6.282350526
MFHAS1	-0.406070319	5.147151871	-5.683817384	2.26E-07	6.50E-06	6.267479108
ACSS2	0.353963031	6.101399312	5.682001703	2.28E-07	6.53E-06	6.239204837
ACVR1B	-0.34502967	4.535335083	-5.672681888	2.37E-07	6.78E-06	6.270107586
EEPD1	-0.317089059	4.88099409	-5.668369195	2.41E-07	6.89E-06	6.208600609
FAM49A	0.805798506	0.423640779	5.666287402	2.43E-07	6.93E-06	6.715154341
TRIM68	0.347587667	2.875231273	5.660576301	2.49E-07	7.06E-06	6.346348767
MOSPD2	-0.374301339	4.18114598	-5.660568262	2.49E-07	7.06E-06	6.23233128
TRIP6	0.30694005	6.69965479	5.6603084	2.49E-07	7.06E-06	6.152360993
WDR76	-0.464991417	2.141583849	-5.65770841	2.52E-07	7.13E-06	6.500600435
RTKN	0.296680115	3.428007467	5.655122928	2.54E-07	7.19E-06	6.2649519
CEL	0.684437425	-0.299063735	5.653986305	2.56E-07	7.21E-06	6.70252326
USP12	-0.341722344	4.182484426	-5.652214518	2.57E-07	7.25E-06	6.223133274
PPIF	-0.423064328	5.894195507	-5.651666734	2.58E-07	7.25E-06	6.125631518
YAF2	-0.273203983	3.825695567	-5.64620608	2.64E-07	7.40E-06	6.21919006
SAMM50	0.249625193	5.653486014	5.64386573	2.66E-07	7.46E-06	6.095693349
RFXAP	-0.396963549	2.021121426	-5.643329675	2.67E-07	7.46E-06	6.505923383
VPS37D	0.402328315	1.784647896	5.633202302	2.78E-07	7.76E-06	6.399797266
B3GNT8	0.378679152	1.900385096	5.631678924	2.80E-07	7.80E-06	6.376215674
MTHFD1L	0.20870703	6.662595569	5.630886645	2.81E-07	7.81E-06	6.035653987
KIAA1211L	1.718221415	-2.888964804	5.625443246	2.87E-07	7.97E-06	6.358961755
AQP5	1.582925594	-3.519287679	5.620705629	2.93E-07	8.11E-06	6.285521582
ZFAT	-0.209255556	3.585641502	-5.619662395	2.94E-07	8.13E-06	6.149600375
ANGPTL4	-0.675472862	2.4189572	-5.61482524	3.00E-07	8.28E-06	6.330206225
NADK2	-0.285312481	4.631940222	-5.613305224	3.02E-07	8.32E-06	6.019245278
SF1	-0.198771632	7.754640133	-5.610506124	3.05E-07	8.40E-06	5.976332682
RGS20	0.840346411	-0.610300493	5.60651391	3.10E-07	8.52E-06	6.523638146
APOL3	0.280455816	5.091969825	5.605816099	3.11E-07	8.53E-06	5.967101663
PSMG3-AS1	-0.356842734	3.837224839	-5.605049076	3.12E-07	8.54E-06	6.090195963
VEGFC	-0.199036251	5.406986372	-5.600840646	3.18E-07	8.67E-06	5.937323828
GPSM1	0.234534109	5.830682005	5.599770033	3.19E-07	8.69E-06	5.913419387
BRIX1	-0.306832164	4.265850565	-5.599326667	3.20E-07	8.69E-06	6.043888606
FBXL7	-0.243534978	5.777906104	-5.596884101	3.23E-07	8.77E-06	5.901806246
PRELID1	0.294149776	4.093959139	5.594200663	3.27E-07	8.85E-06	5.989558682
PRKAG2	-0.284631518	3.425174674	-5.593749336	3.27E-07	8.85E-06	6.060611965
XRCC2	-0.717335868	-0.479939049	-5.589838483	3.32E-07	8.96E-06	6.459262119
HTR7P1	-0.317874908	2.700114598	-5.589033999	3.34E-07	8.96E-06	6.169390767

Gene	Log fold change	Average Expression	t	P-value	Adjusted P-value	B
DDIT4	0.621508491	3.769428838	5.590279402	3.32E-07	8.96E-06	6.000207084
LRP3	0.226647432	7.069257127	5.589427654	3.33E-07	8.96E-06	5.873854651
RFTN2	0.383693784	3.17546814	5.584562768	3.40E-07	9.10E-06	6.092043403
TWF2	0.265353927	5.613795479	5.580813498	3.45E-07	9.21E-06	5.844259738
ACAT2	0.400250822	5.335345129	5.580772614	3.45E-07	9.21E-06	5.842555136
TMEM170B	-0.496954789	2.7584743	-5.580096907	3.46E-07	9.22E-06	6.095229751
NUP85	0.231537026	4.06753551	5.575501666	3.52E-07	9.38E-06	5.917235042
ENDOG	0.543089326	1.413662177	5.573886193	3.55E-07	9.43E-06	6.265118335
LMCD1	-0.566497478	6.67097804	-5.570402586	3.60E-07	9.54E-06	5.793143291
ATF3	0.868074128	2.357876858	5.567292873	3.65E-07	9.65E-06	6.244255629
BTBD10	0.253306712	4.128512242	5.565487075	3.67E-07	9.69E-06	5.861469886
ATP5B	0.165809152	8.603648314	5.565454635	3.67E-07	9.69E-06	5.84232796
MEN1	0.298882191	5.350119782	5.557193764	3.80E-07	1.00E-05	5.761332746
SMG9	0.285041321	4.22268709	5.556089088	3.82E-07	1.00E-05	5.817235729
ASB1	-0.262955703	5.014020565	-5.550876507	3.90E-07	1.02E-05	5.764194435
PLCL1	-0.500860174	2.611429262	-5.546832203	3.96E-07	1.04E-05	6.068177544
CASP3	-0.280205061	4.970203619	-5.547084539	3.96E-07	1.04E-05	5.73346538
FNDC3B	-0.475411397	7.321398866	-5.542758181	4.03E-07	1.05E-05	5.698889985
SMTN	0.283201508	6.261889785	5.541787356	4.04E-07	1.05E-05	5.676631817
DTWD2	-0.659520312	1.27694599	-5.535984249	4.14E-07	1.08E-05	6.150022515
CEP120	0.381812725	4.029291108	5.535009769	4.16E-07	1.08E-05	5.764373083
GABPA	-0.247330378	4.605709174	-5.52993819	4.25E-07	1.10E-05	5.694313457
DDX20	-0.265424588	4.389039194	-5.526226383	4.31E-07	1.12E-05	5.707920949
LAP3	0.204802532	5.716237855	5.524632361	4.34E-07	1.12E-05	5.612058016
DNAJB5	-0.282898824	4.03227693	-5.52327125	4.36E-07	1.13E-05	5.710632309
PARP12	0.29538161	3.028443045	5.522266854	4.38E-07	1.13E-05	5.832138176
C19orf68	0.36108328	3.024283823	5.522606209	4.37E-07	1.13E-05	5.816636082
IFT88	-0.288133934	3.299475648	-5.516246756	4.49E-07	1.15E-05	5.787917225
ZNRF3	-0.449577711	2.697124024	-5.514944602	4.51E-07	1.15E-05	5.829078043
PARP2	0.337079868	3.136684638	5.514840937	4.51E-07	1.15E-05	5.78454361
MGC12916	-0.747976399	0.649175589	-5.513131342	4.55E-07	1.16E-05	6.126071429
MIPOL1	-0.365855739	1.996836849	-5.510716902	4.59E-07	1.17E-05	5.961705532
LOXL1-AS1	-0.274364454	2.924088279	-5.509744246	4.61E-07	1.17E-05	5.782325963
PRPSAP2	0.245591706	3.188632442	5.510080997	4.60E-07	1.17E-05	5.760064356
FOXK2	-0.222409326	5.727992708	-5.510039284	4.60E-07	1.17E-05	5.554155225
TNFAIP1	0.21922217	5.729832804	5.50609189	4.68E-07	1.18E-05	5.539703921
SH3BP2	0.229458931	4.365007446	5.503943951	4.72E-07	1.19E-05	5.604813572
EGR1	1.322587043	6.810030351	5.503348427	4.73E-07	1.19E-05	5.532634158
RPTN	2.011870612	-4.033151148	5.500532367	4.78E-07	1.20E-05	5.638987006
FGD1	0.246789267	5.443223203	5.500904713	4.78E-07	1.20E-05	5.529005464
PISD	-0.265147186	4.044412885	-5.496768944	4.86E-07	1.22E-05	5.601761766
PTEN	0.17219601	6.632082941	5.496990987	4.85E-07	1.22E-05	5.498013885
CORO1C	-0.257370993	8.259969258	-5.479311393	5.22E-07	1.31E-05	5.48515459
TRAPPC4	0.230934196	4.37295111	5.473313935	5.34E-07	1.33E-05	5.484561404
ILF2	0.170335892	6.887906996	5.47366912	5.34E-07	1.33E-05	5.413456875
E2F5	-0.428905925	1.062135124	-5.472645055	5.36E-07	1.33E-05	5.902915852
RABGGTA	0.336479329	4.057980442	5.471637407	5.38E-07	1.34E-05	5.534331812
IFT81	-0.240819269	3.62777345	-5.461312472	5.61E-07	1.39E-05	5.514042119
EFNB1	0.2441916	6.236242073	5.461712067	5.60E-07	1.39E-05	5.357633351
TRIQK	-0.242083114	4.673065596	-5.460465122	5.63E-07	1.39E-05	5.428019605
SKIDA1	-0.495812915	0.899748966	-5.459186043	5.66E-07	1.40E-05	5.842532336
PSMD3	0.235294137	6.717412405	5.455768782	5.74E-07	1.41E-05	5.336520177
ZBTB42	-0.373691618	1.949226508	-5.451271273	5.84E-07	1.44E-05	5.691758875
NR1D2	-0.409586216	6.57873226	-5.44446115	6.01E-07	1.48E-05	5.290003589
CHST12	0.176418428	4.701401628	5.436262365	6.21E-07	1.52E-05	5.308343934
NCALD	-0.25454917	4.460875411	-5.434139812	6.26E-07	1.53E-05	5.344058435
KCNN4	0.808164774	0.729205748	5.431165733	6.34E-07	1.55E-05	5.765491066
DPF3	-0.612160225	1.59132432	-5.428667551	6.40E-07	1.56E-05	5.565861917
ZNF395	-0.23125533	5.354431303	-5.427382166	6.44E-07	1.57E-05	5.240444765
AUNIP	-0.632895221	-0.253603411	-5.426010555	6.47E-07	1.57E-05	5.830082455
CNKSR3	-0.344700155	2.766523794	-5.425683145	6.48E-07	1.57E-05	5.475848744
DUSP6	0.573817882	4.819377349	5.425591286	6.48E-07	1.57E-05	5.304335471
PBX1	-0.26987458	7.279924818	-5.42584656	6.48E-07	1.57E-05	5.227594677
USP40	-0.318484668	5.300185567	-5.420770573	6.61E-07	1.60E-05	5.226811926
ATXN1	-0.390134577	5.561416007	-5.417159348	6.71E-07	1.62E-05	5.196355439
SLC2A10	-0.307401022	6.191886874	-5.416704694	6.72E-07	1.62E-05	5.182857352
CLEC16A	0.288921999	4.464108646	5.413541527	6.81E-07	1.64E-05	5.269800052
CCDC28A	0.3852351	2.465225822	5.411823796	6.86E-07	1.65E-05	5.446077053
C20orf24	0.441852496	2.143175913	5.407757016	6.97E-07	1.67E-05	5.496106387
LANCL2	-0.281927716	4.704874196	-5.407765428	6.97E-07	1.67E-05	5.206121748

Gene	Log fold change	Average Expression	t	P-value	Adjusted P-value	B
PTER	-0.33667721	3.711802265	-5.398067955	7.25E-07	1.73E-05	5.291270635
MLF2	0.24375088	7.175422647	5.393132276	7.39E-07	1.76E-05	5.098934599
PHAX	-0.24845177	5.877160807	-5.386594594	7.59E-07	1.81E-05	5.067369121
ASPM	-0.618456933	2.90914576	-5.385609675	7.62E-07	1.81E-05	5.3230616
EIF4H	0.118538677	7.236233068	5.381444325	7.75E-07	1.84E-05	5.055828398
ABHD4	0.253232919	7.141092608	5.381189081	7.76E-07	1.84E-05	5.048600372
MIR3189	1.856262974	-3.122669002	5.378206804	7.85E-07	1.86E-05	5.460163613
FAM122B	-0.234582724	3.634101005	-5.378552524	7.84E-07	1.86E-05	5.192658228
NCOA2	0.715214919	5.315487863	5.377774275	7.87E-07	1.86E-05	5.051449474
KCTD9	-0.230444628	4.508153883	-5.375472792	7.94E-07	1.87E-05	5.085854601
BMP4	0.896534617	1.417703532	5.36988373	8.12E-07	1.91E-05	5.472221037
SRC	0.210098802	6.046066664	5.368866131	8.15E-07	1.92E-05	4.992855923
THOP1	0.225484278	4.967169799	5.367944094	8.18E-07	1.92E-05	5.027904027
CCT6A	0.126838968	7.47940173	5.358306627	8.51E-07	1.99E-05	4.973777621
POLR1E	-0.197142673	5.354264436	-5.357168349	8.55E-07	2.00E-05	4.969855536
TGFB1	0.282819455	7.251656543	5.357409792	8.54E-07	2.00E-05	4.959632767
C1orf174	-0.21370201	3.818757063	-5.352288805	8.72E-07	2.03E-05	5.04814674
MAT2A	-0.547039759	6.516654653	-5.349657873	8.81E-07	2.05E-05	4.917996743
RTN4RL1	0.54389896	2.56083332	5.344836303	8.98E-07	2.09E-05	5.157804791
SERTAD1	0.463028196	5.025227155	5.341973418	9.08E-07	2.10E-05	4.975084154
NAT10	0.180902348	5.644662629	5.34252631	9.06E-07	2.10E-05	4.892378954
AGFG1	-0.309073178	5.999771392	-5.342288288	9.07E-07	2.10E-05	4.887769874
AGO2	-0.395149843	6.18688829	-5.337502108	9.25E-07	2.14E-05	4.869569393
RAD51C	0.323035886	3.025764187	5.33625755	9.30E-07	2.14E-05	5.133032477
INSIG1	-0.528172662	8.714058717	-5.333585352	9.40E-07	2.16E-05	4.951740879
POLRMT	0.23603306	4.041310195	5.331703066	9.47E-07	2.17E-05	4.932782307
KPNA4	-0.227296814	6.625467506	-5.331693577	9.47E-07	2.17E-05	4.846077544
SPIDR	-0.171627218	5.630945731	-5.327865109	9.61E-07	2.20E-05	4.843763527
LOC101930085	-0.23139376	3.392213531	-5.326365955	9.67E-07	2.21E-05	5.029129623
PARN	-0.142402557	5.362290095	-5.324727917	9.74E-07	2.22E-05	4.844794479
PAFAH1B2	-0.237140044	6.19126496	-5.324934409	9.73E-07	2.22E-05	4.819123614
ICE2	-0.238421452	4.492896077	-5.321579461	9.86E-07	2.25E-05	4.896087716
ZFYVE1	0.297271855	5.491491583	5.317931516	1.00E-06	2.28E-05	4.79521414
ZNF596	0.476203666	1.220380087	5.315850805	1.01E-06	2.29E-05	5.251980176
LOC105371267	0.672318239	-0.056561502	5.313629834	1.02E-06	2.31E-05	5.402038608
TFDP2	-0.208219428	5.222223075	-5.313612225	1.02E-06	2.31E-05	4.80598823
LOC100129484	0.540709512	0.594312904	5.313192578	1.02E-06	2.31E-05	5.342113106
NAA25	-0.451640012	3.741589155	-5.303712366	1.06E-06	2.39E-05	4.847921155
ANKRD33B	0.578015504	4.71865765	5.302991151	1.06E-06	2.40E-05	4.776311319
PDE12	-0.31818237	4.810012686	-5.302357159	1.07E-06	2.40E-05	4.783474633
LURAP1L	0.322037321	5.060392173	5.294245002	1.10E-06	2.47E-05	4.734249679
AK1	0.32350684	4.047391097	5.293099399	1.11E-06	2.48E-05	4.80899695
HIF1A	-0.590656967	8.480555201	-5.288703369	1.12E-06	2.52E-05	4.752495974
EPS15L1	0.194094899	5.364930811	5.284790291	1.14E-06	2.56E-05	4.683810066
FAM214B	-0.287547216	4.182907705	-5.283757779	1.15E-06	2.56E-05	4.741045069
TRIM28	0.225936413	8.119257323	5.283637589	1.15E-06	2.56E-05	4.703930005
WASF1	-0.199627132	5.019080802	-5.280682626	1.16E-06	2.59E-05	4.690533936
CDCA8	-0.548326543	1.85808182	-5.279113327	1.17E-06	2.60E-05	5.04606529
GCLM	-0.393225293	5.13316337	-5.279366064	1.17E-06	2.60E-05	4.665260074
MAML2	-0.537100947	5.027235201	-5.274168767	1.19E-06	2.65E-05	4.663350713
PRKAA1	-0.453334887	5.663321824	-5.271928534	1.20E-06	2.67E-05	4.621353764
TNKS1BP1	-0.167881174	8.768560684	-5.269733005	1.21E-06	2.68E-05	4.686811979
SIN3A	-0.389850902	5.491863357	-5.269780272	1.21E-06	2.68E-05	4.612255668
POGK	-0.21415864	5.761933486	-5.267151054	1.23E-06	2.70E-05	4.603673461
CCDC90B	0.222211086	5.688668447	5.265847005	1.23E-06	2.71E-05	4.60261505
CES3	1.542107585	-3.711593821	5.260411555	1.26E-06	2.77E-05	4.95299963
TUBB2A	0.233153753	4.516978986	5.258510881	1.27E-06	2.79E-05	4.611024914
ORC5	0.256972504	3.347553121	5.255784248	1.28E-06	2.81E-05	4.713029104
PMAIP1	0.805652729	1.715422618	5.254550966	1.29E-06	2.82E-05	5.021272866
ELMOD3	0.296166492	2.856801377	5.253766262	1.29E-06	2.83E-05	4.818864624
FAM173B	-0.244303231	3.470259647	-5.248247246	1.32E-06	2.89E-05	4.714963412
ARHGAP35	-0.368301288	7.381156821	-5.246929541	1.33E-06	2.90E-05	4.529051809
INHBB	0.533061116	4.105963332	5.24464316	1.34E-06	2.92E-05	4.593837066
SEC22A	0.196697957	3.528300214	5.244062291	1.34E-06	2.92E-05	4.663656093
SPATS2	0.172713425	4.290155797	5.243633866	1.35E-06	2.92E-05	4.597851991
CNPPD1	0.230456012	5.611593759	5.242251322	1.35E-06	2.94E-05	4.508982017
ZNF354B	-0.409684817	1.277720564	-5.241072542	1.36E-06	2.95E-05	4.959969479
ARNTL	-0.264448563	3.101560028	-5.239646036	1.37E-06	2.96E-05	4.700598416
UVRAG	0.250357576	4.424454725	5.237880278	1.38E-06	2.97E-05	4.576716272
USP1	-0.262234321	5.082302706	-5.237652731	1.38E-06	2.97E-05	4.509895774

Gene	Log fold change	Average Expression	t	P-value	Adjusted P-value	B
PLEKHG2	-0.272184067	4.956632951	-5.235120842	1.39E-06	3.00E-05	4.516719678
BAHD1	-0.409249175	4.528411111	-5.234491589	1.40E-06	3.00E-05	4.521704048
CASP2	-0.241040003	4.346811738	-5.231411293	1.41E-06	3.03E-05	4.521239387
SNRPB	0.309926541	5.61870166	5.231729325	1.41E-06	3.03E-05	4.461699174
LSR	0.677269609	-0.029139993	5.227949817	1.43E-06	3.07E-05	5.055012952
SS18	-0.251312068	5.341592048	-5.223033384	1.46E-06	3.12E-05	4.436047587
SOCS6	-0.299453264	4.845977168	-5.220853089	1.48E-06	3.14E-05	4.46461008
TEAD1	-0.589907848	7.309698512	-5.221200227	1.47E-06	3.14E-05	4.438210392
LARP1	-0.279317282	7.448734189	-5.211060925	1.53E-06	3.26E-05	4.398546775
EIF2B4	0.26369138	4.922494569	5.208878998	1.55E-06	3.28E-05	4.407011836
CSNK1D	-0.186028935	6.373260013	-5.20911981	1.55E-06	3.28E-05	4.366879982
SPTLC3	-0.310834479	2.509336805	-5.203754417	1.58E-06	3.35E-05	4.704697754
MINDY2	-0.453233616	5.539931885	-5.203284241	1.58E-06	3.35E-05	4.362561434
TNFAIP2	-0.376725563	5.180757656	-5.200456454	1.60E-06	3.38E-05	4.37578083
BCDIN3D	0.333262744	1.463905654	5.19853354	1.61E-06	3.40E-05	4.746811676
MID1IP1	-0.209327229	5.248961084	-5.190200241	1.67E-06	3.51E-05	4.32448622
ZNF398	-0.39433372	4.836544897	-5.180921407	1.73E-06	3.64E-05	4.307197299
XRCC6	0.129221377	8.083173543	5.179773247	1.74E-06	3.65E-05	4.301264312
ZC2HC1A	-0.319796315	3.417683388	-5.174795038	1.77E-06	3.72E-05	4.450249406
WASL	-0.281237462	6.017048397	-5.172811069	1.79E-06	3.74E-05	4.229587932
BCAS3	-0.204031355	4.58841566	-5.168542791	1.82E-06	3.80E-05	4.285851002
SETD1B	-0.461409396	4.847184981	-5.16842665	1.82E-06	3.80E-05	4.231350215
CEP68	0.281657583	4.418249454	5.163455827	1.85E-06	3.87E-05	4.248223467
RPGR	-0.352395274	1.789116968	-5.161335873	1.87E-06	3.89E-05	4.636165912
SCAMP3	0.193222714	6.025341279	5.155231753	1.91E-06	3.98E-05	4.161830456
UBTF	0.149175192	7.103248139	5.150745875	1.95E-06	4.05E-05	4.153416872
RNF168	-0.256504866	4.640752936	-5.147671591	1.97E-06	4.09E-05	4.191086478
CYFIP2	0.372050846	3.247507398	5.145922283	1.99E-06	4.11E-05	4.327291537
ZNRF1	0.240932095	4.04494041	5.146239573	1.98E-06	4.11E-05	4.230524488
GFOD1	-0.617840355	0.851941371	-5.144312554	2.00E-06	4.13E-05	4.666104799
ASPHD1	0.376165198	1.865123863	5.143414839	2.01E-06	4.14E-05	4.511518201
AKAP17A	-0.303067056	4.437689016	-5.143192805	2.01E-06	4.14E-05	4.192247868
LPIN2	-0.271130664	5.651252628	-5.137679982	2.05E-06	4.22E-05	4.102922151
KY	0.701058542	0.221966586	5.137265409	2.06E-06	4.22E-05	4.689785031
CDK6	-0.444020713	4.581216609	-5.131222327	2.11E-06	4.32E-05	4.130966321
SPRN	0.604737368	0.49758932	5.129528804	2.12E-06	4.34E-05	4.636376151
PDP2	-0.59071572	3.563233644	-5.1280983	2.13E-06	4.36E-05	4.221826028
SCYL1	0.191023862	6.840004899	5.123004875	2.17E-06	4.45E-05	4.040693777
NCEH1	0.459941168	3.408338628	5.117368904	2.22E-06	4.54E-05	4.227172021
RECQL5	0.276479682	3.005568504	5.114435811	2.25E-06	4.59E-05	4.270659181
ACOT2	0.222398875	3.497236462	5.113122552	2.26E-06	4.60E-05	4.163722553
EIF2B3	0.25761347	4.459012576	5.113205549	2.26E-06	4.60E-05	4.084111636
TMEM160	0.574649703	1.813599221	5.108781645	2.30E-06	4.66E-05	4.423513943
JARID2	-0.382065273	3.250508947	-5.109057609	2.30E-06	4.66E-05	4.180125753
RASA2	-0.496820996	3.925605774	-5.106456532	2.32E-06	4.70E-05	4.104732694
TAF5L	-0.331428031	4.046197342	-5.104550371	2.34E-06	4.73E-05	4.090804603
ANKRD13B	0.295537584	4.322104598	5.102691316	2.36E-06	4.76E-05	4.051820095
SPATA7	0.229325548	3.805807203	5.095147007	2.43E-06	4.90E-05	4.058106554
RHOBTB1	-0.327728325	4.118399134	-5.092476546	2.45E-06	4.94E-05	4.035159226
PLEKHJ1	0.299440181	4.541051096	5.092262857	2.46E-06	4.94E-05	3.986834752
NFKBIE	0.349714418	2.782827442	5.082380039	2.55E-06	5.13E-05	4.119510253
E2F2	-0.910069296	-0.456034884	-5.079213799	2.58E-06	5.18E-05	4.526611128
RTP4	0.41792399	1.140072407	5.076893389	2.61E-06	5.23E-05	4.345735551
MRPL44	0.17450313	4.157932323	5.074630632	2.63E-06	5.27E-05	3.951294492
LINC00909	0.331953932	1.925596144	5.072150196	2.66E-06	5.31E-05	4.262873226
PAG1	0.574628808	3.04878266	5.071366812	2.67E-06	5.32E-05	4.0674421
WDR3	-0.270561056	5.182466021	-5.070536986	2.67E-06	5.33E-05	3.853432467
SLC4A11	0.582899084	1.252786579	5.070031506	2.68E-06	5.33E-05	4.328984707
LOC101927204	-0.404271517	1.173666624	-5.063183606	2.75E-06	5.47E-05	4.336974252
LINC00294	0.464062305	2.899267723	5.061734418	2.77E-06	5.50E-05	4.073054818
LINC00968	-0.650901201	1.055484408	-5.059986154	2.79E-06	5.53E-05	4.278807353
LRRC8A	-0.280624037	6.696913039	-5.054573802	2.85E-06	5.64E-05	3.774026103
LYRM7	-0.222638779	3.78862417	-5.050241864	2.90E-06	5.72E-05	3.916361795
ACTA2	0.232772359	6.35846942	5.050174813	2.90E-06	5.72E-05	3.755515413
SPTSSA	0.175443343	4.848344126	5.049172994	2.91E-06	5.74E-05	3.79169415
SLC35C2	-0.158696829	5.428686615	-5.042834036	2.98E-06	5.87E-05	3.752117792
GIT1	-0.201360644	5.775790984	-5.036315476	3.06E-06	6.02E-05	3.706435196
KIF4A	-0.53810941	2.224821626	-5.035408652	3.07E-06	6.03E-05	4.090196413
SNX30	-0.353146096	5.460957192	-5.034939713	3.08E-06	6.03E-05	3.709984451
ALG6	-0.233420294	3.337235658	-5.032349731	3.11E-06	6.09E-05	3.917072962

Gene	Log fold change	Average Expression	t	P-value	Adjusted P-value	B
NPL	0.488585179	0.461148695	5.031371857	3.12E-06	6.10E-05	4.296108662
MNT	-0.278647334	5.318655716	-5.031278174	3.12E-06	6.10E-05	3.737717184
NBEA	-0.562287196	2.515692331	-5.027813399	3.16E-06	6.17E-05	3.992730921
SELENOO	0.297811801	3.877890603	5.0277567	3.16E-06	6.17E-05	3.793607732
RHOU	0.396777888	2.862563164	5.02632994	3.18E-06	6.18E-05	3.85775556
SMARCAL1	0.183176532	4.950116576	5.0263557	3.18E-06	6.18E-05	3.714901012
PXYLP1	0.331265156	3.890893083	5.024356017	3.21E-06	6.22E-05	3.766395788
TARS	-0.271041391	7.411816593	-5.022637701	3.23E-06	6.26E-05	3.661676473
TRMT2A	0.251900008	3.844730701	5.020556936	3.25E-06	6.30E-05	3.762397698
GAL3ST4	0.226083917	4.543830175	5.018991582	3.27E-06	6.33E-05	3.710787607
ATP6V1B2	-0.372475668	7.147421766	-5.018629001	3.28E-06	6.33E-05	3.647145559
HS6ST1	-0.31158842	4.628138271	-5.016265861	3.31E-06	6.38E-05	3.705229712
PNN	-0.363552018	5.141509249	-5.015642125	3.32E-06	6.39E-05	3.650425427
KIF14	-0.79958661	0.815482183	-5.01341725	3.35E-06	6.44E-05	4.204571225
COA7	-0.246663341	5.227673055	-5.007870949	3.42E-06	6.57E-05	3.624071953
COX5A	0.261870948	5.730418439	4.998504399	3.55E-06	6.81E-05	3.567708331
HAUS3	-0.369683359	2.308327909	-4.997470192	3.56E-06	6.83E-05	3.874944395
MSX2	0.613188804	2.439421403	4.995822663	3.59E-06	6.84E-05	3.875114386
ROCK2	-0.398311351	6.426017177	-4.996217501	3.58E-06	6.84E-05	3.550339254
ANKRD28	-0.304544612	6.333690819	-4.996025118	3.58E-06	6.84E-05	3.549465964
MAP3K10	0.365666815	2.729229699	4.992265269	3.64E-06	6.93E-05	3.830393326
MLLT3	0.386502427	3.68347239	4.989764406	3.67E-06	6.99E-05	3.699053242
SOX12	-0.219839677	5.779609378	-4.986397278	3.72E-06	7.07E-05	3.517759499
NEDD4	-0.368702879	4.385058781	-4.985574561	3.73E-06	7.09E-05	3.605019931
REXO4	0.172315873	4.968767575	4.985303639	3.74E-06	7.09E-05	3.545159048
MB21D2	-0.339302666	2.723524154	-4.978705593	3.83E-06	7.26E-05	3.846136738
BNC2	-0.474058757	5.432235117	-4.976293794	3.87E-06	7.32E-05	3.49330784
INPP1	0.17599952	4.694934988	4.974268289	3.90E-06	7.37E-05	3.521395895
TOR2A	0.330486444	2.166293741	4.973822728	3.91E-06	7.37E-05	3.789179249
PPM1B	-0.210658632	4.549818065	-4.972666372	3.92E-06	7.40E-05	3.525890239
HRH1	-0.390043885	4.530205225	-4.971427428	3.94E-06	7.43E-05	3.520845697
HERC3	-0.400104248	4.699388129	-4.969246809	3.98E-06	7.48E-05	3.509075961
CENPF	-0.397356871	4.782521553	-4.968845319	3.98E-06	7.48E-05	3.516424157
SKI	-0.196782402	7.801894787	-4.96572269	4.03E-06	7.56E-05	3.468772768
GCLC	-0.275624401	3.824491427	-4.964042019	4.06E-06	7.60E-05	3.556650077
C1D	-0.280807426	2.341325739	-4.962954727	4.08E-06	7.63E-05	3.771520261
RGCC	-0.634036393	3.982165762	-4.959025394	4.14E-06	7.72E-05	3.529022959
ANKRD27	-0.299208967	4.439683229	-4.959065728	4.14E-06	7.72E-05	3.465312965
ANTXR2	-0.29918728	7.764460551	-4.959580305	4.13E-06	7.72E-05	3.437231403
SLC39A7	0.11978413	7.498341312	4.958367038	4.15E-06	7.72E-05	3.430073845
PAPOLA	-0.212973144	7.031438212	-4.95811596	4.15E-06	7.72E-05	3.412499052
NEK1	-0.396244754	4.777719625	-4.95286611	4.24E-06	7.85E-05	3.444848452
ADO	-0.164121154	5.077587352	-4.953062754	4.24E-06	7.85E-05	3.42369261
KDELR3	0.188569767	6.931293572	4.953215935	4.23E-06	7.85E-05	3.392671854
WWC2	-0.344357307	4.914778638	-4.952304299	4.25E-06	7.86E-05	3.423958039
SUGP2	-0.202886627	4.7017483	-4.951054643	4.27E-06	7.89E-05	3.442096614
SLC12A7	0.395051953	4.13281493	4.950259259	4.28E-06	7.90E-05	3.479988959
PCMTD2	-0.278922022	4.41777094	-4.950476587	4.28E-06	7.90E-05	3.467627855
TUBA4A	0.428407671	2.672043361	4.949011679	4.30E-06	7.92E-05	3.614705295
BTF3L4	-0.24383287	4.714338382	-4.944705445	4.38E-06	8.05E-05	3.43075556
HAND2-AS1	-0.314699661	2.820986694	-4.943419068	4.40E-06	8.08E-05	3.642725033
FZD4	-0.275054834	4.397280289	-4.941655474	4.43E-06	8.11E-05	3.414843846
IMPDH1	0.192571508	5.744370048	4.94214428	4.42E-06	8.11E-05	3.352111455
PSMC4	0.230038656	5.951134861	4.941638931	4.43E-06	8.11E-05	3.346152013
TBC1D31	0.35110213	1.815630738	4.939628162	4.46E-06	8.16E-05	3.750245039
TBC1D20	-0.144521368	6.723038235	-4.935972428	4.53E-06	8.27E-05	3.322290806
LINC00324	0.752127271	-0.699277596	4.934954783	4.55E-06	8.29E-05	4.017897252
ZNHIT6	-0.213728404	5.00331552	-4.932759284	4.58E-06	8.35E-05	3.351324318
TOMM34	0.199606958	5.009159278	4.931763144	4.60E-06	8.37E-05	3.337358834
RFX1	0.251314752	3.721508768	4.929033941	4.65E-06	8.45E-05	3.41186279
KAT14	0.257017454	3.726469447	4.92795198	4.67E-06	8.48E-05	3.406238029
PTPN14	-0.554077954	6.387033778	-4.927667037	4.68E-06	8.48E-05	3.292158267
TCF25	0.165125798	6.966272345	4.925155086	4.72E-06	8.55E-05	3.286754505
LRRC8C	-0.586208046	2.640381899	-4.922847921	4.76E-06	8.61E-05	3.605888799
FSCN1	0.253044656	8.71078901	4.922152935	4.78E-06	8.61E-05	3.356847887
RAD21	-0.158538599	6.684411681	-4.922643021	4.77E-06	8.61E-05	3.273169614
PIP4K2A	-0.181678534	6.495564363	-4.922431552	4.77E-06	8.61E-05	3.270550918
SRSF2	0.249578285	5.800198373	4.921221034	4.79E-06	8.63E-05	3.267179677
ARHGEF10L	0.186037249	5.046495641	4.920047067	4.82E-06	8.66E-05	3.298758119
SMARCD1	-0.153515371	5.586574899	-4.919302775	4.83E-06	8.67E-05	3.27734774

Gene	Log fold change	Average Expression	t	P-value	Adjusted P-value	B
EPHB4	0.193225763	6.292327357	4.918642517	4.84E-06	8.68E-05	3.255834377
RFXANK	0.31175134	3.880620562	4.917434955	4.87E-06	8.71E-05	3.404854638
CACUL1	-0.248475321	5.114398553	-4.91556752	4.90E-06	8.77E-05	3.268223339
ABTB2	0.341797499	3.118089837	4.91461339	4.92E-06	8.79E-05	3.419367245
TTC14	-0.34250162	3.373045466	-4.910272991	5.00E-06	8.93E-05	3.435015206
ADORA2B	0.296353825	2.509792558	4.907466204	5.06E-06	9.00E-05	3.494903049
IFT140	-0.152336009	5.056182142	-4.907738295	5.05E-06	9.00E-05	3.262120573
ARRDC4	-0.560094813	4.172866516	-4.906799246	5.07E-06	9.02E-05	3.29406535
HDGFRP2	0.295181212	6.318456763	4.904000417	5.13E-06	9.10E-05	3.201132804
RAP2C	-0.192096114	4.570085976	-4.903120755	5.14E-06	9.12E-05	3.264166422
FZR1	-0.15333042	5.336701384	-4.903101787	5.14E-06	9.12E-05	3.217594867
ZXDB	-0.400048915	3.143796067	-4.89959714	5.21E-06	9.23E-05	3.434976505
LDLRAP1	0.207208235	4.813476688	4.899350182	5.22E-06	9.23E-05	3.240369208
MAGED1	0.137481234	8.072603753	4.898792413	5.23E-06	9.24E-05	3.232147827
C1GALT1	-0.317947037	5.544368065	-4.894030748	5.33E-06	9.40E-05	3.187646775
GMEB1	-0.264906794	3.882419068	-4.893379491	5.34E-06	9.41E-05	3.320504529
SYDE2	-0.656486664	0.822354332	-4.892576679	5.36E-06	9.43E-05	3.797209776
NEU1	0.207121104	6.179088923	4.888548132	5.44E-06	9.56E-05	3.144260464
SLC19A3	-0.901081396	-1.412230403	-4.886383219	5.49E-06	9.63E-05	3.843585024
SYNPO2	-0.593476257	4.932095841	-4.886324561	5.49E-06	9.63E-05	3.219927165
OSBPL7	0.4166246	1.54037571	4.882430451	5.57E-06	9.76E-05	3.602653892
CHD7	-0.694648178	0.313095976	-4.877683549	5.68E-06	9.93E-05	3.652255919
FARS2	-0.248797444	3.477805263	-4.876045809	5.71E-06	9.98E-05	3.30852975
CREM	0.228528531	3.593687959	4.875549066	5.72E-06	9.99E-05	3.260764988
PDLIM5	-0.511447921	5.51750303	-4.873348617	5.77E-06	0.000100618	3.091332779
MRPL2	0.273782762	4.176568333	4.872933739	5.78E-06	0.000100663	3.184317058
GJA1	-0.413006171	8.197251695	-4.872443733	5.79E-06	0.000100737	3.131128857
STC1	-0.518036079	3.542155622	-4.868255369	5.89E-06	0.000102224	3.284674297
PPP1R21	-0.175138837	4.134649469	-4.868053942	5.89E-06	0.000102224	3.188389284
DKC1	-0.24429312	5.493462168	-4.865513038	5.95E-06	0.000103114	3.063345124
NACAD	0.307235241	3.537341547	4.8610215	6.05E-06	0.000104797	3.207204375
MYDGF	0.20924796	6.663510475	4.860199357	6.07E-06	0.000105009	3.038323969
USP27X	-0.317570645	2.537054077	-4.85731965	6.14E-06	0.000106061	3.353880713
EHD1	-0.235075666	7.202123632	-4.853757156	6.22E-06	0.000107406	3.02241143
OSGEPL1	0.32856928	1.747334524	4.846399552	6.40E-06	0.000110345	3.454271956
SH3RF3	-0.236821277	4.53157013	-4.846162864	6.41E-06	0.000110345	3.04224646
CARD6	0.266901749	4.86330035	4.842292785	6.51E-06	0.000111765	3.013842196
FKBP4	0.247999311	5.639054938	4.842254136	6.51E-06	0.000111765	2.981378297
VPS33B	0.216373404	3.279246016	4.840186052	6.56E-06	0.000112531	3.15190605
MAP7D3	-0.17628105	5.105172578	-4.839178806	6.58E-06	0.00011284	3.000830794
ANKRD34A	-0.548406133	1.725401346	-4.838348894	6.61E-06	0.000113072	3.347517068
TIPRL	-0.147979202	5.724023636	-4.837755338	6.62E-06	0.000113203	2.965496562
KCNJ8	0.362967133	2.721926012	4.835763726	6.67E-06	0.000113559	3.240482859
CRY1	-0.27832183	3.768772653	-4.836221451	6.66E-06	0.000113559	3.064315341
SLC25A11	0.235746408	5.103066906	4.83633623	6.66E-06	0.000113559	2.989752315
RAB1A	0.130336051	7.590318183	4.835808857	6.67E-06	0.000113559	2.970961357
SUV39H2	-0.330671228	2.765080786	-4.835415458	6.68E-06	0.000113583	3.189491578
USP32	-0.378326476	5.566734917	-4.83268746	6.75E-06	0.000114653	2.949594651
ZNF639	-0.14324431	4.696835495	-4.825651764	6.94E-06	0.000117271	2.979114012
LARP7	-0.175382929	4.916535587	-4.825662077	6.94E-06	0.000117271	2.955625528
WWP2	-0.167318101	5.477560417	-4.82622996	6.92E-06	0.000117271	2.925046042
PA2G4	0.232606207	5.856659021	4.825819233	6.93E-06	0.000117271	2.911910649
PGAM1	0.157529591	4.262173437	4.823479396	6.99E-06	0.000118123	2.989260107
SOCS7	-0.357431871	3.328797056	-4.821933124	7.04E-06	0.000118694	3.066576559
CSNK1G2	0.180149554	6.492699089	4.820437251	7.08E-06	0.000119246	2.887778587
UBIAD1	-0.178795687	3.913843371	-4.817164221	7.17E-06	0.000120621	3.013856603
TFAP2C	0.37957452	2.694240977	4.815549118	7.21E-06	0.000121236	3.189720269
GCFC2	-0.240263891	3.537444657	-4.812853367	7.29E-06	0.000122363	3.04749343
SKIL	-0.330037047	4.434777334	-4.809325766	7.39E-06	0.000123894	2.963060115
HGH1	0.217373732	4.649972896	4.806684196	7.46E-06	0.000125018	2.891958998
PREP	0.163936456	5.196415207	4.805028615	7.51E-06	0.000125676	2.85744941
NECTIN4	1.585761715	-3.371736808	4.802499198	7.58E-06	0.000126761	3.471758279
TCP1	0.141421049	7.349444151	4.801736077	7.60E-06	0.000126992	2.835413649
CEP128	-0.528473988	0.765277155	-4.800844154	7.63E-06	0.000127181	3.426467886
RPAP2	0.248707476	4.091635692	4.800773193	7.63E-06	0.000127181	2.919115482
LAMTOR3	-0.147623381	5.231015097	-4.800100826	7.65E-06	0.000127368	2.839911556
IL12A	0.809631072	-1.299688675	4.797919338	7.72E-06	0.000128297	3.531844619
TBC1D25	-0.176023347	4.002444183	-4.794685317	7.81E-06	0.00012947	2.917183088
KLHL12	0.161118467	4.599746151	4.794767236	7.81E-06	0.00012947	2.864857212
CRK	-0.224773121	6.680729383	-4.795089028	7.80E-06	0.00012947	2.796058485

Gene	Log fold change	Average Expression	t	P-value	Adjusted P-value	B
ETV5	-0.325378823	5.039797635	-4.791441922	7.91E-06	0.000130947	2.815633115
DCAF17	-0.285629369	3.617658571	-4.788777465	7.99E-06	0.000132046	2.952901562
RBM5	0.332086684	4.582961735	4.788690212	7.99E-06	0.000132046	2.863430244
TWISTNB	-0.422315926	4.149839235	-4.786230535	8.07E-06	0.000133151	2.877270787
PRMT9	0.259657082	2.537940735	4.784627008	8.12E-06	0.000133825	3.057582803
SUSD6	0.301961367	4.48969887	4.783455691	8.16E-06	0.00013428	2.833303795
ENOX2	-0.172862399	4.325429542	-4.782851051	8.18E-06	0.000134444	2.841609955
ARHGAP10	0.242398943	5.192340943	4.781435105	8.22E-06	0.000135028	2.767542806
ARMCX2	0.168688368	6.10288627	4.779008058	8.30E-06	0.000136141	2.734232304
RNF182	-0.650478226	0.460033987	-4.775917056	8.40E-06	0.000137612	3.381263517
ALPK1	-0.267532258	3.877307704	-4.774871534	8.43E-06	0.000138014	2.883609553
MKI67	-0.605731728	4.849061925	-4.770325776	8.58E-06	0.000140284	2.764928963
JMJD6	-0.401107208	4.369266347	-4.768197588	8.65E-06	0.000141277	2.841693269
C2orf69	-0.252645842	4.739995909	-4.765341632	8.74E-06	0.000142675	2.743438813
FIGNL1	-0.353745348	2.316710624	-4.763950165	8.79E-06	0.000143281	3.074895773
ZFP2	0.696406647	-0.631515057	4.760764985	8.90E-06	0.00014488	3.367906063
MIS12	-0.23247354	3.776767353	-4.759735447	8.93E-06	0.000145294	2.811578297
BTG1	0.236732123	6.794363827	4.757158571	9.02E-06	0.000146574	2.656714134
TESK2	0.377953061	1.521625108	4.755655267	9.07E-06	0.000147101	3.143918657
NFIB	-0.378569539	4.516170979	-4.755734602	9.07E-06	0.000147101	2.730949263
KLHDC1	0.497982815	0.626942896	4.753748161	9.14E-06	0.000147858	3.272919504
RNF139	-0.196125534	5.456104894	-4.753967277	9.13E-06	0.000147858	2.666810949
FAM136A	0.168748871	4.758417162	4.753160924	9.16E-06	0.000148032	2.693659258
HOMER1	-0.488381026	2.313763299	-4.751931602	9.20E-06	0.000148569	2.938825703
TTC7A	0.196092882	5.419718498	4.75159997	9.21E-06	0.000148598	2.64080051
UFSP2	0.167303899	4.373292972	4.750469579	9.25E-06	0.000148922	2.722091974
TOR3A	0.168259759	4.77197474	4.75049516	9.25E-06	0.000148922	2.686310265
COBLL1	0.474653853	3.812922996	4.749815277	9.28E-06	0.000149135	2.745304563
ZFHX2	0.506088906	0.566619232	4.749073755	9.30E-06	0.00014924	3.17651616
MICALL2	0.424801732	3.966483063	4.749208709	9.30E-06	0.00014924	2.790234402
BCL2L13	0.142537872	5.734815929	4.747520142	9.36E-06	0.000149968	2.626844177
ZNF542P	-0.30960876	2.900199118	-4.746470001	9.40E-06	0.00015041	2.867620018
KAT5	-0.167043236	4.366403128	-4.742990785	9.52E-06	0.000152098	2.694520714
ELK4	-0.269810747	5.430342609	-4.743183872	9.51E-06	0.000152098	2.619915734
NUP35	-0.321020953	2.40407387	-4.740088044	9.63E-06	0.000153629	2.944371779
TRMT12	-0.224508774	3.284047589	-4.736041359	9.78E-06	0.000155854	2.787893009
TICRR	-0.557748659	0.834241833	-4.734188836	9.85E-06	0.000156793	3.112968258
AP5Z1	0.194470322	5.424633145	4.732528024	9.91E-06	0.000157622	2.578258162
USP20	0.181819608	4.269455515	4.73090095	9.97E-06	0.000158436	2.649353749
SLC43A1	0.270318387	5.143887895	4.730381344	9.99E-06	0.000158582	2.586610721
RAB32	-0.18523099	6.104218277	-4.728911002	1.00E-05	0.000159306	2.551523972
PSPH	-0.228427929	3.635333007	-4.728097849	1.01E-05	0.000159632	2.72514791
TCF12	-0.313236346	6.687911214	-4.725608496	1.02E-05	0.000160984	2.536921917
CCT4	0.112810124	7.480936256	4.725310376	1.02E-05	0.000160998	2.557055979
DIAPH2	-0.2273064	4.896775412	-4.724146237	1.02E-05	0.00016152	2.573289551
RPF2	-0.162142702	5.311659101	-4.723910333	1.02E-05	0.00016152	2.551221958
CEP131	0.399148853	3.092239795	4.719745453	1.04E-05	0.000163759	2.79559741
INPPL1	0.183727463	6.102900878	4.720016477	1.04E-05	0.000163759	2.520562381
ARF6	-0.198321786	6.576744563	-4.717586664	1.05E-05	0.000164938	2.507267433
METAP1D	-0.264087086	2.506577814	-4.717286938	1.05E-05	0.000164955	2.857715928
PRELID3B	-0.18328056	5.026968209	-4.714864797	1.06E-05	0.000166309	2.537301133
CREBZF	-0.409818004	3.287571038	-4.711517839	1.07E-05	0.000168265	2.665875123
CCT7	0.172339171	7.621543462	4.711082882	1.08E-05	0.000168369	2.509300253
MFSD2A	-0.812870795	1.234575099	-4.704771763	1.10E-05	0.000172101	3.105431579
GATAD2B	-0.233338863	4.585623012	-4.704898356	1.10E-05	0.000172101	2.50332578
IFIT5	-0.190995812	5.293657265	-4.7038065	1.11E-05	0.000172554	2.481988069
AP3M2	-0.264193928	2.934245384	-4.703503219	1.11E-05	0.000172574	2.686199608
URB2	-0.602169718	3.619618995	-4.702799246	1.11E-05	0.000172858	2.645434006
BCKDK	0.205298084	5.512009376	4.700478678	1.12E-05	0.000174209	2.461225929
SLC35A5	-0.205063807	5.087413857	-4.697326037	1.13E-05	0.000176126	2.468719286
SH3PXD2B	-0.369922565	8.009816068	-4.694344144	1.15E-05	0.000177949	2.47367599
SEC14L1	-0.163584108	5.957519167	-4.693058633	1.15E-05	0.000178635	2.417406611
CCDC34	0.271013707	3.224456663	4.691874589	1.16E-05	0.000179256	2.637604685
CRY2	-0.255021422	4.576926977	-4.686314456	1.18E-05	0.000182891	2.470304305
TBC1D19	-0.234949108	3.220573574	-4.684130535	1.19E-05	0.000184223	2.62728296
BAG1	0.264707218	5.768153532	4.68280941	1.20E-05	0.000184959	2.385522301
TBC1D23	-0.294822728	5.309770981	-4.682389435	1.20E-05	0.000185064	2.396938466
DYRK1B	0.261296767	4.087965791	4.680842623	1.21E-05	0.000185962	2.465369505
CCDC85B	0.432245833	6.94206842	4.680217791	1.21E-05	0.000186213	2.371223467
EXOC6B	-0.268580881	5.194681598	-4.679425137	1.21E-05	0.000186583	2.39493406

Gene	Log fold change	Average Expression	t	P-value	Adjusted P-value	B
PTPN13	-0.303707959	6.833740535	-4.677804875	1.22E-05	0.000187541	2.363953322
YBX3	0.221035622	8.427965335	4.677220395	1.22E-05	0.000187765	2.421397556
PITHD1	0.221412385	4.953223653	4.676374241	1.23E-05	0.000188177	2.400854349
SHROOM4	0.572809899	1.326717819	4.672168411	1.25E-05	0.000191006	2.813592941
ZNF843	0.400568038	1.165968751	4.668275983	1.26E-05	0.000193646	2.834829642
ZNF584	-0.247000279	3.594916629	-4.66719396	1.27E-05	0.000193791	2.474669142
FAM102B	-0.346795478	4.725748207	-4.667007883	1.27E-05	0.000193791	2.387677184
KLF13	-0.390779494	6.748194258	-4.667504553	1.27E-05	0.000193791	2.328246076
ZFP36	0.583914956	6.422503888	4.667376133	1.27E-05	0.000193791	2.326937284
ZDHHC8	0.14125792	5.922642323	4.661498571	1.30E-05	0.000197675	2.305608376
SOX8	-1.097365731	-0.510047906	-4.660956853	1.30E-05	0.000197881	3.029951165
MTO1	0.194387015	4.091398572	4.660634943	1.30E-05	0.000197922	2.400640169
TEAD4	-0.331309643	3.580425489	-4.658448913	1.31E-05	0.000199365	2.468152049
BOLA3-AS1	0.363315876	1.389448032	4.651896753	1.35E-05	0.000203787	2.740073758
ABCF3	0.148349169	5.711367533	4.651890725	1.35E-05	0.000203787	2.270853459
ATP1B3	0.132253835	6.467787381	4.651850607	1.35E-05	0.000203787	2.265043955
G0S2	-0.688420726	1.688798662	-4.64902134	1.36E-05	0.000205771	2.682780464
ZNF710	-0.312134204	3.118340092	-4.647928075	1.37E-05	0.000206416	2.458016607
CCDC91	-0.173715493	4.149659278	-4.644301347	1.39E-05	0.000209055	2.358341242
PNRC1	0.24251043	6.351458449	4.642673908	1.39E-05	0.000210133	2.231461844
HNRNPK	0.113398695	8.373261462	4.641610375	1.40E-05	0.000210768	2.297803669
APOBEC3G	0.379781398	2.408273822	4.639663669	1.41E-05	0.000212111	2.590906728
NOL4L	-0.267728726	3.283901799	-4.635486212	1.43E-05	0.000215268	2.35480463
RANBP1	0.167771377	4.415466222	4.633213939	1.44E-05	0.000216906	2.274394462
PPP4R1	-0.338810982	5.69961015	-4.6318491	1.45E-05	0.000217808	2.200249241
NNMT	-0.305084069	8.739822523	-4.630179739	1.46E-05	0.000218966	2.251046001
RAB11FIP4	0.541560706	1.170652976	4.627511986	1.48E-05	0.000220742	2.720255472
TSPYL4	-0.280705506	4.680265176	-4.627716732	1.47E-05	0.000220742	2.231797318
C12orf45	0.384325471	3.008769857	4.626567949	1.48E-05	0.000221309	2.429501003
U2AF2	0.142827681	6.976453233	4.624526823	1.49E-05	0.000222798	2.173021397
HSDL1	-0.209107813	4.146225073	-4.623841278	1.50E-05	0.000223153	2.265132963
PELI2	0.378403138	3.372986083	4.62207473	1.51E-05	0.000224247	2.400125092
SLC27A1	0.15315947	5.667226714	4.622019042	1.51E-05	0.000224247	2.168669923
MTSS1L	-0.293866734	6.369706764	-4.621669376	1.51E-05	0.000224321	2.155170192
SHB	-0.358097626	2.379781588	-4.62092053	1.51E-05	0.000224734	2.508256699
PRKD2	0.187412686	4.668302801	4.617469644	1.53E-05	0.000227451	2.187242812
PBRM1	-0.341078676	5.943743675	-4.613069203	1.56E-05	0.000231024	2.129603406
ATAD3A	0.242845919	4.426195674	4.612433593	1.56E-05	0.00023135	2.192495281
WIPF1	-0.236936004	6.825158531	-4.611809709	1.57E-05	0.000231666	2.120235585
ZNF346	0.191948637	3.178793408	4.610616844	1.57E-05	0.000232253	2.331897438
RABIF	-0.192800308	3.767015032	-4.610806353	1.57E-05	0.000232253	2.275926662
FTSJ1	0.154816103	5.010764771	4.610244731	1.57E-05	0.000232351	2.150363969
TMUB1	0.298008721	4.547735852	4.609822391	1.58E-05	0.000232493	2.193533838
MED29	0.154788159	6.188227727	4.608530239	1.59E-05	0.000233398	2.107744864
STIP1	0.172890244	7.143542889	4.607047536	1.59E-05	0.000234475	2.113650837
GNA13	-0.374521539	5.840941588	-4.605313631	1.60E-05	0.000235779	2.098149428
LMNB1	-0.421833188	3.308811724	-4.604943466	1.61E-05	0.000235878	2.290167309
KEAP1	0.182750861	6.327611378	4.601298845	1.63E-05	0.000238901	2.080866514
MOSPD1	0.251618875	3.921414029	4.60071792	1.63E-05	0.00023919	2.208234827
TRMT1L	0.263071373	3.999700328	4.600436615	1.63E-05	0.000239211	2.1536827
CLCN2	0.507511633	0.556052139	4.597938943	1.65E-05	0.000241053	2.691429719
PAOX	0.343971592	1.682053499	4.59788025	1.65E-05	0.000241053	2.521756592
CENPE	-0.460687818	2.42227836	-4.595374667	1.67E-05	0.000243098	2.43543142
CARMIL1	-0.329289713	3.254570003	-4.592827063	1.68E-05	0.000244962	2.24262804
FOS	1.415946829	5.295860894	4.592856819	1.68E-05	0.000244962	2.102309
MED25	0.208412763	4.946566717	4.59253372	1.68E-05	0.000244995	2.099946658
PI4K2A	-0.189239061	5.977470258	-4.592274852	1.68E-05	0.000244997	2.049751788
CBX5	-0.225460292	6.525932197	-4.591012985	1.69E-05	0.000245924	2.043790151
SLTM	-0.180750781	5.472935436	-4.588398539	1.71E-05	0.000248111	2.051133348
AAMP	0.21689077	6.180086521	4.587144134	1.72E-05	0.000249043	2.030310242
SMG6	0.343232662	5.2030081	4.584800383	1.73E-05	0.000251003	2.04405758
TOLLIP-AS1	0.72865442	-1.108287628	4.582268954	1.75E-05	0.000252913	2.771965262
TRDMT1	-0.263157193	2.474244428	-4.582284647	1.75E-05	0.000252913	2.34513188
EIF4ENIF1	-0.263814046	4.348288526	-4.580085527	1.76E-05	0.000254749	2.077329007
ETS1	-0.359194471	6.103241171	-4.577720524	1.78E-05	0.000256774	2.005427063
ZADH2	-0.215805754	4.528999418	-4.576184577	1.79E-05	0.00025801	2.044338705
TNFRSF12A	-0.482964735	6.06408896	-4.573601028	1.81E-05	0.000260024	1.99637171
OSBPL5	0.193633761	5.922964281	4.573780427	1.81E-05	0.000260024	1.9861418
CDR2	-0.180501793	5.775345281	-4.573057956	1.81E-05	0.000260286	1.997779088
TCF20	-0.264100488	5.287808616	-4.572823969	1.81E-05	0.000260286	1.993681607

Gene	Log fold change	Average Expression	t	P-value	Adjusted P-value	B
SH2B2	-0.448769948	1.939205095	-4.571334303	1.82E-05	0.000260885	2.36775821
ECT2	-0.498230553	3.420371016	-4.571442286	1.82E-05	0.000260885	2.17687573
PLCL2	0.350743679	3.926483013	4.571885559	1.82E-05	0.000260885	2.094409273
TRMT10C	-0.214664058	4.573374928	-4.571196088	1.82E-05	0.000260885	2.062225024
CPSF7	-0.222840924	6.007773989	-4.570681987	1.83E-05	0.00026114	1.974067619
CPT2	0.270844637	2.309152066	4.570024498	1.83E-05	0.000261536	2.277676565
C5orf24	-0.299774453	5.657242905	-4.568251018	1.84E-05	0.000263031	1.969256297
PANK1	0.376856183	2.497608233	4.567718744	1.85E-05	0.000263307	2.150729262
KIAA1958	-0.413946911	2.569847404	-4.5660596	1.86E-05	0.000264698	2.290169581
MIEF2	-0.227085457	2.934980279	-4.563024243	1.88E-05	0.000267472	2.158022611
TACO1	0.227317572	4.176246346	4.562029469	1.89E-05	0.000268218	2.04117497
ATMIN	-0.139114549	6.299980029	-4.56042925	1.90E-05	0.000269575	1.932351942
PDCD1LG2	-0.315494538	3.443938333	-4.559527257	1.91E-05	0.000270233	2.105698181
POLR2A	0.224836688	7.800904758	4.558961973	1.91E-05	0.000270304	1.969004734
NFKB1	-0.264003293	6.041470767	-4.558954397	1.91E-05	0.000270304	1.927695715
PDSS2	-0.232272364	3.87787076	-4.557533068	1.92E-05	0.000271491	2.073532056
ETV1	-0.588364646	5.020864707	-4.556956191	1.92E-05	0.000271567	1.967375391
XXYLT1	0.169548338	5.328633658	4.556978898	1.92E-05	0.000271567	1.943774285
LARP4B	-0.260043056	5.082449967	-4.552112247	1.96E-05	0.000276276	1.931529752
RPUSD3	0.319224812	3.567499787	4.548975111	1.98E-05	0.000279275	2.073077498
STX2	-0.181317167	4.896285176	-4.545480444	2.01E-05	0.000282684	1.927227653
EZR	0.237553114	4.786240728	4.539897746	2.05E-05	0.0002882	1.910229837
YWHAG	-0.243940538	7.748982635	-4.539809125	2.05E-05	0.0002882	1.891781913
PHF20	-0.378942443	5.158402586	-4.539548736	2.05E-05	0.000288212	1.887602419
HNMT	-0.211408198	4.908326028	-4.538415537	2.06E-05	0.000289165	1.908765395
SSSCA1	0.253657467	3.801978222	4.537578507	2.07E-05	0.000289801	1.994548059
ZNF26	-0.300388951	2.644310353	-4.53329189	2.10E-05	0.000294203	2.157704725
XYLT2	0.227255652	4.736358876	4.532774862	2.11E-05	0.000294498	1.883554871
CDC42EP4	0.227748125	5.93872219	4.526461198	2.16E-05	0.000301239	1.812065295
SPATA13	-0.313323673	3.876472371	-4.524328484	2.17E-05	0.000303364	1.928321662
EIF3B	0.129055585	7.872466218	4.523094818	2.18E-05	0.00030448	1.843156378
SEC13	0.187043322	7.107981143	4.520015203	2.21E-05	0.000307712	1.797438847
MRPS26	0.335948046	4.395126871	4.518170605	2.22E-05	0.000309548	1.851899096
TOB2	-0.393283306	6.217899311	-4.51748204	2.23E-05	0.000310057	1.778193088
EIF6	0.236338301	5.825795846	4.515492079	2.25E-05	0.000312077	1.777040162
LRRC6	-0.285203187	1.644741979	-4.514958341	2.25E-05	0.00031241	2.267926446
MAD2L2	0.290249617	4.65037539	4.513804609	2.26E-05	0.000313412	1.83398445
CHSY1	-0.339624129	5.739827884	-4.513603964	2.26E-05	0.000313412	1.777344379
DCUN1D4	-0.232875753	4.840408347	-4.512886579	2.27E-05	0.000313962	1.803420432
DNM2	0.123129057	6.598203373	4.512582383	2.27E-05	0.000314029	1.760513023
RAB3D	-0.303199348	2.009851896	-4.509667991	2.29E-05	0.000316842	2.108840752
NAPG	-0.213963212	3.779086621	-4.509571759	2.30E-05	0.000316842	1.906600026
STAT2	-0.242524005	6.916260039	-4.509449496	2.30E-05	0.000316842	1.750941737
TRMT6	-0.199531531	3.625830464	-4.508231137	2.31E-05	0.000317991	1.937685265
TAF1B	-0.231332423	3.600098847	-4.507573696	2.31E-05	0.000318479	1.921193872
MFSD4A	1.336570765	-3.010213319	4.506003863	2.33E-05	0.000320053	2.484527444
TRIM35	0.195211228	4.745762943	4.500711071	2.37E-05	0.000326068	1.762180805
NLRP1	0.17052384	4.995874754	4.500509476	2.37E-05	0.000326068	1.760991374
KCNJ15	-0.310625912	3.214059481	-4.499850148	2.38E-05	0.000326081	1.963533093
EIF5	-0.329982064	7.200811647	-4.499811908	2.38E-05	0.000326081	1.734804374
APOL2	0.233080531	5.502636902	4.499764375	2.38E-05	0.000326081	1.72135325
UBE2F	-0.152784308	3.920904371	-4.49925022	2.39E-05	0.000326408	1.854439429
CLIP1	-0.406050073	6.734265497	-4.493761817	2.43E-05	0.000332835	1.696050128
ULK1	0.209280947	5.493694662	4.491826237	2.45E-05	0.000334934	1.710211268
ZFP30	-0.393829055	2.578094895	-4.491274028	2.46E-05	0.000335318	1.911894209
RAB27A	-0.232781363	4.563711705	-4.489635845	2.47E-05	0.00033706	1.747676809
GOT1	-0.259167961	5.087643078	-4.48891051	2.48E-05	0.000337664	1.717471567
MAP4K4	0.163633105	9.396904448	4.487806217	2.49E-05	0.000338745	1.789935998
SRF	-0.250333415	5.688321914	-4.486343002	2.50E-05	0.000340284	1.707142591
IDH2	0.211689283	5.363452337	4.484676588	2.52E-05	0.000341779	1.683668419
ABI2	-0.13959438	5.461755579	-4.484768121	2.52E-05	0.000341779	1.677855962
CIR1	0.201944921	5.297025115	4.483920579	2.53E-05	0.000342431	1.681321947
HSPA6	1.197747954	-2.915794262	4.483289624	2.53E-05	0.000342926	2.410062168
KIAA1109	-0.458175432	5.430957883	-4.482292858	2.54E-05	0.000343888	1.675221485
ARHGEF6	-0.229181228	4.944442994	-4.481943445	2.54E-05	0.000344025	1.698686149
BRF1	0.200169543	4.718201846	4.481581168	2.55E-05	0.000344179	1.713116396
USP10	-0.26571987	5.554497513	-4.480422247	2.56E-05	0.000345353	1.652627939
ODF2L	-0.194092277	4.121733651	-4.480067024	2.56E-05	0.000345499	1.768007872
APEX2	0.184010308	4.288411392	4.479013545	2.57E-05	0.000346542	1.725055452
SEPT5	0.233702869	4.539338269	4.477545485	2.59E-05	0.000348123	1.710289233

Gene	Log fold change	Average Expression	t	P-value	Adjusted P-value	B
FMNL3	0.174009847	5.019458685	4.476367009	2.60E-05	0.000349336	1.659024124
SRPRA	0.103191091	7.521943679	4.474166524	2.62E-05	0.000351885	1.647980179
TMEM216	0.213822211	3.416335662	4.471228064	2.65E-05	0.000355414	1.811159784
GAREM2	0.222903966	3.417955551	4.470994681	2.65E-05	0.000355414	1.794796391
C12orf29	-0.21339774	3.707494347	-4.470189638	2.66E-05	0.000356159	1.768069245
GNL3	-0.254144999	5.852258361	-4.469461989	2.66E-05	0.000356804	1.607552128
C19orf25	0.214894144	3.626277342	4.46921182	2.67E-05	0.000356818	1.761849962
CSTF1	-0.148988433	4.762728841	-4.468447617	2.67E-05	0.000357512	1.661261331
SDF2L1	0.274243048	3.443700475	4.466088432	2.70E-05	0.000360332	1.786285063
ELF4	-0.293378956	5.821034394	-4.465579991	2.70E-05	0.000360691	1.595544251
LFNG	-0.870292169	2.359853396	-4.463333264	2.73E-05	0.000362422	1.959850243
PAN2	-0.25096915	2.813105424	-4.463721027	2.72E-05	0.000362422	1.881741248
CDC25B	-0.242742352	7.025777452	-4.463543804	2.72E-05	0.000362422	1.59984908
YKT6	0.102673231	7.034687745	4.463812069	2.72E-05	0.000362422	1.595660128
SGMS2	-0.618355549	5.100412531	-4.463084173	2.73E-05	0.000362437	1.618716349
NCBP3	-0.204686422	4.854833033	-4.462490714	2.73E-05	0.000362914	1.646428988
RPS6KA1	0.484982285	0.379179437	4.458692565	2.77E-05	0.00036773	2.154029432
PAXIP1-AS2	-0.343769334	1.931201846	-4.457421186	2.79E-05	0.000368895	2.005786575
MCMBP	-0.194987716	5.982667319	-4.457362998	2.79E-05	0.000368895	1.563294906
GDPGP1	0.380946657	0.662493425	4.452834335	2.83E-05	0.0003748	2.096489454
MAP9	-0.415212871	3.284135657	-4.45032822	2.86E-05	0.000377959	1.760039004
QKI	-0.242467887	7.219208595	-4.449999228	2.86E-05	0.000378088	1.552720036
IL1R1	-0.277547055	9.240111281	-4.449242499	2.87E-05	0.000378153	1.643451496
NUP93	0.152213309	5.097048503	4.449408362	2.87E-05	0.000378153	1.565572689
ADRM1	0.253452193	5.999633909	4.4496833	2.87E-05	0.000378153	1.537552634
RNF38	-0.331028281	4.453090313	-4.447724032	2.89E-05	0.00037995	1.600507127
OPA3	-0.271200393	4.615059307	-4.446504142	2.90E-05	0.000381335	1.586290794
RGS4	-0.510902697	4.915745894	-4.444574949	2.92E-05	0.000383729	1.597200484
TAX1BP3	0.229480262	4.68262904	4.444117351	2.93E-05	0.000384044	1.577774782
ARMC5	0.278235736	3.825049993	4.443019028	2.94E-05	0.00038527	1.719767927
NLRX1	0.192014936	4.660294321	4.442367757	2.95E-05	0.000385863	1.573407538
RETSAT	0.144518912	5.984234506	4.441623799	2.95E-05	0.000386589	1.510541506
NUDCD1	-0.243519549	3.611852437	-4.440508156	2.97E-05	0.000387849	1.651876542
VPS8	-0.172517734	5.310319636	-4.44013567	2.97E-05	0.000388046	1.533245893
TMCO6	0.327658414	1.726678436	4.437932172	2.99E-05	0.00039054	1.95325062
CCDC28B	0.367499406	2.225801655	4.438009691	2.99E-05	0.00039054	1.887007154
ANKRD13C	-0.205872484	5.02597667	-4.436147619	3.01E-05	0.000392782	1.52659061
LOXL1	0.235810147	7.685087268	4.435846057	3.02E-05	0.00039288	1.516524668
EHMT2	0.21057269	5.895375297	4.435575561	3.02E-05	0.000392933	1.489917532
CEP85	-0.343316556	1.97111822	-4.434233406	3.04E-05	0.000394544	1.809560837
ZNF136	-0.219712741	2.64367285	-4.430437458	3.08E-05	0.000399766	1.786490565
SIAH2	-0.185447869	4.965326035	-4.429120889	3.09E-05	0.000401367	1.508761646
CSGALNACT2	-0.425670689	5.002651203	-4.42784161	3.11E-05	0.000402918	1.513595986
JADE2	-0.288774775	5.012797156	-4.426458807	3.12E-05	0.000404631	1.475870988
KLF11	-0.3558503	4.000951792	-4.425351528	3.14E-05	0.000405589	1.608521183
GALNT15	-0.406402829	5.807921695	-4.425395664	3.14E-05	0.000405589	1.473568075
CTDSP2	-0.291379689	8.313114621	-4.42244111	3.17E-05	0.000409613	1.494497607
NAA15	-0.34788284	5.347745619	-4.421701007	3.18E-05	0.000410381	1.452566835
GLIS1	0.480002277	1.759228295	4.419195006	3.21E-05	0.00041348	1.912633703
PSMD2	0.109217684	7.615390256	4.419224595	3.21E-05	0.00041348	1.454978603
GMEB2	-0.155268481	4.300655369	-4.418196063	3.22E-05	0.000414296	1.53569227
DSCR3	-0.162275224	5.823114205	-4.418225602	3.22E-05	0.000414296	1.433377377
CUL9	-0.223158706	4.180257561	-4.416104098	3.25E-05	0.000417146	1.538691085
PJA1	-0.149978393	5.217616821	-4.415551745	3.25E-05	0.000417639	1.442196585
CDKAL1	-0.213288748	3.588642609	-4.414119798	3.27E-05	0.00041949	1.586400237
ZNF783	-0.305501134	2.476198636	-4.413274923	3.28E-05	0.000420439	1.748363152
LOC389641	0.817448513	-1.500220927	4.411849995	3.30E-05	0.000422292	2.199485132
SNCAIP	0.479186973	0.814193648	4.409397321	3.33E-05	0.00042576	1.951306477
FZD7	-0.273629462	6.606377244	-4.408949972	3.33E-05	0.0004261	1.393147275
FAM50B	-0.349708361	2.674194203	-4.408583132	3.34E-05	0.000426314	1.724942101
DYNC1LI1	-0.160632363	5.299706318	-4.407759633	3.35E-05	0.000427246	1.41216076
HSPA1A	0.2317005	5.250289599	4.406993257	3.36E-05	0.00042809	1.408031518
CYP1B1	-0.273616732	9.171803602	-4.404960163	3.38E-05	0.000430939	1.488639389
ETFBKMT	0.386437522	0.872196008	4.4018091	3.42E-05	0.000435227	1.956442646
ELK1	-0.149675553	5.884369928	-4.401963932	3.42E-05	0.000435227	1.370002786
MIR34A	0.738200477	0.706988747	4.399851419	3.45E-05	0.000438002	1.943328482
NME1	0.301542898	4.183521776	4.398307632	3.47E-05	0.000440124	1.454031665
OPLAH	0.192969529	4.170025649	4.398059455	3.47E-05	0.000440154	1.461921918
CSRNP3	1.619919396	-2.732934304	4.396798824	3.48E-05	0.000441827	2.131542298
SRM	0.236716801	7.019175171	4.396016028	3.49E-05	0.000442727	1.352724234

Gene	Log fold change	Average Expression	t	P-value	Adjusted P-value	B
SAV1	0.21651012	6.157821536	4.395574494	3.50E-05	0.000443073	1.346260625
FRMD5	-0.569290531	1.257888259	-4.392384241	3.54E-05	0.000447637	1.832877839
SERPINB9	-0.451072607	1.529848247	-4.392328789	3.54E-05	0.000447637	1.811957909
WSB2	-0.23640551	5.702922819	-4.391700767	3.55E-05	0.000448295	1.340180897
HIST1H3E	0.686909597	-0.536527095	4.389003692	3.59E-05	0.000452	2.052991089
FBXO11	-0.303143778	5.322493572	-4.389089843	3.58E-05	0.000452	1.341555196
CMAS	0.184234201	4.437472426	4.387978646	3.60E-05	0.000453325	1.40513109
EFNA5	-0.27225429	4.593930497	-4.386755251	3.62E-05	0.000454606	1.406763956
SUGT1	0.227540933	5.280697045	4.38692006	3.61E-05	0.000454606	1.341399426
TRIM44	-0.252019814	7.020193003	-4.384633021	3.64E-05	0.000457779	1.310550665
KLHL36	-0.312392635	5.194680456	-4.383779925	3.66E-05	0.000458832	1.333063704
ADPRH	0.371585437	1.874756938	4.383530396	3.66E-05	0.00045887	1.760193977
RAB12	0.125903133	5.66495698	4.383162747	3.66E-05	0.000459108	1.308473225
PLSCR4	-0.236224299	5.777041196	-4.381408298	3.69E-05	0.000461689	1.299992019
DUBR	0.222507673	2.413173462	4.380874631	3.69E-05	0.000462026	1.635256028
REV1	-0.266661128	3.91669958	-4.380756257	3.70E-05	0.000462026	1.403060504
RRAGC	-0.133996389	5.70905231	-4.379159657	3.72E-05	0.000464355	1.292783153
ZNF234	-0.347673671	2.574632038	-4.378549176	3.73E-05	0.000465011	1.56209736
MOB4	-0.202956686	4.220203624	-4.377836311	3.74E-05	0.000465119	1.392642716
CLMP	-0.166065716	8.371915616	-4.377808881	3.74E-05	0.000465119	1.345097914
ZC3H14	-0.158460436	5.455031886	-4.378153358	3.73E-05	0.000465119	1.299078151
COG7	0.202381385	3.509966312	4.376244452	3.76E-05	0.000467409	1.49753781
RADIL	0.467431895	1.702855776	4.370218847	3.84E-05	0.000477306	1.799826525
PAXIP1	-0.283210907	2.953098513	-4.370077964	3.84E-05	0.000477306	1.48448396
PPP1R14C	0.813626637	0.128396647	4.369044133	3.86E-05	0.000478722	1.912659182
SNRPA1	0.313067767	2.520385501	4.368145319	3.87E-05	0.000479906	1.547778783
PAFAH1B1	-0.30099176	6.874646025	-4.367382522	3.88E-05	0.000480853	1.250964859
SLC25A42	0.290531353	2.120043608	4.365720879	3.91E-05	0.000483391	1.649972868
OMA1	-0.231989312	3.087921336	-4.364982846	3.92E-05	0.000483507	1.530486548
CCNF	-0.374421499	2.641123585	-4.365083519	3.91E-05	0.000483507	1.497963731
LMBR1L	0.185315316	3.495223172	4.365330499	3.91E-05	0.000483507	1.403918682
ELP3	0.130490533	4.924137175	4.364200639	3.93E-05	0.000484497	1.270268814
LSM11	-0.363096134	2.549601979	-4.361934643	3.96E-05	0.000488133	1.500740846
RAB7B	0.308228887	3.951820994	4.360582984	3.98E-05	0.000490153	1.338574069
TRO	-0.329443317	3.479643341	-4.358645213	4.01E-05	0.000492231	1.384612817
SGTB	-0.220514937	4.091010876	-4.358971414	4.00E-05	0.000492231	1.322523254
RASA1	-0.364149898	4.766646739	-4.35853507	4.01E-05	0.000492231	1.257990087
ARFGAP3	-0.327759398	5.557324152	-4.358964018	4.00E-05	0.000492231	1.220871383
SNX29	-0.372254785	6.298805708	-4.355936744	4.05E-05	0.000496528	1.203134853
CCNJ	-0.249403473	2.538351003	-4.354388726	4.07E-05	0.000498941	1.571246142
NIPAL2	-0.271600442	5.857101226	-4.353643068	4.08E-05	0.000499896	1.201907782
AJUBA	-0.380140964	4.579324189	-4.351960306	4.11E-05	0.000502573	1.285109557
DNAJC27-AS1	0.787094085	-1.633319723	4.351728877	4.11E-05	0.00050259	2.004642718
MARCKSL1	0.279975603	5.946022783	4.350927895	4.12E-05	0.000503656	1.188476751
ATP6AP1	0.138919981	6.981062297	4.34863505	4.16E-05	0.000507483	1.183092902
RABEP1	-0.169421953	6.593265251	-4.347854683	4.17E-05	0.00050852	1.175907256
CSRP1	0.203215723	7.230610016	4.346765438	4.19E-05	0.000510135	1.187784195
KIF1B	-0.531867678	6.169122652	-4.345206631	4.21E-05	0.000512634	1.165968218
PRRX1	-0.195079602	9.256175293	-4.342160087	4.26E-05	0.000517952	1.260079674
NECTIN3	-0.455110802	5.015483106	-4.341121279	4.27E-05	0.000519501	1.189158898
PARP11	-0.184407813	3.419180643	-4.340586756	4.28E-05	0.000520096	1.353488849
SLC9A1	0.173338189	6.583520309	4.337033811	4.34E-05	0.000526255	1.137901371
ASAP3	0.167937103	6.725121096	4.336920275	4.34E-05	0.000526255	1.13582762
HCFC2	-0.360643486	4.034823166	-4.335550533	4.36E-05	0.000528467	1.203144942
EOGT	-0.307114402	4.095278724	-4.334041737	4.38E-05	0.000530957	1.226115556
FHOD1	0.278202282	5.55138205	4.33266152	4.41E-05	0.000533209	1.125149496
USP3	-0.16451893	3.861447233	-4.332241881	4.41E-05	0.000533597	1.271051317
PLPP3	-0.306777824	9.332650706	-4.331893339	4.42E-05	0.000533847	1.231328038
CCSAP	-0.352612279	2.957839922	-4.331414906	4.43E-05	0.000534351	1.333687018
ZNF638	-0.181953739	5.905801067	-4.331115102	4.43E-05	0.000534507	1.12080008
ZNF449	-0.317118157	2.329986081	-4.330578996	4.44E-05	0.000535125	1.355892
FAM133B	-0.311644638	1.308945101	-4.327341342	4.49E-05	0.000541047	1.611445977
BLOC1S4	-0.198586703	3.532735878	-4.326981868	4.50E-05	0.000541324	1.293501477
KLHL9	-0.302916724	6.145302146	-4.323856136	4.55E-05	0.000547089	1.091871326
NOC2L	0.154681237	5.55895829	4.320595719	4.60E-05	0.000553184	1.091254185
FSIP1	-0.544237266	0.233365003	-4.320043861	4.61E-05	0.000553856	1.814798865
BAG5	-0.288660007	5.593550964	-4.318854974	4.63E-05	0.000555816	1.091473496
ARL15	-0.214430236	3.427625651	-4.318575654	4.64E-05	0.000555939	1.283895647
IFNGR1	-0.14786397	5.757372947	-4.317940649	4.65E-05	0.000556783	1.084533635
STOX2	0.647290558	-0.863129416	4.316600597	4.67E-05	0.000558619	1.845319508

Gene	Log fold change	Average Expression	t	P-value	Adjusted P-value	B
MARCH5	-0.136631298	5.394881261	-4.316640154	4.67E-05	0.000558619	1.090912838
ZMYM2	-0.345952903	5.097287031	-4.315411068	4.69E-05	0.000560598	1.079439896
CUL5	-0.315331094	4.752082652	-4.313306456	4.73E-05	0.000564461	1.107240221
CDC20	-0.483815269	2.904816015	-4.312872717	4.74E-05	0.000564905	1.363100434
ETF1	-0.196189953	7.215437237	-4.311838347	4.75E-05	0.000566587	1.06504241
C4orf48	0.463540687	2.505846463	4.310955245	4.77E-05	0.000567789	1.392722851
FBXO45	-0.301148228	3.83216195	-4.310821218	4.77E-05	0.000567789	1.191674953
SYNE3	-0.285816644	5.747099827	-4.308669863	4.81E-05	0.000571793	1.047114839
SLC25A5	0.14642138	6.776025535	4.308455325	4.81E-05	0.000571793	1.038667451
DOCK11	-0.253949659	5.139383708	-4.306310242	4.85E-05	0.000575364	1.064580083
FBXL5	-0.134708	5.635445994	-4.306457428	4.85E-05	0.000575364	1.03848879
MIR100HG	-0.171055241	7.012308717	-4.305862206	4.86E-05	0.000575848	1.03399066
MON1A	0.2389412	3.87618841	4.304636137	4.88E-05	0.000577966	1.158458997
PRKAR2A-AS1	0.56481894	-0.817053585	4.303326289	4.90E-05	0.000580269	1.81277259
GATA2	-0.482350131	3.218539868	-4.302023829	4.93E-05	0.000582564	1.269675222
RUSC2	0.208416248	5.867802485	4.301773725	4.93E-05	0.000582637	1.025953204
USP39	0.12117154	5.748653165	4.299954091	4.96E-05	0.000586042	1.01495626
CYTH1	-0.228316161	3.982642941	-4.298345338	4.99E-05	0.000589016	1.120615551
CDK14	-0.28828374	5.423136117	-4.298108232	5.00E-05	0.000589062	1.019554099
KARS	0.178617613	7.080145036	4.294486833	5.06E-05	0.000596394	0.99688023
NCS1	0.127016554	6.745144247	4.293367395	5.08E-05	0.000598355	0.987834499
NECTIN1	0.237100138	4.913320088	4.292494852	5.10E-05	0.000599317	0.999561177
ASCC3	0.405998068	6.341735674	4.292510261	5.10E-05	0.000599317	0.98125514
ZNF512B	-0.174328454	4.961439968	-4.291457988	5.12E-05	0.000601108	1.019185481
SNRNP27	0.199620478	4.236216915	4.288895809	5.17E-05	0.00060625	1.079377265
ATG12	-0.130749599	5.833501327	-4.288162229	5.18E-05	0.000607393	0.972275221
BTBD3	-0.387869056	3.221702382	-4.286890858	5.21E-05	0.000609727	1.19833072
SF3A3	0.102710536	6.104155828	4.28623452	5.22E-05	0.000610705	0.959746541
DLGAP5	-0.616175294	2.139291775	-4.285015627	5.24E-05	0.000612935	1.392636341
SDCBP2-AS1	-0.356785571	1.120915585	-4.284267627	5.25E-05	0.000613647	1.543532279
DOT1L	-0.355612794	4.476610279	-4.284354549	5.25E-05	0.000613647	1.042131005
TCF4	-0.396919529	6.941735363	-4.282560263	5.29E-05	0.000616979	0.948324779
CCDC77	0.236965007	2.407474073	4.280883168	5.32E-05	0.000620261	1.28227609
GRWD1	-0.148388956	5.45606528	-4.280219883	5.33E-05	0.000621273	0.955998429
FAM26E	-0.341847087	4.81364623	-4.279508819	5.35E-05	0.000622395	1.006798925
KIAA1462	-0.517286381	7.075087872	-4.279251891	5.35E-05	0.000622494	0.942138354
TCHH	0.798167548	-1.869780875	4.277623661	5.38E-05	0.000624771	1.757566802
FAXDC2	0.205572248	4.699607346	4.277823874	5.38E-05	0.000624771	1.003889066
BOP1	0.254233913	5.170160483	4.277605573	5.38E-05	0.000624771	0.953169165
RBMXL1	-0.258157189	4.172779415	-4.271732073	5.50E-05	0.000637714	1.050995486
LCA5	0.372959203	1.982248428	4.271108796	5.51E-05	0.000638663	1.229662636
WDR35	-0.471397458	3.765736181	-4.269721281	5.54E-05	0.000641106	1.036344287
WDFY2	-0.271846023	4.56994492	-4.269629504	5.54E-05	0.000641106	0.975372101
DET1	0.20211642	2.602152071	4.26599532	5.61E-05	0.000649095	1.212501165
OSBPL6	0.306377611	3.195223668	4.265035439	5.63E-05	0.000650658	1.075909577
FBXO8	0.187391708	3.835099089	4.264906508	5.64E-05	0.000650658	1.029283763
MAGI2-AS3	-0.147530649	5.659663206	-4.262903022	5.68E-05	0.000654889	0.88705414
BCORL1	-0.447445578	4.2941112	-4.262666952	5.68E-05	0.000654947	0.911316539
MTSS1	0.407736773	3.733504322	4.260388391	5.73E-05	0.000659861	0.97837541
PSMD8	0.227135016	6.895910096	4.259853489	5.74E-05	0.000660633	0.871684053
CDK12	-0.453253449	5.773545984	-4.258591751	5.77E-05	0.000663146	0.871741655
PRPF19	0.126932363	6.646422122	4.257866109	5.78E-05	0.000664381	0.861067473
MEF2C	-0.39417299	3.014628543	-4.255623374	5.83E-05	0.000669277	1.119785266
PIBF1	0.161123255	4.022707839	4.254043026	5.86E-05	0.000672597	0.984525998
SPOPL	-0.294632166	3.835672486	-4.25316793	5.88E-05	0.000674213	0.991636492
SRRM3	0.304310925	2.876424743	4.251843914	5.91E-05	0.000675729	1.098012323
PGAM5	-0.180330825	3.799188359	-4.251704504	5.91E-05	0.000675729	0.972014531
RRP15	-0.192607203	4.392212933	-4.251749909	5.91E-05	0.000675729	0.929540199
PAF1	0.198416775	6.180383868	4.252265161	5.90E-05	0.000675729	0.842147288
SNORA65	1.019111506	-2.331330908	4.250936137	5.93E-05	0.000676581	1.670584217
SIPA1L1	-0.262977669	5.902498033	-4.251028564	5.93E-05	0.000676581	0.839934456
ABCF1	-0.146445282	6.995716803	-4.247982222	5.99E-05	0.000683318	0.832854087
POLB	0.257178498	2.247428308	4.24654777	6.02E-05	0.000686345	1.219819306
DNPEP	0.159886675	5.893853045	4.244384143	6.07E-05	0.000691203	0.816849849
WDR33	0.165966009	5.158983501	4.241771332	6.13E-05	0.000696539	0.84916304
WDR19	-0.168228417	5.283380199	-4.241746754	6.13E-05	0.000696539	0.828687231
STX12	-0.162861841	6.341301696	-4.241624596	6.13E-05	0.000696539	0.803148006
KLHL13	-0.521497889	-0.237431564	-4.240901365	6.15E-05	0.000697832	1.553226222
NRBP1	0.108504683	7.558028906	4.240214809	6.16E-05	0.000699036	0.826004396
TMEM101	0.239647875	4.289288071	4.239573343	6.18E-05	0.000700127	0.893913372

Gene	Log fold change	Average Expression	t	P-value	Adjusted P-value	B
TMEM67	-0.365239394	2.677748996	-4.238876491	6.19E-05	0.000701306	1.114849069
PSIP1	-0.131605008	6.136290999	-4.23869003	6.20E-05	0.000701306	0.794411556
ZBTB7C	1.174638664	-2.273065857	4.238384503	6.20E-05	0.000701551	1.633610963
RYK	-0.11247581	6.29346394	-4.237744193	6.22E-05	0.000702645	0.790575325
EML2	0.264307139	2.977948348	4.236522212	6.25E-05	0.00070469	1.058949615
PLXNA2	0.448328057	3.387141399	4.23657118	6.24E-05	0.00070469	0.974736956
RREB1	-0.289458039	4.55708321	-4.235581429	6.27E-05	0.000706194	0.838545144
SLC2A1	-0.355880043	4.850747086	-4.23551552	6.27E-05	0.000706194	0.819295586
MRPL18	0.278832829	5.158702655	4.234543894	6.29E-05	0.000708142	0.817180627
EXT1	-0.174922019	8.076924861	-4.233473698	6.31E-05	0.000710347	0.83608068
ZNF385D	-0.338929733	3.550484636	-4.230886748	6.37E-05	0.000715396	0.935486795
DTWD1	-0.147434925	5.053434572	-4.231168154	6.37E-05	0.000715396	0.812069183
CDC42BPA	-0.272428836	6.238744506	-4.230945797	6.37E-05	0.000715396	0.768164523
SYNCRIP	-0.234614733	7.560640125	-4.23026221	6.39E-05	0.000716473	0.792351009
SLFN5	-0.240766543	7.365251933	-4.229557601	6.40E-05	0.00071776	0.775167679
MVK	0.273650154	4.389544604	4.229126421	6.41E-05	0.000718201	0.817637524
MAP1S	-0.208493706	5.592577546	-4.228767958	6.42E-05	0.000718201	0.780067987
POLDIP2	0.11359829	6.569880676	4.228881073	6.42E-05	0.000718201	0.759268545
PATJ	-0.264577078	2.187536221	-4.22809491	6.44E-05	0.000719409	1.153638359
GOLPH3L	0.229961226	4.941956626	4.227752333	6.45E-05	0.000719763	0.794055355
S100A16	0.211063839	7.758351563	4.226540672	6.47E-05	0.000722375	0.790370098
ARRB2	0.265571456	2.66961478	4.225010201	6.51E-05	0.000725828	1.073208783
GBP3	-0.210589536	4.838555984	-4.224690735	6.52E-05	0.000726126	0.804631004
KIAA0907	0.327069854	3.340576907	4.223470499	6.55E-05	0.000728783	0.914443688
ATP13A3	-0.616385583	6.929385497	-4.222879444	6.56E-05	0.000729795	0.76411693
CPEB2	0.460602533	3.635671408	4.222387636	6.57E-05	0.000730548	0.899705158
C4orf36	-0.322703509	1.87916097	-4.221565462	6.59E-05	0.000731633	1.216651553
ASMTL	0.194251839	5.051194808	4.221616317	6.59E-05	0.000731633	0.773656401
APTX	0.134847634	4.408293267	4.221129784	6.60E-05	0.000732241	0.820607811
MAFB	-0.234293291	6.369397895	-4.22046442	6.62E-05	0.000733456	0.730321591
USP4	0.101898382	6.044328543	4.220145862	6.62E-05	0.000733757	0.731385571
USP22	-0.304340575	7.189989486	-4.219811963	6.63E-05	0.000734099	0.741361915
MSI2	-0.237859637	3.492143305	-4.219036124	6.65E-05	0.00073561	0.907978764
TMEM63A	-0.335160115	4.472597967	-4.216970217	6.70E-05	0.000740009	0.815447319
USP7	-0.155628702	6.724009608	-4.21701506	6.70E-05	0.000740009	0.72134922
GPKOW	0.223311237	4.351074194	4.2160181	6.72E-05	0.000742003	0.811594313
TMEM184C	-0.155441579	5.540114535	-4.214904241	6.75E-05	0.000744435	0.726971756
WDR36	-0.351134244	4.808245767	-4.214026073	6.77E-05	0.000746242	0.747045771
CNTLN	-0.215318973	4.042753594	-4.212984605	6.80E-05	0.000748494	0.873929286
TACC1	-0.372694245	6.364950534	-4.211247484	6.84E-05	0.000752631	0.698852837
RTCB	0.099347268	6.441486695	4.210089062	6.87E-05	0.000755218	0.694174317
B3GALNT1	-0.307849982	3.925116582	-4.208733716	6.90E-05	0.000758351	0.816076821
ANO7	0.459458596	0.410193168	4.207419327	6.93E-05	0.000760495	1.30568013
SUDS3	-0.162845882	4.456171382	-4.207413681	6.93E-05	0.000760495	0.769455205
SRSF8	-0.133582417	5.147589372	-4.20733908	6.94E-05	0.000760495	0.721419058
LINC01465	-0.549074142	-0.174656792	-4.206556631	6.96E-05	0.00076208	1.448800946
USP37	-0.573921795	1.859431593	-4.20624842	6.96E-05	0.00076237	1.054660223
ISOC1	0.199167252	2.825597499	4.205988934	6.97E-05	0.000762526	0.943068951
HIC2	0.271987176	1.901026669	4.204256033	7.01E-05	0.000766729	1.095671982
NAV1	-0.323008539	7.893032257	-4.204039446	7.02E-05	0.000766769	0.718024078
RRAS2	-0.213507333	5.5924253	-4.203457059	7.03E-05	0.000767817	0.680804387
RNF150	-0.496937004	3.555162537	-4.203154268	7.04E-05	0.000768095	0.852121798
EPS8	-0.335248298	6.85565671	-4.201527962	7.08E-05	0.000772033	0.671900251
SERAC1	-0.262483234	3.915414965	-4.199359487	7.14E-05	0.000777501	0.79834356
EIF5A2	-0.274930474	3.784739633	-4.198724342	7.15E-05	0.000778711	0.855907939
PARVB	0.169541819	5.590101887	4.197746463	7.18E-05	0.000780884	0.6667361
LPIN3	-0.275488399	2.856857359	-4.194248425	7.27E-05	0.000790171	0.956613728
JPH2	-0.710053648	0.812016049	-4.191233413	7.35E-05	0.000797694	1.264041727
TMEM199	0.275401354	2.366875285	4.191202845	7.35E-05	0.000797694	1.017106507
EVI2B	0.475900507	2.727352227	4.188859167	7.41E-05	0.000803289	0.976963042
SPG11	-0.327649364	5.688497729	-4.188850638	7.41E-05	0.000803289	0.636256992
LONRF1	-0.282123258	3.465584677	-4.188412556	7.42E-05	0.000803395	0.802652691
THADA	-0.163834147	4.921707282	-4.18843313	7.42E-05	0.000803395	0.675672268
TSR2	0.184118032	4.700500937	4.185372435	7.51E-05	0.000811609	0.671063935
PTCH2	0.663584213	-0.8311147	4.184006959	7.54E-05	0.000815002	1.411028404
STAM	0.129860121	5.356189712	4.182410321	7.59E-05	0.000819087	0.623548333
CRTC3	-0.279636801	5.740173738	-4.182069781	7.59E-05	0.000819498	0.604912039
SSX2IP	-0.343919014	3.971005894	-4.18185383	7.60E-05	0.000819545	0.728683934
PDGFRA	-0.382583646	8.246157833	-4.180749632	7.63E-05	0.000822202	0.648408802
MIR214	-0.300962543	2.86164075	-4.179526173	7.66E-05	0.00082522	0.862807587

Gene	Log fold change	Average Expression	t	P-value	Adjusted P-value	B
EGLN3	-0.567861145	-0.054739553	-4.177484045	7.72E-05	0.000830678	1.307908258
TKT	0.229822889	9.503230614	4.176953384	7.73E-05	0.000831663	0.703049535
ZDHHC16	0.185393323	4.988515016	4.174695474	7.80E-05	0.000837809	0.624064985
NUFIP2	-0.349033256	6.694748287	-4.173871651	7.82E-05	0.000839682	0.572027084
NATD1	0.181826695	5.373809546	4.172863134	7.85E-05	0.000842115	0.585376189
THAP12	-0.216949788	4.413666259	-4.170385331	7.92E-05	0.000849004	0.633922296
RBM3	0.184086073	7.082893927	4.170025087	7.93E-05	0.000849493	0.574540727
PCCA	-0.220334227	3.473871277	-4.169155347	7.95E-05	0.000851532	0.775788344
FDFT1	0.208857534	7.369933844	4.167306165	8.01E-05	0.000856569	0.569905715
MPI	0.121942103	4.663375839	4.166114186	8.04E-05	0.000859614	0.612822824
SLC26A6	0.385246634	2.03765608	4.162947194	8.13E-05	0.000868776	0.904998604
CAMK1	0.214739519	2.214314331	4.162356852	8.15E-05	0.000869992	0.913976142
CALM2	0.131644438	9.28489651	4.160388286	8.21E-05	0.000875508	0.638729962
ATP5A1	0.092168026	8.194112673	4.159424205	8.23E-05	0.000877284	0.575614123
DICER1	-0.350644417	4.679057723	-4.159461581	8.23E-05	0.000877284	0.563024701
PLEKHH3	-0.291382598	3.977505756	-4.159154552	8.24E-05	0.000877508	0.66146244
HUS1	-0.164584334	3.25033555	-4.15863632	8.26E-05	0.000878512	0.738260825
TET3	-0.501826382	3.302868369	-4.15839588	8.27E-05	0.000878645	0.715135107
ACSL4	-0.418200533	6.580916194	-4.157324974	8.30E-05	0.000881388	0.517262289
STX6	0.166617553	5.570593247	4.156765872	8.31E-05	0.000882525	0.523313037
WDR46	0.139979182	5.745658002	4.154335517	8.39E-05	0.000889584	0.508684204
IFT20	0.254733569	4.223395662	4.153578934	8.41E-05	0.00089136	0.615797909
MCHR1	0.623008332	-0.870021042	4.152250644	8.45E-05	0.000894962	1.22654419
CCNK	0.188425312	3.887381912	4.151518055	8.47E-05	0.000896673	0.627217277
DDX21	-0.536623703	6.723208104	-4.150269962	8.51E-05	0.000900039	0.497597043
SMAD6	-0.297038702	4.686621818	-4.14976105	8.52E-05	0.000901041	0.550772384
UBXN2A	-0.151860699	4.673106186	-4.148821658	8.55E-05	0.000903429	0.549902706
LOC100129940	-0.30352821	2.07477141	-4.146141278	8.63E-05	0.000911461	0.896701401
INPP4A	-0.229090518	4.234555589	-4.144626306	8.68E-05	0.000915753	0.58915925
CNTN3	-0.379417922	4.929539113	-4.14271809	8.74E-05	0.000921353	0.509372809
NOTCH4	0.623461525	-1.092941624	4.141500901	8.78E-05	0.000924708	1.282790241
TK2	-0.123400785	5.490420324	-4.139324261	8.85E-05	0.000931252	0.47216351
TTLL7	-0.4461056	1.52854077	-4.137822811	8.89E-05	0.000935588	0.968205819
MRPS30	-0.132491083	4.733217967	-4.136914844	8.92E-05	0.000937961	0.497525172
VPS26A	-0.16714873	6.522092107	-4.136650726	8.93E-05	0.000938189	0.443527011
MAX	0.094417517	5.949285316	4.13593748	8.95E-05	0.000939918	0.443380942
EHMT1	0.153368862	5.1063869	4.135683868	8.96E-05	0.000940111	0.467657684
EFTUD2	0.115166554	6.235507254	4.134019167	9.01E-05	0.000945038	0.433541781
NSD3	-0.19512595	5.634840997	-4.132083525	9.08E-05	0.000950905	0.440887961
BDKRB2	-0.608009977	6.242183572	-4.13082318	9.12E-05	0.000954513	0.426638197
HTRA2	0.187710614	4.250571187	4.130274625	9.14E-05	0.000955713	0.529805487
WASF3	-0.222451829	5.681985992	-4.128420328	9.20E-05	0.000961366	0.424269674
CST3	0.248832169	8.492572375	4.127127169	9.24E-05	0.000965126	0.474718256
TTC9C	0.172556133	3.837304174	4.126912644	9.25E-05	0.000965193	0.521452643
KCND2	-0.318461291	4.199278463	-4.125151186	9.30E-05	0.000970581	0.514331885
MGC20647	1.351307311	-4.286925105	4.120313384	9.47E-05	0.000986721	1.130956536
DYRK1A	-0.169737031	5.425501902	-4.11922082	9.50E-05	0.000989871	0.409994796
KTI12	-0.197038871	3.77299	-4.118720783	9.52E-05	0.000990945	0.555730363
PKNOX1	-0.18062198	4.683564458	-4.118184076	9.54E-05	0.000992149	0.436124364
AMER1	-0.454817528	3.437917893	-4.117427255	9.56E-05	0.000994132	0.527992962
ZSCAN26	0.19518166	3.204013778	4.116785917	9.58E-05	0.000995024	0.560534114
LETM1	-0.193685321	5.232678098	-4.116922768	9.58E-05	0.000995024	0.398082897
GLIS3	-0.38331663	4.7081096	-4.114579121	9.66E-05	0.001002157	0.436508217
FAM58A	0.25673054	3.579667113	4.112681034	9.72E-05	0.001008234	0.528015285
ZNF587	-0.41854212	1.906493437	-4.11210094	9.74E-05	0.001009616	0.774130614
LDAH	-0.185653467	3.536278178	-4.110555839	9.80E-05	0.00101377	0.580251599
RHBDD1	-0.129706002	5.118278764	-4.110683674	9.79E-05	0.00101377	0.402161886
YAP1	-0.235885816	7.323748076	-4.110181138	9.81E-05	0.001013851	0.37154969
DDX23	0.120015388	6.095195324	4.110146096	9.81E-05	0.001013851	0.354100966
ZNF529	-0.270573407	3.107817609	-4.109591634	9.83E-05	0.00101515	0.577227657
BZW2	0.1700316	5.677736543	4.108446125	9.87E-05	0.001018584	0.354351817
CCDC117	0.189117298	4.966505348	4.107829833	9.89E-05	0.001020113	0.372240189
HHAT	-0.378404751	1.88068685	-4.106687892	9.93E-05	0.001022851	0.799960283
HM13	0.152011798	8.152803381	4.10670943	9.93E-05	0.001022851	0.391434687
ADAT2	-0.290620123	1.243818607	-4.105944513	9.96E-05	0.001024849	0.795117107
HECA	-0.17768067	4.928659174	-4.104403127	0.000100146	0.001029759	0.390679064
PRDM10	-0.37211618	3.067861748	-4.103943988	0.000100309	0.001030732	0.536201859
TMEM87B	-0.397283488	5.275172518	-4.103312643	0.000100534	0.001032336	0.349165972
SGPP1	-0.184129196	4.699774945	-4.102705013	0.00010075	0.001033632	0.382059486
PURB	-0.269657149	5.964151163	-4.102573917	0.000100797	0.001033632	0.334460386

Gene	Log fold change	Average Expression	t	P-value	Adjusted P-value	B
PRSS36	0.648581501	-0.975931398	4.101406145	0.000101215	0.001037211	1.149386438
SSH1	-0.307200311	6.283163648	-4.099477038	0.000101909	0.001043612	0.317459287
ZNF441	0.251071582	3.029300148	4.098549633	0.000102244	0.001046333	0.503630318
C15orf52	-0.306932977	2.85873684	-4.098306503	0.000102332	0.001046523	0.605256961
DDIT4L	-0.502526876	3.144948644	-4.09617785	0.000103106	0.001053724	0.576785639
FAM118B	0.178156964	4.662245137	4.095607245	0.000103315	0.001055139	0.366602396
SH3GLB1	-0.201312051	7.133444197	-4.093855649	0.000103957	0.00106098	0.310645611
SLC25A1	0.234790497	6.55098851	4.093469935	0.000104099	0.00106171	0.295999493
ZCCHC2	-0.400888357	3.576307931	-4.093033399	0.00010426	0.001062632	0.446465696
CLTA	0.179141756	7.68904408	4.092701853	0.000104382	0.00106316	0.322564686
PXK	-0.193527923	6.37636074	-4.089184093	0.000105689	0.001075744	0.28272182
KNSTRN	-0.217865874	2.798134175	-4.088135707	0.000106082	0.001078282	0.547668764
VEZT	-0.158870832	5.377023165	-4.088282318	0.000106027	0.001078282	0.303776498
CHN2	1.096690428	-1.07700627	4.086636638	0.000106645	0.001083281	1.112121683
MAPK1IP1L	-0.15113128	6.291143158	-4.085602697	0.000107036	0.001086515	0.269574741
GTPBP3	0.257491648	1.816883155	4.084863105	0.000107316	0.00108798	0.689662353
HIST1H2AC	0.197407792	4.384570022	4.084840449	0.000107325	0.00108798	0.366000209
KIAA0513	0.263303438	4.716627643	4.083768731	0.000107732	0.001091374	0.310291624
MED7	-0.167390561	2.881418051	-4.082516139	0.00010821	0.001095478	0.531771702
KLF12	-0.281546278	4.517058216	-4.081491928	0.000108602	0.001098711	0.337950737
SNX7	0.131889954	5.566779264	4.080249179	0.00010908	0.001102804	0.266007436
RFWD3	-0.341610789	4.23672806	-4.080038849	0.000109161	0.001102884	0.336245693
BRIP1	-0.472725711	1.236700707	-4.077479546	0.000110152	0.00111215	0.775135671
BRINP1	0.421519902	4.296092071	4.075104619	0.000111079	0.001120763	0.290813431
C5orf51	-0.218786596	5.562360364	-4.074408573	0.000111353	0.001122767	0.248973284
BACH2	-0.608859765	0.656526184	-4.073759303	0.000111608	0.00112459	0.82446029
MARS2	-0.320105838	1.563310003	-4.073281364	0.000111796	0.001125736	0.757916594
RBM19	0.133524069	4.752206789	4.071224002	0.000112611	0.001133179	0.274747408
GTF3C4	-0.466351985	5.514072909	-4.06759515	0.000114061	0.00114701	0.222754314
POLI	-0.222647441	3.201498718	-4.067299984	0.00011418	0.001147438	0.449455708
BARD1	-0.389420995	1.54624861	-4.06679339	0.000114384	0.001148723	0.712409081
C2orf81	0.282560836	2.728406403	4.065260225	0.000115004	0.001154179	0.480938602
ZNF408	0.242558179	3.824678985	4.064380023	0.000115362	0.00115626	0.357621489
AMOT	-0.474123099	4.257087503	-4.064371393	0.000115365	0.00115626	0.31964625
ANKRD40	-0.28203313	5.223962171	-4.0623805	0.000116177	0.001163626	0.222734704
WRAP73	0.285317674	2.941920392	4.060843033	0.000116808	0.001169169	0.492380276
RAB23	-0.366245195	5.431830417	-4.059287579	0.00011745	0.0011717	0.202108689
SLC25A24	-0.293963868	5.419290366	-4.059288482	0.00011745	0.0011717	0.196831411
NELFCD	0.1189472	5.617576864	4.059308118	0.000117442	0.0011717	0.192107791
FNBP1	-0.138476197	6.103338647	-4.059301681	0.000117444	0.0011717	0.183648831
RNF4	-0.272680645	5.786585465	-4.059356527	0.000117421	0.0011717	0.183278356
PRDX1	0.183256607	7.345007249	4.058520088	0.000117768	0.001174094	0.195065546
LRCH4	0.246841767	1.902587643	4.05628114	0.0001187	0.001182604	0.561905242
CDH10	0.827747161	-0.097625409	4.05607904	0.000118785	0.001182663	0.925829047
GEM	0.330878446	5.441893037	4.055050802	0.000119215	0.001186168	0.184573372
ZKSCAN7	0.34533483	1.131765305	4.05456656	0.000119419	0.001187407	0.640806125
PSMA7	0.231056464	6.828141815	4.054316526	0.000119524	0.001187669	0.167457317
RASD2	0.521246708	0.130717568	4.053665652	0.000119798	0.001189286	0.79188306
CCDC171	-0.363525055	1.434248042	-4.053368101	0.000119923	0.001189286	0.742239791
CNOT10	0.137270792	4.394555074	4.053439336	0.000119893	0.001189286	0.258669161
RBFOX2	-0.323868261	7.25392317	-4.053094154	0.000120039	0.00118965	0.173304462
POLG	0.173770482	4.915980749	4.051897478	0.000120546	0.001193885	0.192645006
MAP4K3	-0.292473539	4.112261692	-4.051343036	0.000120781	0.00119543	0.269654584
MEX3A	-0.453446216	1.797947981	-4.047836072	0.00012228	0.001209473	0.564282339
BCAR3	-0.261702904	4.476338419	-4.047221741	0.000122545	0.001211293	0.257492311
SCAPER	-0.296066015	2.305336864	-4.04572344	0.000123192	0.001216892	0.548233883
KDM2A	-0.249470149	6.559740558	-4.04545965	0.000123306	0.001217223	0.135023823
ZFP91	-0.264325064	6.879737992	-4.044768733	0.000123606	0.001219384	0.137988945
AKAP2	-0.728360786	1.82086826	-4.043931944	0.00012397	0.001222175	0.588221214
NPR3	-0.509450067	4.586719636	-4.043156614	0.000124308	0.001224709	0.207481607
NFATC1	0.226194402	3.954438508	4.042228628	0.000124714	0.001227342	0.24680648
HK2	-0.331647595	5.150617754	-4.0421733	0.000124739	0.001227342	0.156057734
RIOK1	-0.202351605	4.498889724	-4.041499568	0.000125034	0.001229448	0.197153487
MARK4	0.173740757	5.94190108	4.041036674	0.000125238	0.001230646	0.124453434
PP7080	-0.217543297	1.888064634	-4.040348358	0.000125541	0.001232821	0.541220034
CEP112	-0.158782007	3.406949595	-4.039825791	0.000125772	0.001234282	0.320575872
PACSIN2	0.1396578	5.329703887	4.038625719	0.000126303	0.001237883	0.129758054
TSC22D1	-0.17850086	6.756704903	-4.0387479	0.000126249	0.001237883	0.114922344
MYRF	0.507107137	-0.700689792	4.038107667	0.000126533	0.001238545	0.906271389
WNT5A	-0.396906694	5.248233809	-4.038036612	0.000126564	0.001238545	0.146847705

Gene	Log fold change	Average Expression	t	P-value	Adjusted P-value	B
ALYREF	0.258702038	5.078105877	4.037918091	0.000126617	0.001238545	0.139853166
BHLHE41	-0.333590844	3.210328112	-4.036257825	0.000127357	0.001244978	0.332577347
SELENOS	-0.210000935	5.3078796	-4.034476855	0.000128156	0.001251974	0.12413213
WDR17	-0.578296409	-0.360056314	-4.034207895	0.000128277	0.001252345	0.855858335
ARVCF	0.275220622	2.722160254	4.033474595	0.000128608	0.001254759	0.380067794
ING4	0.217103874	4.103207248	4.032542439	0.000129029	0.001258055	0.237705183
RPP25L	0.236577505	3.253551134	4.03094686	0.000129754	0.001264132	0.309404202
PARP9	-0.165317073	5.648832961	-4.030800469	0.00012982	0.001264132	0.100401524
UBE2M	0.203772383	5.036285761	4.030507504	0.000129954	0.001264614	0.128470641
ARF1	0.114705923	8.449831954	4.029593365	0.000130371	0.001267857	0.152274874
EMC6	0.446227304	0.197222865	4.028973038	0.000130655	0.001269341	0.809329853
C17orf62	-0.106402948	5.773855368	-4.028892036	0.000130692	0.001269341	0.091855848
ZNF620	-0.48673803	-0.148168452	-4.028114354	0.000131049	0.001271988	0.830584068
IDH3B	0.178030702	5.510376877	4.027540272	0.000131313	0.00127373	0.090176759
LOC101929709	-0.395656837	0.384363742	-4.026769157	0.000131669	0.001276357	0.790613889
IFFO1	0.229838582	5.764899492	4.02550233	0.000132255	0.001281214	0.075385344
PHLPP1	-0.371904059	3.907200325	-4.023909028	0.000132996	0.001286859	0.203038539
FZD1	-0.233756301	5.858148964	-4.023881573	0.000133008	0.001286859	0.070986225
AMOTL1	-0.454305515	7.254369008	-4.021993244	0.000133892	0.001294572	0.075473947
FAM180A	-0.282060739	5.276057543	-4.019873043	0.00013489	0.001302861	0.093180058
EIF4EBP2	-0.195926963	7.275569194	-4.019805364	0.000134922	0.001302861	0.0611612
PCGF5	-0.289602188	5.881539381	-4.019524909	0.000135054	0.001303306	0.051446946
GNL2	-0.231158423	6.088498473	-4.018842435	0.000135378	0.001305589	0.045549939
LEO1	0.173584107	4.861074304	4.017424018	0.000136052	0.00131117	0.082341786
IDUA	0.230280707	5.290937739	4.017258987	0.000136131	0.00131117	0.067482397
RNASET2	0.250164823	3.498672886	4.016640055	0.000136426	0.001313175	0.224795595
LHFPL2	-0.403519822	7.028739195	-4.014466435	0.000137468	0.001322361	0.041734959
LOC100129534	-0.422460975	0.265730459	-4.012398844	0.000138466	0.001331115	0.763959517
C1orf159	0.283712704	2.219729501	4.011935544	0.000138691	0.001332425	0.408862892
DIS3L2	0.216100393	3.207069131	4.011621801	0.000138843	0.001333038	0.286536542
SNAP23	-0.313701735	4.706166411	-4.010729463	0.000139278	0.001336355	0.081608873
LIMK2	0.300579454	2.994397414	4.009219285	0.000140015	0.001342578	0.234057616
CCSER2	-0.198401508	5.722479411	-4.008827324	0.000140207	0.001343565	0.020818772
EVI2A	0.485747889	3.246375771	4.008504907	0.000140366	0.001344226	0.2806361
B3GALT2	-1.031877624	0.308947518	-4.005809445	0.000141695	0.001355233	0.791768636
RIOX1	-0.187498098	3.384309151	-4.00589941	0.00014165	0.001355233	0.20311643
MED19	0.210630172	3.80663775	4.004360952	0.000142414	0.001360172	0.149042068
CDCA7L	-0.296849954	4.266778088	-4.004224375	0.000142482	0.001360172	0.098303762
ELF1	-0.378800827	5.817395193	-4.004553667	0.000142318	0.001360172	-0.00068861
ERMARD	-0.194503488	2.952360528	-4.003313379	0.000142937	0.001362096	0.276072995
KRAS	-0.313057367	4.937454237	-4.003244073	0.000142971	0.001362096	0.034350989
H2AFY	0.170464167	6.224304542	4.003313304	0.000142937	0.001362096	-0.006789728
PI4KB	0.111341151	6.217793221	4.003095528	0.000143045	0.001362096	-0.007485649
RORA	-0.335136528	3.82302801	-4.000039478	0.000144581	0.001375849	0.159570474
RPS6KA4	0.149615769	6.213833542	3.998514526	0.000145353	0.001382324	-0.023004329
ALS2	-0.261637383	4.187937874	-3.997614322	0.000145811	0.001385802	0.071730785
LYPLAL1	-0.24311261	3.268407022	-3.996289124	0.000146487	0.001389609	0.22718903
INHBA	0.642678526	5.264476187	3.996582279	0.000146337	0.001389609	0.017673183
CABIN1	-0.129647028	6.393439527	-3.996287212	0.000146488	0.001389609	-0.030847541
ADRB2	1.158526225	-2.860654557	3.995110871	0.000147091	0.001392704	0.857813512
C19orf57	-0.618972729	-0.799973599	-3.995109382	0.000147092	0.001392704	0.83552203
SLC38A9	0.238542775	2.826221988	3.99535293	0.000146967	0.001392704	0.255770331
NEURL1B	0.429787942	0.991437861	3.993147222	0.000148103	0.001401394	0.484032326
QTRT2	-0.269002196	3.472561385	-3.992652455	0.000148359	0.001402935	0.117429508
BRF2	0.188177547	2.232055947	3.992361033	0.00014851	0.001403481	0.298270057
NUP37	0.18748306	3.386314104	3.991671174	0.000148868	0.001405982	0.171250213
ANAPC15	0.249236389	2.063633528	3.991475382	0.000148969	0.001406061	0.364577536
SPG7	0.153991724	5.457389482	3.990910761	0.000149263	0.001407952	-0.02869383
ZBED4	-0.293506365	4.285177119	-3.990355185	0.000149553	0.0014098	0.046153453
SLC15A3	0.241570216	4.151121681	3.986757654	0.000151441	0.001426708	0.051597131
DTX3L	-0.220945305	5.692946277	-3.986069244	0.000151805	0.001429243	-0.055077046
HMGB3	0.160521885	3.956111096	3.985520397	0.000152096	0.001431086	0.04894101
ZNF33A	-0.195368894	3.864810401	-3.984105791	0.000152848	0.001437263	0.06107044
ATP6V0D1	0.140473539	7.173848232	3.983402516	0.000153223	0.001439891	-0.060725526
RP2	-0.319961141	3.949836283	-3.983028498	0.000153422	0.001440871	0.074415527
KRT17	0.97676472	-1.999051811	3.980980173	0.000154521	0.001450287	0.809890454
SSBP4	0.277773396	5.868167414	3.980675966	0.000154685	0.001450921	-0.074101388
DEAF1	0.19315595	4.982343353	3.979955319	0.000155074	0.001453663	-0.042399988
SRRM2	-0.28804769	8.730959797	-3.97968511	0.00015522	0.00145395	-0.004517972
CCT8	0.117911628	6.961784686	3.979541673	0.000155298	0.00145395	-0.078871328

Gene	Log fold change	Average Expression	t	P-value	Adjusted P-value	B
SLFN11	-0.383960176	5.64041666	-3.978299978	0.000155971	0.001459345	-0.079142105
ZNF837	0.357302328	0.855533237	3.976726533	0.000156828	0.001466452	0.483425404
SLC20A2	-0.283713324	5.443241045	-3.975119934	0.000157707	0.001473763	-0.097384207
ST3GAL4	0.214663407	4.456448977	3.972477877	0.000159164	0.001486455	-0.022930742
SNAI2	-0.282083824	6.800166099	-3.971350005	0.00015979	0.001491376	-0.114013645
DDX3X	-0.36808195	7.822588676	-3.971084189	0.000159938	0.001491831	-0.077735831
RCSD1	1.197382767	-3.41937982	3.970181235	0.000160441	0.001495599	0.775504967
RAI1	-0.253539279	5.205463477	-3.969463076	0.000160843	0.001498413	-0.102855264
SPRY4	-0.350393756	3.754660943	-3.96846734	0.000161401	0.001502261	0.154583981
COQ7	-0.161355054	3.161389543	-3.968370192	0.000161455	0.001502261	0.120518721
YIPF4	-0.144042695	5.556530055	-3.963919839	0.000163973	0.001524744	-0.12145826
SAE1	0.109567699	6.802638803	3.963557582	0.000164179	0.001525723	-0.135197892
HSPB1	0.330485688	7.853606312	3.962916413	0.000164546	0.001528184	-0.107365194
BRMS1	0.247337595	5.447152675	3.962450021	0.000164812	0.00152972	-0.12487981
G6PD	0.217012981	7.225634283	3.959958577	0.000166245	0.001542068	-0.138665452
PTGER2	-0.380506492	2.853236957	-3.958545417	0.000167063	0.00154775	0.139628825
CCNB1	-0.352121242	3.902917573	-3.958651793	0.000167002	0.00154775	0.009127526
ARL13B	-0.43189288	3.334324178	-3.955177414	0.000169028	0.001564993	0.032889912
PARD3	-0.154226248	5.38636833	-3.9540606	0.000169685	0.001570107	-0.146974187
ZFP90	0.235085104	4.836887464	3.952719217	0.000170477	0.001576465	-0.136293018
DUSP7	-0.33292354	4.922825719	-3.952350993	0.000170695	0.001577512	-0.116623671
CAND2	0.332339829	1.874595508	3.949274197	0.000172526	0.001592486	0.266041865
RAB5C	0.166844175	5.724682126	3.94941973	0.000172439	0.001592486	-0.177536308
RAB27B	-0.543269167	2.60546856	-3.947814899	0.000173402	0.001599413	0.203183731
PUSL1	0.237746236	3.472066732	3.947669432	0.000173489	0.001599413	-0.017336799
TMED6	-0.766499974	-1.342795931	-3.946918206	0.000173942	0.001600646	0.678051117
SLC4A4	-0.410105077	0.476354238	-3.947169985	0.00017379	0.001600646	0.418355506
UCK1	0.166836049	4.799372464	3.946994799	0.000173896	0.001600646	-0.134796281
GNB2	0.204149514	7.466947938	3.946206058	0.000174372	0.001603624	-0.17915009
DCK	-0.277018919	3.502135041	-3.944439906	0.000175443	0.0016116	-0.015122404
PINK1	0.154090276	4.875322563	3.944422693	0.000175453	0.0016116	-0.154591109
RDH11	0.119870063	6.061976038	3.944083355	0.000175659	0.001612513	-0.2029293
ARPC2	0.110081759	8.254087487	3.943406527	0.000176072	0.001615316	-0.147269888
TMEM65	-0.238567366	5.479330044	-3.943056211	0.000176286	0.001616294	-0.18588898
DEPDC1	-0.514566933	1.154009176	-3.942447344	0.000176658	0.001618722	0.397213492
USF1	0.211140167	4.0235056	3.9420292	0.000176914	0.001619099	-0.121011846
AP2A1	0.122631505	7.937477936	3.942200863	0.000176809	0.001619099	-0.171004985
OMG	0.523973865	2.248261576	3.939778184	0.000178299	0.001629794	0.208195432
FRG1	0.184945154	3.95938266	3.939914771	0.000178215	0.001629794	-0.096563801
GPATCH3	0.168646021	3.628358312	3.939203127	0.000178655	0.001632053	-0.044271356
MANEA	-0.293549321	4.050298154	-3.938069892	0.000179357	0.001636652	-0.1032433
MAP4K5	-0.183413967	5.920165537	-3.938040632	0.000179375	0.001636652	-0.219429497
PSMA3	0.219525234	5.652823717	3.937497097	0.000179713	0.001638742	-0.21778403
PTPRE	-0.605998136	0.691084505	-3.936871819	0.000180103	0.001641301	0.36364523
XPOT	-0.17032831	7.676060488	-3.936350758	0.000180428	0.00164327	-0.201016156
TBC1D13	0.121788968	5.61479244	3.935621522	0.000180884	0.001646428	-0.221209481
TIGD3	0.964667549	-2.532390607	3.933832483	0.000182008	0.001655654	0.670606488
MAP7D1	-0.147325795	7.48370726	-3.933075486	0.000182485	0.001658995	-0.216023212
FIG4	-0.146959354	4.803186196	-3.932671982	0.00018274	0.001660311	-0.171776434
SLC35F6	-0.117582985	6.200907328	-3.931795638	0.000183295	0.001664349	-0.242054271
BEND7	-0.200971285	2.675097587	-3.930221992	0.000184296	0.001672426	0.028050072
TSHZ2	-0.292070106	5.995178989	-3.929643021	0.000184665	0.00167477	-0.24585574
ATP6V0A1	-0.171156477	5.56764939	-3.9283601	0.000185486	0.001681205	-0.244139419
ST6GALNAC4	0.191662962	5.547283024	3.927859636	0.000185807	0.001683104	-0.24371478
TMEM19	-0.188925936	4.472669593	-3.927176251	0.000186247	0.001686072	-0.177015318
POLD3	-0.211956416	3.910892564	-3.926479307	0.000186696	0.001689124	-0.127939396
VRK3	0.163952196	4.243580353	3.926025585	0.000186989	0.00169076	-0.167304171
NOC4L	0.272522973	3.848303524	3.925088288	0.000187596	0.001694213	-0.118431848
BMI1	-0.121032783	5.077482244	-3.925092451	0.000187593	0.001694213	-0.224238984
NPC1	-0.337493092	6.177670535	-3.923653496	0.000188529	0.001701615	-0.271191875
MTCL1	0.212939524	6.909153688	3.922959345	0.000188981	0.00170468	-0.264302406
GATAD2A	-0.154262941	6.496354754	-3.920946663	0.0001903	0.001715547	-0.280443338
SPATA2L	-0.27131194	2.970331552	-3.920665579	0.000190485	0.001716186	0.027426734
HIST1H2BK	0.245726008	3.126630661	3.918138072	0.000192155	0.001730197	-0.051415409
NSUN2	-0.191413002	5.378357571	-3.912212292	0.000196125	0.001764893	-0.282618651
APOBEC3F	0.333821098	1.16355274	3.910851806	0.000197048	0.001772137	0.272031967
CYSRT1	0.804547826	-1.547473644	3.909763602	0.000197789	0.001777739	0.574555113
SNAPC4	-0.154743213	3.720390271	-3.909431144	0.000198016	0.001778717	-0.197784605
BMP2K	-0.299288219	4.45940182	-3.908855445	0.000198409	0.00178119	-0.256457075
ZNF330	-0.151809137	4.792549649	-3.908439383	0.000198694	0.001782685	-0.258349622

Gene	Log fold change	Average Expression	t	P-value	Adjusted P-value	B
CCDC96	-0.335139955	0.65396698	-3.908235828	0.000198834	0.001782875	0.258186158
RNF219	-0.305295381	3.222851008	-3.906429914	0.000200075	0.001792942	-0.126591156
FDXACB1	-0.421885085	-0.094022186	-3.906235793	0.000200209	0.001793075	0.41965287
TEP1	-0.386337528	3.967411334	-3.904406131	0.000201476	0.001803346	-0.194234895
DDX54	0.121522259	5.817737169	3.903382329	0.000202188	0.001808644	-0.334094469
GFOD2	-0.145115929	4.193608722	-3.90297849	0.000202469	0.001810088	-0.247985366
CERCAM	0.146726491	8.292245114	3.90279765	0.000202595	0.001810141	-0.282264332
HIPK3	-0.376576325	5.994863136	-3.901812848	0.000203284	0.001815216	-0.338632131
NUP50	-0.269371536	4.812006591	-3.901089715	0.000203791	0.001818665	-0.308311392
PLK1	-0.427411966	2.51621726	-3.898862871	0.00020536	0.001830599	0.010061047
RRBP1	0.120389523	9.518901448	3.898846622	0.000205371	0.001830599	-0.217607431
EIF1AD	-0.179947635	4.363042681	-3.898514235	0.000205606	0.001830632	-0.263478515
NUP62	-0.123872615	5.626037825	-3.898497951	0.000205618	0.001830632	-0.347842004
PPP1R15A	0.318063772	6.371601883	3.898296322	0.000205761	0.001830821	-0.352571116
RAP2C-AS1	-0.485685202	0.629915254	-3.896096941	0.000207325	0.001842558	0.305310202
BTBD7	-0.28515942	5.414905789	-3.896254828	0.000207212	0.001842558	-0.346610958
RBM43	0.231112813	3.923322896	3.894093655	0.000208759	0.001854211	-0.245137014
FAM196A	0.466537045	0.187746065	3.893333858	0.000209305	0.001857969	0.252053137
EXOC3L2	1.202069088	-3.257144852	3.892038312	0.00021024	0.001864706	0.541453207
CLK2	0.352480304	3.206244649	3.891939319	0.000210312	0.001864706	-0.19732238
MECP2	-0.159765122	6.027324893	-3.891536363	0.000210603	0.001866194	-0.375143949
MDH2	0.118077217	7.233953266	3.890068578	0.000211669	0.001874536	-0.368023959
CEP162	-0.171266075	3.327955042	-3.889702013	0.000211936	0.001874696	-0.126866638
CAPN1	0.13724271	7.724351795	3.889857876	0.000211823	0.001874696	-0.352517552
EMD	0.213495048	5.839487486	3.88923213	0.000212279	0.001876625	-0.377385689
NR1D1	0.230653056	4.789334349	3.8884811	0.000212828	0.001879293	-0.308233081
NDN	0.142632646	6.536982252	3.8884778	0.00021283	0.001879293	-0.387011296
MAPRE2	-0.146307639	5.053295491	-3.887244932	0.000213734	0.001886169	-0.34570501
SGIP1	-0.274973881	3.047557208	-3.886975366	0.000213932	0.001886812	-0.132769649
EIF5B	0.156991452	7.530388384	3.886017224	0.000214638	0.001889717	-0.365386669
SRP54	0.131606739	5.446264757	3.886155106	0.000214536	0.001889717	-0.382909239
NAPA	0.144289861	6.13400156	3.886168083	0.000214527	0.001889717	-0.393396437
PSMD14	0.172848402	5.741505264	3.88453677	0.000215733	0.001898245	-0.393270215
PPP2R1A	0.132402254	8.146183873	3.883167466	0.00021675	0.001906083	-0.351770627
METTL12	0.271996388	1.93214468	3.882424333	0.000217304	0.00190984	0.017379421
SLAIN2	-0.201028701	5.938467515	-3.88140272	0.000218068	0.001915436	-0.402619276
MTMR10	-0.292043427	3.931062264	-3.881162097	0.000218248	0.001915902	-0.27947361
SRA1	0.25974531	5.006115942	3.880355549	0.000218853	0.001920097	-0.372457331
FBXO30	-0.232648638	4.981149681	-3.880084936	0.000219057	0.001920764	-0.359485769
NUF2	-0.530033795	0.918441433	-3.878502629	0.00022025	0.001929085	0.236700892
HSF2	-0.213056522	3.404001352	-3.878394588	0.000220332	0.001929085	-0.232677735
HMGCL	0.200585399	4.744770578	3.878317647	0.00022039	0.001929085	-0.346731984
EXOSC7	0.240261297	4.079633125	3.877778582	0.000220798	0.001931536	-0.30352772
PTMA	0.169082881	7.049989946	3.876843006	0.000221508	0.001936624	-0.416770755
LINC01021	0.514444694	-0.101640471	3.876196818	0.000222	0.001939648	0.309358252
APPL2	-0.13276855	6.662963494	-3.876050321	0.000222112	0.001939648	-0.42686014
GTSE1	-0.415923757	1.76841565	-3.875234157	0.000222735	0.001943961	0.036552593
ARL6IP6	-0.341404967	2.614454875	-3.87445983	0.000223327	0.001948004	-0.078110372
ZRSR2	0.291037332	2.507361967	3.873876286	0.000223775	0.00194965	-0.11288351
CERK	-0.124596055	6.108360938	-3.873977556	0.000223697	0.00194965	-0.432543243
COX8A	0.258043518	5.208632621	3.870882214	0.000226085	0.001968636	-0.409376687
AGTPBP1	-0.202381526	3.385715422	-3.869860286	0.000226878	0.001974405	-0.237966559
RBM42	0.251975379	6.129198654	3.866833536	0.000229244	0.001993844	-0.455975346
CHRNB1	0.333847356	1.177703682	3.865625153	0.000230196	0.002000962	0.080799804
RCOR2	-0.278791404	1.695706982	-3.863960347	0.000231512	0.002011247	-0.105053401
RGS17	-0.332594395	2.180347559	-3.863782942	0.000231653	0.00201131	-0.106009535
TRPS1	-0.343819053	5.598755458	-3.86133481	0.000233604	0.002027076	-0.465555003
PARD3B	-0.199710718	5.301825729	-3.86115991	0.000233743	0.002027123	-0.447047766
MAGEH1	0.129981104	4.888739397	3.860258736	0.000234466	0.002032217	-0.436121501
YIF1A	0.214581401	5.928268544	3.859161005	0.000235349	0.002038696	-0.476978201
DAAM2	-0.312291664	5.300265095	-3.856801463	0.000237257	0.002054045	-0.464183005
PAXIP1-AS1	-0.341625559	0.844356857	-3.856317827	0.00023765	0.002055851	0.152557456
PDXDC1	-0.166215822	6.10472065	-3.856208798	0.000237739	0.002055851	-0.492402088
C16orf52	-0.256220164	3.663838903	-3.854944327	0.000238769	0.002063273	-0.277533704
TSPYL1	-0.148220013	6.045568774	-3.854820203	0.000238871	0.002063273	-0.494545077
TBL1XR1	-0.18478432	6.279531309	-3.854213214	0.000239367	0.002066376	-0.498374675
PSMD12	-0.161700704	5.718504508	-3.852134075	0.000241075	0.002079931	-0.493150858
FBXL3	-0.263468606	5.471833524	-3.851650246	0.000241475	0.002080991	-0.488070157
TMEM214	0.104248501	7.131425908	3.851683224	0.000241447	0.002080991	-0.49554639
NCKAP5L	-0.158096796	5.510310694	-3.850498233	0.000242427	0.002088009	-0.489127973

Gene	Log fold change	Average Expression	t	P-value	Adjusted P-value	B
PITPNM2	0.221387382	3.715021363	3.849824526	0.000242986	0.002090756	-0.371811581
MAPKAP1	-0.091294851	6.846731728	-3.849779256	0.000243024	0.002090756	-0.511333638
NPLOC4	-0.156996989	6.750527381	-3.849102714	0.000243587	0.002094401	-0.513668431
PRMT1	0.191182548	6.212401438	3.848889318	0.000243764	0.002094733	-0.515828883
TRUB1	0.189278151	4.069405647	3.848576963	0.000244025	0.002095775	-0.399447858
TPI1	0.199649102	7.643812051	3.848154434	0.000244377	0.002097608	-0.491339737
LYPLA2	0.173982858	4.116710247	3.846293307	0.000245937	0.002109788	-0.422512581
PEPD	0.127779396	6.964684197	3.844654133	0.000247318	0.002120428	-0.524004911
BCAS2	0.173426647	4.616384963	3.843906541	0.00024795	0.00212464	-0.455271269
CIART	-0.397220269	2.017818515	-3.842610659	0.00024905	0.002129805	-0.010460563
TBC1D1	0.135113828	4.709707742	3.842778861	0.000248907	0.002129805	-0.474552729
DDAH1	-0.180154547	7.17706387	-3.843010268	0.00024871	0.002129805	-0.506273841
CYC1	0.245376895	5.616999325	3.842530054	0.000249118	0.002129805	-0.523034461
FOXO3	-0.28889425	5.416132466	-3.841446586	0.000250041	0.002136486	-0.521210895
ARF4	0.117748057	8.532967593	3.839898728	0.000251366	0.002144156	-0.462925285
FBXO31	-0.158245389	5.648502226	-3.840021721	0.000251261	0.002144156	-0.542954298
CDK16	0.09885665	6.650804306	3.840104236	0.00025119	0.002144156	-0.54416502
WDR59	0.163518739	3.992057207	3.837666852	0.000253288	0.002159328	-0.411035192
RPL13	0.226670315	9.914367865	3.837435333	0.000253488	0.002159812	-0.404496378
ALDOA	0.214545279	10.01998868	3.836562102	0.000254245	0.002165032	-0.397831647
RLIM	-0.289666087	5.657162129	-3.835308008	0.000255335	0.002173085	-0.549453615
EEFSEC	0.225302983	3.977332831	3.833835341	0.00025662	0.002182794	-0.420275762
ATIC	0.132198491	6.460109309	3.832252865	0.000258009	0.002193366	-0.570567738
CBFB	-0.151942365	5.350373981	-3.8313656	0.00025879	0.002198769	-0.553348721
DPM2	0.217789458	4.71933195	3.830786661	0.000259302	0.002201871	-0.511069658
FAM35A	-0.433434744	3.064103521	-3.827562004	0.000262167	0.002224947	-0.32648052
FLYWCH1	0.229140523	5.64940981	3.82533881	0.00026416	0.002240598	-0.575110575
EFCAB11	-0.216387431	2.258504788	-3.824379863	0.000265024	0.002246662	-0.188701444
ZNF562	-0.236803479	3.552224616	-3.822843389	0.000266414	0.002257177	-0.414229116
HARS2	0.137206572	3.968278409	3.819772089	0.000269214	0.002279615	-0.508015541
NRROS	-0.336867109	1.904221204	-3.819517847	0.000269447	0.002280306	-0.16241335
EML4	-0.343476974	5.369241108	-3.818686878	0.00027021	0.002285479	-0.596458841
CC2D2A	-0.185746853	4.384234363	-3.817670478	0.000271146	0.002292109	-0.505878815
RBM4	-0.272495055	1.632967201	-3.816996273	0.000271768	0.002296083	-0.108705417
NR3C1	-0.253272342	7.480725544	-3.816752435	0.000271994	0.0022967	-0.600053979
ZNF865	0.197909973	4.565038375	3.816254925	0.000272454	0.002299301	-0.542811844
GREB1	0.69928915	-1.432071639	3.815614496	0.000273048	0.002303025	0.265370802
FOXP2	-0.26100972	3.408115283	-3.815263939	0.000273374	0.002304481	-0.41097904
ITPK1	0.153164266	6.733183776	3.81477812	0.000273826	0.002307001	-0.624226696
SLC7A6OS	0.239038964	1.925921067	3.812772904	0.000275699	0.002320187	-0.203564892
LPAR1	-0.162666883	7.542792162	-3.812841457	0.000275635	0.002320187	-0.614975899
MAP3K6	0.145037745	5.655311299	3.812585606	0.000275875	0.002320369	-0.62147928
LINC01204	-0.599925932	-1.006833618	-3.812108294	0.000276323	0.002321862	0.212799348
PRX	0.158225973	3.633013993	3.812067971	0.000276361	0.002321862	-0.469568616
BBS2	-0.12576122	4.842411954	-3.811385783	0.000277002	0.002325955	-0.577679598
TMPO	-0.207143361	5.357886982	-3.811137412	0.000277236	0.002326622	-0.610664074
PRLR	-0.711705395	3.142975278	-3.810503901	0.000277834	0.002330338	-0.351940748
AASDH	0.298238536	2.623290751	3.808881944	0.000279369	0.002341912	-0.351654813
KLHL35	0.531998302	-0.067311872	3.808347662	0.000279877	0.002344862	0.15167836
SPSB1	-0.361945292	5.790773619	-3.807077798	0.000281087	0.002353689	-0.642427821
ATP5D	0.297590325	6.010490349	3.806303397	0.000281827	0.002358577	-0.64893306
CAP1	0.108798907	8.094123786	3.805441189	0.000282653	0.002364179	-0.599823858
SFPQ	-0.194860047	7.289650633	-3.804998448	0.000283078	0.002366422	-0.638472399
PDLIM1	0.176790863	5.130980746	3.804461175	0.000283595	0.002369428	-0.624932043
RAP2A	-0.198700358	5.311452829	-3.804152134	0.000283893	0.0023706	-0.642645406
INSIG2	-0.193534495	4.163325817	-3.802783814	0.000285215	0.002380318	-0.54306292
CFLAR	-0.167109439	5.308854358	-3.802317631	0.000285666	0.002381448	-0.638695727
DEGS1	0.107433535	6.850436318	3.802371846	0.000285614	0.002381448	-0.660267809
FBXO44	0.252182701	3.787852458	3.801142897	0.000286807	0.002389639	-0.49659824
C19orf12	-0.124811365	4.902248112	-3.800397558	0.000287534	0.002394366	-0.619100943
HEIH	0.246023088	2.813316265	3.798738484	0.000289157	0.00240655	-0.412461356
SNRNP70	0.213743604	6.764295481	3.798159672	0.000289725	0.002409948	-0.677306508
CLINT1	-0.221631723	6.366305812	-3.797651651	0.000290225	0.002412772	-0.681392425
BMP1	0.115564404	6.919760689	3.797259887	0.00029061	0.002414647	-0.676572472
KCNJ5	1.125781874	-3.12281035	3.796585459	0.000291276	0.002418842	0.257830101
TP63	-1.080343716	-2.035905522	-3.794916933	0.000292928	0.002429667	0.253472792
SLFN13	-0.531141467	-0.467487594	-3.794781154	0.000293063	0.002429667	0.117699271
GSKIP	-0.150556002	4.25765988	-3.794858219	0.000292987	0.002429667	-0.599040947
IMPACT	-0.192015277	5.050298605	-3.794443016	0.000293399	0.002431115	-0.655239168
PGS1	-0.182747721	3.820448266	-3.794023195	0.000293817	0.002433239	-0.527665292

Gene	Log fold change	Average Expression	t	P-value	Adjusted P-value	B
PEAK1	-0.34531341	7.467487483	-3.793393687	0.000294445	0.002437097	-0.673675042
CCNA2	-0.360040325	2.790799771	-3.792766883	0.000295071	0.002440938	-0.407830275
PLCD4	-0.268616815	1.632737545	-3.792549419	0.000295288	0.002441397	-0.213962882
RNF213	-0.318622921	7.191184432	-3.791787323	0.000296052	0.002446368	-0.692059651
NSDHL	0.199520442	5.653501009	3.791404475	0.000296436	0.002448029	-0.698602835
PPP2CA	-0.119991079	6.51490808	-3.791263357	0.000296578	0.002448029	-0.702882964
ASB13	-0.210024114	3.853794379	-3.790396707	0.00029745	0.002453883	-0.594406883
TMEM184A	1.144383873	-3.323615353	3.788629447	0.000299236	0.002465916	0.226974882
SLC6A8	0.139272461	5.39886838	3.788631686	0.000299234	0.002465916	-0.692556626
HCFC1	-0.24390405	6.343263795	-3.787365781	0.00030052	0.002475138	-0.71540582
FAM172A	-0.159052376	4.930541493	-3.786800722	0.000301095	0.002477169	-0.67266955
MAP1LC3B	0.105742284	7.202556774	3.786837027	0.000301058	0.002477169	-0.702230589
LIAS	0.2294496	2.711540426	3.786146273	0.000301763	0.002481309	-0.397166878
DPP3	0.12042709	4.7221837	3.785760055	0.000302158	0.002481846	-0.664287478
RARS	0.116359013	6.557508502	3.785817826	0.000302099	0.002481846	-0.717487511
KANK1	-0.269568776	4.342509423	-3.78442724	0.000303524	0.00249171	-0.615288614
ZNF697	-0.422398096	3.847131254	-3.78394698	0.000304018	0.002494404	-0.570759966
PTPN9	-0.186547995	5.668537586	-3.78356047	0.000304416	0.00249631	-0.718245419
MYL12B	0.172344694	7.758310137	3.783279083	0.000304706	0.002497328	-0.693625633
GNL1	0.113772943	6.700336968	3.7827211	0.000305282	0.002500688	-0.72824621
LRRC57	0.137604718	3.473660738	3.782288147	0.000305729	0.002501774	-0.5444022
AP1AR	-0.202311147	3.820026589	-3.782111031	0.000305913	0.002501774	-0.584924043
RSBN1	-0.35273966	3.909299609	-3.782208237	0.000305812	0.002501774	-0.614180431
ISCU	0.175630201	6.726221609	3.780519833	0.000307564	0.002513913	-0.735803408
ANKRD50	-0.443085048	5.388952753	-3.780057875	0.000308045	0.002516479	-0.702485413
SRSF4	-0.140643079	6.023767113	-3.778588197	0.00030958	0.002527648	-0.74236472
UGP2	-0.124424982	6.961433714	-3.778261095	0.000309922	0.002529074	-0.740239484
SLC46A3	-0.227044656	3.536748483	-3.77801511	0.00031018	0.002529808	-0.536058046
P4HB	0.097508953	10.00204979	3.777475524	0.000310747	0.002533057	-0.586369859
STUB1	0.203785976	5.108505273	3.776907214	0.000311344	0.002536556	-0.710316268
SHROOM2	-1.102188995	-1.250475304	-3.775075369	0.000313278	0.002550932	0.167345393
DDX59	0.200230745	3.174670206	3.77340717	0.000315049	0.002563586	-0.46334392
RPP30	0.179278101	4.297869736	3.773291391	0.000315173	0.002563586	-0.654513376
RXFP1	-0.692988646	0.42300431	-3.771641017	0.000316935	0.002576529	0.014413362
KNL1	-0.772719129	1.70345132	-3.770612537	0.000318038	0.002581316	-0.244429178
SCN1B	0.246163879	3.511155587	3.770662084	0.000317985	0.002581316	-0.585817032
MGAT4B	0.113278239	7.631135498	3.770828882	0.000317806	0.002581316	-0.73994454
SELENOK	0.220502829	4.23003612	3.770230582	0.000318448	0.002583256	-0.675884056
OAT	0.119733322	6.839568747	3.769878668	0.000318827	0.002584936	-0.76380938
PRMT6	-0.118059369	4.726519232	-3.769388318	0.000319355	0.002587827	-0.717391531
PPP2R5B	0.178902365	4.13071913	3.765827292	0.000323218	0.002617018	-0.669807099
MSX1	-0.408040921	5.581025563	-3.765747225	0.000323305	0.002617018	-0.76417284
HAUS8	-0.279633008	1.762659267	-3.765141666	0.000323967	0.002620963	-0.286355637
FAM19A3	0.698185771	-1.262328266	3.763033349	0.000326279	0.002638258	0.055726494
CLOCK	-0.331713112	4.919103238	-3.761338415	0.00032815	0.002651961	-0.750850455
EXO1	-0.428551552	1.611178508	-3.761145915	0.000328363	0.00265226	-0.302882768
APOBEC3C	0.176173149	6.219214999	3.760613227	0.000328954	0.002655606	-0.800890507
KIAA0232	-0.475247214	5.419633019	-3.759471918	0.000330222	0.002664417	-0.783469524
ZNF524	0.265378586	4.272103714	3.756759391	0.000333256	0.002686616	-0.700922668
SEPT7	0.085148832	7.540776204	3.756692962	0.00033333	0.002686616	-0.787541156
TRPC6	-1.112437414	-0.993977741	-3.75599412	0.000334116	0.002690466	0.080244455
AKAP8L	-0.212538999	4.601314544	-3.755950755	0.000334165	0.002690466	-0.748923976
MAGED2	0.126680417	7.384309912	3.755744639	0.000334397	0.002690897	-0.797700466
PER3	-0.227131584	3.841174059	-3.753665662	0.000336748	0.002708365	-0.685587914
SMYD4	0.184114044	2.515640738	3.753048517	0.000337448	0.002712553	-0.512664162
ZNF551	-0.251713357	2.388069497	-3.752321125	0.000338276	0.002717084	-0.45206194
FBRS	0.119392283	5.970577001	3.752236292	0.000338373	0.002717084	-0.82123193
LBH	-0.284960031	6.052272862	-3.750630358	0.000340208	0.002730362	-0.826185401
GNA11	0.099631905	6.391579946	3.747510281	0.0003438	0.002757721	-0.84316442
THBD	-0.424663391	5.224645924	-3.746404936	0.000345081	0.002764829	-0.816793717
ARHGAP12	-0.232572648	5.939040469	-3.746271023	0.000345237	0.002764829	-0.839456276
CLEC11A	0.261981122	6.093782873	3.746306195	0.000345196	0.002764829	-0.843044646
LRRC14	-0.172238934	4.100163217	-3.745535195	0.000346092	0.00277021	-0.750598321
SGO2	-0.314589393	2.750130244	-3.743258399	0.000348753	0.002790025	-0.514629002
LRCH1	-0.182093647	4.130630219	-3.741220635	0.000351151	0.002807717	-0.766258771
E2F8	-0.712349701	-0.715361117	-3.740587396	0.000351899	0.002812209	-0.027700675
FAM129C	-0.90564232	-2.767849929	-3.739927671	0.000352681	0.002816958	0.091068226
FBRSL1	0.158076165	4.821798694	3.738920245	0.000353877	0.002825015	-0.81407058
FBXL15	0.310076456	2.576941669	3.738414096	0.000354479	0.002825966	-0.515513309
C6orf47	-0.134048291	4.195859606	-3.738347397	0.000354559	0.002825966	-0.783674593

Gene	Log fold change	Average Expression	t	P-value	Adjusted P-value	B
FASTKD2	-0.171397837	4.684228162	-3.73852965	0.000354342	0.002825966	-0.81802926
PFKP	0.151991082	5.715942624	3.737629264	0.000355416	0.002831296	-0.865928715
BCLAF1	-0.135895263	6.371033063	-3.73645118	0.000356825	0.002841023	-0.878564174
GBP5	-0.656592968	-0.822413087	-3.736058071	0.000357297	0.002843275	-0.000116979
FBXL8	0.301181037	1.469256932	3.735787121	0.000357622	0.002844361	-0.387885253
IL6R	-0.374286203	3.176895308	-3.735540898	0.000357918	0.002845213	-0.659125581
ANKRD10	-0.196152696	4.017134949	-3.735000011	0.000358569	0.002848883	-0.765292626
C22orf29	-0.205763879	4.402367796	-3.734566048	0.000359092	0.002851535	-0.777073079
DTX4	-0.457700758	1.888107559	-3.731816925	0.000362422	0.002876464	-0.472905592
KIDINS220	-0.266483936	6.593338291	-3.731410753	0.000362917	0.002878872	-0.893584301
KIAA1841	-0.214725713	2.714012615	-3.730884565	0.000363558	0.002882443	-0.578551479
B3GNT5	-0.529235598	1.986153596	-3.728845275	0.000366055	0.002900712	-0.481822339
NXPH4	0.278009275	2.88423744	3.726397025	0.000369074	0.002923099	-0.613307683
TIFA	-0.19810656	2.547158037	-3.72534553	0.000370378	0.002931885	-0.613155346
NRG2	0.962125112	-2.442306532	3.724070799	0.000371965	0.002941668	0.029457563
IQCH-AS1	-0.25196494	2.40671137	-3.724038868	0.000372005	0.002941668	-0.524176704
IQGAP3	-0.469027574	2.62611247	-3.723611669	0.000372538	0.002943514	-0.519599626
WDR37	-0.309358916	4.431369237	-3.723538869	0.000372629	0.002943514	-0.839075268
ZNF778	-0.325288982	1.995276026	-3.723050701	0.000373239	0.00294679	-0.503362093
LRRFIP1	-0.137132156	6.573507517	-3.722791288	0.000373564	0.002947809	-0.91733916
XIAP	-0.460738901	4.653415011	-3.721954373	0.000374613	0.002954543	-0.854836285
SOCS3	0.572158086	5.479201848	3.719913902	0.000377184	0.00297326	-0.894914184
PPP1R18	-0.149709996	7.447912329	-3.719353067	0.000377893	0.002977295	-0.917963361
CXorf23	-0.226547928	2.716494753	-3.719042707	0.000378287	0.002977367	-0.634207874
DDX42	-0.090223733	6.90525154	-3.718948118	0.000378407	0.002977367	-0.927966405
ZMAT2	0.10466844	6.306969379	3.718877882	0.000378496	0.002977367	-0.934211675
KRIT1	-0.183228008	4.207953914	-3.718369406	0.000379141	0.002980887	-0.843942699
ADIPOR2	-0.237134137	6.067103656	-3.71809724	0.000379487	0.002982049	-0.935938696
B4GAT1	0.144433999	5.168786311	3.717824103	0.000379834	0.002982569	-0.904315959
SEC61B	0.224292894	6.098193787	3.717733953	0.000379949	0.002982569	-0.936281079
TRIB2	-0.303612806	5.096596276	-3.717140772	0.000380705	0.002986945	-0.881072853
CBX2	-0.445644361	0.174233429	-3.716910788	0.000380998	0.00298769	-0.301059555
CPZ	0.338474137	1.136887272	3.715562406	0.000382722	0.002995091	-0.377072775
FUT11	-0.179391465	4.945652816	-3.715395848	0.000382936	0.002995091	-0.909696062
SLC9A9	-0.235137753	5.559864873	-3.715641635	0.000382621	0.002995091	-0.921646431
EEF1D	0.189782834	6.371070631	3.715875741	0.000382321	0.002995091	-0.943829314
DNAJC3	-0.285970976	6.369051041	-3.715530789	0.000382763	0.002995091	-0.94489811
HPRT1	0.191759371	4.2926419	3.714072516	0.000384636	0.00300683	-0.855120344
SPIN1	-0.27701917	6.468882843	-3.713728816	0.000385079	0.00300873	-0.950028302
CCDC102A	0.249848147	3.690003772	3.713268432	0.000385673	0.003010248	-0.774552077
TMEM185B	-0.224715303	4.666702005	-3.713276725	0.000385662	0.003010248	-0.885999953
MAP3K7	-0.159049918	6.010043757	-3.71105097	0.000388546	0.0030311	-0.957981609
SLC25A22	-0.188756897	4.694653487	-3.710762173	0.000388922	0.003032461	-0.913973669
PPP3CB	-0.121442985	6.748162968	-3.710405604	0.000389386	0.00303451	-0.960158636
RABGAP1L	-0.109330154	4.500055607	-3.709034654	0.000391176	0.003046884	-0.888181323
NFIL3	0.22532998	5.801989246	3.708834479	0.000391438	0.003047349	-0.947510269
ISYNA1	0.235300431	3.69154865	3.707501907	0.000393186	0.003058309	-0.79282745
HIC1	-0.238915314	5.6249989	-3.707452255	0.000393252	0.003058309	-0.959356244
CTU2	0.219557861	3.590548954	3.706923798	0.000393947	0.003062137	-0.797749308
PAPD7	-0.292795179	4.09578995	-3.706193423	0.000394911	0.003068042	-0.871751446
SDF4	0.161468583	8.067884065	3.706037398	0.000395117	0.00306806	-0.927729734
RSRC1	-0.15634751	4.755138161	-3.705769869	0.00039547	0.003069224	-0.903561035
NUP188	0.151446913	6.441077637	3.705604231	0.000395689	0.003069343	-0.975158877
GMFB	-0.310883056	6.314656246	-3.702159216	0.000400272	0.003103292	-0.987324036
RABGGTB	0.165858461	4.330407765	3.701125576	0.000401657	0.003112426	-0.897011226
AMIGO2	-0.351703704	2.876548319	-3.700552932	0.000402426	0.003115181	-0.640165383
VDAC1	0.122049401	6.921008979	3.700574346	0.000402397	0.003115181	-0.986621781
MIER1	-0.134058292	5.456200614	-3.700394026	0.000402639	0.003115234	-0.970810984
KATNAL2	-0.265020709	1.690975473	-3.700197576	0.000402904	0.003115679	-0.474699176
ID3	0.439205211	5.613528326	3.69995365	0.000403232	0.003116619	-0.937045102
RTL9	-0.821434362	-2.170446348	-3.699411503	0.000403963	0.003120666	-0.030950009
DUS2	0.185286289	2.788009318	3.699194514	0.000404256	0.003121328	-0.725973769
OPA1	-0.183929966	5.872616139	-3.697851781	0.000406073	0.00313375	-0.996544691
EBF2	-0.557208919	2.106258751	-3.697158437	0.000407014	0.003139405	-0.558431658
TES	-0.280102298	3.133756485	-3.69685097	0.000407432	0.003141021	-0.776819512
CHST14	0.127100269	5.909258369	3.696143765	0.000408395	0.003146835	-1.000332046
CEP290	-0.227453758	3.462879633	-3.695298266	0.000409549	0.003154116	-0.782667189
DDI2	-0.279302417	4.570503237	-3.69462509	0.000410471	0.003159595	-0.965229034
NLN	-0.184392089	5.134065621	-3.693934562	0.000411418	0.003165268	-0.993940164
GFER	0.22136091	3.237679104	3.693269514	0.000412332	0.003170681	-0.786929056

Gene	Log fold change	Average Expression	t	P-value	Adjusted P-value	B
IGFL3	1.135429945	-3.69679767	3.691186613	0.000415207	0.003190704	-0.054210116
OTUD4	-0.303504611	4.878128453	-3.691076701	0.000415359	0.003190704	-0.986502257
NUS1	-0.192124528	4.200855689	-3.690605619	0.000416012	0.003194092	-0.924748104
GPR3	-0.553054597	0.166128515	-3.688659697	0.000418721	0.003213249	-0.185699897
ACAP2	-0.228945648	5.786501239	-3.688182815	0.000419387	0.003216724	-1.025274536
NPM1	0.166977172	8.004633134	3.688001121	0.000419641	0.003217035	-0.987224137
TTI1	0.160123284	4.141844402	3.687793214	0.000419932	0.003217628	-0.930275226
JUNB	0.477062461	6.337617529	3.687427848	0.000420444	0.003219912	-1.023945256
ARSJ	-0.309328992	4.000690519	-3.687250398	0.000420693	0.00322018	-0.881188742
C12orf49	-0.234533042	5.960099709	-3.686911559	0.000421168	0.003222182	-1.033159267
PSMC6	0.116661781	4.762919141	3.686534649	0.000421698	0.003224595	-0.984753209
ING2	-0.246269044	3.252382284	-3.682976988	0.000426727	0.003261395	-0.80969494
GMPS	-0.113134296	6.378701753	-3.682616383	0.000427239	0.003263659	-1.049290969
FBXL19	0.155529228	3.903546725	3.682012112	0.0004281	0.003268577	-0.951845056
EBNA1BP2	0.17133429	5.23148561	3.681739821	0.000428489	0.003268633	-1.030654766
ATP2B1	-0.470583944	6.843180908	-3.681702771	0.000428542	0.003268633	-1.035328703
DST	-0.309305242	7.942679674	-3.681525733	0.000428794	0.003268906	-1.011935954
FAM84B	1.18949019	-1.886005954	3.681196257	0.000429265	0.003270839	-0.095583611
GPI	0.130133618	7.898670936	3.680348305	0.000430479	0.003278001	-1.016884805
DCAF10	-0.164196799	5.411794681	-3.680235879	0.00043064	0.003278001	-1.03684451
PSMB1	0.200982596	6.665475839	3.680056284	0.000430898	0.003278305	-1.055848511
DUT	0.160593107	5.443439514	3.679844041	0.000431202	0.003278966	-1.03852881
KNOP1	-0.163169134	3.037104652	-3.679400039	0.00043184	0.00328216	-0.815097006
CYP4V2	-0.156628701	4.624791229	-3.678961595	0.000432471	0.003285297	-0.991429282
DUSP1	0.495950415	5.323536731	3.678580479	0.00043302	0.00328781	-1.004506054
RWDD2B	0.208034123	4.479696959	3.676204089	0.000436459	0.003312248	-0.972911981
COX10-AS1	-0.350318099	0.229094861	-3.675450563	0.000437554	0.00331329	-0.323559636
SLC39A3	0.228790373	4.243547751	3.675660472	0.000437249	0.00331329	-0.966822085
PCBP4	0.16736458	5.192729423	3.675613604	0.000437317	0.00331329	-1.037774269
TRIM8	-0.197978128	7.375398702	-3.675411234	0.000437612	0.00331329	-1.066052129
PSME1	0.181351513	6.693123923	3.675353549	0.000437696	0.00331329	-1.071136659
FAM109A	-0.155694629	4.534413758	-3.672750738	0.000441502	0.003340423	-0.997773304
VTI1A	-0.142726053	4.404171187	-3.672342011	0.000442102	0.003343288	-0.990666877
TPM3	0.100966617	7.567959467	3.671029079	0.000444037	0.003356231	-1.056616077
ZNF566	-0.297737402	2.91586506	-3.670659136	0.000444583	0.003357126	-0.859849651
TCEAL3	0.243026609	4.179444809	3.670592569	0.000444682	0.003357126	-0.977426212
LRRC58	-0.312098748	6.185858313	-3.670496419	0.000444824	0.003357126	-1.087079888
NPHP1	-0.215809105	2.216933845	-3.670008326	0.000445546	0.003360894	-0.612598479
LINC01686	0.725558251	-2.033962841	3.66874932	0.000447415	0.00337197	-0.135472527
PPM1F	-0.127083751	5.294571103	-3.668717449	0.000447462	0.00337197	-1.052044488
CRKL	-0.221746814	6.175590327	-3.668561144	0.000447694	0.003372035	-1.093263599
SART1	0.208137018	6.448224005	3.668077704	0.000448414	0.003375769	-1.095077092
NR2F6	0.199810467	4.563286301	3.667918595	0.000448652	0.003375867	-1.024977003
MB21D1	-0.308332481	1.417670612	-3.667503387	0.000449271	0.003378841	-0.60170999
UBA3	-0.110169496	5.229684686	-3.66548746	0.00045229	0.00339985	-1.071016138
ZNF239	-0.286199332	1.382257286	-3.664727139	0.000453434	0.003406662	-0.556413793
ARHGAP1	0.122620951	7.889616429	3.664584617	0.000453649	0.003406662	-1.065108165
YY1	-0.106471593	6.925073282	-3.664312797	0.000454058	0.00340804	-1.099020418
PICALM	-0.198204022	7.71003092	-3.663070586	0.000455935	0.003420424	-1.072888255
NDUFS3	0.246351935	5.249450791	3.662767107	0.000456395	0.003422168	-1.079901382
ELOA	-0.195868943	5.499537878	-3.662044658	0.000457491	0.003427047	-1.094045437
CKAP5	0.220581249	6.776980433	3.662038362	0.000457501	0.003427047	-1.109453568
MYADM	-0.220956052	7.413165802	-3.66178879	0.00045788	0.003428183	-1.093333991
APBA3	0.23997385	3.233606158	3.660735381	0.000459484	0.003438483	-0.851467839
SIK3	-0.290527363	4.855741306	-3.660236755	0.000460245	0.003442469	-1.065483025
A4GALT	0.175130985	6.119492771	3.658312881	0.000463193	0.003462798	-1.123552934
BAG4	-0.168681865	3.976315167	-3.658051096	0.000463595	0.003464088	-1.017699548
SYBU	-0.486096518	1.748972468	-3.657773568	0.000464023	0.00346556	-0.501447692
CUL7	-0.134970417	6.204979144	-3.65752182	0.00046441	0.003466737	-1.125895035
ZNF674	-0.34668818	0.878530875	-3.655516238	0.00046751	0.003488149	-0.484746416
GTF2E2	0.183016421	4.906275539	3.655165942	0.000468054	0.003490475	-1.084173991
TRPM7	-0.251089551	5.827134773	-3.654068834	0.000469759	0.003501463	-1.135300387
PODXL	-0.38466531	4.322985651	-3.653509744	0.000470631	0.003506225	-1.059258569
RTN4R	1.032280192	-2.966679715	3.652788787	0.000471757	0.003512878	-0.163351873
CCDC102B	-0.46724654	2.131542411	-3.652520237	0.000472178	0.00351427	-0.669922391
ROCK1	-0.399922865	6.169854438	-3.652234191	0.000472625	0.003515867	-1.144017337
CEP135	-0.330379755	2.370517042	-3.652036154	0.000472936	0.003516439	-0.709482859
EVA1A	-0.311060822	4.040942249	-3.650275062	0.000475704	0.003535278	-0.958618711
HOOK2	-0.188960203	2.827851331	-3.649603896	0.000476763	0.003537913	-0.820201084
RHOBTB2	-0.123714309	4.862865882	-3.649664905	0.000476667	0.003537913	-1.101630077

Gene	Log fold change	Average Expression	t	P-value	Adjusted P-value	B
SCRN1	-0.204290485	7.39064579	-3.64961271	0.000476749	0.003537913	-1.134839944
RPRD2	0.155240358	5.607265704	3.647563728	0.000479997	0.003560152	-1.153841304
ORC1	-0.480636186	0.691455883	-3.646286587	0.000482031	0.003573483	-0.522693047
FEN1	-0.216271267	4.05336547	-3.646098047	0.000482332	0.003573956	-1.052508445
PHF21A	0.18874477	5.12586995	3.645905666	0.00048264	0.003574475	-1.129012101
IFI16	-0.132883536	8.343996348	-3.645520816	0.000483255	0.003577275	-1.109842797
ACSF3	0.239578756	4.044476897	3.644875881	0.000484288	0.003583161	-1.028327491
SOWAHC	-0.260914622	4.613073255	-3.644568234	0.000484782	0.003585051	-1.045099294
TALDO1	0.162892534	6.876372072	3.643227168	0.000486938	0.003599235	-1.168821741
C2CD3	0.2188402	3.703373678	3.641243628	0.000490145	0.003619388	-1.016821983
TRIP4	0.10932352	4.10445108	3.641353633	0.000489967	0.003619388	-1.054153221
SHF	0.291894398	2.182869901	3.640679679	0.000491061	0.003624371	-0.758629036
PSMA6	0.209502666	3.041585888	3.640469404	0.000491403	0.003625117	-0.935841166
RPS6KB2	0.201188593	4.361559701	3.639851155	0.000492409	0.003630761	-1.086344624
FBXO6	0.255848269	2.875606335	3.639412682	0.000493123	0.003634252	-0.881785502
RFFL	-0.204397135	2.141976211	-3.639034185	0.000493741	0.003635248	-0.795299569
HSD17B10	0.207069262	4.829590221	3.639043151	0.000493727	0.003635248	-1.131996417
EPS8L2	0.203157818	5.347554253	3.638709205	0.000494272	0.00363738	-1.154813856
GIMAP2	0.309965916	1.522767657	3.638309502	0.000494926	0.003640413	-0.657795337
ADNP	-0.168912357	6.338062392	-3.637886257	0.000495619	0.003643733	-1.189761409
GCNT1	-0.352827006	4.144926818	-3.637398501	0.000496419	0.003647835	-1.08177589
RAD51B	-0.194942229	2.108739784	-3.636956234	0.000497146	0.003651393	-0.74268413
CEP83	0.243858644	2.592401383	3.636424832	0.00049802	0.003655552	-0.88676221
TNRC6B	0.21662456	5.035963896	3.636317137	0.000498198	0.003655552	-1.163047834
PSPN	0.284622309	1.971980278	3.635651549	0.000499295	0.003661821	-0.741224955
ACER3	-0.224227286	4.240749622	-3.635450918	0.000499626	0.003662467	-1.103729086
GNG5	0.151954441	5.884153976	3.634885696	0.000500561	0.003667532	-1.193553252
RAB36	-0.240295553	2.782679183	-3.634463818	0.000501259	0.00366908	-0.903489911
FAM120B	-0.140667763	5.333834922	-3.634569379	0.000501084	0.00366908	-1.174905974
TRIM47	-0.310860333	4.790896977	-3.630849882	0.000507281	0.003711353	-1.16785393
RPL28	0.272678857	9.710130806	3.629159955	0.00051012	0.003730314	-1.083657173
INIP	-0.221332821	4.594262352	-3.628032507	0.000512023	0.003742411	-1.153872059
HRAS	0.256200082	4.744966714	3.627597406	0.000512759	0.003745973	-1.158681523
STARD3NL	-0.161276383	4.286532781	-3.627318498	0.000513231	0.003747607	-1.11390376
TMC7	0.444835043	-0.248817321	3.626758827	0.00051418	0.003751136	-0.439137652
SOD3	0.327259761	7.19633116	3.626739795	0.000514212	0.003751136	-1.21330372
OXSR1	-0.233140383	5.092676662	-3.626244051	0.000515055	0.003753644	-1.197190908
SEC22C	-0.184892751	5.585333474	-3.626364285	0.00051485	0.003753644	-1.214185526
FTSJ3	0.120106677	5.817856805	3.624572039	0.000517905	0.003772589	-1.223560464
ST6GAL1	0.431724616	0.761398763	3.623935607	0.000518993	0.003778693	-0.588813741
TUBA1B	0.153038209	5.975300953	3.622312043	0.000521781	0.003797152	-1.23673378
TMED9	0.168250638	7.328249426	3.621897768	0.000522494	0.003800508	-1.222372716
PALM2	-0.666502813	0.68488533	-3.62134714	0.000523444	0.003805579	-0.668274545
ATXN7L3B	-0.087247565	6.368388381	-3.620945047	0.000524139	0.003808791	-1.242992828
NXN	0.201177355	6.236071406	3.620752992	0.000524471	0.003809366	-1.241463657
SMNDC1	-0.162572494	4.763821767	-3.620369974	0.000525133	0.003812342	-1.192595548
GYS1	0.102787086	5.504048977	3.620062176	0.000525667	0.003814376	-1.23104178
PROB1	-0.3546952	1.816631984	-3.6197327	0.000526238	0.003816683	-0.76380253
ZNF710-AS1	0.388928254	0.877530417	3.618999854	0.000527511	0.003822799	-0.612199099
C3orf58	-0.213350656	4.484756733	-3.618809301	0.000527843	0.003822799	-1.142614996
OGFRL1	-0.315789197	5.427515963	-3.618842202	0.000527785	0.003822799	-1.2311166
HADHB	0.100363402	7.160219206	3.617908578	0.000529412	0.003832324	-1.241198359
NAMPT	-0.277515083	6.491049589	-3.617739381	0.000529708	0.00383262	-1.249108853
LOC101928053	-0.830274799	-2.330120866	-3.617306369	0.000530464	0.003835482	-0.27002228
NDUFA8	0.160223474	4.460542595	3.617221639	0.000530612	0.003835482	-1.165632226
SLC22A15	-0.31966907	2.009207023	-3.616272254	0.000532275	0.003845655	-0.829452746
ARMCX6	0.13277257	4.075710992	3.615551181	0.000533541	0.003852956	-1.145535359
WDR18	0.249082178	4.785103201	3.613811537	0.000536608	0.003873245	-1.205863271
RTL3	-0.781204239	-1.789153179	-3.612759324	0.000538471	0.00388483	-0.316060842
FAM173A	0.339913324	3.229243435	3.612312192	0.000539264	0.003888692	-1.020907677
CCT3	0.129868158	7.753541949	3.611985897	0.000539844	0.00389101	-1.236810278
PSMC2	0.135068655	6.331468364	3.610589208	0.000542332	0.003907076	-1.275186132
SUMO3	0.117229392	7.072867855	3.610284588	0.000542877	0.003909127	-1.265965353
TAOK1	-0.446900949	6.673080157	-3.609031248	0.000545121	0.003923412	-1.274835766
SLC2A6	0.23341535	4.194463355	3.607131093	0.00054854	0.003946138	-1.204906517
NUMB	0.130634703	6.059045549	3.606346497	0.000549958	0.003954449	-1.286629809
NBPF15	-0.418792586	2.281451441	-3.601782971	0.000558274	0.004012147	-0.933286963
RUFY2	-0.206585869	4.133214402	-3.60158885	0.000558631	0.004012147	-1.188374253
RNF40	0.123630196	6.398580378	3.601506722	0.000558782	0.004012147	-1.303376715
CCDC88A	-0.279918613	5.12992299	-3.601255048	0.000559244	0.004013555	-1.273820442

Gene	Log fold change	Average Expression	t	P-value	Adjusted P-value	B
REPS2	-0.393677713	1.461141749	-3.600512277	0.000560611	0.004020333	-0.754713097
GTDC1	-0.113541289	4.542091356	-3.60030715	0.000560989	0.004020333	-1.221781679
ALG3	0.168377658	5.236578846	3.600374836	0.000560864	0.004020333	-1.280452162
ARHGEF12	-0.354226921	7.37081581	-3.600065102	0.000561436	0.004021619	-1.285645723
ITFG2	0.155515385	3.735882407	3.599306332	0.000562837	0.004029744	-1.170030329
SMAD2	-0.084618734	6.699627375	-3.598868874	0.000563647	0.004033623	-1.309854348
STARD9	0.350183988	2.480107704	3.598676963	0.000564002	0.004034251	-1.027551734
CDK13	-0.188386943	5.468153652	-3.598069563	0.000565129	0.004040392	-1.293062721
GRIN2D	0.237774838	3.994546724	3.597701411	0.000565813	0.004043363	-1.177822687
FAM83D	-0.394912618	1.944522417	-3.596249843	0.000568517	0.004059107	-0.85222087
PSMC3	0.219093714	6.798342736	3.596229616	0.000568555	0.004059107	-1.316297969
P2RX5-TAX1BP3	-0.592830255	-1.042594546	-3.595949368	0.000569079	0.00406092	-0.407181723
PLSCR1	-0.162319868	4.718027814	-3.595726035	0.000569496	0.004061975	-1.263938093
HAS3	0.822358942	-1.67712824	3.595214247	0.000570454	0.004066883	-0.358800991
CYR61	0.942472955	7.172185715	3.594653397	0.000571506	0.004072452	-1.303349462
BNC1	-0.354375054	3.921660165	-3.593964487	0.0005728	0.004079649	-1.162432462
TRAF3	-0.237308947	5.104016223	-3.593827618	0.000573057	0.004079649	-1.292575538
CHPF	0.190988108	8.008471933	3.591154611	0.000578107	0.004113657	-1.286750841
RTTN	0.266274221	2.307343341	3.589284053	0.000581667	0.004137028	-0.914065778
GTF2F1	0.1327895	7.076808228	3.5890066	0.000582196	0.004138725	-1.333692934
NRAS	-0.2034628	5.89588094	-3.588803153	0.000582585	0.004138725	-1.335828368
SH3BP4	-0.207948292	6.751646902	-3.588727567	0.000582729	0.004138725	-1.341408393
MAP1LC3A	0.264161178	4.199291193	3.587163636	0.000585726	0.004158049	-1.228712764
SLC16A7	-0.403324136	5.038564364	-3.586556622	0.000586893	0.004164371	-1.30065722
GNPNAT1	-0.329713082	4.827337306	-3.586138363	0.000587699	0.004168123	-1.304866554
SPOP	0.095932746	5.599192332	3.585359177	0.000589202	0.004176819	-1.341358828
PELP1	0.145796057	5.941454846	3.584855728	0.000590175	0.004179784	-1.351769609
NDUFS1	-0.144695315	6.033543904	-3.584892174	0.000590105	0.004179784	-1.35272357
MICA	0.250643475	4.226614839	3.583746634	0.000592325	0.004193036	-1.264141855
FAM129B	0.128257366	8.731516394	3.580692274	0.000598283	0.004233223	-1.274413124
METTL14	-0.125408229	4.335918817	-3.580474547	0.00059871	0.004234254	-1.27675168
PINX1	0.294350052	2.189753086	3.580067826	0.000599508	0.00423791	-0.985651371
C6orf226	0.28701975	1.967321415	3.579106523	0.000601398	0.00424928	-0.927155812
ARHGAP6	-0.323707942	3.446527653	-3.578930447	0.000601745	0.004249738	-1.156532644
GOPC	-0.185635266	5.188147742	-3.577597665	0.000604377	0.004266325	-1.339628127
ABCC10	0.179767008	4.031343186	3.577071128	0.00060542	0.004271274	-1.244918687
EIF2AK4	-0.136149526	6.467734011	-3.576957504	0.000605645	0.004271274	-1.379525289
BAG6	0.100423729	7.873821161	3.576217805	0.000607114	0.004279626	-1.344612879
AHCTF1	-0.234890088	4.034760832	-3.575062649	0.000609413	0.004293828	-1.271078967
HSF1	0.157058399	6.387354282	3.574457574	0.000610621	0.004300328	-1.38698365
FAM208A	-0.316257888	5.106893592	-3.574265717	0.000611005	0.004301018	-1.343917345
C6orf132	-0.253828103	4.228694502	-3.572519882	0.000614505	0.004323636	-1.271450848
MRPL41	0.292255164	5.039242725	3.571720664	0.000616114	0.004332931	-1.347393869
STRBP	-0.213626988	2.582459723	-3.570694348	0.000618185	0.004343444	-1.017279843
RRP1B	-0.215081368	5.831547713	-3.570753674	0.000618065	0.004343444	-1.395831351
LY6E	0.227674486	8.883324815	3.57036233	0.000618857	0.004344506	-1.310270932
SLC11A2	-0.280985519	5.189735133	-3.570334544	0.000618913	0.004344506	-1.377390236
LIFR	-0.425522034	4.309615615	-3.569931359	0.00061973	0.004348211	-1.311852646
AGL	-0.299930214	4.256305114	-3.568391873	0.000622857	0.004368118	-1.313978515
DHX36	-0.224078477	5.598697765	-3.567366356	0.000624949	0.004380747	-1.400384357
CLP1	0.151764357	3.834240506	3.567098186	0.000625497	0.004382549	-1.266543947
PTGIS	-0.206292186	8.933455483	-3.566781892	0.000626143	0.004383004	-1.31571256
ZNF281	-0.255470495	5.148538314	-3.566811039	0.000626084	0.004383004	-1.359750997
HIVEP3	-0.296309614	3.167710952	-3.564169406	0.000631511	0.004416347	-1.154170454
WAC	-0.136863237	6.66580173	-3.564036188	0.000631786	0.004416347	-1.417085747
DLST	-0.173452096	6.710247905	-3.564075095	0.000631706	0.004416347	-1.417963632
PIGW	-0.225371566	3.010101968	-3.563551473	0.000632787	0.004421294	-1.212934335
OTUD6B	-0.326472993	2.101854584	-3.563168236	0.00063358	0.00442478	-0.986694392
SLC29A3	0.183778318	3.722881265	3.562613278	0.00063473	0.004430753	-1.25334176
NME2	0.235220512	4.256950776	3.562298352	0.000635383	0.004433258	-1.309343595
CNOT9	-0.136766877	5.654598282	-3.560937298	0.000638213	0.004450944	-1.420322661
PTGES2	0.227898723	5.068589329	3.56071506	0.000638676	0.004452113	-1.388990254
ACOT9	-0.100753145	4.744485441	-3.560462234	0.000639204	0.004453729	-1.3804006
STYX	-0.195486062	4.33725848	-3.56012	0.000639918	0.004456646	-1.345629911
VWA1	0.311253113	2.308761165	3.55956029	0.000641088	0.004462734	-1.040842618
FAM199X	-0.330212518	3.660765809	-3.559169416	0.000641907	0.004466367	-1.280895801
LRRK2	-0.306297806	3.697537029	-3.558760065	0.000642765	0.004470274	-1.219639596
ZC3H6	0.252793116	2.303811512	3.557748773	0.00064489	0.004482982	-1.093916588
TMEM232	-0.418336287	-0.073791475	-3.557578385	0.000645249	0.004483407	-0.62304242
MPST	0.222984335	5.59476438	3.557121832	0.000646211	0.004488021	-1.422188821

Gene	Log fold change	Average Expression	t	P-value	Adjusted P-value	B
MBP	-0.297986847	5.392287048	-3.556102972	0.000648362	0.00450089	-1.422621641
DRAP1	0.264731189	6.853170723	3.554331818	0.000652119	0.004524883	-1.44457738
RSPRY1	-0.149455779	5.020277954	-3.552535959	0.000655949	0.004549363	-1.415925786
PHF10	-0.120344615	4.503828054	-3.55108287	0.000659064	0.00456722	-1.373721495
PAK1IP1	-0.165367941	4.538035139	-3.551052008	0.00065913	0.00456722	-1.387095585
KSR1	0.329462835	3.080993261	3.550020815	0.000661349	0.004580489	-1.212079813
MIGA2	0.173561532	3.662711559	3.549285306	0.000662936	0.004586909	-1.297153277
MTMR6	-0.382658863	5.079664089	-3.549352016	0.000662792	0.004586909	-1.430271394
ZDHHC7	-0.138181021	6.726386739	-3.549168022	0.00066319	0.004586909	-1.464314874
DAGLA	-0.22256747	3.055441779	-3.548608805	0.000664399	0.004593166	-1.265582696
ASB16-AS1	0.349105285	1.310781084	3.547148077	0.000667568	0.00461121	-0.874665647
CFAP97	-0.41418697	5.670391886	-3.547123682	0.000667621	0.00461121	-1.463293206
CDC37	0.14314556	6.965361074	3.546917562	0.00066807	0.004612193	-1.46568309
SCRN3	-0.180975017	4.169192113	-3.545385316	0.000671412	0.004633143	-1.361976592
FLRT2	-0.252341396	6.931790448	-3.544742366	0.000672819	0.004640728	-1.471889693
PKM	0.132795564	10.65965816	3.543447974	0.000675661	0.004658193	-1.280402769
SEMA7A	-0.330296619	4.732776758	-3.542367929	0.00067804	0.00467246	-1.450214989
ESCO1	-0.362258392	3.539905333	-3.541859566	0.000679163	0.004678057	-1.314467714
TNFRSF11B	-0.303228949	5.035568748	-3.541365665	0.000680256	0.004683441	-1.423711036
EIF5A	0.209741491	7.168839771	3.541112397	0.000680816	0.004685161	-1.476731951
TMEM121	0.398384923	0.743407651	3.539645787	0.000684073	0.004703275	-0.861300289
MIEF1	-0.210642109	5.728275309	-3.539709209	0.000683932	0.004703275	-1.487379444
TTYH3	0.134353436	8.10045619	3.537389562	0.000689112	0.004734142	-1.446244052
TTC37	-0.228585554	6.32015986	-3.537213972	0.000689505	0.004734142	-1.501287264
MRPL37	0.145048954	6.419107607	3.537325272	0.000689256	0.004734142	-1.501363967
PRR13	0.135656622	4.294836751	3.536668387	0.00069073	0.004738619	-1.399820284
DRG1	0.126161817	5.537865608	3.536643053	0.000690786	0.004738619	-1.489334954
TSPAN5	-0.306499148	6.059763102	-3.535989309	0.000692256	0.004746541	-1.503265074
SIX4	-0.297793636	3.898773199	-3.535709702	0.000692886	0.004748696	-1.385424906
RGS10	0.213689349	5.289423628	3.534023831	0.000696693	0.00477262	-1.483785627
NHLRC3	-0.233699609	3.590802255	-3.532835328	0.00069939	0.004788911	-1.335697858
EMX2OS	-0.196237291	6.808360217	-3.531267641	0.000702961	0.004811179	-1.518214571
POLM	0.173786491	3.084189166	3.53062716	0.000704425	0.004817435	-1.259231072
TMEM200B	-0.145927049	4.009529533	-3.53058803	0.000704515	0.004817435	-1.39223568
ALDH18A1	0.103262633	7.053608694	3.528879216	0.000708436	0.004842047	-1.513803881
FAM76B	-0.282027048	2.251743325	-3.528159505	0.000710093	0.004851175	-1.139877896
ARHGEF1	0.152032665	5.504353187	3.527398028	0.000711851	0.004860979	-1.50853804
HSDL2	0.101533528	5.7195862	3.52703798	0.000712683	0.004863893	-1.522923158
MYBBP1A	0.11421938	6.157068329	3.526934301	0.000712923	0.004863893	-1.533335756
DDX60L	-0.247447499	4.369156137	-3.525648787	0.000715904	0.004880322	-1.449008081
GPR161	-0.216777354	5.056771922	-3.525616472	0.000715979	0.004880322	-1.499996889
A2M-AS1	-0.479140104	-0.473985257	-3.524973343	0.000717475	0.004888307	-0.679172141
CCDC121	0.346283249	0.445252795	3.524618081	0.000718303	0.004891733	-0.838860043
SCN8A	-0.335845542	3.370237799	-3.52424506	0.000719173	0.004895444	-1.353976859
ZNF195	0.161263735	3.50271428	3.523585517	0.000720713	0.004903715	-1.350079843
URGCP	-0.147929351	5.101937245	-3.522660974	0.000722878	0.004916225	-1.508835525
FLII	0.090304606	7.858651408	3.522164343	0.000724044	0.004921929	-1.50888977
C9orf16	0.278165977	5.458669955	3.521901872	0.00072466	0.004923899	-1.524634053
CCDC191	-0.352990526	0.884994294	-3.521173098	0.000726375	0.004933325	-0.903593346
BUB1B	-0.448731916	1.821387364	-3.520872327	0.000727084	0.004935914	-1.077075142
CASP4	-0.136678395	5.730405493	-3.519936904	0.000729293	0.004948678	-1.543411767
RHEB	0.174895815	5.002864234	3.519702413	0.000729847	0.004950212	-1.519903164
TIMM10B	-0.142141379	4.943211744	-3.519481083	0.000730371	0.004951536	-1.519790681
STK36	-0.266413399	3.522254343	-3.518317515	0.000733131	0.004964893	-1.310871507
RUBCNL	-0.303020873	3.343656875	-3.518270739	0.000733243	0.004964893	-1.346988084
PLAGL1	-0.204009271	4.902171002	-3.518233862	0.00073333	0.004964893	-1.507952425
WDR6	-0.150896047	6.794397311	-3.517209809	0.000735768	0.004979161	-1.558595214
LOC105376805	0.959589755	-2.496590708	3.516761497	0.000736838	0.004984162	-0.549989727
NFS1	0.155580947	3.313378176	3.516563869	0.00073731	0.004985116	-1.336836647
POLR2E	0.19480662	7.122543734	3.515949507	0.000738779	0.004992809	-1.558540594
DDB1	0.187761276	8.36451884	3.514966205	0.000741137	0.005006494	-1.498087355
FBXO3	-0.226436447	4.241299343	-3.513625675	0.000744362	0.005026027	-1.467392262
DENND2C	-0.419145376	2.317854819	-3.512611416	0.000746811	0.005040304	-1.170308669
CREB5	-0.360928769	4.303520357	-3.512274262	0.000747627	0.00504355	-1.481996662
ARIH2OS	0.514072473	-1.049531557	3.511327653	0.000749921	0.005056767	-0.693536429
CTNNBL1	0.167412694	5.46878929	3.50904367	0.000755486	0.005092011	-1.568309342
WDR26	-0.134529235	7.247463343	-3.508536522	0.000756727	0.005098094	-1.577969813
PKNOX2	1.059611185	-2.136134217	3.508187699	0.000757582	0.00510157	-0.598286066
NDUFA11	0.263359723	3.691374822	3.505474989	0.000764259	0.005144237	-1.42322792
LOC730101	0.325374703	2.834763664	3.504117804	0.000767621	0.005163623	-1.357863337

Gene	Log fold change	Average Expression	t	P-value	Adjusted P-value	B
WDR82	-0.168204398	7.158953875	-3.504035623	0.000767825	0.005163623	-1.588538533
SP4	-0.376942314	2.365578077	-3.50382521	0.000768348	0.005164831	-1.193478138
STK17A	-0.2095721	5.560302075	-3.503276207	0.000769713	0.005171699	-1.576953611
SPAG5	-0.365922783	2.539868313	-3.502141241	0.000772542	0.005188395	-1.223821955
SMARCD2	0.094231765	5.813709675	3.501040332	0.000775296	0.005204569	-1.60521859
VWA8	-0.197360665	4.720390691	-3.500751925	0.000776019	0.005207102	-1.555418826
COPS2	0.076858302	7.147510258	3.500091921	0.000777675	0.005215896	-1.604790804
GPR39	-0.635470596	-0.865455551	-3.498618484	0.000781386	0.005238453	-0.722894382
RCOR1	-0.296378719	4.6078857	-3.498471015	0.000781759	0.005238617	-1.572034122
LIF	0.886796308	1.379831199	3.497681156	0.000783756	0.005249665	-0.861661989
METTL3	0.233091042	3.394746689	3.496842927	0.00078588	0.005261556	-1.391315317
GREM1	-0.284565148	10.85685006	-3.496365233	0.000787094	0.005267338	-1.397696117
SSB	0.108335664	6.178630201	3.495705055	0.000788773	0.005276234	-1.628473189
YTHDC1	0.131904303	5.058275286	3.493437099	0.000794569	0.005312646	-1.588042486
NCL	0.114288249	9.27532227	3.492651106	0.000796587	0.005321415	-1.508917095
HMGCR	-0.524512336	6.877863802	-3.492773359	0.000796273	0.005321415	-1.624129129
PYHIN1	1.20986352	-3.438538938	3.491997066	0.00079827	0.005329713	-0.623372683
HIST1H4I	0.256514268	2.670197063	3.491880965	0.000798569	0.005329713	-1.32132089
LANCL1	-0.252917908	5.584114155	-3.491756224	0.000798891	0.005329713	-1.625488406
DNAJC17	0.222670834	2.601543973	3.491577873	0.000799351	0.005330421	-1.304119756
CRCP	-0.197265384	4.08297045	-3.490687311	0.000801651	0.005343392	-1.537650257
FAM46A	0.323675178	6.475562028	3.489261505	0.000805346	0.005365649	-1.646741583
PRKD3	-0.301276678	6.181553863	-3.489065925	0.000805854	0.005366661	-1.648146562
CBARP	-0.335594693	2.767527913	-3.488360723	0.000807689	0.005376503	-1.298843556
TAF4	-0.182975521	3.287278591	-3.485613088	0.000814875	0.005421944	-1.48357124
MINDY1	0.172917071	3.508709087	3.485329409	0.000815621	0.005424508	-1.435005983
ATG101	-0.159438253	4.779394231	-3.483979386	0.000819177	0.005445754	-1.598577784
TRIM32	0.200987	5.178829829	3.483470341	0.000820522	0.005452288	-1.639977374
UBE2D3	-0.080065099	7.929629293	-3.481427744	0.000825938	0.005485862	-1.635126024
SMARCC2	-0.119798943	6.136754647	-3.481272081	0.000826352	0.005486194	-1.669157078
NIPAL4	0.751634247	-1.642971042	3.480423909	0.000828613	0.005498776	-0.722058544
APOLD1	0.308287006	0.544944238	3.479822761	0.000830218	0.005507004	-1.008328563
ACOX2	-0.16972498	3.686839009	-3.479601368	0.00083081	0.005508506	-1.474557462
LRP12	-0.252749961	5.232634376	-3.479292209	0.000831638	0.005511566	-1.646985443
TMEM116	-0.175737679	2.79379766	-3.478348139	0.000834169	0.005525911	-1.353490908
LPP	-0.205130221	6.662250642	-3.478159087	0.000834677	0.005526845	-1.679240205
PGRMC2	-0.097188331	7.7403914	-3.476584531	0.000838917	0.005552483	-1.651357905
TADA1	-0.186715783	2.468011259	-3.476099232	0.000840229	0.005558719	-1.355955794
PEG13	-0.566795062	-0.365634216	-3.475712466	0.000841275	0.005563198	-0.862781599
CDCP1	-0.407310058	2.23311105	-3.475006867	0.000843187	0.005570959	-1.286503565
PIK3CB	0.269165708	3.44721703	3.475006408	0.000843188	0.005570959	-1.481245942
ECI2	0.173082984	5.269412494	3.474553213	0.000844418	0.005576641	-1.661547699
RPL22L1	0.206986167	5.707824889	3.473160627	0.000848209	0.005599221	-1.685724078
ZNF670	-0.396995269	0.787410614	-3.472693371	0.000849485	0.005605185	-1.027259393
PTBP1	0.096368711	7.729849333	3.472313156	0.000850524	0.005607707	-1.658488935
CEP170	-0.356809836	5.128582928	-3.472281178	0.000850611	0.005607707	-1.667929564
POLN	-0.44198384	0.082189625	-3.471905931	0.000851638	0.005612022	-0.913700619
TPBG	-0.280671884	6.58306804	-3.471223328	0.00085351	0.005621893	-1.700833469
TPGS1	0.345892524	3.015329994	3.470932624	0.000854308	0.00562469	-1.417946623
UFD1	0.197717301	5.232015103	3.46947969	0.000858307	0.005646085	-1.678830922
GOLPH3	-0.196665122	6.777177416	-3.469553444	0.000858103	0.005646085	-1.706101899
PHF14	0.131642108	4.745889535	3.469057511	0.000859472	0.005651284	-1.646940243
DHX29	-0.167366876	5.718691151	-3.4688889	0.000859938	0.00565188	-1.703358985
CAPN15	-0.129306161	5.105728619	-3.468414213	0.000861251	0.005655985	-1.678882667
TBC1D2B	-0.36762041	6.874553462	-3.468391542	0.000861314	0.005655985	-1.707210928
PTP4A2	-0.077573175	8.142175113	-3.46659584	0.000866297	0.005686232	-1.662714875
COPG1	0.101342432	7.886035643	3.464108195	0.000873246	0.005729348	-1.679804997
KIAA1143	-0.367424864	3.840114007	-3.461600304	0.000880305	0.005773148	-1.583392511
ZFAND6	-0.15013033	5.268632775	-3.46057214	0.000883215	0.005789708	-1.710076658
PTPN21	-0.165999697	5.321439332	-3.459954548	0.000884967	0.00579867	-1.717742735
FARSB	0.132050918	5.478744497	3.45947177	0.000886339	0.005805134	-1.723568624
HSPA8	0.156544048	9.938707479	3.457708759	0.000891366	0.005835521	-1.578431108
PNPLA6	0.123436792	5.93706266	3.456881609	0.000893734	0.00584848	-1.739310395
MOGS	0.110622964	5.307167141	3.456405292	0.0008951	0.005854877	-1.729686762
RHOA	0.083136581	9.484159322	3.455330096	0.000898191	0.005872546	-1.620413952
PHF23	-0.332080641	5.726053083	-3.455101008	0.000898851	0.005874311	-1.747254393
ILF3-AS1	-0.180509524	2.889159939	-3.45479378	0.000899736	0.005874327	-1.465482463
FMR1	-0.178681084	4.346495581	-3.454720884	0.000899947	0.005874327	-1.66602306
GREM2	-0.226157703	7.565617287	-3.454694448	0.000900023	0.005874327	-1.720911622
FKRP	0.134223816	3.861484264	3.454535522	0.000900482	0.005874775	-1.607340106

Gene	Log fold change	Average Expression	t	P-value	Adjusted P-value	B
ATAD2	-0.265186838	3.683879379	-3.454140829	0.000901622	0.005877122	-1.590262628
MBOAT7	0.123211319	6.725798185	3.454153092	0.000901586	0.005877122	-1.752519514
PEX13	-0.154842549	3.853787195	-3.453725324	0.000902823	0.005880599	-1.613467809
XRCC5	0.083393927	8.302150959	3.453686382	0.000902936	0.005880599	-1.689310519
UBAP2	0.128136265	4.824195194	3.452495385	0.000906389	0.005900537	-1.713125086
ZNF48	-0.266772197	2.688541279	-3.451160043	0.000910275	0.005923277	-1.465807255
RALY	0.196847561	7.035854062	3.450471161	0.000912286	0.0059338	-1.75749168
MRE11	-0.165212135	3.678078543	-3.44995684	0.00091379	0.005941018	-1.586540259
ATM	-0.316296716	5.341834456	-3.449659698	0.00091466	0.005944109	-1.751122682
ACADSB	-0.202372902	3.742720628	-3.44938786	0.000915457	0.005946721	-1.622890436
TRAP1	0.133515015	4.641932455	3.44841424	0.000918316	0.00596272	-1.701483313
TMEM179B	0.179835381	4.563691092	3.448001359	0.000919531	0.005968036	-1.694439142
SLC30A9	-0.132529255	5.681041488	-3.446717322	0.000923319	0.00599004	-1.765113606
DFFB	-0.314735982	0.696547653	-3.446358575	0.00092438	0.005994341	-1.165033232
ZNF800	-0.299515083	4.341723492	-3.443493965	0.000932892	0.006046942	-1.654007766
GPX1	0.194115019	7.234744238	3.443166968	0.000933869	0.006050667	-1.774848214
THOC2	-0.219550281	5.051311387	-3.442939656	0.000934548	0.006052466	-1.746213774
PARP4	-0.204476042	5.955243552	-3.442445665	0.000936026	0.006059432	-1.782387376
LINC00665	-0.185213418	2.742644141	-3.441576396	0.000938632	0.006073693	-1.46694696
INPP5D	0.591747316	-1.684886869	3.441144345	0.00093993	0.00607687	-0.826483363
SF3B2	0.117392038	8.039582576	3.441209905	0.000939733	0.00607687	-1.744170301
KIF2C	-0.341056389	2.469385035	-3.440304681	0.000942457	0.006090595	-1.420220874
FOXF2	-0.181499135	3.562790976	-3.438910123	0.000946669	0.006113853	-1.633991355
BET1	0.124613017	4.377828161	3.438844304	0.000946868	0.006113853	-1.713393162
WDR61	0.123259851	4.359525443	3.438092877	0.000949145	0.006125929	-1.717332998
NUDT19	-0.167552544	3.60586929	-3.437112357	0.000952124	0.006141818	-1.608388908
CCNH	-0.218106755	3.802064041	-3.437014378	0.000952422	0.006141818	-1.651209611
ENAH	-0.260702512	7.499572036	-3.436291272	0.000954625	0.006150904	-1.774117657
RNPEP	0.120924772	6.150292534	3.436283918	0.000954648	0.006150904	-1.806555896
KDM4C	0.212900116	2.243529528	3.435928777	0.000955732	0.006155255	-1.367413721
IRF7	0.229507873	3.162413979	3.434564957	0.000959905	0.00617685	-1.562766962
CKB	0.266052655	4.754313605	3.43465815	0.000959619	0.00617685	-1.744506504
UBE2K	-0.140868638	6.017792523	-3.434246771	0.000960881	0.006180491	-1.811362709
VPS33A	0.141039295	3.488089358	3.433614987	0.000962822	0.006190332	-1.632340619
FNIP2	-0.343426873	4.606094739	-3.431289063	0.000969999	0.006231618	-1.754878087
SIVA1	0.221638884	4.768668034	3.431265946	0.000970071	0.006231618	-1.763398419
ZNF75A	0.206990667	2.136221349	3.430528092	0.000972358	0.00624099	-1.412326258
NAF1	-0.269351245	3.040285619	-3.430654091	0.000971967	0.00624099	-1.589802258
LLPH	-0.113081779	3.847898578	-3.430234199	0.000973271	0.006244187	-1.671970647
TRIM2	-0.30460856	5.856099962	-3.429604356	0.000975229	0.006253829	-1.822905381
SLC5A3	-0.466186538	5.671111002	-3.429483963	0.000975604	0.006253829	-1.82556813
UAP1L1	0.17480235	5.523121159	3.429323386	0.000976104	0.006254373	-1.812910771
HOOK3	-0.261430838	5.502229192	-3.42855108	0.000978513	0.00626714	-1.820159741
MAP3K1	-0.419573337	1.389539381	-3.428358951	0.000979113	0.006268318	-1.338483052
UQCRC1	0.19656138	6.651896727	3.426758125	0.000984126	0.006297737	-1.83611834
C2CD2	-0.209305626	4.662901941	-3.426282936	0.000985619	0.006304611	-1.779000129
IDH3G	0.21229172	5.597020857	3.42567014	0.000987547	0.006314264	-1.823240507
TTC28	-0.318908843	6.483897061	-3.425313493	0.000988671	0.006318768	-1.840308135
MRPL38	0.394619387	0.223703687	3.423396883	0.000994731	0.006354804	-1.116920965
ERCC6	-0.333600193	3.344735646	-3.42308049	0.000995735	0.00635852	-1.667592693
FAM105A	0.492945506	-1.277034498	3.422537534	0.00099746	0.006366603	-0.942582013
OSGIN1	-0.324132883	3.356745763	-3.422416058	0.000997846	0.006366603	-1.584336293
USP35	-0.222484434	2.676570771	-3.421021289	0.001002291	0.006392258	-1.572587569
CSNK2B	0.196051965	4.385565212	3.42056302	0.001003756	0.006395227	-1.760652931
SLC43A3	-0.222537469	6.168430816	-3.4204772	0.00100403	0.006395227	-1.852213353
EIF2AK2	-0.271517772	6.18714437	-3.420590089	0.001003669	0.006395227	-1.854800983
CLIP4	-0.170207606	5.563345765	-3.420070645	0.001005332	0.006400059	-1.841954431
USP25	-0.100453215	5.541046678	-3.419974823	0.001005639	0.006400059	-1.84195985
WSB1	-0.339131439	4.513856037	-3.419214294	0.001008078	0.006410168	-1.724152713
GRAMD3	-0.267195593	5.098709264	-3.419272405	0.001007892	0.006410168	-1.786474135
SNRPC	0.219852074	5.36435431	3.418790812	0.001009439	0.006412282	-1.835483569
AP1S1	0.135251888	5.670620448	3.418699655	0.001009732	0.006412282	-1.848801178
SLC39A9	-0.194439874	5.628252051	-3.418581154	0.001010113	0.006412282	-1.849492213
PSMG2	0.173367694	5.870394288	3.418633476	0.001009945	0.006412282	-1.854237443
DTNA	-0.276578528	2.508625193	-3.41779638	0.001012641	0.006424578	-1.500221455
C17orf51	-0.18297679	4.181546502	-3.417715106	0.001012903	0.006424578	-1.752080712
ARHGAP21	-0.185343555	6.769833038	-3.416306694	0.001017456	0.00645074	-1.866471005
TMEM60	-0.169254059	4.082389166	-3.415315453	0.001020672	0.006461836	-1.743379791
INTS4	0.099736117	4.094853121	3.415212107	0.001021008	0.006461836	-1.758897584
PATL1	-0.206227389	5.966114027	-3.415263904	0.001020839	0.006461836	-1.868403282

Gene	Log fold change	Average Expression	t	P-value	Adjusted P-value	B
FYCO1	-0.260117155	6.782931126	-3.41529731	0.001020731	0.006461836	-1.868551513
UBE2I	0.105487201	6.740166105	3.415106483	0.001021351	0.006461836	-1.869042773
KMT2E	-0.245488165	5.885080405	-3.413891395	0.001025309	0.006484152	-1.871727959
SMYD5	-0.097725809	4.982741525	-3.413530753	0.001026486	0.006488873	-1.828429146
RNPS1	-0.173667518	4.740413378	-3.413079977	0.001027959	0.006492801	-1.820126757
UTP6	0.145979099	4.942510143	3.413076979	0.001027969	0.006492801	-1.825564767
PAN3	-0.255487215	4.027113539	-3.412921145	0.001028479	0.006493298	-1.766454918
NUP54	-0.123896039	5.260574437	-3.411166932	0.001034235	0.006526902	-1.861128489
PAWR	-0.268352241	4.382820025	-3.409812101	0.001038701	0.006552343	-1.777319032
PHB	0.134100506	5.402449973	3.407928157	0.001044942	0.006588951	-1.871475291
CCDC92	0.197895818	6.349789467	3.407309177	0.001047	0.006599166	-1.894055529
PURA	-0.229088688	5.277527283	-3.4061163	0.001050977	0.006621463	-1.873710891
IP6K2	0.179946665	4.603300081	3.404669064	0.001055821	0.006649203	-1.81639656
RUBCN	-0.155697609	4.302767589	-3.404467791	0.001056497	0.006650676	-1.805526457
BUD23	0.171889713	5.295759585	3.404160159	0.00105753	0.006654398	-1.87779249
TIMELESS	-0.169893363	3.812881166	-3.403900818	0.001058401	0.006657102	-1.735165735
GPX8	-0.108576218	7.486409877	-3.403217256	0.001060702	0.006668788	-1.885124778
CAMTA1	0.234688722	4.178821865	3.402812883	0.001062065	0.00667017	-1.792882423
TMEM8A	0.126121168	6.447896332	3.402985383	0.001061484	0.00667017	-1.90779174
ATP6V1E1	0.107581598	6.497789158	3.402758057	0.00106225	0.00667017	-1.908097475
ACBD6	0.16315036	5.547800913	3.401347701	0.001067019	0.00669732	-1.89694279
NAGPA	0.139333387	4.364989811	3.400934859	0.001068418	0.006703312	-1.819688829
TAF13	-0.222539481	4.724611693	-3.400043417	0.001071446	0.006719511	-1.851375276
SLC25A12	-0.121340749	4.308761142	-3.399391015	0.001073667	0.00673064	-1.824247688
PPDPF	0.281890043	7.479238945	3.397597138	0.001079797	0.006764404	-1.905854315
C19orf43	0.213054828	6.947133289	3.397552125	0.001079952	0.006764404	-1.919529354
SAR1A	-0.126057703	7.544992381	-3.397016717	0.001081788	0.006773089	-1.895707599
TMEM168	-0.384716835	3.924236005	-3.395519009	0.00108694	0.006802519	-1.800870847
ANGEL1	-0.229828702	3.175233398	-3.39519384	0.001088062	0.006806711	-1.6724904
FAM193B	-0.285582081	3.265370847	-3.394710269	0.001089732	0.006814328	-1.733559029
CLCF1	-0.282545486	3.281300019	-3.394094232	0.001091863	0.006824821	-1.636847889
SGCD	-0.325000757	6.23896983	-3.393689368	0.001093265	0.006830754	-1.932902167
CDK5R1	-0.362395107	0.241563335	-3.393324573	0.001094531	0.006832266	-1.131602433
KLHL11	-0.734614883	-0.176994975	-3.393386399	0.001094316	0.006832266	-1.134964285
GAMT	0.250531717	5.242681732	3.393227377	0.001094868	0.006832266	-1.900229403
PSD3	-0.362247654	5.835421026	-3.393006897	0.001095634	0.006834213	-1.93085163
TSPOAP1	-0.306994466	2.101370722	-3.392836937	0.001096224	0.006835066	-1.526199881
HMGCS1	-0.364429178	6.92190708	-3.391770134	0.001099938	0.006855383	-1.919844947
HLA-E	0.129203636	8.63179938	3.391455968	0.001101034	0.006859375	-1.863660778
DLG2	-0.501377443	-0.833382912	-3.39114826	0.001102108	0.00686323	-1.030868139
CDT1	-0.257441552	2.9975587	-3.390634571	0.001103904	0.006871572	-1.671878891
RTL8C	0.185338129	7.719794511	3.389767353	0.001106941	0.006887635	-1.917606852
HLCS	-0.269809162	4.911234344	-3.387395161	0.001115291	0.006933862	-1.910204804
GLRX3	0.138192491	5.493439488	3.38743912	0.001115136	0.006933862	-1.938187305
SMIM3	-0.242077975	4.841932451	-3.387217151	0.00111592	0.006934911	-1.872871395
CMTM7	0.228717524	3.297640628	3.386331843	0.001119053	0.006951513	-1.724262984
TANC1	0.377811267	3.992294632	3.38569339	0.001121318	0.00696271	-1.857137651
CPSF3	0.110711231	5.236781811	3.385068091	0.00112354	0.006973634	-1.932183073
LDB2	-0.189053534	4.950270054	-3.383747477	0.001128247	0.006999963	-1.894471808
LZIC	-0.231529862	4.578209648	-3.382129907	0.001134037	0.007032992	-1.897575096
KRBOX4	-0.19484222	2.18863759	-3.381290047	0.001137055	0.007047133	-1.557610668
ANKRD26	0.227756523	3.005540625	3.381234939	0.001137253	0.007047133	-1.712774574
STRAP	0.08168038	7.104749927	3.380984584	0.001138154	0.007049817	-1.960775396
TCFL5	0.124981327	4.645576654	3.379951885	0.001141878	0.007069977	-1.905797679
PRKG1	-0.333022603	5.509786375	-3.37788872	0.001149353	0.007113332	-1.968377032
MAGEF1	-0.168843028	4.635653305	-3.377264095	0.001151625	0.007124466	-1.911684125
COG4	-0.104775095	5.895662801	-3.377133873	0.001152099	0.007124474	-1.976987195
KDM1B	-0.26295911	4.602598516	-3.376559333	0.001154193	0.00713157	-1.914116397
PSMA5	0.164031884	5.885989407	3.376600822	0.001154042	0.00713157	-1.980262373
MAP1B	-0.30707686	8.598287048	-3.376328064	0.001155037	0.00713386	-1.899928021
DNAJB4	0.289230663	4.632870545	3.375844671	0.001156804	0.007141839	-1.911427755
KLHDC2	-0.099891647	4.782172718	-3.374995243	0.001159913	0.007158104	-1.920806829
CCDC93	-0.257200247	5.495104535	-3.37299392	0.001167271	0.00720056	-1.985147265
NUP153	-0.411128904	5.501163225	-3.372864085	0.00116775	0.007200565	-1.980474087
YES1	-0.203759823	6.139752248	-3.371533164	0.001172669	0.007227939	-2.001212266
SOX9	-0.348385166	3.989759656	-3.369492071	0.001180251	0.007271697	-1.880286163
STXBP4	-0.184685306	3.496400562	-3.368337917	0.001184559	0.007295253	-1.813228895
CEP55	-0.36209571	3.020681392	-3.366496097	0.001191464	0.00733478	-1.716264346
CREBL2	-0.112869055	6.55994004	-3.364580565	0.001198685	0.007376223	-2.021840373
BPGM	0.234589082	3.85672248	3.363323063	0.001203448	0.00740251	-1.851500996

Gene	Log fold change	Average Expression	t	P-value	Adjusted P-value	B
CDKN2A	0.214151254	4.219818169	3.362820925	0.001205355	0.007411214	-1.914692956
LIMD2	0.2104294	2.924597232	3.362522656	0.001206489	0.00741516	-1.784117997
C3orf67	-0.287918385	1.187505077	-3.3612338	0.001211402	0.007442314	-1.383464809
GDNF-AS1	0.551228793	-1.298585879	3.359330935	0.001218688	0.007484028	-1.087576547
IFRD1	0.279554752	3.969340128	3.358761792	0.001220876	0.007494406	-1.928858054
ARFRP1	0.182934224	4.695713089	3.35791224	0.001224148	0.007511431	-1.986432575
TRIM52	-0.231049298	1.759386629	-3.357748948	0.001224778	0.007511512	-1.542929345
ROR2	0.194173632	5.404381789	3.357650286	0.001225158	0.007511512	-2.011491821
YPEL3	0.301951797	4.964538293	3.35697995	0.001227748	0.007524327	-1.965705481
MAD1L1	0.194429278	5.121614358	3.356329542	0.001230266	0.00753669	-1.999917069
SLC12A2	-0.34473257	3.981168418	-3.356004819	0.001231524	0.007541292	-1.942173581
RBM39	-0.151627276	6.380794647	-3.355877541	0.001232018	0.007541292	-2.04586433
ALDH6A1	-0.158970856	4.180486699	-3.355590002	0.001233134	0.007545057	-1.934372888
SOX11	-0.473381716	1.7816466	-3.355350007	0.001234066	0.007545576	-1.551054126
SP1	-0.236950195	6.322856822	-3.355310284	0.001234221	0.007545576	-2.048979392
WTAPP1	-1.022529109	-2.915476463	-3.355022903	0.001235338	0.007549343	-0.993614094
IQCA1	-0.772169631	-1.501544269	-3.354248833	0.001238352	0.007563582	-1.067624382
CUX1	0.110914362	6.76880595	3.354166747	0.001238672	0.007563582	-2.049957631
TPM4	0.122592447	9.91754617	3.353774715	0.001240202	0.007569854	-1.89179503
SLC52A2	0.177188478	5.347903905	3.351518314	0.00124904	0.007620713	-2.036965012
THEMIS2	0.241334494	2.038766014	3.351090247	0.001250724	0.007627895	-1.679431454
ZNF480	0.171873532	3.823526492	3.349738418	0.001256054	0.007654229	-1.913171576
SKP1	0.078657705	6.624664829	3.349737436	0.001256058	0.007654229	-2.064279602
NKAP	0.12178319	4.315082287	3.348731787	0.001260037	0.007675372	-1.964737503
AGPS	-0.228862512	5.356386162	-3.348368683	0.001261476	0.007681036	-2.04679188
C19orf48	0.132570378	4.472322093	3.348014272	0.001262883	0.007683389	-1.987112526
GID8	-0.090398477	5.96613208	-3.348025541	0.001262838	0.007683389	-2.068374434
FAM210A	-0.21145131	3.82461132	-3.347062181	0.001266669	0.007697098	-1.927130437
FBXL20	0.2318561	4.065768714	3.347089728	0.00126656	0.007697098	-1.963355662
CTDSPL2	-0.265247842	4.396635241	-3.34713004	0.001266399	0.007697098	-1.982937873
TRIB1	0.302313967	3.62736882	3.34654358	0.001268736	0.00770612	-1.795045066
DDX49	0.207583964	5.597052107	3.346433034	0.001269177	0.00770612	-2.061534767
MADD	0.165028974	4.961093935	3.346020102	0.001270826	0.007709914	-2.049660748
TMEM109	0.105502871	7.171075708	3.346054317	0.001270689	0.007709914	-2.06558736
RNF19A	-0.240878212	4.845406702	-3.34331689	0.001281667	0.007772558	-2.022300702
PRPS2	0.132739044	4.194869802	3.34311784	0.001282469	0.007774291	-1.985694802
CYBA	0.282066521	5.725885512	3.342829014	0.001283633	0.007778218	-2.0701252
RGMB	-0.193959804	5.708384809	-3.341729337	0.001288075	0.007801994	-2.062447392
ZBED6	-0.645077231	1.422917967	-3.341390903	0.001289445	0.007807152	-1.607355525
NOD1	-0.257259186	2.621129408	-3.341213534	0.001290163	0.007808364	-1.735908909
SYNJ2	-0.244691931	6.831374369	-3.340743028	0.001292071	0.007816769	-2.076073376
KIF2A	-0.343390823	4.767270152	-3.340458255	0.001293227	0.007820622	-2.052326358
ZYX	-0.170317759	9.079186863	-3.339527477	0.001297012	0.007840363	-1.997446461
TFB2M	-0.161909572	3.598560964	-3.339320102	0.001297856	0.007842322	-1.898309763
FLJ37453	0.399173286	-0.010569217	3.337643404	0.001304705	0.007877386	-1.275491198
PPTC7	-0.285785649	4.078242439	-3.337741282	0.001304304	0.007877386	-1.969411666
LCTL	-0.98083853	-3.015420429	-3.337407998	0.001305669	0.007877548	-1.04240971
MFSD14B	0.145232744	5.44906755	3.337381527	0.001305778	0.007877548	-2.08831329
EGFR	-0.394064108	7.382863654	-3.336741917	0.001308401	0.007884532	-2.083970403
TMED3	0.12577607	6.956073881	3.336716526	0.001308506	0.007884532	-2.098079748
YTHDF3	-0.349905886	6.652844491	-3.336918597	0.001307676	0.007884532	-2.102097104
PPP3CC	-0.135315458	3.681963541	-3.336148603	0.00131084	0.007895436	-1.936970222
COG1	0.102443656	4.727977434	3.335549738	0.001313305	0.007907123	-2.052260002
SASH1	-0.211812788	7.215031413	-3.334735742	0.001316663	0.007924173	-2.097615044
ENO3	0.444007325	-0.272695792	3.332431507	0.001326212	0.007968908	-1.283327402
CNOT7	-0.104582128	5.565986102	-3.332663972	0.001325245	0.007968908	-2.10103112
LRPAP1	0.157329408	7.088343923	3.332776799	0.001324777	0.007968908	-2.105894521
EGLN1	-0.131294285	5.631939821	-3.332432747	0.001326206	0.007968908	-2.106121644
ZDHHC17	-0.288042624	4.007282439	-3.331821797	0.001328749	0.007980972	-1.984867473
ELAC2	0.102200191	5.660476503	3.331536118	0.00132994	0.007984939	-2.108590383
TMED8	-0.228729727	3.93711071	-3.331337756	0.001330767	0.007986723	-2.003865798
ISG15	0.31178056	4.371719869	3.330432204	0.00133455	0.008006235	-2.018345698
SMARCA4	0.090735463	6.6391332	3.329289196	0.001339339	0.008031767	-2.122879937
NANP	-0.302781928	3.714574901	-3.328555175	0.001342423	0.008047057	-1.947961998
MARCH6	-0.203708461	6.220274583	-3.327949287	0.001344973	0.00805914	-2.129519452
RPS6KC1	0.163982202	5.088635713	3.325921763	0.001353542	0.008107259	-2.092858252
CNTF	-0.694230874	-1.055368535	-3.323960538	0.001361879	0.008153954	-1.214415024
ZNRF2	-0.212629777	2.612814509	-3.323477898	0.001363938	0.008163038	-1.806192011
MNS1	-0.226054643	1.938689119	-3.322446587	0.001368348	0.008186177	-1.65986177
CDC73	-0.342767844	4.958329254	-3.322277344	0.001369073	0.008187263	-2.099255962

Gene	Log fold change	Average Expression	t	P-value	Adjusted P-value	B
FOSB	1.507026038	2.399014547	3.321917344	0.001370616	0.008192559	-1.636136063
HIRA	0.175179497	3.756112912	3.321690357	0.00137159	0.008192559	-1.998145108
SAMD4B	-0.191344062	7.688249713	-3.321748458	0.001371341	0.008192559	-2.123964543
TFCP2	-0.098392125	4.721485911	-3.320924096	0.001374882	0.008208969	-2.091416175
IWS1	-0.132158442	5.201351274	-3.320714133	0.001375786	0.008211109	-2.122453617
ABHD12	0.139883896	5.505211564	3.319661045	0.001380326	0.008234941	-2.136260837
ZDHHC21	-0.328741465	2.652438697	-3.318551295	0.001385125	0.008255198	-1.825735766
MID1	-0.256787951	4.930445542	-3.318621019	0.001384823	0.008255198	-2.099673627
SEMA3D	-0.403027341	5.455746279	-3.318495858	0.001385365	0.008255198	-2.143186569
BCAP29	-0.103698215	6.008662888	-3.318301043	0.001386209	0.008256963	-2.156285742
STXBP5	-0.196929885	5.450903045	-3.317451317	0.001389897	0.008275658	-2.13918744
G2E3	-0.249668102	3.053312085	-3.317079445	0.001391514	0.008282012	-1.960042939
NDUFA2	0.244213329	4.064340098	3.315356263	0.00139903	0.008323456	-2.030893138
SMPD1	0.098684888	7.033026243	3.314351735	0.001403429	0.00834633	-2.160451486
RARA	-0.197189122	5.573033032	-3.313183907	0.001408559	0.008373535	-2.156194368
PLEKHA2	-0.316624751	2.4352914	-3.31217459	0.001413007	0.008396664	-1.830810903
TNIP3	-0.37775738	0.495451394	-3.311274594	0.001416985	0.008415833	-1.444730193
ARFGAP2	0.101758693	5.902517873	3.311191979	0.001417351	0.008415833	-2.173574876
SLC16A13	0.317869675	1.181073801	3.310972457	0.001418323	0.008418287	-1.674903838
NFKBIB	0.195822269	3.790992763	3.310262371	0.001421471	0.008433651	-2.026922146
PKP4	-0.145086392	4.927577169	-3.309329994	0.001425615	0.008454907	-2.150222246
CRAT	0.112866731	6.594063384	3.308470064	0.001429447	0.008474298	-2.186297724
DGKE	-0.318602773	1.454982901	-3.307263237	0.001434841	0.008502931	-1.613254824
SPRED3	-0.311555777	1.571119905	-3.3068935	0.001436498	0.008509399	-1.661554013
PLPPR3	0.533688998	-0.846802169	3.306518339	0.00143818	0.008510549	-1.277796865
STK38L	-0.268262975	4.421934173	-3.306472371	0.001438387	0.008510549	-2.111472878
CPSF6	-0.088768184	5.621213729	-3.306714614	0.0014373	0.008510549	-2.178038118
THAP9	0.332350075	1.029203905	3.305519543	0.001442669	0.00852584	-1.542333798
TMEM242	0.149856661	4.269956896	3.305658642	0.001442043	0.00852584	-2.082643203
SERP1	0.078711114	7.426713943	3.305529467	0.001442624	0.00852584	-2.171739648
NFKBIA	0.252068295	4.74152153	3.304679595	0.001446454	0.008544856	-2.116128533
SIAH1	0.192393452	3.072333779	3.303200322	0.001453143	0.008581004	-1.916745116
INAFM1	0.270250971	3.124986361	3.302918554	0.00145442	0.008585181	-1.919987425
KLF5	-0.487864355	2.982587523	-3.302465537	0.001456476	0.008593949	-1.773532038
BBS9	-0.124535213	4.337634408	-3.30166753	0.001460104	0.008611983	-2.109588686
IVNS1ABP	-0.222674986	5.704951212	-3.300920036	0.00146351	0.008628694	-2.204285724
CDC27	-0.210550693	6.296168113	-3.300200201	0.001466797	0.008644692	-2.210811815
LINC01354	-0.532929281	-0.633528642	-3.299728629	0.001468954	0.008654019	-1.265059134
LPGAT1	-0.215374784	5.554739039	-3.2985579	0.001474323	0.008682251	-2.203204317
SPCS1	0.126785501	6.100871713	3.298132417	0.001476278	0.00869037	-2.214655716
GALK1	0.232906433	4.584795212	3.297380262	0.001479741	0.008707353	-2.128974849
PPM1G	0.109816061	6.566790143	3.296040687	0.001485928	0.008740343	-2.222444306
FAM213B	0.198687682	3.939278398	3.295348941	0.001489132	0.008755771	-2.088368367
LOC100287808	-0.601968411	-1.90826703	-3.294399117	0.001493542	0.008776026	-1.223238125
JKAMP	-0.208076992	4.33498359	-3.294230896	0.001494325	0.008776026	-2.123796615
PANX1	-0.326891804	5.283134911	-3.294337078	0.001493831	0.008776026	-2.19653837
RCN3	0.23591008	8.381167218	3.292834509	0.001500834	0.008810818	-2.17044567
TPD52L2	0.07836335	7.60221458	3.292686685	0.001501524	0.008811438	-2.203426544
GABPB2	-0.170948576	3.036022441	-3.2924053	0.00150284	0.008812479	-1.959665128
ATP5C1	0.175350108	6.435426618	3.29239848	0.001502872	0.008812479	-2.233493178
PTPN1	-0.207788438	4.853898859	-3.289903125	0.001514585	0.008877706	-2.174594415
FLCN	-0.231490607	4.585723238	-3.288929245	0.001519179	0.008901174	-2.165363257
RAB3IP	-0.306748559	1.351970477	-3.288478639	0.00152131	0.00891019	-1.701698986
NOP2	-0.204064322	5.140181867	-3.28805979	0.001523292	0.00891487	-2.217072633
PCNX4	-0.225890546	5.491922628	-3.288081514	0.001523189	0.00891487	-2.22751328
UBA6	-0.271863464	4.535133199	-3.287479257	0.001526044	0.008927508	-2.162115919
RBM15	-0.225422019	2.428941939	-3.287019839	0.001528226	0.008933329	-1.834464358
TUT1	0.185066447	2.333310399	3.287053304	0.001528067	0.008933329	-1.941721748
PAK1	0.119482375	5.015145655	3.286030647	0.001532932	0.008957365	-2.204852319
ATRX	-0.310706302	5.942271456	-3.284993685	0.00153788	0.008982795	-2.249916803
SHQ1	0.118491334	4.708591681	3.284292544	0.001541234	0.008998898	-2.200377766
NAXD	0.115005624	4.455908544	3.282609434	0.001549314	0.00904257	-2.184127887
PTGER1	0.773282085	-1.624887598	3.282241742	0.001551085	0.009049397	-1.252649225
TONSL	0.20507939	2.922356872	3.281950596	0.001552488	0.009054077	-1.96815202
SYNE2	-0.292425728	4.109769046	-3.281679372	0.001553796	0.009054696	-2.150113675
ABCF2	0.095195455	6.431963292	3.281777359	0.001553324	0.009054696	-2.264099439
AKT1	0.097467608	6.986128542	3.279879541	0.001562504	0.009101921	-2.263474373
OAZ1	0.148018526	8.347866992	3.27866068	0.001568428	0.009132892	-2.210350765
PPP1R12B	-0.361005102	1.452352365	-3.278163412	0.00157085	0.009143464	-1.742048176
ZNF35	-0.21640566	3.183629839	-3.277089566	0.001576094	0.009166901	-2.023255281

Gene	Log fold change	Average Expression	t	P-value	Adjusted P-value	B
BRD4	-0.113339448	6.503785662	-3.277152176	0.001575787	0.009166901	-2.277630631
SAP18	0.18221582	6.706825719	3.275702992	0.001582888	0.009202867	-2.280464201
KIF18B	-0.556087332	0.347449908	-3.275348331	0.001584631	0.009205891	-1.532133919
ZNF142	-0.178717181	4.11726683	-3.275382785	0.001584461	0.009205891	-2.190550597
BOLA1	0.362912939	2.538348106	3.275092928	0.001585887	0.009208124	-1.922068956
SOBP	0.232983506	3.895379749	3.275021575	0.001586238	0.009208124	-2.163245581
HLA-F	0.302375189	1.781237634	3.274612841	0.00158825	0.009216254	-1.798413792
ZNF607	-0.301791392	1.684454597	-3.273978056	0.00159138	0.009220208	-1.934554634
MMP11	0.237794717	2.851016764	3.274338887	0.0015896	0.009220208	-2.02854039
SNAP47	0.097160423	4.86463363	3.273984247	0.001591349	0.009220208	-2.22885507
RAB3GAP2	-0.265271623	5.247134924	-3.274191857	0.001590325	0.009220208	-2.272652397
TAGLN2	0.144308794	7.782506885	3.271879695	0.001601767	0.009276821	-2.258542161
C12orf60	0.571550972	-1.300787833	3.271612172	0.001603095	0.009280949	-1.356023766
SSNA1	0.209614912	4.979460725	3.271035319	0.001605964	0.009293621	-2.242193036
CDC123	0.110864122	5.550977284	3.27092409	0.001606518	0.009293621	-2.28352015
C12orf76	0.238370739	1.560369708	3.27028414	0.001609707	0.009308496	-1.811061692
NR1H2	0.15730949	6.305181585	3.26982499	0.001611999	0.009318172	-2.297952823
HYKK	0.458584474	0.054518706	3.268868351	0.001616784	0.009342248	-1.49725248
AP1G1	-0.318098616	6.279043912	-3.268053107	0.001620873	0.00936228	-2.303756036
PPA2	0.116263233	4.914961425	3.267837635	0.001621955	0.00936494	-2.256949418
CISD1	-0.111716754	4.576402071	-3.267421933	0.001624045	0.009373413	-2.224671509
RAPGEF6	0.25755027	3.240990295	3.265100955	0.001635759	0.009433793	-2.056361374
NUCB1	0.115414787	9.170904291	3.265120222	0.001635661	0.009433793	-2.204587194
SRSF10	-0.111990225	5.639692449	-3.264927572	0.001636637	0.009435246	-2.300176206
INPP5B	-0.264551959	2.727513312	-3.264396004	0.001639332	0.009447169	-2.013075746
STX17	-0.31649568	3.255622926	-3.263408145	0.001644352	0.009472474	-2.091029252
SHISA4	0.204428501	4.929634603	3.262788104	0.00164751	0.00948704	-2.264456133
NBPF9	-0.319381701	2.145838879	-3.262567972	0.001648633	0.009489877	-1.937640152
RNF166	0.166293097	3.007809659	3.260624748	0.001658574	0.009543454	-2.048445257
XYLB	-0.261496465	2.349570372	-3.260322624	0.001660125	0.009548729	-1.931300465
ARCN1	-0.170926281	7.929784895	-3.259588522	0.001663898	0.00956313	-2.283115697
P3H3	0.164682929	6.921177317	3.259662458	0.001663518	0.00956313	-2.32337921
POU3F2	0.887726972	-3.389150479	3.259434889	0.001664689	0.009564026	-1.270808429
ANP32B	0.200350836	7.417519784	3.258958031	0.001667146	0.009574488	-2.311946659
DLG3	-0.147158432	3.055496867	-3.258290378	0.001670591	0.009590617	-2.020716709
ZBTB33	-0.281904514	4.571290088	-3.258009459	0.001672042	0.009594144	-2.270849766
ALKBH7	0.234239575	5.343379925	3.257925016	0.001672479	0.009594144	-2.303554894
FAM8A1	-0.124880393	5.909476391	-3.256943442	0.001677562	0.009619637	-2.331011579
CLPB	0.173216053	3.010024354	3.256301883	0.001680891	0.009631399	-2.071390187
RANBP2	-0.225265949	6.253890805	-3.256402821	0.001680367	0.009631399	-2.337437518
ERI1	-0.203829015	4.645240352	-3.256070492	0.001682094	0.009634624	-2.27405198
ZC3H10	-0.167693694	2.723872693	-3.255753545	0.001683742	0.0096404	-2.052441895
UTP23	-0.246958582	4.260827769	-3.255352308	0.001685831	0.009648693	-2.234742712
LGALS8	-0.139650271	5.641767096	-3.255089605	0.0016872	0.009652861	-2.32906248
SACS	-0.362188	5.982548921	-3.254853721	0.00168843	0.009656232	-2.337251413
OTULIN	-0.222148591	4.273871708	-3.254403358	0.001690781	0.009666008	-2.257451086
LYRM2	-0.112762522	4.909786658	-3.253194776	0.001697106	0.009698482	-2.297086735
B4GALT7	0.112530642	4.303827351	3.252880321	0.001698755	0.009704225	-2.250107772
ZNF205	0.199239616	3.017005368	3.252294703	0.00170183	0.009718107	-2.110437898
TNRC18	0.163201025	7.251181503	3.251446901	0.001706291	0.00973989	-2.336875706
RCAN3	0.246833467	2.455784435	3.251063372	0.001708313	0.009747737	-2.017564772
SORBS1	-0.419934093	0.756990613	-3.250590059	0.001710811	0.009758296	-1.70295917
FADS1	0.296292354	8.619203198	3.249291847	0.001717681	0.009793771	-2.263827085
GADD45GIP1	0.265123145	5.573462503	3.248027667	0.001724395	0.009824617	-2.342979186
SLU7	0.086916925	5.71957031	3.248131089	0.001723845	0.009824617	-2.354435087
TRIM33	-0.33241974	4.628776701	-3.246912237	0.00173034	0.00985476	-2.313615096
ZNF148	-0.275727519	5.665327483	-3.246580192	0.001732113	0.009861131	-2.355198985
LAS1L	0.133045169	4.721972991	3.2460275	0.001735069	0.00987008	-2.306227537
FARP2	-0.247106908	5.006668063	-3.246047286	0.001734963	0.00987008	-2.342409744
USP11	0.126071198	6.414960399	3.245918805	0.001735651	0.00987008	-2.368001326
LDHB	0.096784875	8.585442998	3.245654841	0.001737064	0.009874391	-2.29294276
HNRNPL	0.155025597	5.90159304	3.245158397	0.001739726	0.009885791	-2.366814616
SF3B6	0.149081158	5.530833331	3.24479004	0.001741703	0.009893295	-2.355622427
ARID5A	-0.27894996	4.627939506	-3.244409717	0.001743747	0.009898996	-2.262051057
USP5	0.086356315	6.585597176	3.244358751	0.001744021	0.009898996	-2.372050732
DLAT	-0.154826571	5.01060562	-3.24359628	0.001748126	0.009918558	-2.337223215
GSG2	-0.459480931	-0.238502388	-3.24296564	0.001751528	0.009934119	-1.565127665
PRIM2	-0.201039274	3.02221562	-3.24283044	0.001752258	0.00993452	-2.131183919
LOC100128398	-0.299979519	0.421174705	-3.241569431	0.001759082	0.009955973	-1.67252348
DDX19A	0.144530066	4.007142971	3.241820494	0.001757722	0.009955973	-2.255473684

Gene	Log fold change	Average Expression	t	P-value	Adjusted P-value	B
TUBG1	0.20590769	4.518485872	3.241729045	0.001758217	0.009955973	-2.300794873
TSPAN17	0.103942684	5.426908594	3.241777053	0.001757957	0.009955973	-2.359033734
ARFGAP1	0.10955132	6.182555375	3.241520623	0.001759347	0.009955973	-2.380063023
NDUFAF6	0.202268005	2.746050043	3.241227283	0.001760938	0.009961235	-2.076317157
TTLL12	0.114317433	5.556258207	3.240945214	0.001762469	0.009966155	-2.370310873
P3H4	0.11470248	5.381786404	3.240740896	0.001763579	0.009968689	-2.35979918
ZSWIM6	-0.291386277	3.109707887	-3.240402678	0.001765418	0.00997534	-2.081315979
S100A3	0.255671955	2.274424054	3.239538237	0.001770126	0.009998192	-1.995079438
BANP	0.195945058	2.642754272	3.238730664	0.001774535	0.010019339	-2.077031331
ZBED3	-0.160483171	3.919199864	-3.237545437	0.001781024	0.010052211	-2.238704693
FBXL17	-0.268129255	4.457325546	-3.236657053	0.001785903	0.010075972	-2.312077579
ELFN1	0.273863485	4.971541276	3.236427314	0.001787167	0.010079326	-2.369912463
FMNL1	0.208805468	3.488331792	3.236063841	0.001789168	0.010086835	-2.183264798
SLC2A5	0.415160328	0.034489333	3.235890264	0.001790124	0.010088037	-1.599310407
FAM76A	-0.229785167	3.280498601	-3.235782099	0.00179072	0.010088037	-2.184180334
LHPP	0.213841984	4.550876163	3.235594	0.001791757	0.010090106	-2.313187279
EXOG	-0.193613751	2.739761964	-3.235417075	0.001792734	0.01009183	-2.136011809
WDPCP	-0.236518729	1.907418537	-3.234592607	0.001797289	0.010112781	-1.937490024
TRPC1	-0.364770955	2.518830723	-3.234500573	0.001797798	0.010112781	-2.055016483
MMS19	0.116337743	5.77489551	3.233960063	0.001800791	0.010125835	-2.39312344
ZNF654	0.214823641	3.798547372	3.233621245	0.001802669	0.010128836	-2.267180812
RPS2	0.186954023	8.401425943	3.233670037	0.001802399	0.010128836	-2.341133207
SAP30	0.175530574	3.256961574	3.233495921	0.001803365	0.010128965	-2.185936806
SEMA6D	-0.396780798	1.263120866	-3.233018654	0.001806015	0.01014007	-1.946763438
PTPRJ	-0.29749468	2.584967151	-3.231979577	0.001811797	0.010164959	-2.134579295
SEPT9	0.13735942	9.223786112	3.232026768	0.001811534	0.010164959	-2.288887913
INO80	-0.14402674	4.829969558	-3.231855413	0.001812489	0.010165056	-2.348637241
C14orf93	0.173948711	3.410705644	3.231671174	0.001813517	0.010167033	-2.184895341
MEMO1	-0.317007533	0.403416321	-3.23064594	0.001819244	0.010191557	-1.680959835
GLTP	0.136843982	5.391452649	3.230709992	0.001818886	0.010191557	-2.39059696
ZNF544	-0.107031081	3.804442695	-3.229639048	0.001824886	0.010215564	-2.254170994
EIF4G3	-0.167066099	6.761451485	-3.229688226	0.00182461	0.010215564	-2.411082917
PJA2	-0.343543166	7.30220264	-3.229312979	0.001826716	0.010218393	-2.396282096
FEZ1	0.151049125	6.226853836	3.229307357	0.001826748	0.010218393	-2.414806368
C19orf60	0.286193785	4.922959572	3.228699525	0.001830165	0.010233706	-2.358469546
ATG9A	0.103125361	6.66005752	3.227540644	0.001836696	0.010266414	-2.41963328
PTPDC1	0.26138184	1.85219879	3.227169115	0.001838794	0.010274332	-1.943334998
GDI2	0.078156795	8.421068241	3.226490825	0.001842631	0.010291953	-2.354543865
SMAD4	-0.192380336	5.799683149	-3.225799062	0.001846552	0.010310029	-2.422282802
ZBTB39	-0.238514824	2.511749274	-3.225372856	0.001848971	0.010319713	-2.093374478
MIR137HG	-0.33010202	0.657027117	-3.225210271	0.001849895	0.010321045	-1.737604964
NRF1	-0.185899767	3.43451821	-3.223960457	0.001857011	0.010353076	-2.22358859
C10orf76	-0.122111507	4.828947064	-3.223979457	0.001856902	0.010353076	-2.364821997
CINP	0.179454921	4.983188361	3.223594185	0.001859101	0.010359507	-2.383227208
RYBP	-0.183974971	6.500205438	-3.223517353	0.00185954	0.010359507	-2.431927775
NDUFA6	0.219331697	5.295120697	3.222814726	0.001863556	0.010378045	-2.405933987
IFRD2	0.114217815	4.966377614	3.222325613	0.001866357	0.010389802	-2.399348021
TBC1D32	-0.376764203	1.194892349	-3.222088736	0.001867715	0.01039352	-1.863298066
NDUFB6	0.155761603	4.50234064	3.221762269	0.001869588	0.010400101	-2.356492914
TNFSF9	0.341464412	2.0157642	3.219853907	0.001880572	0.010449623	-1.946861183
SYVN1	0.130136193	5.355591006	3.219877496	0.001880435	0.010449623	-2.423187913
SBDS	0.121164704	6.427652966	3.22003019	0.001879554	0.010449623	-2.441731087
PPP6R3	-0.24366099	4.896616384	-3.219675296	0.001881603	0.010451498	-2.382703957
ACSL6	-0.895682843	-3.049335262	-3.219034559	0.001885306	0.010468207	-1.364575744
RTN4	0.075357215	9.225115347	3.218316428	0.001889464	0.010485689	-2.326987712
ARL5A	-0.166168924	5.386405143	-3.218250536	0.001889846	0.010485689	-2.426011238
ACOT4	0.427689083	-0.011242697	3.217949692	0.001891591	0.010491507	-1.643198252
SAT1	0.161766261	4.677453332	3.216285391	0.001901272	0.01054132	-2.397595102
RNASEH2B	0.16330064	4.670835922	3.215428265	0.001906276	0.010564356	-2.367813464
GUK1	0.218817819	7.351078157	3.215333571	0.00190683	0.010564356	-2.439951074
PGM3	-0.135998048	5.90699653	-3.215067345	0.001908387	0.010569096	-2.454881416
LIPT2	0.5982637	-1.50355919	3.213505827	0.001917544	0.010612008	-1.487051415
TXLNG	-0.274933547	3.856445561	-3.213522854	0.001917444	0.010612008	-2.33974559
NDUFS8	0.228910907	5.666703128	3.211903289	0.001926985	0.010660337	-2.449993408
TMEM151A	0.70278225	-1.57718837	3.211415742	0.001929865	0.010664908	-1.502534187
ZNF486	-0.285782514	2.252566564	-3.211404002	0.001929935	0.010664908	-2.117394233
SLC35E4	-0.191371172	4.071682153	-3.211456345	0.001929625	0.010664908	-2.35600249
SYN1	0.356064857	0.138262675	3.21121953	0.001931026	0.010667024	-1.699445144
JRK	-0.226373278	3.203215135	-3.210674184	0.001934255	0.010677825	-2.283430727
ASB6	-0.130363522	4.515718642	-3.21064988	0.001934399	0.010677825	-2.388500512

Gene	Log fold change	Average Expression	t	P-value	Adjusted P-value	B
DOHH	0.233020555	3.96072976	3.210372413	0.001936044	0.01068299	-2.322262541
ANXA5	0.131834156	10.57060671	3.210220043	0.001936947	0.010684063	-2.272360221
CLPX	-0.140106866	4.782252345	-3.208588189	0.001946652	0.010733663	-2.435025395
STPG1	0.355699163	2.089099174	3.208393232	0.001947815	0.010736143	-1.997755077
OSCP1	0.19595559	2.228371254	3.207251083	0.001954638	0.010769811	-2.073825239
GTF2IP4	-0.265508338	1.699027323	-3.206542652	0.001958881	0.010789246	-1.968869513
FBXO27	0.258296655	2.394584571	3.206042084	0.001961885	0.010795532	-2.099578049
MCM8	-0.24553058	2.49420224	-3.205991096	0.001962191	0.010795532	-2.112190693
TMEM14B	0.190810014	4.263474655	3.205874862	0.001962889	0.010795532	-2.377609913
AZI2	-0.12296934	5.371624755	-3.206227031	0.001960775	0.010795532	-2.45551195
LOC401472	-0.480525251	-0.165907443	-3.205464685	0.001965355	0.010801205	-1.610733505
SCAF1	0.128359008	6.637841298	3.20557248	0.001964706	0.010801205	-2.482431686
FAM86JP	-0.335523884	1.290472063	-3.204675894	0.001970104	0.010823359	-1.860129756
PSMB6	0.172436264	5.998649493	3.204206482	0.001972936	0.010834964	-2.483337986
C1QL3	-0.791367629	-1.287858462	-3.203555954	0.001976866	0.010852592	-1.51126489
SIPA1L2	-0.338447166	5.484342471	-3.203068012	0.001979819	0.010864844	-2.468425759
CLK1	0.435806117	3.847084894	3.202690615	0.001982106	0.010873432	-2.260512716
MDM1	0.230381676	1.43194903	3.20248251	0.001983368	0.010875598	-1.959789562
SLC7A6	-0.372097902	2.77974605	-3.202387431	0.001983945	0.010875598	-2.220902049
GCH1	-0.367099513	2.292021016	-3.201942745	0.001986645	0.010886437	-2.060729915
OTUB1	0.167941735	5.768646269	3.201805685	0.001987478	0.010887041	-2.48436823
PIMREG	-0.373037873	1.60180412	-3.201127529	0.001991603	0.010905675	-1.941279818
SYT7	0.270885534	4.948455907	3.200767422	0.001993797	0.010911657	-2.465868643
COASY	0.155895815	5.563466061	3.200710485	0.001994144	0.010911657	-2.477935761
AASDHPPT	-0.092881406	4.728699671	-3.200564533	0.001995034	0.010912563	-2.442853196
KLHL23	-0.398540882	0.203078344	-3.20025744	0.001996908	0.010918849	-1.690671512
COPE	0.23242772	6.04951871	3.19978223	0.001999811	0.01092679	-2.495977006
BIN1	0.188218484	6.406271293	3.19989092	0.001999147	0.01092679	-2.498851424
CHID1	0.112213562	7.126021486	3.199507371	0.002001492	0.010932009	-2.491087775
BCL7A	-0.233470791	3.164140339	-3.199280621	0.00200288	0.010935624	-2.262290044
ZNF580	0.241164387	4.463951641	3.199121885	0.002003852	0.010936967	-2.407114907
CAVIN3	0.27668182	6.514018258	3.198905468	0.002005178	0.010940239	-2.502261954
MYO19	0.158432058	3.369166551	3.198188585	0.002009576	0.010956916	-2.286145387
BAIAP2-AS1	-0.114165656	4.947990367	-3.198170128	0.002009689	0.010956916	-2.456776202
CDHR2	-0.893428229	-2.402177259	-3.197838283	0.002011728	0.010964064	-1.439959225
FAM126A	-0.295626263	7.093404924	-3.196864595	0.002017722	0.010992754	-2.486076068
SMTNL2	0.998102685	-3.144419689	3.196489637	0.002020035	0.011000223	-1.435064508
COX5B	0.220164695	6.17577685	3.196405568	0.002020553	0.011000223	-2.507369976
GPAA1	0.141364303	7.312346962	3.195831254	0.002024101	0.011015557	-2.49641511
BAALC	0.246806504	5.724483045	3.194980378	0.002029368	0.011040233	-2.503835381
FAF1	-0.085160434	5.314342223	-3.194418785	0.002032852	0.01105519	-2.487647881
PPP1R16A	0.206140849	3.403161881	3.194122024	0.002034695	0.011059847	-2.297137191
KMT2A	-0.351497783	5.290177431	-3.194044511	0.002035176	0.011059847	-2.50308308
TXNL1	0.150409734	6.626479765	3.193696612	0.002037339	0.011067608	-2.516547955
ZNF575	0.300571622	2.097849521	3.191924445	0.002048391	0.01111824	-2.088719126
C5orf22	-0.163212224	3.976601455	-3.191892299	0.002048592	0.01111824	-2.397695041
POMP	0.152576221	6.432507223	3.191847206	0.002048874	0.01111824	-2.522495219
RPN1	0.070149251	8.3724184	3.19155226	0.002050719	0.011124245	-2.454916238
DENND1A	-0.137447713	5.088579226	-3.191159607	0.002053178	0.011133574	-2.473176715
DARS2	-0.15141506	3.836197016	-3.190959583	0.002054431	0.011136363	-2.369810817
C19orf66	0.190336666	4.227801013	3.190293776	0.002058609	0.011154997	-2.415796784
TRAIP	0.327188692	0.651484825	3.190140399	0.002059573	0.011156205	-1.79542561
ZNF836	-0.399516863	1.598460955	-3.189440914	0.002063973	0.01117602	-2.089138234
ZNF528	-0.187838766	3.318102633	-3.189295274	0.00206489	0.011176969	-2.325261322
RBPJ	-0.296675586	7.293550585	-3.188777539	0.002068154	0.011189308	-2.515977831
PAPSS1	0.07364312	5.943627365	3.188698025	0.002068655	0.011189308	-2.527855431
ERCC5	0.181913575	3.884994273	3.187479893	0.002076355	0.011219284	-2.394844957
PGK1	0.083889954	7.475342806	3.187467784	0.002076432	0.011219284	-2.507529565
MED13L	-0.277410497	6.535222446	-3.187645484	0.002075307	0.011219284	-2.533936482
VPS41	-0.234154225	5.90592355	-3.186906152	0.002079991	0.011234485	-2.532999394
HILPDA	-0.208636599	2.29563701	-3.186421238	0.002083068	0.011247075	-2.194841481
PIP4K2C	-0.214536983	3.26336162	-3.18542078	0.002089431	0.011277388	-2.2704633
ZNF630	0.284376597	0.455592206	3.185158167	0.002091104	0.011278337	-1.788828698
NUBP2	0.209212681	5.031238048	3.185245234	0.002090549	0.011278337	-2.490827198
CACNB3	0.126554789	4.381478004	3.184741708	0.00209376	0.01128862	-2.437405126
CHAC1	1.170720815	1.1046177	3.184165032	0.002097443	0.011303717	-1.805946696
RBM15B	-0.089177081	6.951067596	-3.184068388	0.002098061	0.011303717	-2.541962336
NPHP3	-0.267249266	1.806840297	-3.18387574	0.002099293	0.011306311	-2.023041518
DRAXIN	0.606823327	-1.241007853	3.183713975	0.002100328	0.01130732	-1.603116215
BSG	0.163495294	9.626928199	3.183413347	0.002102253	0.01130732	-2.410924274

Gene	Log fold change	Average Expression	t	P-value	Adjusted P-value	B
LNPK	-0.149210652	5.462098598	-3.183377469	0.002102483	0.01130732	-2.529327389
PDZD8	-0.304700816	5.78751405	-3.18351659	0.002101592	0.01130732	-2.539282486
SUZ12	-0.215468887	4.567375886	-3.182785681	0.002106277	0.011319643	-2.487253276
CAMSAP1	-0.216749328	4.681779917	-3.1828511	0.002105857	0.011319643	-2.498288737
S100A10	0.23863876	9.180016004	3.181760365	0.002112866	0.011349323	-2.44516026
C11orf95	-0.139584573	5.616224786	-3.181691941	0.002113307	0.011349323	-2.530872085
OCEL1	0.200557587	3.555348854	3.180061923	0.002123824	0.011401739	-2.346458125
ANLN	-0.362828198	4.326788436	-3.179503579	0.002127437	0.01141707	-2.463607149
HSPBAP1	0.168785683	2.512935066	3.179104933	0.002130021	0.011426864	-2.218117811
UQCRC2	0.082139544	6.751268197	3.178912953	0.002131266	0.011429474	-2.556369606
FAM168B	-0.138210961	7.31236823	-3.178574369	0.002133464	0.011437189	-2.541811131
KLHL8	-0.220524659	3.87931323	-3.177146891	0.002142754	0.011482902	-2.424167961
MYBL1	-0.380521603	0.815617791	-3.176343693	0.002147997	0.011506909	-1.899249381
NCR3LG1	-0.69793113	2.966133948	-3.176089907	0.002149656	0.011509707	-2.336042842
BAZ2A	-0.303286713	5.273616158	-3.176030091	0.002150048	0.011509707	-2.557155644
TSPYL2	0.161012769	4.562315945	3.175866188	0.00215112	0.011511358	-2.517173945
TRAK1	-0.139055991	6.431975182	-3.174172071	0.002162234	0.011566724	-2.572745142
RIPOR1	-0.124000485	6.619388177	-3.17386263	0.00216427	0.011573505	-2.571255691
MRPL3	0.126173287	6.303274979	3.173302333	0.002167961	0.011589128	-2.575243537
SLF2	-0.277889453	4.12250508	-3.171998406	0.002176573	0.011629426	-2.519244986
NOL8	0.128245667	5.280525483	3.171927327	0.002177043	0.011629426	-2.558336956
UNC13B	-0.160525544	5.282724054	-3.171752526	0.002178201	0.011631483	-2.550361008
ZNF225	-0.19868014	2.83235177	-3.1715189	0.002179748	0.011635622	-2.330312299
DNAJC2	-0.147952608	4.647989673	-3.171393182	0.002180581	0.011635946	-2.527556931
RHOJ	-0.220986815	4.361867655	-3.170640779	0.002185574	0.011658457	-2.477218388
SELENOM	0.224367623	6.698519317	3.170461979	0.002186762	0.011660665	-2.581638724
CENPC	0.18975269	3.020053265	3.170227887	0.002188318	0.011664835	-2.271335181
DDIAS	-0.398931907	0.583187237	-3.169925386	0.002190331	0.011671433	-1.861600918
METTL26	0.272674433	5.289208604	3.168207889	0.00220179	0.011728349	-2.554714288
GPATCH2L	-0.267404705	4.51010023	-3.167649392	0.002205529	0.011742691	-2.520975164
C1orf21	-0.121481667	6.319564399	-3.167572829	0.002206042	0.011742691	-2.590679098
MOAP1	-0.165341055	3.319403167	-3.167121032	0.002209071	0.011754662	-2.343861171
LEMD2	0.112817182	4.858318767	3.166807208	0.002211178	0.011761716	-2.532452046
TET1	-0.438871507	0.74698406	-3.166100764	0.002215926	0.011782815	-1.975717167
TRIM21	0.138160771	4.536462591	3.165551144	0.002219628	0.011794169	-2.519384507
TMEM14C	0.158497175	6.680791705	3.165656067	0.002218921	0.011794169	-2.595599595
HJURP	-0.38820695	1.620401551	-3.164814186	0.002224599	0.011812636	-2.066515782
THRB	-0.260515868	5.865648118	-3.164803516	0.002224671	0.011812636	-2.58269426
GHR	-0.385442683	3.152473356	-3.164225925	0.002228575	0.011829196	-2.331717206
CTIF	-0.143636998	6.615018612	-3.163502501	0.002233474	0.011851022	-2.60155591
R3HCC1L	0.20211377	2.601201292	3.163230706	0.002235317	0.011856626	-2.335772463
ZNF610	0.252956567	1.317509166	3.160986073	0.002250593	0.011929544	-2.166519836
CCNB2	-0.253018482	2.922017162	-3.160977946	0.002250648	0.011929544	-2.293845505
FMNL2	-0.320721697	4.528023301	-3.160794852	0.002251898	0.011931973	-2.490541192
GTF2B	0.137578587	4.085202935	3.16045154	0.002254245	0.011940205	-2.491343015
CYLD	-0.172808714	5.128623436	-3.159673063	0.002259573	0.011964222	-2.576737171
CTSW	0.897803123	-3.058447497	3.159409219	0.002261382	0.011966291	-1.520743911
PSME4	-0.27967469	5.892497415	-3.159384321	0.002261553	0.011966291	-2.612697756
UBE3A	-0.2223523	5.998880218	-3.157924853	0.002271583	0.01201514	-2.614196379
GSTO1	0.178963262	6.92707266	3.157297737	0.002275905	0.012033778	-2.614722485
SELENOF	0.078695007	7.369374199	3.155602941	0.002287625	0.012091503	-2.604942554
NDUFS6	0.237072477	5.24526055	3.154732398	0.002293667	0.012119186	-2.592913676
USP47	-0.118965605	6.004015725	-3.154345376	0.002296358	0.01212915	-2.62561924
RIN2	-0.211104756	5.501865384	-3.153750262	0.002300501	0.012146776	-2.596080516
N4BP2	-0.49958832	3.052686401	-3.153273077	0.002303829	0.01215615	-2.397975412
RABL2B	-0.121838279	3.25235256	-3.153264222	0.002303891	0.01215615	-2.403923317
GEN1	-0.256423517	2.182790133	-3.152867034	0.002306664	0.012164557	-2.236593608
STOM	0.147048425	8.150947027	3.152695514	0.002307862	0.012164557	-2.569490548
HSPBP1	0.193391717	5.067467851	3.152689153	0.002307907	0.012164557	-2.589943842
NCOA3	-0.38364197	5.69715857	-3.151980489	0.002312865	0.012186426	-2.623855136
C22orf46	0.138345625	4.417773195	3.15182691	0.00231394	0.012187831	-2.540127112
ARHGAP26	-0.249280219	4.06296594	-3.1505648	0.002322799	0.012230214	-2.534245768
B3GNT2	-0.284072704	3.423324051	-3.149686821	0.00232898	0.012258475	-2.412418133
MAFF	-0.270506954	4.856315638	-3.148942519	0.002334232	0.012281827	-2.482128156
RASGRF1	0.994229455	-4.096777116	3.148149951	0.002339837	0.012307018	-1.524665146
RPL30	0.129225514	8.579175406	3.147326369	0.002345674	0.012333415	-2.574936062
FLJ20021	0.295732352	1.914310567	3.146588544	0.002350915	0.012356659	-2.148819223
CDC14A	-0.392744277	0.478102799	-3.145918146	0.002355687	0.01237742	-1.961772088
TXNDC17	0.190768935	4.457739163	3.145375969	0.002359552	0.012391948	-2.564570137
RBL2	-0.184222173	5.512103415	-3.145299652	0.002360097	0.012391948	-2.638896109

Gene	Log fold change	Average Expression	t	P-value	Adjusted P-value	B
POLR2H	0.182125703	4.241819917	3.144377157	0.002366689	0.012415921	-2.546072789
ARL10	-0.305756712	3.942580402	-3.144534698	0.002365562	0.012415921	-2.554565884
TBK1	0.132962198	4.594493836	3.144314799	0.002367135	0.012415921	-2.581809323
GSTP1	0.218876802	7.931409587	3.143524037	0.002372801	0.012436976	-2.621100058
SGSH	0.091019725	6.229337668	3.143570322	0.002372469	0.012436976	-2.658250109
ERAL1	0.156856959	5.06824702	3.142263508	0.002381858	0.012478781	-2.614270427
MINK1	-0.092706175	6.329890778	-3.142183596	0.002382433	0.012478781	-2.66143434
TMEM106B	-0.178689746	5.896182786	-3.14191925	0.002384337	0.012484413	-2.659356665
SAP130	-0.100328249	5.391350741	-3.141636423	0.002386376	0.012490746	-2.645976645
ZNF565	-0.24191207	1.625272464	-3.141352859	0.002388421	0.01249711	-2.129024941
ATP2A2	-0.326185851	8.258141099	-3.141106258	0.002390202	0.012502083	-2.601658917
LENG8	-0.266372129	4.98386071	-3.140831041	0.00239219	0.012508139	-2.622060612
DDR1	0.16833067	3.785765114	3.140318274	0.002395899	0.012521962	-2.54536168
GNAI2	0.134431842	8.617416378	3.140235738	0.002396496	0.012521962	-2.592732561
UBE2O	-0.170924045	4.723938882	-3.138390152	0.002409892	0.01258759	-2.634423856
YIF1B	0.170499519	5.006845043	3.138198292	0.002411288	0.012590519	-2.626672818
RFWD2	0.085858407	4.866840801	3.137642159	0.002415341	0.012607309	-2.61958205
KIF5B	-0.296082117	7.16655869	-3.137521159	0.002416223	0.012607547	-2.664138293
CEBPZ	-0.125278724	5.291372375	-3.135797997	0.002428824	0.012668909	-2.657446083
ATP13A1	0.106024123	6.022900255	3.135010223	0.002434605	0.012694668	-2.680211638
HMMR	-0.384590158	1.780702259	-3.133916468	0.002442653	0.012732225	-2.162579561
ZNF581	0.15644638	5.266048167	3.133369314	0.002446689	0.012748848	-2.656404965
C12orf10	0.209610905	5.139809727	3.1325633	0.002452644	0.012775462	-2.647364822
RAB3IL1	-0.207465743	6.353346769	-3.131778364	0.002458458	0.012801315	-2.691136182
GLI3	-0.256953961	6.411526831	-3.131076543	0.002463666	0.012824002	-2.693602223
CTNNAL1	0.15827875	5.554344358	3.130921701	0.002464816	0.012825559	-2.689995154
TCEANC2	0.188674729	2.99316318	3.129397032	0.002476172	0.012879952	-2.476518207
AHSA1	0.161989957	5.946491666	3.12928881	0.00247698	0.012879952	-2.693807778
RAP1GAP2	0.531675447	-0.862009846	3.129076423	0.002478566	0.012883752	-1.846611779
FAAP24	0.224508455	1.996682434	3.128658223	0.002481692	0.012887485	-2.275632798
RRN3	-0.214577273	4.325944307	-3.128637016	0.00248185	0.012887485	-2.611864397
ANKRD52	-0.31899028	5.587835376	-3.128802712	0.002480611	0.012887485	-2.697043425
LOC100507557	-0.25403819	1.30602404	-3.128144268	0.002485538	0.012897744	-2.030890161
ATXN1L	-0.247397525	5.081723134	-3.12824634	0.002484774	0.012897744	-2.666836411
SPATC1L	0.338088904	3.805722138	3.126090248	0.002500968	0.012973339	-2.52388176
NR1H3	0.176172625	3.758234859	3.125771737	0.002503368	0.012979096	-2.532730653
PPIG	-0.097691211	5.660416213	-3.125714396	0.002503801	0.012979096	-2.697676958
ACADVL	0.140878545	6.708484023	3.124929695	0.002509725	0.01300533	-2.709394677
CSE1L	0.10003905	6.408178055	3.12408782	0.002516095	0.013033858	-2.713552177
KCNC4	0.144359068	3.064614465	3.123119019	0.002523445	0.013067436	-2.482531649
ITGB8	-0.3528761	3.16492782	-3.122822833	0.002525696	0.013074598	-2.438940531
APPL1	-0.231524948	5.622160531	-3.122613095	0.002527291	0.013078361	-2.71004658
BAK1	0.20622951	3.210743888	3.122448748	0.002528541	0.013080339	-2.527054753
ZNF274	0.235405464	2.771523423	3.121771337	0.002533702	0.013098543	-2.378101706
MED4	0.136082339	4.554677816	3.121758543	0.002533799	0.013098543	-2.650104336
EMILIN1	0.120567447	9.232627965	3.120822969	0.002540943	0.013130967	-2.605832246
ZMYM4	-0.159740359	6.024854998	-3.120658253	0.002542203	0.013132971	-2.722461533
VKORC1L1	-0.279257684	5.640139591	-3.120208825	0.002545643	0.013146233	-2.71901708
HMOX1	-0.344285778	9.028612763	-3.119214283	0.00255327	0.013181105	-2.595195002
LONRF3	-0.738983783	-2.262736016	-3.118377545	0.002559704	0.013209793	-1.703377318
NUDT8	0.277734737	2.168279954	3.117349677	0.002567628	0.013246149	-2.298576991
UTP18	-0.121477042	5.13029844	-3.117181741	0.002568925	0.013248302	-2.704203998
ZMYND19	-0.190877957	4.16589729	-3.116589494	0.002573504	0.013266677	-2.616349882
LUZP1	-0.412978263	6.533055689	-3.116493091	0.00257425	0.013266677	-2.734111551
AURKA	-0.309571625	2.123851608	-3.115591869	0.002581233	0.013298117	-2.294734701
ZNF85	-0.240708287	1.375207717	-3.115152482	0.002584644	0.013300193	-2.193283251
UPF3B	0.307639266	1.860720918	3.115117014	0.00258492	0.013300193	-2.267947748
POP4	0.154567288	4.77662865	3.115085112	0.002585168	0.013300193	-2.678736962
SORBS3	0.212198741	7.625547937	3.115269744	0.002583733	0.013300193	-2.716658393
DTD1	0.153275675	5.010243314	3.114969418	0.002586067	0.013300276	-2.695811093
PPM1E	-0.764451165	-2.028168137	-3.114541194	0.002589397	0.013312858	-1.686751675
UBASH3B	-0.292381178	3.413826286	-3.1120797	0.002608617	0.013407099	-2.587687539
BRCA2	-0.31303597	1.225138569	-3.111859011	0.002610347	0.013411414	-2.222487887
DGKZ	0.154233817	4.649499284	3.111519411	0.002613011	0.013420522	-2.679462196
FAM131C	0.546627176	-1.122613527	3.111289651	0.002614814	0.01342188	-1.82671839
MDFIC	-0.144643779	6.585123917	-3.111258754	0.002615057	0.01342188	-2.748617492
DYNC2LI1	-0.209068116	3.59506619	-3.111121394	0.002616136	0.013422844	-2.534763315
FIGN	-0.311015578	1.373045486	-3.110615114	0.002620116	0.013438689	-2.236085776
PRICKLE2	0.178647255	4.488938029	3.110184091	0.002623509	0.013451512	-2.669609283
ADNP2	-0.256865562	4.364535424	-3.109881558	0.002625893	0.013459155	-2.619366749

Gene	Log fold change	Average Expression	t	P-value	Adjusted P-value	B
AARS	-0.100508881	8.052486475	-3.109692619	0.002627383	0.013462211	-2.714155707
EBP	0.262456525	4.45335743	3.109524844	0.002628707	0.013464414	-2.675875941
SNRPG	0.217980626	3.387187411	3.107948388	0.002641175	0.013523678	-2.546692223
AP4M1	0.140747747	4.05444503	3.107231493	0.002646863	0.013527152	-2.627705136
CFL1	0.201756912	9.260252618	3.107316565	0.002646188	0.013527152	-2.645556592
DCAF8	-0.109948548	4.800691042	-3.107248986	0.002646724	0.013527152	-2.694428633
C15orf39	-0.175535283	5.695733119	-3.107371294	0.002645753	0.013527152	-2.73601278
RASAL2	-0.3041523	5.004305336	-3.10718389	0.002647241	0.013527152	-2.74251476
CCDC6	-0.14809115	5.865198053	-3.107686052	0.002643255	0.013527152	-2.756485541
DSEL	-0.280431434	6.668897407	-3.107020055	0.002648543	0.013529214	-2.75988153
DDHD1	-0.193650932	4.077206096	-3.105846047	0.002657888	0.013567749	-2.638682534
COPRS	0.198484851	6.113082845	3.105847982	0.002657873	0.013567749	-2.762373657
IKBKAP	-0.363826379	4.73915097	-3.105356163	0.002661797	0.013583098	-2.701211577
SPOUT1	-0.115389496	3.969904111	-3.104310808	0.002670155	0.013621137	-2.622440127
KCTD3	-0.117293467	5.698796096	-3.103661596	0.002675358	0.01364306	-2.758350418
RAB11B	0.164673873	6.45008476	3.102915327	0.002681351	0.013668993	-2.772468669
DDX18	-0.173419152	5.681502694	-3.102671081	0.002683315	0.013674378	-2.767821756
SHROOM3	0.302285492	4.119030279	3.102461945	0.002684998	0.013676946	-2.607193569
TIMM13	0.20746679	5.404898937	3.102382823	0.002685635	0.013676946	-2.74893716
SV2A	0.208708991	4.643727386	3.101588628	0.002692036	0.01370028	-2.712798274
FNBP4	-0.199402378	4.497839246	-3.101644554	0.002691585	0.01370028	-2.713049348
UTP15	-0.143311527	3.664211041	-3.101461015	0.002693066	0.013700892	-2.591665999
DBR1	-0.12965895	3.651182318	-3.100055481	0.002704433	0.013747352	-2.609091304
AKAP11	-0.350018368	5.877948565	-3.100007047	0.002704825	0.013747352	-2.774321783
DNTTIP2	-0.118785016	6.084594931	-3.099993418	0.002704936	0.013747352	-2.776508706
NDUFAB1	0.180779411	4.58934426	3.099828857	0.00270627	0.013749492	-2.713731241
BUB1	-0.336158164	2.280603028	-3.099449852	0.002709344	0.013760473	-2.35795979
DCBLD2	-0.267612551	6.466069193	-3.099080938	0.00271234	0.013771046	-2.781693066
DIP2A	0.210268816	4.319463277	3.098568869	0.002716504	0.013787539	-2.677357258
CCDC125	-0.232021985	2.511687493	-3.098396391	0.002717908	0.013790017	-2.369096966
ZFP3	0.216819514	1.959823545	3.096840666	0.0027306	0.01384975	-2.395465805
VPS51	0.14518029	6.821742012	3.095901674	0.002738287	0.013884066	-2.789837187
CTSD	0.159126075	9.474059248	3.095166389	0.002744321	0.013909978	-2.668003238
GPR180	-0.322448824	3.676088733	-3.094903557	0.002746481	0.013916242	-2.639809648
CPT1A	-0.258198993	5.590222817	-3.094745521	0.00274778	0.013918145	-2.782857861
TUBB6	0.161327025	8.062682417	3.094274586	0.002751656	0.013933091	-2.739364202
NDUFAF4	-0.16634642	3.206151095	-3.092740825	0.002764313	0.013987644	-2.556683763
KIF11	-0.275378453	3.535164154	-3.092766635	0.0027641	0.013987644	-2.595046368
PSMB9	0.215358907	4.013991351	3.092631771	0.002765215	0.013987644	-2.661317174
GFPT1	-0.212217339	6.436910723	-3.092398771	0.002767143	0.013992698	-2.801575402
LRCH3	-0.240218941	5.101604955	-3.091266046	0.002776535	0.014035477	-2.777882887
PUS7L	-0.22501929	3.700438311	-3.090639541	0.002781742	0.014057082	-2.631964234
PPP2R2A	-0.12463102	4.876669053	-3.090262911	0.002784877	0.014068204	-2.76041505
C5orf38	0.233700657	3.382865184	3.089899905	0.002787901	0.014078761	-2.581147667
SATB2	-0.270461736	3.051674447	-3.088817348	0.002796939	0.014119667	-2.575028404
SCX	0.537682925	-0.85473243	3.088510961	0.002799502	0.01412787	-1.920475454
MAGEE1	0.161932692	2.407857495	3.088395711	0.002800466	0.014128005	-2.46681517
TGIF2	-0.236172659	2.247703574	-3.087076231	0.002811532	0.014176447	-2.446376278
MRPS34	0.225776397	5.533484798	3.087026378	0.00281195	0.014176447	-2.796830439
C6orf89	-0.182042308	7.376734771	-3.086881356	0.002813169	0.014177846	-2.798293269
UTP3	-0.114108965	5.568817662	-3.086086034	0.002819862	0.014206825	-2.796236267
APBB1	0.104244335	5.818094618	3.085815982	0.002822138	0.014213538	-2.81020311
PSMD1	0.10470294	7.224594903	3.08552832	0.002824565	0.014221003	-2.801871243
LVRN	-0.411002856	1.277681733	-3.085326331	0.002826269	0.014224832	-2.227486264
MACROD1	0.23092097	3.642518694	3.084267102	0.002835225	0.014265141	-2.638607612
STX11	-0.701773762	-1.386777973	-3.083443639	0.002842206	0.014295489	-1.799149068
MKL1	-0.160273069	5.692887945	-3.082211098	0.002852684	0.014343403	-2.820942339
NUDC	0.175361879	6.365107232	3.080138368	0.002870387	0.014427595	-2.83564384
USP31	-0.180447546	2.433152368	-3.079967021	0.002871855	0.014430159	-2.512582196
LOC100132215	0.789901296	-1.939247579	3.079197519	0.002878456	0.014458505	-1.779297438
THAP8	0.236471827	3.635757337	3.078579434	0.002883768	0.014480361	-2.665557118
AGPAT4	0.188421872	4.109336602	3.078208687	0.002886959	0.014491553	-2.736064839
REPS1	-0.083052735	4.849108226	-3.076087658	0.002905278	0.014568942	-2.791465462
AFG3L2	-0.118384059	4.933753665	-3.076137009	0.00290485	0.014568942	-2.804239914
TRUB2	0.10247641	5.085169387	3.076264066	0.00290375	0.014568942	-2.809112522
PABPC4L	-0.451767895	-0.706390466	-3.075733726	0.002908345	0.014569772	-1.933158312
AGO4	-0.168281411	2.859436633	-3.075841675	0.002907409	0.014569772	-2.574936708
MRPS12	0.176945563	4.450247872	3.075774071	0.002907995	0.014569772	-2.765754248
CENPO	-0.191374034	2.692659138	-3.075213322	0.00291286	0.014587542	-2.557134193
IGF2BP1	-0.270442842	2.940781826	-3.075011126	0.002914616	0.014591486	-2.586201621

Gene	Log fold change	Average Expression	t	P-value	Adjusted P-value	B
SMC3	-0.119862034	5.583223219	-3.074762157	0.00291678	0.014597467	-2.838647951
ULK2	-0.149704955	4.448339808	-3.074520995	0.002918878	0.014603112	-2.75162511
PSMB10	0.208515123	3.224473978	3.073988158	0.002923517	0.014615815	-2.607553023
EIF1	0.149594451	8.906277667	3.074116847	0.002922395	0.014615815	-2.773579222
WTAP	-0.132749808	6.414560728	-3.073895112	0.002924327	0.014615815	-2.853150945
ATG4D	0.257063625	1.488687401	3.072585059	0.002935765	0.014668114	-2.296429025
PGA5	0.904377755	-3.408491666	3.071062858	0.002949107	0.014720129	-1.734566877
KLHL18	-0.24679297	3.893727947	-3.071178196	0.002948094	0.014720129	-2.735776096
ZHX1	-0.160437343	5.362812707	-3.071085847	0.002948905	0.014720129	-2.836125237
MYL12A	0.123609594	7.295952438	3.070775838	0.002951629	0.014722961	-2.842148103
AP1B1	0.09071337	5.995006169	3.070781128	0.002951583	0.014722961	-2.858796203
DYNLL1	0.201724178	7.134025524	3.070657799	0.002952667	0.014723263	-2.855940013
ZBTB46	-0.224937459	1.894438849	-3.070298729	0.002955826	0.014734138	-2.418467929
IRF2BPL	0.210956756	5.950122269	3.069599581	0.002961986	0.01475996	-2.85711591
RBM38	0.171796065	3.228707059	3.068869221	0.002968434	0.014787198	-2.677073581
ST6GALNAC6	0.104594787	7.106005849	3.068362761	0.002972913	0.014804613	-2.858309595
SHC4	0.537157872	-1.261508947	3.066861904	0.002986222	0.014851254	-1.922275801
DHTKD1	-0.193050147	3.421786634	-3.0670758	0.002984322	0.014851254	-2.646735403
FPGS	0.12253318	5.455366822	3.066963105	0.002985323	0.014851254	-2.853020726
DCLK1	-0.315675353	6.579926979	-3.066896696	0.002985913	0.014851254	-2.870748046
CXXC1	0.162821024	4.920979352	3.066611218	0.002988451	0.014857432	-2.802382125
PSMD10	0.121405799	5.035091867	3.066396146	0.002990364	0.014862038	-2.826932841
MRPL52	0.204023745	4.79200436	3.065380168	0.002999416	0.014897709	-2.811661768
NDUFB4	0.211562932	5.579987555	3.065368691	0.002999519	0.014897709	-2.859477718
SMIM4	0.275459732	2.774673316	3.064349666	0.003008624	0.014933087	-2.570808288
PFN1	0.218077042	7.975288258	3.064442135	0.003007797	0.014933087	-2.836255121
COMMD2	-0.144836995	4.936189334	-3.063966823	0.003012052	0.014945175	-2.83644175
GPR135	-0.256690884	0.559586414	-3.06344854	0.003016698	0.014956445	-2.184903439
KIF18A	-0.376675245	1.06605173	-3.063572719	0.003015584	0.014956445	-2.208674927
FANCE	-0.217801998	2.641553643	-3.063381229	0.003017302	0.014956445	-2.56627991
GPS2	0.658519705	-1.474751121	3.06249738	0.003025242	0.01499087	-1.887678282
ASNA1	0.16037311	6.525493091	3.06209798	0.003028836	0.015003745	-2.885505577
HIST3H2A	0.267781766	2.310592735	3.060779591	0.003040729	0.015057706	-2.468117858
PLD3	0.128900321	8.611185723	3.059731346	0.003050216	0.015099721	-2.816316297
SLC4A7	-0.489781805	3.934628563	-3.058959956	0.003057215	0.015124465	-2.763562251
NAA50	-0.171237834	7.297121091	-3.058959103	0.003057222	0.015124465	-2.873169886
NEURL2	0.579607559	-1.729203655	3.058540131	0.00306103	0.015133362	-1.860849119
MUTYH	0.233186651	2.239168178	3.058635021	0.003060167	0.015133362	-2.467290278
ZNF333	-0.231263858	3.011875632	-3.056815913	0.003076746	0.01520607	-2.659651437
CDK2AP2	0.228928763	4.61882228	3.056622486	0.003078514	0.015209816	-2.817712038
METTL2B	-0.146387117	3.308782423	-3.055796975	0.003086069	0.015237148	-2.687333193
FAT4	-0.419311492	5.258213049	-3.055836076	0.00308571	0.015237148	-2.87206045
ALG13	-0.120061419	4.065535617	-3.055385974	0.003089836	0.015250752	-2.789393821
CCDC36	0.34821048	0.388094421	3.055270957	0.003090892	0.015250964	-2.194519468
BFAR	0.078825399	6.394921202	3.055153058	0.003091974	0.015251307	-2.904522379
B9D2	0.266008018	1.552679943	3.054946499	0.00309387	0.015255666	-2.346079661
NEK9	-0.1802929	7.237998999	-3.054605672	0.003097002	0.01526611	-2.890683848
VSTM2L	0.250264363	2.574870925	3.054152026	0.003101174	0.015281679	-2.594142934
CTPS2	-0.152787458	3.313468486	-3.053945146	0.003103079	0.015286064	-2.665641951
ZFPL1	0.169845099	2.518879796	3.053193803	0.003110006	0.015315176	-2.52706686
PDCD5	0.201946999	5.35698653	3.05138532	0.003126737	0.015392536	-2.889875828
H3F3B	-0.12422195	7.944467256	-3.051042621	0.003129916	0.015403156	-2.88516089
SF3B5	0.127304006	6.09053585	3.050522149	0.003134752	0.015421913	-2.915065009
CCM2	0.140694263	5.767071259	3.050025651	0.00313937	0.015439594	-2.908218892
ABHD13	-0.230958201	4.122296236	-3.04981278	0.003141353	0.015444301	-2.784027067
LINC01336	0.826781708	-2.653768092	3.049021102	0.003148735	0.015475545	-1.824336334
HNRNPA1L2	0.348860273	-0.199861615	3.048443038	0.003154136	0.015497032	-2.094837596
DES	0.307466105	2.019513904	3.0481127	0.003157226	0.015502102	-2.502165167
DIMT1	-0.09023054	5.127065329	-3.04812025	0.003157155	0.015502102	-2.896840959
EPM2AIP1	-0.113929734	5.019399446	-3.047920136	0.003159028	0.015505899	-2.879674223
PREPL	-0.205300801	5.383620706	-3.047735379	0.003160759	0.015509339	-2.898924363
MRPS18B	0.150192492	5.352103989	3.047375156	0.003164135	0.01552085	-2.902219248
RGS2	-0.269670896	1.942536116	-3.04707967	0.003166907	0.015529391	-2.487060115
RNF5	0.20448704	3.540493759	3.046893782	0.003168652	0.015532892	-2.717287986
DNAJC8	0.126109113	6.531032084	3.045951446	0.003177512	0.015571257	-2.92967064
MBLAC2	-0.171688284	2.671706852	-3.045639692	0.003180448	0.015580578	-2.635756256
NRP1	-0.211564633	7.86714704	-3.045445183	0.003182282	0.01558449	-2.887342784
RUNX1	-0.289866379	7.370506412	-3.045145387	0.003185109	0.015593268	-2.92631923
PITX2	0.213803997	3.182327469	3.045003919	0.003186444	0.015594736	-2.72860433
RALB	0.091879153	6.499990368	3.044626243	0.003190011	0.015607121	-2.933293212

Gene	Log fold change	Average Expression	t	P-value	Adjusted P-value	B
NHP2	0.187403474	4.207039506	3.043917491	0.003196714	0.01563484	-2.824870741
MMAB	0.180705177	4.901683566	3.043211548	0.003203404	0.015662474	-2.879876006
LINC00565	-0.297281615	0.942016633	-3.042972973	0.003205668	0.015663374	-2.278594869
PRDX6	0.146719098	7.815454763	3.04307792	0.003204672	0.015663374	-2.901173354
SOAT1	-0.18728335	6.466205691	-3.042666098	0.003208582	0.015672529	-2.93831332
RBM45	-0.149132541	2.892165106	-3.042258637	0.003212455	0.015686361	-2.652542571
ZNF45	-0.179048268	3.296168803	-3.040359302	0.003230566	0.015769685	-2.757656866
ATP10D	-0.41113726	4.824328417	-3.040068024	0.003233352	0.015778171	-2.910477843
PARM1	0.635118101	-1.556682715	3.036876435	0.003264023	0.015922684	-1.963113123
MOCOS	-0.234966378	4.12417174	-3.03661282	0.003266569	0.015929942	-2.805350469
CRTC1	0.141452635	4.795920447	3.036252682	0.003270049	0.015941754	-2.887013055
MTUS1	-0.486237873	2.608450198	-3.034341899	0.003288573	0.016021688	-2.58379181
FOSL2	0.300494217	7.248480997	3.034415987	0.003287853	0.016021688	-2.959526512
RABAC1	0.222563666	6.766698705	3.033970747	0.003292182	0.016034087	-2.960645793
TTC31	0.157188578	3.697092878	3.033311768	0.003298599	0.016060149	-2.766887392
SHOC2	-0.138806298	6.195389656	-3.031750961	0.003313846	0.016129166	-2.964902191
DLX3	-0.567666809	0.578335009	-3.031407512	0.003317209	0.016135111	-2.235254988
ANXA2	0.140934565	9.537275773	3.031476823	0.00331653	0.016135111	-2.835316434
PHACTR2	-0.186762455	4.383484695	-3.031153233	0.003319702	0.016142022	-2.885076336
PPP4R2	-0.151507966	5.013655155	-3.030422633	0.003326872	0.01617167	-2.928617872
UNK	0.128979983	4.130567624	3.029619904	0.003334768	0.016203762	-2.865271613
KDM5B	-0.281663778	6.442522007	-3.029532753	0.003335626	0.016203762	-2.97165797
FAF2	-0.241136198	6.253241232	-3.028485868	0.003345951	0.016248681	-2.977122828
HNRNPDL	0.12162855	7.306849616	3.02833159	0.003347475	0.016250844	-2.94818933
TRAF6	-0.23159688	3.075992013	-3.027581337	0.003354896	0.01627638	-2.722138428
TMEM268	-0.14980862	3.477255988	-3.027588847	0.003354822	0.01627638	-2.765205811
ZNF706	0.137462393	5.017876886	3.027421173	0.003356483	0.016278832	-2.935890123
AFF3	-0.40942381	3.499356198	-3.025053251	0.003380015	0.016382416	-2.751973801
FH	0.140977601	5.951986842	3.025094501	0.003379604	0.016382416	-2.984098828
UNC50	0.104526756	4.338459046	3.024713348	0.003383406	0.016393575	-2.88439383
RALGPS1	-0.210623822	1.396612326	-3.024504256	0.003385493	0.016398414	-2.410501154
PLAGL2	-0.194419767	3.823042757	-3.023858405	0.003391948	0.016424398	-2.830770084
SOGA3	-0.611848631	-1.990824192	-3.023649228	0.003394041	0.016429252	-1.924050112
TRMT11	-0.139517776	3.148661232	-3.022739207	0.00340316	0.016468104	-2.754303384
VAMP3	0.073590672	7.445697395	3.021838321	0.003412211	0.016506597	-2.973334883
UBB	0.149129324	7.657959032	3.021445712	0.003416162	0.016520406	-2.970742817
LINC00517	-0.677251328	-1.514099503	-3.020647685	0.003424206	0.016553812	-1.937616067
ADCK5	0.235631786	1.932731035	3.020542518	0.003425268	0.016553812	-2.50925082
KSR2	-0.86672982	-2.97005806	-3.020279196	0.003427927	0.01656135	-1.865684928
DENND5A	-0.149717728	6.99456393	-3.019864653	0.003432117	0.016576276	-2.993243866
MTG1	0.389579571	-0.422084697	3.019684741	0.003433936	0.016578483	-2.108618983
PRR11	-0.312609264	2.944184579	-3.019601919	0.003434775	0.016578483	-2.714573985
C1orf122	0.212144317	5.217959147	3.018722623	0.003443684	0.016616161	-2.971133011
PDRG1	0.186481123	2.584883623	3.018544394	0.003445492	0.016619564	-2.678057752
PABPC4	0.134104683	8.668545728	3.017724814	0.00345382	0.0166544	-2.928614127
VHL	-0.292131267	4.491115182	-3.017334155	0.003457796	0.016668237	-2.928381804
RAB11FIP2	-0.206127075	4.861563552	-3.016938122	0.003461831	0.01668235	-2.953052189
KDELR1	0.12160724	8.391929352	3.016406757	0.003467252	0.016703129	-2.947569314
AFF4	-0.415868853	6.712496149	-3.015954577	0.003471871	0.016720034	-3.009542921
ESRRA	0.146777503	3.765314423	3.015724789	0.003474221	0.016726002	-2.883178107
GALNT18	0.459470608	-1.140273082	3.013532332	0.003496712	0.016828906	-2.06114182
SOCS5	-0.278199744	7.134241271	-3.013076153	0.003501409	0.016846128	-3.004042903
CEBPD	0.362023444	5.811572083	3.012557613	0.003506755	0.016862173	-3.001021656
MDH1	0.093993993	6.580344457	3.012535549	0.003506983	0.016862173	-3.020312069
ZNF304	-0.183360253	3.5065892	-3.012290627	0.003509511	0.016868943	-2.842144346
ZBTB7A	-0.138003173	6.778198068	-3.011975602	0.003512764	0.016879197	-3.019704809
AP4B1	0.170182946	2.725771101	3.010332074	0.003529785	0.016955577	-2.721455204
C17orf97	0.291607636	1.192324686	3.009815767	0.003535148	0.016975924	-2.458045151
LOC100131315	-0.342006427	1.188092122	-3.009414514	0.003539321	0.016990547	-2.435417548
NOP53-AS1	-0.660416167	-2.216378921	-3.009146844	0.003542108	0.016997997	-1.963272599
RFX7	0.334269012	4.621602837	3.008972286	0.003543926	0.016997997	-3.003533767
EPG5	-0.312468399	5.233286024	-3.008940364	0.003544258	0.016997997	-3.012984835
KAZALD1	0.178766708	4.758885464	3.008831461	0.003545393	0.016998028	-2.976334756
ILKAP	0.170339919	3.930917836	3.008370063	0.003550205	0.017015682	-2.904940267
RILP	0.242629953	3.234898733	3.00738641	0.003560484	0.017059519	-2.790408452
PEX14	0.172429691	3.954841805	3.0057802	0.003577327	0.017133935	-2.89014742
IFIT1	0.187237582	4.839778056	3.005688576	0.00357829	0.017133935	-2.979146194
LMAN2	0.111546675	7.138068809	3.005431315	0.003580995	0.01714144	-3.029991936
EBF3	-0.267703633	2.839311784	-3.005280962	0.003582577	0.017143565	-2.775811664
DENND4A	-0.246270828	4.348926299	-3.004477077	0.003591046	0.017178634	-2.958606343

Gene	Log fold change	Average Expression	t	P-value	Adjusted P-value	B
LINC02361	-0.893071544	-3.15911232	-3.003458334	0.003601805	0.017220531	-1.90453375
FUCA2	0.096641672	6.180426224	3.003431378	0.003602091	0.017220531	-3.044479844
KIF21A	0.375678744	-0.063609245	3.001848214	0.003618871	0.017295266	-2.344352552
ARL4A	0.376718214	2.033279854	3.001334871	0.003624328	0.017304875	-2.497867694
CYB5RL	-0.364594147	2.209646715	-3.001534288	0.003622207	0.017304875	-2.643587014
TSPAN14	-0.162796309	5.552408422	-3.001378341	0.003623866	0.017304875	-3.032503085
ICA1	-0.95855773	-3.493203843	-2.999770365	0.003641006	0.017378338	-1.893927647
LACC1	-0.193646625	3.97253754	-2.999632844	0.003642475	0.017378338	-2.91815416
BCAR1	-0.201785923	6.234415133	-2.99956734	0.003643175	0.017378338	-3.053732604
BRD8	0.129307119	4.753636132	2.999053317	0.003648673	0.017399054	-3.01201647
BEND5	0.467484848	-0.415516448	2.998756379	0.003651853	0.017406575	-2.162382041
MRPL12	0.265263111	2.881370395	2.998690226	0.003652561	0.017406575	-2.773189309
LAGE3	0.22282386	3.931347766	2.998331968	0.003656402	0.017413859	-2.90743711
MRPS11	0.146584905	4.228346631	2.998354382	0.003656161	0.017413859	-2.943530813
SPHK2	0.136168612	3.92285909	2.998093466	0.00365896	0.017420537	-2.914875157
PNPT1	-0.136841804	4.003010283	-2.997906515	0.003660967	0.017424584	-2.928155077
SMIM8	-0.211897244	1.722345176	-2.997305482	0.003667426	0.01744981	-2.520286794
CLCN3	-0.224783357	6.391881101	-2.996849275	0.003672335	0.017467652	-3.063564566
RBM23	0.072332433	5.705491777	2.996079932	0.003680628	0.017501571	-3.054894355
CCBE1	-0.249566237	6.125474041	-2.995621826	0.003685574	0.01751956	-3.066394536
SH2D5	-0.286446798	0.982303741	-2.994449522	0.00369826	0.017574316	-2.552423705
G6PC3	0.182320422	5.92118242	2.994132856	0.003701693	0.017585085	-3.064979913
DCAF1	-0.246927104	4.62230697	-2.993829498	0.003704985	0.017595176	-3.01668615
SKAP2	-0.189523208	5.268826529	-2.993630124	0.00370715	0.017599909	-3.046893
B4GALT2	0.123794629	7.165713686	2.992850562	0.003715627	0.017634596	-3.06296039
STOML1	-0.161532097	3.962772903	-2.992132854	0.003723447	0.017666145	-2.918453224
SLC16A6	-0.835135192	-2.646874378	-2.991175002	0.003733908	0.017704624	-1.973958166
PLEKHM1	-0.09518617	5.4942437	-2.991230317	0.003733303	0.017704624	-3.060634904
ZNF200	-0.157750499	2.57223761	-2.990618143	0.003740002	0.017727938	-2.766487907
TJP1	-0.214678462	6.104721058	-2.99016833	0.003744931	0.017745719	-3.0807604
FAM207A	0.214561174	2.694020372	2.989355697	0.003753851	0.017782395	-2.766538371
OXA1L	0.139527206	7.301810593	2.988487009	0.003763409	0.017822065	-3.071566861
SSBP2	-0.120218808	4.384750437	-2.987784101	0.003771159	0.017853154	-2.947875939
PPP2R1B	-0.15836664	4.035900846	-2.987378578	0.003775637	0.017868737	-2.975607579
ZNF611	-0.320429282	1.57365603	-2.986972659	0.003780124	0.017884355	-2.59892073
THAP3	0.18091926	3.063095242	2.986432238	0.003786105	0.017907031	-2.865874979
FUBP1	-0.126028143	4.945938243	-2.985726823	0.003793927	0.017932761	-3.061292524
ARPC1B	0.159053847	7.195327395	2.985829832	0.003792784	0.017932761	-3.081657181
TSACC	1.011505621	-3.312923599	2.985491129	0.003796543	0.0179395	-1.93799442
ZNF271P	-0.081729039	5.283592734	-2.984718354	0.003805134	0.017974454	-3.068019752
LRRC8D	-0.17576271	3.795165132	-2.984269497	0.003810132	0.017992421	-2.927626263
BRMS1L	0.109903927	3.461705376	2.984097791	0.003812045	0.017995816	-2.912589898
FANCG	0.14765943	3.719013258	2.983657467	0.003816956	0.018013356	-2.917388699
FKBP11	0.193232239	4.678925631	2.98301055	0.003824183	0.018041806	-3.02909299
NUDT11	0.101295923	3.682270677	2.981962709	0.003835914	0.018091486	-2.953286223
AFF1	-0.346851628	4.747683154	-2.980731548	0.00384974	0.018151013	-3.036774046
PQBP1	0.223585407	5.425430869	2.980494188	0.003852411	0.018157922	-3.082124624
SERPINA5	0.728220602	-2.593247029	2.980147515	0.003856315	0.018170638	-1.998160648
UBQLN2	-0.084164572	6.227759362	-2.980023561	0.003857712	0.018171536	-3.108858436
DCTN3	0.160904861	5.357606069	2.979883524	0.003859291	0.018173289	-3.083218599
BHLHE22	-0.544477457	-0.093730697	-2.979435302	0.003864347	0.018191415	-2.255475226
SLC25A44	-0.207631261	5.241383375	-2.978903884	0.00387035	0.018213983	-3.08052639
PHPT1	0.214810458	5.898789705	2.978669176	0.003873005	0.018220781	-3.104942522
NDUFAF8	0.242692366	4.410149802	2.977984608	0.003880756	0.018251546	-3.020062495
XIRP1	-0.663840007	-0.855075934	-2.977793481	0.003882922	0.018256036	-2.156944156
ENGASE	0.217455302	2.209327188	2.977327363	0.003888211	0.018270922	-2.715143526
PSMB5	0.132317116	6.55828956	2.977300644	0.003888514	0.018270922	-3.116010248
TTC17	-0.163419108	5.724350638	-2.977068732	0.003891148	0.018277597	-3.104178337
CDC42EP1	-0.201147684	6.684426529	-2.97620108	0.003901017	0.018318242	-3.11875477
ZNF432	-0.208749582	2.274554503	-2.975885171	0.003904616	0.018323718	-2.77180269
NOP53	0.204445082	8.505795059	2.975985967	0.003903467	0.018323718	-3.05283666
UBE2Z	-0.083084835	6.738731405	-2.974474945	0.00392072	0.01839356	-3.121421529
OCIAD1	0.093579758	6.333449809	2.974117335	0.003924813	0.018407031	-3.125024232
LRCH2	-0.282524481	2.249260565	-2.972920466	0.003938542	0.018465668	-2.680513743
MSANTD3	-0.091462778	5.643247655	-2.972811299	0.003939796	0.018465802	-3.115837082
ACVR1C	0.616701739	-1.251778051	2.972393606	0.003944599	0.018482564	-2.109037918
NEK7	-0.282971631	6.550585388	-2.971504244	0.003954844	0.018519144	-3.126323238
PPP1CC	0.070016284	6.780655583	2.971502421	0.003954865	0.018519144	-3.128398591
RAB9B	-0.264213504	0.7852346	-2.971032164	0.003960292	0.018527279	-2.464416322
ZBTB37	-0.233352862	2.286955537	-2.971179694	0.003958589	0.018527279	-2.767727418

Gene	Log fold change	Average Expression	t	P-value	Adjusted P-value	B
EAPP	0.106061535	4.824822442	2.971057341	0.003960001	0.018527279	-3.073236034
POR	0.127544807	6.620584154	2.970446605	0.003967059	0.018553176	-3.134256835
IFI6	0.217841623	5.483341641	2.969982881	0.003972426	0.01857251	-3.11517176
PTGS2	0.626390908	2.583827388	2.969855433	0.003973902	0.018573647	-2.733191699
GOT2	0.095821581	5.850446217	2.969676403	0.003975977	0.018577579	-3.129058915
IKZF4	-0.16210915	2.713697658	-2.969281434	0.003980557	0.018581689	-2.819707983
PRPF39	0.177618503	3.247166238	2.969330569	0.003979987	0.018581689	-2.947305175
TMED5	-0.177047485	5.854946392	-2.969307412	0.003980256	0.018581689	-3.132957013
SYNJ2BP	-0.164096229	4.053917993	-2.968745115	0.003986784	0.018601751	-3.004979002
KIF13A	-0.18309242	6.371284462	-2.96869862	0.003987324	0.018601751	-3.139204476
AATBC	0.926318389	-2.984673416	2.968104098	0.003994239	0.018628239	-2.016264791
SRRM1	-0.206203128	5.307009885	-2.96757254	0.00400043	0.01865134	-3.121743404
ID4	-0.361605603	3.327427716	-2.967351794	0.004003004	0.018657566	-2.753610811
TBL1X	-0.139010658	5.878280617	-2.967016383	0.004006918	0.01867003	-3.13811601
PCTP	-0.127101314	2.682131371	-2.966546226	0.00401241	0.018684061	-2.898027552
ENO1	0.137639994	9.952647571	2.966579611	0.004012019	0.018684061	-2.98249645
TPST1	-0.103344017	5.469395438	-2.966336426	0.004014863	0.018689706	-3.116435528
C9orf142	0.243451031	3.801320897	2.966037007	0.004018366	0.018700235	-2.976516381
SEC14L2	0.183466644	2.301861406	2.965870133	0.004020319	0.018703548	-2.791168743
RARA-AS1	-0.358669227	-0.565213073	-2.965008847	0.004030416	0.018739735	-2.254291776
ZNF215	-0.301597905	0.689767743	-2.964994439	0.004030586	0.018739735	-2.461081003
KIAA1524	-0.259800596	2.209132728	-2.964873894	0.004032001	0.01874053	-2.739131047
RUVBL2	0.160981388	5.954084984	2.96383231	0.004044247	0.018791654	-3.14772321
PHB2	0.119995964	7.600168507	2.963522135	0.004047901	0.018802831	-3.128161649
C11orf54	-0.223145719	4.140439788	-2.963121517	0.004052624	0.018818969	-3.035629519
TRIM66	-0.3544542	1.334224702	-2.962888683	0.004055372	0.018825924	-2.547004208
KIF1C	0.105475806	7.700806025	2.962766238	0.004056818	0.018826833	-3.123680805
CHCHD2	0.178151442	6.374600965	2.962625795	0.004058476	0.01882873	-3.155667018
TBC1D10A	-0.256093096	3.008107458	-2.962270007	0.004062681	0.018839201	-2.888338926
SRFBP1	-0.215417713	3.284320713	-2.96222321	0.004063234	0.018839201	-2.919850298
PLEKHF2	0.206179172	3.218555619	2.962022986	0.004065603	0.018844382	-2.954853903
LOC100507156	-0.846207661	-2.857566301	-2.96162034	0.004070369	0.018855066	-2.037590018
SPAST	-0.294505937	3.88659037	-2.961616809	0.004070411	0.018855066	-3.01870439
MRPL1	0.144016329	4.500614804	2.961411609	0.004072842	0.018860528	-3.066703905
AQP3	0.331184789	0.970927904	2.960957475	0.004078228	0.018879663	-2.520903276
SNX22	0.67039415	-1.877114631	2.960674849	0.004081583	0.018889389	-2.090764559
ZNF141	-0.298564368	1.543141822	-2.959827118	0.004091661	0.018930214	-2.672433562
KAT2B	-0.187184045	4.178412729	-2.959324497	0.004097647	0.018952089	-3.041223975
INCA1	-0.249315437	0.63798366	-2.958719356	0.004104865	0.018979645	-2.458593435
RPLP2	0.224905678	9.431971875	2.958279054	0.004110124	0.018998131	-3.047605149
PTPN3	0.685785023	-2.791365657	2.958167627	0.004111456	0.018998458	-2.078168556
ZNF317	-0.144154906	5.033866321	-2.957958287	0.004113959	0.019004196	-3.127385674
YTHDF1	-0.099698967	5.584664389	-2.957438587	0.00412018	0.019027099	-3.157010409
SEC61G	0.108319776	5.112313751	2.956423531	0.004132356	0.019077478	-3.14084295
CAPN5	0.131061608	5.82510044	2.955621192	0.004142004	0.019110304	-3.168954719
PFKM	-0.087160968	5.982296008	-2.955711839	0.004140913	0.019110304	-3.170344038
NRAV	0.197458903	2.256918932	2.955376147	0.004144954	0.01911221	-2.777520353
ELMSAN1	-0.28016209	4.077134731	-2.955425221	0.004144363	0.01911221	-3.025971016
ZSWIM1	-0.231302318	2.715969509	-2.955103305	0.004148242	0.019115667	-2.895644915
STMN3	0.16406282	5.267413734	2.955190626	0.004147189	0.019115667	-3.147771624
CYTH3	-0.160721244	6.091493826	-2.954291696	0.004158036	0.019154939	-3.177292136
MPHOSPH8	-0.193653809	5.147377378	-2.953962794	0.004162011	0.01916739	-3.140113126
PAIP2	0.080870469	4.596712723	2.95384647	0.004163418	0.019168008	-3.113427209
SNHG7	0.245796123	4.710643947	2.953252015	0.004170613	0.019195269	-3.104470866
IZUMO4	0.523279862	-1.203796924	2.952927857	0.004174542	0.019201618	-2.179679614
DYNC2H1	-0.260345631	5.390360281	-2.952951873	0.004174251	0.019201618	-3.162613896
NBEAL1	-0.454562921	2.227153232	-2.952253771	0.004182722	0.019233373	-2.803787577
ESPL1	0.271387345	1.293888101	2.951699111	0.004189465	0.019258497	-2.684716474
KANK3	0.793660294	-4.44364303	2.951357516	0.004193622	0.01927139	-2.005097037
ATP7A	-0.423891192	3.842874305	-2.951258485	0.004194828	0.01927139	-3.041714295
WWC1	0.294606149	1.205063327	2.950441304	0.004204792	0.019299507	-2.683271977
C14orf2	0.186147118	5.719266409	2.950611484	0.004202715	0.019299507	-3.175811005
MRAS	-0.130143587	6.095586284	-2.950496359	0.00420412	0.019299507	-3.1871552
NPDC1	0.19257324	6.196191707	2.950196053	0.004207786	0.019307368	-3.186494823
KLHL2	-0.166288119	3.258042699	-2.949218125	0.004219746	0.019356351	-2.976829969
C1QBP	0.167884854	6.028495869	2.9475336	0.004240422	0.019439354	-3.194227898
PRDX4	0.123997115	6.623623549	2.947540959	0.004240331	0.019439354	-3.195222988
TPRG1	-0.310028586	0.751669721	-2.946903456	0.00424818	0.019460915	-2.460374145
DLG1	-0.151709704	5.616546403	-2.946836861	0.004249001	0.019460915	-3.183788958
PSMB4	0.170235617	7.200529837	2.94687596	0.004248519	0.019460915	-3.186560349

Gene	Log fold change	Average Expression	t	P-value	Adjusted P-value	B
RORB	0.831437465	-2.553213369	2.945628026	0.004263924	0.019523327	-2.087893428
AMACR	0.251864831	1.191093028	2.945307715	0.004267886	0.019523666	-2.609028628
IL27RA	0.346664358	1.690024939	2.94543114	0.004266359	0.019523666	-2.726160925
KIF20A	-0.314263816	3.429055651	-2.945390919	0.004266856	0.019523666	-2.956524503
FAM214A	0.22054731	3.816023579	2.944981383	0.004271926	0.019536215	-3.040958815
RSPO3	-0.38581799	5.770080018	-2.944542214	0.004277369	0.01955517	-3.178459468
TBC1D8B	-0.220029875	4.104027406	-2.942272464	0.004305601	0.019674592	-3.103634165
PRKCI	-0.166895915	4.603567934	-2.942232261	0.004306103	0.019674592	-3.145159352
CDC14B	-0.206342846	5.582746181	-2.942084452	0.004307947	0.019677053	-3.200609341
KCCAT198	-0.814077069	-3.252864253	-2.941517413	0.004315031	0.019697462	-2.045730426
NCOA6	-0.165707327	5.350914944	-2.941586276	0.00431417	0.019697462	-3.199391817
PLEKHG1	-0.35250777	4.577222543	-2.941386175	0.004316672	0.019698984	-3.12333964
ATOX1	0.173135301	5.108728754	2.939465747	0.004340751	0.019802867	-3.176495658
EXOC2	0.115236858	4.851660866	2.939115371	0.004345157	0.019816968	-3.161422387
VCP	0.082518663	8.752860766	2.9386283	0.00435129	0.01983893	-3.124014983
USP28	0.17025667	4.548020771	2.938202308	0.00435666	0.019857404	-3.133723588
HEG1	-0.4610481	8.25907164	-2.937815475	0.004361541	0.019873641	-3.150516426
GLOD4	0.111799108	5.317713316	2.937501974	0.004365501	0.01988567	-3.198390046
VPS13C	-0.344000702	5.285139556	-2.935699298	0.004388336	0.019983644	-3.207775039
PKIG	0.212773007	7.035308689	2.935479122	0.004391133	0.019990336	-3.219091248
KCTD20	-0.148644869	6.640818084	-2.93528051	0.004393657	0.019995784	-3.227574974
BAP1	0.067273446	6.297394016	2.934686814	0.00440121	0.020024109	-3.230532581
LUCAT1	-0.929650383	-4.245620305	-2.933754732	0.004413092	0.020072107	-2.0495291
EIF3A	-0.143988975	8.725321525	-2.932458485	0.004429666	0.020141409	-3.14261104
MKRN1	0.064317405	6.539805385	2.932099215	0.00443427	0.020156258	-3.237179652
SLC6A17	0.512802372	-0.265040207	2.931850076	0.004437465	0.020163184	-2.446999117
PTAR1	-0.209117587	4.667948221	-2.931771708	0.00443847	0.020163184	-3.184245203
BHLHB9	-0.190585849	2.906496659	-2.931125886	0.004446765	0.020194773	-2.955543947
NUDT3	-0.136240447	4.210213457	-2.9305981	0.004453553	0.020219508	-3.131915286
DDOST	0.093687588	7.916032235	2.930183016	0.004458899	0.02023768	-3.200250087
ARFGEF2	-0.353015654	4.938953593	-2.929927425	0.004462194	0.020246534	-3.210721511
ELMOD2	-0.367514319	4.398708077	-2.929238113	0.004471091	0.020280794	-3.15942696
ATP5F1	0.090685191	6.345903187	2.928461789	0.004481131	0.020320214	-3.247079947
NKRF	-0.199623424	3.010242763	-2.92793352	0.004487974	0.020345123	-2.980550529
G3BP1	-0.190521781	7.633784607	-2.927554607	0.004492889	0.020361275	-3.214958278
DCBLD1	-0.153494803	3.310697961	-2.927208079	0.004497388	0.020375534	-3.036013369
MERTK	0.580396005	-1.703056415	2.926108804	0.004511687	0.020428031	-2.219109915
BTF3	0.170517954	8.823500493	2.926127493	0.004511444	0.020428031	-3.164805086
NDUFAF3	0.208111744	5.34076011	2.925424802	0.004520606	0.020462263	-3.226350171
IMPG2	-1.063047151	-3.806124442	-2.924822339	0.004528475	0.020479421	-2.071899719
FAM86EP	-0.216382246	1.167811093	-2.924987474	0.004526317	0.020479421	-2.625640797
CYB5B	0.10619266	6.31030687	2.924851077	0.0045281	0.020479421	-3.256599644
RHBDD2	0.132388082	5.932854676	2.924564792	0.004531843	0.0204885	-3.252533609
GPR1	-0.279915027	4.476630301	-2.924396012	0.004534051	0.020492334	-3.165721219
ZNF555	-0.172017266	2.23977075	-2.923913968	0.004540364	0.020514709	-2.902841497
WDFY3-AS2	-0.157304088	3.416083068	-2.923207311	0.004549632	0.020538106	-3.035714124
FSTL3	0.262125358	3.656414614	2.923271863	0.004548785	0.020538106	-3.066755201
DIS3	-0.160139449	5.900230872	-2.923230117	0.004549333	0.020538106	-3.254462962
STK19	0.154249835	2.9072418	2.922676048	0.004556612	0.020563451	-2.977608339
EIF2AK1	0.091519804	6.725602059	2.922213595	0.004562695	0.020584739	-3.262127489
LDHA	0.090218009	9.37778389	2.922082491	0.004564421	0.02058636	-3.133188477
TMCC3	-0.510765807	-0.551564232	-2.921794865	0.00456821	0.020597282	-2.410881602
ZNF813	-0.309098831	1.603538229	-2.921200093	0.004576054	0.020614139	-2.794894842
NIT2	0.155799331	4.431776026	2.921280191	0.004574997	0.020614139	-3.172033328
SNX3	0.10399441	7.966557562	2.92137999	0.00457368	0.020614139	-3.223268886
LEF1	0.714336104	-1.962263569	2.920702935	0.00458262	0.020637547	-2.221833314
RNH1	0.156757571	9.060639546	2.91960549	0.004597146	0.020696773	-3.16754799
ERCC3	0.105843229	4.696984115	2.917646585	0.004623179	0.020807757	-3.193385532
TXNDC2	0.725500992	-2.698853085	2.916757999	0.004635032	0.020854876	-2.153259945
POMGNT2	0.113336575	4.595536218	2.916634918	0.004636676	0.020856044	-3.195285402
PSMD13	0.148592351	6.62059653	2.915897285	0.004646541	0.020894177	-3.27948732
CCDC170	-0.320121476	0.952401718	-2.915407628	0.0046531	0.020917426	-2.624193055
DMXL2	-0.417305021	2.666867166	-2.915107567	0.004657123	0.020923026	-2.938770543
C19orf70	0.228894154	4.980700247	2.915190648	0.004656009	0.020923026	-3.230446196
CELF1	-0.161681381	5.049632998	-2.914911419	0.004659755	0.020928609	-3.240370841
VPS13B	-0.372171108	5.431807747	-2.914546878	0.00466465	0.020944349	-3.267405833
RCN1	0.073622383	8.296496259	2.913072906	0.004684491	0.021027168	-3.223637953
PNPLA2	0.177427876	5.713519845	2.912787944	0.004688336	0.021038157	-3.273762461
ST7-AS1	-0.613476432	-1.282096205	-2.912461131	0.004692749	0.021051689	-2.197975688
C11orf68	-0.115416543	6.153473379	-2.912329471	0.004694528	0.0210534	-3.288247364

Gene	Log fold change	Average Expression	t	P-value	Adjusted P-value	B
ATP11B	-0.233730882	5.811238478	-2.910894069	0.004713963	0.021134267	-3.28921807
FAM185A	0.282251719	0.419941729	2.910725095	0.004716256	0.021138255	-2.503234945
CNOT6L	-0.242321486	4.255809879	-2.910175291	0.004723723	0.021165425	-3.233111998
ATL2	-0.185019855	4.202463624	-2.909403286	0.004734226	0.02120618	-3.193618356
ODF2	0.114387375	4.906544959	2.908861962	0.004741604	0.021232913	-3.254745885
HAUS1	0.185314601	4.14120394	2.908602088	0.004745149	0.021242476	-3.166521045
VGLL3	-0.220780065	8.705675528	-2.907589395	0.00475899	0.021298104	-3.230082698
DUSP14	-0.199677131	6.059343135	-2.906785351	0.004770005	0.021341061	-3.294577605
ADH1A	-0.772084588	-2.090475002	-2.905988277	0.004780948	0.021377321	-2.2083909
PYCR1	0.150869074	5.722282244	2.906023897	0.004780458	0.021377321	-3.293373518
TYMP	0.178083036	4.571372209	2.905625857	0.004785931	0.021386906	-3.224580573
CDK2AP1	0.110455768	6.686936176	2.905628117	0.0047859	0.021386906	-3.305471764
PEX16	0.177182886	3.856514691	2.904892886	0.004796024	0.021425653	-3.151646617
AKAP13	0.295712198	6.256427256	2.904549979	0.004800752	0.021440418	-3.309787119
OSBPL1A	-0.139769185	6.146241702	-2.903572285	0.004814258	0.021491027	-3.312076654
PUM1	-0.244091112	6.099072642	-2.903523163	0.004814937	0.021491027	-3.313019515
AMMECR1	-0.213504324	3.927771864	-2.90337566	0.004816978	0.021493767	-3.189907858
RNFT1	-0.136656112	2.307757228	-2.902583835	0.004827948	0.021536335	-2.920975409
KCNK1	0.238761826	1.994933992	2.902126864	0.004834289	0.021558237	-2.896095843
NTNG1	-0.207342681	4.683321521	-2.901883669	0.004837666	0.021566915	-3.247693699
EIF3D	0.096454286	8.343652241	2.901351473	0.004845066	0.021593512	-3.256207643
FAM110A	0.232444245	1.758564553	2.899739835	0.004867537	0.021674423	-2.835899377
ASAP1	-0.273468673	6.552989534	-2.899807316	0.004866594	0.021674423	-3.319744292
RBBP7	0.096639718	6.399495608	2.899874498	0.004865655	0.021674423	-3.322575005
CEBPZOS	-0.089582959	4.674417434	-2.898840751	0.004880114	0.021724008	-3.250862243
SENP3	0.139097215	3.644186593	2.898234865	0.004888607	0.021755385	-3.14534267
UBLCP1	-0.086534884	5.446547923	-2.897632261	0.004897068	0.0217866	-3.31334026
GPN1	0.094984577	4.697416776	2.896963369	0.004906474	0.021815599	-3.266017453
SRPRB	0.098968534	6.229750362	2.896962816	0.004906482	0.021815599	-3.330474953
LETM2	-0.255821981	0.504808091	-2.896731855	0.004909734	0.021823616	-2.630864768
FNTA	0.149486776	4.87015007	2.896417069	0.00491417	0.021836888	-3.274013419
SON	-0.397741494	7.625609719	-2.895983546	0.004920284	0.021857611	-3.295286884
PPP1R2	-0.130866588	5.165773092	-2.895811255	0.004922716	0.021861968	-3.303084435
USP36	-0.17295915	5.900659722	-2.895372494	0.004928914	0.021883044	-3.325158331
SDC1	0.207424928	5.370480067	2.895079374	0.004933059	0.021894993	-3.313562896
MIER3	-0.248310369	4.136575363	-2.894885279	0.004935806	0.02190073	-3.232202883
PROKR1	-0.166873554	2.010559965	-2.894537837	0.004940725	0.021916104	-2.853526776
ZNF558	-0.125076112	3.259449283	-2.894184875	0.004945728	0.021931836	-3.117442804
FOCAD	-0.157916053	5.334760988	-2.893008404	0.004962436	0.021999451	-3.313189789
PIF1	0.349910999	0.638267223	2.892713404	0.004966633	0.022005108	-2.6141066
CCDC15	0.332161958	0.375727893	2.892740152	0.004966253	0.022005108	-2.641706999
KHSRP	0.075701197	7.868583538	2.892168009	0.004974403	0.02203305	-3.301209171
PRPF3	0.122823532	4.518464293	2.891905159	0.004978151	0.022043171	-3.272977986
ZNF334	0.326284221	0.726150413	2.891309543	0.004986654	0.022074335	-2.66540724
TTC27	-0.103895457	3.921013615	-2.890969159	0.00499152	0.022083982	-3.199733489
ERCC1	0.159130643	6.160546629	2.890951964	0.004991766	0.022083982	-3.34439163
PEX12	0.149475917	3.17643657	2.890714881	0.004995157	0.022085527	-3.149854346
ZNF134	-0.149303144	4.346256079	-2.890517725	0.004997979	0.022085527	-3.247676935
USP38	-0.199923658	4.825458031	-2.890566401	0.004997283	0.022085527	-3.290039721
ECHS1	0.179483305	6.240628215	2.890610337	0.004996654	0.022085527	-3.345487805
MROH6	0.247497474	2.608380162	2.890369516	0.005000102	0.022088427	-2.966796745
C3orf80	-0.462180692	0.13575132	-2.890005206	0.005005323	0.022105007	-2.572793902
RIOX2	-0.10660656	4.168157167	-2.889902772	0.005006791	0.022105013	-3.222834599
SLC12A6	-0.262510343	4.809504904	-2.88929484	0.005015517	0.022130563	-3.279610837
STK4	-0.194419741	4.612949241	-2.889390526	0.005014142	0.022130563	-3.284228721
RCC1L	0.112558327	5.631202416	2.888772727	0.005023021	0.022157187	-3.340077858
MBNL1-AS1	-0.143348954	2.690370695	-2.888095618	0.00503277	0.022186557	-3.036184964
NFAT5	-0.369036607	4.764752784	-2.88808144	0.005032974	0.022186557	-3.301523162
NR2C2	-0.345070097	4.905265936	-2.887926491	0.005035207	0.022186557	-3.315064261
PHLDA1	0.289723421	5.933007737	2.887901256	0.005035571	0.022186557	-3.347795942
TGFBR3L	0.545547192	-1.317975754	2.887223439	0.005045352	0.022223151	-2.329657337
ATP2B1-AS1	-0.235611806	1.248914542	-2.887109162	0.005047002	0.022223924	-2.775304623
N4BP2L2	-0.115967649	4.854686877	-2.886913932	0.005049824	0.022229849	-3.288672441
GAPDH	0.161935925	10.43978028	2.886466641	0.005056293	0.022245325	-3.171151894
ZNF84	-0.143899968	4.125316642	-2.886563456	0.005054892	0.022245325	-3.248137698
ZNF540	0.315532275	-0.293585751	2.885891674	0.00506462	0.022275455	-2.598793744
GPR162	-0.298331885	1.585861495	-2.885044101	0.005076918	0.022323027	-2.747667051
ICE1	-0.237561917	5.573652395	-2.884679952	0.00508221	0.022339776	-3.353565973
ATP13A2	0.154043942	4.036931067	2.88447491	0.005085192	0.022346363	-3.264713684
SLC39A11	-0.103382774	3.810860505	-2.884180602	0.005089475	0.022356653	-3.198643372

Gene	Log fold change	Average Expression	t	P-value	Adjusted P-value	B
TTPAL	-0.17417216	4.831675496	-2.88400819	0.005091986	0.022356653	-3.318431705
EIF3M	0.102436413	7.275374653	2.8840977	0.005090682	0.022356653	-3.347740151
WDR78	-0.196838478	1.516422753	-2.883635246	0.005097421	0.022373994	-2.773303808
IL17RA	-0.161663435	5.362533683	-2.881332101	0.005131103	0.022508717	-3.349601162
DDX41	0.092321955	5.643280065	2.881381053	0.005130385	0.022508717	-3.358360698
SETD5	-0.223410896	5.509727411	-2.880988779	0.005136141	0.02252426	-3.362687707
COX19	0.29653161	1.794097413	2.880640279	0.00514126	0.022527035	-2.946240866
OFD1	-0.139940889	3.738678204	-2.880785455	0.005139127	0.022527035	-3.193189808
SDCCAG3	0.132680269	4.696351761	2.880692279	0.005140496	0.022527035	-3.288967103
NUMA1	0.101236841	7.466547827	2.879976827	0.005151019	0.022563229	-3.357498995
ATF4	0.181635934	7.770699085	2.878942974	0.005166259	0.022622732	-3.350273287
TMEM167A	-0.134329501	6.753730493	-2.878851714	0.005167606	0.022622732	-3.374723469
AMOTL2	0.524905895	6.437816275	2.878077599	0.005179048	0.022666233	-3.354110724
PRRC1	-0.23251211	6.584779433	-2.877808496	0.00518303	0.022677075	-3.377885372
MDFI	0.6162182	-1.067412876	2.877308955	0.005190431	0.022702862	-2.409543684
ETS2	0.231225812	4.99814084	2.876536312	0.005201897	0.022746409	-3.311224288
MTHFR	-0.221769724	4.93323832	-2.876059988	0.005208978	0.02277076	-3.362784252
FAM84A	-0.371569916	1.890130797	-2.875566615	0.005216321	0.022796244	-2.901102108
DIP2C	-0.225183219	5.445180597	-2.874908009	0.005226138	0.022832523	-3.352886628
FHOD3	-0.180660956	3.765445685	-2.874663411	0.005229788	0.022841846	-3.201954288
DSTYK	-0.251879279	5.349707729	-2.874128297	0.005237782	0.02287013	-3.375475803
LPP-AS2	-0.180600146	1.913592699	-2.873622684	0.005245346	0.022895295	-2.932662819
PATZ1	-0.092090576	4.269901727	-2.873539904	0.005246585	0.022895295	-3.29615035
RIPOR3	0.22565984	3.815470904	2.872851548	0.005256901	0.022927028	-3.185255813
SESTD1	-0.136805276	5.870253667	-2.872930819	0.005255712	0.022927028	-3.385956113
ENTPD3-AS1	-0.353036118	0.144587824	-2.872671583	0.005259601	0.022932165	-2.560107047
ERCC6L	-0.379549664	0.655590292	-2.872525203	0.005261798	0.022935106	-2.690186393
TMEM91	0.277738245	1.146389799	2.871120928	0.005282919	0.023020506	-2.776237024
GRAMD1A	0.137502169	5.456938896	2.870534434	0.005291763	0.023052377	-3.373837187
ARHGEF2	-0.199208697	6.47624324	-2.869865858	0.005301862	0.023089692	-3.39828513
VDAC2	0.122554682	4.693772039	2.868668729	0.005319988	0.023155245	-3.336013415
RTL6	-0.200516596	6.29816355	-2.868744304	0.005318842	0.023155245	-3.404259983
PSEN2	-0.151193585	4.212161039	-2.868535146	0.005322014	0.023157373	-3.27922198
ATG5	0.08472397	4.81530257	2.867552408	0.005336942	0.023215623	-3.343160711
RIPK1	0.182950423	5.697796634	2.867105185	0.005343749	0.023238519	-3.398000816
SLC25A39	0.160871539	6.381535485	2.866859619	0.005347489	0.023248075	-3.408999418
SOCS2	0.234662057	3.663194408	2.866419137	0.005354205	0.023270556	-3.200141222
CEP85L	0.203612968	3.077160616	2.865721243	0.005364861	0.023310145	-3.176628725
CNKSR2	-0.362824613	0.912245861	-2.865618758	0.005366428	0.023310228	-2.751756588
MRGPRE	0.62337448	-2.119420587	2.865195028	0.005372909	0.023325885	-2.336510068
PANK3	-0.218958082	5.811925513	-2.865180641	0.005373129	0.023325885	-3.408005848
MORN1	-0.248929171	1.503572648	-2.864787679	0.005379147	0.023331836	-2.819671422
S100A11	0.208864655	8.851937739	2.864857739	0.005378073	0.023331836	-3.325742129
RBM26	0.096990198	4.777061123	2.864791817	0.005379083	0.023331836	-3.362404024
TXN	0.196250298	7.37124409	2.864348629	0.005385877	0.023354304	-3.398504077
CAMK1D	-0.250389748	4.313738754	-2.864222802	0.005387807	0.023355951	-3.301019002
TMEM147	0.169612585	6.265085951	2.863853051	0.005393483	0.02337383	-3.416175431
ARL1	0.062905649	6.941014357	2.863563714	0.005397929	0.023386367	-3.409416199
SSH2	-0.283480861	3.92427788	-2.86308563	0.005405281	0.02340476	-3.322471721
MRPL4	0.175682069	4.638527687	2.863160495	0.005404129	0.02340476	-3.343207058
ERH	0.150084326	5.70302957	2.862364847	0.005416384	0.023446095	-3.411339343
AIFM1	0.123141449	4.798807638	2.861865994	0.005424081	0.023472666	-3.357702288
TDO2	-0.66403635	-1.339990106	-2.861654974	0.005427339	0.023472987	-2.349451401
NAA20	0.158221135	5.115102506	2.861756739	0.005425768	0.023472987	-3.390699644
TRIM22	0.091740746	6.847950027	2.861457685	0.005430388	0.023472987	-3.417445569
GOLGA5	0.076541543	6.028088836	2.861531263	0.005429251	0.023472987	-3.420947525
KIAA1147	-0.199296223	2.790028244	-2.861118995	0.005435624	0.023488883	-3.121410467
TNPO3	0.077544517	5.984627984	2.860035222	0.005452413	0.023554673	-3.422883981
XRCC4	-0.163363547	2.219044321	-2.859500256	0.005460717	0.023577026	-3.006323299
CRABP2	0.235272386	7.29646842	2.859570696	0.005459623	0.023577026	-3.413226012
TCF19	-0.20476952	3.726010138	-2.858908074	0.005469923	0.023610008	-3.251352853
HEATR3	-0.18687331	3.43733902	-2.858639929	0.005474097	0.023620311	-3.248497112
CDC42EP5	0.220546547	5.141516244	2.858553247	0.005475447	0.023620311	-3.389010933
METTL2A	-0.121504068	3.05540141	-2.858452565	0.005477015	0.023620312	-3.167330799
TMEM44	0.17915051	3.784954949	2.857735851	0.005488189	0.02366173	-3.253645025
ZFP36L1	-0.214733803	8.824299119	-2.856363143	0.005509651	0.023747462	-3.346381651
EPHA2	0.340069981	3.036133609	2.85618829	0.00551239	0.023752472	-3.11158147
TRAPPC10	-0.287620567	3.980474417	-2.855143165	0.005528789	0.023809514	-3.324927101
HARS	0.138333531	6.17949831	2.855219267	0.005527593	0.023809514	-3.438215816
SSBP1	0.137126763	5.760696793	2.854931753	0.005532112	0.023817014	-3.430991117

Gene	Log fold change	Average Expression	t	P-value	Adjusted P-value	B
VPS18	-0.11436733	6.276373165	-2.85460935	0.005537183	0.023832034	-3.440338544
NIPA1	-0.205336829	3.143569113	-2.854346259	0.005541324	0.023843044	-3.220625507
SH3GL1	0.15291681	7.603290905	2.853730354	0.005551029	0.023877984	-3.415372452
TMEM259	0.110256988	5.722453887	2.852908356	0.005564006	0.023926974	-3.432920787
CHCHD10	0.250576044	3.14746856	2.85198724	0.005578582	0.023982806	-3.183866155
ABCB6	0.30447578	0.952966416	2.851655969	0.005583832	0.023984843	-2.760545472
AIFM2	-0.125934952	4.673544803	-2.851662743	0.005583725	0.023984843	-3.373772534
SMS	0.152241247	4.975190031	2.851771484	0.005582001	0.023984843	-3.396044118
SLC7A2	-0.560813437	0.106953881	-2.851347632	0.005588723	0.02399217	-2.642735565
SCAF11	-0.268397496	6.16565328	-2.851418082	0.005587605	0.02399217	-3.449189602
UNG	-0.117208563	5.244097847	-2.850937982	0.005595227	0.024013249	-3.420640821
BMPR2	-0.245318442	6.952287229	-2.850366633	0.005604311	0.024045382	-3.443875285
TMEM218	-0.142507358	3.409886254	-2.849629969	0.005616042	0.024088855	-3.198161134
SLC25A21-AS1	0.836417617	-2.715134087	2.8482736	0.005637701	0.024154248	-2.332598277
UPRT	0.119677399	2.958363393	2.848366004	0.005636223	0.024154248	-3.171498766
UBOX5	-0.145206332	3.005105491	-2.848501783	0.005634052	0.024154248	-3.214461915
FAM96B	0.193705061	4.921856601	2.848335138	0.005636717	0.024154248	-3.402125013
ANKRD31	0.926829841	-3.111475376	2.847456481	0.005650787	0.024195177	-2.274712598
SREK1IP1	-0.125348939	4.292814023	-2.847376252	0.005652073	0.024195177	-3.354930814
OXR1	-0.163805074	4.942810817	-2.847535151	0.005649526	0.024195177	-3.408370272
MT2A	0.206537051	7.278274126	2.847144447	0.005655791	0.024204215	-3.445531767
ETFDH	-0.104721874	4.081484844	-2.846606249	0.005664432	0.02423431	-3.32980137
C2orf16	-0.315422525	0.618952352	-2.846403459	0.005667691	0.024235696	-2.734229895
CAPNS1	0.174775536	8.841498896	2.846385883	0.005667974	0.024235696	-3.37529268
SRRD	-0.101834823	3.653666443	-2.846039004	0.005673553	0.024246984	-3.305191267
TBC1D15	-0.139294348	5.440811118	-2.846021582	0.005673833	0.024246984	-3.445284063
ADAM15	0.111624343	6.794138426	2.845806	0.005677303	0.024254932	-3.4609393
MFN1	-0.176127665	4.520996319	-2.845693691	0.005679111	0.024255779	-3.39275945
IER2	0.325686115	4.53734825	2.845442097	0.005683165	0.024266211	-3.320915308
MYO3B	-0.51663414	-0.451701148	-2.843779629	0.005710014	0.024373946	-2.557016392
KIFC2	0.200626016	1.611897978	2.842741631	0.005726838	0.024438834	-2.952381139
FAAP20	0.196946149	5.326379448	2.841866178	0.005741062	0.024492596	-3.444677304
BID	0.180728278	4.026561563	2.841159779	0.005752563	0.024534715	-3.355202648
RPS19	0.216462166	10.24515926	2.840934706	0.005756232	0.024543084	-3.304447306
GNB4	-0.283628352	5.500905296	-2.840839541	0.005757784	0.024543084	-3.467959996
PANK4	0.118079158	3.655700487	2.840255034	0.005767325	0.024576797	-3.309219251
NUDT22	0.116710301	3.867987636	2.840129412	0.005769377	0.02457859	-3.323452618
PPOX	-0.233353007	2.30898531	-2.839687392	0.005776604	0.024600264	-3.029933632
PFDN6	0.245619817	3.834962844	2.839618524	0.005777731	0.024600264	-3.310157639
RGS7	-0.510488523	0.019011368	-2.839440361	0.005780646	0.024605724	-2.592936912
GTF2H2C	0.339507659	0.190803927	2.838314933	0.005799096	0.024675414	-2.666631307
IFITM1	0.192712332	5.584891936	2.838241928	0.005800295	0.024675414	-3.465701942
LOC93622	0.199735456	2.769980272	2.838097086	0.005802674	0.024678565	-3.182295796
RAPH1	-0.845003937	2.601282191	-2.837824505	0.005807153	0.024684369	-2.996564321
DNAJB14	-0.301344746	4.701364579	-2.8378146	0.005807316	0.024684369	-3.435881779
IDI1	-0.24346872	6.579023238	-2.837617672	0.005810554	0.024691166	-3.478327275
TP53BP2	-0.156434947	4.943234056	-2.837465036	0.005813065	0.02469487	-3.427068994
NEK6	-0.184497647	6.443261634	-2.836645112	0.00582657	0.024737627	-3.486911127
GSK3B	-0.174717361	6.256663097	-2.836601555	0.005827288	0.024737627	-3.487056671
ABCE1	-0.109739749	6.066220783	-2.836555003	0.005828056	0.024737627	-3.488213733
KITLG	-0.249985291	6.688324879	-2.836454148	0.00582972	0.024737718	-3.486522595
ENG	0.159534763	8.266123938	2.836000059	0.005837215	0.024762549	-3.429249308
CAB39	-0.173999217	6.069025499	-2.833860458	0.005872653	0.024905869	-3.49462406
AGAP1	-0.123167618	5.089503073	-2.833610354	0.005876808	0.024916476	-3.450040468
CAMKMT	-0.275103791	1.58657358	-2.833383472	0.00588058	0.024925453	-2.916640865
DDX39A	0.176535165	5.048583919	2.832836518	0.005889682	0.02495701	-3.467686428
COL9A3	-0.819051331	-2.495732972	-2.832340815	0.005897942	0.024984985	-2.350105433
PRKACA	0.104050223	6.882452079	2.831161763	0.005917633	0.02506135	-3.497816122
NOX4	-0.428637313	0.957379716	-2.830863234	0.005922628	0.025070544	-2.791450085
TADA2B	-0.112037652	4.859989616	-2.830833099	0.005923132	0.025070544	-3.447893267
EXOC5	-0.177298583	5.915082213	-2.83064465	0.005926288	0.025076853	-3.498882803
PSMA1	0.171615956	4.094487126	2.829931634	0.00593824	0.025120375	-3.38302558
SDHAF1	0.155896406	2.887721719	2.829264929	0.005949437	0.025160673	-3.239092494
BCAP31	0.109319922	7.036365919	2.828131893	0.005968509	0.025234248	-3.502696078
CHCHD3	-0.082076193	5.136820208	-2.827753324	0.005974894	0.025254155	-3.47089871
HMGN1	0.114748836	5.38469997	2.82752866	0.005978687	0.025263095	-3.488328314
PYCARD	0.270059342	3.415327133	2.825858061	0.006006954	0.025375424	-3.293501119
SIX1	-0.279542322	3.822617765	-2.82524665	0.006017331	0.02541213	-3.390960659
TJAP1	0.110522086	4.288261181	2.82481361	0.00602469	0.025436078	-3.417945226
TAF10	0.20433896	5.883407703	2.824706335	0.006026514	0.025436651	-3.510685784

Gene	Log fold change	Average Expression	t	P-value	Adjusted P-value	B
USP42	-0.16893963	3.788652755	-2.824013969	0.006038301	0.025464976	-3.368990631
RSBN1L	-0.196725467	3.75563	-2.82409748	0.006036878	0.025464976	-3.399847408
RPRD1A	-0.153181046	4.774367815	-2.82401	0.006038368	0.025464976	-3.458425141
MRPL10	0.114934905	5.599940968	2.823815852	0.006041677	0.025464976	-3.506171003
SIK2	-0.246973231	5.742859194	-2.823908112	0.006040104	0.025464976	-3.51578205
NOP16	-0.239349846	3.354261287	-2.822446168	0.006065068	0.025556416	-3.341622844
SLC35E2B	-0.304260432	5.131864479	-2.821120047	0.006087795	0.025645006	-3.484055873
UBALD2	0.25624432	3.832810347	2.820893808	0.00609168	0.025654197	-3.349160892
SLC35F2	-0.242772987	3.556395561	-2.820324802	0.006101461	0.025688207	-3.345651614
CYB5R4	-0.138560288	4.086529414	-2.819753096	0.006111303	0.025715269	-3.402453014
PPIB	0.165079304	8.083671446	2.8197642	0.006111111	0.025715269	-3.48278595
LOC101929066	0.696164699	-2.104518617	2.819613129	0.006113715	0.025718234	-2.407908015
RNF170	-0.234097315	4.07287573	-2.819206057	0.006120734	0.025740573	-3.400074803
ARID1A	-0.276350046	5.638625079	-2.81850055	0.006132916	0.025777987	-3.526116818
SRI	0.112483941	5.733420806	2.81849267	0.006133053	0.025777987	-3.526233992
LSM3	0.118484814	5.013581439	2.818149702	0.006138983	0.025792841	-3.491136283
PDLIM4	0.197486504	5.831899888	2.818090282	0.006140011	0.025792841	-3.53112384
MRPS31	0.212604459	3.071299946	2.817886819	0.006143532	0.025800439	-3.249217183
KCTD15	-0.156885359	5.134996637	-2.816497655	0.006167624	0.025894394	-3.504790506
ZNF689	-0.121328619	3.648618044	-2.81567203	0.006181983	0.025937498	-3.392019078
COPS4	0.064590837	5.587945508	2.815610429	0.006183056	0.025937498	-3.531096353
ATP5G2	0.175535332	7.203107952	2.815789199	0.006179944	0.025937498	-3.532659513
SFXN5	0.12067618	3.533403252	2.81337415	0.00622211	0.026086798	-3.371231981
C3orf38	-0.12163318	4.664330641	-2.813437222	0.006221005	0.026086798	-3.47708847
HSPB6	0.288081471	7.674919531	2.81311851	0.006226589	0.026098313	-3.5241602
VSTM4	-0.243292571	7.094897495	-2.812709369	0.006233763	0.026121116	-3.5400619
MACROD2	-0.294129113	0.947053842	-2.81246775	0.006238004	0.026131615	-2.93184321
AMY2B	0.770125531	-2.691175561	2.812310506	0.006240765	0.026135913	-2.428831445
TIMM8B	0.193394919	3.874235516	2.811948436	0.006247127	0.026155285	-3.402288109
IL1RAP	-0.281609212	4.009022428	-2.811651827	0.006252343	0.02616985	-3.441679841
BET1L	0.098152406	5.874189111	2.811433857	0.006256178	0.026178631	-3.548858818
ZNF763	0.538028899	-1.848791333	2.810718954	0.006268774	0.026212016	-2.549091657
CHAF1A	-0.157121741	3.247205117	-2.810684776	0.006269377	0.026212016	-3.308323099
DRG2	0.09877506	4.643740385	2.810740716	0.00626839	0.026212016	-3.48007804
PPP1R7	0.154595345	6.258035399	2.809750224	0.006285879	0.026273718	-3.556190353
PTPMT1	0.162587124	2.592626041	2.809091593	0.006297533	0.026307542	-3.217838458
ANKS6	-0.13352258	4.995605902	-2.808996884	0.00629921	0.026307542	-3.519341058
SSFA2	-0.34760013	5.489776292	-2.809079973	0.006297739	0.026307542	-3.541211325
PALM	0.205729564	6.405529363	2.808802015	0.006302663	0.026314666	-3.55301056
NOSIP	0.195976188	5.267122378	2.808676697	0.006304884	0.026316647	-3.526745557
TSR3	0.20273968	5.418128291	2.808450972	0.006308887	0.02632606	-3.534104195
ENKD1	0.21874181	3.768302727	2.80770007	0.00632222	0.026368314	-3.364927588
AKR7A2	0.155172608	6.128587101	2.807683522	0.006322514	0.026368314	-3.559783444
ACTR8	-0.123507058	4.781040357	-2.807578637	0.006324378	0.02636879	-3.514140392
LDLR	-0.287620611	7.938518811	-2.807153651	0.006331938	0.026393004	-3.526859208
TRIM69	-0.090177628	5.014696874	-2.806521812	0.006343193	0.026432602	-3.510660507
DDX10	0.147085075	4.152316312	2.805277288	0.006365415	0.026517866	-3.479642492
MCRIP2	0.246545205	2.868635134	2.805068869	0.006369143	0.026522575	-3.257640854
ATF2	-0.173211624	4.957529127	-2.805017283	0.006370066	0.026522575	-3.541468983
PFKL	0.121144313	7.142088459	2.804043136	0.006387522	0.026587904	-3.561900318
EIF3F	0.121797619	7.790316461	2.803846276	0.006391054	0.02659526	-3.538956884
ZFP28	-0.145847403	2.917647505	-2.803685521	0.006393941	0.026599914	-3.297152693
MAEA	0.07169052	5.621563409	2.803587327	0.006395704	0.026599914	-3.559144528
ZNF106	-0.181424712	7.481087086	-2.803408994	0.006398908	0.026605893	-3.551633747
ORMDL2	0.167405839	2.872197102	2.802683549	0.006411957	0.026652792	-3.274474887
TSPY26P	0.161398303	2.697019713	2.802464678	0.006415899	0.02666182	-3.29844107
DEF6	0.722737562	-1.998400111	2.801958447	0.006425024	0.026670962	-2.46295202
MIR29A	-0.329013427	0.827839892	-2.801949767	0.006425181	0.026670962	-2.863819014
KATNB1	0.128903451	3.941512189	2.802091375	0.006422627	0.026670962	-3.418387295
TUFM	0.143034281	7.17279622	2.802086025	0.006422723	0.026670962	-3.566835508
ELP4	-0.156772695	3.077515289	-2.801427527	0.006434607	0.026697067	-3.273789882
RPS19BP1	0.199432972	5.131095065	2.801382085	0.006435428	0.026697067	-3.53803109
PXN	-0.099076862	7.064224426	-2.801306906	0.006436786	0.026697067	-3.568093888
MAT2B	0.083850839	5.557644376	2.799907967	0.006462108	0.026794714	-3.571449053
SDCCAG8	-0.109758844	4.288020671	-2.799369414	0.006471881	0.026827851	-3.446522835
TMEM88	0.940940611	-3.269148091	2.798774593	0.00648269	0.026865266	-2.373646813
PCNX1	-0.332400511	6.076003003	-2.798533686	0.006487072	0.026876034	-3.583684906
PGAP1	-0.289305322	4.067193341	-2.798152897	0.006494005	0.026897359	-3.472721294
MRPL20	0.134039101	5.093474331	2.797993533	0.006496909	0.026901988	-3.551495595
IFT172	-0.106278548	4.487125798	-2.796095853	0.006531574	0.027038097	-3.489404868

Gene	Log fold change	Average Expression	t	P-value	Adjusted P-value	B
ZNF277	0.100906555	5.172078645	2.795790576	0.006537167	0.027053813	-3.555183136
RRAGB	-0.090944297	4.177897338	-2.795312229	0.006545939	0.027082675	-3.482446083
MYCBP2	-0.301407657	6.513224095	-2.79496534	0.006552306	0.027098114	-3.593903981
SRRT	0.11723174	6.235927944	2.794912981	0.006553268	0.027098114	-3.595052377
STEAP2	-0.21056755	3.691509044	-2.794357963	0.00656347	0.027132283	-3.420297568
HIGD2A	0.204550037	5.300951801	2.794267521	0.006565134	0.027132283	-3.564347237
PRPF31	0.160016715	4.764164944	2.794163865	0.006567041	0.027132722	-3.533635883
CLDN12	-0.213996608	3.903166571	-2.793853916	0.006572747	0.027148852	-3.433231627
GTF3C5	0.117302427	5.239322757	2.793187751	0.006585027	0.027192118	-3.563090183
HABP4	0.113586518	5.437903198	2.792921522	0.00658994	0.02720495	-3.587508454
SNU13	0.132849311	6.548085555	2.792405789	0.006599468	0.027236819	-3.601419444
CASP9	-0.157720152	3.125271164	-2.792206257	0.006603158	0.027237122	-3.299861562
RGS3	-0.273558408	5.908003305	-2.792207474	0.006603135	0.027237122	-3.58532624
RAB8B	-0.167793776	6.316743594	-2.792033295	0.006606358	0.027242861	-3.602768484
SRGAP2C	0.325336566	1.157714945	2.791791027	0.006610842	0.027253892	-2.966926572
RASSF8	-0.249684975	6.827635528	-2.791650626	0.006613442	0.027257152	-3.601984475
ZC3H18	0.096239044	5.702953738	2.791069922	0.006624206	0.027294048	-3.597432632
NAGLU	0.106867763	6.405673184	2.789373811	0.006655737	0.027416469	-3.609034304
LINC02185	-0.96337094	-4.283389401	-2.788517101	0.006671716	0.027474777	-2.376593253
ACTR3	-0.14138658	8.462119387	-2.787227192	0.006695841	0.027566589	-3.53908599
DECR1	0.119916607	5.447411457	2.786767614	0.006704456	0.027594514	-3.593903929
MTMR12	-0.245297601	4.23272024	-2.786661955	0.006706438	0.027595132	-3.517751891
FAM212A	-0.297354229	2.452264684	-2.786340783	0.006712466	0.027612393	-3.263642083
PDCD6IP	-0.085813164	7.247319922	-2.785806087	0.006722512	0.027646171	-3.601091387
KDSR	-0.167032718	7.14808933	-2.785398529	0.006730179	0.027670147	-3.608560657
MYEF2	-0.576542691	-1.275007911	-2.784556119	0.006746052	0.027727838	-2.579062553
RASSF3	0.193991795	3.394557802	2.784422444	0.006748574	0.027730637	-3.414115844
XAB2	0.116698372	5.632140206	2.783713287	0.006761967	0.027778094	-3.611824906
ATF6	-0.135987466	5.586779268	-2.783412121	0.006767662	0.027793911	-3.612631559
PRKCSH	0.107699987	9.039245585	2.783056873	0.006774386	0.027813941	-3.525077237
B3GALT6	0.145523819	5.105195245	2.782823884	0.006778799	0.027824476	-3.583453788
GUCY1A2	-0.29513689	2.990899235	-2.78206722	0.006793149	0.027875781	-3.401898643
BYSL	-0.139449857	4.018675171	-2.78122573	0.00680914	0.027933792	-3.479655648
HELQ	0.124944135	3.243677384	2.780616068	0.006820747	0.027973792	-3.39896207
LINC01139	-0.629009292	-0.980576515	-2.779988	0.006832724	0.028015284	-2.646500574
HS1BP3	0.125520304	5.737655643	2.779810512	0.006836112	0.028021548	-3.621087755
CCAR1	-0.120074614	5.45224619	-2.779469255	0.00684263	0.028040638	-3.616060271
SLC2A13	-0.283105382	3.20582873	-2.779261646	0.006846599	0.02804927	-3.413929597
AURKAIP1	0.245090452	5.700118425	2.779076942	0.006850131	0.028056111	-3.621301367
RIC8B	0.13560398	3.217850105	2.778686538	0.006857603	0.028071448	-3.424862455
PLPPR2	0.100783469	6.067270219	2.778731917	0.006856734	0.028071448	-3.634550766
COPS7B	0.076684056	4.122414883	2.778463961	0.006861866	0.028081268	-3.52330938
FIBP	0.164122066	6.382580365	2.778083114	0.006869166	0.028103509	-3.637884032
ARMCX4	-0.225680793	3.570159601	-2.77752012	0.006879971	0.028140071	-3.484553988
PTGES	0.164807271	6.745037846	2.776680126	0.00689612	0.028198468	-3.634754466
PPP1CA	0.12387113	6.29985332	2.776366388	0.006902161	0.02821551	-3.642261981
PPT2	0.243269009	1.829503479	2.775812467	0.006912838	0.02823617	-3.153748614
CELF2	-0.258791068	7.382551826	-2.77600559	0.006909114	0.02823617	-3.621934948
RPS15	0.218914051	7.036262667	2.775899139	0.006911167	0.02823617	-3.638093802
TMCC2	0.227942747	1.883116238	2.775421815	0.006920377	0.028259302	-3.233386233
CUL4B	-0.194899034	6.399474625	-2.775143098	0.006925761	0.028273622	-3.645639664
IL17RB	-0.273140174	1.141775596	-2.774911298	0.006930241	0.028284246	-3.072779791
SLC22A17	0.130164373	4.718208905	2.774641155	0.006935466	0.028297903	-3.580094096
C1QL1	0.44437822	-0.258242857	2.774337908	0.006941335	0.028306516	-2.750095325
GMIP	0.157060612	2.603977769	2.774369487	0.006940724	0.028306516	-3.326783235
PVR	-0.202055423	4.440538993	-2.774154062	0.006944896	0.028313371	-3.560432634
SLC25A3	0.090711393	8.863387244	2.773937849	0.006949085	0.028322786	-3.554411784
LIG4	-0.255871397	2.907945246	-2.773619679	0.006955254	0.028340262	-3.302523506
C9orf78	0.084600486	5.714113873	2.772265797	0.006981562	0.028439764	-3.643770025
LY6K	0.507024487	0.031177946	2.771393597	0.006998558	0.028493588	-2.823817361
MAP3K20	-0.24446418	7.127471267	-2.771476748	0.006996936	0.028493588	-3.640630065
FAM227B	-0.26787642	0.559563788	-2.771026643	0.00700572	0.028499639	-2.912000668
ERMAP	-0.198920965	3.776265157	-2.771090026	0.007004482	0.028499639	-3.499114682
SAMD1	0.171820616	5.505014943	2.771038774	0.007005483	0.028499639	-3.639099273
SVBP	-0.12551956	3.661662149	-2.77058629	0.007014323	0.028526934	-3.474155518
LOC105369748	0.399371415	-0.97168445	2.768976413	0.007045858	0.028647452	-2.677744699
HEXIM2	0.267720638	0.39297475	2.768433658	0.007056519	0.028683057	-2.940090639
ACLY	0.173884423	7.882078012	2.768085258	0.007063371	0.02870316	-3.609963167
FAU	0.169963616	7.507508177	2.767866974	0.007067666	0.02871287	-3.643853946
SEMA4D	-0.268560258	0.320379108	-2.767600215	0.007072919	0.02871872	-2.940961132

Gene	Log fold change	Average Expression	t	P-value	Adjusted P-value	B
HMGN2	0.131532592	5.098821765	2.76768659	0.007071218	0.02871872	-3.626315392
FAM13B	-0.209910589	5.065322747	-2.767486367	0.007075162	0.028720086	-3.620882927
MBNL3	-0.21245243	3.62583882	-2.766705164	0.00709057	0.028774876	-3.496116734
TMEM47	-0.16161562	6.93463932	-2.766288587	0.007098798	0.028800512	-3.657980293
MEX3B	0.274600896	2.657906876	2.765982533	0.00710485	0.028814558	-3.351625998
APEX1	0.099922637	7.234170656	2.765920019	0.007106086	0.028814558	-3.656066149
SLC25A38	0.081498256	5.070807248	2.765206063	0.007120223	0.028864111	-3.632141221
SNRPF	0.199889596	4.225220963	2.764257975	0.007139035	0.028931874	-3.560488686
RPL10A	0.142699558	8.91972648	2.764073516	0.0071427	0.028931874	-3.580685383
NAA30	-0.196383549	4.909718706	-2.764136117	0.007141456	0.028931874	-3.63369848
ZSCAN12	-0.282012134	1.517281409	-2.762536328	0.007173313	0.029048061	-3.187314105
NAIF1	0.146667647	2.903628999	2.761643454	0.007191149	0.029112462	-3.395372877
CYB5R3	0.137923429	9.444733958	2.761342067	0.007197179	0.02912122	-3.552818277
PNPLA8	-0.131634271	5.183917698	-2.761413158	0.007195756	0.02912122	-3.65524373
ZNF407	-0.186182204	4.171562389	-2.760943671	0.007205157	0.029145671	-3.610675345
ENO2	0.161392638	4.872701056	2.760504783	0.007213954	0.029173426	-3.621696551
FIZ1	0.132801337	3.938184887	2.760317964	0.007217702	0.029180749	-3.528605224
WAPL	-0.201386586	5.414829092	-2.760011541	0.007223854	0.029197781	-3.669267138
AP2S1	0.181207062	6.791222923	2.759896116	0.007226172	0.029199317	-3.682219585
COQ4	0.183416751	4.669615471	2.759585042	0.007232423	0.02921674	-3.612657376
REXO2	0.119764015	6.440334066	2.759428348	0.007235574	0.029221632	-3.685289563
FAM20C	0.123011687	7.493109861	2.758794101	0.00724834	0.029265344	-3.663919874
RAB28	-0.152740789	3.699108249	-2.758536571	0.00725353	0.02927845	-3.502340903
STX3	-0.170008073	3.958574505	-2.758118879	0.007261954	0.029288909	-3.547064676
VEGFB	0.216315733	8.316121145	2.758185307	0.007260614	0.029288909	-3.633543655
HMOX2	0.135698492	5.193981922	2.758298114	0.007258338	0.029288909	-3.655630932
DEPDC4	-0.597675318	-1.805826823	-2.757957975	0.007265202	0.029294164	-2.59518259
CCDC159	0.181222673	2.901897637	2.757755978	0.00726928	0.029302767	-3.406993126
RPL39L	0.215102428	2.474555006	2.757381204	0.007276854	0.029317605	-3.283284147
COPS9	0.191714488	4.939191945	2.757404462	0.007276383	0.029317605	-3.637673658
CFAP69	-0.189359039	1.854568087	-2.756622013	0.007292217	0.029371646	-3.190492588
TSEN34	0.128155932	5.475525547	2.756397301	0.00729677	0.029382129	-3.674777098
GAREM1	0.208853176	2.395899547	2.755611853	0.007312705	0.029438426	-3.409592158
POC5	0.202883622	1.519071588	2.755467026	0.007315647	0.0294424	-3.182876501
EPB41	-0.35805301	3.024574455	-2.754993881	0.007325265	0.029457498	-3.412486992
PARK7	0.164016659	7.434063586	2.755061649	0.007323886	0.029457498	-3.678260294
MEPCE	0.116104053	5.992783684	2.755122654	0.007322646	0.029457498	-3.691436942
MAN1B1	0.085709456	6.859206143	2.754700806	0.007331228	0.029473611	-3.692428043
ZBTB10	-0.226655407	4.893751817	-2.754372435	0.007337915	0.029492623	-3.647564945
EID1	0.088309705	8.43620879	2.753225058	0.007361323	0.029578815	-3.632315013
MEF2A	-0.235578413	5.611066911	-2.752788534	0.007370247	0.029606775	-3.690512252
PDZD4	0.395005242	-0.662224215	2.752169491	0.007382919	0.029649029	-2.777651364
PGD	0.122685143	7.718217519	2.752082465	0.007384702	0.029649029	-3.669699595
MZT2B	0.238600344	4.673175138	2.751484757	0.007396959	0.029690326	-3.630691265
NDUFB11	0.19678797	5.796633321	2.750979936	0.007407326	0.029724015	-3.695906255
ZNF700	-0.17953145	1.573650179	-2.750773616	0.007411566	0.029733112	-3.209057752
PTMS	0.194211254	8.031811315	2.748677881	0.007454767	0.02989846	-3.669513057
PPP3CA	-0.127002286	6.253356849	-2.748463313	0.007459203	0.029908288	-3.712872732
DPP8	-0.191860544	5.093413701	-2.748354076	0.007461463	0.029909386	-3.681996907
EZH1	0.117018964	4.500825299	2.74764706	0.007476101	0.029960091	-3.646030472
ABCD4	-0.102248847	4.386277043	-2.747532419	0.007478477	0.029961642	-3.608326294
KCTD16	-0.527794189	-0.788285962	-2.746985277	0.007489826	0.029999133	-2.730745849
PGBD5	0.451538718	-0.6401411	2.746852387	0.007492585	0.030002207	-2.873021772
GFPT2	0.088414186	6.503074872	2.746307078	0.007503916	0.030039593	-3.718780541
COX7A2	0.169932014	5.486234106	2.746085772	0.007508519	0.030050034	-3.699638836
RBM7	-0.124344045	4.773921916	-2.745573276	0.007519188	0.030068768	-3.64370958
CORO1B	0.145919803	7.290600131	2.745746309	0.007515584	0.030068768	-3.707792966
PRKAG1	0.110651869	6.274696667	2.745588457	0.007518872	0.030068768	-3.719611348
KIAA0355	-0.252549733	4.217981293	-2.745337091	0.00752411	0.030072483	-3.579316204
PPARA	-0.324110144	4.873101732	-2.745369086	0.007523443	0.030072483	-3.685124326
PRR14	0.174444533	4.609291518	2.744977956	0.007531599	0.030094432	-3.645875853
CD81	0.154223006	10.35611929	2.744822166	0.00753485	0.030099438	-3.538716418
TP53I3	0.14719558	4.76722176	2.74452025	0.007541154	0.030116634	-3.652282003
TAPT1	-0.1310141	3.457280306	-2.74423351	0.007547145	0.030124588	-3.504066395
MED20	0.098598855	3.960890266	2.744327789	0.007545175	0.030124588	-3.59132103
C14orf28	0.143415003	2.362889902	2.744077253	0.007550412	0.030129645	-3.360264858
NMD3	-0.123700548	5.433920529	-2.743478065	0.007562951	0.030171689	-3.702701817
FANCB	-0.444855543	-0.808744542	-2.743338395	0.007565877	0.030175369	-2.776681834
STAT5B	-0.087485416	5.635193891	-2.743204954	0.007568673	0.03017853	-3.712285683
TMEM254	-0.110131681	3.679782533	-2.74271236	0.007579003	0.030203729	-3.537347929

Gene	Log fold change	Average Expression	t	P-value	Adjusted P-value	B
RNF181	0.196169683	5.074054631	2.742769882	0.007577796	0.030203729	-3.68313692
RAD50	-0.133464569	6.152436346	-2.742613446	0.007581078	0.030204011	-3.725852948
ZNF518B	-0.134347541	3.987994628	-2.742196953	0.007589825	0.03022689	-3.596068513
AVL9	-0.222047018	4.248665784	-2.742148913	0.007590834	0.03022689	-3.631472655
MAP3K2	-0.201958762	5.267452147	-2.741841219	0.007597302	0.030244651	-3.705217891
TM2D3	-0.109770873	4.605651352	-2.740914344	0.007616817	0.030314327	-3.655031695
CARF	-0.226045326	1.840824885	-2.740430868	0.007627014	0.030346894	-3.214538288
RNASEH1	-0.246524165	3.80138684	-2.740253454	0.007630759	0.030353778	-3.587057107
ARHGEF40	0.077190859	6.673246667	2.739420257	0.00764837	0.030415799	-3.735241944
RAB34	0.130280589	7.301930044	2.738648653	0.007664712	0.030464703	-3.722698565
ZFR	-0.219918678	6.762372602	-2.738729903	0.00766299	0.030464703	-3.73274967
COMMD4	0.15796056	5.068549458	2.738523153	0.007667373	0.030467241	-3.695122705
DBNL	0.116517014	6.816495207	2.738420561	0.007669549	0.03046785	-3.735229062
MFN2	0.075555233	6.768633121	2.738072274	0.00767694	0.030489172	-3.73542441
ACOT7	0.181023175	4.504165445	2.737889362	0.007680825	0.030496558	-3.657493337
MRPL55	0.222322031	4.016660562	2.73760383	0.007686892	0.030512605	-3.591506507
TMEM71	-0.362025192	-0.549104355	-2.737251764	0.007694379	0.030534278	-2.822057303
LOC101927151	-0.272729867	-0.134739595	-2.737017438	0.007699365	0.030534332	-2.929129205
SUCLG2-AS1	-0.34440774	0.848032831	-2.736965394	0.007700473	0.030534332	-3.05631784
TULP4	-0.171826631	4.624567996	-2.737003989	0.007699652	0.030534332	-3.67658425
IK	0.129150332	6.013035746	2.735992099	0.007721221	0.030608544	-3.741170259
CRIP2	0.206269335	5.315942654	2.735871395	0.007723797	0.030610702	-3.706049883
C9orf172	0.201454434	1.481067431	2.735412193	0.007733606	0.030641517	-3.202253583
INTS14	0.077048377	4.698486696	2.73495878	0.007743303	0.03067187	-3.690274275
TMEM173	0.105183203	6.514005658	2.734324032	0.007756897	0.030717638	-3.748479599
RSAD1	0.156412209	4.360944924	2.734186719	0.00775984	0.030721218	-3.630669218
DAD1	0.134411389	7.204346177	2.734086009	0.007762	0.030721694	-3.73777999
RPS29	0.197422057	6.899049579	2.733354922	0.007777693	0.03077572	-3.747910732
CLSPN	-0.48013406	1.180755265	-2.733045587	0.007784342	0.030785856	-3.170747299
ROR1	-0.35300258	5.672843947	-2.733120346	0.007782734	0.030785856	-3.742322386
PLOD1	0.084204087	8.630808614	2.732133934	0.007803967	0.030855369	-3.673394041
MFSD11	-0.113154695	3.819250642	-2.73159675	0.007815552	0.030893066	-3.609658704
SLC25A37	-0.15262464	4.388053707	-2.731100125	0.007826276	0.030927341	-3.672319811
P2RY11	-0.290583125	1.080171196	-2.730769967	0.007833413	0.030947426	-3.041747156
YIPF2	0.105876705	4.928694761	2.730530386	0.007838596	0.030959782	-3.707882062
RPS25	0.183204053	8.487242161	2.730426866	0.007840836	0.030960513	-3.691061361
SLC35B4	-0.227470287	5.126993736	-2.730283126	0.007843948	0.030960987	-3.724549669
C20orf27	0.151526466	5.668260833	2.730136553	0.007847122	0.030960987	-3.74559209
FBXW2	-0.110575556	6.111054504	-2.730157345	0.007846672	0.030960987	-3.758274867
RRS1	-0.177710959	4.733177432	-2.729717817	0.007856197	0.030972456	-3.687402718
DLD	-0.09566734	5.67812182	-2.729808684	0.007854227	0.030972456	-3.749927483
RBM25	-0.200926413	5.931469036	-2.729755235	0.007855386	0.030972456	-3.75999087
MAB21L1	0.283542931	2.395450548	2.729579157	0.007859204	0.030976204	-3.432242373
SLC38A4	-0.380074769	2.454963776	-2.729478862	0.00786138	0.030976675	-3.381340896
GTF2H3	-0.171182317	4.328619617	-2.729013423	0.007871484	0.031008378	-3.679987
CREB3L4	-0.192199891	1.925388353	-2.728199721	0.007889178	0.031061832	-3.239627824
LMLN	-0.294398638	2.820919854	-2.728290248	0.007887207	0.031061832	-3.448052046
MUL1	-0.08796338	5.614089537	-2.727956467	0.007894474	0.031074564	-3.7490392
DLX4	-0.979572054	-3.281452776	-2.727118615	0.007912741	0.0311302	-2.539520565
FAM171B	-0.316371365	4.304352719	-2.727157704	0.007911888	0.0311302	-3.671926326
BIVM	-0.106455223	4.074847019	-2.726867985	0.007918213	0.031139734	-3.619810681
MTDH	-0.223798638	7.104523868	-2.726818278	0.007919299	0.031139734	-3.752043507
CCNI	0.134805374	9.455056541	2.7262402	0.007931935	0.03118128	-3.646304581
PP2D1	-0.776417972	-2.264523762	-2.72462711	0.007967291	0.031303933	-2.597659712
MLLT1	-0.08550763	7.038455564	-2.724705683	0.007965566	0.031303933	-3.7666874
E2F6	-0.161344425	2.67381161	-2.724437173	0.007971464	0.03131216	-3.376628723
FUOM	0.27882446	1.149082317	2.724107449	0.007978712	0.031332462	-3.148181623
MTCH2	0.0957395	6.479641091	2.723854829	0.00798427	0.031346114	-3.77515916
LSM1	0.166563	3.63048943	2.723691715	0.00798786	0.031352038	-3.593623391
CIPC	-0.24255763	2.420102924	-2.723502335	0.00799203	0.031352068	-3.444566423
NUFIP1	-0.172303236	2.95549316	-2.723502694	0.007992022	0.031352068	-3.468060966
RBSN	-0.219624713	5.030765948	-2.723075164	0.008001444	0.031380825	-3.743574061
RWDD2A	-0.163511528	2.96371086	-2.72285824	0.008006228	0.031383247	-3.505908459
ZNF318	-0.179242327	5.026761088	-2.722874959	0.008005859	0.031383247	-3.744770322
PEMT	0.181355517	4.1207215	2.722538635	0.008013282	0.031399856	-3.65265294
ORC3	0.090593008	4.526614994	2.722477392	0.008014634	0.031399856	-3.700541234
AHRR	-0.163858747	5.529719084	-2.7223038	0.008018468	0.031406709	-3.771999754
ZDHHC24	0.144947625	4.335613227	2.722087288	0.008023253	0.031407771	-3.677468872
MPDZ	-0.238756707	5.489280731	-2.722008511	0.008024994	0.031407771	-3.763116014
CSNK1A1	-0.2569973	6.421270647	-2.722152115	0.00802182	0.031407771	-3.779109927

Gene	Log fold change	Average Expression	t	P-value	Adjusted P-value	B
NFIC	-0.09750651	7.901152932	-2.720883048	0.008049911	0.031497108	-3.738736804
ESD	0.11248125	7.363420249	2.720718706	0.008053556	0.031503185	-3.766005729
PTGES3	0.067225124	7.489607452	2.719979934	0.008069957	0.031559147	-3.761371846
FLT1	-0.38588404	2.545852607	-2.719521217	0.008080156	0.031568332	-3.441005623
LAMTOR2	0.17329806	4.901331268	2.719497303	0.008080688	0.031568332	-3.729782516
AKIRIN1	-0.154649598	5.356802422	-2.719600187	0.0080784	0.031568332	-3.742832575
NECTIN2	0.112859574	5.754775762	2.719549047	0.008079537	0.031568332	-3.778148726
EFHD2	0.131785309	5.77758998	2.719031744	0.008091052	0.031600625	-3.781201766
TAB3	-0.143689019	4.049626332	-2.717611538	0.008122743	0.031714043	-3.662648835
GRSF1	-0.074838911	6.55582332	-2.717541781	0.008124303	0.031714043	-3.790185573
FAM129A	-0.287243472	7.326064741	-2.717065829	0.00813495	0.031747381	-3.77218547
CD99	0.139468954	9.391622465	2.716949962	0.008137544	0.031749279	-3.668730519
WDR73	-0.226084515	1.072498339	-2.716323228	0.008151589	0.031795839	-3.158385178
EDF1	0.203319768	7.648140743	2.715083966	0.008179424	0.031896154	-3.771626484
FUBP3	-0.203756242	5.710469326	-2.714157618	0.008200288	0.031969238	-3.79575749
LRR1	-0.158839435	2.383012946	-2.713961794	0.008204704	0.03197818	-3.435299508
RCBTB1	-0.119951289	4.786217446	-2.713532294	0.008214399	0.032007684	-3.74199708
SLC16A3	0.19867978	5.600657132	2.7131595	0.008222822	0.032032219	-3.796310551
C20orf144	0.744858482	-2.910105689	2.712409469	0.008239793	0.032089944	-2.648802785
ALKBH6	0.620973942	-1.531386461	2.712316399	0.008241901	0.032089944	-2.743540681
RPL10	0.150383835	8.731554285	2.711992631	0.008249238	0.032110212	-3.722783386
KDM7A	-0.268539614	4.28390656	-2.711766046	0.008254377	0.032113618	-3.705901128
EIF3I	0.135584354	7.768580639	2.711784527	0.008253957	0.032113618	-3.773526041
PCID2	-0.083644559	5.128347447	-2.71145222	0.008261498	0.032133026	-3.767336436
CCDC106	0.183904779	4.231354555	2.711261036	0.00826584	0.032141613	-3.681999554
DNAJC18	0.104241994	4.191559031	2.710668211	0.008279314	0.032185701	-3.68835462
EFEMP2	0.105114278	8.423843518	2.709741144	0.008300427	0.032251129	-3.744440905
SUMO2	0.102223079	6.083963092	2.709831795	0.00829836	0.032251129	-3.808166017
FAM49B	-0.137951691	3.347825531	-2.709562408	0.008304503	0.032258646	-3.567166701
FAM200A	0.173134353	2.545182441	2.708925863	0.008319034	0.032306761	-3.545543654
MT1X	0.219730496	1.485465098	2.708653194	0.008325266	0.032322629	-3.280151974
TMEM115	0.111654029	6.331427715	2.708426515	0.008330449	0.032334421	-3.813664204
MSL2	-0.25309651	4.126309749	-2.70688681	0.008365738	0.032463028	-3.705912897
YPEL5	-0.063003338	6.658527068	-2.706458077	0.008375588	0.032492883	-3.817799919
SLC25A32	-0.177696999	4.825436785	-2.705869633	0.008389125	0.03253702	-3.740225255
TCTE3	-0.394865664	-0.859038369	-2.705616812	0.008394948	0.032542844	-2.862339888
NCKAP1	-0.20434706	7.522326983	-2.705679772	0.008393497	0.032542844	-3.791140141
POLR2J	0.205449127	3.733202491	2.705362141	0.008400816	0.032557215	-3.643140355
TMEM250	0.073757389	6.368877724	2.705072047	0.008407506	0.03257476	-3.822156302
RILPL2	0.140112289	4.770626413	2.70427434	0.008425925	0.032637732	-3.757924481
TMEM94	-0.088658577	5.420900409	-2.704142028	0.008428984	0.032641187	-3.801705299
RPLP1	0.15807719	9.948795264	2.703784386	0.008437257	0.032664827	-3.668618026
LOC100507053	-0.359039628	-0.012688585	-2.703688584	0.008439474	0.032665016	-2.948281566
WDR11	-0.12592742	5.157580324	-2.702338694	0.008470774	0.03277774	-3.796308926
GSPT2	-0.203249086	4.066378338	-2.701245489	0.0084962	0.032867682	-3.725024137
SIGMAR1	0.135853768	6.762064178	2.700904589	0.008504143	0.032889962	-3.830356589
MESP2	0.360912357	-0.11977672	2.700632201	0.008510494	0.03290559	-2.956886883
THEM4	-0.132814941	2.87211172	-2.700543969	0.008512552	0.03290559	-3.484457352
KAT6B	0.164215892	4.998370542	2.700071122	0.00852359	0.032939807	-3.791646472
CSTB	0.197963182	7.595659405	2.699919308	0.008527137	0.032945061	-3.810206978
PPM1L	-0.333758811	2.943446782	-2.699632294	0.008533846	0.03296088	-3.540374488
ABHD11	0.194775409	3.07858363	2.699556975	0.008535608	0.03296088	-3.573184729
CDKN2D	-0.221944581	1.634868405	-2.698804231	0.008553229	0.032975744	-3.270042407
ARHGEF19	0.274912466	1.615056548	2.698825779	0.008552724	0.032975744	-3.296502502
SLC30A4	-0.192963435	2.779238956	-2.699049837	0.008547476	0.032975744	-3.530370672
TCHP	0.131155806	3.852572961	2.69923971	0.008543031	0.032975744	-3.674832448
HAUS2	-0.161030972	3.886393213	-2.698738062	0.00855478	0.032975744	-3.707901281
RIF1	-0.285307645	5.21177444	-2.699027974	0.008547988	0.032975744	-3.817457403
ERP29	0.144102393	7.428186751	2.698744818	0.008554622	0.032975744	-3.819210016
FMO5	-0.285219294	0.912869352	-2.698340111	0.008564111	0.032996525	-3.164756379
NECAP2	0.067702577	6.194173317	2.698321304	0.008564552	0.032996525	-3.837529832
ANKRD37	0.269165589	0.577725696	2.697918691	0.008574002	0.033024488	-3.101334094
WDFY1	-0.138545932	6.422632304	-2.697280341	0.008589005	0.033073818	-3.840614166
PLEKHA6	0.232629941	2.86151841	2.69591093	0.008621271	0.033172624	-3.491087215
TADA2A	-0.160009986	3.1718641	-2.69595389	0.008620257	0.033172624	-3.625227251
MAK16	-0.165052792	3.875494573	-2.695991191	0.008619377	0.033172624	-3.699944252
NDUFA10	0.079100222	6.047076246	2.695269438	0.008636423	0.033222442	-3.843519159
ZBED8	0.223439855	1.013492394	2.694819244	0.008647072	0.033254912	-3.391033046
AKAP9	0.267090293	4.11318565	2.69418195	0.008662166	0.033304459	-3.74836674
C7orf50	0.184683441	5.440039948	2.693151896	0.008686614	0.033389934	-3.825366232

Gene	Log fold change	Average Expression	t	P-value	Adjusted P-value	B
DOC2B	0.857283429	-4.642036791	2.69285844	0.00869359	0.033408226	-2.592785764
MFSD10	0.130728294	5.763818705	2.6923368	0.008706004	0.033447398	-3.84381976
ZNF275	-0.229581869	5.464952035	-2.691571721	0.00872424	0.033508913	-3.813907749
DOCK4	-0.314304974	2.79547175	-2.691471836	0.008726624	0.033509524	-3.52838241
KIAA0556	-0.111565047	4.245468488	-2.690959218	0.008738865	0.033547978	-3.742601678
BTN3A1	-0.128550465	4.866730295	-2.690859225	0.008741255	0.033548602	-3.800588172
GDF1	0.280817523	0.783222727	2.690723183	0.008744507	0.033552535	-3.18170962
TAF12	0.174974959	4.774225171	2.690421208	0.00875173	0.03356315	-3.787041516
DHRS7	0.131550503	5.616885629	2.690451057	0.008751015	0.03356315	-3.845075596
MED26	-0.202134979	2.26878313	-2.689841319	0.008765615	0.033607844	-3.415355952
PIPSL	-0.412984606	-0.63485326	-2.689465743	0.008774618	0.033633804	-2.89654613
ZSCAN16-AS1	0.2465083	2.171779451	2.688851483	0.008789362	0.033681747	-3.420147266
GPX4	0.167790777	8.679077841	2.688557234	0.008796433	0.03370027	-3.783906099
PPP4C	0.137383179	5.936140842	2.688302991	0.008802546	0.033715117	-3.85886495
ALDH1B1	-0.14921675	5.628182755	-2.688031303	0.008809083	0.03373158	-3.846456057
JPT1	0.136799973	5.375219829	2.687834232	0.008813828	0.033732598	-3.841299289
FKBP1A	0.133528312	7.427072953	2.687918068	0.008811809	0.033732598	-3.845452445
ZNF688	0.225965997	3.586943076	2.687489601	0.008822131	0.033755799	-3.669007179
FOPNL	-0.078593885	5.009981582	-2.687358301	0.008825296	0.033759335	-3.813185004
KIAA1586	0.130396327	3.400579002	2.687087592	0.008831825	0.033775734	-3.649926755
AMMECR1L	-0.198504259	4.214633974	-2.686929758	0.008835634	0.033781723	-3.763111814
CAMSAP2	-0.189443854	6.25202288	-2.686588583	0.008843872	0.033804641	-3.866838586
PLIN4	0.391555973	0.827825111	2.686298037	0.008850893	0.033814318	-3.163474955
SOD1	0.16464794	7.024784783	2.686368327	0.008849194	0.033814318	-3.862472789
KCTD7	-0.193970598	3.632113853	-2.686062578	0.008856587	0.033816557	-3.736599628
FUS	0.142951632	7.407780241	2.685995318	0.008858214	0.033816557	-3.839713749
DHX15	-0.130672453	6.484887718	-2.686113678	0.008855351	0.033816557	-3.867928718
ZC4H2	-0.124264383	3.151688014	-2.685610527	0.008867527	0.033843534	-3.590300907
SYTL3	-0.303222754	0.633305959	-2.685414353	0.008872279	0.033853092	-3.114930623
ICAM1	-0.208726098	5.265109286	-2.685058055	0.008880915	0.033868886	-3.8254134
CSF1	-0.1424868	7.726231558	-2.685124075	0.008879314	0.033868886	-3.846214156
PPIP5K2	-0.207123959	4.634111644	-2.684494537	0.008894589	0.033900553	-3.808582922
TNPO1	-0.254267239	7.088688017	-2.684437676	0.00889597	0.033900553	-3.857514938
SYT11	-0.355251169	5.992969381	-2.684568438	0.008892794	0.033900553	-3.871047686
CAPZB	0.120254139	8.347626574	2.683920342	0.008908542	0.033923745	-3.811677865
SEC23IP	-0.208598834	5.787131513	-2.683918831	0.008908578	0.033923745	-3.872470269
GRHPR	0.141542954	6.211814528	2.683909248	0.008908811	0.033923745	-3.873367217
ELAC1	0.182120436	2.007886057	2.683680487	0.008914376	0.033936356	-3.42337397
MAPK8IP1	0.129655472	4.305049983	2.683553784	0.008917459	0.033939517	-3.770966624
CLYBL	0.269103191	0.507901611	2.683355594	0.008922284	0.033949304	-3.111248174
TCP11L1	0.142005768	3.647692727	2.682128807	0.008952204	0.034054546	-3.704396393
NFKBIL1	0.200628778	4.449658033	2.682000967	0.008955327	0.034057826	-3.782380358
MED14	-0.166644086	4.659183325	-2.681859097	0.008958794	0.034062412	-3.83009928
PEX11A	0.182107882	1.944064876	2.681409576	0.008969788	0.034095604	-3.447152799
POLR2L	0.221152493	7.086634963	2.680879926	0.008982756	0.034136287	-3.874532937
RPL41	0.151483826	8.337435909	2.680520234	0.008991573	0.034161176	-3.822567854
PCDHA10	-0.605158697	-1.985221213	-2.679156749	0.009025068	0.034279783	-2.772925506
DCXR	0.199722898	4.486226276	2.678865928	0.009032226	0.034298325	-3.791009626
NARFL	0.178156818	3.878020926	2.677915671	0.009055654	0.03437862	-3.740296284
SLC7A11-AS1	-0.851394802	-4.350509696	-2.677383196	0.009068805	0.034419874	-2.626181251
CSTF2	0.121421464	3.122349131	2.676669345	0.009086464	0.034478209	-3.667524178
HDHD3	0.173351978	2.276762491	2.6765189	0.009090189	0.03448366	-3.536436692
FBL	0.138197881	6.783574974	2.675322871	0.009119857	0.034587494	-3.893741116
CASKIN2	-0.118131636	4.181000156	-2.675196228	0.009123003	0.034590718	-3.809914989
FAM20A	0.120349576	7.179042375	2.675034968	0.009127012	0.034597207	-3.884792868
STX7	-0.138382642	5.749008874	-2.674466619	0.00914115	0.034642085	-3.886699843
SUV39H1	-0.198172663	1.731947667	-2.673950073	0.009154018	0.034682124	-3.395962246
NUDT12	0.1874754	2.76159686	2.672765953	0.009183577	0.034785368	-3.610801589
ALG2	-0.069626562	5.933632885	-2.672548392	0.009189018	0.034797225	-3.898445009
WDR89	-0.165282791	2.797425055	-2.67229096	0.009195459	0.034805965	-3.630294673
KMT5A	-0.142417293	3.746699588	-2.672271471	0.009195947	0.034805965	-3.737845271
PRDX2	0.174807981	6.452058434	2.671489635	0.009215535	0.034871345	-3.905143703
TACSTD2	0.485805705	-0.838815006	2.671355948	0.009218889	0.034872729	-2.98108707
TMEM54	0.211343938	4.475061007	2.671290485	0.009220531	0.034872729	-3.811380554
AKAP3	0.576986294	-1.057163545	2.670852167	0.009231535	0.034905583	-2.862919239
TAF1A	-0.167286567	2.311224519	-2.670391078	0.009243124	0.034940631	-3.548446651
PTK2B	-0.251996244	2.700464454	-2.670186774	0.009248263	0.034951286	-3.520663078
VPS45	-0.099043383	4.553587555	-2.670028583	0.009252243	0.034957561	-3.81693856
CD151	0.13824221	8.478696343	2.669746509	0.009259346	0.034975622	-3.841526384
PDAP1	0.148853326	6.215826057	2.669162891	0.009274056	0.035022407	-3.909675414

Gene	Log fold change	Average Expression	t	P-value	Adjusted P-value	B
RAC3	0.298745856	0.641563006	2.668251515	0.00929707	0.035094069	-3.203470162
DNTTIP1	0.168336908	4.378138231	2.668216575	0.009297954	0.035094069	-3.814832409
CRYBG1	-0.256159955	4.375885278	-2.668134797	0.009300021	0.035094069	-3.821426017
ITCH	-0.267839705	5.438591397	-2.66795929	0.009304461	0.035102027	-3.900361163
FLJ31104	-0.829755065	-2.937266503	-2.667773444	0.009309163	0.035110976	-2.680690509
BICRAL	-0.20797194	4.480551892	-2.667449732	0.00931736	0.035133094	-3.822982064
NEDD4L	-0.234682438	3.340966251	-2.665712217	0.009361468	0.035290578	-3.673895519
CDK11B	0.109305632	4.4173471	2.66549384	0.009367025	0.035302692	-3.82904341
CCDC127	-0.101716965	5.073980381	-2.665400005	0.009369413	0.035302862	-3.889315396
ROBO1	-0.239749975	5.326316137	-2.664554557	0.009390961	0.035375201	-3.900810422
NUAK2	0.546155838	0.782516435	2.664284428	0.009397855	0.035392321	-3.117372791
SLC39A14	-0.376023154	6.954996391	-2.664058463	0.009403625	0.035405201	-3.905026291
CHD1	-0.25281994	4.706783403	-2.663368925	0.009421254	0.03546271	-3.845561281
CXCL16	0.202542364	2.716968267	2.662076901	0.009454367	0.035569231	-3.622927292
APOOL	-0.202267648	4.062361895	-2.662156116	0.009452333	0.035569231	-3.79748957
BAD	0.187319	4.680530881	2.661988482	0.009456636	0.035569231	-3.852700604
MDC1	-0.157927867	4.348761857	-2.66187539	0.00945954	0.035571273	-3.840771093
ANAPC11	0.187745501	5.563955772	2.661664224	0.009464965	0.035582789	-3.910566442
SH3RF1	-0.176369662	4.827775463	-2.660906158	0.009484462	0.035647191	-3.813402995
CTDNEP1	0.112047835	6.504929502	2.660715272	0.009489378	0.035656768	-3.931926599
CTNNB1	-0.108799114	8.227851697	-2.660512676	0.009494597	0.035662444	-3.872304018
URM1	0.142759968	6.232684066	2.660472868	0.009495623	0.035662444	-3.930789505
DPP7	0.17165544	7.41013579	2.659197117	0.009528552	0.035777196	-3.918713174
TMSB4X	0.150094615	8.876996932	2.658761722	0.009539814	0.035810557	-3.843145217
PANO1	-0.346328485	-0.590936423	-2.658356347	0.009550311	0.035832101	-3.021809963
MRPL19	-0.128651845	4.902607237	-2.658391321	0.009549405	0.035832101	-3.888114142
TRIM37	-0.167195295	4.575865602	-2.658039719	0.009558517	0.035853958	-3.871572193
ENKUR	-0.786591607	-2.927327008	-2.657499329	0.009572536	0.035897608	-2.74652416
ADAM17	-0.27251005	4.898540068	-2.656517042	0.009598069	0.035975443	-3.902489651
MFSD14A	-0.238962069	5.783326398	-2.656594882	0.009596043	0.035975443	-3.940467954
PCDH9	-0.349854508	1.735610488	-2.655512379	0.009624246	0.036064588	-3.430184762
BLMH	0.094178516	5.271474201	2.654713896	0.009645098	0.036133737	-3.920170334
IL16	-0.277332294	2.26459542	-2.654502742	0.009650619	0.036145431	-3.545866205
NDFIP2	-0.263305616	4.833454032	-2.654329631	0.009655147	0.036153403	-3.91621635
MARCH4	-0.303619729	3.494616595	-2.654160268	0.00965958	0.03616101	-3.753669828
SCOC	-0.075451225	5.734836411	-2.654000099	0.009663773	0.03616772	-3.93850741
PPP2R2C	0.638508735	-2.252646964	2.653890293	0.009666649	0.036169497	-2.817834882
DPM3	0.204741268	3.582241708	2.653154254	0.009685945	0.036219014	-3.754160775
DDR2	-0.31879523	8.455201727	-2.653110409	0.009687096	0.036219014	-3.873838926
SLC36A1	-0.193773602	4.80513809	-2.653260075	0.009683169	0.036219014	-3.905038316
MIR646HG	-0.782012616	-2.724099023	-2.652370739	0.009706525	0.036282652	-2.768909872
ADCY8	0.582601002	0.04061329	2.652133363	0.009712767	0.036287978	-3.142038931
VAPB	-0.132023368	5.771267117	-2.65217424	0.009711692	0.036287978	-3.9436439
NLRP10	-0.618534228	-1.979444126	-2.651828242	0.009720797	0.036308506	-2.803068893
KLF9	-0.173122227	4.966092688	-2.651741446	0.009723082	0.036308506	-3.89258189
LOC101928820	-0.65318008	-2.813623376	-2.651520517	0.009728901	0.036312232	-2.772144638
HIPK1	-0.227001906	5.021188988	-2.651530661	0.009728634	0.036312232	-3.925219903
CCDC84	0.347048911	1.165030615	2.650930543	0.009744456	0.036343275	-3.330653234
RPL5	0.12502499	9.729726405	2.650954757	0.009743817	0.036343275	-3.810386668
ZDHHC5	-0.112136548	7.05572994	-2.651000728	0.009742605	0.036343275	-3.946108969
TUSC1	0.119826121	3.94002048	2.650323112	0.009760495	0.036394083	-3.799125865
PCOLCE	0.169623069	9.991099509	2.649528144	0.009781522	0.03646346	-3.800363939
ZCCHC3	0.106137668	4.431519196	2.648999653	0.009795523	0.036497505	-3.868062419
TSPO	0.199028751	6.878853741	2.649080652	0.009793376	0.036497505	-3.956363256
TAF15	0.100550858	6.857626642	2.648909126	0.009797923	0.036497505	-3.957021341
CDC23	0.096061097	4.639632315	2.648291043	0.009814324	0.036549563	-3.900114486
BCAT1	-0.227590456	6.385118629	-2.647740629	0.009828951	0.036594988	-3.961753835
MAMDC4	0.227753806	0.328867434	2.647063865	0.009846963	0.036625839	-3.199805862
DUSP10	0.223578105	3.00009696	2.647136519	0.009845028	0.036625839	-3.719542719
PSMB3	0.176216303	5.560442356	2.647173569	0.009844041	0.036625839	-3.948459304
ARF5	0.146012002	6.403746508	2.647243017	0.009842192	0.036625839	-3.96467489
ZNF358	0.21594481	6.706734372	2.646831294	0.00985316	0.036639841	-3.965346385
GPN2	0.083891431	4.233620678	2.646521446	0.009861421	0.03666151	-3.867470199
YJEFN3	0.664607705	-1.427217239	2.646242671	0.009868859	0.03668011	-2.946328507
CHMP4B	0.110694008	6.739273263	2.645802153	0.009880623	0.036714775	-3.965799539
ZNF107	-0.215944509	1.723255996	-2.644953115	0.009903332	0.036781013	-3.536373479
COTL1	0.124868429	7.056563987	2.644982192	0.009902553	0.036781013	-3.960454155
TSTA3	0.167774079	5.171694333	2.64457048	0.009913582	0.036810006	-3.934453596
MAP1LC3B2	0.27351767	0.23752053	2.643413557	0.009944631	0.036908424	-3.191425399
MRPL21	0.187570242	4.422098785	2.643400302	0.009944988	0.036908424	-3.876728476

Gene	Log fold change	Average Expression	t	P-value	Adjusted P-value	B
IL6ST	-0.237566402	9.469403397	-2.642956737	0.009956916	0.036943592	-3.834167487
CARNMT1	-0.178704494	4.130918238	-2.642724986	0.009963153	0.036957632	-3.878714987
DDX31	-0.14084946	3.49004792	-2.642196286	0.009977396	0.037001353	-3.797529537
LRRC45	-0.17603788	3.566138979	-2.641697688	0.009990845	0.037042109	-3.73660783
DNPH1	0.201900755	4.397597684	2.641375883	0.009999534	0.037065201	-3.877438278
TAF6	0.099946627	5.16132689	2.640931873	0.010011533	0.037100551	-3.941073302
PHF1	0.109705727	5.546171839	2.640117915	0.010033565	0.037173052	-3.96203721
LIPE	0.326146168	-0.087888788	2.639629437	0.010046808	0.037203815	-3.115835223
OGFR	-0.138677176	5.633646422	-2.639648154	0.010046301	0.037203815	-3.973663525
PLTP	0.163655885	6.86975571	2.639249397	0.010057122	0.037232856	-3.980997791
MRPL30	0.082238943	4.199960718	2.639027743	0.010063142	0.037236841	-3.877628837
CACNA1C	-0.256681514	4.661911617	-2.639049549	0.01006255	0.037236841	-3.909603548
PDIA5	0.111277856	5.613986976	2.63871063	0.010071761	0.037259579	-3.973005992
RTL8A	0.173637291	5.565297659	2.638365585	0.010081146	0.037285142	-3.967910072
CEP41	-0.120067509	3.512522471	-2.637482616	0.010105199	0.037356087	-3.757940092
CTTNBP2NL	-0.226741201	5.736615994	-2.637479348	0.010105288	0.037356087	-3.973804872
SPNS2	0.679545307	-2.308335543	2.63661709	0.010128827	0.037424735	-2.861652658
MTCH1	0.115472511	8.095319736	2.636644299	0.010128083	0.037424735	-3.943446956
MTFR1	0.092710726	4.56430485	2.635909915	0.010148169	0.037487008	-3.925290807
TMTC2	-0.33708732	3.892060639	-2.635522476	0.01015878	0.037517006	-3.817453737
C11orf63	-0.183505682	2.432765876	-2.635428709	0.01016135	0.037517298	-3.566201147
ZNF675	-0.199259832	1.479333014	-2.634606236	0.010183915	0.037591397	-3.492187759
SCYL2	-0.169886605	5.295305786	-2.634393329	0.010189763	0.037603771	-3.978506142
TCF7	0.255855292	4.362222623	2.634257271	0.010193502	0.037608357	-3.921636665
LARP4	-0.247195136	5.145740453	-2.634066854	0.010198737	0.037618458	-3.959172897
COL6A2	0.116701296	13.2905484	2.633837064	0.010205058	0.037632558	-3.619105532
HIGD1A	0.125976096	4.163056836	2.633099235	0.010225378	0.037689036	-3.890407083
VPS37A	-0.087885702	5.0465653	-2.633128307	0.010224576	0.037689036	-3.958323017
NUP98	-0.178383202	6.661368835	-2.632530716	0.010241059	0.037737602	-3.998069123
MFSD12	0.09428725	5.325483028	2.631229315	0.010277039	0.037851666	-3.982666251
FXYD5	0.227194104	7.424559882	2.631241075	0.010276713	0.037851666	-3.986595274
IPO4	0.316672269	-0.044278138	2.630892663	0.010286365	0.037876753	-3.17675806
DGCR6L	0.196744817	4.889428274	2.629841378	0.010315537	0.03797489	-3.945614843
PEBP1	0.148030318	8.119112642	2.629680772	0.01032	0.037982039	-3.958219031
DCAF7	-0.207780535	6.093280689	-2.629373186	0.010328553	0.038004231	-4.008542794
POLL	0.13796402	4.282545823	2.628070866	0.010364837	0.038128425	-3.891394607
SH3GLB2	0.144762941	5.064885424	2.627107208	0.01039176	0.038218132	-3.97574855
COX6B1	0.202338511	6.514782531	2.626955782	0.010395996	0.03822438	-4.014194751
CFAP46	0.580676884	-2.040672239	2.626581439	0.010406475	0.038248012	-2.901087581
ZNF674-AS1	-0.344860478	0.239611108	-2.626363543	0.01041258	0.038248012	-3.150406767
NPM3	0.127806798	4.56812767	2.62645165	0.010410111	0.038248012	-3.935501818
SLK	-0.2572839	6.537911526	-2.626431114	0.010410686	0.038248012	-4.01401942
ERVK3-1	0.268242715	0.905285905	2.625990315	0.010423043	0.038267252	-3.35214174
PXMP2	0.198470827	1.467776198	2.626036213	0.010421756	0.038267252	-3.45621117
ARSI	0.252212271	1.443051012	2.62585275	0.010426902	0.038267252	-3.506821156
LEPR	-0.247040874	3.238149592	-2.625814372	0.010427979	0.038267252	-3.807035437
EIF3G	0.154348312	7.591062936	2.625714258	0.010430788	0.03826824	-3.993655142
KRT18	0.626584817	-1.208740364	2.625245258	0.010443959	0.03830723	-2.971550962
ZNF221	-0.404298477	-0.568697559	-2.624103754	0.010476078	0.038402634	-3.219881519
CD44	-0.127129196	10.06345768	-2.624137862	0.010475117	0.038402634	-3.85444115
DERL1	-0.097773404	6.010397917	-2.624049127	0.010477617	0.038402634	-4.017462793
GPS1	0.155265116	6.124256528	2.623356858	0.010497142	0.038464838	-4.019978748
CALR	0.100181583	9.73151435	2.622714405	0.010515292	0.038521973	-3.872246389
TIMM21	0.139432541	3.595499672	2.62217037	0.010530683	0.038568979	-3.836878631
ATP2C1	-0.115850305	6.942124996	-2.621991892	0.010535737	0.038578109	-4.019752144
SWAP70	-0.192467719	7.131390782	-2.621847029	0.010539841	0.038583756	-4.020516122
CCDC130	0.184854994	3.300791651	2.621574996	0.01054755	0.038602599	-3.804780286
THTPA	0.171346152	2.226616528	2.621328137	0.010554551	0.038618839	-3.613735106
RANBP6	-0.200934775	4.279284678	-2.620804153	0.010569425	0.038663871	-3.936661759
ZCWPW1	-0.166522321	2.757426913	-2.620661926	0.010573466	0.038669262	-3.688303708
SLC39A8	-0.161644262	6.191259184	-2.62013154	0.010588546	0.038715014	-4.030220145
ZNF101	-0.200915075	1.334846743	-2.619668363	0.010601731	0.038734977	-3.505986519
NMB	0.258313962	3.263992213	2.619578493	0.010604291	0.038734977	-3.804232631
ITPR2	-0.310386911	4.082860536	-2.619721317	0.010600223	0.038734977	-3.915958092
PCYT2	0.123674539	6.175663073	2.619660331	0.01060196	0.038734977	-4.0310736
CCDC124	0.2056157	5.74720605	2.619471298	0.010607346	0.038736741	-4.019091231
MYO10	-0.232498056	6.285132718	-2.619052922	0.010619274	0.038770903	-4.032045779
PTCH1	-0.296203855	2.246800279	-2.618763356	0.010627537	0.038791669	-3.663215809
PLK4	-0.311829557	1.22523945	-2.618430804	0.010637034	0.038811274	-3.419105968
MAU2	0.099368412	4.89619244	2.618394855	0.010638061	0.038811274	-3.9862164

Gene	Log fold change	Average Expression	t	P-value	Adjusted P-value	B
PROSER2	0.337159961	0.948317089	2.618164042	0.010644658	0.038816539	-3.311899707
MRPL16	0.166587384	4.337899454	2.618215176	0.010643196	0.038816539	-3.930602964
ANK2	-0.196956358	6.817270048	-2.617683577	0.010658401	0.038857249	-4.035185649
CACHD1	-0.348654863	4.233920405	-2.617551579	0.01066218	0.03886162	-3.910965595
MAFG-AS1	0.203520608	2.497132719	2.617385767	0.010666928	0.038869521	-3.663483784
CCDC82	-0.143015251	3.901666129	-2.617169723	0.010673117	0.03888267	-3.90316365
MRPL48	0.144661797	4.089239304	2.616178739	0.01070155	0.038976826	-3.909452003
GPR153	0.10813089	6.672479104	2.615591691	0.010718426	0.039028854	-4.038409578
PEG3	0.518506112	-0.671997394	2.615208094	0.010729466	0.039059613	-3.108194856
GPANK1	0.100449916	3.942276909	2.615010428	0.010735159	0.039070895	-3.933776619
ZIK1	-0.180217979	1.930102826	-2.613282689	0.010785036	0.039242944	-3.601186058
NTHL1	0.251263273	2.826580217	2.612319909	0.010812921	0.03932541	-3.702755581
PIGN	-0.271784158	4.108350041	-2.612396472	0.010810702	0.03932541	-3.941252523
RPS7	0.171055412	6.3775648	2.611600367	0.010833804	0.039391848	-4.051177295
CARS	-0.128335634	6.15774437	-2.611082687	0.010848851	0.03943704	-4.047027981
DRP2	-0.52875623	-1.186545344	-2.610512576	0.010865444	0.039440252	-3.00682046
NBEAL2	-0.225857193	1.383945495	-2.610625548	0.010862154	0.039440252	-3.447256709
MLKL	0.17161153	2.938903918	2.610574203	0.010863649	0.039440252	-3.73427568
MBD5	-0.222900201	3.465421716	-2.610600964	0.01086287	0.039440252	-3.792663186
RPL36	0.20350813	8.769018799	2.610939101	0.010853028	0.039440252	-3.969932237
ZFYVE16	-0.21819297	5.054465212	-2.610654721	0.010861305	0.039440252	-4.016077064
UVSSA	-0.324399633	1.531044884	-2.610121262	0.010876846	0.03947213	-3.455811145
BRK1	0.096030816	7.37565603	2.609504816	0.01089483	0.039527872	-4.039186655
PIGO	-0.165847442	3.813312998	-2.607855055	0.010943093	0.039683861	-3.894398071
MAST2	-0.101473997	6.036414366	-2.607909552	0.010941496	0.039683861	-4.052245146
TPRA1	0.113416358	5.038765604	2.607320135	0.010958784	0.039731196	-4.019689501
LMF2	0.122153481	6.882325409	2.606946982	0.010969741	0.039761353	-4.059683792
RAB40B	0.126746801	3.744506563	2.606493624	0.010983067	0.039800078	-3.913720528
TTC9B	-0.799167517	-2.85574683	-2.606373333	0.010986606	0.039803325	-2.813286731
CCDC136	-0.163633903	2.191747924	-2.604925165	0.011029284	0.039948336	-3.628426962
POLR1C	-0.208792076	3.869990066	-2.604591917	0.011039126	0.039974373	-3.910759566
ETNK2	0.204450385	1.458483586	2.603950853	0.011058082	0.040033391	-3.528449652
ZNF71	-0.151826015	2.979323064	-2.603829778	0.011061665	0.040036742	-3.82870773
POLD2	0.130506583	6.539453317	2.603299474	0.011077372	0.040083963	-4.071567852
ATP5G1	0.153890613	4.489905174	2.603148763	0.01108184	0.0400905	-3.982403323
MRPL22	0.183329499	3.836040438	2.602509541	0.011100808	0.040139838	-3.913666906
RNF185	-0.080761972	5.500636236	-2.602587417	0.011098495	0.040139838	-4.056160165
ZNF845	-0.200168936	2.418996062	-2.602328716	0.011106178	0.040139988	-3.746009705
RASSF1	0.137526897	4.18009197	2.602349669	0.011105556	0.040139988	-3.959416769
XPO4	-0.245399767	4.148267055	-2.601858146	0.011120166	0.040180903	-3.971651009
LOC101928865	-0.8074228	-4.883449387	-2.601303305	0.01113668	0.040211636	-2.779375767
UHRF1BP1	-0.271205066	3.357074721	-2.60136476	0.01113485	0.040211636	-3.860019593
TECR	0.153918503	5.432087656	2.601371679	0.011134644	0.040211636	-4.05246138
ARPC5	-0.101121764	7.008936216	-2.601027844	0.011144886	0.040231625	-4.066451387
CCNO	0.501733943	-1.486947403	2.600679368	0.011155276	0.040259483	-3.026680754
DCTN2	0.092227452	7.630401797	2.600465953	0.011161643	0.040272814	-4.053180597
MCMDC2	-0.265543847	-0.023733078	-2.600271307	0.011167454	0.040284129	-3.225549827
LSM7	0.176475592	4.432170074	2.599086949	0.011202866	0.040402195	-3.990898464
KCTD2	-0.086662124	5.520536308	-2.598834164	0.011210437	0.040419824	-4.073175797
MRPS6	0.133840886	4.812155419	2.598308024	0.011226211	0.040467011	-4.022293631
MFSD2B	0.707616741	-2.302170041	2.597498639	0.011250516	0.040542905	-2.931895586
RMDN3	0.080109778	4.790829481	2.597427707	0.011252648	0.040542905	-4.023641264
C1QTNF1	0.192833431	3.428942222	2.59646411	0.01128165	0.040637681	-3.884119276
BTBD2	0.148207281	7.752510379	2.596334961	0.011285543	0.040641983	-4.05999701
EIF5AL1	0.313711752	1.024538304	2.59578475	0.011302139	0.040672579	-3.447017035
GRN	0.102589311	9.820964147	2.595789951	0.011301982	0.040672579	-3.937930711
MSANTD4	-0.131873679	4.591236125	-2.595802423	0.011301605	0.040672579	-4.025503409
KCNB1	0.387930571	1.59786816	2.594591719	0.0113382	0.040773129	-3.632391589
PSMG1	0.1459884	5.003452083	2.594713878	0.011334502	0.040773129	-4.051141281
FOXJ2	-0.226671903	5.101670683	-2.594613357	0.011337545	0.040773129	-4.066083082
URI1	-0.089483081	6.240158166	-2.59431687	0.011346522	0.040793319	-4.092701759
TRIM27	-0.104564191	5.648169855	-2.594168643	0.011351013	0.040799727	-4.088379575
PIK3IP1	0.14625901	4.790847669	2.593823483	0.011361476	0.040827593	-4.056864204
SPCS2	0.107507271	3.832361087	2.593715072	0.011364764	0.040829669	-3.941262955
NOC3L	-0.163088459	3.951497273	-2.593298672	0.011377401	0.040865325	-4.010410804
TRAPPC1	0.188586744	5.91289207	2.592504099	0.011401551	0.040942305	-4.089101056
CDCA5	-0.210028453	2.44591877	-2.592364757	0.011405791	0.040947769	-3.737479629
IGFBP6	0.247859802	8.281466186	2.591819558	0.011422394	0.040996032	-4.043150842
ENTPD4	-0.157601002	5.151523317	-2.591744641	0.011424678	0.040996032	-4.071282569
CPN2	0.579505208	-2.083185622	2.591410896	0.011434854	0.041022777	-2.995322123

Gene	Log fold change	Average Expression	t	P-value	Adjusted P-value	B
HIBCH	-0.116347221	3.843567779	-2.59105985	0.011445567	0.041051432	-3.924177716
RDH10-AS1	0.994716343	-4.229011684	2.590112077	0.011474535	0.041104235	-2.827478352
ARMC4	-0.396271252	0.269658317	-2.590069048	0.011475851	0.041104235	-3.237263799
ZNF784	0.171995986	2.512503942	2.590098512	0.01147495	0.041104235	-3.689188061
TRIM13	-0.113224692	4.576769283	-2.589953459	0.011479389	0.041104235	-4.041539146
RAB4A	0.071648441	5.272980987	2.590054319	0.011476302	0.041104235	-4.075254601
CBR1	0.182799065	5.715188035	2.590396849	0.011465824	0.041104235	-4.091287744
RNF145	-0.194219386	5.673633291	-2.589969576	0.011478896	0.041104235	-4.098977563
RBMX	-0.080812522	6.323829238	-2.589780488	0.011484685	0.041113425	-4.103753548
UBE2D1	-0.098721081	5.291665848	-2.589264739	0.011500489	0.04116022	-4.079403976
PTGER3	-0.457934868	2.443080773	-2.588872658	0.011512517	0.041186642	-3.710527264
NDUFB10	0.195114445	5.950590586	2.588845832	0.01151334	0.041186642	-4.099018055
NAP1L1	0.071884009	9.258497315	2.588625923	0.011520091	0.04120101	-3.987854627
CLTB	0.18706632	6.02340573	2.588365909	0.011528079	0.04121979	-4.103148717
FUT4	-0.131721806	2.953472036	-2.587791195	0.011545751	0.041273182	-3.798195284
ARF3	0.059730909	7.000143237	2.587038118	0.011568945	0.041346284	-4.102509028
ZNF627	-0.156362703	3.247464017	-2.586483367	0.011586058	0.041386491	-3.897113516
TDP2	0.082504283	5.081066421	2.586406276	0.011588437	0.041386491	-4.073497655
VTA1	-0.065109322	5.782619961	-2.5865133	0.011585134	0.041386491	-4.102769934
CCDC74A	0.176191823	3.22582909	2.582912543	0.011696758	0.041763442	-3.879844682
SLC26A2	-0.269882499	4.756499184	-2.582815763	0.011699771	0.041764302	-4.076498638
HP1BP3	-0.147985857	7.15547024	-2.582647178	0.011705023	0.041773149	-4.097947751
PGGT1B	-0.161922163	3.289454566	-2.581708654	0.011734295	0.041867698	-3.884217204
RPF1	-0.108073263	4.384927498	-2.58107606	0.011754063	0.041928298	-4.035240054
NCAPG	-0.267077286	2.396593345	-2.580875706	0.01176033	0.04192841	-3.741945446
PPIA	0.12475729	6.187815449	2.580928041	0.011758693	0.04192841	-4.124853122
RAB18	-0.077327941	6.441823098	-2.580808137	0.011762444	0.04192841	-4.125416632
LOC100506688	-0.844583505	-4.49946382	-2.580131204	0.011783645	0.041975633	-2.832106485
SPRYD4	0.169386781	2.690349339	2.579685347	0.011797627	0.041975633	-3.793565791
IGIP	0.195610409	3.487007047	2.580114654	0.011784164	0.041975633	-3.952046038
UBA52	0.171769459	9.21063427	2.579874307	0.011791699	0.041975633	-4.018503999
CLPP	0.194960135	5.109685568	2.579896558	0.011791002	0.041975633	-4.080934082
TMEM87A	-0.101741482	5.537191058	-2.579734401	0.011796088	0.041975633	-4.110493068
SNX17	0.094099861	7.333446678	2.58014589	0.011783185	0.041975633	-4.111769979
ARHGAP31	-0.201720587	5.436060015	-2.579673976	0.011797984	0.041975633	-4.114814744
CTC1	0.140214092	2.69420865	2.579117757	0.011815449	0.042027845	-3.767014593
PAFAH2	0.148254299	3.022976839	2.5785082	0.011834616	0.042069641	-3.847929396
FADS3	0.151723449	5.010073294	2.578477636	0.011835577	0.042069641	-4.087733476
UQCR10	0.167944392	5.268442553	2.578600642	0.011831707	0.042069641	-4.094673069
VPS53	0.085969697	5.168958353	2.577985601	0.01185107	0.042114773	-4.107159982
KIRREL	-0.340972412	7.375393847	-2.577660086	0.01186133	0.042141291	-4.109399808
CCDC122	-0.254242228	1.005425036	-2.577565699	0.011864306	0.042141927	-3.408951451
AES	0.122309137	7.556180558	2.577316521	0.011872167	0.042159907	-4.111199447
KLF15	-0.330461841	1.261396025	-2.576677401	0.011892351	0.042212863	-3.369805974
FBXO18	-0.062419373	6.084524858	-2.576666873	0.011892684	0.042212863	-4.131693197
ARIH2	-0.140528836	5.490245573	-2.576304705	0.011904136	0.042233608	-4.119849565
APOL6	-0.143661698	6.030459816	-2.576323121	0.011903553	0.042233608	-4.131484278
LOC148413	0.163954622	2.695672297	2.575742979	0.011921917	0.042286734	-3.870491905
IFITM3	0.192238773	9.165639612	2.575320101	0.011935319	0.042324305	-4.033562723
RHOT2	0.135392888	4.541572692	2.574807641	0.011951578	0.042352053	-4.0557276
CLIP3	0.169169248	7.815117032	2.574878558	0.011949327	0.042352053	-4.10745962
DPY19L4	-0.243821249	5.165108758	-2.574858016	0.011949979	0.042352053	-4.11294319
MAPK8	-0.111439938	3.963004281	-2.574494034	0.011961538	0.042367414	-3.994293922
FAM222B	-0.107285189	4.066181935	-2.574506654	0.011961137	0.042367414	-4.036549165
IGFBP4	0.134061472	10.86267061	2.573667296	0.011987831	0.042450561	-3.92086897
VDAC3	0.098271392	5.922932793	2.573455622	0.011994571	0.042454469	-4.136541553
PTPRK	-0.258140393	6.100313097	-2.573517103	0.011992613	0.042454469	-4.141790358
GLRX	-0.19230367	4.885567713	-2.573168584	0.012003717	0.04246267	-4.069685989
SLC35C1	-0.077584383	5.621359918	-2.573117511	0.012005345	0.04246267	-4.128866336
CETN2	0.123963444	6.15493909	2.57314271	0.012004541	0.04246267	-4.142400607
VPS11	0.073146551	5.905180366	2.57282463	0.012014684	0.042485728	-4.137916218
OTUD7B	-0.252118968	4.921651664	-2.571921749	0.012043516	0.042558163	-4.107895926
MED13	-0.323904087	5.651176333	-2.571917762	0.012043644	0.042558163	-4.136954414
GLMP	0.08948665	6.239489648	2.572012644	0.012040611	0.042558163	-4.146108268
MFAP2	0.110296242	7.0229039	2.571828944	0.012046483	0.042558214	-4.139923622
CACNA2D1	-0.30733038	4.693080875	-2.571250457	0.012064993	0.042613613	-4.098484588
TIMM50	0.084996007	5.482383559	2.57082912	0.012078491	0.042651288	-4.131782562
LRRC34	-0.226692978	0.552354786	-2.570515454	0.012088549	0.042676798	-3.387346376
SERINC3	-0.107269146	7.352570452	-2.57005875	0.012103206	0.042718532	-4.130449602
SEMA3G	-0.700619778	-2.829419254	-2.569779013	0.012112192	0.042740234	-2.974400808

Gene	Log fold change	Average Expression	t	P-value	Adjusted P-value	B
ZNF25	-0.135252133	3.442108039	-2.568922304	0.012139749	0.042827441	-3.943544822
CTNNA1	0.063421145	8.574290467	2.568705961	0.012146716	0.042840219	-4.072121028
RAD23B	-0.071492248	7.968297492	-2.568633268	0.012149058	0.042840219	-4.108006415
NCAPH2	0.134628851	4.762118974	2.56814152	0.012164912	0.042886085	-4.093485227
ZMPSTE24	-0.130598162	6.665082375	-2.567286261	0.012192531	0.042973393	-4.156129128
PDXP	-0.416722641	-1.343034526	-2.566139997	0.012229635	0.043073935	-3.133301208
RAB29	-0.101478988	5.312299533	-2.566157853	0.012229056	0.043073935	-4.124989144
BMPER	-0.226052608	5.999203168	-2.566194345	0.012227873	0.043073935	-4.156990015
SGO1	-0.428400491	-0.79043992	-2.565910532	0.012237075	0.043090064	-3.197106103
TLK2	-0.11414626	4.02110149	-2.565734488	0.012242786	0.043099186	-4.040668747
DVL1	0.123959654	5.613867365	2.565654308	0.012245387	0.043099186	-4.14218197
RPL8	0.168722318	10.40382548	2.565488455	0.012250771	0.043108061	-3.977582473
SIPA1L3	0.15251436	4.251695845	2.565348915	0.012255301	0.043113933	-4.065273762
METRN	0.172764753	5.39158978	2.564616117	0.01227912	0.043187642	-4.139405137
PHC3	-0.279612784	4.593671349	-2.563802458	0.012305617	0.043270731	-4.107722952
GABRB2	-0.903406055	-4.423876029	-2.5636049	0.012312058	0.043283276	-2.872184688
HINT1	0.152561507	6.903215574	2.563140487	0.012327211	0.043326438	-4.163589987
ITSN1	-0.195732866	5.667732137	-2.562623785	0.012344091	0.043375644	-4.15721772
IL18R1	-0.221806757	2.705082479	-2.562336371	0.012353489	0.043398545	-3.83931101
STRN	-0.230890208	5.105904863	-2.561874741	0.012368598	0.043441491	-4.144817059
IER3	0.298468814	4.822627109	2.561692106	0.01237458	0.04345237	-4.033058025
TMEM192	-0.189288894	4.468372649	-2.561600856	0.012377569	0.04345274	-4.090989836
STK32C	0.188903527	3.431267426	2.561292689	0.012387671	0.043478071	-3.945714152
LINC00641	-0.310368824	1.937020656	-2.560605366	0.012410229	0.043539309	-3.725192309
TTLL5	-0.100331378	4.577309846	-2.56042436	0.012416175	0.043539309	-4.085347348
PRAF2	0.177067269	4.728969012	2.560422526	0.012416236	0.043539309	-4.105714835
MMADHC	0.073808085	6.259991482	2.560408989	0.01241668	0.043539309	-4.172272288
KIF6	-0.317807835	0.30092187	-2.560254507	0.012421758	0.043546977	-3.345015237
NUP160	-0.15431818	5.196145969	-2.560038902	0.012428847	0.04355156	-4.146100772
GNAI3	-0.182070878	6.910862739	-2.56004053	0.012428794	0.04355156	-4.167653267
EPHA1	-0.511937443	-1.177629273	-2.559472654	0.012447484	0.043606722	-3.120922475
GLUD1	0.079352516	6.822119824	2.558926543	0.012465482	0.043649471	-4.173950171
UTRN	-0.330230448	6.621552068	-2.558966089	0.012464178	0.043649471	-4.174327653
CRACR2A	-0.171784511	2.118708557	-2.558650076	0.012474603	0.043671256	-3.754058121
ZNF311	0.456723481	-0.661301942	2.558402361	0.01248278	0.04367958	-3.247532118
SNX4	-0.126917882	4.15356101	-2.558411236	0.012482487	0.04367958	-4.068317989
IMP4	0.106616351	5.325369401	2.557883147	0.012499935	0.04372945	-4.160367705
LMCD1-AS1	-0.422726606	-1.909489508	-2.557522571	0.01251186	0.043730549	-3.055316106
CEBPG	-0.171335882	4.967368268	-2.557620261	0.012508628	0.043730549	-4.091402824
ZNF213	0.129563604	4.585731165	2.557724513	0.01250518	0.043730549	-4.114504438
KIAA1217	-0.187038538	4.971694137	-2.55757625	0.012510084	0.043730549	-4.137740139
PLAC9P1	0.630921736	-2.279314534	2.557089416	0.012526201	0.043770514	-3.019241482
THAP6	0.155733756	2.711114022	2.556954223	0.012530679	0.043776009	-3.85958534
PKIA	0.17188504	2.611278688	2.55673984	0.012537784	0.043790676	-3.822442791
ZNF160	-0.187411948	4.132474462	-2.556581091	0.012543048	0.043798905	-4.090584875
MX1	0.284257569	4.896320685	2.555673478	0.01257318	0.043883778	-4.142644295
SRSF1	-0.0578601	6.552949731	-2.55570302	0.012572198	0.043883778	-4.18452557
SETX	-0.279278866	6.87172435	-2.555434154	0.012581137	0.043901376	-4.177478305
NT5C	0.202718035	3.082237805	2.554903764	0.012598785	0.04395278	-3.911656808
PRDX3	0.059998479	6.452079505	2.554480023	0.012612902	0.043991838	-4.188414203
PQLC1	0.099873557	5.9571085	2.553788049	0.012635984	0.044062145	-4.185874958
MSRA	0.118486902	3.584022705	2.553055449	0.012660463	0.044137288	-4.012843921
SCLT1	-0.142297094	2.588704384	-2.552863683	0.012666878	0.044149434	-3.79846083
ST3GAL5	-0.151097832	4.973827767	-2.552673802	0.012673233	0.044151152	-4.153396526
TOP1	-0.171913446	6.21002974	-2.552751174	0.012670643	0.044151152	-4.191784441
CEMIP	-0.530008681	8.931976953	-2.55192621	0.01269828	0.044228183	-4.070818447
CCDC8	-0.16363176	5.829093424	-2.551645412	0.012707699	0.044250759	-4.193783425
FAR1	-0.164225595	5.274114226	-2.551261603	0.012720584	0.04428539	-4.175969943
LCAT	0.392713009	1.53497235	2.550654822	0.012740979	0.044346143	-3.658186317
LMO2	0.290218058	1.464042662	2.550216373	0.012755734	0.044387243	-3.654868862
REEP6	0.188794075	2.35526386	2.549847131	0.012768172	0.04441645	-3.789547895
CCDC149	-0.087746121	4.969666061	-2.549777678	0.012770513	0.04441645	-4.153449113
MYO18A	0.190843706	5.548280822	2.549529807	0.01277887	0.04441645	-4.177728866
UBL7	0.149876827	5.555766562	2.549570303	0.012777504	0.04441645	-4.178588625
TRAF7	0.075646514	7.24958236	2.549675048	0.012773972	0.04441645	-4.187376266
NPC2	0.139980924	7.741656996	2.549045473	0.012795214	0.044462999	-4.16968922
CNIH2	0.481968133	-1.616298323	2.548063841	0.012828397	0.044568028	-3.115416719
METTL5	0.176649441	4.027946955	2.547965075	0.01283174	0.044569363	-4.037267746
C16orf72	-0.213863795	5.69253131	-2.547858645	0.012835343	0.044571602	-4.189383815
HES4	0.545450585	-1.997259102	2.547077448	0.012861819	0.044644878	-3.073831268

Gene	Log fold change	Average Expression	t	P-value	Adjusted P-value	B
ENPP4	-0.398397024	1.289159491	-2.547061149	0.012862372	0.044644878	-3.612820036
PEX26	-0.083243957	5.514784116	-2.546839553	0.012869891	0.044660688	-4.190829244
LYPLA1	-0.1851503	3.990534636	-2.546153494	0.012893197	0.044731257	-4.082052171
STAT3	-0.178950786	7.741881238	-2.545909797	0.012901484	0.044749703	-4.164395305
PIP5K1A	-0.19995231	5.240849492	-2.544977035	0.01293325	0.044849557	-4.158173749
ORMDL3	0.093502523	5.378545878	2.544705034	0.012942526	0.044871395	-4.187522781
CTGF	-0.313988346	8.199047148	-2.543496219	0.012983824	0.045004217	-4.200887659
TIMM17B	0.209863487	3.43209582	2.543402679	0.012987025	0.045004955	-3.983782232
MAVS	-0.156301732	6.665097431	-2.54297941	0.013001517	0.045044813	-4.214574463
CSNK1E	-0.08634708	6.081231438	-2.542849178	0.013005978	0.04504991	-4.211841315
UGDH-AS1	-0.331562197	-0.385261103	-2.54244297	0.013019904	0.045056434	-3.286314402
HDAC10	0.355051744	-0.315759785	2.542336916	0.013023542	0.045056434	-3.327147875
CPEB1	0.173168964	2.935544899	2.542572954	0.013015447	0.045056434	-3.990789927
PRR14L	-0.22481972	4.074871247	-2.542270879	0.013025808	0.045056434	-4.122744228
ANXA7	0.085914501	7.115400192	2.542438705	0.01302005	0.045056434	-4.205464851
ZNHIT1	0.194204059	5.977872459	2.54242097	0.013020659	0.045056434	-4.210041411
LSM2	0.163097246	4.851623286	2.542151625	0.0130299	0.045060243	-4.147241833
C17orf53	0.199836551	1.180980984	2.541692653	0.013045662	0.045104396	-3.67086173
PRPF38B	-0.160694563	4.779239809	-2.5413227	0.013058379	0.045138005	-4.169668504
NUDT6	0.278215417	-0.065298233	2.540518581	0.01308606	0.045223308	-3.368310257
FAM126B	-0.183326132	2.705849189	-2.539583786	0.013118305	0.045324344	-3.950299651
RPL24	0.183448445	8.100812332	2.538849617	0.013143679	0.045401602	-4.177911932
PPA1	0.109931866	6.329201697	2.538141144	0.013168208	0.045475903	-4.227029453
ZFAND2A	0.224141956	2.904871642	2.537992675	0.013173354	0.045483245	-3.929457266
B3GNTL1	0.276532176	0.830696412	2.537705651	0.013183306	0.045507178	-3.521305909
CARNS1	0.640558037	-2.124066966	2.537497243	0.013190537	0.045521706	-3.106940836
SURF6	-0.114800058	4.954363541	-2.537094144	0.013204533	0.04555957	-4.198647773
DEK	-0.155451697	6.732746294	-2.53699127	0.013208107	0.045561465	-4.225802574
VLDLR	-0.199288054	3.72814129	-2.536865797	0.013212467	0.045566072	-4.059358905
NICN1	-0.15585006	3.400249789	-2.536306845	0.013231907	0.045622669	-4.024660191
DHX34	-0.131475842	3.673246655	-2.536165474	0.013236828	0.045629192	-4.066402857
ZNF253	-0.195533021	1.851385379	-2.535504823	0.013259845	0.04567891	-3.878041511
MAP4	0.086700146	9.383899055	2.535664107	0.013254292	0.04567891	-4.105144885
CBLL1	-0.133185144	4.959081548	-2.535489227	0.013260389	0.04567891	-4.185478856
GAA	0.102226644	8.028909996	2.535403466	0.01326338	0.04567891	-4.18638634
RPL18	0.185432954	9.414735788	2.535136753	0.013272685	0.045700508	-4.112127433
MTIF2	0.10488389	4.822285297	2.534328156	0.013300931	0.045787299	-4.170055874
ADRA2C	0.304749535	0.042012378	2.533734007	0.01332172	0.045848388	-3.436995429
MCOLN1	0.131706631	4.626366286	2.533467729	0.013331047	0.045870007	-4.142459401
NT5M	0.222347753	1.053691439	2.532978158	0.01334821	0.045918573	-3.566633517
PSMB7	0.137980929	7.081032802	2.53276073	0.013355839	0.045934327	-4.232245452
LRIG2	-0.202893671	3.033187719	-2.532599759	0.013361489	0.045943271	-3.981852158
C9orf85	0.107209543	3.419002233	2.532416511	0.013367924	0.045954909	-4.024784175
AP3M1	-0.152210372	5.404969614	-2.531936239	0.013384803	0.046002435	-4.220806321
IL22RA1	0.560357377	-1.370284567	2.531407208	0.013403418	0.046040192	-3.327671285
TMOD2	-0.211290425	3.950830814	-2.531363469	0.013404958	0.046040192	-4.092933374
ATP5J	0.159925668	5.562905355	2.531509347	0.013399822	0.046040192	-4.225803472
E2F1	-0.235161675	2.333440655	-2.530924014	0.01342044	0.046082859	-3.808253034
CDKN2B	0.153510048	4.464495356	2.529762023	0.013461455	0.046213161	-4.126090647
TMEM102	0.305733194	-0.124321669	2.528355455	0.013511253	0.046365276	-3.442426479
RBMS3	0.123299437	5.707183789	2.528336662	0.01351192	0.046365276	-4.23587241
LOC105370333	0.290402885	0.513098925	2.527408956	0.013544857	0.046467713	-3.455716407
SNRPD2	0.182908648	7.02033634	2.527269334	0.01354982	0.046474156	-4.247464402
ACVR2A	-0.227454988	5.348943487	-2.526343877	0.013582761	0.046576532	-4.206954943
TTLL11	0.145609245	2.453445363	2.526234416	0.013586662	0.046579306	-3.915041472
BTBD11	0.569117848	-1.230818602	2.525849865	0.013600374	0.046606128	-3.209364209
TRPC4	-0.241393335	2.110136071	-2.525581383	0.013609955	0.046606128	-3.856919477
NUDT1	0.194758788	3.741014897	2.525631436	0.013608168	0.046606128	-4.077418611
BRI3	0.129699869	6.961543268	2.525823643	0.013601309	0.046606128	-4.250897881
CIAO1	0.070509981	5.874794124	2.525629308	0.013608244	0.046606128	-4.251684643
TMEM80	-0.154194697	3.218623909	-2.525471494	0.013613878	0.046608967	-4.001255183
SNRNP25	0.170378584	3.891498018	2.525309458	0.013619665	0.046618184	-4.102753329
FRMD3	0.421015842	-0.855067133	2.524428537	0.013651163	0.046704775	-3.288689268
PSENEN	0.20272464	3.501527282	2.524483993	0.013649179	0.046704775	-4.05178337
CRTAP	0.061959727	9.390842627	2.524254883	0.01365738	0.046715436	-4.132344131
HEBP1	0.123210371	5.867011892	2.524137685	0.013661578	0.046719185	-4.252447049
S100A6	0.205997188	10.42415569	2.523946827	0.013668416	0.046731959	-4.073243559
TMA7	0.206367675	2.389096935	2.523462961	0.013685765	0.046780658	-3.881634916
ZNF230	-0.180201315	1.889952638	-2.52294813	0.013704246	0.046833203	-3.864870337
VBP1	0.079881125	5.222620627	2.522234167	0.013729913	0.046896564	-4.23554667

Gene	Log fold change	Average Expression	t	P-value	Adjusted P-value	B
CPQ	0.064990951	6.857697059	2.522172656	0.013732126	0.046896564	-4.260486987
PRKAR2A	-0.254603232	6.146498597	-2.522313387	0.013727062	0.046896564	-4.26373928
PSMC5	0.168091961	6.591367609	2.522065924	0.013735967	0.04689905	-4.264802779
RNF8	0.095611673	4.228875717	2.521707449	0.013748876	0.046932486	-4.179676095
RSPH3	-0.216811348	3.150290228	-2.521445297	0.013758322	0.046947233	-4.008480139
UBXN1	0.149211713	6.981205925	2.521414607	0.013759429	0.046947233	-4.261851176
FOXL2	0.341903672	1.371314136	2.521254289	0.013765209	0.04695632	-3.763487439
DNAJC30	0.16294948	3.736904803	2.521035733	0.013773093	0.046961945	-4.092294026
ARPC3	0.131334877	6.053466746	2.521109015	0.013770449	0.046961945	-4.26455884
STIM2	-0.114684213	3.559695884	-2.520743764	0.013783631	0.046987242	-4.095419794
CS	0.078453069	6.676172259	2.52024548	0.013801633	0.047037965	-4.268033061
ECHDC2	0.177236374	3.065264841	2.520055664	0.013808497	0.047050711	-4.015740173
ABCC9	-0.219028176	5.687433054	-2.519923701	0.01381327	0.047056331	-4.261671039
PYROXD1	-0.26513736	1.957260823	-2.519375039	0.013833131	0.047102688	-3.74565922
FBXW4	0.132168339	4.89936357	2.519425409	0.013831307	0.047102688	-4.210171244
PSMB8	0.169402146	5.531934016	2.519251171	0.013837619	0.04710732	-4.250718245
MYPOP	0.14892369	2.752010157	2.51909119	0.013843417	0.04711641	-4.002517746
CLCN6	-0.151426463	4.558957491	-2.518475474	0.013865751	0.047181765	-4.202709171
C9orf40	-0.12600543	3.43682433	-2.518033803	0.013881792	0.047210455	-4.055042687
RPL37A	0.176757806	9.724348599	2.518045337	0.013881373	0.047210455	-4.134507382
GABPB1	-0.101312386	4.623964466	-2.517984486	0.013883584	0.047210455	-4.202344494
GPRASP1	-0.156505224	2.519533456	-2.517545023	0.013899564	0.047252201	-3.91917872
MORC3	-0.262868854	4.43008486	-2.517474361	0.013902135	0.047252201	-4.19698642
INTS9	0.098516804	3.83151495	2.516837018	0.013925342	0.047320405	-4.114934463
ISOC2	0.171755804	5.19888112	2.516662112	0.013931717	0.047331391	-4.243150906
ATP5G3	0.123415737	6.610447687	2.516453719	0.013939316	0.04734653	-4.277428497
NOP56	-0.126195185	6.216329045	-2.516238779	0.013947158	0.047362486	-4.278374176
GNAQ	-0.179618692	5.846869617	-2.516103196	0.013952106	0.047368612	-4.273933578
FAM189B	0.121182556	5.966650076	2.515360732	0.013979233	0.047450014	-4.276180405
NDUFV1	0.143831068	6.424831825	2.5151887	0.013985525	0.047460677	-4.281018523
DGCR8	-0.094950455	4.832418643	-2.514500236	0.014010731	0.047535506	-4.236484093
YBX1	0.153201439	8.676138094	2.51432523	0.014017145	0.047546558	-4.203021686
MBLAC1	0.359952273	0.012550015	2.514203329	0.014021614	0.047551011	-3.453870975
ADGRF3	0.480451269	-1.513957794	2.513898644	0.01403279	0.0475782	-3.202901415
STARD8	-0.235273934	1.813538951	-2.513550278	0.014045578	0.047610842	-3.823811015
MVB12A	0.162007253	4.951337045	2.513068755	0.014063271	0.047649374	-4.228566655
CAND1	-0.23638996	6.948256362	-2.513151287	0.014060237	0.047649374	-4.27544389
YTHDC2	-0.177943826	3.948452168	-2.512862516	0.014070855	0.04766435	-4.187289957
SLC1A3	-0.157913698	6.279002073	-2.512768416	0.014074317	0.047665358	-4.28552023
TBCB	0.178323887	5.68216582	2.512627741	0.014079493	0.047672171	-4.272644807
GOSR2	-0.087940644	3.680019556	-2.512333457	0.014090328	0.047698135	-4.131379757
ZBTB48	0.168372839	2.385115124	2.512058141	0.014100471	0.047721746	-3.90010637
PDF	0.31698628	-0.224981461	2.511521522	0.014120259	0.047777984	-3.43516218
EXD2	-0.138646188	3.877783352	-2.5114069	0.014124489	0.047781564	-4.11850289
CLCN7	-0.107081836	6.699298792	-2.51050916	0.014157659	0.047883021	-4.291326395
SGCE	0.076800872	5.980695526	2.51029929	0.014165423	0.047898527	-4.289407248
NAA10	0.207808772	4.325545354	2.510004788	0.014176325	0.047924633	-4.184804285
KLC1	0.153773499	3.932627554	2.509847997	0.014182133	0.047933508	-4.133881675
ISPD	-0.247535535	0.838339361	-2.509404969	0.014198553	0.047956727	-3.594088169
LMF1	0.134922716	4.432793716	2.509434969	0.014197441	0.047956727	-4.201921224
RBBP9	-0.219203776	4.646566607	-2.509411198	0.014198322	0.047956727	-4.226991238
CHM	-0.268583104	4.497411197	-2.508665228	0.014226009	0.04803869	-4.218898403
ACSL3	-0.21792046	6.830336771	-2.508496417	0.014232281	0.0480491	-4.28359976
CAPG	0.179938623	7.548428376	2.508280057	0.014240324	0.04806548	-4.274953278
IRAK4	-0.08991329	4.095342833	-2.50818674	0.014243794	0.048066422	-4.165303901
RASEF	-0.425138826	-0.655050916	-2.507849729	0.014256332	0.048069041	-3.354869637
SNAPC1	-0.125777025	3.179940536	-2.507742835	0.014260311	0.048069041	-4.01299472
LATS1	-0.287682934	4.611000804	-2.507967012	0.014251967	0.048069041	-4.233388955
EPN1	0.131957912	7.833219788	2.50773709	0.014260525	0.048069041	-4.264702656
KAT6A	-0.343668655	5.818159351	-2.508073451	0.014248007	0.048069041	-4.293120616
ABCB5	-0.678604524	-3.081314868	-2.507310429	0.014276417	0.048101424	-3.056805479
LINC02407	-0.572207682	-1.823269183	-2.507307717	0.014276518	0.048101424	-3.190733732
ATXN2	-0.136468812	4.782994952	-2.506845062	0.014293769	0.048148777	-4.259341125
ACVR1	-0.28359213	6.150111912	-2.506566202	0.014304176	0.048173061	-4.299917799
USF3	-0.168391619	3.280548645	-2.506472198	0.014307685	0.048174111	-4.091070202
C8orf82	0.149264741	4.34838493	2.505830092	0.014331679	0.048244116	-4.195670649
CLCN5	-0.277501518	3.696393724	-2.50549258	0.014344306	0.048256875	-4.167523328
SLC27A3	0.159555551	4.415531902	2.505618705	0.014339586	0.048256875	-4.191487243
MCRIP1	0.144820252	6.959639111	2.505471894	0.01434508	0.048256875	-4.299215879
ZNF704	-0.226686837	4.015356885	-2.505225372	0.014354309	0.048277142	-4.159336474

Gene	Log fold change	Average Expression	t	P-value	Adjusted P-value	B
RNLS	0.120147489	3.128304428	2.504854213	0.014368215	0.048303795	-4.054950194
EMC8	0.11702941	4.59141697	2.504842679	0.014368647	0.048303795	-4.237694636
BDP1	-0.252678479	3.938005657	-2.504670312	0.014375109	0.048314736	-4.175698021
TBCA	0.159042416	5.49689862	2.504429904	0.014384127	0.048334259	-4.285465914
DDIT3	0.299994715	3.664493537	2.50422496	0.014391818	0.048348468	-4.13358015
NHSL2	-0.195890088	5.86352157	-2.50414621	0.014394774	0.048348468	-4.303090834
RBP4	0.830094138	-3.069387433	2.504023017	0.0143994	0.048351974	-3.067590477
PIGV	-0.101774256	3.860513859	-2.503947463	0.014402238	0.048351974	-4.124683585
SULT4A1	0.554446203	-1.714419821	2.503810127	0.014407397	0.048358518	-3.203667734
ZDHHC23	-0.284380484	0.236032548	-2.503627952	0.014414243	0.048359947	-3.537846881
HBS1L	-0.070105128	5.566933372	-2.503658386	0.014413099	0.048359947	-4.294524498
ANKRD55	-0.868378387	-2.669160837	-2.503392094	0.014423112	0.048378925	-3.086449562
PSMA4	0.10237733	6.54177769	2.503182584	0.014430993	0.048394587	-4.308919393
DENND6B	0.15568858	2.663285736	2.502905604	0.014441419	0.048418771	-3.960056448
RASL11A	0.30691654	1.14694813	2.501985115	0.014476116	0.048513507	-3.650197203
HDGF	0.072891019	8.074312713	2.502066772	0.014473035	0.048513507	-4.259773069
MAP3K5	-0.225007473	3.394716865	-2.501491467	0.014494754	0.048554365	-4.117397367
ST13	0.06540283	7.27442541	2.50150733	0.014494155	0.048554365	-4.296991006
UBXN2B	-0.140928222	3.629316271	-2.500735642	0.014523333	0.048639283	-4.120171797
IFT22	0.120294712	5.103221969	2.500322915	0.014538961	0.048680797	-4.270427147
PSME2	0.163160384	4.989800852	2.500028982	0.014550099	0.048707266	-4.269213901
NGFR	0.801792639	-2.578505231	2.499868192	0.014556196	0.048716437	-3.126690858
CYP7B1	-0.449782391	2.45148605	-2.499765361	0.014560096	0.048716437	-3.96685102
HADHA	0.074819095	8.230956845	2.499625796	0.014565391	0.048716437	-4.259998214
ENY2	0.122362256	5.08819758	2.49961567	0.014565775	0.048716437	-4.28458919
TP53TG1	0.20321645	2.081058242	2.499423054	0.014573085	0.048730068	-3.85683311
DGCR14	0.124733222	4.350541158	2.49891698	0.014592308	0.048783517	-4.237839188
EIF4A2	0.073233769	7.535242812	2.49826883	0.014616961	0.048855091	-4.295668723
GACAT2	0.348890561	1.823421738	2.498120189	0.01462262	0.048863164	-3.797740917
CCDC22	0.116631668	4.467528931	2.49738875	0.014650495	0.048945455	-4.227482276
APH1B	-0.140952803	4.259826899	-2.497272445	0.014654932	0.048949422	-4.206280087
SMAD7	0.135930359	4.36427296	2.496595955	0.014680763	0.04902483	-4.164592483
LOC101927497	-0.931138772	-3.51551578	-2.496338291	0.014690612	0.04903598	-3.044254463
PTPRS	-0.151716437	6.119778719	-2.496384858	0.014688832	0.04903598	-4.321388649
XXYLT1-AS2	0.86203734	-3.280312121	2.495969391	0.014704724	0.04907221	-3.056114972
ZNF549	-0.216286946	2.372045033	-2.495400267	0.014726518	0.049123178	-3.99205089
DKK2	-0.384406013	2.924007837	-2.495454157	0.014724453	0.049123178	-4.059644703
THAP1	-0.141392274	3.211325032	-2.495301656	0.014730298	0.049124907	-4.044581595
GLRX5	0.139255143	4.935127364	2.495070501	0.01473916	0.049143583	-4.277628887
HSPH1	0.152440458	6.855993433	2.494690551	0.014753738	0.049181303	-4.318680338
ZNF14	0.120342185	2.261855996	2.494028853	0.014779156	0.049255135	-3.968836231
HNRNPM	0.078717347	7.264959329	2.493522565	0.014798632	0.049309132	-4.314347853
KNTC1	-0.247755753	2.54666427	-2.493349224	0.014805305	0.049320458	-3.994461744
C18orf32	0.208559733	1.267396826	2.493183088	0.014811703	0.049330863	-3.750263255
CCDC148	-0.508008337	-1.626788976	-2.492754807	0.014828209	0.049369859	-3.223164574
C5	-0.182788045	3.293830933	-2.492709224	0.014829966	0.049369859	-4.101704444
PLOD3	0.099990053	7.662573926	2.492423292	0.014840996	0.049395662	-4.304731525
GTF3C6	0.154336591	4.796403354	2.491977052	0.014858225	0.049442081	-4.272781887
PPIH	0.17558834	3.156175763	2.491751518	0.014866939	0.049460153	-4.081129952
ZNF440	-0.227504918	2.472412604	-2.491047184	0.014894183	0.049507056	-4.032796756
TELO2	0.130955472	4.576867282	2.491110084	0.014891749	0.049507056	-4.251505247
TERF2	-0.086339539	4.457008254	-2.491055686	0.014893854	0.049507056	-4.257704296
GPC6	-0.500979167	5.082468865	-2.491163816	0.014889669	0.049507056	-4.309445148
BLOC1S6	-0.067270978	6.245480963	-2.490678698	0.014908455	0.049543561	-4.337839682
NEK2	-0.336466012	0.921078728	-2.490548747	0.01491349	0.049549365	-3.60940598
MINDY3	0.094661907	4.385911066	2.490230037	0.014925847	0.049572338	-4.226560645
SEC24D	-0.164979307	7.362345681	-2.490200673	0.014926986	0.049572338	-4.307505435
PPP4R4	-0.390130892	-0.693124519	-2.489436625	0.01495665	0.049659901	-3.381931987
ZNF585B	0.145334791	2.722905975	2.489075161	0.014970701	0.049695603	-4.038070013
MOSPD3	0.154660397	3.96417978	2.488896047	0.014977669	0.049707778	-4.192950675
JTB	0.128606309	6.727679986	2.48879477	0.01498161	0.049709905	-4.341456791
PRRC2C	-0.340808357	7.182401155	-2.487468725	0.015033295	0.049870415	-4.328782723
