# Supplementary material for: Comparison of time and dose dependent gene expression and affected pathways in primary human fibroblasts after exposure to ionizing radiation
Source: Mol Med. 2020 Sep 9;26:85. doi: 10.1186/s10020-020-00203-0 (PMC7488023; doi:10.1186/s10020-020-00203-0)
Supplement: Supplementary file 12 — Additional file 12: Web Table 2. Differential expression activity in cellular pathways and involved molecules [file 10020_2020_203_MOESM12_ESM.pdf]

Web Table 2: Differential expression activity in cellular pathways and involved molecules

Ingenuity Canonical Pathways	Model	-log(p-value)	Ratio	z-score	Molecules
14-3-3-mediated Signaling	0.05Gy 2h	0.41	0.02	NaN	CDKN1B
14-3-3-mediated Signaling	0.05Gy 4h	0.76	0.08	0.00	FOS,PRKCD,PRKCI,STK11
14-3-3-mediated Signaling	2Gy 2h	0.52	0.17	-0.38	CDKN1B,ELK1,MAPK8,PIK3CB,PIK3CD,PRKCE,RRAS2,TUBB2A,TUBB4B
**14-3-3-mediated Signaling**	**2Gy 4h**	**1.37**	**0.37**	**-0.24**	**AKT1,ELK1,FOS,GSK3B,HRAS,KRAS,MAP2K1,MAPK8,PIK3CB,PIK3CD,PRKCD,PRKCE,PRKCI,PRKD3,RRAS2,SRC,STK11,TUBB2A,YAP1**
2-ketoglutarate Dehydrogenase Complex	2Gy 4h	0.60	1.00	NaN	DLD
2-oxobutanoate Degradation I	2Gy 4h	0.60	1.00	NaN	DLD
3-phosphoinositide Biosynthesis	0.05Gy 2h	0.65	0.04	NaN	SOCS3
3-phosphoinositide Biosynthesis	0.05Gy 4h	0.50	0.07	NaN	CDC25C,PTPN13
3-phosphoinositide Biosynthesis	2Gy 2h	0.77	0.22	-1.63	CDC25B,DUSP1,PIK3CB,PIK3CD,PTPN2,SOCS3
3-phosphoinositide Biosynthesis	2Gy 4h	1.28	0.41	0.30	CDC25B,DUSP1,NUDT11,PAWR,PIK3CB,PIK3CD,PPP1CA,PPP3CA,PTEN,PTPN13,SOCS3
3-phosphoinositide Degradation	0.05Gy 2h	0.75	0.05	NaN	SOCS3
3-phosphoinositide Degradation	0.05Gy 4h	0.66	0.10	NaN	CDC25C,PTPN13
3-phosphoinositide Degradation	2Gy 2h	0.47	0.19	-1.00	CDC25B,DUSP1,PTPN2,SOCS3
3-phosphoinositide Degradation	2Gy 4h	1.25	0.43	0.33	CDC25B,DUSP1,NUDT11,PAWR,PPP1CA,PPP3CA,PTEN,PTPN13,SOCS3
4-1BB Signaling in T Lymphocytes	2Gy 2h	0.63	0.25	NaN	IKBKB,IKBKE,MAPK8
4-1BB Signaling in T Lymphocytes	2Gy 4h	0.80	0.42	-1.00	ATF2,MAP2K1,MAPK8,NFKB1,NFKBIA
Acetone Degradation I (to Methylglyoxal)	2Gy 4h	0.00	0.06	NaN	CYP1B1
Acetyl-CoA Biosynthesis I (Pyruvate Dehydrogenase Complex)	2Gy 4h	0.60	1.00	NaN	DLD
Actin Cytoskeleton Signaling	0.05Gy 4h	0.43	0.05	NaN	ACTA2,FGF5,RHOA,VAV2
Actin Cytoskeleton Signaling	2Gy 2h	0.66	0.18	-1.00	ACTA2,CDC42,FGF2,FGF7,FGF9,ITGA5,LIMK2,PDGFA,PIK3CB,PIK3CD,RAC1,RRAS2,SOS1
Actin Cytoskeleton Signaling	2Gy 4h	0.73	0.30	0.89	ACTA2,BCAR1,CFL1,CRK,EZR,FGF2,FGF5,HRAS,KRAS,LIMK2,MAP2K1,PFN1,PIK3CB,PIK3CD,RAC3,RHOA,ROCK1,ROCK2,RRAS2,SLC9A1,TMSB10/TMSB4X,VAV2
Actin Nucleation by ARP-WASP Complex	0.05Gy 4h	0.55	0.08	NaN	RHOA,RHOG
**Actin Nucleation by ARP-WASP Complex**	**2Gy 2h**	**2.30**	**0.36**	**0.00**	**CDC42,ITGA5,NCK1,RAC1,RHOB,RHOQ,RND3,RRAS2,SOS1**
Actin Nucleation by ARP-WASP Complex	2Gy 4h	0.36	0.28	-0.82	HRAS,KRAS,RAC3,RHOA,ROCK1,ROCK2,RRAS2
Activation of IRF by Cytosolic Pattern Recognition Receptors	0.05Gy 2h	0.66	0.04	NaN	IL6
Activation of IRF by Cytosolic Pattern Recognition Receptors	2Gy 2h	0.52	0.19	-0.45	IKBKB,IKBKE,IL6,MAPK8,RIPK1
Activation of IRF by Cytosolic Pattern Recognition Receptors	2Gy 4h	0.74	0.35	0.33	ATF2,IFIH1,IRF7,MAPK8,NFKB1,NFKBIA,PPIB,RIPK1,TRAF3
Acute Myeloid Leukemia Signaling	0.05Gy 2h	0.51	0.03	NaN	MYC
Acute Myeloid Leukemia Signaling	0.05Gy 4h	0.31	0.05	NaN	KIT,KITLG
Acute Myeloid Leukemia Signaling	2Gy 2h	0.76	0.21	-1.89	MAP2K5,MTOR,MYC,PIK3CB,PIK3CD,RRAS2,RUNX1,SOS1
**Acute Myeloid Leukemia Signaling**	**2Gy 4h**	**1.36**	**0.39**	**-0.58**	**AKT1,HRAS,KIT,KITLG,KRAS,LEF1,MAP2K1,NFKB1,PIK3CB,PIK3CD,RARA,RRAS2,RUNX1,TCF4,TCF7**
Acute Phase Response Signaling	0.05Gy 2h	0.61	0.02	NaN	IL6,SOCS3
Acute Phase Response Signaling	0.05Gy 4h	0.00	0.04	NaN	FOS,JAK2,NR3C1,SOCS6
**Acute Phase Response Signaling**	**2Gy 2h**	**1.91**	**0.23**	**0.00**	**CEBPB,ELK1,HMOX1,IKBKB,IKBKE,IL6,IL6R,MAPK8,MTOR,NOLC1,PDPK1,PIK3CB,PIK3CD,RIPK1,RRAS2,SERPINE1,SOCS1,SOCS2,SOCS3,SOCS5,SOCS6,SOS1,TNFRSF1B**

Ingenuity Canonical Pathways	Model	-log(p-value)	Ratio	z-score	Molecules
Acute Phase Response Signaling	2Gy 4h	1.07	0.31	-0.76	AKT1,CRABP2,ELK1,FOS,HMOX1,HMOX2,HRAS,IL1R1,IL6R,IL6ST,JAK2,KRAS,MAP2K1,MAP3K1,MAP3K7,MAPK8,NFKB1,NFKBIA,NGFR,NR3C1,PIK3CB,PIK3CD,RIPK1,RRAS2,SOCS2,SOCS3,SOCS5,SOCS6,SOCS7,TCF4,TNFRSF11B,TNFRSF1B
Adenine and Adenosine Salvage III	2Gy 4h	0.60	1.00	NaN	HPRT1
Adipogenesis pathway	0.05Gy 2h	0.87	0.03	NaN	DGKD,EGR2
Adipogenesis pathway	0.05Gy 4h	0.00	0.01	NaN	TGFB1
Adipogenesis pathway	2Gy 2h	0.00	0.13	NaN	CEBPB,CEBPD,DGKD,EGR2,FGF2,HDAC6,NFATC4,SMAD3,TXNIP
Adipogenesis pathway	2Gy 4h	1.13	0.33	NaN	AKT1,ARNTL,BMP2,BMP4,BMPR2,CCNH,CEBPD,CTNNB1,DDIT3,FGF2,FZD1,HIF1A,KDM1A,KLF5,NR1D2,RBBP7,SMAD3,SOX9,TCF7,TGFB1,TXNIP,WNT5A,ZNF423
Adrenomedullin signaling pathway	0.05Gy 4h	0.28	0.04	2.00	BCL2,FOS,MAX,RXRB
Adrenomedullin signaling pathway	2Gy 2h	0.43	0.16	-0.83	ADM,CASP3,CEBPB,ELK1,MAP2K5,MAPK7,MAPK8,MAX,PIK3CB,PIK3CD,RRAS2,RXRA,RXRB,SOS1
Adrenomedullin signaling pathway	2Gy 4h	0.00	0.25	-0.22	ADCY8,AKT1,BCL2,CASP3,ELK1,FOS,GSK3B,HIF1A,HRAS,ITPR2,KRAS,MAP2K1,MAPK8,MAX,NFKB1,NPR3,PIK3CB,PIK3CD,PRKAR2A,PTK2B,RRAS2,RXRB
Agranulocyte Adhesion and Diapedesis	0.05Gy 4h	0.00	0.04	NaN	ACTA2,GNAI2,HRH1
Agranulocyte Adhesion and Diapedesis	2Gy 2h	0.00	0.11	NaN	ACTA2,CCL2,CCL7,CXCL1,CXCL2,CXCL8,ITGA5,MMP15,SDC4
Agranulocyte Adhesion and Diapedesis	2Gy 4h	0.00	0.12	NaN	ACTA2,CD99,CXCL16,EZR,GNAI2,GNAI3,HRH1,ICAM1,IL1R1,MMP11
Agrin Interactions at Neuromuscular Junction	0.05Gy 4h	0.42	0.06	NaN	ACTA2,GABPA
Agrin Interactions at Neuromuscular Junction	2Gy 2h	0.58	0.19	0.82	ACTA2,CDC42,ITGA5,MAPK8,RAC1,RRAS2
Agrin Interactions at Neuromuscular Junction	2Gy 4h	0.43	0.29	-0.33	ACTA2,EGFR,GABPA,HRAS,KRAS,MAPK8,RAC3,RRAS2,SRC
Airway Pathology in Chronic Obstructive Pulmonary Disease	2Gy 2h	0.18	0.14	NaN	CXCL8
Aldosterone Signaling in Epithelial Cells	0.05Gy 4h	0.00	0.04	NaN	PRKCD,PRKCI
Aldosterone Signaling in Epithelial Cells	2Gy 2h	0.77	0.20	-2.65	DUSP1,HSPA4L,HSPA9,NR3C2,PDPK1,PIK3CB,PIK3CD,PRKCE,SGK1,SOS1
Aldosterone Signaling in Epithelial Cells	2Gy 4h	0.73	0.31	-0.30	DUSP1,HSP90AB1,HSPA4L,HSPA6,HSPA8,ITPR2,KRAS,MAP2K1,PIK3CB,PIK3CD,PRKCD,PRKCE,PRKCI,PRKD3,SLC12A2,SLC9A1
Allograft Rejection Signaling	2Gy 2h	0.00	0.06	NaN	FAS
Allograft Rejection Signaling	2Gy 4h	0.00	0.11	NaN	FAS,HLA-E
Altered T Cell and B Cell Signaling in Rheumatoid Arthritis	0.05Gy 2h	1.13	0.04	NaN	IL12A,IL6
Altered T Cell and B Cell Signaling in Rheumatoid Arthritis	0.05Gy 4h	0.00	0.02	NaN	TGFB1
Altered T Cell and B Cell Signaling in Rheumatoid Arthritis	2Gy 2h	0.00	0.06	NaN	FAS,IL6,RELB
Altered T Cell and B Cell Signaling in Rheumatoid Arthritis	2Gy 4h	0.00	0.13	NaN	CSF1,FAS,IL12A,NFKB1,TGFB1,TRAF3
AMPK Signaling	0.05Gy 2h	0.28	0.01	NaN	RAB3A
AMPK Signaling	0.05Gy 4h	0.38	0.05	NaN	CDKN1A,PPM1D,SMARCA4,STK11
**AMPK Signaling**	**2Gy 2h**	**1.69**	**0.23**	**-1.70**	**ACACB,ATF4,CDKN1A,CHRM3,HMGCR,IRS1,IRS2,LIPE,MTOR,PDPK1,PIK3CB,PIK3CD,PPARGC1A,PPM1D,PPP2CA,RAB3A,RAB6A,SLC2A1**
AMPK Signaling	2Gy 4h	0.67	0.30	-0.22	ADRB2,AKT1,ATF2,ATF4,CCNA2,CDKN1A,CPT1A,CPT2,FOXO3,HMGCR,LIPE,MAP3K7,PIK3CB,PIK3CD,PPM1D,PPM1G,PPP2CA,PRKAR2A,RAB27A,SLC2A1,SMARCA4,SRC,STK11
Amyloid Processing	2Gy 2h	0.00	0.06	NaN	PRKCE
Amyloid Processing	2Gy 4h	0.86	0.39	NaN	AKT1,CDK5R1,CSNK2B,GSK3B,PRKAR2A,PRKCE,PSEN2
Amyotrophic Lateral Sclerosis Signaling	0.05Gy 4h	0.00	0.02	NaN	BCL2

Ingenuity Canonical Pathways	Model	-log(p-value)	Ratio	z-score	Molecules
Amyotrophic Lateral Sclerosis Signaling	2Gy 2h	0.45	0.17	0.00	BCL2L1,BIRC2,CACNA1A,CASP3,PIK3CB,PIK3CD,RAC1
Amyotrophic Lateral Sclerosis Signaling	2Gy 4h	1.18	0.37	0.28	ALS2,BCL2,BCL2L1,BID,CACNA1C,CASP3,GDNF,GLUL,GPX1,PGF,PIK3CB,PIK3CD,PPP3CA,SOD1,XIAP
Androgen Signaling	0.05Gy 4h	0.92	0.09	NaN	GNAI2,GNG5,PRKCD,PRKCI
Androgen Signaling	2Gy 2h	0.00	0.11	NaN	CACNA1A,GNAO1,PRKCE,SMAD3,TGFB1I1
Androgen Signaling	2Gy 4h	0.88	0.33	-0.91	CACNA1C,CALR,CCNH,GNAI2,GNAI3,GNG5,NCOA2,NFKB1,PRKAR2A,PRKCD,PRKCE,PRKCI,PRKD3,SMAD3,SRC
Angiopoietin Signaling	2Gy 2h	1.06	0.25	0.45	IKBKB,IKBKE,NCK1,PIK3CB,PIK3CD,RRAS2,SOS1
Angiopoietin Signaling	2Gy 4h	0.60	0.32	-1.13	AKT1,CRK,HRAS,KRAS,NFKB1,NFKBIA,PIK3CB,PIK3CD,RRAS2
Antigen Presentation Pathway	0.05Gy 4h	1.05	0.17	NaN	PSMB9,TAP1
Antigen Presentation Pathway	2Gy 4h	0.45	0.33	NaN	CALR,HLA-E,PSMB9,TAP1
Antioxidant Action of Vitamin C	0.05Gy 4h	0.00	0.03	NaN	JAK2
Antioxidant Action of Vitamin C	2Gy 2h	0.97	0.23	0.38	HMOX1,IKBKB,IKBKE,MAPK8,NAPEPLD,PLD1,PNPLA8,SLC2A1
Antioxidant Action of Vitamin C	2Gy 4h	0.61	0.31	1.89	GLRX,HMOX1,JAK2,MAPK8,NFKB1,NFKBIA,PLD1,PNPLA8,SLC2A1,SLC2A5,TXN
Antiproliferative Role of Somatostatin Receptor 2	0.05Gy 2h	0.59	0.03	NaN	CDKN1B
Antiproliferative Role of Somatostatin Receptor 2	0.05Gy 4h	0.42	0.06	NaN	CDKN1A,GNG5
Antiproliferative Role of Somatostatin Receptor 2	2Gy 2h	0.58	0.19	-0.82	CDKN1A,CDKN1B,ELK1,PIK3CB,PIK3CD,RRAS2
Antiproliferative Role of Somatostatin Receptor 2	2Gy 4h	0.89	0.36	0.00	CDKN1A,ELK1,GNG5,HRAS,KRAS,MAP2K1,NPR3,PIK3CB,PIK3CD,RRAS2,SRC
Antiproliferative Role of TOB in T Cell Signaling	0.05Gy 2h	0.80	0.06	NaN	CDKN1B
Antiproliferative Role of TOB in T Cell Signaling	0.05Gy 4h	0.76	0.11	NaN	TGFB1,TGFBR1
Antiproliferative Role of TOB in T Cell Signaling	2Gy 2h	0.62	0.22	NaN	CCNE1,CDKN1B,SMAD3,TOB1
**Antiproliferative Role of TOB in T Cell Signaling**	**2Gy 4h**	**1.72**	**0.50**	**NaN**	**CCNA2,CCNE1,CCNE2,SMAD2,SMAD3,SMAD4,TGFB1,TGFBR1,TOB1**
Apelin Adipocyte Signaling Pathway	0.05Gy 4h	0.00	0.02	NaN	GNAI2
Apelin Adipocyte Signaling Pathway	2Gy 2h	0.00	0.10	1.00	LIPE,MAPK7,MAPK8,PPARGC1A
Apelin Adipocyte Signaling Pathway	2Gy 4h	0.45	0.29	0.58	ADCY8,GNAI2,GNAI3,GPX1,GSTP1,HIF1A,LIPE,MAPK8,NOX4,PRDX6,PRKAR2A,SOD1
Apelin Cardiac Fibroblast Signaling Pathway	0.05Gy 2h	0.88	0.07	NaN	IL6
Apelin Cardiac Fibroblast Signaling Pathway	0.05Gy 4h	0.32	0.07	NaN	TGFB1
Apelin Cardiac Fibroblast Signaling Pathway	2Gy 2h	0.00	0.13	NaN	IL6,SERPINE1
Apelin Cardiac Fibroblast Signaling Pathway	2Gy 4h	0.00	0.20	NaN	AKT1,CCN2,TGFB1
Apelin Cardiomyocyte Signaling Pathway	0.05Gy 4h	0.92	0.09	0.00	GNAI2,PRKCD,PRKCI,TGFB1
Apelin Cardiomyocyte Signaling Pathway	2Gy 2h	0.00	0.11	-0.45	MAPK7,MAPK8,PIK3CB,PIK3CD,PRKCE
Apelin Cardiomyocyte Signaling Pathway	2Gy 4h	0.67	0.31	-0.28	AKT1,ATP2A2,GNAI2,GNAI3,HIF1A,MAPK8,PIK3CB,PIK3CD,PRKCD,PRKCE,PRKCI,PRKD3,SLC9A1,TGFB1
Apelin Endothelial Signaling Pathway	0.05Gy 4h	0.56	0.06	1.00	FOS,GNAI2,PRKCD,PRKCI
Apelin Endothelial Signaling Pathway	2Gy 2h	0.58	0.18	-1.90	CCL2,GNAO1,KLF2,MAPK8,MEF2D,MTOR,PIK3CB,PIK3CD,PRKCE,RRAS2,SMAD3
Apelin Endothelial Signaling Pathway	2Gy 4h	1.07	0.33	-1.09	ADCY8,AKT1,FOS,GNAI2,GNAI3,HIF1A,HRAS,ICAM1,KRAS,MAP2K1,MAPK8,NFKB1,PIK3CB,PIK3CD,PRKCD,PRKCE,PRKCI,PRKD3,RRAS2,SMAD3,SP1
Apelin Liver Signaling Pathway	2Gy 2h	0.75	0.25	0.00	EDN1,FAS,IRS1,MAPK8
Apelin Liver Signaling Pathway	2Gy 4h	0.00	0.19	NaN	FAS,GSK3B,MAPK8
Apelin Muscle Signaling Pathway	2Gy 2h	0.33	0.18	NaN	PPARGC1A,TFAM
Apelin Muscle Signaling Pathway	2Gy 4h	0.00	0.18	NaN	AKT1,NRF1
Apelin Pancreas Signaling Pathway	2Gy 2h	0.26	0.15	NaN	MAPK8,PIK3CB,PIK3CD
Apelin Pancreas Signaling Pathway	2Gy 4h	0.42	0.30	0.82	DDIT3,MAPK8,NFKB1,PIK3CB,PIK3CD,PRKAR2A
Apoptosis Signaling	0.05Gy 4h	0.27	0.05	NaN	BCL2,LMNA
**Apoptosis Signaling**	**2Gy 2h**	**1.98**	**0.29**	**1.16**	**BCL2L1,BCL2L11,BIRC2,CASP3,CASP8,FAS,IKBKB,IKBKE,MAPK8,PRKCE,RRAS2,TNFRSF1B**

Ingenuity Canonical Pathways	Model	-log(p-value)	Ratio	z-score	Molecules
**Apoptosis Signaling**	**2Gy 4h**	**2.93**	**0.48**	**0.69**	**AIFM1,BCL2,BCL2L1,BID,CASP3,ENDOG,FAS,HRAS,KRAS,LMNA,MAP2K1,MAP4K4,MAPK8,NFKB1,NFKBIA,PRKCE,ROCK1,RRAS2,TNFRSF1B,XIAP**
April Mediated Signaling	0.05Gy 4h	0.66	0.10	NaN	FOS,NFATC1
April Mediated Signaling	2Gy 2h	1.20	0.29	-0.82	ELK1,IKBKB,IKBKE,MAPK8,NFATC1,NFATC4
April Mediated Signaling	2Gy 4h	1.25	0.43	-1.67	ELK1,FOS,MAP3K1,MAPK8,NFAT5,NFATC1,NFKB1,NFKBIA,TRAF3
Arginine Biosynthesis IV	2Gy 2h	0.34	0.25	NaN	OAT
Arginine Biosynthesis IV	2Gy 4h	0.58	0.50	NaN	GLUD1,OAT
Arginine Degradation I (Arginase Pathway)	2Gy 2h	0.44	0.33	NaN	OAT
Arginine Degradation I (Arginase Pathway)	2Gy 4h	0.24	0.33	NaN	OAT
Arginine Degradation VI (Arginase 2 Pathway)	2Gy 2h	0.34	0.25	NaN	OAT
Arginine Degradation VI (Arginase 2 Pathway)	2Gy 4h	0.58	0.50	NaN	OAT,PYCR1
**Aryl Hydrocarbon Receptor Signaling**	**0.05Gy 2h**	**1.40**	**0.04**	**NaN**	**CDKN1B,IL6,MYC**
Aryl Hydrocarbon Receptor Signaling	0.05Gy 4h	1.26	0.09	-1.13	CDKN1A,FOS,MDM2,RARG,RXRB,SMARCA4,TGFB1
Aryl Hydrocarbon Receptor Signaling	2Gy 2h	0.59	0.17	-0.54	CCNE1,CDKN1A,CDKN1B,CHEK1,FAS,IL6,MAPK8,MDM2,MYC,NCOR2,PTGES3,RARG,RXRA,RXRB
Aryl Hydrocarbon Receptor Signaling	2Gy 4h	1.19	0.33	-1.63	ALDH18A1,ATM,CCNA2,CCNE1,CCNE2,CDKN1A,CDKN2A,CTSD,CYP1B1,E2F1,FAS,FOS,GSTP1,HSP90AB1,MAPK8,MDM2,NCOA2,NFIB,NFKB1,PTGES3,RARA,RBL2,RXRB,SMARCA4,SP1,SRC,TGFB1
Ascorbate Recycling (Cytosolic)	2Gy 4h	0.60	1.00	NaN	GLRX
Assembly of RNA Polymerase II Complex	2Gy 4h	0.60	1.00	NaN	CCNH
**Assembly of RNA Polymerase III Complex**	**0.05Gy 4h**	**1.37**	**1.00**	**NaN**	**BRF1**
Assembly of RNA Polymerase III Complex	2Gy 4h	0.60	1.00	NaN	BRF1
Atherosclerosis Signaling	0.05Gy 2h	0.31	0.01	NaN	IL6
Atherosclerosis Signaling	0.05Gy 4h	0.00	0.01	NaN	TGFB1
Atherosclerosis Signaling	2Gy 2h	0.00	0.11	NaN	APOE,CCL2,CXCL8,F3,IL6,PDGFA,PNPLA8,TNFRSF12A
Atherosclerosis Signaling	2Gy 4h	0.00	0.09	NaN	CSF1,ICAM1,NFKB1,PNPLA8,TGFB1,TNFRSF12A
**ATM Signaling**	**0.05Gy 4h**	**2.06**	**0.16**	**-0.45**	**CDC25C,CDKN1A,MDM2,PPM1D,TRIM28**
ATM Signaling	2Gy 2h	0.87	0.23	-0.38	ATF4,CDKN1A,CHEK1,MAPK8,MDM2,PPM1D,PPP2CA
**ATM Signaling**	**2Gy 4h**	**1.95**	**0.45**	**-0.58**	**ABL1,ATF2,ATF4,ATM,BID,CCNB1,CDKN1A,GADD45A,MAPK8,MDM2,NFKBIA,PPM1D,PPP2CA,TRIM28**
Autoimmune Thyroid Disease Signaling	2Gy 2h	0.00	0.06	NaN	FAS
Autoimmune Thyroid Disease Signaling	2Gy 4h	0.00	0.12	NaN	FAS,HLA-E
Autophagy	0.05Gy 4h	0.34	0.07	NaN	BCL2
Autophagy	2Gy 2h	0.50	0.21	NaN	ATG9A,MAP1LC3B,MTOR
Autophagy	2Gy 4h	0.59	0.36	NaN	ATG9A,BCL2,CTSD,MAP1LC3A,MAP1LC3B
Axonal Guidance Signaling	0.05Gy 4h	0.26	0.04	NaN	ADAM15,EFNA4,GNAI2,GNG5,NFATC1,PRKCD,PRKCI,RHOA
Axonal Guidance Signaling	2Gy 2h	0.00	0.13	NaN	BDNF,CDC42,EFNB1,EPHA2,EPHB3,EPHB4,GLI3,GNAO1,ITGA5,LIMK2,MMP15,NCK1,NFATC1,NFATC4,PDGFA,PIK3CB,PIK3CD,PPP3CC,PPP3R1,PRKCE,RAC1,RRAS2,SOS1,TUBB2A,TUBB4B
Axonal Guidance Signaling	2Gy 4h	0.48	0.27	NaN	ABL1,ADAM15,ADAM17,AKT1,BCAR1,BMP1,BMP2,BMP4,CFL1,CRK,EFNB1,EPHA2,EPHB4,FZD1,GLI3,GNAI2,GNAI3,GNG5,GSK3B,HRAS,KRAS,LIMK2,MAP2K1,MMP11,NFAT5,NFATC1,NGFR,NRP1,PFN1,PGF,PIK3CB,PIK3CD,PPP3CA,PPP3CB,PPP3CC,PPP3R1,PRKAR2A,PRKCD,PRKCE,PRKCI,PRKD3,PTCH1,RAC3,RHOA,ROCK1,ROCK2,RRAS2,RTN4,TUBB2A,WNT5A
B Cell Activating Factor Signaling	0.05Gy 4h	0.66	0.10	NaN	FOS,NFATC1
B Cell Activating Factor Signaling	2Gy 2h	1.20	0.29	-0.82	ELK1,IKBKB,IKBKE,MAPK8,NFATC1,NFATC4

Ingenuity Canonical Pathways	Model	-log(p-value)	Ratio	z-score	Molecules
B Cell Activating Factor Signaling	2Gy 4h	1.25	0.43	-1.89	ELK1,FOS,MAP3K1,MAPK8,NFAT5,NFATC1,NFKB1,NFKBIA,TRAF3
B Cell Receptor Signaling	0.05Gy 2h	0.29	0.01	NaN	EGR1
B Cell Receptor Signaling	0.05Gy 4h	0.00	0.04	NaN	EGR1,NFATC1,VAV2
**B Cell Receptor Signaling**	**2Gy 2h**	**2.97**	**0.28**	**-1.53**	**ATF4,BCL2L1,BCL6,CAMK2G,CDC42,EGR1,ELK1,IKBKB,IKBKE,MAPK8,MTOR,NFATC1,NFATC4,PDPK1,PIK3CB,PIK3CD,PPP3CC,PPP3R1,RAC1,RRAS2,SOS1**
**B Cell Receptor Signaling**	**2Gy 4h**	**2.90**	**0.41**	**-0.54**	**ABL1,AKT1,ATF2,ATF4,BCL2L1,BCL6,CFL1,EGR1,ELK1,ETS1,GSK3B,HRAS,KRAS,MAP2K1,MAP3K1,MAP3K7,MAPK8,NFAT5,NFATC1,NFKB1,NFKBIA,PIK3CB,PIK3CD,PPP3CA,PPP3CB,PPP3CC,PPP3R1,PTEN,PTK2B,RRAS2,VAV2**
BAG2 Signaling Pathway	0.05Gy 2h	0.88	0.07	NaN	MYC
BAG2 Signaling Pathway	0.05Gy 4h	0.89	0.13	NaN	CDKN1A,MDM2
**BAG2 Signaling Pathway**	**2Gy 2h**	**1.32**	**0.33**	**1.34**	**CASP3,CDKN1A,HSPA9,MDM2,MYC**
**BAG2 Signaling Pathway**	**2Gy 4h**	**1.76**	**0.53**	**0.00**	**CASP3,CDKN1A,HSPA6,HSPA8,MDM2,NFKB1,SP1,STUB1**
Basal Cell Carcinoma Signaling	2Gy 2h	0.00	0.03	NaN	GLI3
**Basal Cell Carcinoma Signaling**	**2Gy 4h**	**1.32**	**0.39**	**-0.71**	**AXIN1,BMP1,BMP2,BMP4,CTNNB1,FZD1,GLI3,GSK3B,LEF1,PTCH1,TCF4,TCF7,WNT5A**
BER pathway	2Gy 2h	0.22	0.17	NaN	PCNA
**BER pathway**	**2Gy 4h**	**1.42**	**0.67**	**NaN**	**APEX1,LIG1,PCNA,POLB**
Bile Acid Biosynthesis, Neutral Pathway	2Gy 2h	0.00	0.13	NaN	SCP2
Biotin-carboxyl Carrier Protein Assembly	2Gy 2h	0.44	0.33	NaN	ACACB
Biotin-carboxyl Carrier Protein Assembly	2Gy 4h	0.24	0.33	NaN	HLCS
Bladder Cancer Signaling	0.05Gy 2h	0.41	0.02	NaN	MYC
Bladder Cancer Signaling	0.05Gy 4h	0.42	0.06	NaN	CDKN1A,FGF5,MDM2
Bladder Cancer Signaling	2Gy 2h	0.73	0.19	-0.45	CDKN1A,CXCL8,FGF2,FGF7,FGF9,MDM2,MMP15,MYC,RASSF1,RRAS2
Bladder Cancer Signaling	2Gy 4h	0.68	0.31	0.00	ABL1,CDKN1A,CDKN2A,E2F1,EGFR,FGF2,FGF5,HRAS,KRAS,MAP2K1,MDM2,MMP11,PA2G4,PGF,RASSF1,RRAS2
BMP signaling pathway	0.05Gy 2h	0.48	0.02	NaN	FST
BMP signaling pathway	2Gy 2h	0.26	0.14	-0.82	FST,MAPK8,RRAS2,RUNX2,SMAD7,SOS1
**BMP signaling pathway**	**2Gy 4h**	**2.93**	**0.48**	**-0.94**	**ATF2,BMP1,BMP2,BMP4,BMPR2,FST,GREM1,HRAS,KRAS,MAP2K1,MAP3K7,MAPK8,NFKB1,PRKAR2A,RRAS2,SMAD4,SMAD6,SMAD7,XIAP,ZNF423**
Branched-chain Î±-keto acid Dehydrogenase Complex	2Gy 4h	0.36	0.50	NaN	DLD
Breast Cancer Regulation by Stathmin1	0.05Gy 2h	0.26	0.01	NaN	CDKN1B
**Breast Cancer Regulation by Stathmin1**	**0.05Gy 4h**	**1.63**	**0.10**	**NaN**	**CDKN1A,E2F4,E2F5,GNAI2,GNG5,PRKCD,PRKCI,RHOA**
Breast Cancer Regulation by Stathmin1	2Gy 2h	0.71	0.18	NaN	CAMK2G,CCNE1,CDC42,CDKN1A,CDKN1B,LIMK2,PIK3CB,PIK3CD,PPP2CA,PRKCE,RAC1,RRAS2,SOS1,TUBB2A,TUBB4B
**Breast Cancer Regulation by Stathmin1**	**2Gy 4h**	**1.75**	**0.36**	**NaN**	**ADCY8,ARHGEF3,CAMK1,CCNE1,CCNE2,CDKN1A,E2F1,E2F5,GNAI2,GNAI3,GNG5,HRAS,ITPR2,KRAS,LIMK2,MAP2K1,PIK3CB,PIK3CD,PPP1CA,PPP2CA,PRKAR2A,PRKCD,PRKCE,PRKCI,PRKD3,RHOA,ROCK1,ROCK2,RRAS2,TUBB2A**
Bupropion Degradation	2Gy 4h	0.00	0.07	NaN	CYP1B1
Calcium Signaling	0.05Gy 4h	0.00	0.03	NaN	ACTA2,NFATC1
Calcium Signaling	2Gy 2h	0.53	0.17	-2.12	ACTA2,ATF4,CACNA1A,CAMK2G,HDAC6,MEF2D,NFATC1,NFATC4,PPP3CC,PPP3R1
Calcium Signaling	2Gy 4h	1.17	0.35	-2.14	ACTA2,ATF2,ATF4,ATP2A2,ATP2B1,ATP2C1,CACNA1C,CALR,CAMK1,ITPR2,NFAT5,NFATC1,PPP3CA,PPP3CB,PPP3CC,PPP3R1,PRKAR2A,TP63,TRPC1,TRPC6
Calcium Transport I	2Gy 4h	0.98	0.60	NaN	ATP2A2,ATP2B1,ATP2C1
Calcium-induced T Lymphocyte Apoptosis	0.05Gy 4h	0.37	0.06	NaN	PRKCD,PRKCI
Calcium-induced T Lymphocyte Apoptosis	2Gy 2h	0.27	0.15	-1.34	MEF2D,NR4A1,PPP3CC,PPP3R1,PRKCE
Calcium-induced T Lymphocyte Apoptosis	2Gy 4h	0.47	0.29	-1.90	ATP2A2,ITPR2,PPP3CA,PPP3CB,PPP3CC,PPP3R1,PRKCD,PRKCE,PRKCI,PRKD3

Ingenuity Canonical Pathways	Model	-log(p-value)	Ratio	z-score	Molecules
cAMP-mediated signaling	0.05Gy 4h	0.00	0.01	NaN	GNAI2
cAMP-mediated signaling	2Gy 2h	0.00	0.14	-0.54	ATF4,CAMK2G,CHRM3,DUSP1,DUSP6,GNAO1,NAPEPLD,PDE4D,PPP3CC,PPP3R1,PTGER4,RGS12,RGS4,S1PR3
cAMP-mediated signaling	2Gy 4h	0.00	0.24	-0.60	ADCY8,ADORA2B,ADRB2,APEX1,ATF2,ATF4,CAMK1,CREM,DUSP1,DUSP6,GNAI2,GNAI3,LPAR1,MAP2K1,NPR3,PKIG,PPP3CA,PPP3CB,PPP3CC,PPP3R1,PRKAR2A,PTGER2,PTGER3,RGS4,SRC
Cancer Drug Resistance By Drug Efflux	0.05Gy 2h	0.56	0.03	NaN	PTGS2
Cancer Drug Resistance By Drug Efflux	0.05Gy 4h	0.00	0.03	NaN	MDM2
Cancer Drug Resistance By Drug Efflux	2Gy 2h	0.27	0.15	NaN	MDM2,PIK3CB,PIK3CD,PTGS2,RRAS2
Cancer Drug Resistance By Drug Efflux	2Gy 4h	1.22	0.38	NaN	AKT1,FOXO3,HRAS,KRAS,MAP2K1,MDM2,NFKB1,PIK3CB,PIK3CD,PTEN,PTGS2,RRAS2,YBX1
Cardiac Hypertrophy Signaling	0.05Gy 2h	0.22	0.01	NaN	IL6
Cardiac Hypertrophy Signaling	0.05Gy 4h	0.64	0.06	0.82	GNAI2,GNG5,RHOA,RHOG,TGFB1,TGFBR1
**Cardiac Hypertrophy Signaling**	**2Gy 2h**	**1.55**	**0.21**	**-1.41**	**CACNA1A,CDC42,ELK1,GNAO1,IL6,IL6R,IRS1,MAPK8,MEF2D,MTOR,NFATC4,PIK3CB,PIK3CD,PPP3CC,PPP3R1,RAC1,RHOB,RHOQ,RND3,RRAS2,SOS1**
Cardiac Hypertrophy Signaling	2Gy 4h	1.28	0.33	-1.62	ADCY8,ADRB2,AKT1,ATF2,CACNA1C,ELK1,GNAI2,GNAI3,GNG5,GSK3B,HRAS,IL6R,KRAS,MAP2K1,MAP3K1,MAP3K7,MAPK8,PIK3CB,PIK3CD,PPP3CA,PPP3CB,PPP3CC,PPP3R1,PRKAR2A,RAC3,RHOA,ROCK1,ROCK2,RRAS2,SRF,TGFB1,TGFBR1
Cardiac Hypertrophy Signaling (Enhanced)	0.05Gy 2h	1.12	0.02	-1.34	IL11,IL12A,IL6,MYC,PTGS2
Cardiac Hypertrophy Signaling (Enhanced)	0.05Gy 4h	0.00	0.04	0.63	FGF5,GNAI2,GNG5,JAK2,NFATC1,PRKCD,PRKCI,RHOA,TGFB1,TGFBR1
Cardiac Hypertrophy Signaling (Enhanced)	2Gy 2h	0.29	0.14	-2.54	ACVR2A,CACNA1A,CAMK2G,CXCL8,EDN1,ELK1,FGF2,FGF7,FGF9,HDAC6,IKBKB,IKBKE,IL11,IL4R,IL6,IL6R,ITGA5,MAP2K5,MAPK7,MAPK8,MEF2D,MTOR,MYC,NAPEPLD,NFATC1,NFATC4,PDE4D,PIK3CB,PIK3CD,PPP3CC,PPP3R1,PRKCE,PTGS2,RRAS2,TNFRSF1B
Cardiac Hypertrophy Signaling (Enhanced)	2Gy 4h	0.92	0.28	-2.81	ACVR1,ACVR1B,ACVR2A,ADCY8,ADRB2,AKT1,APEX1,ATF2,ATF6,ATP2A2,BMPR2,CACNA1C,CLCF1,CNTF,CTNNB1,DLG1,ELK1,FGF2,FGF5,FZD1,GHR,GNAI2,GNAI3,GNG5,GSK3B,HRAS,IL12A,IL17RA,IL18R1,IL1R1,IL22RA1,IL4R,IL6R,IL6ST,ITPR2,JAK2,KRAS,LIF,MAP2K1,MAP3K1,MAP3K7,MAPK8,NFAT5,NFATC1,NFKB1,NGFR,PIK3CB,PIK3CD,PPP3CA,PPP3CB,PPP3CC,PPP3R1,PRKAR2A,PRKCD,PRKCE,PRKCI,PRKD3,PTEN,PTGS2,RHOA,ROCK1,ROCK2,RRAS2,SRF,TGFB1,TGFBR1,TNFRSF11B,TNFRSF1B,TNFSF10,WNT5A
Cardiac Î²-adrenergic Signaling	0.05Gy 4h	0.00	0.03	NaN	GNG5
Cardiac Î²-adrenergic Signaling	2Gy 2h	0.00	0.11	-2.00	CACNA1A,NAPEPLD,PDE4D,PPP2CA
Cardiac Î²-adrenergic Signaling	2Gy 4h	0.00	0.25	-0.71	ADCY8,APEX1,ATP2A2,CACNA1C,GNG5,PKIG,PPP1CA,PPP2CA,PRKAR2A
**Cardiomyocyte Differentiation via BMP Receptors**	**2Gy 4h**	**1.85**	**0.58**	**-1.34**	**ATF2,BMP2,BMP4,BMPR2,MAP3K7,SMAD4,SMAD6**
Caveolar-mediated Endocytosis Signaling	0.05Gy 4h	0.00	0.03	NaN	ACTA2
Caveolar-mediated Endocytosis Signaling	2Gy 2h	0.00	0.09	NaN	ACTA2,CD55,ITGA5
Caveolar-mediated Endocytosis Signaling	2Gy 4h	0.00	0.18	NaN	ABL1,ACTA2,EGFR,HLA-E,ITGB8,SRC
CCR3 Signaling in Eosinophils	0.05Gy 4h	1.18	0.10	NaN	GNAI2,GNG5,PRKCD,PRKCI,RHOA
CCR3 Signaling in Eosinophils	2Gy 2h	0.00	0.12	-0.45	LIMK2,PIK3CB,PIK3CD,PRKCE,RAC1,RRAS2
**CCR3 Signaling in Eosinophils**	**2Gy 4h**	**1.37**	**0.37**	**0.00**	**CFL1,GNAI2,GNAI3,GNG5,HRAS,ITPR2,KRAS,LIMK2,MAP2K1,PIK3CB,PIK3CD,PRKCD,PRKCE,PRKCI,PRKD3,RHOA,ROCK1,ROCK2,RRAS2**
**CCR5 Signaling in Macrophages**	**0.05Gy 4h**	**2.00**	**0.16**	**1.00**	**FOS,GNAI2,GNG5,PRKCD,PRKCI**
CCR5 Signaling in Macrophages	2Gy 2h	0.00	0.13	NaN	CACNA1A,FAS,MAPK8,PRKCE
CCR5 Signaling in Macrophages	2Gy 4h	1.10	0.38	-0.33	CACNA1C,FAS,FOS,GNAI2,GNAI3,GNG5,MAPK8,PRKCD,PRKCE,PRKCI,PRKD3,PTK2B
CD27 Signaling in Lymphocytes	0.05Gy 4h	0.00	0.04	NaN	FOS
CD27 Signaling in Lymphocytes	2Gy 2h	1.29	0.28	-0.38	BCL2L1,CASP3,CASP8,IKBKB,IKBKE,MAP2K5,MAPK8
**CD27 Signaling in Lymphocytes**	**2Gy 4h**	**1.53**	**0.44**	**-0.63**	**BCL2L1,BID,CASP3,FOS,MAP2K1,MAP3K1,MAP3K7,MAPK8,NFKB1,NFKBIA,SIVA1**

Ingenuity Canonical Pathways	Model	-log(p-value)	Ratio	z-score	Molecules
CD28 Signaling in T Helper Cells	0.05Gy 4h	0.00	0.04	NaN	FOS,NFATC1
**CD28 Signaling in T Helper Cells**	**2Gy 2h**	**1.34**	**0.24**	**-1.16**	**CDC42,IKBKB,IKBKE,MAPK8,NFATC1,NFATC4,PDPK1,PIK3CB,PIK3CD,PPP3CC,PPP3R1,RAC1**
CD28 Signaling in T Helper Cells	2Gy 4h	0.73	0.31	-1.29	AKT1,FOS,ITPR2,MAP2K1,MAP3K1,MAPK8,NFAT5,NFATC1,NFKB1,NFKBIA,PIK3CB,PIK3CD,PPP3CA,PPP3CB,PPP3CC,PPP3R1
CD40 Signaling	0.05Gy 2h	0.63	0.04	NaN	PTGS2
CD40 Signaling	0.05Gy 4h	0.00	0.04	NaN	FOS
**CD40 Signaling**	**2Gy 2h**	**1.47**	**0.29**	**-1.13**	**IKBKB,IKBKE,MAP2K5,MAPK8,PIK3CB,PIK3CD,PTGS2,TNFAIP3**
CD40 Signaling	2Gy 4h	1.17	0.39	-1.27	FOS,ICAM1,MAP2K1,MAP3K7,MAPK8,NFKB1,NFKBIA,PIK3CB,PIK3CD,PTGS2,TRAF3
Cdc42 Signaling	0.05Gy 4h	1.28	0.12	NaN	CDC42BPA,FOS,PRKCI,VAV2
Cdc42 Signaling	2Gy 2h	0.00	0.12	0.00	CDC42,ITGA5,LIMK2,MAPK8
Cdc42 Signaling	2Gy 4h	0.68	0.32	0.33	ATF2,CDC42BPA,CFL1,FOS,GSK3B,HLA-E,LIMK2,MAPK8,PRKCI,SRC,VAV2
CDK5 Signaling	0.05Gy 2h	0.49	0.02	NaN	EGR1
CDK5 Signaling	0.05Gy 4h	0.00	0.02	NaN	EGR1
CDK5 Signaling	2Gy 2h	0.68	0.20	-1.41	BDNF,CABLES1,CACNA1A,EGR1,MAPK7,MAPK8,PPP2CA,RRAS2
CDK5 Signaling	2Gy 4h	0.91	0.34	-0.54	ABL1,ADCY8,CABLES1,CDK5R1,EGR1,HRAS,KRAS,MAP2K1,MAPK8,NGFR,PPP1CA,PPP2CA,PRKAR2A,RRAS2
Cell Cycle Control of Chromosomal Replication	0.05Gy 2h	0.85	0.06	NaN	ORC2
Cell Cycle Control of Chromosomal Replication	2Gy 2h	0.75	0.25	1.00	CDC6,ORC2,ORC3,PCNA
Cell Cycle Control of Chromosomal Replication	2Gy 4h	1.10	0.44	-0.38	CDC6,CDK14,CDT1,LIG1,ORC1,ORC3,PCNA
**Cell Cycle Regulation by BTG Family Proteins**	**0.05Gy 4h**	**1.72**	**0.21**	**NaN**	**BTG2,E2F4,E2F5**
Cell Cycle Regulation by BTG Family Proteins	2Gy 2h	0.50	0.21	NaN	BTG2,CCNE1,PPP2CA
**Cell Cycle Regulation by BTG Family Proteins**	**2Gy 4h**	**1.42**	**0.50**	**-1.34**	**BTG2,CCNE1,CCNE2,CNOT7,E2F1,E2F5,PPP2CA**
Cell Cycle: G1/S Checkpoint Regulation	0.05Gy 2h	1.20	0.05	NaN	CDKN1B,MYC
**Cell Cycle: G1/S Checkpoint Regulation**	**0.05Gy 4h**	**2.68**	**0.16**	**-0.82**	**CDKN1A,E2F4,E2F5,GNL3,MAX,MDM2,TGFB1**
Cell Cycle: G1/S Checkpoint Regulation	2Gy 2h	0.81	0.21	1.00	CCNE1,CDKN1A,CDKN1B,GNL3,HDAC6,MAX,MDM2,MYC,SMAD3
**Cell Cycle: G1/S Checkpoint Regulation**	**2Gy 4h**	**2.62**	**0.46**	**-0.47**	**ABL1,ATM,BMI1,CCNE1,CCNE2,CDKN1A,CDKN2A,CDKN2B,CDKN2D,E2F1,E2F5,GNL3,GSK3B,MAX,MDM2,PA2G4,RBL2,SMAD3,SMAD4,TGFB1**
**Cell Cycle: G2/M DNA Damage Checkpoint Regulation**	**0.05Gy 4h**	**1.91**	**0.18**	**-1.00**	**CDC25C,CDKN1A,MDM2,PPM1D**
Cell Cycle: G2/M DNA Damage Checkpoint Regulation	2Gy 2h	1.11	0.27	0.00	AURKA,CDC25B,CDKN1A,CHEK1,MDM2,PPM1D
**Cell Cycle: G2/M DNA Damage Checkpoint Regulation**	**2Gy 4h**	**1.53**	**0.46**	**0.63**	**ABL1,ATM,AURKA,CCNB1,CDC25B,CDKN1A,CDKN2A,GADD45A,MDM2,PPM1D**
Cellular Effects of Sildenafil (Viagra)	0.05Gy 4h	0.00	0.02	NaN	ACTA2
Cellular Effects of Sildenafil (Viagra)	2Gy 2h	0.00	0.07	NaN	ACTA2,CACNA1A,PDE4D
Cellular Effects of Sildenafil (Viagra)	2Gy 4h	0.00	0.11	NaN	ACTA2,ADCY8,CACNA1C,ITPR2,PRKAR2A
Ceramide Signaling	0.05Gy 4h	0.41	0.06	NaN	BCL2,FOS
Ceramide Signaling	2Gy 2h	0.82	0.22	2.65	MAPK8,PIK3CB,PIK3CD,PPP2CA,RRAS2,S1PR3,TNFRSF1B
**Ceramide Signaling**	**2Gy 4h**	**3.24**	**0.53**	**-0.73**	**AKT1,BCL2,CTSD,FOS,HRAS,KRAS,MAP2K1,MAP3K1,MAPK8,NFKB1,NGFR,PIK3CB,PIK3CD,PPP2CA,RRAS2,TNFRSF11B,TNFRSF1B**
Chemokine Signaling	0.05Gy 4h	0.57	0.07	NaN	FOS,GNAI2,RHOA
Chemokine Signaling	2Gy 2h	0.00	0.14	0.00	CAMK2G,CCL2,CCL7,LIMK2,MAPK8,RRAS2
Chemokine Signaling	2Gy 4h	1.02	0.35	0.26	CAMK1,CFL1,FOS,GNAI2,GNAI3,HRAS,KRAS,LIMK2,MAP2K1,MAPK8,PTK2B,RHOA,ROCK2,RRAS2,SRC
Cholecystokinin/Gastrin-mediated Signaling	0.05Gy 2h	0.35	0.02	NaN	PTGS2

Ingenuity Canonical Pathways	Model	-log(p-value)	Ratio	z-score	Molecules
Cholecystokinin/Gastrin-mediated Signaling	0.05Gy 4h	0.92	0.08	1.34	FOS,PRKCD,PRKCI,RHOA,RHOG
**Cholecystokinin/Gastrin-mediated Signaling**	**2Gy 2h**	**1.35**	**0.23**	**-0.58**	**CDC42,ELK1,MAP2K5,MAPK7,MAPK8,MEF2D,PRKCE,PTGS2,RAC1,RHOB,RHOQ,RND3,RRAS2,SOS1**
**Cholecystokinin/Gastrin-mediated Signaling**	**2Gy 4h**	**1.95**	**0.39**	**-1.04**	**ATF2,BCAR1,CREM,EGFR,ELK1,FOS,HRAS,ITPR2,KRAS,MAP2K1,MAPK8,PRKCD,PRKCE,PRKCI,PRKD3,PTGS2,PTK2B,RAC3,RHOA,ROCK1,ROCK2,RRAS2,SRC,SRF**
Cholesterol Biosynthesis I	2Gy 2h	0.27	0.20	NaN	SC5D
Cholesterol Biosynthesis II (via 24,25-dihydrolanosterol)	2Gy 2h	0.27	0.20	NaN	SC5D
Cholesterol Biosynthesis III (via Desmosterol)	2Gy 2h	0.27	0.20	NaN	SC5D
**Choline Biosynthesis III**	**2Gy 2h**	**1.77**	**0.50**	**0.00**	**CHPT1,HMOX1,NAPEPLD,PLD1**
Choline Biosynthesis III	2Gy 4h	0.00	0.25	NaN	HMOX1,PLD1
Chondroitin Sulfate Biosynthesis	0.05Gy 4h	0.37	0.08	NaN	HS6ST1
Chondroitin Sulfate Biosynthesis	2Gy 2h	0.56	0.23	NaN	CHST11,CHST2,HS6ST1
Chondroitin Sulfate Biosynthesis	2Gy 4h	0.00	0.08	NaN	HS6ST1
Chondroitin Sulfate Biosynthesis (Late Stages)	0.05Gy 4h	0.39	0.08	NaN	HS6ST1
Chondroitin Sulfate Biosynthesis (Late Stages)	2Gy 2h	0.63	0.25	NaN	CHST11,CHST2,HS6ST1
Chondroitin Sulfate Biosynthesis (Late Stages)	2Gy 4h	0.00	0.08	NaN	HS6ST1
Chondroitin Sulfate Degradation (Metazoa)	2Gy 4h	0.00	0.25	NaN	CEMIP
Chronic Myeloid Leukemia Signaling	0.05Gy 2h	1.05	0.04	NaN	CDKN1B,MYC
**Chronic Myeloid Leukemia Signaling**	**0.05Gy 4h**	**1.61**	**0.11**	**NaN**	**CDKN1A,E2F4,E2F5,MDM2,TGFB1,TGFBR1**
**Chronic Myeloid Leukemia Signaling**	**2Gy 2h**	**1.49**	**0.24**	**NaN**	**BCL2L1,CDKN1A,CDKN1B,HDAC6,IKBKB,IKBKE,MDM2,MYC,PIK3CB,PIK3CD,RRAS2,SMAD3,SOS1**
**Chronic Myeloid Leukemia Signaling**	**2Gy 4h**	**2.12**	**0.41**	**NaN**	**ABL1,AKT1,BCL2L1,CDKN1A,CDKN2A,CRK,E2F1,E2F5,HRAS,KRAS,MAP2K1,MDM2,NFKB1,PA2G4,PIK3CB,PIK3CD,RBL2,RRAS2,SMAD3,SMAD4,TGFB1,TGFBR1**
Circadian Rhythm Signaling	0.05Gy 4h	0.42	0.09	NaN	CRY1
Circadian Rhythm Signaling	2Gy 2h	0.71	0.27	NaN	ATF4,CRY1,PER1
**Circadian Rhythm Signaling**	**2Gy 4h**	**1.46**	**0.55**	**NaN**	**ARNTL,ATF2,ATF4,CRY1,NR1D1,PER3**
Citrulline Biosynthesis	2Gy 2h	0.27	0.20	NaN	OAT
Citrulline Biosynthesis	2Gy 4h	0.43	0.40	NaN	ALDH18A1,OAT
Clathrin-mediated Endocytosis Signaling	0.05Gy 4h	0.59	0.06	NaN	ACTA2,ARF6,FGF5,LDLRAP1,MDM2
**Clathrin-mediated Endocytosis Signaling**	**2Gy 2h**	**1.54**	**0.22**	**NaN**	**ACTA2,AP3B1,AP3S1,APOE,ARF6,CDC42,FGF2,FGF7,FGF9,ITGA5,LDLRAP1,MDM2,PDGFA,PIK3CB,PIK3CD,PPP3CC,PPP3R1,RAC1**
Clathrin-mediated Endocytosis Signaling	2Gy 4h	0.43	0.27	NaN	ACTA2,AP3M1,ARF6,CSNK2B,FGF2,FGF5,HSPA8,ITGB8,LDLR,LDLRAP1,MDM2,PGF,PIK3CB,PIK3CD,PPP3CA,PPP3CB,PPP3CC,PPP3R1,RAB4A,SH3BP4,SRC,UBC
CNTF Signaling	0.05Gy 4h	0.20	0.04	NaN	JAK2
CNTF Signaling	2Gy 2h	1.03	0.26	-1.63	MTOR,PIK3CB,PIK3CD,RRAS2,SOS1,TYK2
**CNTF Signaling**	**2Gy 4h**	**1.83**	**0.48**	**-0.91**	**AKT1,CNTF,HRAS,IL6ST,JAK2,KRAS,LIFR,MAP2K1,PIK3CB,PIK3CD,RRAS2**
Coagulation System	2Gy 2h	0.43	0.18	-1.00	BDKRB1,F3,PLAUR,SERPINE1
Coagulation System	2Gy 4h	0.00	0.09	NaN	SERPINA5,THBD
**Colanic Acid Building Blocks Biosynthesis**	**0.05Gy 4h**	**2.29**	**0.67**	**NaN**	**GALK1,GPI**
Colanic Acid Building Blocks Biosynthesis	2Gy 2h	0.44	0.33	NaN	UGDH
Colanic Acid Building Blocks Biosynthesis	2Gy 4h	0.80	0.67	NaN	GALK1,GPI
Colorectal Cancer Metastasis Signaling	0.05Gy 2h	0.82	0.02	NaN	IL6,MYC,PTGS2
Colorectal Cancer Metastasis Signaling	0.05Gy 4h	0.58	0.06	0.82	E2F4,FOS,GNG5,JAK2,RHOA,RHOG,TGFB1,TGFBR1
Colorectal Cancer Metastasis Signaling	2Gy 2h	0.33	0.15	-0.94	BCL2L1,CASP3,CDC42,IL6,IL6R,MAPK8,MMP15,MSH6,MYC,PIK3CB,PIK3CD,PTGER4,PTGS2,RAC1,RHOB,RHOQ,RND3,RRAS2,SMAD3,SOS1,TYK2

Ingenuity Canonical Pathways	Model	-log(p-value)	Ratio	z-score	Molecules
Colorectal Cancer Metastasis Signaling	2Gy 4h	1.14	0.31	0.33	ADCY8,AKT1,APPL1,AXIN1,BCL2L1,CASP3,CTNNB1,EGFR,FOS,FZD1,GNG5,GSK3B,HRAS,IFNGR1,IL6R,IL6ST,JAK2,KRAS,LEF1,MAP2K1,MAPK8,MMP11,MSH6,NFKB1,PGF,PIK3CB,PIK3CD,PRKAR2A,PTGER1,PTGER2,PTGER3,PTGS2,RAC3,RHOA,RRAS2,SMAD2,SMAD3,SMAD4,SRC,TCF4,TCF7,TGFB1,TGFBR1,WNT5A
Communication between Innate and Adaptive Immune Cells	0.05Gy 2h	1.18	0.04	NaN	IL12A,IL6
Communication between Innate and Adaptive Immune Cells	2Gy 2h	0.00	0.04	NaN	CXCL8,IL6
Communication between Innate and Adaptive Immune Cells	2Gy 4h	0.00	0.04	NaN	HLA-E,IL12A
Complement System	2Gy 2h	0.00	0.08	NaN	CD55
Corticotropin Releasing Hormone Signaling	0.05Gy 2h	0.31	0.01	NaN	PTGS2
Corticotropin Releasing Hormone Signaling	0.05Gy 4h	0.45	0.06	1.00	FOS,GNAI2,PRKCD,PRKCI
Corticotropin Releasing Hormone Signaling	2Gy 2h	0.38	0.16	-1.00	ATF4,BDNF,CACNA1A,ELK1,GLI3,GNAO1,JUND,MEF2D,NR4A1,PRKCE,PTGS2
Corticotropin Releasing Hormone Signaling	2Gy 4h	0.38	0.27	0.50	ADCY8,ATF2,ATF4,CACNA1C,ELK1,FOS,GLI3,GNAI2,GNAI3,ITPR2,MAP2K1,NPR3,PRKAR2A,PRKCD,PRKCE,PRKCI,PRKD3,PTCH1,PTGS2
Creatine-phosphate Biosynthesis	2Gy 4h	0.60	1.00	NaN	MAP4K4
CREB Signaling in Neurons	0.05Gy 4h	0.44	0.06	NaN	GNAI2,GNG5,PRKCD,PRKCI
CREB Signaling in Neurons	2Gy 2h	0.00	0.14	-1.13	ATF4,CACNA1A,CAMK2G,ELK1,GNAO1,PIK3CB,PIK3CD,PRKCE,RRAS2,SOS1
CREB Signaling in Neurons	2Gy 4h	0.61	0.29	-0.23	ADCY8,AKT1,ATF2,ATF4,CACNA1C,ELK1,GNAI2,GNAI3,GNG5,HRAS,ITPR2,KRAS,MAP2K1,PIK3CB,PIK3CD,PRKAR2A,PRKCD,PRKCE,PRKCI,PRKD3,RRAS2
Crosstalk between Dendritic Cells and Natural Killer Cells	0.05Gy 2h	1.26	0.05	NaN	IL12A,IL6
Crosstalk between Dendritic Cells and Natural Killer Cells	0.05Gy 4h	0.00	0.02	NaN	ACTA2
Crosstalk between Dendritic Cells and Natural Killer Cells	2Gy 2h	0.00	0.12	NaN	ACTA2,CAMK2G,FAS,IL6,TNFRSF1B
Crosstalk between Dendritic Cells and Natural Killer Cells	2Gy 4h	0.00	0.22	1.63	ACTA2,FAS,HLA-E,IL12A,MICA,NECTIN2,NFKB1,TNFRSF1B,TNFSF10
CTLA4 Signaling in Cytotoxic T Lymphocytes	0.05Gy 4h	0.21	0.05	NaN	JAK2
CTLA4 Signaling in Cytotoxic T Lymphocytes	2Gy 2h	0.00	0.14	NaN	PIK3CB,PIK3CD,PPP2CA
CTLA4 Signaling in Cytotoxic T Lymphocytes	2Gy 4h	0.31	0.27	NaN	AKT1,HLA-E,JAK2,PIK3CB,PIK3CD,PPP2CA
CXCR4 Signaling	0.05Gy 2h	0.29	0.01	NaN	EGR1
**CXCR4 Signaling**	**0.05Gy 4h**	**1.87**	**0.11**	**1.89**	**EGR1,FOS,GNAI2,GNG5,PRKCD,PRKCI,RHOA,RHOG**
CXCR4 Signaling	2Gy 2h	0.58	0.17	-0.30	CDC42,EGR1,ELK1,GNAO1,MAPK8,PIK3CB,PIK3CD,PRKCE,RAC1,RHOB,RHOQ,RND3,RRAS2
**CXCR4 Signaling**	**2Gy 4h**	**1.60**	**0.36**	**-0.60**	**ADCY8,AKT1,BCAR1,CRK,EGR1,ELK1,FOS,GNAI2,GNAI3,GNG5,HRAS,ITPR2,KRAS,MAP2K1,MAPK8,PIK3CB,PIK3CD,PRKCD,PRKCE,PRKCI,PRKD3,RAC3,RHOA,ROCK1,ROCK2,RRAS2,SRC**
Cyclins and Cell Cycle Regulation	0.05Gy 2h	0.52	0.03	NaN	CDKN1B
Cyclins and Cell Cycle Regulation	0.05Gy 4h	1.13	0.11	-1.00	CDKN1A,E2F4,E2F5,TGFB1
Cyclins and Cell Cycle Regulation	2Gy 2h	0.00	0.13	-1.00	CCNE1,CDKN1A,CDKN1B,HDAC6,PPP2CA
**Cyclins and Cell Cycle Regulation**	**2Gy 4h**	**2.21**	**0.45**	**-1.81**	**ABL1,ATM,CCNA2,CCNB1,CCNE1,CCNE2,CCNH,CDKN1A,CDKN2A,CDKN2B,CDKN2D,E2F1,E2F5,GSK3B,PA2G4,PPP2CA,TGFB1**
Cytotoxic T Lymphocyte-mediated Apoptosis of Target Cells	0.05Gy 4h	0.39	0.08	NaN	BCL2

Ingenuity Canonical Pathways	Model	-log(p-value)	Ratio	z-score	Molecules
Cytotoxic T Lymphocyte-mediated Apoptosis of Target Cells	2Gy 2h	0.63	0.25	NaN	CASP3,CASP8,FAS
Cytotoxic T Lymphocyte-mediated Apoptosis of Target Cells	2Gy 4h	0.80	0.42	1.00	BCL2,BID,CASP3,FAS,HLA-E
Death Receptor Signaling	0.05Gy 4h	0.65	0.08	NaN	ACTA2,BCL2,LMNA
**Death Receptor Signaling**	**2Gy 2h**	**2.76**	**0.33**	**0.83**	**ACTA2,BIRC2,CASP3,CASP8,CFLAR,FAS,IKBKB,IKBKE,MAPK8,PARP2,RIPK1,TNFRSF10B,TNFRSF1B**
**Death Receptor Signaling**	**2Gy 4h**	**3.46**	**0.51**	**1.34**	**ACTA2,BCL2,BID,CASP3,CFLAR,FAS,LMNA,MAP4K4,MAPK8,NFKB1,NFKBIA,PARP2,PARP9,RIPK1,ROCK1,TNFRSF10A,TNFRSF10B,TNFRSF1B,TNFSF10,XIAP**
Dendritic Cell Maturation	0.05Gy 2h	0.80	0.03	NaN	IL12A,IL6
Dendritic Cell Maturation	0.05Gy 4h	0.00	0.01	NaN	JAK2
Dendritic Cell Maturation	2Gy 2h	0.00	0.13	-0.63	ATF4,CD58,IKBKB,IKBKE,IL6,MAPK8,PIK3CB,PIK3CD,RELB,TNFRSF1B
Dendritic Cell Maturation	2Gy 4h	0.00	0.21	-0.78	AKT1,ATF2,ATF4,HLA-E,ICAM1,IL12A,JAK2,LEPR,MAPK8,NFKB1,NFKBIA,NGFR,PIK3CB,PIK3CD,TNFRSF11B,TNFRSF1B
Dermatan Sulfate Biosynthesis	0.05Gy 4h	0.37	0.08	NaN	HS6ST1
Dermatan Sulfate Biosynthesis	2Gy 2h	0.56	0.23	NaN	CHST11,CHST2,HS6ST1
Dermatan Sulfate Biosynthesis	2Gy 4h	0.00	0.08	NaN	HS6ST1
Dermatan Sulfate Biosynthesis (Late Stages)	0.05Gy 4h	0.39	0.08	NaN	HS6ST1
Dermatan Sulfate Biosynthesis (Late Stages)	2Gy 2h	0.63	0.25	NaN	CHST11,CHST2,HS6ST1
Dermatan Sulfate Biosynthesis (Late Stages)	2Gy 4h	0.00	0.08	NaN	HS6ST1
Dermatan Sulfate Degradation (Metazoa)	2Gy 4h	0.43	0.40	NaN	CEMIP,IDUA
Differential Regulation of Cytokine Production in Intestinal Epithelial Cells by IL-17A and IL-17F	0.05Gy 2h	0.80	0.06	NaN	IL12A
Differential Regulation of Cytokine Production in Intestinal Epithelial Cells by IL-17A and IL-17F	2Gy 2h	0.00	0.11	NaN	CCL2,CXCL1
Differential Regulation of Cytokine Production in Intestinal Epithelial Cells by IL-17A and IL-17F	2Gy 4h	0.00	0.06	NaN	IL12A
**Differential Regulation of Cytokine Production in Macrophages and T Helper Cells by IL-17A and IL-17F**	**0.05Gy 2h**	**2.14**	**0.14**	**NaN**	**IL12A,IL6**
Differential Regulation of Cytokine Production in Macrophages and T Helper Cells by IL-17A and IL-17F	2Gy 2h	0.50	0.21	NaN	CCL2,CXCL1,IL6
Differential Regulation of Cytokine Production in Macrophages and T Helper Cells by IL-17A and IL-17F	2Gy 4h	0.00	0.07	NaN	IL12A
D-myo-inositol (1,3,4)-trisphosphate Biosynthesis	2Gy 4h	0.24	0.33	NaN	PTEN
D-myo-inositol (1,4,5)-trisphosphate Degradation	2Gy 2h	0.44	0.33	NaN	IMPA2
D-myo-inositol (1,4,5,6)-Tetrakisphosphate Biosynthesis	0.05Gy 2h	0.76	0.05	NaN	SOCS3
D-myo-inositol (1,4,5,6)-Tetrakisphosphate Biosynthesis	0.05Gy 4h	0.69	0.10	NaN	CDC25C,PTPN13
D-myo-inositol (1,4,5,6)-Tetrakisphosphate Biosynthesis	2Gy 2h	0.51	0.20	-1.00	CDC25B,DUSP1,PTPN2,SOCS3
**D-myo-inositol (1,4,5,6)-Tetrakisphosphate Biosynthesis**	**2Gy 4h**	**1.39**	**0.45**	**0.33**	**CDC25B,DUSP1,NUDT11,PAWR,PPP1CA,PPP3CA,PTEN,PTPN13,SOCS3**
D-myo-inositol (3,4,5,6)-tetrakisphosphate Biosynthesis	0.05Gy 2h	0.76	0.05	NaN	SOCS3
D-myo-inositol (3,4,5,6)-tetrakisphosphate Biosynthesis	0.05Gy 4h	0.69	0.10	NaN	CDC25C,PTPN13

Ingenuity Canonical Pathways	Model	-log(p-value)	Ratio	z-score	Molecules
D-myo-inositol (3,4,5,6)-tetrakisphosphate Biosynthesis	2Gy 2h	0.51	0.20	-1.00	CDC25B,DUSP1,PTPN2,SOCS3
**D-myo-inositol (3,4,5,6)-tetrakisphosphate Biosynthesis**	**2Gy 4h**	**1.39**	**0.45**	**0.33**	**CDC25B,DUSP1,NUDT11,PAWR,PPP1CA,PPP3CA,PTEN,PTPN13,SOCS3**
D-myo-inositol-5-phosphate Metabolism	0.05Gy 2h	0.69	0.04	NaN	SOCS3
D-myo-inositol-5-phosphate Metabolism	0.05Gy 4h	0.57	0.08	NaN	CDC25C,PTPN13
D-myo-inositol-5-phosphate Metabolism	2Gy 2h	0.35	0.17	-1.00	CDC25B,DUSP1,PTPN2,SOCS3
D-myo-inositol-5-phosphate Metabolism	2Gy 4h	0.92	0.38	0.33	CDC25B,DUSP1,NUDT11,PAWR,PPP1CA,PPP3CA,PTEN,PTPN13,SOCS3
DNA damage-induced 14-3-3Ïƒ Signaling	2Gy 2h	0.00	0.09	NaN	CCNE1
DNA damage-induced 14-3-3Ïƒ Signaling	2Gy 4h	0.94	0.46	NaN	AKT1,ATM,CCNB1,CCNE1,CCNE2
**DNA Double-Strand Break Repair by Homologous Recombination**	**2Gy 4h**	**1.42**	**0.67**	**NaN**	**ABL1,ATM,BRCA2,LIG1**
DNA Double-Strand Break Repair by Non-Homologous End Joining	2Gy 4h	0.00	0.20	NaN	ATM
DNA Methylation and Transcriptional Repression Signaling	2Gy 2h	0.38	0.20	NaN	MTA1,MTA2
DNA Methylation and Transcriptional Repression Signaling	2Gy 4h	0.00	0.20	NaN	MECP2,RBBP7
Docosahexaenoic Acid (DHA) Signaling	0.05Gy 4h	0.22	0.05	NaN	BCL2
Docosahexaenoic Acid (DHA) Signaling	2Gy 2h	0.79	0.24	NaN	BCL2L1,CASP3,PDPK1,PIK3CB,PIK3CD
Docosahexaenoic Acid (DHA) Signaling	2Gy 4h	0.89	0.38	NaN	AKT1,BCL2,BCL2L1,BID,CASP3,GSK3B,PIK3CB,PIK3CD
Dolichyl-diphosphooligosaccharide Biosynthesis	2Gy 2h	0.58	0.50	NaN	ALG5
Dopamine Receptor Signaling	2Gy 2h	0.00	0.08	NaN	GCH1,PPP2CA
Dopamine Receptor Signaling	2Gy 4h	0.00	0.19	NaN	ADCY8,GCH1,PPP1CA,PPP2CA,PRKAR2A
Dopamine-DARPP32 Feedback in cAMP Signaling	0.05Gy 4h	0.28	0.05	NaN	GNAI2,PRKCD,PRKCI
Dopamine-DARPP32 Feedback in cAMP Signaling	2Gy 2h	0.00	0.09	-0.82	ATF4,CACNA1A,PPP2CA,PPP3CC,PPP3R1,PRKCE
**Dopamine-DARPP32 Feedback in cAMP Signaling**	**2Gy 4h**	**1.41**	**0.35**	**-1.89**	**ADCY8,ATF2,ATF4,ATP2A2,CACNA1C,CREM,GNAI2,GNAI3,ITPR2,KCNJ15,KCNJ8,PAWR,PPP1CA,PPP2CA,PPP3CA,PPP3CB,PPP3CC,PPP3R1,PRKAR2A,PRKCD,PRKCE,PRKCI,PRKD3**
EGF Signaling	0.05Gy 4h	0.00	0.03	NaN	FOS
EGF Signaling	2Gy 2h	0.54	0.19	-0.82	ELK1,MAPK8,MTOR,PIK3CB,PIK3CD,SOS1
**EGF Signaling**	**2Gy 4h**	**1.81**	**0.44**	**0.00**	**AKT1,CSNK2B,EGFR,ELK1,FOS,HRAS,ITPR2,MAP2K1,MAP3K1,MAPK8,PIK3CB,PIK3CD,SRC,SRF**
Eicosanoid Signaling	0.05Gy 2h	0.61	0.03	NaN	PTGS2
Eicosanoid Signaling	2Gy 2h	0.00	0.10	NaN	PNPLA8,PTGER4,PTGS2
Eicosanoid Signaling	2Gy 4h	0.00	0.23	NaN	PNPLA8,PTGER1,PTGER2,PTGER3,PTGES,PTGES2,PTGS2
EIF2 Signaling	0.05Gy 2h	0.25	0.01	NaN	MYC
EIF2 Signaling	0.05Gy 4h	0.31	0.05	NaN	ACTA2,BCL2,PTBP1,RPL28
EIF2 Signaling	2Gy 2h	0.00	0.11	-0.38	ACTA2,ATF4,MYC,PDPK1,PIK3CB,PIK3CD,PTBP1,RRAS2,SOS1
**EIF2 Signaling**	**2Gy 4h**	**1.86**	**0.36**	**1.63**	**ACTA2,AKT1,ATF4,BCL2,DDIT3,EIF2AK1,EIF2AK2,EIF4A2,FAU,GSK3B,HRAS,KRAS,MAP2K1,NOX4,PIK3CB,PIK3CD,PPP1CA,PTBP1,RPL10,RPL28,RPL30,RPL36,RPL37A,RPL41,RPLP1,RPS19,RPS2,RPS29,RRAS2,TRIB3,XIAP**
**Endocannabinoid Cancer Inhibition Pathway**	**0.05Gy 2h**	**1.46**	**0.04**	**NaN**	**CDKN1B,MYC,SNAI2**
Endocannabinoid Cancer Inhibition Pathway	0.05Gy 4h	0.00	0.04	NaN	CDKN1A,GNAI2,RHOA
Endocannabinoid Cancer Inhibition Pathway	2Gy 2h	0.53	0.17	0.28	ATF4,CASP3,CASP8,CCNE1,CDKN1A,CDKN1B,GNAO1,MAP2K5,MTOR,MYC,PIK3CB,PIK3CD,SNAI2

Ingenuity Canonical Pathways	Model	-log(p-value)	Ratio	z-score	Molecules
**Endocannabinoid Cancer Inhibition Pathway**	**2Gy 4h**	**1.70**	**0.36**	**0.96**	**ADCY8,AKT1,ATF2,ATF4,CASP3,CCNE1,CCNE2,CDKN1A,CTNNB1,DDIT3,GNAI2,GNAI3,GSK3B,HIF1A,LEF1,MAP2K1,PGF,PIK3CB,PIK3CD,PRKAR2A,RHOA,ROCK1,ROCK2,SNAI2,SRC,TCF4,TCF7,TRIB3**
Endocannabinoid Developing Neuron Pathway	0.05Gy 2h	0.38	0.02	NaN	CDKN1B
Endocannabinoid Developing Neuron Pathway	0.05Gy 4h	0.36	0.05	NaN	GNAI2,GNG5,RHOA
Endocannabinoid Developing Neuron Pathway	2Gy 2h	0.77	0.19	-0.91	ATF4,CDKN1B,GNAO1,MAP2K5,MAPK7,MAPK8,MTOR,PIK3CB,PIK3CD,RAC1,RRAS2
Endocannabinoid Developing Neuron Pathway	2Gy 4h	1.25	0.35	0.69	ADCY8,AKT1,ATF2,ATF4,CTNNB1,GNAI2,GNAI3,GNG5,GSK3B,HRAS,KRAS,MAP2K1,MAPK8,PIK3CB,PIK3CD,PRKAR2A,RAC3,RHOA,RRAS2,SRC
Endocannabinoid Neuronal Synapse Pathway	0.05Gy 2h	0.46	0.02	NaN	PTGS2
Endocannabinoid Neuronal Synapse Pathway	0.05Gy 4h	0.24	0.04	NaN	GNAI2,GNG5
Endocannabinoid Neuronal Synapse Pathway	2Gy 2h	0.53	0.18	-0.71	CACNA1A,GNAO1,MAPK7,MAPK8,NAPEPLD,PPP3CC,PPP3R1,PTGS2
Endocannabinoid Neuronal Synapse Pathway	2Gy 4h	0.49	0.29	-1.39	ADCY8,CACNA1C,DAGLA,GNAI2,GNAI3,GNG5,MAPK8,PPP3CA,PPP3CB,PPP3CC,PPP3R1,PRKAR2A,PTGS2
Endometrial Cancer Signaling	0.05Gy 2h	0.57	0.03	NaN	MYC
Endometrial Cancer Signaling	2Gy 2h	0.76	0.21	-1.13	ELK1,MYC,PDPK1,PIK3CB,PIK3CD,RRAS2,SOS1
**Endometrial Cancer Signaling**	**2Gy 4h**	**1.68**	**0.42**	**-0.30**	**AKT1,AXIN1,CTNNB1,ELK1,FOXO3,GSK3B,HRAS,KRAS,LEF1,MAP2K1,PIK3CB,PIK3CD,PTEN,RRAS2**
Endoplasmic Reticulum Stress Pathway	2Gy 2h	0.71	0.27	NaN	ATF4,CASP3,MAPK8
**Endoplasmic Reticulum Stress Pathway**	**2Gy 4h**	**1.46**	**0.55**	**0.00**	**ATF4,ATF6,CALR,CASP3,DDIT3,MAPK8**
Endothelin-1 Signaling	0.05Gy 2h	0.75	0.02	NaN	MYC,PTGS2
Endothelin-1 Signaling	0.05Gy 4h	0.34	0.05	1.00	FOS,GNAI2,PRKCD,PRKCI
Endothelin-1 Signaling	2Gy 2h	1.22	0.21	-1.21	CASP3,CASP8,EDN1,GNAO1,HMOX1,MAPK7,MAPK8,MYC,NAPEPLD,PIK3CB,PIK3CD,PLD1,PNPLA8,PRKCE,PTGS2,RRAS2,SOS1
Endothelin-1 Signaling	2Gy 4h	0.41	0.27	-0.85	ADCY8,CASP3,FOS,GNAI2,GNAI3,HMOX1,HRAS,ITPR2,KRAS,MAPK8,PIK3CB,PIK3CD,PLD1,PNPLA8,PRKCD,PRKCE,PRKCI,PRKD3,PTGER2,PTGS2,RRAS2,SRC
eNOS Signaling	0.05Gy 4h	0.31	0.05	NaN	AQP5,PRKCD,PRKCI,SLC7A1
eNOS Signaling	2Gy 2h	0.00	0.12	-1.90	BDKRB1,CASP3,CASP8,CHRM3,HSPA9,LPAR6,PDPK1,PIK3CB,PIK3CD,PRKCE
eNOS Signaling	2Gy 4h	0.33	0.26	-1.00	ADCY8,AKT1,AQP3,AQP5,CASP3,CCNA2,FLT1,HSP90AB1,HSPA6,HSPA8,ITPR2,LPAR1,PGF,PIK3CB,PIK3CD,PRKAR2A,PRKCD,PRKCE,PRKCI,PRKD3,SLC7A1,STUB1
Ephrin A Signaling	0.05Gy 4h	1.15	0.13	NaN	EFNA4,RHOA,VAV2
Ephrin A Signaling	2Gy 2h	0.67	0.22	NaN	CDC42,EPHA2,PIK3CB,PIK3CD,RAC1
**Ephrin A Signaling**	**2Gy 4h**	**1.39**	**0.44**	**NaN**	**BCAR1,CFL1,EPHA2,NGFR,PIK3CB,PIK3CD,RHOA,ROCK1,ROCK2,VAV2**
Ephrin B Signaling	0.05Gy 4h	1.24	0.11	NaN	GNAI2,GNG5,RHOA,VAV2
Ephrin B Signaling	2Gy 2h	0.43	0.17	0.82	CDC42,EFNB1,EPHB3,EPHB4,GNAO1,RAC1
**Ephrin B Signaling**	**2Gy 4h**	**1.44**	**0.40**	**0.58**	**AXIN1,CFL1,CTNNB1,EFNB1,EPHB4,GNAI2,GNAI3,GNG5,HRAS,RAC3,RHOA,ROCK1,ROCK2,VAV2**
Ephrin Receptor Signaling	0.05Gy 4h	0.89	0.07	1.34	EFNA4,GNAI2,GNG5,JAK2,PTPN13,RHOA
Ephrin Receptor Signaling	2Gy 2h	0.77	0.18	-1.27	ATF4,CDC42,DOK1,EFNB1,EPHA2,EPHB3,EPHB4,GNAO1,ITGA5,LIMK2,NCK1,PDGFA,RAC1,RRAS2,SOS1
**Ephrin Receptor Signaling**	**2Gy 4h**	**1.65**	**0.35**	**0.21**	**ABL1,AKT1,ATF2,ATF4,AXIN1,BCAR1,CFL1,CRK,EFNB1,EPHA2,EPHB4,GNAI2,GNAI3,GNG5,HRAS,JAK2,KRAS,LIMK2,MAP2K1,MAP4K4,PGF,PTPN13,RAC3,RHOA,ROCK1,ROCK2,RRAS2,SORBS1,SRC**
Epithelial Adherens Junction Signaling	0.05Gy 2h	0.33	0.02	NaN	SNAI2
Epithelial Adherens Junction Signaling	0.05Gy 4h	0.84	0.08	NaN	ACTA2,RHOA,TGFBR1,VAV2,YES1
Epithelial Adherens Junction Signaling	2Gy 2h	0.00	0.14	NaN	ACTA2,ACVR2A,CDC42,RAC1,RRAS2,SNAI1,SNAI2,TUBB2A,TUBB4B

Ingenuity Canonical Pathways	Model	-log(p-value)	Ratio	z-score	Molecules
**Epithelial Adherens Junction Signaling**	**2Gy 4h**	**2.52**	**0.41**	**NaN**	**ACTA2,ACVR1,ACVR1B,ACVR2A,AKT1,BMPR2,CRK,CTNNB1,EGFR,HRAS,KRAS,LEF1,NECTIN1,NECTIN2,NOTCH4,PTEN,RHOA,RRAS2,SNAI2,SORBS1,SRC,TCF4,TCF7,TGFBR1,TUBB2A,VAV2,YES1**
ErbB Signaling	0.05Gy 4h	0.44	0.06	NaN	FOS,PRKCD,PRKCI
**ErbB Signaling**	**2Gy 2h**	**1.34**	**0.24**	**-0.58**	**AREG,CDC42,ELK1,MAPK8,MTOR,NCK1,PDPK1,PIK3CB,PIK3CD,PRKCE,RRAS2,SOS1**
ErbB Signaling	2Gy 4h	0.73	0.31	0.00	AKT1,EGFR,ELK1,FOS,GSK3B,HRAS,KRAS,MAP2K1,MAPK8,PIK3CB,PIK3CD,PRKCD,PRKCE,PRKCI,PRKD3,RRAS2
**ErbB2-ErbB3 Signaling**	**0.05Gy 2h**	**1.45**	**0.06**	**NaN**	**CDKN1B,MYC**
**ErbB2-ErbB3 Signaling**	**2Gy 2h**	**1.55**	**0.28**	**-1.41**	**CDKN1B,ELK1,MYC,PDPK1,PIK3CB,PIK3CD,RRAS2,SOS1,TYK2**
ErbB2-ErbB3 Signaling	2Gy 4h	0.81	0.34	-0.30	AKT1,ELK1,GSK3B,HRAS,KRAS,MAP2K1,PIK3CB,PIK3CD,PTEN,RRAS2,SP1
ErbB4 Signaling	0.05Gy 4h	0.36	0.06	NaN	PRKCD,PRKCI
ErbB4 Signaling	2Gy 2h	0.43	0.17	-1.63	PDPK1,PIK3CB,PIK3CD,PRKCE,RRAS2,SOS1
**ErbB4 Signaling**	**2Gy 4h**	**1.44**	**0.40**	**0.00**	**ADAM17,AKT1,HRAS,KRAS,MAP2K1,PIK3CB,PIK3CD,PRKCD,PRKCE,PRKCI,PRKD3,PSEN2,RRAS2,YAP1**
ERK/MAPK Signaling	0.05Gy 2h	0.30	0.01	NaN	MYC
ERK/MAPK Signaling	0.05Gy 4h	0.43	0.05	1.00	FOS,NFATC1,PRKCD,PRKCI
ERK/MAPK Signaling	2Gy 2h	0.86	0.19	-0.54	ATF4,DUSP1,DUSP6,ELK1,ITGA5,MYC,NFATC1,PIK3CB,PIK3CD,PPP2CA,PRKCE,RAC1,RRAS2,SOS1
**ERK/MAPK Signaling**	**2Gy 4h**	**1.84**	**0.37**	**-0.58**	**ATF2,ATF4,BCAR1,CRK,DUSP1,DUSP6,ELK1,ETS1,ETS2,FOS,HRAS,KRAS,MAP2K1,NFATC1,PIK3CB,PIK3CD,PPP1CA,PPP2CA,PRKAR2A,PRKCD,PRKCE,PRKCI,PTK2B,RAC3,RRAS2,SRC,SRF**
ERK5 Signaling	0.05Gy 2h	0.63	0.04	NaN	MYC
ERK5 Signaling	0.05Gy 4h	0.00	0.04	NaN	FOS
ERK5 Signaling	2Gy 2h	1.06	0.25	-1.13	ATF4,MAP2K5,MAPK7,MEF2D,MYC,RRAS2,SGK1
**ERK5 Signaling**	**2Gy 4h**	**1.54**	**0.43**	**0.58**	**AKT1,ATF2,ATF4,EGFR,FOS,FOXO3,HRAS,IL6ST,KRAS,LIF,RRAS2,SRC**
Erythropoietin Signaling	0.05Gy 2h	0.48	0.02	NaN	SOCS3
Erythropoietin Signaling	0.05Gy 4h	1.01	0.10	NaN	FOS,JAK2,PRKCD,PRKCI
Erythropoietin Signaling	2Gy 2h	0.90	0.21	NaN	ELK1,PDPK1,PIK3CB,PIK3CD,PRKCE,RRAS2,SOCS1,SOCS3,SOS1
**Erythropoietin Signaling**	**2Gy 4h**	**2.08**	**0.43**	**NaN**	**AKT1,ELK1,FOS,HRAS,JAK2,KRAS,MAP2K1,NFKB1,NFKBIA,PIK3CB,PIK3CD,PRKCD,PRKCE,PRKCI,PRKD3,RRAS2,SOCS3,SRC**
Estrogen Biosynthesis	2Gy 4h	0.00	0.10	NaN	CYP1B1,HSD17B10
Estrogen Receptor Signaling	0.05Gy 4h	0.36	0.06	NaN	NR3C1,SMARCA4
Estrogen Receptor Signaling	2Gy 2h	0.43	0.17	NaN	CCNC,NCOR2,PPARGC1A,RRAS2,RUNX2,SOS1
Estrogen Receptor Signaling	2Gy 4h	0.43	0.29	NaN	CCNH,HRAS,KRAS,MAP2K1,NCOA2,NR3C1,RRAS2,SMARCA4,SRC,THRAP3
Estrogen-Dependent Breast Cancer Signaling	0.05Gy 4h	0.00	0.03	NaN	FOS
Estrogen-Dependent Breast Cancer Signaling	2Gy 2h	0.25	0.14	-0.45	ATF4,ELK1,PIK3CB,PIK3CD,RRAS2
**Estrogen-Dependent Breast Cancer Signaling**	**2Gy 4h**	**1.81**	**0.43**	**-0.54**	**AKT1,ATF2,ATF4,EGFR,ELK1,FOS,HRAS,HSD17B10,KRAS,NFKB1,PIK3CB,PIK3CD,RRAS2,SP1,SRC**
**Estrogen-mediated S-phase Entry**	**0.05Gy 2h**	**1.93**	**0.11**	**NaN**	**CDKN1B,MYC**
**Estrogen-mediated S-phase Entry**	**0.05Gy 4h**	**1.42**	**0.17**	**NaN**	**CDKN1A,E2F4,E2F5**
Estrogen-mediated S-phase Entry	2Gy 2h	0.62	0.22	-1.00	CCNE1,CDKN1A,CDKN1B,MYC
Estrogen-mediated S-phase Entry	2Gy 4h	0.55	0.33	-2.45	CCNA2,CCNE1,CCNE2,CDKN1A,E2F1,E2F5
Extrinsic Prothrombin Activation Pathway	2Gy 2h	0.00	0.09	NaN	F3
Extrinsic Prothrombin Activation Pathway	2Gy 4h	0.00	0.09	NaN	THBD
Factors Promoting Cardiogenesis in Vertebrates	0.05Gy 4h	0.63	0.07	NaN	PRKCD,PRKCI,TGFB1,TGFBR1

Ingenuity Canonical Pathways	Model	-log(p-value)	Ratio	z-score	Molecules
Factors Promoting Cardiogenesis in Vertebrates	2Gy 2h	0.00	0.08	NaN	ACVR2A,CCNE1,CDC6,PRKCE,TBX5
**Factors Promoting Cardiogenesis in Vertebrates**	**2Gy 4h**	**4.34**	**0.49**	**NaN**	**ACVR1,ACVR1B,ACVR2A,ATF2,AXIN1,BMP1,BMP2,BMP4,BMPR2,CCNE1,CCNE2,CDC6,CTNNB1,DKK1,FZD1,GSK3B,LEF1,MAP3K7,NOX4,PRKCD,PRKCE,PRKCI,PRKD3,SMAD2,SMAD4,TCF4,TCF7,TGFB1,TGFBR1**
FAK Signaling	0.05Gy 4h	0.00	0.02	NaN	ACTA2
FAK Signaling	2Gy 2h	0.60	0.19	NaN	ACTA2,ITGA5,PDPK1,PIK3CB,PIK3CD,RAC1,RRAS2,SOS1
FAK Signaling	2Gy 4h	0.58	0.30	NaN	ACTA2,AKT1,BCAR1,CRK,EGFR,HRAS,KRAS,MAP2K1,PIK3CB,PIK3CD,PTEN,RRAS2,SRC
FAT10 Cancer Signaling Pathway	0.05Gy 2h	0.54	0.03	NaN	IL6
FAT10 Cancer Signaling Pathway	0.05Gy 4h	0.34	0.06	NaN	TGFB1,TGFBR1
FAT10 Cancer Signaling Pathway	2Gy 2h	0.63	0.19	-1.89	ACVR2A,IKBKB,IKBKE,IL6,PCNA,SMAD3,TNFRSF1B
**FAT10 Cancer Signaling Pathway**	**2Gy 4h**	**3.52**	**0.53**	**-2.07**	**ACVR1,ACVR1B,ACVR2A,AKT1,BMPR2,CTNNB1,GSK3B,NFKB1,NFKBIA,NGFR,PCNA,SMAD2,SMAD3,SMAD4,TCF4,TGFB1,TGFBR1,TNFRSF11B,TNFRSF1B**
FAT10 Signaling Pathway	2Gy 2h	0.58	0.50	NaN	MAP1LC3B
FAT10 Signaling Pathway	2Gy 4h	0.36	0.50	NaN	MAP1LC3B
Fatty Acid Î±-oxidation	0.05Gy 2h	1.19	0.14	NaN	PTGS2
Fatty Acid Î±-oxidation	2Gy 2h	0.18	0.14	NaN	PTGS2
Fatty Acid Î±-oxidation	2Gy 4h	0.00	0.14	NaN	PTGS2
Fatty Acid Î²-oxidation I	2Gy 2h	0.00	0.13	NaN	SCP2
Fatty Acid Î²-oxidation I	2Gy 4h	0.00	0.13	NaN	HSD17B10
Fc Epsilon RI Signaling	0.05Gy 4h	0.51	0.07	NaN	PRKCD,PRKCI,VAV2
Fc Epsilon RI Signaling	2Gy 2h	0.50	0.17	-0.71	MAPK8,PDPK1,PIK3CB,PIK3CD,PRKCE,RAC1,RRAS2,SOS1
Fc Epsilon RI Signaling	2Gy 4h	0.61	0.30	0.54	AKT1,HRAS,KRAS,MAP2K1,MAPK8,PIK3CB,PIK3CD,PRKCD,PRKCE,PRKCI,PRKD3,RAC3,RRAS2,VAV2
**FcÎ³ Receptor-mediated Phagocytosis in Macrophages and Monocytes**	**0.05Gy 4h**	**2.02**	**0.14**	**0.00**	**ACTA2,ARF6,PRKCD,PRKCI,VAV2,YES1**
FcÎ³ Receptor-mediated Phagocytosis in Macrophages and Monocytes	2Gy 2h	0.81	0.21	-0.33	ACTA2,ARF6,CDC42,HMOX1,NAPEPLD,NCK1,PLD1,PRKCE,RAC1
**FcÎ³ Receptor-mediated Phagocytosis in Macrophages and Monocytes**	**2Gy 4h**	**1.51**	**0.39**	**0.24**	**ACTA2,AKT1,ARF6,CRK,EZR,HMOX1,PLD1,PRKCD,PRKCE,PRKCI,PRKD3,PTEN,PTK2B,RAC3,SRC,VAV2,YES1**
**FcÎ³RIIB Signaling in B Lymphocytes**	**2Gy 2h**	**2.12**	**0.36**	**-1.13**	**CACNA1A,DOK1,MAPK8,PDPK1,PIK3CB,PIK3CD,RRAS2,SOS1**
FcÎ³RIIB Signaling in B Lymphocytes	2Gy 4h	0.79	0.36	-0.38	AKT1,CACNA1C,HRAS,KRAS,MAPK8,PIK3CB,PIK3CD,RRAS2
FGF Signaling	0.05Gy 4h	0.00	0.03	NaN	FGF5
FGF Signaling	2Gy 2h	1.06	0.23	-1.00	ATF4,FGF2,FGF7,FGF9,MAPK8,PIK3CB,PIK3CD,RAC1,SOS1
FGF Signaling	2Gy 4h	0.60	0.31	0.00	AKT1,ATF2,ATF4,CRK,FGF2,FGF5,HRAS,MAP2K1,MAP3K1,MAPK8,PIK3CB,PIK3CD
**FLT3 Signaling in Hematopoietic Progenitor Cells**	**2Gy 2h**	**1.30**	**0.27**	**-1.41**	**ATF4,ELK1,MTOR,PDPK1,PIK3CB,PIK3CD,RRAS2,SOS1**
FLT3 Signaling in Hematopoietic Progenitor Cells	2Gy 4h	0.71	0.33	0.00	AKT1,ATF2,ATF4,ELK1,HRAS,KRAS,MAP2K1,PIK3CB,PIK3CD,RRAS2
fMLP Signaling in Neutrophils	0.05Gy 4h	0.95	0.08	1.34	GNAI2,GNG5,NFATC1,PRKCD,PRKCI
fMLP Signaling in Neutrophils	2Gy 2h	0.64	0.18	-0.30	CDC42,ELK1,NFATC1,NFATC4,PIK3CB,PIK3CD,PPP3CC,PPP3R1,PRKCE,RAC1,RRAS2
**fMLP Signaling in Neutrophils**	**2Gy 4h**	**2.05**	**0.39**	**-1.63**	**ELK1,GNAI2,GNAI3,GNG5,HRAS,ITPR2,KRAS,MAP2K1,NFAT5,NFATC1,NFKB1,NFKBIA,NOX4,PIK3CB,PIK3CD,PPP3CA,PPP3CB,PPP3CC,PPP3R1,PRKCD,PRKCE,PRKCI,PRKD3,RRAS2**
Folate Transformations I	0.05Gy 4h	0.92	0.33	NaN	MTHFD2
Folate Transformations I	2Gy 2h	0.44	0.33	NaN	MTHFD2
FXR/RXR Activation	2Gy 2h	0.00	0.06	NaN	APOE,MAPK8,PPARGC1A,RXRA,SLC4A2
FXR/RXR Activation	2Gy 4h	0.00	0.09	NaN	AKT1,MAPK8,NR1H3,PPARA,RARA,SDC1,VLDLR
G Beta Gamma Signaling	0.05Gy 4h	0.85	0.08	1.00	GNAI2,GNG5,PRKCD,PRKCI

Ingenuity Canonical Pathways	Model	-log(p-value)	Ratio	z-score	Molecules
G Beta Gamma Signaling	2Gy 2h	0.44	0.17	-0.71	CACNA1A,CAV2,CDC42,GNAO1,PDPK1,PRKCE,RRAS2,SOS1
G Beta Gamma Signaling	2Gy 4h	0.70	0.31	-0.26	AKT1,CACNA1C,EGFR,GNAI2,GNAI3,GNG5,HRAS,KRAS,PRKAR2A,PRKCD,PRKCE,PRKCI,PRKD3,RRAS2,SRC
G Protein Signaling Mediated by Tubby	0.05Gy 4h	0.94	0.14	NaN	GNG5,JAK2
G Protein Signaling Mediated by Tubby	2Gy 4h	0.00	0.21	NaN	ABL1,GNG5,JAK2
GABA Receptor Signaling	2Gy 2h	0.00	0.04	NaN	CACNA1A
GABA Receptor Signaling	2Gy 4h	0.00	0.17	NaN	ADCY8,CACNA1C,GABRB2,UBC
GADD45 Signaling	0.05Gy 4h	0.32	0.07	NaN	CDKN1A
GADD45 Signaling	2Gy 2h	0.45	0.20	NaN	CCNE1,CDKN1A,PCNA
GADD45 Signaling	2Gy 4h	1.25	0.47	NaN	ATM,CCNB1,CCNE1,CCNE2,CDKN1A,GADD45A,PCNA
**Galactose Degradation I (Leloir Pathway)**	**0.05Gy 4h**	**1.37**	**1.00**	**NaN**	**GALK1**
Galactose Degradation I (Leloir Pathway)	2Gy 4h	0.60	1.00	NaN	GALK1
Gap Junction Signaling	0.05Gy 4h	0.44	0.05	NaN	ACTA2,GJC1,GNAI2,PRKCD,PRKCI
Gap Junction Signaling	2Gy 2h	0.00	0.13	NaN	ACTA2,MAP2K5,MAPK7,PIK3CB,PIK3CD,PPP3CC,PPP3R1,PRKCE,RRAS2,SOS1,TUBB2A,TUBB4B
**Gap Junction Signaling**	**2Gy 4h**	**1.31**	**0.33**	**NaN**	**ACTA2,ADCY8,AKT1,CTNNB1,EGFR,GJA1,GJC1,GNAI2,GNAI3,HRAS,ITPR2,KRAS,LPAR1,MAP2K1,NPR3,PIK3CB,PIK3CD,PPP3CA,PPP3CB,PPP3CC,PPP3R1,PRKAR2A,PRKCD,PRKCE,PRKCI,PRKD3,RRAS2,SP1,SRC,TJP1,TUBB2A**
GDNF Family Ligand-Receptor Interactions	0.05Gy 4h	0.00	0.03	NaN	FOS
**GDNF Family Ligand-Receptor Interactions**	**2Gy 2h**	**1.99**	**0.30**	**-0.30**	**CDC42,DOK1,IRS1,IRS2,MAPK8,NCK1,PIK3CB,PIK3CD,RAC1,RRAS2,SOS1**
GDNF Family Ligand-Receptor Interactions	2Gy 4h	0.35	0.27	0.33	FOS,GDNF,HRAS,ITPR2,KRAS,MAP2K1,MAPK8,PIK3CB,PIK3CD,RRAS2
**GDP-mannose Biosynthesis**	**0.05Gy 4h**	**1.37**	**1.00**	**NaN**	**GPI**
GDP-mannose Biosynthesis	2Gy 4h	0.60	1.00	NaN	GPI
Germ Cell-Sertoli Cell Junction Signaling	0.05Gy 4h	1.19	0.09	NaN	ACTA2,RAB8B,RHOA,RHOG,TGFB1,TGFBR1
Germ Cell-Sertoli Cell Junction Signaling	2Gy 2h	1.06	0.21	NaN	ACTA2,CDC42,LIMK2,MAPK8,PDPK1,PIK3CB,PIK3CD,RAC1,RHOB,RHOQ,RND3,RRAS2,TUBB2A,TUBB4B
**Germ Cell-Sertoli Cell Junction Signaling**	**2Gy 4h**	**1.99**	**0.38**	**NaN**	**ACTA2,AKT1,AXIN1,BCAR1,CFL1,CTNNB1,HRAS,KRAS,LIMK2,MAP2K1,MAP3K1,MAP3K7,MAPK8,NECTIN2,PIK3CB,PIK3CD,RAB8B,RAC3,RHOA,RRAS2,SORBS1,SRC,TGFB1,TGFBR1,TJP1,TUBB2A**
GÎ±12/13 Signaling	0.05Gy 4h	0.00	0.04	NaN	RHOA,VAV2
GÎ±12/13 Signaling	2Gy 2h	0.81	0.20	-0.30	CDC42,ELK1,IKBKB,IKBKE,LPAR6,MAPK7,MAPK8,MEF2D,PIK3CB,PIK3CD,RRAS2
**GÎ±12/13 Signaling**	**2Gy 4h**	**1.32**	**0.36**	**-1.15**	**AKT1,CTNNB1,ELK1,HRAS,KRAS,LPAR1,MAP2K1,MAP3K1,MAPK8,NFKB1,NFKBIA,PIK3CB,PIK3CD,PTK2B,RHOA,ROCK1,ROCK2,RRAS2,SRC,VAV2**
GÎ±i Signaling	0.05Gy 4h	0.00	0.04	NaN	GNAI2,GNG5
GÎ±i Signaling	2Gy 2h	0.00	0.09	1.34	RGS12,RGS4,RRAS2,S1PR3,SOS1
GÎ±i Signaling	2Gy 4h	0.00	0.23	-0.28	ADCY8,GNAI2,GNAI3,GNG5,HRAS,KRAS,LPAR1,NPR3,PRKAR2A,PTGER3,RGS4,RRAS2,SRC
**GÎ±q Signaling**	**0.05Gy 4h**	**1.33**	**0.09**	**1.13**	**GNG5,HRH1,NFATC1,PRKCD,PRKCI,RHOA,RHOG**
**GÎ±q Signaling**	**2Gy 2h**	**2.29**	**0.25**	**-1.89**	**CDC42,CHRM3,ELK1,HMOX1,IKBKB,IKBKE,NAPEPLD,NFATC1,NFATC4,PIK3CB,PIK3CD,PLD1,PPP3CC,PPP3R1,PRKCE,RAC1,RGS4,RHOB,RHOQ,RND3**
**GÎ±q Signaling**	**2Gy 4h**	**1.62**	**0.35**	**-1.57**	**AKT1,ELK1,GNG5,GSK3B,HMOX1,HRH1,ITPR2,MAP2K1,NFATC1,NFKB1,NFKBIA,PIK3CB,PIK3CD,PLD1,PPP3CA,PPP3CB,PPP3CC,PPP3R1,PRKCD,PRKCE,PRKCI,PRKD3,PTK2B,RAC3,RGS4,RHOA,ROCK1,ROCK2**
GÎ±s Signaling	0.05Gy 4h	0.00	0.02	NaN	GNG5
GÎ±s Signaling	2Gy 2h	0.00	0.07	-1.00	ATF4,CHRM3,ELK1,PTGER4
GÎ±s Signaling	2Gy 4h	0.00	0.20	0.91	ADCY8,ADORA2B,ADRB2,ATF2,ATF4,ELK1,GNG5,MAP2K1,PRKAR2A,PTGER2,SRC

Ingenuity Canonical Pathways	Model	-log(p-value)	Ratio	z-score	Molecules
Glioblastoma Multiforme Signaling	0.05Gy 2h	0.74	0.02	NaN	CDKN1B,MYC
Glioblastoma Multiforme Signaling	0.05Gy 4h	1.23	0.08	1.34	CDKN1A,E2F4,E2F5,MDM2,PRKCD,RHOA,RHOG
Glioblastoma Multiforme Signaling	2Gy 2h	0.95	0.19	-0.78	CCNE1,CDC42,CDKN1A,CDKN1B,MDM2,MTOR,MYC,PDGFA,PIK3CB,PIK3CD,RAC1,RHOB,RHOQ,RND3,RRAS2,SOS1
Glioblastoma Multiforme Signaling	2Gy 4h	1.13	0.33	-0.22	AKT1,AXIN1,CCNE1,CDKN1A,CDKN2A,CTNNB1,E2F1,E2F5,EGFR,FZD1,GSK3B,HRAS,ITPR2,KRAS,LEF1,MAP2K1,MDM2,PDGFRA,PIK3CB,PIK3CD,PRKCD,PTEN,RAC3,RHOA,RRAS2,SRC,WNT5A
Glioma Invasiveness Signaling	0.05Gy 4h	0.37	0.06	NaN	RHOA,RHOG
**Glioma Invasiveness Signaling**	**2Gy 2h**	**1.39**	**0.27**	**-0.38**	**CDC42,PIK3CB,PIK3CD,PLAUR,RAC1,RHOB,RHOQ,RND3,RRAS2**
Glioma Invasiveness Signaling	2Gy 4h	0.00	0.24	-0.38	CD44,HRAS,KRAS,PIK3CB,PIK3CD,RAC3,RHOA,RRAS2
Glioma Signaling	0.05Gy 4h	1.21	0.09	NaN	CDKN1A,E2F4,E2F5,MDM2,PRKCD,PRKCI
Glioma Signaling	2Gy 2h	0.32	0.15	-1.41	CAMK2G,CDKN1A,MDM2,MTOR,PDGFA,PIK3CB,PIK3CD,PRKCE,RRAS2,SOS1
**Glioma Signaling**	**2Gy 4h**	**1.79**	**0.37**	**-0.26**	**ABL1,AKT1,CAMK1,CDKN1A,CDKN2A,CDKN2B,CDKN2D,E2F1,E2F5,EGFR,HRAS,KRAS,MAP2K1,MDM2,PA2G4,PDGFRA,PIK3CB,PIK3CD,PRKCD,PRKCE,PRKCI,PRKD3,PTEN,RBL2,RRAS2**
Glucocorticoid Receptor Signaling	0.05Gy 2h	0.42	0.01	NaN	IL6,PTGS2
Glucocorticoid Receptor Signaling	0.05Gy 4h	1.16	0.07	NaN	BCL2,CDKN1A,FOS,JAK2,NFATC1,NR3C1,SMARCA4,TGFB1,TGFBR1,UBE2I
**Glucocorticoid Receptor Signaling**	**2Gy 2h**	**1.52**	**0.20**	**NaN**	**BCL2L1,CCL2,CDKN1A,CEBPB,CXCL8,DUSP1,ELK1,HSPA9,IKBKB,IKBKE,IL6,MAPK8,NCOR2,NFATC1,NFATC4,NR3C2,PIK3CB,PIK3CD,PPP3CC,PPP3R1,PTGES3,PTGS2,RAC1,RRAS2,SERPINE1,SGK1,SMAD3,SOS1**
**Glucocorticoid Receptor Signaling**	**2Gy 4h**	**1.50**	**0.32**	**NaN**	**ADRB2,AKT1,BCL2,BCL2L1,CCNH,CDKN1A,DUSP1,ELK1,FOS,FOXO3,HRAS,HSP90AB1,HSPA6,HSPA8,ICAM1,JAK2,KRAS,KRT17,KRT18,MAP2K1,MAP3K1,MAP3K7,MAPK8,NCOA2,NFAT5,NFATC1,NFKB1,NFKBIA,NR3C1,PIK3CB,PIK3CD,PPP3CA,PPP3CB,PPP3CC,PPP3R1,PTGES3,PTGS2,RRAS2,SMAD2,SMAD3,SMAD4,SMARCA4,TGFB1,TGFBR1,UBE2I**
Gluconeogenesis I	0.05Gy 4h	0.80	0.25	NaN	GPI
**Gluconeogenesis I**	**2Gy 4h**	**2.41**	**1.00**	**2.00**	**ENO1,GAPDH,GPI,PGK1**
Glutamate Biosynthesis II	2Gy 4h	0.60	1.00	NaN	GLUD1
Glutamate Degradation X	2Gy 4h	0.60	1.00	NaN	GLUD1
Glutamate Receptor Signaling	0.05Gy 4h	1.12	0.18	NaN	GNG5,SLC38A1
Glutamate Receptor Signaling	2Gy 4h	0.26	0.27	NaN	GLUL,GNG5,SLC38A1
Glutamine Biosynthesis I	2Gy 4h	0.60	1.00	NaN	GLUL
Glutaryl-CoA Degradation	2Gy 4h	0.24	0.33	NaN	HSD17B10
Glutathione Biosynthesis	2Gy 4h	0.60	1.00	NaN	GCLC
Glutathione Redox Reactions I	2Gy 4h	0.49	0.38	NaN	GPX1,GSTP1,PRDX6
Glutathione Redox Reactions II	2Gy 4h	0.60	1.00	NaN	GLRX
Glutathione-mediated Detoxification	2Gy 4h	0.00	0.20	NaN	GSTP1
Glycine Cleavage Complex	2Gy 4h	0.36	0.50	NaN	DLD
Glycogen Degradation II	2Gy 2h	0.85	1.00	NaN	MTAP
Glycogen Degradation III	2Gy 2h	0.44	0.33	NaN	MTAP
Glycogen Degradation III	2Gy 4h	0.24	0.33	NaN	GAA
Glycolysis I	0.05Gy 4h	0.71	0.20	NaN	GPI
**Glycolysis I**	**2Gy 4h**	**3.01**	**1.00**	**2.24**	**ENO1,GAPDH,GPI,PGK1,PKM**
GM-CSF Signaling	0.05Gy 4h	0.00	0.03	NaN	JAK2
**GM-CSF Signaling**	**2Gy 2h**	**1.90**	**0.29**	**-0.33**	**BCL2L1,CAMK2G,CISH,ELK1,PIK3CB,PIK3CD,PPP3CC,PPP3R1,RRAS2,RUNX1,SOS1**
**GM-CSF Signaling**	**2Gy 4h**	**1.82**	**0.42**	**0.00**	**AKT1,BCL2L1,ELK1,ETS1,HRAS,JAK2,KRAS,MAP2K1,PIK3CB,PIK3CD,PPP3CA,PPP3CB,PPP3CC,PPP3R1,RRAS2,RUNX1**

Ingenuity Canonical Pathways	Model	-log(p-value)	Ratio	z-score	Molecules
GNRH Signaling	0.05Gy 2h	0.29	0.01	NaN	EGR1
GNRH Signaling	0.05Gy 4h	1.00	0.08	1.63	EGR1,FOS,GNAI2,GNG5,PRKCD,PRKCI
GNRH Signaling	2Gy 2h	0.42	0.16	0.30	ATF4,CACNA1A,CAMK2G,CDC42,EGR1,ELK1,MAPK7,MAPK8,PRKCE,RAC1,RRAS2,SOS1
**GNRH Signaling**	**2Gy 4h**	**1.60**	**0.36**	**-0.39**	**ADCY8,ATF2,ATF4,CACNA1C,EGFR,EGR1,ELK1,FOS,GNAI2,GNAI3,GNG5,HRAS,ITPR2,KRAS,MAP2K1,MAP3K1,MAP3K7,MAPK8,NFKB1,PRKAR2A,PRKCD,PRKCE,PRKCI,PRKD3,PTK2B,RRAS2,SRC**
GP6 Signaling Pathway	0.05Gy 4h	0.41	0.06	NaN	PRKCD,PRKCI,RHOG
GP6 Signaling Pathway	2Gy 2h	0.00	0.09	-1.34	PDPK1,PIK3CB,PIK3CD,PRKCE,RAC1
GP6 Signaling Pathway	2Gy 4h	0.00	0.19	1.27	AKT1,COL6A2,GSK3B,LAMC2,PIK3CB,PIK3CD,PRKCD,PRKCE,PRKCI,PRKD3
GPCR-Mediated Integration of Enteroendocrine Signaling Exemplified by an L Cell	0.05Gy 4h	0.00	0.03	NaN	GNAI2
GPCR-Mediated Integration of Enteroendocrine Signaling Exemplified by an L Cell	2Gy 4h	0.00	0.16	0.00	ADCY8,ADRB2,GNAI2,GNAI3,ITPR2,PRKAR2A
GPCR-Mediated Nutrient Sensing in Enteroendocrine Cells	0.05Gy 4h	0.90	0.09	0.00	GNAI2,GNG5,PRKCD,PRKCI
GPCR-Mediated Nutrient Sensing in Enteroendocrine Cells	2Gy 2h	0.00	0.04	NaN	CACNA1A,PRKCE
GPCR-Mediated Nutrient Sensing in Enteroendocrine Cells	2Gy 4h	0.00	0.24	-0.30	ADCY8,CACNA1C,GNAI2,GNAI3,GNG5,ITPR2,PRKAR2A,PRKCD,PRKCE,PRKCI,PRKD3
G-Protein Coupled Receptor Signaling	0.05Gy 4h	0.00	0.01	NaN	GNAI2,HRH1
G-Protein Coupled Receptor Signaling	2Gy 2h	0.36	0.15	NaN	ATF4,CAMK2G,CHRM3,DUSP1,DUSP6,GNAO1,IKBKB,IKBKE,NAPEPLD,PDE4D,PDPK1,PIK3CB,PIK3CD,PRKCE,PTGER4,RGS12,RGS4,RRAS2,S1PR3,SOS1
G-Protein Coupled Receptor Signaling	2Gy 4h	0.00	0.22	NaN	ADCY8,ADORA2B,ADRB2,AKT1,APEX1,ATF2,ATF4,DUSP1,DUSP6,GNAI2,GNAI3,HRAS,HRH1,KRAS,LPAR1,MAP2K1,NFKB1,NFKBIA,NPR3,PIK3CB,PIK3CD,PRKAR2A,PRKCE,PTGER2,PTGER3,PTK2B,RGS4,RRAS2,SRC
Graft-versus-Host Disease Signaling	0.05Gy 2h	0.75	0.05	NaN	IL6
Graft-versus-Host Disease Signaling	2Gy 2h	0.00	0.10	NaN	FAS,IL6
Graft-versus-Host Disease Signaling	2Gy 4h	0.00	0.10	NaN	FAS,HLA-E
Granulocyte Adhesion and Diapedesis	0.05Gy 4h	0.00	0.02	NaN	GNAI2,HRH1
Granulocyte Adhesion and Diapedesis	2Gy 2h	0.00	0.10	NaN	CCL2,CCL7,CXCL1,CXCL2,CXCL8,ITGA5,MMP15,SDC4,TNFRSF1B
Granulocyte Adhesion and Diapedesis	2Gy 4h	0.00	0.14	NaN	CD99,CXCL16,EZR,GNAI2,GNAI3,HRH1,ICAM1,IL1R1,MMP11,NGFR,SDC1,TNFRSF11B,TNFRSF1B
Granzyme A Signaling	2Gy 4h	0.00	0.17	NaN	APEX1
Granzyme B Signaling	2Gy 2h	0.92	0.33	NaN	CASP3,CASP8,LMNB1
Granzyme B Signaling	2Gy 4h	0.78	0.44	0.00	BID,CASP3,ENDOG,LMNB1
Growth Hormone Signaling	0.05Gy 2h	0.50	0.03	NaN	SOCS3
**Growth Hormone Signaling**	**0.05Gy 4h**	**1.61**	**0.13**	**0.45**	**FOS,JAK2,PRKCD,PRKCI,SOCS6**
**Growth Hormone Signaling**	**2Gy 2h**	**1.73**	**0.28**	**-1.51**	**ELK1,IRS1,PDPK1,PIK3CB,PIK3CD,PRKCE,SOCS1,SOCS2,SOCS3,SOCS5,SOCS6**
**Growth Hormone Signaling**	**2Gy 4h**	**1.59**	**0.40**	**-0.50**	**ELK1,FOS,GHR,JAK2,PIK3CB,PIK3CD,PRKCD,PRKCE,PRKCI,PRKD3,SOCS2,SOCS3,SOCS5,SOCS6,SOCS7,SRF**
Guanine and Guanosine Salvage I	2Gy 4h	0.60	1.00	NaN	HPRT1
Gustation Pathway	0.05Gy 4h	0.00	0.02	NaN	GNG5
Gustation Pathway	2Gy 2h	0.00	0.08	NaN	CACNA1A,LPAR6,NAPEPLD,PDE4D
Gustation Pathway	2Gy 4h	0.00	0.14	NaN	ADCY8,APEX1,CACNA1C,GNG5,ITPR2,PANX1,PRKAR2A
Hematopoiesis from Multipotent Stem Cells	0.05Gy 4h	0.46	0.10	NaN	KITLG

Ingenuity Canonical Pathways	Model	-log(p-value)	Ratio	z-score	Molecules
Hematopoiesis from Multipotent Stem Cells	2Gy 4h	0.00	0.20	NaN	CSF1,KITLG
**Hematopoiesis from Pluripotent Stem Cells**	**0.05Gy 2h**	**3.34**	**0.18**	**NaN**	**IL11,IL12A,IL6**
Hematopoiesis from Pluripotent Stem Cells	0.05Gy 4h	0.28	0.06	NaN	KITLG
Hematopoiesis from Pluripotent Stem Cells	2Gy 2h	0.36	0.18	NaN	CXCL8,IL11,IL6
Hematopoiesis from Pluripotent Stem Cells	2Gy 4h	0.00	0.24	NaN	CSF1,IL12A,KITLG,LIF
Heme Biosynthesis II	2Gy 2h	0.58	0.50	NaN	ALAS1
Heme Degradation	2Gy 2h	0.58	0.50	NaN	HMOX1
Heme Degradation	2Gy 4h	1.20	1.00	NaN	HMOX1,HMOX2
Heparan Sulfate Biosynthesis	0.05Gy 4h	0.24	0.05	NaN	HS6ST1
Heparan Sulfate Biosynthesis	2Gy 2h	1.29	0.30	0.00	AARS1,CHST11,CHST2,EXT1,HS6ST1,LIPE
Heparan Sulfate Biosynthesis	2Gy 4h	0.42	0.30	0.00	AARS1,CES2,EXT1,HS6ST1,LIPE,PRDX6
Heparan Sulfate Biosynthesis (Late Stages)	0.05Gy 4h	0.25	0.05	NaN	HS6ST1
**Heparan Sulfate Biosynthesis (Late Stages)**	**2Gy 2h**	**1.39**	**0.32**	**0.00**	**AARS1,CHST11,CHST2,EXT1,HS6ST1,LIPE**
Heparan Sulfate Biosynthesis (Late Stages)	2Gy 4h	0.48	0.32	0.00	AARS1,CES2,EXT1,HS6ST1,LIPE,PRDX6
Hepatic Cholestasis	0.05Gy 2h	1.00	0.03	NaN	IL11,IL12A,IL6
Hepatic Cholestasis	0.05Gy 4h	0.00	0.03	NaN	ATP8B1,PRKCD,PRKCI,TGFB1
Hepatic Cholestasis	2Gy 2h	0.00	0.08	NaN	CXCL8,IKBKB,IKBKE,IL11,IL6,MAPK8,PRKCE,RXRA,SLC4A2,TNFRSF1B
Hepatic Cholestasis	2Gy 4h	0.00	0.21	NaN	ADCY8,ATP8B1,CLCF1,CNTF,CYP7B1,IL12A,IL1R1,LIF,MAP3K7,MAPK8,NFKB1,NFKBIA,NGFR,NR1H3,PPARA,PRKAR2A,PRKCD,PRKCE,PRKCI,PRKD3,RARA,TGFB1,TNFRSF11B,TNFRSF1B,TNFSF10
Hepatic Fibrosis / Hepatic Stellate Cell Activation	0.05Gy 2h	0.50	0.02	NaN	IL6,KLF6
Hepatic Fibrosis / Hepatic Stellate Cell Activation	0.05Gy 4h	0.00	0.03	NaN	ACTA2,BCL2,TGFB1,TGFBR1
Hepatic Fibrosis / Hepatic Stellate Cell Activation	2Gy 2h	0.00	0.13	NaN	ACTA2,BAMBI,CCL2,CXCL8,EDN1,FAS,FGF2,IL4R,IL6,IL6R,KLF6,PDGFA,SERPINE1,SMAD3,SMAD7,TNFRSF1B
Hepatic Fibrosis / Hepatic Stellate Cell Activation	2Gy 4h	0.00	0.23	NaN	ACTA2,BCL2,CCN2,COL6A2,CSF1,EGFR,FAS,FGF2,FLT1,ICAM1,IFNGR1,IGFBP4,IL1R1,IL4R,IL6R,LEPR,NFKB1,NGFR,PDGFRA,PGF,SMAD2,SMAD3,SMAD4,SMAD7,TGFB1,TGFBR1,TNFRSF11B,TNFRSF1B
Hepatic Fibrosis Signaling Pathway	0.05Gy 2h	0.00	0.01	NaN	CDKN1B,MYC
Hepatic Fibrosis Signaling Pathway	0.05Gy 4h	0.73	0.06	1.16	ACTA2,BCL2,FOS,FTH1,GNAI2,JAK2,PRKCD,PRKCI,RHOA,RHOG,TGFB1,TGFBR1
**Hepatic Fibrosis Signaling Pathway**	**2Gy 2h**	**1.51**	**0.19**	**-1.58**	**ACTA2,ACVR2A,ATF4,BAMBI,CACNA1A,CASP3,CCL2,CDC42,CDKN1B,CEBPB,CXCL8,EDN1,ELK1,FGF2,FTH1,GLI3,IKBKB,IKBKE,IRS2,ITGA5,MAP2K5,MAPK8,MTOR,MYC,PDGFA,PIK3CB,PIK3CD,PRKCE,RAC1,RHOB,RHOQ,RIPK1,RND3,RRAS2,SERPINE1,SMAD3,SMAD7,SNAI1,SOS1,TNFRSF1B**
**Hepatic Fibrosis Signaling Pathway**	**2Gy 4h**	**2.27**	**0.33**	**-2.29**	**ACTA2,ACVR1,ACVR1B,ACVR2A,AKT1,ATF2,ATF4,AXIN1,BCL2,BMPR2,CACNA1C,CASP3,CCN2,CTNNB1,ELK1,FGF2,FLT1,FOS,FTH1,FZD1,GLI3,GNAI2,GNAI3,GSK3B,HIF1A,HRAS,ICAM1,IL17RA,IL1R1,JAK2,KRAS,LEF1,LEPR,MAP2K1,MAP3K7,MAPK8,NFKB1,NFKBIA,NGFR,NOX4,PDGFRA,PGF,PIK3CB,PIK3CD,PRKAR2A,PRKCD,PRKCE,PRKCI,PRKD3,PTCH1,PTEN,RAC3,RHOA,RIPK1,ROCK1,ROCK2,RRAS2,SMAD2,SMAD3,SMAD4,SMAD7,SP1,TCF4,TCF7,TGFB1,TGFBR1,TNFRSF11B,TNFRSF1B,WNT5A,YAP1**
HER-2 Signaling in Breast Cancer	0.05Gy 2h	0.42	0.02	NaN	CDKN1B
HER-2 Signaling in Breast Cancer	0.05Gy 4h	0.80	0.08	NaN	CDKN1A,MDM2,PRKCD,PRKCI
HER-2 Signaling in Breast Cancer	2Gy 2h	1.08	0.22	NaN	AREG,CCNE1,CDC42,CDKN1A,CDKN1B,MDM2,PIK3CB,PIK3CD,PRKCE,RRAS2,SOS1
HER-2 Signaling in Breast Cancer	2Gy 4h	1.01	0.34	NaN	AKT1,CCNE1,CCNE2,CDKN1A,EGFR,GSK3B,HRAS,ITGB8,KRAS,MDM2,PIK3CB,PIK3CD,PRKCD,PRKCE,PRKCI,PRKD3,RRAS2
Hereditary Breast Cancer Signaling	0.05Gy 4h	0.53	0.07	NaN	CDC25C,CDKN1A,SMARCA4

Ingenuity Canonical Pathways	Model	-log(p-value)	Ratio	z-score	Molecules
Hereditary Breast Cancer Signaling	2Gy 2h	0.76	0.20	NaN	CDKN1A,CHEK1,HDAC6,MSH6,NPM1,PIK3CB,PIK3CD,RRAS2,XPC
**Hereditary Breast Cancer Signaling**	**2Gy 4h**	**2.48**	**0.44**	**NaN**	**AKT1,ATM,BARD1,BRCA2,CCNB1,CDKN1A,DDB2,E2F1,GADD45A,HRAS,KRAS,MSH6,NPM1,PIK3CB,PIK3CD,PTEN,RRAS2,SMARCA4,UBC,XPC**
HGF Signaling	0.05Gy 2h	0.93	0.03	NaN	IL6,PTGS2
HGF Signaling	0.05Gy 4h	0.56	0.06	0.00	CDKN1A,FOS,PRKCD,PRKCI
HGF Signaling	2Gy 2h	1.02	0.21	-0.58	CDC42,CDKN1A,ELK1,IL6,ITGA5,MAPK8,PIK3CB,PIK3CD,PRKCE,PTGS2,RAC1,RRAS2,SOS1
**HGF Signaling**	**2Gy 4h**	**1.30**	**0.35**	**-1.15**	**AKT1,ATF2,CDKN1A,CDKN2A,ELK1,ETS1,ETS2,FOS,HRAS,KRAS,MAP2K1,MAP3K1,MAP3K7,MAPK8,PIK3CB,PIK3CD,PRKCD,PRKCE,PRKCI,PRKD3,PTGS2,RRAS2**
HIF1Î± Signaling	0.05Gy 4h	0.00	0.02	NaN	MDM2
HIF1Î± Signaling	2Gy 2h	0.41	0.16	NaN	EDN1,MAPK7,MAPK8,MDM2,MMP15,PIK3CB,PIK3CD,RRAS2,SLC2A1
HIF1Î± Signaling	2Gy 4h	0.37	0.27	NaN	AKT1,APEX1,HIF1A,HRAS,KRAS,MAPK8,MDM2,MMP11,NAA10,PGF,PIK3CB,PIK3CD,RRAS2,SLC2A1,SLC2A5
HIPPO signaling	2Gy 2h	1.21	0.27	-0.82	FRMD6,PPP2CA,RASSF1,SMAD3,STK3,TEAD4,TP53BP2
**HIPPO signaling**	**2Gy 4h**	**3.43**	**0.58**	**0.00**	**AMOT,CD44,DLG1,LATS1,PPP1CA,PPP2CA,RASSF1,SMAD2,SMAD3,SMAD4,STK4,TEAD1,TEAD4,TP53BP2,YAP1**
**Histidine Degradation III**	**0.05Gy 4h**	**1.37**	**1.00**	**NaN**	**MTHFD2**
Histidine Degradation III	2Gy 2h	0.85	1.00	NaN	MTHFD2
Histidine Degradation VI	2Gy 4h	0.00	0.20	NaN	CYP7B1
**HMGB1 Signaling**	**0.05Gy 2h**	**1.39**	**0.04**	**NaN**	**IL11,IL12A,IL6**
HMGB1 Signaling	0.05Gy 4h	0.33	0.05	2.00	FOS,RHOA,RHOG,TGFB1
HMGB1 Signaling	2Gy 2h	1.18	0.21	0.00	CCL2,CDC42,CXCL8,ELK1,IL11,IL6,MAP2K5,MAPK8,PIK3CB,PIK3CD,RAC1,RHOB,RHOQ,RND3,RRAS2,SERPINE1,TNFRSF1B
HMGB1 Signaling	2Gy 4h	1.13	0.33	0.00	AKT1,CLCF1,CNTF,ELK1,FOS,HRAS,ICAM1,IFNGR1,IL12A,IL1R1,KRAS,LIF,MAP2K1,MAPK8,NFKB1,NGFR,PIK3CB,PIK3CD,RAC3,RBBP7,RHOA,RRAS2,SP1,TGFB1,TNFRSF11B,TNFRSF1B,TNFSF10
HOTAIR Regulatory Pathway	0.05Gy 2h	0.67	0.02	NaN	MYC,SNAI2
HOTAIR Regulatory Pathway	0.05Gy 4h	0.71	0.06	-0.82	CDKN1A,HSF1,JARID2,MDM2,REST,TGFB1
HOTAIR Regulatory Pathway	2Gy 2h	0.00	0.09	-0.71	CDKN1A,EED,MDM2,MMP15,MYC,PIK3CB,PIK3CD,SNAI2
**HOTAIR Regulatory Pathway**	**2Gy 4h**	**1.37**	**0.33**	**-1.62**	**AKT1,CD44,CDKN1A,CTNNB1,EED,HSF1,ICAM1,JARID2,KDM1A,KMT2A,LEF1,MDM2,mir-29,MMP11,MRTFA,NFKB1,NFKBIA,PIK3CB,PIK3CD,PTEN,RBBP7,REST,ROCK1,ROCK2,SNAI2,SRF,TCF4,TCF7,TGFB1,WNT5A,XIAP**
Human Embryonic Stem Cell Pluripotency	0.05Gy 4h	0.00	0.02	NaN	TGFB1,TGFBR1
Human Embryonic Stem Cell Pluripotency	2Gy 2h	0.00	0.12	NaN	BDNF,FGF2,INHBA,PDGFA,PDPK1,PIK3CB,PIK3CD,S1PR3,SMAD3,SMAD7
Human Embryonic Stem Cell Pluripotency	2Gy 4h	0.94	0.31	NaN	ACVR1,AKT1,AXIN1,BMP1,BMP2,BMP4,BMPR2,CTNNB1,FGF2,FZD1,GSK3B,INHBA,LEF1,PDGFRA,PIK3CB,PIK3CD,SMAD2,SMAD3,SMAD4,SMAD6,SMAD7,TCF4,TCF7,TGFB1,TGFBR1,WNT5A
Huntington's Disease Signaling	0.05Gy 4h	0.31	0.05	1.00	GNG5,PRKCD,PRKCI,REST
Huntington's Disease Signaling	2Gy 2h	1.06	0.20	-2.31	ATF4,BCL2L1,BDNF,CASP3,CASP8,HDAC6,HSPA9,MAPK8,MTOR,NCOR2,PDPK1,PIK3CB,PIK3CD,PRKCE,SGK1,SOS1,STX16
Huntington's Disease Signaling	2Gy 4h	0.52	0.28	1.50	AKT1,ATF2,ATF4,ATP5F1A,ATP5PF,BCL2L1,CASP3,CDK5R1,CTSD,EGFR,GNG5,HRAS,HSPA6,HSPA8,MAPK8,PIK3CB,PIK3CD,PRKCD,PRKCE,PRKCI,PRKD3,REST,SP1,UBC
Hypoxia Signaling in the Cardiovascular System	0.05Gy 4h	0.46	0.07	NaN	MDM2,UBE2I
Hypoxia Signaling in the Cardiovascular System	2Gy 2h	0.00	0.14	NaN	ATF4,EDN1,MDM2,UBE2E1
Hypoxia Signaling in the Cardiovascular System	2Gy 4h	1.07	0.38	0.71	AKT1,ATF2,ATF4,ATM,HIF1A,HSP90AB1,MDM2,NFKBIA,P4HB,PTEN,UBE2I
Hypusine Biosynthesis	2Gy 4h	0.60	1.00	NaN	EIF5A

Ingenuity Canonical Pathways	Model	-log(p-value)	Ratio	z-score	Molecules
Î±-Adrenergic Signaling	0.05Gy 4h	1.07	0.10	NaN	GNAI2,GNG5,PRKCD,PRKCI
Î±-Adrenergic Signaling	2Gy 2h	0.00	0.05	NaN	PRKCE,RRAS2
Î±-Adrenergic Signaling	2Gy 4h	0.99	0.35	-0.30	ADCY8,GNAI2,GNAI3,GNG5,HRAS,ITPR2,KRAS,MAP2K1,PRKAR2A,PRKCD,PRKCE,PRKCI,PRKD3,RRAS2
Î³-glutamyl Cycle	2Gy 4h	0.24	0.33	NaN	GCLC
Î³-linolenate Biosynthesis II (Animals)	2Gy 4h	0.00	0.17	NaN	FADS1
iCOS-iCOSL Signaling in T Helper Cells	0.05Gy 4h	0.00	0.02	NaN	NFATC1
iCOS-iCOSL Signaling in T Helper Cells	2Gy 2h	1.03	0.22	-2.11	CAMK2G,IKBKB,IKBKE,NFATC1,NFATC4,PDPK1,PIK3CB,PIK3CD,PPP3CC,PPP3R1,RAC1
iCOS-iCOSL Signaling in T Helper Cells	2Gy 4h	0.28	0.26	-1.51	AKT1,ITPR2,NFAT5,NFATC1,NFKB1,NFKBIA,PIK3CB,PIK3CD,PPP3CA,PPP3CB,PPP3CC,PPP3R1,PTEN
IGF-1 Signaling	0.05Gy 2h	0.38	0.02	NaN	SOCS3
IGF-1 Signaling	0.05Gy 4h	0.68	0.07	0.00	FOS,JAK2,PRKCI,SOCS6
**IGF-1 Signaling**	**2Gy 2h**	**2.07**	**0.27**	**-1.07**	**CCN1,ELK1,IRS1,IRS2,MAPK8,PDPK1,PIK3CB,PIK3CD,RRAS2,SOCS1,SOCS2,SOCS3,SOCS5,SOCS6,SOS1**
**IGF-1 Signaling**	**2Gy 4h**	**3.01**	**0.45**	**-0.45**	**AKT1,CCN1,CCN2,CSNK2B,ELK1,FOS,FOXO3,HRAS,IGFBP4,IGFBP6,JAK2,KRAS,MAP2K1,MAPK8,PIK3CB,PIK3CD,PRKAR2A,PRKCI,RRAS2,SOCS2,SOCS3,SOCS5,SOCS6,SOCS7,SRF**
IL-1 Signaling	0.05Gy 4h	0.67	0.08	NaN	FOS,GNAI2,GNG5
IL-1 Signaling	2Gy 2h	0.00	0.11	NaN	GNAO1,IKBKB,IKBKE,MAPK8
IL-1 Signaling	2Gy 4h	0.65	0.32	-1.41	ADCY8,FOS,GNAI2,GNAI3,GNG5,IL1R1,MAP3K1,MAP3K7,MAPK8,NFKB1,NFKBIA,PRKAR2A
IL-10 Signaling	0.05Gy 2h	1.27	0.05	NaN	IL6,SOCS3
IL-10 Signaling	0.05Gy 4h	0.00	0.03	NaN	FOS
IL-10 Signaling	2Gy 2h	1.00	0.23	NaN	ELK1,HMOX1,IKBKB,IKBKE,IL4R,IL6,MAPK8,SOCS3,TYK2
IL-10 Signaling	2Gy 4h	0.54	0.30	NaN	ELK1,FOS,HMOX1,IL1R1,IL4R,MAP3K7,MAP4K4,MAPK8,NFKB1,NFKBIA,SOCS3,SP1
IL-12 Signaling and Production in Macrophages	0.05Gy 2h	0.30	0.01	NaN	IL12A
IL-12 Signaling and Production in Macrophages	0.05Gy 4h	0.42	0.05	NaN	FOS,PRKCD,PRKCI,TGFB1
IL-12 Signaling and Production in Macrophages	2Gy 2h	0.00	0.12	NaN	APOE,CEBPB,IKBKB,IKBKE,MAPK8,PIK3CB,PIK3CD,PRKCE,RXRA
IL-12 Signaling and Production in Macrophages	2Gy 4h	0.00	0.19	NaN	AKT1,FOS,IFNGR1,IL12A,MAP2K1,MAPK8,NFKB1,PIK3CB,PIK3CD,PRKCD,PRKCE,PRKCI,PRKD3,TGFB1
IL-15 Production	0.05Gy 2h	0.38	0.02	NaN	IL6
IL-15 Production	0.05Gy 4h	0.66	0.07	NaN	JAK2,KIT,PRKCI,YES1
IL-15 Production	2Gy 2h	0.00	0.14	NaN	EPHA2,EPHB3,EPHB4,IL6,MAP2K5,MERTK,TWF1,TYK2
IL-15 Production	2Gy 4h	0.46	0.28	NaN	ABL1,DDR1,EGFR,EPHA2,EPHB4,FLT1,JAK2,KIT,MAP2K1,MERTK,NFKB1,PDGFRA,PRKCI,PTK2B,SRC,YES1
IL-15 Signaling	0.05Gy 2h	0.53	0.03	NaN	IL6
IL-15 Signaling	0.05Gy 4h	0.00	0.03	NaN	JAK2
IL-15 Signaling	2Gy 2h	0.59	0.19	NaN	BCL2L1,CXCL8,IL6,PIK3CB,PIK3CD,RRAS2,TYK2
IL-15 Signaling	2Gy 4h	0.51	0.30	NaN	AKT1,BCL2L1,HRAS,JAK2,KRAS,MAP2K1,NFKB1,PIK3CB,PIK3CD,RAC3,RRAS2
IL-17 Signaling	0.05Gy 2h	1.18	0.04	NaN	IL6,PTGS2
IL-17 Signaling	0.05Gy 4h	0.00	0.02	NaN	JAK2
**IL-17 Signaling**	**2Gy 2h**	**1.74**	**0.27**	**NaN**	**CCL2,CEBPB,CXCL1,CXCL8,ELK1,IL19,IL6,MAPK8,PIK3CB,PIK3CD,PTGS2,RRAS2**
IL-17 Signaling	2Gy 4h	1.13	0.36	NaN	AKT1,ATF2,ELK1,GSK3B,HRAS,IL17RA,JAK2,KRAS,MAP2K1,MAP3K7,MAPK8,NFKB1,PIK3CB,PIK3CD,PTGS2,RRAS2
IL-17A Signaling in Airway Cells	0.05Gy 2h	0.52	0.03	NaN	IL6
IL-17A Signaling in Airway Cells	0.05Gy 4h	0.00	0.03	NaN	JAK2
IL-17A Signaling in Airway Cells	2Gy 2h	1.12	0.24	-1.41	CXCL1,IKBKB,IKBKE,IL19,IL6,MAPK8,PIK3CB,PIK3CD,TYK2

Ingenuity Canonical Pathways	Model	-log(p-value)	Ratio	z-score	Molecules
IL-17A Signaling in Airway Cells	2Gy 4h	0.65	0.32	-1.51	AKT1,GSK3B,IL17RA,JAK2,MAP2K1,MAP3K7,MAPK8,NFKB1,NFKBIA,PIK3CB,PIK3CD,PTEN
**IL-17A Signaling in Fibroblasts**	**0.05Gy 2h**	**1.84**	**0.10**	**NaN**	**IL6,NFKBIZ**
IL-17A Signaling in Fibroblasts	0.05Gy 4h	0.24	0.05	NaN	FOS
**IL-17A Signaling in Fibroblasts**	**2Gy 2h**	**2.42**	**0.40**	**NaN**	**CCL2,CCL7,CEBPB,CEBPD,IKBKB,IKBKE,IL6,NFKBIZ**
IL-17A Signaling in Fibroblasts	2Gy 4h	0.67	0.35	NaN	CEBPD,FOS,GSK3B,IL17RA,MAP3K7,NFKB1,NFKBIA
IL-17A Signaling in Gastric Cells	0.05Gy 4h	0.32	0.07	NaN	FOS
IL-17A Signaling in Gastric Cells	2Gy 2h	0.45	0.20	NaN	CXCL1,CXCL8,MAPK8
IL-17A Signaling in Gastric Cells	2Gy 4h	0.50	0.33	-1.00	EGFR,FOS,IL17RA,MAPK8,NFKB1
IL-2 Signaling	0.05Gy 4h	0.00	0.03	NaN	FOS
IL-2 Signaling	2Gy 2h	0.76	0.21	-0.82	ELK1,MAPK8,PIK3CB,PIK3CD,RRAS2,SOCS1,SOS1
IL-2 Signaling	2Gy 4h	1.01	0.36	0.00	AKT1,CSNK2B,ELK1,FOS,HRAS,KRAS,MAP2K1,MAPK8,PIK3CB,PIK3CD,PTK2B,RRAS2
IL-22 Signaling	0.05Gy 2h	0.97	0.08	NaN	SOCS3
IL-22 Signaling	2Gy 2h	0.63	0.25	NaN	MAPK8,SOCS3,TYK2
IL-22 Signaling	2Gy 4h	0.45	0.33	0.00	AKT1,IL22RA1,MAPK8,SOCS3
IL-23 Signaling Pathway	0.05Gy 2h	0.63	0.04	NaN	SOCS3
IL-23 Signaling Pathway	0.05Gy 4h	0.00	0.04	NaN	JAK2
IL-23 Signaling Pathway	2Gy 2h	0.45	0.18	-1.34	PIK3CB,PIK3CD,RUNX1,SOCS3,TYK2
IL-23 Signaling Pathway	2Gy 4h	0.86	0.36	-1.90	AKT1,HIF1A,JAK2,NFKB1,NFKBIA,PIK3CB,PIK3CD,RORA,RUNX1,SOCS3
IL-3 Signaling	0.05Gy 4h	0.95	0.09	0.00	FOS,JAK2,PRKCD,PRKCI
IL-3 Signaling	2Gy 2h	0.81	0.21	-0.33	ELK1,PIK3CB,PIK3CD,PPP3CC,PPP3R1,PRKCE,RAC1,RRAS2,SOS1
**IL-3 Signaling**	**2Gy 4h**	**1.84**	**0.41**	**-0.94**	**AKT1,ELK1,FOS,HRAS,JAK2,KRAS,MAP2K1,PIK3CB,PIK3CD,PPP3CA,PPP3CB,PPP3CC,PPP3R1,PRKCD,PRKCE,PRKCI,PRKD3,RRAS2**
IL-4 Signaling	0.05Gy 4h	0.67	0.08	NaN	JAK2,NFATC1,NR3C1
**IL-4 Signaling**	**2Gy 2h**	**2.36**	**0.32**	**NaN**	**IL4R,IRS1,MTOR,NFATC1,NFATC4,NR3C2,PIK3CB,PIK3CD,RRAS2,SOCS1,SOS1,TYK2**
IL-4 Signaling	2Gy 4h	0.46	0.29	NaN	AKT1,HRAS,IL4R,JAK2,KRAS,NFAT5,NFATC1,NR3C1,PIK3CB,PIK3CD,RRAS2
IL-6 Signaling	0.05Gy 2h	0.87	0.03	NaN	IL6,SOCS3
IL-6 Signaling	0.05Gy 4h	0.00	0.03	NaN	FOS,JAK2
IL-6 Signaling	2Gy 2h	1.28	0.22	-0.26	CEBPB,CXCL8,ELK1,IKBKB,IKBKE,IL6,IL6R,MAPK8,PIK3CB,PIK3CD,RRAS2,SOCS1,SOCS3,SOS1,TNFRSF1B
**IL-6 Signaling**	**2Gy 4h**	**1.36**	**0.35**	**-1.63**	**AKT1,CSNK2B,ELK1,FOS,HRAS,IL1R1,IL6R,IL6ST,JAK2,KRAS,MAP2K1,MAP3K7,MAP4K4,MAPK8,NFKB1,NFKBIA,NGFR,PIK3CB,PIK3CD,RRAS2,SOCS3,SRF,TNFRSF11B,TNFRSF1B**
IL-7 Signaling Pathway	0.05Gy 2h	1.24	0.05	NaN	CDKN1B,MYC
IL-7 Signaling Pathway	0.05Gy 4h	0.27	0.05	NaN	BCL2,NFATC1
IL-7 Signaling Pathway	2Gy 2h	1.21	0.24	-2.33	BCL6,CDKN1B,MYC,NFATC1,PDPK1,PIK3CB,PIK3CD,SLC2A1,SOCS1,SOS1
IL-7 Signaling Pathway	2Gy 4h	0.00	0.21	0.38	AKT1,BCL2,BCL6,FOXO3,GSK3B,NFATC1,PIK3CB,PIK3CD,SLC2A1
IL-8 Signaling	0.05Gy 2h	0.20	0.01	NaN	PTGS2
IL-8 Signaling	0.05Gy 4h	1.16	0.08	1.41	BCL2,FOS,GNAI2,GNG5,PRKCD,PRKCI,RHOA,RHOG
IL-8 Signaling	2Gy 2h	1.29	0.20	-1.79	BCL2L1,CDC42,CXCL1,CXCL8,HMOX1,IKBKB,IKBKE,LIMK2,MAPK8,MTOR,NAPEPLD,PIK3CB,PIK3CD,PLD1,PRKCE,PTGS2,RAC1,RHOB,RHOQ,RND3,RRAS2
**IL-8 Signaling**	**2Gy 4h**	**1.77**	**0.35**	**0.17**	**AKT1,BCL2,BCL2L1,CSTB,EGFR,FLT1,FOS,GNAI2,GNAI3,GNG5,HMOX1,HRAS,ICAM1,KRAS,LIMK2,MAP2K1,MAP4K4,MAPK8,NFKB1,NOX4,PGF,PIK3CB,PIK3CD,PLD1,PRKCD,PRKCE,PRKCI,PRKD3,PTGS2,PTK2B,RAC3,RHOA,ROCK1,ROCK2,RRAS2,SRC**
**IL-9 Signaling**	**0.05Gy 2h**	**1.88**	**0.11**	**NaN**	**BCL3,SOCS3**
**IL-9 Signaling**	**2Gy 2h**	**2.58**	**0.42**	**-1.63**	**BCL3,CISH,IRS1,IRS2,PIK3CB,PIK3CD,SOCS2,SOCS3**
IL-9 Signaling	2Gy 4h	0.27	0.26	-1.00	NFKB1,PIK3CB,PIK3CD,SOCS2,SOCS3

Ingenuity Canonical Pathways	Model	-log(p-value)	Ratio	z-score	Molecules
**ILK Signaling**	**0.05Gy 2h**	**1.36**	**0.04**	**NaN**	**MYC,PTGS2,SNAI2**
ILK Signaling	0.05Gy 4h	0.54	0.06	1.34	ACTA2,FERMT2,FOS,RHOA,RHOG
**ILK Signaling**	**2Gy 2h**	**2.60**	**0.26**	**-1.96**	**ACTA2,ATF4,CASP3,CDC42,FERMT2,IRS1,IRS2,MAPK8,MTOR,MYC,PDPK1,PIK3CB,PIK3CD,PPP2CA,PTGS2,RAC1,RHOB,RHOQ,RND3,SNAI1,SNAI2,TGFB1I1**
ILK Signaling	2Gy 4h	1.02	0.32	0.82	ACTA2,AKT1,ATF2,ATF4,BMP2,CASP3,CFL1,CTNNB1,FERMT2,FOS,GSK3B,HIF1A,ITGB8,KRT18,LEF1,MAPK8,NFKB1,PGF,PIK3CB,PIK3CD,PPP2CA,PTEN,PTGS2,RAC3,RHOA,SNAI2,TMSB10/TMSB4X
Induction of Apoptosis by HIV1	0.05Gy 2h	0.61	0.03	NaN	BBC3
Induction of Apoptosis by HIV1	0.05Gy 4h	0.44	0.07	NaN	BBC3,BCL2
**Induction of Apoptosis by HIV1**	**2Gy 2h**	**2.79**	**0.37**	**0.91**	**BBC3,BCL2L1,BIRC2,CASP3,CASP8,FAS,IKBKB,IKBKE,MAPK8,RIPK1,TNFRSF1B**
**Induction of Apoptosis by HIV1**	**2Gy 4h**	**2.58**	**0.50**	**0.26**	**BBC3,BCL2,BCL2L1,BID,CASP3,FAS,MAPK8,NFKB1,NFKBIA,NGFR,RIPK1,SLC25A5,TNFRSF11B,TNFRSF1B,XIAP**
Inflammasome pathway	2Gy 2h	0.00	0.08	NaN	CASP8
Inflammasome pathway	2Gy 4h	0.00	0.23	NaN	NFKB1,PANX1,PYCARD
Inhibition of Angiogenesis by TSP1	0.05Gy 4h	0.57	0.08	NaN	TGFB1,TGFBR1
Inhibition of Angiogenesis by TSP1	2Gy 2h	0.00	0.08	NaN	CASP3,MAPK8
Inhibition of Angiogenesis by TSP1	2Gy 4h	0.00	0.25	-0.45	AKT1,CASP3,MAPK8,SDC1,TGFB1,TGFBR1
Inhibition of ARE-Mediated mRNA Degradation Pathway	2Gy 2h	0.00	0.13	1.00	MAPK7,PPP2CA,TNFRSF1B,ZFP36
Inhibition of ARE-Mediated mRNA Degradation Pathway	2Gy 4h	0.58	0.31	0.00	AKT1,CNOT7,MAP3K7,NGFR,PPP2CA,PRKAR2A,TNFRSF11B,TNFRSF1B,TNFSF10,ZFP36
Inhibition of Matrix Metalloproteases	2Gy 2h	0.00	0.04	NaN	MMP15
Inhibition of Matrix Metalloproteases	2Gy 4h	0.00	0.11	NaN	ADAM17,MMP11,SDC1
iNOS Signaling	0.05Gy 4h	0.66	0.10	NaN	FOS,JAK2
iNOS Signaling	2Gy 2h	0.23	0.14	NaN	IKBKB,IKBKE,TYK2
iNOS Signaling	2Gy 4h	0.00	0.24	-0.45	FOS,IFNGR1,JAK2,NFKB1,NFKBIA
Insulin Receptor Signaling	0.05Gy 2h	0.36	0.02	NaN	SOCS3
Insulin Receptor Signaling	0.05Gy 4h	0.00	0.03	NaN	JAK2,PRKCI
**Insulin Receptor Signaling**	**2Gy 2h**	**1.46**	**0.23**	**-1.60**	**IRS1,IRS2,LIPE,MAPK8,MTOR,NCK1,PDPK1,PIK3CB,PIK3CD,RHOQ,RRAS2,SGK1,SOCS3,SOS1**
Insulin Receptor Signaling	2Gy 4h	0.83	0.32	0.00	AKT1,CRK,FOXO3,GSK3B,HRAS,JAK2,KRAS,LIPE,MAP2K1,MAPK8,PIK3CB,PIK3CD,PPP1CA,PRKAR2A,PRKCI,PTEN,RRAS2,SOCS3,STXBP4
Integrin Signaling	0.05Gy 4h	0.62	0.06	1.34	ACTA2,ARF3,ARF6,RHOA,RHOG
Integrin Signaling	2Gy 2h	1.10	0.20	-1.29	ACTA2,ARF4,ARF6,CDC42,ITGA5,MAPK8,NCK1,NEDD9,PIK3CB,PIK3CD,RAC1,RHOB,RHOQ,RND3,RRAS2,SOS1
Integrin Signaling	2Gy 4h	0.96	0.32	-0.60	ABL1,ACTA2,AKT1,ARF1,ARF3,ARF4,ARF5,ARF6,BCAR1,CRK,GSK3B,HRAS,ITGB8,KRAS,MAP2K1,MAPK8,PFN1,PIK3CB,PIK3CD,PTEN,RAC3,RHOA,ROCK1,RRAS2,SRC
**Interferon Signaling**	**0.05Gy 4h**	**1.91**	**0.18**	**1.00**	**BCL2,IFITM1,JAK2,TAP1**
Interferon Signaling	2Gy 2h	0.00	0.14	NaN	PTPN2,SOCS1,TYK2
Interferon Signaling	2Gy 4h	0.79	0.36	1.41	BCL2,IFIT1,IFITM1,IFITM3,IFNGR1,JAK2,MX1,TAP1
Intrinsic Prothrombin Activation Pathway	2Gy 4h	0.00	0.06	NaN	THBD
Iron homeostasis signaling pathway	0.05Gy 2h	0.39	0.02	NaN	IL6
Iron homeostasis signaling pathway	0.05Gy 4h	0.39	0.05	NaN	ATP6V1B2,FTH1,JAK2
Iron homeostasis signaling pathway	2Gy 2h	0.62	0.18	NaN	ATP6V0A1,FTH1,HMOX1,HSPA9,IL6,IL6R,PDGFA,SLC46A1,SMAD3,TYK2
**Iron homeostasis signaling pathway**	**2Gy 4h**	**1.40**	**0.36**	**NaN**	**ATP6V0A1,ATP6V1B2,ATP6V1E1,BMP1,BMP2,BMP4,BMPR2,EGFR,FTH1,HIF1A,HMOX1,HMOX2,IL6R,ISCU,JAK2,PDGFRA,SLC11A2,SMAD2,SMAD3,SMAD4**
Isoleucine Degradation I	2Gy 4h	1.20	1.00	NaN	DLD,HSD17B10

Ingenuity Canonical Pathways	Model	-log(p-value)	Ratio	z-score	Molecules
JAK/Stat Signaling	0.05Gy 2h	1.29	0.05	NaN	IL6,SOCS3
JAK/Stat Signaling	0.05Gy 4h	1.10	0.10	0.00	CDKN1A,FOS,JAK2,SOCS6
**JAK/Stat Signaling**	**2Gy 2h**	**4.53**	**0.41**	**-1.00**	**BCL2L1,CDKN1A,CEBPB,CISH,IL6,MTOR,PIK3CB,PIK3CD,RRAS2,SOCS1,SOCS2,SOCS3,SOCS5,SOCS6,SOS1,TYK2**
**JAK/Stat Signaling**	**2Gy 4h**	**2.08**	**0.44**	**0.24**	**AKT1,BCL2L1,CDKN1A,FOS,HRAS,JAK2,KRAS,MAP2K1,NFKB1,PIK3CB,PIK3CD,RRAS2,SOCS2,SOCS3,SOCS5,SOCS6,SOCS7**
Ketogenesis	2Gy 2h	0.58	0.50	NaN	HMGCS1
Ketogenesis	2Gy 4h	0.36	0.50	NaN	HMGCS1
Leptin Signaling in Obesity	0.05Gy 2h	0.52	0.03	NaN	SOCS3
Leptin Signaling in Obesity	0.05Gy 4h	0.00	0.03	NaN	JAK2
Leptin Signaling in Obesity	2Gy 2h	0.00	0.08	NaN	PIK3CB,PIK3CD,SOCS3
Leptin Signaling in Obesity	2Gy 4h	0.00	0.24	-0.38	ADCY8,AKT1,JAK2,LEPR,MAP2K1,PIK3CB,PIK3CD,PRKAR2A,SOCS3
Leukocyte Extravasation Signaling	0.05Gy 4h	0.72	0.07	1.63	ACTA2,GNAI2,PRKCD,PRKCI,RHOA,VAV2
Leukocyte Extravasation Signaling	2Gy 2h	0.00	0.10	-0.33	ACTA2,CDC42,ITGA5,MAPK8,MMP15,PIK3CB,PIK3CD,PRKCE,RAC1
Leukocyte Extravasation Signaling	2Gy 4h	0.45	0.27	-0.82	ABL1,ACTA2,BCAR1,CD44,CD99,CRK,CTNNB1,EZR,GNAI2,GNAI3,ICAM1,MAPK8,MMP11,PIK3CB,PIK3CD,PRKCD,PRKCE,PRKCI,PRKD3,PTK2B,RHOA,ROCK1,ROCK2,SRC,VAV2
Lipid Antigen Presentation by CD1	0.05Gy 4h	0.64	0.17	NaN	ARF6
Lipid Antigen Presentation by CD1	2Gy 2h	0.22	0.17	NaN	ARF6
Lipid Antigen Presentation by CD1	2Gy 4h	0.33	0.33	NaN	ARF6,CALR
LPS/IL-1 Mediated Inhibition of RXR Function	0.05Gy 4h	0.00	0.03	NaN	HS6ST1,NR1H2,PPARGC1B
LPS/IL-1 Mediated Inhibition of RXR Function	2Gy 2h	0.00	0.10	1.63	ALAS1,APOE,CHST11,CHST2,HMGCS1,HS6ST1,MAPK8,PPARGC1A,PPARGC1B,RXRA,TNFRSF1B
LPS/IL-1 Mediated Inhibition of RXR Function	2Gy 4h	0.00	0.18	-1.07	ALDH18A1,CES2,CPT1A,CPT2,GSTP1,HMGCS1,HS6ST1,IL1R1,MAP3K1,MAP3K7,MAPK8,NGFR,NR1H2,NR1H3,PPARA,PPARGC1B,RARA,SOD3,TNFRSF11B,TNFRSF1B
LPS-stimulated MAPK Signaling	0.05Gy 4h	0.60	0.07	NaN	FOS,PRKCD,PRKCI
LPS-stimulated MAPK Signaling	2Gy 2h	1.28	0.24	0.00	CDC42,ELK1,IKBKB,IKBKE,MAPK8,PIK3CB,PIK3CD,PRKCE,RAC1,RRAS2
**LPS-stimulated MAPK Signaling**	**2Gy 4h**	**2.21**	**0.44**	**-0.47**	**ATF2,ELK1,FOS,HRAS,KRAS,MAP2K1,MAP3K7,MAPK8,NFKB1,NFKBIA,PIK3CB,PIK3CD,PRKCD,PRKCE,PRKCI,PRKD3,RRAS2,SRF**
LXR/RXR Activation	0.05Gy 2h	0.76	0.03	NaN	IL6,PTGS2
LXR/RXR Activation	0.05Gy 4h	0.00	0.03	NaN	NR1H2,RXRB
LXR/RXR Activation	2Gy 2h	0.00	0.13	-0.71	APOE,CCL2,CCL7,HMGCR,IL6,NCOR2,PTGS2,RXRA,RXRB,TNFRSF1B
LXR/RXR Activation	2Gy 4h	0.00	0.14	1.27	HMGCR,IL1R1,LDLR,NFKB1,NGFR,NR1H2,NR1H3,PTGS2,RXRB,TNFRSF11B,TNFRSF1B
**Lymphotoxin Î² Receptor Signaling**	**2Gy 2h**	**2.75**	**0.39**	**-1.89**	**BCL2L1,BIRC2,CASP3,CXCL1,IKBKB,IKBKE,PDPK1,PIK3CB,PIK3CD,RELB**
Lymphotoxin Î² Receptor Signaling	2Gy 4h	0.50	0.31	-1.13	AKT1,BCL2L1,CASP3,NFKB1,NFKBIA,PIK3CB,PIK3CD,TRAF3
Macropinocytosis Signaling	0.05Gy 4h	0.92	0.09	NaN	ARF6,PRKCD,PRKCI,RHOA
Macropinocytosis Signaling	2Gy 2h	0.76	0.20	0.00	ARF6,CDC42,ITGA5,PDGFA,PIK3CB,PIK3CD,PRKCE,RAC1,RRAS2
Macropinocytosis Signaling	2Gy 4h	0.67	0.31	-0.58	ARF6,CSF1,HRAS,ITGB8,KRAS,PIK3CB,PIK3CD,PRKCD,PRKCE,PRKCI,PRKD3,RHOA,RRAS2,SRC
Maturity Onset Diabetes of Young (MODY) Signaling	2Gy 2h	0.00	0.08	NaN	CACNA1A
Maturity Onset Diabetes of Young (MODY) Signaling	2Gy 4h	0.00	0.15	NaN	CACNA1C,GAPDH
**Mechanisms of Viral Exit from Host Cells**	**0.05Gy 4h**	**1.72**	**0.21**	**NaN**	**ACTA2,PRKCD,PRKCI**
Mechanisms of Viral Exit from Host Cells	2Gy 2h	0.50	0.21	NaN	ACTA2,LMNB1,PRKCE
Mechanisms of Viral Exit from Host Cells	2Gy 4h	0.95	0.43	NaN	ACTA2,LMNB1,PRKCD,PRKCE,PRKCI,PRKD3
Melanocyte Development and Pigmentation Signaling	0.05Gy 4h	0.47	0.06	NaN	BCL2,KIT,KITLG
Melanocyte Development and Pigmentation Signaling	2Gy 2h	0.26	0.14	-1.63	ATF4,MITF,PIK3CB,PIK3CD,RRAS2,SH2B2,SOS1

Ingenuity Canonical Pathways	Model	-log(p-value)	Ratio	z-score	Molecules
Melanocyte Development and Pigmentation Signaling	2Gy 4h	0.85	0.33	-0.78	ADCY8,ATF2,ATF4,BCL2,CRK,HRAS,KIT,KITLG,KRAS,MAP2K1,PIK3CB,PIK3CD,PRKAR2A,RRAS2,SH2B2,SRC
Melanoma Signaling	0.05Gy 4h	0.50	0.07	NaN	CDKN1A,MDM2
Melanoma Signaling	2Gy 2h	0.77	0.22	-0.45	CDKN1A,MDM2,MITF,PIK3CB,PIK3CD,RRAS2
**Melanoma Signaling**	**2Gy 4h**	**1.67**	**0.44**	**0.00**	**AKT1,CDKN1A,CDKN2A,E2F1,HRAS,KRAS,MAP2K1,MDM2,PIK3CB,PIK3CD,PTEN,RRAS2**
Melatonin Degradation I	2Gy 4h	0.00	0.05	NaN	CYP1B1
Melatonin Signaling	0.05Gy 4h	0.77	0.09	NaN	GNAI2,PRKCD,PRKCI
Melatonin Signaling	2Gy 2h	0.00	0.12	-1.00	CAMK2G,GNAO1,MAP2K5,PRKCE
Melatonin Signaling	2Gy 4h	0.31	0.27	-0.33	GNAI2,GNAI3,MAP2K1,PRKAR2A,PRKCD,PRKCE,PRKCI,PRKD3,RORA
Mevalonate Pathway I	2Gy 2h	1.27	0.67	NaN	HMGCR,HMGCS1
Mevalonate Pathway I	2Gy 4h	0.80	0.67	NaN	HMGCR,HMGCS1
MIF Regulation of Innate Immunity	0.05Gy 2h	0.80	0.06	NaN	PTGS2
MIF Regulation of Innate Immunity	0.05Gy 4h	0.27	0.06	NaN	FOS
MIF Regulation of Innate Immunity	2Gy 2h	0.00	0.11	NaN	MAPK8,PTGS2
MIF Regulation of Innate Immunity	2Gy 4h	0.32	0.28	0.45	FOS,MAPK8,NFKB1,NFKBIA,PTGS2
MIF-mediated Glucocorticoid Regulation	0.05Gy 2h	0.94	0.08	NaN	PTGS2
MIF-mediated Glucocorticoid Regulation	0.05Gy 4h	0.37	0.08	NaN	NR3C1
MIF-mediated Glucocorticoid Regulation	2Gy 2h	0.00	0.08	NaN	PTGS2
MIF-mediated Glucocorticoid Regulation	2Gy 4h	0.38	0.31	1.00	NFKB1,NFKBIA,NR3C1,PTGS2
Mismatch Repair in Eukaryotes	2Gy 2h	1.27	0.67	NaN	MSH6,PCNA
Mismatch Repair in Eukaryotes	2Gy 4h	0.80	0.67	NaN	MSH6,PCNA
Mitochondrial Dysfunction	0.05Gy 4h	0.60	0.07	NaN	BCL2,NDUFS8,NDUFV3
Mitochondrial Dysfunction	2Gy 2h	0.00	0.10	NaN	CASP3,CASP8,MAPK8,PRDX3
**Mitochondrial Dysfunction**	**2Gy 4h**	**1.48**	**0.39**	**NaN**	**AIFM1,ATP5F1A,ATP5PF,BCL2,CASP3,CPT1A,HSD17B10,MAPK8,NDUFS1,NDUFS3,NDUFS8,NDUFV1,NDUFV3,PRDX3,PSEN2,VDAC1**
Mitochondrial L-carnitine Shuttle Pathway	2Gy 4h	0.00	0.25	NaN	CPT1A,CPT2
Mitotic Roles of Polo-Like Kinase	0.05Gy 4h	0.94	0.14	NaN	CDC25C,TGFB1
Mitotic Roles of Polo-Like Kinase	2Gy 2h	0.22	0.14	NaN	CDC25B,PPP2CA
Mitotic Roles of Polo-Like Kinase	2Gy 4h	0.95	0.43	-2.00	CCNB1,CDC25B,HSP90AB1,PPP2CA,SLK,TGFB1
Molecular Mechanisms of Cancer	0.05Gy 2h	0.56	0.02	NaN	BBC3,CDKN1B,MYC
**Molecular Mechanisms of Cancer**	**0.05Gy 4h**	**2.53**	**0.09**	**NaN**	**BBC3,BCL2,CDC25C,CDKN1A,E2F4,E2F5,FOS,GNAI2,JAK2,MAX,MDM2,PRKCD,PRKCI,RHOA,RHOG,TGFB1,TGFBR1**
**Molecular Mechanisms of Cancer**	**2Gy 2h**	**1.49**	**0.19**	**NaN**	**AURKA,BBC3,BCL2L1,BCL2L11,BIRC2,CAMK2G,CASP3,CASP8,CCNE1,CDC25B,CDC42,CDKN1A,CDKN1B,CFLAR,CHEK1,ELK1,FAS,GNAO1,IRS1,ITGA5,MAPK8,MAX,MDM2,MYC,PIK3CB,PIK3CD,PRKCE,RAC1,RHOB,RHOQ,RND3,RRAS2,SMAD3,SMAD7,SOS1,TYK2,ZBTB17**
**Molecular Mechanisms of Cancer**	**2Gy 4h**	**4.53**	**0.38**	**NaN**	**ABL1,ADCY8,AKT1,ARHGEF3,ATM,AURKA,AXIN1,BBC3,BCL2,BCL2L1,BID,BMP1,BMP2,BMP4,BMPR2,CASP3,CCNE1,CCNE2,CDC25B,CDK14,CDKN1A,CDKN2A,CDKN2B,CDKN2D,CFLAR,CRK,CTNNB1,E2F1,E2F5,ELK1,FAS,FOS,FZD1,GNAI2,GNAI3,GSK3B,HIF1A,HRAS,JAK2,KRAS,LEF1,MAP2K1,MAP3K7,MAPK8,MAX,MDM2,NFKB1,NFKBIA,PA2G4,PIK3CB,PIK3CD,PRKAR2A,PRKCD,PRKCE,PRKCI,PRKD3,PSEN2,PTCH1,RAC3,RASGRF1,RBPJ,RHOA,RRAS2,SMAD2,SMAD3,SMAD4,SMAD6,SMAD7,SRC,TCF4,TGFB1,TGFBR1,WNT5A,XIAP**
Mouse Embryonic Stem Cell Pluripotency	0.05Gy 2h	0.40	0.02	NaN	MYC
Mouse Embryonic Stem Cell Pluripotency	0.05Gy 4h	0.00	0.02	NaN	JAK2
Mouse Embryonic Stem Cell Pluripotency	2Gy 2h	0.00	0.13	-1.13	ID3,MYC,PIK3CB,PIK3CD,RRAS2,SOS1,TYK2

Ingenuity Canonical Pathways	Model	-log(p-value)	Ratio	z-score	Molecules
**Mouse Embryonic Stem Cell Pluripotency**	**2Gy 4h**	**3.45**	**0.47**	**0.00**	**AKT1,AXIN1,BMP4,BMPR2,CTNNB1,FZD1,GSK3B,HRAS,ID3,ID4,IL6ST,JAK2,KRAS,LEF1,LIF,LIFR,MAP2K1,MAP3K7,PIK3CB,PIK3CD,RRAS2,SMAD4,TCF4,TCF7,XIAP**
MSP-RON Signaling Pathway	0.05Gy 2h	0.66	0.04	NaN	IL12A
MSP-RON Signaling Pathway	0.05Gy 4h	0.52	0.08	NaN	ACTA2,JAK2
MSP-RON Signaling Pathway	2Gy 2h	0.29	0.15	NaN	ACTA2,CCL2,PIK3CB,PIK3CD
MSP-RON Signaling Pathway	2Gy 4h	0.00	0.23	NaN	ACTA2,CSF1,IL12A,JAK2,PIK3CB,PIK3CD
mTOR Signaling	0.05Gy 4h	0.80	0.07	0.45	PRKCD,PRKCI,RHOA,RHOG,STK11
**mTOR Signaling**	**2Gy 2h**	**1.63**	**0.24**	**-1.81**	**CDC42,HMOX1,IRS1,MTOR,NAPEPLD,PDPK1,PIK3CB,PIK3CD,PLD1,PPP2CA,PRKCE,RAC1,RHOB,RHOQ,RND3,RRAS2**
mTOR Signaling	2Gy 4h	1.20	0.34	-0.50	AKT1,EIF4A2,FAU,HIF1A,HMOX1,HRAS,KRAS,PGF,PIK3CB,PIK3CD,PLD1,PPP2CA,PRKCD,PRKCE,PRKCI,PRKD3,RAC3,RHOA,RPS19,RPS2,RPS29,RRAS2,STK11
Myc Mediated Apoptosis Signaling	0.05Gy 2h	0.58	0.03	NaN	MYC
Myc Mediated Apoptosis Signaling	0.05Gy 4h	0.00	0.03	NaN	BCL2
**Myc Mediated Apoptosis Signaling**	**2Gy 2h**	**1.55**	**0.28**	**NaN**	**CASP3,CASP8,FAS,MAPK8,MYC,PIK3CB,PIK3CD,RRAS2,SOS1**
Myc Mediated Apoptosis Signaling	2Gy 4h	1.10	0.38	NaN	AKT1,BCL2,BID,CASP3,CDKN2A,FAS,HRAS,KRAS,MAPK8,PIK3CB,PIK3CD,RRAS2
Myo-inositol Biosynthesis	2Gy 2h	0.58	0.50	NaN	IMPA2
NAD Biosynthesis from 2-amino-3-carboxymuconate Semialdehyde	2Gy 4h	0.60	1.00	NaN	ABL1
NAD biosynthesis II (from tryptophan)	2Gy 4h	0.60	1.00	NaN	ABL1
NAD Biosynthesis III	2Gy 2h	0.85	1.00	NaN	NAMPT
NAD Biosynthesis III	2Gy 4h	0.60	1.00	NaN	NAMPT
NADH Repair	2Gy 4h	0.60	1.00	NaN	GAPDH
Natural Killer Cell Signaling	0.05Gy 4h	0.65	0.08	NaN	PRKCD,PRKCI,VAV2
Natural Killer Cell Signaling	2Gy 2h	0.52	0.18	NaN	NCK1,PIK3CB,PIK3CD,PRKCE,RAC1,RRAS2,SOS1
Natural Killer Cell Signaling	2Gy 4h	0.81	0.33	NaN	AKT1,HRAS,KRAS,MAP2K1,PIK3CB,PIK3CD,PRKCD,PRKCE,PRKCI,PRKD3,RAC3,RRAS2,VAV2
NER Pathway	0.05Gy 4h	0.42	0.09	NaN	UBE2I
NER Pathway	2Gy 2h	0.33	0.18	NaN	PCNA,XPC
**NER Pathway**	**2Gy 4h**	**2.12**	**0.64**	**1.89**	**CCNH,DDB2,ERCC5,LIG1,PCNA,UBE2I,XPC**
Netrin Signaling	0.05Gy 4h	0.00	0.04	NaN	NFATC1
Netrin Signaling	2Gy 2h	1.21	0.27	-1.13	CACNA1A,NCK1,NFATC1,NFATC4,PPP3CC,PPP3R1,RAC1
Netrin Signaling	2Gy 4h	0.74	0.35	-1.67	CACNA1C,NFAT5,NFATC1,PPP3CA,PPP3CB,PPP3CC,PPP3R1,PRKAR2A,RAC3
Neuregulin Signaling	0.05Gy 2h	0.98	0.03	NaN	CDKN1B,MYC
Neuregulin Signaling	0.05Gy 4h	0.34	0.05	NaN	ERBIN,PRKCD,PRKCI
Neuregulin Signaling	2Gy 2h	0.50	0.17	-1.00	AREG,CDKN1B,ELK1,ITGA5,MTOR,MYC,PDPK1,PRKCE,RRAS2,SOS1
Neuregulin Signaling	2Gy 4h	0.70	0.31	-0.78	ADAM17,AKT1,CDK5R1,CRK,EGFR,ELK1,ERBIN,HRAS,HSP90AB1,KRAS,MAP2K1,PRKCD,PRKCE,PRKCI,PRKD3,PTEN,RRAS2,SRC
Neuroinflammation Signaling Pathway	0.05Gy 2h	0.75	0.02	NaN	IL12A,IL6,PTGS2
Neuroinflammation Signaling Pathway	0.05Gy 4h	0.00	0.04	-0.82	BCL2,FOS,JAK2,NFATC1,TGFB1,TGFBR1
Neuroinflammation Signaling Pathway	2Gy 2h	0.70	0.17	-0.78	ACVR2A,ATF4,BDNF,BIRC2,CASP3,CASP8,CCL2,CFLAR,CXCL8,FAS,HMOX1,IKBKB,IKBKE,IL6,IL6R,MAPK7,MAPK8,NFATC1,NFATC4,PIK3CB,PIK3CD,PPP3CC,PPP3R1,PTGS2,RIPK1,TYK2
Neuroinflammation Signaling Pathway	2Gy 4h	1.09	0.30	-1.81	ACVR1,ACVR1B,ACVR2A,AKT1,ATF2,ATF4,BCL2,BMPR2,CASP3,CFLAR,CNTF,CTNNB1,FAS,FOS,FZD1,GABRB2,GDNF,GLUL,GSK3B,HLA-E,HMOX1,ICAM1,IFNGR1,IL12A,IL1R1,IL6R,IRF7,JAK2,MAPK8,NFAT5,NFATC1,NFKB1,NOX4,PIK3CB,PIK3CD,PPP3CA,PPP3CB,PPP3CC,PPP3R1,PSEN2,PTGS2,PYCARD,RIPK1,TGFB1,TGFBR1,TRAF3,XIAP

Ingenuity Canonical Pathways	Model	-log(p-value)	Ratio	z-score	Molecules
Neuropathic Pain Signaling In Dorsal Horn Neurons	0.05Gy 4h	0.55	0.07	NaN	FOS,PRKCD,PRKCI
Neuropathic Pain Signaling In Dorsal Horn Neurons	2Gy 2h	0.00	0.14	-1.63	BDNF,CAMK2G,ELK1,PIK3CB,PIK3CD,PRKCE
Neuropathic Pain Signaling In Dorsal Horn Neurons	2Gy 4h	0.38	0.27	0.00	CAMK1,ELK1,FOS,ITPR2,PIK3CB,PIK3CD,PRKAR2A,PRKCD,PRKCE,PRKCI,PRKD3,SRC
Neuroprotective Role of THOP1 in Alzheimer's Disease	2Gy 4h	0.00	0.08	NaN	HLA-E,PREP,PRKAR2A
Neurotrophin/TRK Signaling	0.05Gy 2h	1.29	0.05	NaN	SPRY1,SPRY2
Neurotrophin/TRK Signaling	0.05Gy 4h	0.00	0.03	NaN	FOS
**Neurotrophin/TRK Signaling**	**2Gy 2h**	**2.26**	**0.31**	**-0.58**	**ATF4,BDNF,CDC42,MAP2K5,MAPK8,PDPK1,PIK3CB,PIK3CD,RRAS2,SOS1,SPRY1,SPRY2**
Neurotrophin/TRK Signaling	2Gy 4h	0.60	0.31	0.58	AKT1,ATF2,ATF4,FOS,HRAS,KRAS,MAP2K1,MAPK8,NGFR,PIK3CB,PIK3CD,RRAS2
NF-Î°B Activation by Viruses	0.05Gy 4h	0.22	0.04	NaN	PRKCD,PRKCI
NF-Î°B Activation by Viruses	2Gy 2h	0.47	0.17	-1.41	IKBKB,IKBKE,ITGA5,PIK3CB,PIK3CD,PRKCE,RIPK1,RRAS2
NF-Î°B Activation by Viruses	2Gy 4h	0.75	0.32	-0.26	AKT1,EIF2AK2,HRAS,KRAS,MAP3K1,NFKB1,NFKBIA,PIK3CB,PIK3CD,PRKCD,PRKCE,PRKCI,PRKD3,RIPK1,RRAS2
NF-Î°B Signaling	0.05Gy 4h	0.00	0.01	NaN	TGFBR1
NF-Î°B Signaling	2Gy 2h	0.00	0.09	0.63	CASP8,IKBKB,MAPK8,PIK3CB,PIK3CD,RELB,RIPK1,RRAS2,TNFAIP3,TNFRSF1B
NF-Î°B Signaling	2Gy 4h	0.49	0.27	-2.04	AKT1,BMP2,BMP4,BMPR2,CSNK2B,EGFR,EIF2AK2,FLT1,GHR,GSK3B,HRAS,IL1R1,KRAS,MAP3K1,MAP3K7,MAP4K4,MAPK8,NFKB1,NFKBIA,NGFR,PDGFRA,PIK3CB,PIK3CD,RIPK1,RRAS2,TGFBR1,TNFRSF11B,TNFRSF1B,TRAF3
NGF Signaling	0.05Gy 4h	0.41	0.06	NaN	PRKCD,RHOA,RHOG
**NGF Signaling**	**2Gy 2h**	**1.55**	**0.25**	**-0.28**	**ATF4,CDC42,ELK1,IKBKB,IKBKE,MAPK7,MAPK8,PDPK1,PIK3CB,PIK3CD,RAC1,RRAS2,SOS1**
**NGF Signaling**	**2Gy 4h**	**1.58**	**0.38**	**-0.47**	**AKT1,ATF2,ATF4,CRK,ELK1,HRAS,KRAS,MAP2K1,MAP3K1,MAP3K7,MAPK8,NFKB1,NGFR,PIK3CB,PIK3CD,PRKCD,RHOA,ROCK1,ROCK2,RRAS2**
Nicotine Degradation II	2Gy 4h	0.00	0.06	NaN	CYP1B1
Nicotine Degradation III	2Gy 4h	0.00	0.06	NaN	CYP1B1
Nitric Oxide Signaling in the Cardiovascular System	0.05Gy 4h	0.39	0.05	NaN	PRKCD,PRKCI,SLC7A1
Nitric Oxide Signaling in the Cardiovascular System	2Gy 2h	0.00	0.07	-2.00	CACNA1A,PIK3CB,PIK3CD,PRKCE
Nitric Oxide Signaling in the Cardiovascular System	2Gy 4h	0.54	0.29	0.26	AKT1,ATP2A2,CACNA1C,FLT1,HSP90AB1,ITPR2,MAP2K1,PGF,PIK3CB,PIK3CD,PRKAR2A,PRKCD,PRKCE,PRKCI,PRKD3,SLC7A1
nNOS Signaling in Neurons	0.05Gy 4h	0.63	0.09	NaN	PRKCD,PRKCI
nNOS Signaling in Neurons	2Gy 2h	0.00	0.14	NaN	PPP3CC,PPP3R1,PRKCE
nNOS Signaling in Neurons	2Gy 4h	0.79	0.36	-2.00	PPP3CA,PPP3CB,PPP3CC,PPP3R1,PRKCD,PRKCE,PRKCI,PRKD3
nNOS Signaling in Skeletal Muscle Cells	2Gy 2h	0.00	0.09	NaN	CACNA1A
nNOS Signaling in Skeletal Muscle Cells	2Gy 4h	0.00	0.09	NaN	CACNA1C
Non-Small Cell Lung Cancer Signaling	0.05Gy 4h	0.00	0.02	NaN	RXRB
Non-Small Cell Lung Cancer Signaling	2Gy 2h	0.60	0.19	-1.63	PDPK1,PIK3CB,PIK3CD,RASSF1,RRAS2,RXRA,RXRB,SOS1
**Non-Small Cell Lung Cancer Signaling**	**2Gy 4h**	**1.61**	**0.40**	**0.28**	**ABL1,AKT1,CDKN2A,E2F1,EGFR,FOXO3,HRAS,ITPR2,KRAS,MAP2K1,PA2G4,PIK3CB,PIK3CD,RASSF1,RRAS2,RXRB,STK4**
Notch Signaling	0.05Gy 2h	0.85	0.06	NaN	HES1
Notch Signaling	2Gy 2h	0.40	0.19	NaN	HES1,JAG2,LFNG
Notch Signaling	2Gy 4h	0.43	0.31	0.00	ADAM17,LFNG,NOTCH4,PSEN2,RBPJ
NRF2-mediated Oxidative Stress Response	0.05Gy 4h	0.82	0.07	0.00	ACTA2,FOS,FTH1,PRKCD,PRKCI
**NRF2-mediated Oxidative Stress Response**	**2Gy 2h**	**1.38**	**0.22**	**0.30**	**ACTA2,ATF4,CUL3,FTH1,HMOX1,JUNB,JUND,MAP2K5,MAPK7,MAPK8,PIK3CB,PIK3CD,PRDX1,PRKCE,RRAS2**
**NRF2-mediated Oxidative Stress Response**	**2Gy 4h**	**3.13**	**0.43**	**0.22**	**ACTA2,AKT1,ATF4,FOS,FTH1,GCLC,GSK3B,GSTP1,HMOX1,HRAS,JUNB,KRAS,MAP2K1,MAP3K1,MAP3K7,MAPK8,PIK3CB,PIK3CD,PPIB,PRDX1,PRKCD,PRKCE,PRKCI,PRKD3,RRAS2,SOD1,SOD3,TXN,VCP**

Ingenuity Canonical Pathways	Model	-log(p-value)	Ratio	z-score	Molecules
Nucleotide Excision Repair Pathway	2Gy 2h	0.44	0.33	NaN	XPC
**Nucleotide Excision Repair Pathway**	**2Gy 4h**	**1.80**	**1.00**	**NaN**	**CCNH,ERCC5,XPC**
Nur77 Signaling in T Lymphocytes	0.05Gy 4h	0.57	0.08	NaN	BCL2,NFATC1
**Nur77 Signaling in T Lymphocytes**	**2Gy 2h**	**2.44**	**0.38**	**NaN**	**CASP3,MAP2K5,MAPK7,MEF2D,NFATC1,NR4A1,PPP3CC,PPP3R1,RXRA**
Nur77 Signaling in T Lymphocytes	2Gy 4h	0.41	0.29	NaN	BCL2,CASP3,NFATC1,PPP3CA,PPP3CB,PPP3CC,PPP3R1
Oleate Biosynthesis II (Animals)	2Gy 4h	0.24	0.33	NaN	FADS1
Oncostatin M Signaling	0.05Gy 4h	0.60	0.09	NaN	JAK2,MT2A
Oncostatin M Signaling	2Gy 2h	0.39	0.17	0.00	ELK1,RRAS2,SOS1,TYK2
Oncostatin M Signaling	2Gy 4h	0.71	0.35	-0.71	ELK1,HRAS,IL6ST,JAK2,KRAS,MAP2K1,MT2A,RRAS2
Opioid Signaling Pathway	0.05Gy 2h	0.65	0.02	NaN	CDKN1B,MYC
Opioid Signaling Pathway	0.05Gy 4h	0.68	0.06	0.82	FOS,GNAI2,GNG5,PRKCD,PRKCI,YES1
Opioid Signaling Pathway	2Gy 2h	0.96	0.19	-0.24	ATF4,CACNA1A,CAMK2G,CDC42,CDKN1B,ELK1,GNAO1,MAP2K5,MAPK7,MYC,PPP3CC,PPP3R1,PRKCE,RAC1,RGS12,RGS4,RRAS2,SOS1
**Opioid Signaling Pathway**	**2Gy 4h**	**2.20**	**0.37**	**-0.85**	**ADCY8,AKT1,ATF2,ATF4,CACNA1C,CAMK1,CTNNB1,ELK1,FOS,GNAI2,GNAI3,GNG5,GSK3B,HRAS,ITPR2,KRAS,MAP2K1,NFKB1,NFKBIA,PPP3CA,PPP3CB,PPP3CC,PPP3R1,PRKAR2A,PRKCD,PRKCE,PRKCI,PRKD3,RAC3,RGS20,RGS4,RRAS2,SRC,SRF,YES1**
Osteoarthritis Pathway	0.05Gy 2h	0.51	0.02	NaN	HES1,PTGS2
Osteoarthritis Pathway	0.05Gy 4h	0.00	0.02	NaN	TGFB1,TGFBR1
Osteoarthritis Pathway	2Gy 2h	0.78	0.18	0.94	ATF4,CASP3,CASP8,CEBPB,CXCL8,FGF2,GLI3,HES1,ITGA5,MTOR,NAMPT,PPARGC1A,PTGS2,PTHLH,RAC1,RUNX2,S1PR3,SDC4,SMAD3,SMAD7,TNFRSF1B
Osteoarthritis Pathway	2Gy 4h	0.53	0.28	-0.93	ATF2,ATF4,BMP2,BMPR2,CASP3,CTNNB1,DKK1,FGF2,FOXO3,FZD1,GLI3,GREM1,HIF1A,IL1R1,LEF1,NAMPT,NFKB1,PGF,PTCH1,PTGS2,RBPJ,SMAD2,SMAD3,SMAD4,SMAD6,SMAD7,SOX9,SP1,TCF4,TCF7,TGFB1,TGFBR1,TNFRSF1B
Ovarian Cancer Signaling	0.05Gy 2h	0.28	0.01	NaN	PTGS2
Ovarian Cancer Signaling	0.05Gy 4h	0.00	0.01	NaN	BCL2
Ovarian Cancer Signaling	2Gy 2h	0.00	0.10	-1.34	EDN1,FGF9,MSH6,MTOR,PIK3CB,PIK3CD,PTGS2,RRAS2
**Ovarian Cancer Signaling**	**2Gy 4h**	**2.17**	**0.38**	**0.28**	**ABL1,AKT1,AXIN1,BCL2,BRCA2,CD44,CDKN2A,CTNNB1,E2F1,EGFR,FZD1,GJA1,GSK3B,HRAS,KRAS,LEF1,MAP2K1,MSH6,PA2G4,PGF,PIK3CB,PIK3CD,PRKAR2A,PTEN,PTGS2,RRAS2,SRC,TCF4,TCF7,WNT5A**
OX40 Signaling Pathway	0.05Gy 4h	0.27	0.06	NaN	BCL2
OX40 Signaling Pathway	2Gy 2h	0.00	0.11	NaN	BCL2L1,MAPK8
OX40 Signaling Pathway	2Gy 4h	0.86	0.39	-1.63	BCL2,BCL2L1,HLA-E,MAPK8,NFKB1,NFKBIA,TRAF3
Oxidative Phosphorylation	0.05Gy 4h	0.72	0.11	NaN	NDUFS8,NDUFV3
Oxidative Phosphorylation	2Gy 4h	0.76	0.37	1.89	ATP5F1A,ATP5PF,NDUFS1,NDUFS3,NDUFS8,NDUFV1,NDUFV3
P2Y Purigenic Receptor Signaling Pathway	0.05Gy 2h	0.36	0.02	NaN	MYC
P2Y Purigenic Receptor Signaling Pathway	0.05Gy 4h	0.95	0.08	0.45	FOS,GNAI2,GNG5,PRKCD,PRKCI
P2Y Purigenic Receptor Signaling Pathway	2Gy 2h	0.00	0.10	-1.63	ATF4,MYC,PIK3CB,PIK3CD,PRKCE,RRAS2
P2Y Purigenic Receptor Signaling Pathway	2Gy 4h	0.98	0.33	0.45	ADCY8,AKT1,ATF2,ATF4,FOS,GNAI2,GNAI3,GNG5,HRAS,KRAS,MAP2K1,NFKB1,PIK3CB,PIK3CD,PRKAR2A,PRKCD,PRKCE,PRKCI,PRKD3,RRAS2
p38 MAPK Signaling	0.05Gy 2h	0.47	0.02	NaN	MYC
p38 MAPK Signaling	0.05Gy 4h	0.55	0.07	NaN	MAX,TGFB1,TGFBR1
p38 MAPK Signaling	2Gy 2h	0.81	0.21	0.00	ATF4,CDC25B,DUSP1,ELK1,FAS,MAX,MEF2D,MYC,TNFRSF1B
p38 MAPK Signaling	2Gy 4h	0.72	0.32	-0.83	ATF2,ATF4,CDC25B,DDIT3,DUSP1,ELK1,FAS,IL1R1,MAP3K7,MAX,SRF,TGFB1,TGFBR1,TNFRSF1B
p53 Signaling	0.05Gy 2h	0.97	0.03	NaN	BBC3,SNAI2

Ingenuity Canonical Pathways	Model	-log(p-value)	Ratio	z-score	Molecules
p53 Signaling	0.05Gy 4h	0.97	0.08	0.45	BBC3,BCL2,CDKN1A,GNL3,MDM2
**p53 Signaling**	**2Gy 2h**	**1.79**	**0.25**	**1.94**	**BBC3,BCL2L1,CDKN1A,CHEK1,FAS,GNL3,MAPK8,MDM2,PCNA,PIK3CB,PIK3CD,SNAI2,TNFRSF10B,TP53BP2,TP53INP1**
**p53 Signaling**	**2Gy 4h**	**3.27**	**0.45**	**0.82**	**AKT1,ATM,BBC3,BCL2,BCL2L1,CDKN1A,CDKN2A,CTNNB1,E2F1,FAS,GADD45A,GNL3,GSK3B,HIF1A,MAPK8,MDM2,PCNA,PIK3CB,PIK3CD,PLAGL1,PTEN,SNAI2,TNFRSF10A,TNFRSF10B,TP53BP2,TP53INP1,TP63**
p70S6K Signaling	0.05Gy 4h	0.35	0.05	NaN	GNAI2,PRKCD,PRKCI
p70S6K Signaling	2Gy 2h	0.74	0.19	-2.71	IL4R,IRS1,MTOR,PDPK1,PIK3CB,PIK3CD,PLD1,PPP2CA,PRKCE,RRAS2,SOS1
p70S6K Signaling	2Gy 4h	0.94	0.33	0.23	AKT1,BCAP31,EGFR,GNAI2,GNAI3,HRAS,IL4R,KRAS,MAP2K1,PIK3CB,PIK3CD,PLD1,PPP2CA,PRKCD,PRKCE,PRKCI,PRKD3,RRAS2,SRC
**PAK Signaling**	**2Gy 2h**	**2.43**	**0.31**	**0.28**	**CASP3,CDC42,EPHB3,ITGA5,LIMK2,MAPK8,NCK1,PDGFA,PIK3CB,PIK3CD,RAC1,RRAS2,SOS1**
PAK Signaling	2Gy 4h	0.45	0.29	-0.91	CASP3,CFL1,HRAS,KRAS,LIMK2,MAP2K1,MAPK8,PDGFRA,PIK3CB,PIK3CD,PTK2B,RRAS2
Pancreatic Adenocarcinoma Signaling	0.05Gy 2h	0.85	0.03	NaN	CDKN1B,PTGS2
**Pancreatic Adenocarcinoma Signaling**	**0.05Gy 4h**	**2.04**	**0.11**	**-1.00**	**BCL2,CDKN1A,E2F4,E2F5,JAK2,MDM2,TGFB1,TGFBR1**
**Pancreatic Adenocarcinoma Signaling**	**2Gy 2h**	**1.78**	**0.24**	**-0.54**	**BCL2L1,CCNE1,CDC42,CDKN1A,CDKN1B,ELK1,HMOX1,MAPK8,MDM2,NAPEPLD,PIK3CB,PIK3CD,PLD1,PTGS2,RAC1,SMAD3,TYK2**
**Pancreatic Adenocarcinoma Signaling**	**2Gy 4h**	**3.39**	**0.44**	**-0.69**	**ABL1,AKT1,BCL2,BCL2L1,BRCA2,CCNE1,CDKN1A,CDKN2A,CDKN2B,E2F1,E2F5,EGFR,ELK1,HMOX1,JAK2,KRAS,MAP2K1,MAPK8,MDM2,NFKB1,PA2G4,PGF,PIK3CB,PIK3CD,PLD1,PTGS2,SMAD2,SMAD3,SMAD4,TGFB1,TGFBR1**
**Parkinson's Signaling**	**2Gy 2h**	**1.70**	**1.00**	**NaN**	**CASP3,MAPK8**
Parkinson's Signaling	2Gy 4h	1.20	1.00	NaN	CASP3,MAPK8
Paxillin Signaling	0.05Gy 4h	0.25	0.05	NaN	ACTA2,ARF6
**Paxillin Signaling**	**2Gy 2h**	**1.43**	**0.25**	**-0.30**	**ACTA2,ARF6,CDC42,ITGA5,MAPK8,NCK1,PIK3CB,PIK3CD,RAC1,RRAS2,SOS1**
Paxillin Signaling	2Gy 4h	0.72	0.32	-0.83	ACTA2,ARF1,ARF6,BCAR1,CRK,HRAS,ITGB8,KRAS,MAPK8,PIK3CB,PIK3CD,PTK2B,RRAS2,SRC
PCP pathway	0.05Gy 4h	0.00	0.04	NaN	RHOA
PCP pathway	2Gy 2h	0.52	0.19	1.34	EFNB1,JUNB,JUND,MAPK8,RAC1
PCP pathway	2Gy 4h	1.04	0.39	-0.63	ATF2,EFNB1,FZD1,JUNB,MAPK8,PFN1,RHOA,ROCK1,ROCK2,WNT5A
PD-1, PD-L1 cancer immunotherapy pathway	0.05Gy 2h	1.06	0.04	NaN	CDKN1B,IL12A
PD-1, PD-L1 cancer immunotherapy pathway	0.05Gy 4h	0.00	0.04	NaN	JAK2,TGFB1
PD-1, PD-L1 cancer immunotherapy pathway	2Gy 2h	0.49	0.17	0.33	BCL2L1,CD274,CDKN1B,ELK1,PIK3CB,PIK3CD,SMAD3,TNFRSF1B,TYK2
**PD-1, PD-L1 cancer immunotherapy pathway**	**2Gy 4h**	**1.58**	**0.38**	**-1.00**	**AKT1,BCL2L1,CSNK2B,ELK1,GSK3B,HLA-E,IFNGR1,IL12A,JAK2,LATS1,NGFR,PDCD1LG2,PIK3CB,PIK3CD,PTEN,SMAD3,TGFB1,TNFRSF11B,TNFRSF1B,YAP1**
PDGF Signaling	0.05Gy 2h	0.47	0.02	NaN	MYC
PDGF Signaling	0.05Gy 4h	0.25	0.05	NaN	FOS,JAK2
PDGF Signaling	2Gy 2h	0.81	0.21	-0.33	ELK1,MAPK8,MYC,PDGFA,PIK3CB,PIK3CD,RRAS2,SOS1,TYK2
**PDGF Signaling**	**2Gy 4h**	**1.84**	**0.41**	**-0.94**	**ABL1,CRK,CSNK2B,EIF2AK2,ELK1,FOS,HRAS,JAK2,KRAS,MAP2K1,MAP3K1,MAPK8,PDGFRA,PIK3CB,PIK3CD,RRAS2,SRC,SRF**
PEDF Signaling	0.05Gy 4h	0.27	0.05	NaN	BCL2,RHOA
**PEDF Signaling**	**2Gy 2h**	**1.98**	**0.29**	**-0.58**	**BCL2L1,BDNF,CASP8,CFLAR,ELK1,FAS,IKBKB,IKBKE,PIK3CB,PIK3CD,RAC1,RRAS2**
**PEDF Signaling**	**2Gy 4h**	**2.93**	**0.48**	**0.23**	**AKT1,BCL2,BCL2L1,CFLAR,ELK1,FAS,GDNF,HRAS,KRAS,NFKB1,NFKBIA,PIK3CB,PIK3CD,RHOA,ROCK1,ROCK2,RRAS2,SRF,TCF4,TCF7**
**Pentose Phosphate Pathway**	**0.05Gy 4h**	**1.37**	**1.00**	**NaN**	**G6PD**

Ingenuity Canonical Pathways	Model	-log(p-value)	Ratio	z-score	Molecules
Pentose Phosphate Pathway	2Gy 4h	0.60	1.00	NaN	G6PD
**Pentose Phosphate Pathway (Oxidative Branch)**	**0.05Gy 4h**	**1.37**	**1.00**	**NaN**	**G6PD**
Pentose Phosphate Pathway (Oxidative Branch)	2Gy 4h	0.60	1.00	NaN	G6PD
**PFKFB4 Signaling Pathway**	**0.05Gy 4h**	**1.31**	**0.15**	**NaN**	**GPI,HK2,TGFB1**
PFKFB4 Signaling Pathway	2Gy 2h	0.51	0.20	0.00	ATF4,FGF2,HK2,MAP2K5
PFKFB4 Signaling Pathway	2Gy 4h	0.99	0.40	0.00	ATF2,ATF4,FGF2,GPI,HK2,MAP2K1,PRKAR2A,TGFB1
Phagosome Formation	0.05Gy 4h	1.07	0.09	NaN	MARCKS,PRKCD,PRKCI,RHOA,RHOG
Phagosome Formation	2Gy 2h	0.59	0.18	NaN	CDC42,ITGA5,MARCKS,PIK3CB,PIK3CD,PRKCE,RAC1,RHOB,RHOQ,RND3
Phagosome Formation	2Gy 4h	0.00	0.16	NaN	MARCKS,PIK3CB,PIK3CD,PRKCD,PRKCE,PRKCI,PRKD3,RAC3,RHOA
Phagosome Maturation	0.05Gy 4h	0.34	0.06	NaN	ATP6V1B2,TAP1
Phagosome Maturation	2Gy 2h	0.00	0.14	NaN	ATP6V0A1,PRDX1,STX16,TUBB2A,TUBB4B
Phagosome Maturation	2Gy 4h	0.56	0.31	NaN	ATP6V0A1,ATP6V1B2,ATP6V1E1,CALR,CTSD,HLA-E,NOX4,PRDX1,PRDX6,TAP1,TUBB2A
Phosphatidylcholine Biosynthesis I	2Gy 2h	0.34	0.25	NaN	CHPT1
Phospholipase C Signaling	0.05Gy 4h	0.97	0.07	1.13	GNG5,MARCKS,NFATC1,PRKCD,PRKCI,RHOA,RHOG
**Phospholipase C Signaling**	**2Gy 2h**	**1.38**	**0.21**	**-1.50**	**ATF4,CDC42,HDAC6,HMOX1,ITGA5,MARCKS,MEF2D,NAPEPLD,NFATC1,NFATC4,PLD1,PPP3CC,PPP3R1,PRKCE,RAC1,RHOB,RHOQ,RND3,RRAS2,SOS1**
Phospholipase C Signaling	2Gy 4h	0.70	0.29	-0.43	ADCY8,ARHGEF3,ATF2,ATF4,GNG5,HMOX1,HRAS,ITPR2,KRAS,MAP2K1,MARCKS,NFAT5,NFATC1,NFKB1,PEBP1,PLD1,PPP3CA,PPP3CB,PPP3CC,PPP3R1,PRKCD,PRKCE,PRKCI,PRKD3,RAC3,RHOA,RRAS2,SRC
Phospholipases	2Gy 2h	0.56	0.21	0.00	HMOX1,NAPEPLD,PLD1,PNPLA8
Phospholipases	2Gy 4h	0.00	0.16	NaN	HMOX1,PLD1,PNPLA8
Phototransduction Pathway	2Gy 4h	0.00	0.07	NaN	PRKAR2A
PI3K Signaling in B Lymphocytes	0.05Gy 4h	0.50	0.06	1.00	FOS,NFATC1,PRKCI,VAV2
**PI3K Signaling in B Lymphocytes**	**2Gy 2h**	**2.03**	**0.25**	**-1.81**	**ATF4,CAMK2G,ELK1,IKBKB,IKBKE,IL4R,IRS1,IRS2,NFATC1,NFATC4,PDPK1,PIK3CB,PIK3CD,PPP3CC,PPP3R1,RAC1,RRAS2**
**PI3K Signaling in B Lymphocytes**	**2Gy 4h**	**2.41**	**0.40**	**-1.57**	**ABL1,AKT1,ATF2,ATF4,ATF6,ELK1,FOS,FOXO3,HRAS,IL4R,ITPR2,KRAS,MAP2K1,NFAT5,NFATC1,NFKB1,NFKBIA,PIK3CB,PIK3CD,PPP3CA,PPP3CB,PPP3CC,PPP3R1,PRKCI,PTEN,RRAS2,VAV2**
PI3K/AKT Signaling	0.05Gy 2h	0.67	0.02	NaN	CDKN1B,PTGS2
PI3K/AKT Signaling	0.05Gy 4h	0.26	0.04	-1.00	BCL2,CDKN1A,JAK2,MDM2
PI3K/AKT Signaling	2Gy 2h	1.06	0.20	-2.00	BCL2L1,CDKN1A,CDKN1B,IKBKB,IKBKE,IL4R,IL6R,ITGA5,MDM2,MTOR,PDPK1,PIK3CB,PIK3CD,PPP2CA,PTGS2,RRAS2,SOS1,TYK2
PI3K/AKT Signaling	2Gy 4h	1.03	0.32	0.45	AKT1,BCL2,BCL2L1,CDKN1A,CTNNB1,FOXO3,GHR,GSK3B,HRAS,HSP90AB1,IL17RA,IL18R1,IL1R1,IL22RA1,IL4R,IL6R,IL6ST,JAK2,KRAS,MAP2K1,MDM2,NFKB1,NFKBIA,PIK3CB,PIK3CD,PPP2CA,PTEN,PTGS2,RRAS2
PKCÎ¸ Signaling in T Lymphocytes	0.05Gy 4h	0.39	0.05	NaN	FOS,NFATC1,VAV2
**PKCÎ¸ Signaling in T Lymphocytes**	**2Gy 2h**	**1.77**	**0.26**	**-1.39**	**CACNA1A,CAMK2G,IKBKB,IKBKE,MAPK8,NFATC1,NFATC4,PIK3CB,PIK3CD,PPP3CC,PPP3R1,RAC1,RRAS2,SOS1**
**PKCÎ¸ Signaling in T Lymphocytes**	**2Gy 4h**	**1.40**	**0.36**	**-1.15**	**CACNA1C,FOS,HRAS,KRAS,MAP3K1,MAP3K7,MAPK8,NFAT5,NFATC1,NFKB1,NFKBIA,PIK3CB,PIK3CD,PPP3CA,PPP3CB,PPP3CC,PPP3R1,RAC3,RRAS2,VAV2**
Polyamine Regulation in Colon Cancer	0.05Gy 2h	1.09	0.11	NaN	MYC
Polyamine Regulation in Colon Cancer	0.05Gy 4h	0.49	0.11	NaN	MAX
**Polyamine Regulation in Colon Cancer**	**2Gy 2h**	**1.57**	**0.44**	**NaN**	**MAX,MXD1,MYC,SAT1**
**Polyamine Regulation in Colon Cancer**	**2Gy 4h**	**1.31**	**0.56**	**NaN**	**CTNNB1,KRAS,MAX,SAT1,TCF4**
PPAR Signaling	0.05Gy 2h	0.36	0.02	NaN	PTGS2

Ingenuity Canonical Pathways	Model	-log(p-value)	Ratio	z-score	Molecules
PPAR Signaling	0.05Gy 4h	0.00	0.02	NaN	FOS
PPAR Signaling	2Gy 2h	0.45	0.16	0.00	IKBKB,IKBKE,NCOR2,PDGFA,PPARGC1A,PTGS2,RRAS2,RXRA,SOS1,TNFRSF1B
PPAR Signaling	2Gy 4h	0.60	0.30	-0.47	FOS,HRAS,HSP90AB1,IL1R1,KRAS,MAP2K1,MAP3K7,MAP4K4,NFKB1,NFKBIA,NGFR,NR1H3,PDGFRA,PPARA,PTGS2,RRAS2,TNFRSF11B,TNFRSF1B
PPARÎ±/RXRÎ± Activation	0.05Gy 2h	0.65	0.02	NaN	BCL3,IL6
PPARÎ±/RXRÎ± Activation	0.05Gy 4h	0.00	0.03	NaN	JAK2,TGFB1,TGFBR1
PPARÎ±/RXRÎ± Activation	2Gy 2h	0.00	0.14	1.39	ACVR2A,BCL3,IKBKB,IKBKE,IL6,IRS1,MAPK8,NCOR2,PPARGC1A,RRAS2,RXRA,SMAD3,SOS1
PPARÎ±/RXRÎ± Activation	2Gy 4h	0.60	0.28	1.96	ACVR1,ACVR1B,ACVR2A,ADCY8,ADIPOR2,BMPR2,GHR,HRAS,HSP90AB1,IL1R1,JAK2,KRAS,MAP2K1,MAP3K7,MAP4K4,MAPK8,NFKB1,NFKBIA,NR2C2,PPARA,PRKAR2A,RRAS2,SMAD2,SMAD3,SMAD4,TGFB1,TGFBR1
Pregnenolone Biosynthesis	2Gy 4h	0.00	0.17	NaN	CYP7B1
Primary Immunodeficiency Signaling	0.05Gy 4h	0.37	0.08	NaN	TAP1
Primary Immunodeficiency Signaling	2Gy 4h	0.00	0.15	NaN	TAP1,UNG
Production of Nitric Oxide and Reactive Oxygen Species in Macrophages	0.05Gy 4h	0.78	0.07	0.82	FOS,JAK2,PRKCD,PRKCI,RHOA,RHOG
Production of Nitric Oxide and Reactive Oxygen Species in Macrophages	2Gy 2h	0.60	0.17	-1.60	APOE,CDC42,IKBKB,IKBKE,MAPK8,PIK3CB,PIK3CD,PPP2CA,PRKCE,RAC1,RHOB,RHOQ,RND3,TNFRSF1B,TYK2
Production of Nitric Oxide and Reactive Oxygen Species in Macrophages	2Gy 4h	0.46	0.27	-0.63	AKT1,FOS,IFNGR1,JAK2,MAP2K1,MAP3K1,MAP3K7,MAPK8,NFKB1,NFKBIA,NGFR,PIK3CB,PIK3CD,PPARA,PPP1CA,PPP2CA,PRKCD,PRKCE,PRKCI,PRKD3,RAC3,RHOA,TNFRSF11B,TNFRSF1B
Prolactin Signaling	0.05Gy 2h	1.12	0.04	NaN	MYC,SOCS3
**Prolactin Signaling**	**0.05Gy 4h**	**1.80**	**0.12**	**0.45**	**FOS,JAK2,NR3C1,PRKCD,PRKCI,SOCS6**
**Prolactin Signaling**	**2Gy 2h**	**2.23**	**0.29**	**-1.94**	**CEBPB,IRS1,MYC,PDPK1,PIK3CB,PIK3CD,PRKCE,RRAS2,SOCS1,SOCS2,SOCS3,SOCS5,SOCS6,SOS1**
**Prolactin Signaling**	**2Gy 4h**	**2.35**	**0.43**	**0.00**	**FOS,HRAS,JAK2,KRAS,MAP2K1,NR3C1,PIK3CB,PIK3CD,PRKCD,PRKCE,PRKCI,PRKD3,PRLR,RRAS2,SOCS2,SOCS3,SOCS5,SOCS6,SOCS7,SP1,TCF7**
Proline Biosynthesis I	2Gy 4h	1.20	1.00	NaN	ALDH18A1,PYCR1
Proline Biosynthesis II (from Arginine)	2Gy 2h	0.44	0.33	NaN	OAT
Proline Biosynthesis II (from Arginine)	2Gy 4h	0.80	0.67	NaN	OAT,PYCR1
Prostanoid Biosynthesis	0.05Gy 2h	1.26	0.17	NaN	PTGS2
Prostanoid Biosynthesis	2Gy 2h	0.70	0.33	NaN	PTGES3,PTGS2
**Prostanoid Biosynthesis**	**2Gy 4h**	**1.42**	**0.67**	**2.00**	**PTGES,PTGES2,PTGES3,PTGS2**
Prostate Cancer Signaling	0.05Gy 2h	0.41	0.02	NaN	CDKN1B
Prostate Cancer Signaling	0.05Gy 4h	0.42	0.06	NaN	BCL2,CDKN1A,MDM2
Prostate Cancer Signaling	2Gy 2h	0.98	0.21	NaN	ATF4,CCNE1,CDKN1A,CDKN1B,MDM2,MTOR,PDPK1,PIK3CB,PIK3CD,RRAS2,SOS1
**Prostate Cancer Signaling**	**2Gy 4h**	**3.62**	**0.48**	**NaN**	**ABL1,AKT1,ATF2,ATF4,BCL2,CCNE1,CCNE2,CDKN1A,CTNNB1,E2F1,GSK3B,GSTP1,HRAS,HSP90AB1,KRAS,LEF1,MAP2K1,MDM2,NFKB1,NFKBIA,PA2G4,PIK3CB,PIK3CD,PTEN,RRAS2**
Protein Kinase A Signaling	0.05Gy 2h	0.00	0.01	NaN	PTGS2
**Protein Kinase A Signaling**	**0.05Gy 4h**	**1.31**	**0.08**	**0.63**	**CDC25C,GNAI2,GNG5,NFATC1,PRKCD,PRKCI,PTPN13,RHOA,TGFB1,TGFBR1**
Protein Kinase A Signaling	2Gy 2h	0.30	0.14	-1.89	ATF4,CAMK2G,CDC25B,DUSP1,DUSP6,ELK1,GLI3,LIPE,NAPEPLD,NFATC1,NFATC4,PDE4D,PPP3CC,PPP3R1,PRKCE,PTGS2,PTP4A1,PTPN2,SMAD3

Ingenuity Canonical Pathways	Model	-log(p-value)	Ratio	z-score	Molecules
**Protein Kinase A Signaling**	**2Gy 4h**	**4.53**	**0.41**	**-0.30**	**ADCY8,APEX1,ATF2,ATF4,CDC25B,CREM,CTNNB1,DUSP1,DUSP6,ELK1,GLI3,GNAI2,GNAI3,GNG5,GSK3B,ITPR2,LEF1,LIPE,MAP2K1,MAP3K1,NFAT5,NFATC1,NFKB1,NFKBIA,NGFR,PPP1CA,PPP3CA,PPP3CB,PPP3CC,PPP3R1,PRKAR2A,PRKCD,PRKCE,PRKCI,PRKD3,PTCH1,PTEN,PTGS2,PTK2B,PTP4A1,PTPN13,PTPN9,PTPRE,PTPRK,PTPRS,RHOA,ROCK1,ROCK2,SMAD3,SMAD4,TCF4,TCF7,TGFB1,TGFBR1**
**Protein Ubiquitination Pathway**	**0.05Gy 4h**	**2.00**	**0.16**	**NaN**	**MDM2,PSMB9,TAP1,UBE2I,USP53**
Protein Ubiquitination Pathway	2Gy 2h	0.54	0.19	NaN	BIRC2,HSPA4L,HSPA9,MDM2,UBE2E1,USP2
**Protein Ubiquitination Pathway**	**2Gy 4h**	**1.43**	**0.41**	**NaN**	**HLA-E,HSP90AB1,HSPA4L,HSPA6,HSPA8,MDM2,PSMB9,STUB1,TAP1,UBC,UBE2I,USP53,XIAP**
PTEN Signaling	0.05Gy 2h	0.28	0.01	NaN	CDKN1B
PTEN Signaling	0.05Gy 4h	0.00	0.04	NaN	BCL2,CDKN1A,TGFBR1
PTEN Signaling	2Gy 2h	0.94	0.20	1.29	BCL2L1,BCL2L11,CASP3,CDC42,CDKN1A,CDKN1B,IKBKB,IKBKE,ITGA5,PDPK1,PIK3CB,PIK3CD,RAC1,RRAS2,SOS1
PTEN Signaling	2Gy 4h	1.07	0.33	1.00	AKT1,BCAR1,BCL2,BCL2L1,BMPR2,CASP3,CDKN1A,CSNK2B,EGFR,FLT1,FOXO3,GHR,GSK3B,HRAS,KRAS,MAP2K1,NFKB1,NGFR,PDGFRA,PIK3CB,PIK3CD,PTEN,RAC3,RRAS2,TGFBR1
Putrescine Degradation III	2Gy 2h	0.00	0.13	NaN	SAT1
Putrescine Degradation III	2Gy 4h	0.00	0.13	NaN	SAT1
PXR/RXR Activation	0.05Gy 2h	0.42	0.02	NaN	IL6
PXR/RXR Activation	0.05Gy 4h	0.00	0.02	NaN	NR3C1
PXR/RXR Activation	2Gy 2h	0.00	0.08	NaN	ALAS1,IL6,PPARGC1A,RXRA
PXR/RXR Activation	2Gy 4h	0.00	0.14	NaN	AKT1,CES2,CPT1A,FOXO3,NR3C1,PPARA,PRKAR2A
Pyridoxal 5'-phosphate Salvage Pathway	0.05Gy 4h	0.21	0.05	NaN	PRKCD
Pyridoxal 5'-phosphate Salvage Pathway	2Gy 2h	1.11	0.27	-0.82	ACVR2A,LIMK2,MAPK7,MAPK8,PRKCE,SGK1
Pyridoxal 5'-phosphate Salvage Pathway	2Gy 4h	0.52	0.32	0.38	ACVR2A,EIF2AK2,LIMK2,MAP2K1,MAPK8,PRKCD,PRKCE
Pyrimidine Deoxyribonucleotides De Novo Biosynthesis I	0.05Gy 4h	1.08	0.50	NaN	NME2
Pyrimidine Deoxyribonucleotides De Novo Biosynthesis I	2Gy 4h	0.36	0.50	NaN	NME2
Pyrimidine Ribonucleotides De Novo Biosynthesis	0.05Gy 4h	0.64	0.17	NaN	NME2
Pyrimidine Ribonucleotides De Novo Biosynthesis	2Gy 2h	0.22	0.17	NaN	CAD
Pyrimidine Ribonucleotides De Novo Biosynthesis	2Gy 4h	0.00	0.17	NaN	NME2
Pyrimidine Ribonucleotides Interconversion	0.05Gy 4h	0.71	0.20	NaN	NME2
Pyrimidine Ribonucleotides Interconversion	2Gy 4h	0.00	0.20	NaN	NME2
Rac Signaling	0.05Gy 4h	0.24	0.04	NaN	PRKCI,RHOA
Rac Signaling	2Gy 2h	1.04	0.22	0.00	CDC42,ELK1,ITGA5,LIMK2,MAPK8,PIK3CB,PIK3CD,PLD1,RAC1,RRAS2
**Rac Signaling**	**2Gy 4h**	**2.09**	**0.42**	**-1.41**	**CD44,CDK5R1,CFL1,ELK1,HRAS,KRAS,LIMK2,MAP2K1,MAP3K1,MAPK8,NFKB1,NOX4,PIK3CB,PIK3CD,PLD1,PRKCI,PTK2B,RHOA,RRAS2**
RAN Signaling	2Gy 2h	0.27	0.20	NaN	KPNA4
RAN Signaling	2Gy 4h	0.43	0.40	NaN	KPNA4,RANBP1
RANK Signaling in Osteoclasts	0.05Gy 4h	0.24	0.04	NaN	FOS,NFATC1
**RANK Signaling in Osteoclasts**	**2Gy 2h**	**1.37**	**0.24**	**-1.51**	**BIRC2,ELK1,IKBKB,IKBKE,MAPK8,MITF,NFATC1,PIK3CB,PIK3CD,PPP3CC,PPP3R1**
**RANK Signaling in Osteoclasts**	**2Gy 4h**	**2.09**	**0.42**	**-1.41**	**AKT1,ELK1,FOS,MAP2K1,MAP3K1,MAP3K7,MAPK8,NFATC1,NFKB1,NFKBIA,PIK3CB,PIK3CD,PPP3CA,PPP3CB,PPP3CC,PPP3R1,PTK2B,SRC,XIAP**
**RAR Activation**	**0.05Gy 4h**	**2.13**	**0.10**	**NaN**	**FOS,JAK2,PNRC1,PRKCD,PRKCI,RARG,RXRB,SMARCA4,TGFB1,TRIM24**
RAR Activation	2Gy 2h	0.53	0.16	NaN	DUSP1,MAPK8,NCOR2,PDPK1,PIK3CB,PIK3CD,PPARGC1A,PRKCE,RAC1,RARG,RELB,RXRA,RXRB,SMAD3,SMAD7,TRIM24

Ingenuity Canonical Pathways	Model	-log(p-value)	Ratio	z-score	Molecules
**RAR Activation**	**2Gy 4h**	**1.72**	**0.35**	**NaN**	**ADCY8,AKT1,BMP2,CCNH,CRABP2,CSNK2B,DUSP1,FOS,JAK2,MAP2K1,MAP3K1,MAPK8,NFKB1,PIK3CB,PIK3CD,PNRC1,PRKAR2A,PRKCD,PRKCE,PRKCI,PRKD3,PTEN,RARA,RDH11,RXRB,SMAD2,SMAD3,SMAD4,SMAD6,SMAD7,SMARCA4,SRC,TGFB1,TRIM24**
Reelin Signaling in Neurons	0.05Gy 4h	0.45	0.06	NaN	APBB1,RHOA,YES1
**Reelin Signaling in Neurons**	**2Gy 2h**	**1.40**	**0.24**	**-0.63**	**APBB1,APOE,CAMK2G,CDC42,ITGA5,MAP2K5,MAPK8,MTOR,NDEL1,PIK3CB,PIK3CD,RAC1**
Reelin Signaling in Neurons	2Gy 4h	0.60	0.30	0.54	AKT1,APBB1,ARHGEF3,CDK5R1,CFL1,CRK,GSK3B,MAP2K1,MAPK8,PIK3CB,PIK3CD,RHOA,SRC,VLDLR,YES1
Regulation of Actin-based Motility by Rho	0.05Gy 4h	0.99	0.11	NaN	ACTA2,RHOA,RHOG
Regulation of Actin-based Motility by Rho	2Gy 2h	1.13	0.26	-0.38	ACTA2,CDC42,ITGA5,RAC1,RHOB,RHOQ,RND3
Regulation of Actin-based Motility by Rho	2Gy 4h	0.00	0.22	1.34	ACTA2,CFL1,PFN1,RAC3,RHOA,ROCK1
Regulation of Cellular Mechanics by Calpain Protease	0.05Gy 2h	0.62	0.03	NaN	CDKN1B
Regulation of Cellular Mechanics by Calpain Protease	2Gy 2h	0.00	0.14	NaN	CCNE1,CDKN1B,ITGA5,RRAS2
Regulation of Cellular Mechanics by Calpain Protease	2Gy 4h	0.35	0.28	0.00	CCNA2,CCNE1,EGFR,EZR,HRAS,KRAS,RRAS2,SRC
Regulation of eIF4 and p70S6K Signaling	2Gy 2h	0.65	0.19	-2.33	IRS1,ITGA5,MTOR,PDPK1,PIK3CB,PIK3CD,PPP2CA,RRAS2,SOS1
Regulation of eIF4 and p70S6K Signaling	2Gy 4h	0.37	0.27	0.71	AKT1,EIF4A2,FAU,HRAS,KRAS,MAP2K1,PIK3CB,PIK3CD,PPP2CA,RPS19,RPS2,RPS29,RRAS2
**Regulation of IL-2 Expression in Activated and Anergic T Lymphocytes**	**0.05Gy 4h**	**1.37**	**0.11**	**NaN**	**FOS,NFATC1,TGFB1,TGFBR1,VAV2**
**Regulation of IL-2 Expression in Activated and Anergic T Lymphocytes**	**2Gy 2h**	**2.06**	**0.28**	**NaN**	**ELK1,IKBKB,IKBKE,MAPK8,NFATC1,NFATC4,PPP3CC,PPP3R1,RAC1,RRAS2,SMAD3,SOS1,TOB1**
**Regulation of IL-2 Expression in Activated and Anergic T Lymphocytes**	**2Gy 4h**	**3.69**	**0.50**	**NaN**	**ELK1,FOS,HRAS,KRAS,MAP2K1,MAP3K1,MAPK8,NFAT5,NFATC1,NFKB1,NFKBIA,PPP3CA,PPP3CB,PPP3CC,PPP3R1,RRAS2,SMAD2,SMAD3,SMAD4,TGFB1,TGFBR1,TOB1,VAV2**
Regulation of the Epithelial-Mesenchymal Transition Pathway	0.05Gy 2h	0.60	0.02	NaN	EGR1,SNAI2
Regulation of the Epithelial-Mesenchymal Transition Pathway	0.05Gy 4h	0.57	0.06	NaN	EGR1,FGF5,JAK2,RHOA,TGFB1,TGFBR1
Regulation of the Epithelial-Mesenchymal Transition Pathway	2Gy 2h	0.43	0.16	NaN	EGR1,FGF2,FGF7,FGF9,JAG2,MAP2K5,mir-34,PIK3CB,PIK3CD,RRAS2,SMAD3,SNAI1,SNAI2,SOS1,TYK2,ZEB2
**Regulation of the Epithelial-Mesenchymal Transition Pathway**	**2Gy 4h**	**1.61**	**0.34**	**NaN**	**ADAM17,AKT1,AXIN1,CTNNB1,EGFR,EGR1,ETS1,FGF2,FGF5,FZD1,GSK3B,HIF1A,HRAS,JAK2,KRAS,LEF1,MAP2K1,mir-34,NFKB1,NOTCH4,PIK3CB,PIK3CD,PSEN2,RBPJ,RHOA,RRAS2,SMAD2,SMAD3,SMAD4,SNAI2,TCF4,TCF7,TGFB1,TGFBR1,WNT5A**
Relaxin Signaling	0.05Gy 4h	0.31	0.05	NaN	FOS,GNAI2,GNG5
Relaxin Signaling	2Gy 2h	0.00	0.10	NaN	ELK1,GNAO1,NAPEPLD,PDE4D,PIK3CB,PIK3CD
Relaxin Signaling	2Gy 4h	0.00	0.24	0.00	ADCY8,AKT1,APEX1,ELK1,FOS,GNAI2,GNAI3,GNG5,MAP2K1,NFKB1,NFKBIA,NPR3,PIK3CB,PIK3CD,PRKAR2A
Remodeling of Epithelial Adherens Junctions	0.05Gy 4h	0.94	0.14	NaN	ACTA2,ARF6
Remodeling of Epithelial Adherens Junctions	2Gy 2h	0.91	0.29	NaN	ACTA2,ARF6,TUBB2A,TUBB4B
Remodeling of Epithelial Adherens Junctions	2Gy 4h	0.59	0.36	NaN	ACTA2,ARF6,CTNNB1,SRC,TUBB2A
Renal Cell Carcinoma Signaling	0.05Gy 4h	0.33	0.05	NaN	FOS,TGFB1
Renal Cell Carcinoma Signaling	2Gy 2h	0.59	0.19	0.00	CDC42,PIK3CB,PIK3CD,RAC1,RRAS2,SLC2A1,SOS1
**Renal Cell Carcinoma Signaling**	**2Gy 4h**	**1.57**	**0.41**	**0.00**	**AKT1,CRK,ETS1,FH,FOS,HIF1A,HRAS,KRAS,MAP2K1,PIK3CB,PIK3CD,RRAS2,SLC2A1,TGFB1,UBC**
Renin-Angiotensin Signaling	0.05Gy 4h	0.55	0.06	0.00	FOS,JAK2,PRKCD,PRKCI
Renin-Angiotensin Signaling	2Gy 2h	0.00	0.14	-0.33	CCL2,ELK1,MAPK8,PIK3CB,PIK3CD,PRKCE,RAC1,RRAS2,SOS1

Ingenuity Canonical Pathways	Model	-log(p-value)	Ratio	z-score	Molecules
Renin-Angiotensin Signaling	2Gy 4h	1.23	0.34	-1.79	ADCY8,ATF2,ELK1,FOS,HRAS,ITPR2,JAK2,KRAS,MAP2K1,MAP3K1,MAPK8,NFKB1,PIK3CB,PIK3CD,PRKAR2A,PRKCD,PRKCE,PRKCI,PRKD3,PTGER2,PTK2B,RRAS2
Retinoate Biosynthesis I	2Gy 4h	0.00	0.14	NaN	BMP2,RDH11
Retinoic acid Mediated Apoptosis Signaling	0.05Gy 4h	0.50	0.07	NaN	RARG,RXRB
**Retinoic acid Mediated Apoptosis Signaling**	**2Gy 2h**	**2.05**	**0.33**	**0.38**	**CASP3,CASP8,CFLAR,PARP2,RARG,RXRA,RXRB,TNFRSF10B,TNFRSF10D**
**Retinoic acid Mediated Apoptosis Signaling**	**2Gy 4h**	**2.12**	**0.48**	**1.51**	**BID,CASP3,CFLAR,CRABP2,PARP2,PARP9,RARA,RXRB,TNFRSF10A,TNFRSF10B,TNFRSF10C,TNFRSF10D,TNFSF10**
Retinol Biosynthesis	2Gy 2h	0.00	0.08	NaN	LIPE
Retinol Biosynthesis	2Gy 4h	0.00	0.23	NaN	CES2,LIPE,RDH11
RhoA Signaling	0.05Gy 4h	0.44	0.07	NaN	ACTA2,RHOA
RhoA Signaling	2Gy 2h	0.38	0.17	-1.34	ACTA2,LIMK2,LPAR6,PLD1,RND3
RhoA Signaling	2Gy 4h	0.98	0.37	0.30	ACTA2,CFL1,EZR,LIMK2,LPAR1,PFN1,PLD1,PTK2B,RHOA,ROCK1,ROCK2
RhoGDI Signaling	0.05Gy 4h	1.21	0.10	NaN	ACTA2,GNAI2,GNG5,RHOA,RHOG
RhoGDI Signaling	2Gy 2h	0.55	0.18	0.71	ACTA2,CDC42,GNAO1,ITGA5,LIMK2,RAC1,RHOB,RHOQ,RND3
RhoGDI Signaling	2Gy 4h	0.40	0.28	-0.63	ACTA2,ARHGEF3,CD44,CFL1,EZR,GNAI2,GNAI3,GNG5,LIMK2,RAC3,RHOA,ROCK1,ROCK2,SRC
**Role of BRCA1 in DNA Damage Response**	**0.05Gy 4h**	**1.91**	**0.18**	**NaN**	**CDKN1A,E2F4,E2F5,SMARCA4**
Role of BRCA1 in DNA Damage Response	2Gy 2h	0.00	0.14	NaN	CDKN1A,CHEK1,MSH6
**Role of BRCA1 in DNA Damage Response**	**2Gy 4h**	**1.53**	**0.46**	**0.71**	**ATM,BARD1,BRCA2,CDKN1A,E2F1,E2F5,GADD45A,MSH6,RBL2,SMARCA4**
**Role of CHK Proteins in Cell Cycle Checkpoint Control**	**0.05Gy 4h**	**2.66**	**0.29**	**0.00**	**CDC25C,CDKN1A,E2F4,E2F5**
Role of CHK Proteins in Cell Cycle Checkpoint Control	2Gy 2h	0.91	0.29	NaN	CDKN1A,CHEK1,PCNA,PPP2CA
Role of CHK Proteins in Cell Cycle Checkpoint Control	2Gy 4h	0.95	0.43	0.45	ATM,CDKN1A,E2F1,E2F5,PCNA,PPP2CA
**Role of Cytokines in Mediating Communication between Immune Cells**	**0.05Gy 2h**	**1.56**	**0.07**	**NaN**	**IL12A,IL6**
Role of Cytokines in Mediating Communication between Immune Cells	0.05Gy 4h	0.00	0.04	NaN	TGFB1
Role of Cytokines in Mediating Communication between Immune Cells	2Gy 2h	0.00	0.07	NaN	CXCL8,IL6
Role of Cytokines in Mediating Communication between Immune Cells	2Gy 4h	0.00	0.07	NaN	IL12A,TGFB1
**Role of Hypercytokinemia/hyperchemokinemia in the Pathogenesis of Influenza**	**0.05Gy 2h**	**1.84**	**0.10**	**NaN**	**IL12A,IL6**
Role of Hypercytokinemia/hyperchemokinemia in the Pathogenesis of Influenza	2Gy 2h	0.26	0.15	NaN	CCL2,CXCL8,IL6
Role of Hypercytokinemia/hyperchemokinemia in the Pathogenesis of Influenza	2Gy 4h	0.00	0.05	NaN	IL12A
Role of IL-17A in Arthritis	0.05Gy 2h	0.63	0.04	NaN	PTGS2
**Role of IL-17A in Arthritis**	**2Gy 2h**	**1.47**	**0.29**	**NaN**	**CCL2,CCL7,CXCL1,CXCL8,MAPK8,PIK3CB,PIK3CD,PTGS2**
Role of IL-17A in Arthritis	2Gy 4h	0.60	0.32	NaN	ATF2,IL17RA,MAP2K1,MAPK8,NFKB1,NFKBIA,PIK3CB,PIK3CD,PTGS2
Role of IL-17A in Psoriasis	2Gy 2h	0.38	0.20	NaN	CXCL1,CXCL8
Role of IL-17A in Psoriasis	2Gy 4h	0.00	0.10	NaN	IL17RA
**Role of IL-17F in Allergic Inflammatory Airway Diseases**	**0.05Gy 2h**	**1.76**	**0.09**	**NaN**	**IL11,IL6**
**Role of IL-17F in Allergic Inflammatory Airway Diseases**	**2Gy 2h**	**1.58**	**0.32**	**-1.63**	**ATF4,CCL2,CCL7,CXCL1,CXCL8,IL11,IL6**

Ingenuity Canonical Pathways	Model	-log(p-value)	Ratio	z-score	Molecules
Role of IL-17F in Allergic Inflammatory Airway Diseases	2Gy 4h	0.00	0.23	0.00	ATF2,ATF4,IL17RA,MAP2K1,NFKB1
**Role of JAK family kinases in IL-6-type Cytokine Signaling**	**0.05Gy 2h**	**2.21**	**0.15**	**NaN**	**IL6,SOCS3**
Role of JAK family kinases in IL-6-type Cytokine Signaling	0.05Gy 4h	0.37	0.08	NaN	JAK2
**Role of JAK family kinases in IL-6-type Cytokine Signaling**	**2Gy 2h**	**2.27**	**0.46**	**NaN**	**IL6,IL6R,MAPK8,SOCS1,SOCS3,TYK2**
Role of JAK family kinases in IL-6-type Cytokine Signaling	2Gy 4h	0.68	0.39	NaN	IL6R,IL6ST,JAK2,MAPK8,SOCS3
Role of JAK1 and JAK3 in Î³c Cytokine Signaling	0.05Gy 2h	0.56	0.03	NaN	SOCS3
Role of JAK1 and JAK3 in Î³c Cytokine Signaling	0.05Gy 4h	0.00	0.03	NaN	JAK2
Role of JAK1 and JAK3 in Î³c Cytokine Signaling	2Gy 2h	1.03	0.24	NaN	IL4R,IRS1,IRS2,PIK3CB,PIK3CD,RRAS2,SOCS1,SOCS3
Role of JAK1 and JAK3 in Î³c Cytokine Signaling	2Gy 4h	0.31	0.27	NaN	HRAS,IL4R,JAK2,KRAS,PIK3CB,PIK3CD,PTK2B,RRAS2,SOCS3
Role of JAK1, JAK2 and TYK2 in Interferon Signaling	0.05Gy 4h	0.42	0.09	NaN	JAK2
Role of JAK1, JAK2 and TYK2 in Interferon Signaling	2Gy 2h	0.71	0.27	NaN	PTPN2,SOCS1,TYK2
Role of JAK1, JAK2 and TYK2 in Interferon Signaling	2Gy 4h	0.26	0.27	NaN	IFNGR1,JAK2,NFKB1
Role of JAK2 in Hormone-like Cytokine Signaling	0.05Gy 2h	0.76	0.05	NaN	SOCS3
Role of JAK2 in Hormone-like Cytokine Signaling	0.05Gy 4h	0.69	0.10	NaN	JAK2,SOCS6
**Role of JAK2 in Hormone-like Cytokine Signaling**	**2Gy 2h**	**3.10**	**0.45**	**NaN**	**IRS1,IRS2,SH2B2,SOCS1,SOCS2,SOCS3,SOCS5,SOCS6,TYK2**
**Role of JAK2 in Hormone-like Cytokine Signaling**	**2Gy 4h**	**1.39**	**0.45**	**NaN**	**GHR,JAK2,PRLR,SH2B2,SOCS2,SOCS3,SOCS5,SOCS6,SOCS7**
Role of Macrophages, Fibroblasts and Endothelial Cells in Rheumatoid Arthritis	0.05Gy 2h	0.63	0.02	NaN	IL6,MYC,SOCS3
Role of Macrophages, Fibroblasts and Endothelial Cells in Rheumatoid Arthritis	0.05Gy 4h	0.00	0.04	NaN	FOS,JAK2,NFATC1,PRKCD,PRKCI,RHOA,TGFB1
Role of Macrophages, Fibroblasts and Endothelial Cells in Rheumatoid Arthritis	2Gy 2h	0.53	0.16	NaN	ATF4,CAMK2G,CCL2,CEBPB,CEBPD,CXCL8,FGF2,GNAO1,IKBKB,IKBKE,IL16,IL6,IL6R,MYC,NFATC1,NFATC4,PDGFA,PIK3CB,PIK3CD,PPP3CC,PPP3R1,PRKCE,RAC1,RIPK1,RRAS2,SOCS1,SOCS3,TNFRSF1B
**Role of Macrophages, Fibroblasts and Endothelial Cells in Rheumatoid Arthritis**	**2Gy 4h**	**1.52**	**0.31**	**NaN**	**AKT1,ATF2,ATF4,AXIN1,CEBPD,CEBPG,CSF1,CTNNB1,DKK1,FGF2,FOS,FZD1,GSK3B,HRAS,ICAM1,IL16,IL17RA,IL18R1,IL1R1,IL6R,IL6ST,JAK2,KRAS,LEF1,MAP2K1,MAP3K7,NFAT5,NFATC1,NFKB1,NFKBIA,NGFR,PGF,PIK3CB,PIK3CD,PPP3CA,PPP3CB,PPP3CC,PPP3R1,PRKCD,PRKCE,PRKCI,PRKD3,RHOA,RIPK1,ROCK1,ROCK2,RRAS2,SOCS3,SRC,TCF4,TCF7,TGFB1,TNFRSF11B,TNFRSF1B,TRAF3,WNT5A**
Role of MAPK Signaling in the Pathogenesis of Influenza	0.05Gy 2h	0.62	0.03	NaN	PTGS2
Role of MAPK Signaling in the Pathogenesis of Influenza	0.05Gy 4h	0.00	0.03	NaN	BCL2
Role of MAPK Signaling in the Pathogenesis of Influenza	2Gy 2h	0.67	0.21	NaN	CASP3,CCL2,MAPK8,PNPLA8,PTGS2,RRAS2
Role of MAPK Signaling in the Pathogenesis of Influenza	2Gy 4h	1.07	0.38	NaN	AKT1,ATF2,BCL2,CASP3,HRAS,KRAS,MAP2K1,MAPK8,PNPLA8,PTGS2,RRAS2
Role of NANOG in Mammalian Embryonic Stem Cell Pluripotency	0.05Gy 4h	0.00	0.02	NaN	JAK2
Role of NANOG in Mammalian Embryonic Stem Cell Pluripotency	2Gy 2h	0.00	0.10	-1.34	GATA6,PIK3CB,PIK3CD,RRAS2,SOS1,TYK2
Role of NANOG in Mammalian Embryonic Stem Cell Pluripotency	2Gy 4h	1.07	0.33	-0.28	AKT1,AXIN1,BMP1,BMP2,BMP4,BMPR2,CTNNB1,FZD1,GSK3B,HRAS,IL6ST,JAK2,KRAS,LIF,LIFR,MAP2K1,PIK3CB,PIK3CD,RRAS2,SMAD4,WNT5A
Role of NFAT in Cardiac Hypertrophy	0.05Gy 2h	0.64	0.02	NaN	IL11,IL6
Role of NFAT in Cardiac Hypertrophy	0.05Gy 4h	0.66	0.06	0.00	GNAI2,GNG5,PRKCD,PRKCI,TGFB1,TGFBR1

Ingenuity Canonical Pathways	Model	-log(p-value)	Ratio	z-score	Molecules
Role of NFAT in Cardiac Hypertrophy	2Gy 2h	0.43	0.16	-1.39	CACNA1A,CAMK2G,HDAC6,IL11,IL6,MAPK8,MEF2D,NFATC4,PIK3CB,PIK3CD,PPP3CC,PPP3R1,PRKCE,RRAS2,SOS1
**Role of NFAT in Cardiac Hypertrophy**	**2Gy 4h**	**1.40**	**0.33**	**-0.71**	**ADCY8,AKT1,CACNA1C,CAMK1,GNAI2,GNAI3,GNG5,GSK3B,HRAS,IL6ST,ITPR2,KRAS,LIF,MAP2K1,MAP3K1,MAP3K7,MAPK8,PIK3CB,PIK3CD,PPP3CA,PPP3CB,PPP3CC,PPP3R1,PRKAR2A,PRKCD,PRKCE,PRKCI,PRKD3,RRAS2,SRC,TGFB1,TGFBR1**
Role of NFAT in Regulation of the Immune Response	0.05Gy 4h	0.48	0.06	NaN	FOS,GNAI2,GNG5,NFATC1
Role of NFAT in Regulation of the Immune Response	2Gy 2h	0.59	0.17	-2.31	GNAO1,IKBKB,IKBKE,MEF2D,NFATC1,NFATC4,PIK3CB,PIK3CD,PPP3CC,PPP3R1,RRAS2,SOS1
Role of NFAT in Regulation of the Immune Response	2Gy 4h	0.92	0.32	-0.69	AKT1,ATF2,FOS,GNAI2,GNAI3,GNG5,GSK3B,HRAS,ITPR2,KRAS,MAP2K1,NFAT5,NFATC1,NFKB1,NFKBIA,PIK3CB,PIK3CD,PPP3CA,PPP3CB,PPP3CC,PPP3R1,RRAS2
Role of Oct4 in Mammalian Embryonic Stem Cell Pluripotency	0.05Gy 4h	1.15	0.13	NaN	JARID2,REST,RXRB
Role of Oct4 in Mammalian Embryonic Stem Cell Pluripotency	2Gy 2h	0.00	0.04	NaN	RXRB
Role of Oct4 in Mammalian Embryonic Stem Cell Pluripotency	2Gy 4h	0.71	0.35	NaN	BMI1,ETS2,IGF2BP1,JARID2,PHB,RARA,REST,RXRB
Role of Osteoblasts, Osteoclasts and Chondrocytes in Rheumatoid Arthritis	0.05Gy 2h	0.44	0.01	NaN	IL11,IL6
Role of Osteoblasts, Osteoclasts and Chondrocytes in Rheumatoid Arthritis	0.05Gy 4h	0.00	0.03	NaN	BCL2,FOS,NFATC1,TGFB1
Role of Osteoblasts, Osteoclasts and Chondrocytes in Rheumatoid Arthritis	2Gy 2h	0.00	0.11	NaN	BIRC2,IKBKB,IKBKE,IL11,IL6,ITGA5,MAPK8,NFATC1,NFATC4,PIK3CB,PIK3CD,PPP3CC,PPP3R1,RUNX2,TNFRSF1B
Role of Osteoblasts, Osteoclasts and Chondrocytes in Rheumatoid Arthritis	2Gy 4h	1.05	0.30	NaN	ADAM17,AKT1,AXIN1,BCL2,BMP1,BMP2,BMP4,BMPR2,CSF1,CTNNB1,DKK1,FOS,FZD1,GSK3B,IL18R1,IL1R1,LEF1,MAP3K7,MAPK8,NFAT5,NFATC1,NFKB1,NFKBIA,NGFR,PIK3CB,PIK3CD,PPP3CA,PPP3CB,PPP3CC,PPP3R1,PTK2B,SMAD4,SMAD6,SRC,TCF4,TCF7,TGFB1,TNFRSF11B,TNFRSF1B,WNT5A,XIAP
Role of p14/p19ARF in Tumor Suppression	0.05Gy 4h	0.34	0.07	NaN	MDM2
**Role of p14/p19ARF in Tumor Suppression**	**2Gy 2h**	**1.44**	**0.36**	**0.00**	**MDM2,NPM1,PIK3CB,PIK3CD,RAC1**
Role of p14/p19ARF in Tumor Suppression	2Gy 4h	0.95	0.43	0.45	CDKN2A,E2F1,MDM2,NPM1,PIK3CB,PIK3CD
**Role of Pattern Recognition Receptors in Recognition of Bacteria and Viruses**	**0.05Gy 2h**	**1.35**	**0.03**	**NaN**	**IL11,IL12A,IL6**
Role of Pattern Recognition Receptors in Recognition of Bacteria and Viruses	0.05Gy 4h	0.00	0.03	NaN	PRKCD,PRKCI,TGFB1
Role of Pattern Recognition Receptors in Recognition of Bacteria and Viruses	2Gy 2h	0.00	0.11	-0.82	CXCL8,IL11,IL6,MAPK8,NOD1,PIK3CB,PIK3CD,PRKCE,RNASEL
Role of Pattern Recognition Receptors in Recognition of Bacteria and Viruses	2Gy 4h	0.00	0.21	-0.58	CLCF1,CNTF,EIF2AK2,IFIH1,IL12A,IRF7,LIF,MAPK8,NFKB1,NOD1,PIK3CB,PIK3CD,PRKCD,PRKCE,PRKCI,PRKD3,TGFB1,TNFSF10
Role of PI3K/AKT Signaling in the Pathogenesis of Influenza	0.05Gy 4h	0.00	0.04	NaN	GNAI2
Role of PI3K/AKT Signaling in the Pathogenesis of Influenza	2Gy 2h	0.00	0.08	NaN	PIK3CB,PIK3CD
Role of PI3K/AKT Signaling in the Pathogenesis of Influenza	2Gy 4h	1.04	0.39	0.33	AKT1,CRK,GNAI2,GNAI3,GSK3B,MAP2K1,NFKB1,NFKBIA,PIK3CB,PIK3CD

Ingenuity Canonical Pathways	Model	-log(p-value)	Ratio	z-score	Molecules
Role of PKR in Interferon Induction and Antiviral Response	2Gy 2h	0.73	0.23	NaN	CASP3,CASP8,IKBKB,IKBKE,RNASEL
Role of PKR in Interferon Induction and Antiviral Response	2Gy 4h	1.13	0.41	NaN	AKT1,ATF2,BID,CASP3,EIF2AK2,MAP3K7,NFKB1,NFKBIA,TRAF3
Role of RIG1-like Receptors in Antiviral Innate Immunity	2Gy 2h	0.75	0.25	-1.00	CASP8,IKBKB,IKBKE,RIPK1
Role of RIG1-like Receptors in Antiviral Innate Immunity	2Gy 4h	0.72	0.38	0.45	IFIH1,IRF7,NFKB1,NFKBIA,RIPK1,TRAF3
Role of Tissue Factor in Cancer	0.05Gy 2h	0.35	0.02	NaN	EGR1
Role of Tissue Factor in Cancer	0.05Gy 4h	0.56	0.06	NaN	EGR1,FGF5,JAK2,YES1
**Role of Tissue Factor in Cancer**	**2Gy 2h**	**1.60**	**0.24**	**NaN**	**BCL2L1,CASP3,CCN1,CDC42,CXCL1,CXCL8,EGR1,F3,LIMK2,MTOR,PIK3CB,PIK3CD,PLAUR,RAC1,RRAS2**
**Role of Tissue Factor in Cancer**	**2Gy 4h**	**1.30**	**0.35**	**NaN**	**AKT1,BCL2L1,CASP3,CCN1,CCN2,CFL1,CSF1,EGFR,EGR1,FGF5,HRAS,JAK2,KRAS,LIMK2,P4HB,PIK3CB,PIK3CD,PTEN,PTK2B,RRAS2,SRC,YES1**
Role of Wnt/GSK-3Î² Signaling in the Pathogenesis of Influenza	2Gy 4h	0.74	0.35	-1.00	AXIN1,CTNNB1,FZD1,GSK3B,LEF1,NCOA2,TCF4,TCF7,WNT5A
Salvage Pathways of Pyrimidine Ribonucleotides	0.05Gy 4h	0.50	0.07	NaN	NME2,PRKCD
Salvage Pathways of Pyrimidine Ribonucleotides	2Gy 2h	0.77	0.22	-0.82	ACVR2A,LIMK2,MAPK7,MAPK8,PRKCE,SGK1
Salvage Pathways of Pyrimidine Ribonucleotides	2Gy 4h	0.45	0.30	0.71	ACVR2A,EIF2AK2,LIMK2,MAP2K1,MAPK8,NME2,PRKCD,PRKCE
SAPK/JNK Signaling	0.05Gy 4h	0.28	0.05	NaN	GNG5,NFATC1
**SAPK/JNK Signaling**	**2Gy 2h**	**1.65**	**0.27**	**0.30**	**CDC42,ELK1,IRS1,MAPK8,NFATC1,PIK3CB,PIK3CD,RAC1,RIPK1,RRAS2,SOS1**
**SAPK/JNK Signaling**	**2Gy 4h**	**1.83**	**0.42**	**-0.73**	**ATF2,CRK,ELK1,GADD45A,GNG5,HRAS,KRAS,MAP3K1,MAP3K7,MAP4K4,MAPK8,NFATC1,PIK3CB,PIK3CD,RAC3,RIPK1,RRAS2**
Semaphorin Signaling in Neurons	0.05Gy 4h	0.60	0.09	NaN	RHOA,RHOG
Semaphorin Signaling in Neurons	2Gy 2h	1.03	0.26	NaN	CDC42,LIMK2,RAC1,RHOB,RHOQ,RND3
Semaphorin Signaling in Neurons	2Gy 4h	0.46	0.30	NaN	CFL1,LIMK2,NRP1,RAC3,RHOA,ROCK1,ROCK2
Senescence Pathway	0.05Gy 2h	0.48	0.02	NaN	CDKN1B,IL6
Senescence Pathway	0.05Gy 4h	1.11	0.07	-0.33	CDC25C,CDKN1A,E2F4,E2F5,MDM2,NFATC1,PCGF2,TGFB1,TGFBR1
**Senescence Pathway**	**2Gy 2h**	**2.79**	**0.24**	**-1.67**	**ACVR2A,CACNA1A,CBX2,CDC25B,CDKN1A,CDKN1B,CEBPB,CHEK1,CXCL8,EED,HBP1,IKBKB,IKBKE,IL6,ING1,MAP2K5,MAPK7,MDM2,MTOR,NFATC1,NFATC4,PIK3CB,PIK3CD,PPP2CA,PPP3CC,PPP3R1,RRAS2,SERPINE1,SMAD3,SMAD7**
**Senescence Pathway**	**2Gy 4h**	**3.61**	**0.39**	**-1.44**	**ACVR1,ACVR1B,ACVR2A,AKT1,ATM,BMPR2,CACNA1C,CBX2,CCNB1,CDC25B,CDKN1A,CDKN2A,CDKN2B,DLD,E2F1,E2F5,EED,ETS1,ETS2,FOXO3,GADD45A,HRAS,ITPR2,KRAS,MAP2K1,MAP3K7,MDM2,NFAT5,NFATC1,NFKB1,PHF1,PIK3CB,PIK3CD,PPP1CA,PPP2CA,PPP3CA,PPP3CB,PPP3CC,PPP3R1,PTEN,RBL2,RRAS2,SMAD2,SMAD3,SMAD4,SMAD6,SMAD7,TGFB1,TGFBR1**
Serine Biosynthesis	2Gy 4h	0.24	0.33	NaN	PSPH
Serotonin Receptor Signaling	2Gy 2h	0.00	0.06	NaN	GCH1
Serotonin Receptor Signaling	2Gy 4h	0.00	0.13	NaN	ADCY8,GCH1
Sertoli Cell-Sertoli Cell Junction Signaling	0.05Gy 4h	0.00	0.03	NaN	ACTA2,RAB8B
Sertoli Cell-Sertoli Cell Junction Signaling	2Gy 2h	0.00	0.13	NaN	ACTA2,CDC42,ELK1,ITGA5,MAPK8,RAC1,RRAS2,TUBB2A,TUBB4B
**Sertoli Cell-Sertoli Cell Junction Signaling**	**2Gy 4h**	**1.54**	**0.36**	**NaN**	**ACTA2,AKT1,ATF2,AXIN1,BCAR1,CTNNB1,DLG1,ELK1,GSK3B,HRAS,KRAS,MAP2K1,MAP3K1,MAP3K7,MAPK8,NECTIN1,NECTIN2,PRKAR2A,PTEN,RAB8B,RRAS2,SORBS1,SRC,TJP1,TUBB2A**
Signaling by Rho Family GTPases	0.05Gy 4h	1.23	0.08	2.00	ACTA2,FOS,GNAI2,GNG5,PRKCI,RHOA,RHOG

Ingenuity Canonical Pathways	Model	-log(p-value)	Ratio	z-score	Molecules
Signaling by Rho Family GTPases	2Gy 2h	0.57	0.17	-0.83	ACTA2,CDC42,ELK1,GNAO1,ITGA5,LIMK2,MAPK8,PIK3CB,PIK3CD,PLD1,RAC1,RHOB,RHOQ,RND3
Signaling by Rho Family GTPases	2Gy 4h	0.77	0.30	0.23	ACTA2,ARHGEF3,CFL1,DES,ELK1,EZR,FOS,GNAI2,GNAI3,GNG5,LIMK2,MAP2K1,MAPK8,NFKB1,NOX4,PIK3CB,PIK3CD,PLD1,PRKCI,PTK2B,RAC3,RHOA,ROCK1,ROCK2,SLC9A1
Sirtuin Signaling Pathway	0.05Gy 2h	0.22	0.01	NaN	MYC
**Sirtuin Signaling Pathway**	**0.05Gy 4h**	**1.35**	**0.08**	**0.38**	**G6PD,GABPA,HSF1,NDUFS8,NDUFV3,NR1H2,STK11,TRIM28**
Sirtuin Signaling Pathway	2Gy 2h	0.00	0.13	0.00	ATG9A,BCL2L11,CXCL8,DUSP6,MAP1LC3B,MAPK7,MTOR,MYC,NAMPT,PPARGC1A,SLC2A1,XPC
**Sirtuin Signaling Pathway**	**2Gy 4h**	**4.18**	**0.43**	**0.54**	**AKT1,APEX1,ARNTL,ATG9A,ATP5F1A,ATP5PF,CPT1A,DUSP6,E2F1,ESRRA,FOXO3,G6PD,GABPA,GADD45A,GLUD1,GSK3B,HIF1A,HSF1,MAP1LC3A,MAP1LC3B,NAMPT,NDUFS1,NDUFS3,NDUFS8,NDUFV1,NDUFV3,NFKB1,NR1H2,NR1H3,PGK1,PPARA,SLC25A5,SLC2A1,SOD1,SOD3,SP1,STK11,TRIM28,TSPO,VDAC1,XPC**
**Small Cell Lung Cancer Signaling**	**0.05Gy 2h**	**2.10**	**0.07**	**NaN**	**CDKN1B,MYC,PTGS2**
Small Cell Lung Cancer Signaling	0.05Gy 4h	0.53	0.07	NaN	BCL2,MAX,RXRB
**Small Cell Lung Cancer Signaling**	**2Gy 2h**	**2.15**	**0.29**	**-2.12**	**BCL2L1,BIRC2,CCNE1,CDKN1B,IKBKB,IKBKE,MAX,MYC,PIK3CB,PIK3CD,PTGS2,RXRA,RXRB**
**Small Cell Lung Cancer Signaling**	**2Gy 4h**	**2.09**	**0.42**	**-0.63**	**ABL1,AKT1,BCL2,BCL2L1,BID,CCNE1,CCNE2,CDKN2B,E2F1,MAX,NFKB1,NFKBIA,PA2G4,PIK3CB,PIK3CD,PTEN,PTGS2,RXRB,TRAF3**
S-methyl-5'-thioadenosine Degradation II	2Gy 2h	0.85	1.00	NaN	MTAP
Sonic Hedgehog Signaling	2Gy 2h	0.00	0.09	NaN	GLI3
Sonic Hedgehog Signaling	2Gy 4h	0.94	0.46	0.00	CCNB1,GLI3,GSK3B,PRKAR2A,PTCH1
Sperm Motility	0.05Gy 4h	0.48	0.06	NaN	JAK2,KIT,PRKCD,PRKCI,YES1
Sperm Motility	2Gy 2h	0.00	0.11	0.00	EPHA2,EPHB3,EPHB4,MAP2K5,MERTK,PDE4D,PNPLA8,PRKCE,TWF1,TYK2
Sperm Motility	2Gy 4h	0.00	0.24	-1.41	ABL1,DDR1,EGFR,EPHA2,EPHB4,FLT1,ITPR2,JAK2,KIT,MAP2K1,MERTK,PDGFRA,PNPLA8,PRKAR2A,PRKCD,PRKCE,PRKCI,PRKD3,PTK2B,SLC12A2,SRC,YES1
Spermine and Spermidine Degradation I	2Gy 2h	0.85	1.00	NaN	SAT1
Spermine and Spermidine Degradation I	2Gy 4h	0.60	1.00	NaN	SAT1
Sphingomyelin Metabolism	2Gy 2h	0.58	0.50	NaN	SGMS1
Sphingomyelin Metabolism	2Gy 4h	0.36	0.50	NaN	SGMS2
Sphingosine and Sphingosine-1-phosphate Metabolism	0.05Gy 4h	1.08	0.50	NaN	SGPP1
Sphingosine and Sphingosine-1-phosphate Metabolism	2Gy 4h	0.36	0.50	NaN	SGPP1
Sphingosine-1-phosphate Signaling	0.05Gy 4h	0.48	0.06	NaN	GNAI2,RHOA,RHOG
Sphingosine-1-phosphate Signaling	2Gy 2h	1.19	0.23	0.00	CASP3,CASP8,CDC42,PDGFA,PIK3CB,PIK3CD,RAC1,RHOB,RHOQ,RND3,S1PR3
Sphingosine-1-phosphate Signaling	2Gy 4h	0.00	0.23	0.00	ADCY8,AKT1,CASP3,GNAI2,GNAI3,PDGFRA,PIK3CB,PIK3CD,PTK2B,RAC3,RHOA
SPINK1 General Cancer Pathway	0.05Gy 2h	0.69	0.04	NaN	IL6
SPINK1 General Cancer Pathway	0.05Gy 4h	0.57	0.08	NaN	JAK2,MT2A
SPINK1 General Cancer Pathway	2Gy 2h	0.96	0.25	-0.82	IL6,IL6R,PIK3CB,PIK3CD,RRAS2,TYK2
**SPINK1 General Cancer Pathway**	**2Gy 4h**	**2.15**	**0.50**	**-1.16**	**AKT1,EGFR,HRAS,IL6R,JAK2,KRAS,MAP2K1,MT1X,MT2A,PIK3CB,PIK3CD,RRAS2**
SPINK1 Pancreatic Cancer Pathway	0.05Gy 4h	1.05	0.17	NaN	TGFB1,TGFBR1
SPINK1 Pancreatic Cancer Pathway	2Gy 2h	0.00	0.08	NaN	SMAD3
SPINK1 Pancreatic Cancer Pathway	2Gy 4h	0.80	0.42	0.45	CPQ,SMAD2,SMAD3,TGFB1,TGFBR1
STAT3 Pathway	0.05Gy 2h	0.68	0.02	NaN	MYC,SOCS3
STAT3 Pathway	0.05Gy 4h	0.74	0.07	-0.45	BCL2,CDKN1A,JAK2,SOCS6,TGFB1,TGFBR1
STAT3 Pathway	2Gy 2h	0.70	0.18	0.00	CDKN1A,CISH,FGF2,IL4R,IL6R,MAPK8,MYC,PTPN2,RAC1,RRAS2,SOCS1,SOCS2,SOCS3,SOCS5,SOCS6,TYK2

Ingenuity Canonical Pathways	Model	-log(p-value)	Ratio	z-score	Molecules
STAT3 Pathway	2Gy 4h	1.28	0.33	-0.82	BCL2,BMPR2,CDKN1A,EGFR,FGF2,FLT1,GHR,HRAS,IL17RA,IL18R1,IL1R1,IL22RA1,IL4R,IL6R,IL6ST,JAK2,KRAS,MAP2K1,MAPK8,NGFR,PDGFRA,RRAS2,SOCS2,SOCS3,SOCS5,SOCS6,SOCS7,SRC,TGFB1,TGFBR1
**Sumoylation Pathway**	**0.05Gy 4h**	**2.12**	**0.14**	**-0.45**	**FOS,MDM2,NR3C1,RHOA,RHOG,UBE2I**
Sumoylation Pathway	2Gy 2h	1.21	0.24	0.38	CDC42,FAS,MAPK8,MDM2,PCNA,RAC1,RHOB,RHOQ,RND3,SP100
**Sumoylation Pathway**	**2Gy 4h**	**1.71**	**0.41**	**-0.58**	**ETS1,FAS,FOS,KDM1A,MAPK8,MDM2,NFKB1,NFKBIA,NR3C1,PCNA,RAC3,RHOA,SMAD4,SP1,STUB1,UBE2I,XIAP**
Superoxide Radicals Degradation	2Gy 4h	0.26	0.29	NaN	SOD1,SOD3
Superpathway of Cholesterol Biosynthesis	2Gy 2h	1.05	0.38	NaN	HMGCR,HMGCS1,SC5D
Superpathway of Cholesterol Biosynthesis	2Gy 4h	0.00	0.25	NaN	HMGCR,HMGCS1
Superpathway of Citrulline Metabolism	2Gy 2h	0.00	0.10	NaN	OAT
Superpathway of Citrulline Metabolism	2Gy 4h	0.00	0.20	NaN	ALDH18A1,OAT
Superpathway of D-myo-inositol (1,4,5)-trisphosphate Metabolism	2Gy 2h	0.27	0.20	NaN	IMPA2
Superpathway of D-myo-inositol (1,4,5)-trisphosphate Metabolism	2Gy 4h	0.00	0.20	NaN	PTEN
Superpathway of Geranylgeranyldiphosphate Biosynthesis I (via Mevalonate)	2Gy 2h	1.27	0.67	NaN	HMGCR,HMGCS1
Superpathway of Geranylgeranyldiphosphate Biosynthesis I (via Mevalonate)	2Gy 4h	0.80	0.67	NaN	HMGCR,HMGCS1
Superpathway of Inositol Phosphate Compounds	0.05Gy 2h	0.57	0.03	NaN	SOCS3
Superpathway of Inositol Phosphate Compounds	0.05Gy 4h	0.39	0.06	NaN	CDC25C,PTPN13
Superpathway of Inositol Phosphate Compounds	2Gy 2h	0.50	0.18	-1.63	CDC25B,DUSP1,PIK3CB,PIK3CD,PTPN2,SOCS3
Superpathway of Inositol Phosphate Compounds	2Gy 4h	0.74	0.33	0.30	CDC25B,DUSP1,NUDT11,PAWR,PIK3CB,PIK3CD,PPP1CA,PPP3CA,PTEN,PTPN13,SOCS3
Superpathway of Melatonin Degradation	2Gy 4h	0.00	0.05	NaN	CYP1B1
Superpathway of Methionine Degradation	2Gy 4h	0.00	0.20	NaN	DLD
Superpathway of Serine and Glycine Biosynthesis I	2Gy 4h	0.00	0.20	NaN	PSPH
Synaptic Long Term Depression	0.05Gy 4h	0.35	0.05	NaN	GNAI2,PRKCD,PRKCI
Synaptic Long Term Depression	2Gy 2h	0.00	0.10	-0.82	CACNA1A,GNAO1,PNPLA8,PPP2CA,PRKCE,RRAS2
Synaptic Long Term Depression	2Gy 4h	0.31	0.26	-0.78	CACNA1C,GNAI2,GNAI3,HRAS,ITPR2,KRAS,MAP2K1,NPR3,PNPLA8,PPP2CA,PRKCD,PRKCE,PRKCI,PRKD3,RRAS2
Synaptic Long Term Potentiation	0.05Gy 4h	0.22	0.04	NaN	PRKCD,PRKCI
Synaptic Long Term Potentiation	2Gy 2h	0.00	0.13	-0.82	ATF4,CAMK2G,PPP3CC,PPP3R1,PRKCE,RRAS2
**Synaptic Long Term Potentiation**	**2Gy 4h**	**1.85**	**0.40**	**-1.41**	**ADCY8,ATF2,ATF4,CACNA1C,HRAS,ITPR2,KRAS,MAP2K1,PPP1CA,PPP3CA,PPP3CB,PPP3CC,PPP3R1,PRKAR2A,PRKCD,PRKCE,PRKCI,PRKD3,RRAS2**
Synaptogenesis Signaling Pathway	0.05Gy 2h	0.24	0.01	NaN	RAB3A
Synaptogenesis Signaling Pathway	0.05Gy 4h	0.47	0.05	0.45	EFNA4,MARCKS,PRKCD,RHOA,YES1
**Synaptogenesis Signaling Pathway**	**2Gy 2h**	**1.34**	**0.21**	**-1.15**	**APOE,ATF4,BDNF,CAMK2G,CDC42,EFNB1,EPHA2,EPHB3,EPHB4,MARCKS,MTOR,PIK3CB,PIK3CD,PRKCE,RAB3A,RAC1,RRAS2,SOS1,STX16**
Synaptogenesis Signaling Pathway	2Gy 4h	0.91	0.31	1.13	ADCY8,AKT1,ATF2,ATF4,CFL1,CRK,CTNNB1,EFNB1,EPHA2,EPHB4,GSK3B,HRAS,HSPA8,KRAS,MARCKS,NECTIN1,PIK3CB,PIK3CD,PRKAR2A,PRKCD,PRKCE,RASGRF1,RHOA,RRAS2,SRC,STXBP4,VLDLR,YES1
**Systemic Lupus Erythematosus In B Cell Signaling Pathway**	**0.05Gy 2h**	**1.48**	**0.03**	**-1.00**	**IL11,IL12A,IL6,MYC**

Ingenuity Canonical Pathways	Model	-log(p-value)	Ratio	z-score	Molecules
Systemic Lupus Erythematosus In B Cell Signaling Pathway	0.05Gy 4h	0.71	0.06	0.00	BCL2,FOS,JAK2,NFATC1,PRKCD,PRKCI,TGFB1,YES1
Systemic Lupus Erythematosus In B Cell Signaling Pathway	2Gy 2h	0.39	0.15	-1.34	BCL2L1,BCL2L11,CXCL8,IL11,IL6,IL6R,MTOR,MYC,NFATC1,NFATC4,PDPK1,PIK3CB,PIK3CD,PPP3CC,PPP3R1,PRKCE,RAC1,RRAS2,SOS1,TYK2
**Systemic Lupus Erythematosus In B Cell Signaling Pathway**	**2Gy 4h**	**1.31**	**0.32**	**0.00**	**AKT1,BCL2,BCL2L1,CLCF1,CNTF,CTNNB1,FOS,FOXO3,GSK3B,HRAS,IFIH1,IFNGR1,IL12A,IL6R,IL6ST,IRF7,JAK2,KRAS,LIF,MAP2K1,MAP3K7,MAP4K4,NFAT5,NFATC1,NFKB1,PIK3CB,PIK3CD,PPP3CA,PPP3CB,PPP3CC,PPP3R1,PRKCD,PRKCE,PRKCI,PRKD3,RAC3,RRAS2,SRC,TGFB1,TNFSF10,TRAF3,YES1**
Systemic Lupus Erythematosus In T Cell Signaling Pathway	0.05Gy 2h	0.23	0.01	NaN	IL6
Systemic Lupus Erythematosus In T Cell Signaling Pathway	0.05Gy 4h	0.45	0.05	0.45	FOS,GNAI2,RHOA,RHOG,YES1
**Systemic Lupus Erythematosus In T Cell Signaling Pathway**	**2Gy 2h**	**1.79**	**0.23**	**0.22**	**ATF4,BCL6,CASP3,CASP8,CDC42,FAS,IL6,MAP2K5,MTOR,PIK3CB,PIK3CD,PPP2CA,PPP3CC,PPP3R1,RAC1,RHOB,RHOQ,RND3,RRAS2,S1PR3,SOS1**
**Systemic Lupus Erythematosus In T Cell Signaling Pathway**	**2Gy 4h**	**1.84**	**0.36**	**-0.17**	**AKT1,ATF2,ATF4,BCL6,CASP3,CD44,CREM,EZR,FAS,FOS,GADD45A,GNAI2,GNAI3,HLA-E,HRAS,KRAS,LEPR,MAP2K1,PIK3CB,PIK3CD,PPP2CA,PPP3CA,PPP3CB,PPP3CC,PPP3R1,RAB4A,RAC3,RHOA,ROCK1,ROCK2,RRAS2,SP1,YES1**
Systemic Lupus Erythematosus Signaling	0.05Gy 2h	0.41	0.02	NaN	IL6
Systemic Lupus Erythematosus Signaling	0.05Gy 4h	0.00	0.04	NaN	FOS,NFATC1
Systemic Lupus Erythematosus Signaling	2Gy 2h	0.52	0.17	NaN	IL6,IL6R,MTOR,NFATC1,NFATC4,PIK3CB,PIK3CD,RRAS2,SOS1
Systemic Lupus Erythematosus Signaling	2Gy 4h	0.00	0.23	NaN	AKT1,CREM,FOS,HLA-E,HRAS,IL6R,KRAS,NFAT5,NFATC1,PIK3CB,PIK3CD,RRAS2
T Cell Exhaustion Signaling Pathway	0.05Gy 2h	0.84	0.03	NaN	IL12A,IL6
T Cell Exhaustion Signaling Pathway	0.05Gy 4h	0.73	0.07	0.00	FOS,JAK2,NFATC1,TGFB1,TGFBR1
T Cell Exhaustion Signaling Pathway	2Gy 2h	1.14	0.21	-0.58	ACVR2A,BCL6,CD274,IL6,IL6R,MAPK8,MTOR,NFATC1,NFATC4,PIK3CB,PIK3CD,PPP2CA,RRAS2,SMAD3,TYK2
**T Cell Exhaustion Signaling Pathway**	**2Gy 4h**	**1.64**	**0.36**	**-2.68**	**ACVR1,ACVR1B,ACVR2A,AKT1,BCL6,BMPR2,FOS,HLA-E,HRAS,IL12A,IL6R,JAK2,KRAS,MAPK8,NFAT5,NFATC1,PDCD1LG2,PIK3CB,PIK3CD,PPP2CA,RRAS2,SMAD2,SMAD3,TCF7,TGFB1,TGFBR1**
T Cell Receptor Signaling	0.05Gy 4h	0.57	0.07	NaN	FOS,NFATC1,VAV2
**T Cell Receptor Signaling**	**2Gy 2h**	**2.34**	**0.30**	**NaN**	**ELK1,IKBKB,IKBKE,MAPK8,NFATC1,NFATC4,PIK3CB,PIK3CD,PPP3CC,PPP3R1,RAC1,RRAS2,SOS1**
**T Cell Receptor Signaling**	**2Gy 4h**	**2.34**	**0.44**	**NaN**	**ELK1,FOS,HRAS,KRAS,MAP2K1,MAP3K1,MAPK8,NFAT5,NFATC1,NFKB1,NFKBIA,PIK3CB,PIK3CD,PPP3CA,PPP3CB,PPP3CC,PPP3R1,RRAS2,VAV2**
T Helper Cell Differentiation	0.05Gy 2h	1.27	0.05	NaN	IL12A,IL6
T Helper Cell Differentiation	0.05Gy 4h	0.29	0.05	NaN	TGFB1,TGFBR1
T Helper Cell Differentiation	2Gy 2h	0.00	0.13	NaN	BCL6,IL4R,IL6,IL6R,TNFRSF1B
T Helper Cell Differentiation	2Gy 4h	0.54	0.30	NaN	BCL6,IFNGR1,IL12A,IL18R1,IL4R,IL6R,IL6ST,NGFR,TGFB1,TGFBR1,TNFRSF11B,TNFRSF1B
TCA Cycle II (Eukaryotic)	2Gy 4h	0.80	0.67	NaN	DLD,FH
**Tec Kinase Signaling**	**0.05Gy 4h**	**3.85**	**0.16**	**1.00**	**ACTA2,FOS,GNAI2,GNG5,JAK2,PRKCD,PRKCI,RHOA,RHOG,VAV2,YES1**
Tec Kinase Signaling	2Gy 2h	1.19	0.21	-1.16	ACTA2,CDC42,FAS,GNAO1,ITGA5,MAPK8,PIK3CB,PIK3CD,PRKCE,RAC1,RHOB,RHOQ,RND3,TNFRSF10B,TYK2
Tec Kinase Signaling	2Gy 4h	1.23	0.34	0.69	ACTA2,FAS,FOS,GNAI2,GNAI3,GNG5,JAK2,MAPK8,NFKB1,PIK3CB,PIK3CD,PRKCD,PRKCE,PRKCI,PRKD3,PTK2B,RAC3,RHOA,SRC,TNFRSF10A,TNFRSF10B,TNFSF10,VAV2,YES1
Telomerase Signaling	0.05Gy 2h	0.40	0.02	NaN	MYC

Ingenuity Canonical Pathways	Model	-log(p-value)	Ratio	z-score	Molecules
Telomerase Signaling	0.05Gy 4h	0.00	0.02	NaN	CDKN1A
Telomerase Signaling	2Gy 2h	0.69	0.19	-1.89	CDKN1A,HDAC6,MYC,PDPK1,PIK3CB,PIK3CD,PPP2CA,PTGES3,RRAS2,SOS1
Telomerase Signaling	2Gy 4h	1.04	0.34	0.54	ABL1,AKT1,CDKN1A,E2F1,EGFR,ETS1,ETS2,HRAS,HSP90AB1,KRAS,MAP2K1,PIK3CB,PIK3CD,PPP2CA,PTGES3,RRAS2,SP1,TEP1
Tetrahydrobiopterin Biosynthesis I	2Gy 2h	0.85	1.00	NaN	GCH1
Tetrahydrobiopterin Biosynthesis I	2Gy 4h	0.60	1.00	NaN	GCH1
Tetrahydrobiopterin Biosynthesis II	2Gy 2h	0.85	1.00	NaN	GCH1
Tetrahydrobiopterin Biosynthesis II	2Gy 4h	0.60	1.00	NaN	GCH1
**Tetrahydrofolate Salvage from 5,10-methenyltetrahydrofolate**	**0.05Gy 4h**	**1.37**	**1.00**	**NaN**	**MTHFD2**
Tetrahydrofolate Salvage from 5,10-methenyltetrahydrofolate	2Gy 2h	0.85	1.00	NaN	MTHFD2
Tetrapyrrole Biosynthesis II	2Gy 2h	0.58	0.50	NaN	ALAS1
TGF-Î² Signaling	0.05Gy 4h	1.15	0.09	NaN	BCL2,FOS,TGFB1,TGFBR1,VDR
TGF-Î² Signaling	2Gy 2h	0.94	0.21	0.00	ACVR2A,CDC42,INHBA,MAPK8,RRAS2,RUNX2,SERPINE1,SMAD3,SMAD7,SOS1,VDR
**TGF-Î² Signaling**	**2Gy 4h**	**4.42**	**0.51**	**-1.09**	**ACVR1,ACVR1B,ACVR2A,BCL2,BMP2,BMP4,BMPR2,FOS,HRAS,INHBA,INHBB,IRF7,KRAS,MAP2K1,MAP3K7,MAPK8,RRAS2,SKI,SMAD2,SMAD3,SMAD4,SMAD6,SMAD7,TGFB1,TGFBR1,VDR,ZNF423**
Th1 and Th2 Activation Pathway	0.05Gy 2h	1.25	0.03	NaN	IL12A,IL6,SOCS3
Th1 and Th2 Activation Pathway	0.05Gy 4h	0.25	0.04	NaN	JAK2,NFATC1,TGFB1,TGFBR1
Th1 and Th2 Activation Pathway	2Gy 2h	0.00	0.14	NaN	ACVR2A,CD274,IL4R,IL6,IL6R,JAG2,NFATC1,NFATC4,PIK3CB,PIK3CD,SOCS1,SOCS3,TYK2
Th1 and Th2 Activation Pathway	2Gy 4h	0.00	0.22	NaN	ACVR1,ACVR1B,ACVR2A,BMPR2,ICAM1,IFNGR1,IL12A,IL18R1,IL4R,IL6R,JAK2,mir-29,NFATC1,NFKB1,NOTCH4,PIK3CB,PIK3CD,PSEN2,SOCS3,TGFB1,TGFBR1
**Th1 Pathway**	**0.05Gy 2h**	**1.72**	**0.05**	**NaN**	**IL12A,IL6,SOCS3**
Th1 Pathway	0.05Gy 4h	0.00	0.03	NaN	JAK2,NFATC1
Th1 Pathway	2Gy 2h	0.43	0.16	-1.41	CD274,IL6,IL6R,NFATC1,NFATC4,PIK3CB,PIK3CD,SOCS1,SOCS3,TYK2
Th1 Pathway	2Gy 4h	0.00	0.23	-1.16	ICAM1,IFNGR1,IL12A,IL18R1,IL6R,JAK2,mir-29,NFATC1,NFKB1,NOTCH4,PIK3CB,PIK3CD,PSEN2,SOCS3
**Th17 Activation Pathway**	**0.05Gy 2h**	**2.18**	**0.07**	**NaN**	**IL12A,IL6,SOCS3**
Th17 Activation Pathway	0.05Gy 4h	0.27	0.05	NaN	JAK2,NFATC1
Th17 Activation Pathway	2Gy 2h	0.90	0.21	-1.41	IL6,IL6R,MTOR,NFATC1,NFATC4,PTGER4,RUNX1,SOCS3,TYK2
Th17 Activation Pathway	2Gy 4h	0.63	0.31	-1.16	HIF1A,HSP90AB1,IL12A,IL1R1,IL6R,JAK2,NFAT5,NFATC1,NFKB1,PTGER2,RORA,RUNX1,SOCS3
Th2 Pathway	0.05Gy 2h	0.84	0.03	NaN	IL12A,SOCS3
Th2 Pathway	0.05Gy 4h	0.00	0.04	NaN	JAK2,TGFB1,TGFBR1
Th2 Pathway	2Gy 2h	0.00	0.10	-1.13	ACVR2A,IL4R,JAG2,PIK3CB,PIK3CD,SOCS3,TYK2
Th2 Pathway	2Gy 4h	0.00	0.22	0.26	ACVR1,ACVR1B,ACVR2A,BMPR2,ICAM1,IL12A,IL4R,JAK2,NFKB1,NOTCH4,PIK3CB,PIK3CD,PSEN2,SOCS3,TGFB1,TGFBR1
The Visual Cycle	2Gy 4h	0.00	0.13	NaN	RDH11
Thioredoxin Pathway	2Gy 4h	0.60	1.00	NaN	TXN
Thrombin Signaling	0.05Gy 4h	0.72	0.07	1.34	GNAI2,GNG5,PRKCD,PRKCI,RHOA,RHOG
Thrombin Signaling	2Gy 2h	0.85	0.19	-1.60	CAMK2G,CDC42,ELK1,GATA2,GATA6,GNAO1,IKBKB,PDPK1,PIK3CB,PIK3CD,PRKCE,RAC1,RHOB,RHOQ,RND3,RRAS2,SOS1
Thrombin Signaling	2Gy 4h	0.71	0.29	-0.60	ADCY8,AKT1,ARHGEF3,CAMK1,EGFR,ELK1,GATA2,GNAI2,GNAI3,GNG5,HRAS,ITPR2,KRAS,MAP2K1,NFKB1,PIK3CB,PIK3CD,PRKCD,PRKCE,PRKCI,PRKD3,RAC3,RHOA,ROCK1,ROCK2,RRAS2,SRC

Ingenuity Canonical Pathways	Model	-log(p-value)	Ratio	z-score	Molecules
Thrombopoietin Signaling	0.05Gy 2h	0.56	0.03	NaN	MYC
Thrombopoietin Signaling	0.05Gy 4h	1.28	0.12	0.00	FOS,JAK2,PRKCD,PRKCI
Thrombopoietin Signaling	2Gy 2h	0.72	0.21	-1.89	IRS2,MYC,PIK3CB,PIK3CD,PRKCE,RRAS2,SOS1
Thrombopoietin Signaling	2Gy 4h	0.92	0.35	0.00	FOS,HRAS,JAK2,KRAS,MAP2K1,PIK3CB,PIK3CD,PRKCD,PRKCE,PRKCI,PRKD3,RRAS2
Thyroid Cancer Signaling	0.05Gy 2h	0.56	0.03	NaN	MYC
Thyroid Cancer Signaling	0.05Gy 4h	0.00	0.03	NaN	RXRB
Thyroid Cancer Signaling	2Gy 2h	0.47	0.18	NaN	BDNF,CXCL8,MYC,RRAS2,RXRA,RXRB
Thyroid Cancer Signaling	2Gy 4h	0.47	0.29	NaN	CTNNB1,GDNF,HRAS,KRAS,LEF1,MAP2K1,RRAS2,RXRB,TCF4,TCF7
Thyroid Hormone Biosynthesis	2Gy 4h	0.60	1.00	NaN	CTSD
**Tight Junction Signaling**	**0.05Gy 4h**	**2.04**	**0.12**	**NaN**	**ACTA2,FOS,HSF1,PRKCI,RHOA,TGFB1,TGFBR1**
Tight Junction Signaling	2Gy 2h	0.00	0.11	NaN	ACTA2,CDC42,PPP2CA,RAC1,STX16,TNFRSF1B
Tight Junction Signaling	2Gy 4h	1.01	0.33	NaN	ACTA2,AKT1,CTNNB1,FOS,HSF1,NECTIN1,NECTIN2,NFKB1,NGFR,PPP2CA,PRKAR2A,PRKCI,PTEN,RHOA,TGFB1,TGFBR1,TJP1,TNFRSF11B,TNFRSF1B
TNFR1 Signaling	0.05Gy 4h	0.22	0.05	NaN	FOS
**TNFR1 Signaling**	**2Gy 2h**	**2.92**	**0.43**	**0.71**	**BIRC2,CASP3,CASP8,CDC42,IKBKB,IKBKE,MAPK8,RIPK1,TNFAIP3**
TNFR1 Signaling	2Gy 4h	1.25	0.43	0.33	BID,CASP3,FOS,MAP3K1,MAPK8,NFKB1,NFKBIA,RIPK1,XIAP
TNFR2 Signaling	0.05Gy 4h	0.32	0.07	NaN	FOS
**TNFR2 Signaling**	**2Gy 2h**	**1.91**	**0.40**	**0.45**	**BIRC2,IKBKB,IKBKE,MAPK8,TNFAIP3,TNFRSF1B**
TNFR2 Signaling	2Gy 4h	1.25	0.47	-0.38	FOS,MAP3K1,MAPK8,NFKB1,NFKBIA,TNFRSF1B,XIAP
Toll-like Receptor Signaling	0.05Gy 2h	0.48	0.02	NaN	IL12A
Toll-like Receptor Signaling	0.05Gy 4h	0.00	0.02	NaN	FOS
Toll-like Receptor Signaling	2Gy 2h	0.00	0.09	1.00	ELK1,IKBKB,MAPK8,TNFAIP3
Toll-like Receptor Signaling	2Gy 4h	0.41	0.28	-1.13	EIF2AK2,ELK1,FOS,IL12A,MAP3K1,MAP3K7,MAP4K4,MAPK8,NFKB1,NFKBIA,PPARA,UBC
TR/RXR Activation	0.05Gy 2h	0.42	0.02	NaN	BCL3
TR/RXR Activation	0.05Gy 4h	0.00	0.04	NaN	MDM2,RXRB
TR/RXR Activation	2Gy 2h	0.77	0.20	NaN	BCL3,MDM2,MTOR,NCOR2,PIK3CB,PIK3CD,PPARGC1A,RXRA,RXRB,SLC2A1
TR/RXR Activation	2Gy 4h	0.00	0.22	NaN	AKT1,ENO1,HIF1A,LDLR,MDM2,NCOA2,PIK3CB,PIK3CD,RXRB,SLC2A1,THRB
Transcriptional Regulatory Network in Embryonic Stem Cells	0.05Gy 4h	0.84	0.13	NaN	REST,TRIM24
Transcriptional Regulatory Network in Embryonic Stem Cells	2Gy 2h	0.40	0.19	NaN	GATA6,SMARCAD1,TRIM24
Transcriptional Regulatory Network in Embryonic Stem Cells	2Gy 4h	0.00	0.19	NaN	REST,SKIL,TRIM24
TREM1 Signaling	0.05Gy 2h	0.48	0.02	NaN	IL6
TREM1 Signaling	0.05Gy 4h	0.00	0.02	NaN	JAK2
TREM1 Signaling	2Gy 2h	0.26	0.14	-0.82	CCL2,CCL7,CXCL8,IL6,ITGA5,NOD1
TREM1 Signaling	2Gy 4h	0.00	0.12	-1.34	AKT1,ICAM1,JAK2,NFKB1,NOD1
Triacylglycerol Biosynthesis	0.05Gy 4h	0.39	0.08	NaN	LPIN2
Triacylglycerol Biosynthesis	2Gy 4h	0.00	0.25	NaN	LPIN2,LPIN3,PLPP3
Triacylglycerol Degradation	2Gy 2h	0.28	0.17	NaN	AARS1,LIPE
Triacylglycerol Degradation	2Gy 4h	0.45	0.33	1.00	AARS1,CES2,LIPE,PRDX6
tRNA Charging	2Gy 2h	0.44	0.33	NaN	AARS1
tRNA Charging	2Gy 4h	0.80	0.67	NaN	AARS1,TARS1
tRNA Splicing	2Gy 2h	0.50	0.25	NaN	NAPEPLD,PDE4D
tRNA Splicing	2Gy 4h	0.00	0.13	NaN	APEX1

Ingenuity Canonical Pathways	Model	-log(p-value)	Ratio	z-score	Molecules
Tryptophan Degradation III (Eukaryotic)	2Gy 4h	0.24	0.33	NaN	HSD17B10
Tumoricidal Function of Hepatic Natural Killer Cells	2Gy 2h	0.50	0.21	NaN	CASP3,CASP8,FAS
Tumoricidal Function of Hepatic Natural Killer Cells	2Gy 4h	0.95	0.43	NaN	AIFM1,BID,CASP3,ENDOG,FAS,ICAM1
**TWEAK Signaling**	**2Gy 2h**	**2.62**	**0.47**	**0.38**	**BIRC2,CASP3,CASP8,IKBKB,IKBKE,RIPK1,TNFRSF12A**
**TWEAK Signaling**	**2Gy 4h**	**1.76**	**0.53**	**-0.71**	**BID,CASP3,NFKB1,NFKBIA,RIPK1,TNFRSF12A,TRAF3,XIAP**
Type I Diabetes Mellitus Signaling	0.05Gy 2h	1.03	0.04	NaN	IL12A,SOCS3
Type I Diabetes Mellitus Signaling	0.05Gy 4h	0.39	0.05	NaN	BCL2,JAK2,SOCS6
**Type I Diabetes Mellitus Signaling**	**2Gy 2h**	**1.43**	**0.24**	**0.28**	**CASP3,CASP8,FAS,IKBKB,IKBKE,MAPK8,RIPK1,SOCS1,SOCS2,SOCS3,SOCS5,SOCS6,TNFRSF1B**
**Type I Diabetes Mellitus Signaling**	**2Gy 4h**	**2.01**	**0.40**	**-0.45**	**BCL2,BID,CASP3,FAS,HLA-E,IFNGR1,IL12A,IL1R1,JAK2,MAP3K7,MAPK8,NFKB1,NFKBIA,NGFR,RIPK1,SOCS2,SOCS3,SOCS5,SOCS6,SOCS7,TNFRSF11B,TNFRSF1B**
Type II Diabetes Mellitus Signaling	0.05Gy 2h	0.33	0.02	NaN	SOCS3
Type II Diabetes Mellitus Signaling	0.05Gy 4h	0.27	0.05	NaN	PRKCD,PRKCI,SOCS6
**Type II Diabetes Mellitus Signaling**	**2Gy 2h**	**2.48**	**0.27**	**-1.16**	**CACNA1A,CEBPB,IKBKB,IKBKE,IRS1,IRS2,MAPK8,MTOR,PDPK1,PIK3CB,PIK3CD,PRKCE,SOCS1,SOCS2,SOCS3,SOCS5,SOCS6,TNFRSF1B**
**Type II Diabetes Mellitus Signaling**	**2Gy 4h**	**1.33**	**0.35**	**-0.24**	**ADIPOR2,AKT1,CACNA1C,MAP3K1,MAP3K7,MAPK8,NFKB1,NFKBIA,NGFR,PIK3CB,PIK3CD,PKM,PRKCD,PRKCE,PRKCI,PRKD3,SOCS2,SOCS3,SOCS5,SOCS6,SOCS7,TNFRSF11B,TNFRSF1B**
Ubiquinol-10 Biosynthesis (Eukaryotic)	2Gy 4h	0.00	0.17	NaN	CYP7B1
UDP-D-xylose and UDP-D-glucuronate Biosynthesis	2Gy 2h	0.85	1.00	NaN	UGDH
UDP-N-acetyl-D-galactosamine Biosynthesis II	0.05Gy 4h	0.92	0.33	NaN	GPI
UDP-N-acetyl-D-galactosamine Biosynthesis II	2Gy 4h	0.80	0.67	NaN	GNPNAT1,GPI
UDP-N-acetyl-D-glucosamine Biosynthesis II	2Gy 4h	0.60	1.00	NaN	GNPNAT1
Unfolded protein response	0.05Gy 4h	0.00	0.04	NaN	BCL2
Unfolded protein response	2Gy 2h	0.83	0.23	0.45	ATF4,CEBPB,CEBPD,HSPA9,INSIG1,MAPK8
**Unfolded protein response**	**2Gy 4h**	**2.83**	**0.54**	**1.73**	**ATF4,ATF6,BCL2,CALR,CEBPD,CEBPG,DDIT3,HSPA6,HSPA8,INSIG1,MAPK8,P4HB,SYVN1,VCP**
Uridine-5'-phosphate Biosynthesis	2Gy 2h	0.85	1.00	NaN	CAD
UVA-Induced MAPK Signaling	0.05Gy 4h	0.00	0.03	NaN	FOS
UVA-Induced MAPK Signaling	2Gy 2h	1.15	0.25	NaN	BCL2L1,CASP3,MAPK8,MTOR,PARP2,PIK3CB,PIK3CD,RRAS2
**UVA-Induced MAPK Signaling**	**2Gy 4h**	**1.43**	**0.41**	**-0.82**	**ATM,BCL2L1,CASP3,EGFR,FOS,HRAS,KRAS,MAPK8,PARP2,PARP9,PIK3CB,PIK3CD,RRAS2**
UVB-Induced MAPK Signaling	0.05Gy 4h	0.99	0.11	NaN	FOS,PRKCD,PRKCI
UVB-Induced MAPK Signaling	2Gy 2h	0.48	0.19	-1.34	MAPK8,MTOR,PIK3CB,PIK3CD,PRKCE
UVB-Induced MAPK Signaling	2Gy 4h	1.28	0.41	0.30	AKT1,EGFR,FOS,MAP2K1,MAPK8,PIK3CB,PIK3CD,PRKCD,PRKCE,PRKCI,PRKD3
UVC-Induced MAPK Signaling	0.05Gy 4h	1.15	0.13	NaN	FOS,PRKCD,PRKCI
UVC-Induced MAPK Signaling	2Gy 2h	0.00	0.13	NaN	MAPK8,PRKCE,RRAS2
**UVC-Induced MAPK Signaling**	**2Gy 4h**	**2.34**	**0.52**	**0.00**	**EGFR,FOS,HRAS,KRAS,MAP2K1,MAPK8,PRKCD,PRKCE,PRKCI,PRKD3,RRAS2,SRC**
Valine Degradation I	2Gy 4h	0.36	0.50	NaN	DLD
**VDR/RXR Activation**	**0.05Gy 2h**	**1.74**	**0.05**	**NaN**	**CDKN1B,HES1,IL12A**
VDR/RXR Activation	0.05Gy 4h	0.95	0.08	0.45	CDKN1A,PRKCD,PRKCI,RXRB,VDR
**VDR/RXR Activation**	**2Gy 2h**	**1.40**	**0.23**	**0.63**	**CCNC,CDKN1A,CDKN1B,CEBPB,HES1,KLF4,MXD1,NCOR2,PDGFA,PRKCE,RUNX2,RXRA,RXRB,VDR**
VDR/RXR Activation	2Gy 4h	0.00	0.23	1.00	CDKN1A,GADD45A,IGFBP6,IL12A,KLF4,NCOA2,PRKCD,PRKCE,PRKCI,PRKD3,RXRB,SP1,THBD,VDR

**Ingenuity Canonical Pathways**	**Model**	**-log(p-value)**	**Ratio**	**z-score**	**Molecules**
VEGF Family Ligand-Receptor Interactions	0.05Gy 4h	0.59	0.07	NaN	FOS,PRKCD,PRKCI
VEGF Family Ligand-Receptor Interactions	2Gy 2h	0.00	0.12	-1.34	PIK3CB,PIK3CD,PRKCE,RRAS2,SOS1
VEGF Family Ligand-Receptor Interactions	2Gy 4h	1.10	0.36	0.54	AKT1,FLT1,FOS,HRAS,KRAS,MAP2K1,NRP1,PGF,PIK3CB,PIK3CD,PRKCD,PRKCE,PRKCI,PRKD3,RRAS2
VEGF Signaling	0.05Gy 4h	0.24	0.04	NaN	ACTA2,BCL2
VEGF Signaling	2Gy 2h	0.00	0.13	-0.82	ACTA2,BCL2L1,PIK3CB,PIK3CD,RRAS2,SOS1
**VEGF Signaling**	**2Gy 4h**	**1.73**	**0.40**	**-0.24**	**ACTA2,AKT1,BCL2,BCL2L1,FLT1,FOXO3,HIF1A,HRAS,KRAS,MAP2K1,PGF,PIK3CB,PIK3CD,PTK2B,ROCK1,ROCK2,RRAS2,SRC**
Virus Entry via Endocytic Pathways	0.05Gy 4h	0.45	0.06	NaN	ACTA2,PRKCD,PRKCI
Virus Entry via Endocytic Pathways	2Gy 2h	1.08	0.22	NaN	ACTA2,AP3B1,AP3S1,CD55,CDC42,ITGA5,PIK3CB,PIK3CD,PRKCE,RAC1,RRAS2
Virus Entry via Endocytic Pathways	2Gy 4h	0.79	0.32	NaN	ABL1,ACTA2,AP3M1,HLA-E,HRAS,ITGB8,KRAS,PIK3CB,PIK3CD,PRKCD,PRKCE,PRKCI,PRKD3,RAC3,RRAS2,SRC
Vitamin-C Transport	2Gy 2h	0.22	0.17	NaN	SLC2A1
Vitamin-C Transport	2Gy 4h	0.77	0.50	NaN	GLRX,SLC2A1,TXN
White Adipose Tissue Browning Pathway	0.05Gy 4h	0.00	0.03	NaN	RARG,RXRB
White Adipose Tissue Browning Pathway	2Gy 2h	0.48	0.17	-2.53	ATF4,BDNF,CACNA1A,CEBPB,LIPE,PPARGC1A,PRDM16,RARG,RXRA,RXRB
White Adipose Tissue Browning Pathway	2Gy 4h	0.00	0.18	-0.91	ADCY8,ATF2,ATF4,CACNA1C,LIPE,NRF1,PPARA,PRKAR2A,RARA,RXRB,THRB
Wnt/Ca+ pathway	0.05Gy 4h	0.20	0.04	NaN	NFATC1
Wnt/Ca+ pathway	2Gy 2h	0.00	0.13	NaN	ATF4,NFATC1,NFATC4
**Wnt/Ca+ pathway**	**2Gy 4h**	**1.39**	**0.44**	**-1.90**	**ATF2,ATF4,AXIN1,FZD1,GSK3B,NFAT5,NFATC1,NFKB1,PPP3CA,WNT5A**
Wnt/Î²-catenin Signaling	0.05Gy 2h	0.26	0.01	NaN	MYC
Wnt/Î²-catenin Signaling	0.05Gy 4h	0.34	0.05	NaN	MDM2,RARG,TGFB1,TGFBR1
Wnt/Î²-catenin Signaling	2Gy 2h	0.00	0.10	-0.82	ACVR2A,GNAO1,MDM2,MYC,PPP2CA,RARG,SOX8,TLE1
**Wnt/Î²-catenin Signaling**	**2Gy 4h**	**1.91**	**0.37**	**1.00**	**ACVR1,ACVR1B,ACVR2A,AKT1,APPL1,AXIN1,BMPR2,CD44,CDKN2A,CSNK2B,CTNNB1,DKK1,FZD1,GJA1,GSK3B,LEF1,MAP3K7,MDM2,PPP2CA,RARA,SOX8,SOX9,SRC,TCF4,TCF7,TGFB1,TGFBR1,TLE1,UBC,WNT5A**
Xenobiotic Metabolism Signaling	0.05Gy 2h	0.00	0.01	NaN	IL6
Xenobiotic Metabolism Signaling	0.05Gy 4h	0.00	0.03	NaN	HS6ST1,PRKCD,PRKCI
Xenobiotic Metabolism Signaling	2Gy 2h	0.68	0.17	NaN	CAMK2G,CHST11,CHST2,CUL3,HMOX1,HS6ST1,IL6,MAP2K5,MAPK7,MAPK8,NCOR2,PIK3CB,PIK3CD,PPARGC1A,PPP2CA,PRKCE,PTGES3,RRAS2,RXRA
Xenobiotic Metabolism Signaling	2Gy 4h	0.00	0.23	NaN	ALDH18A1,CAMK1,CES2,CYP1B1,GCLC,GSTP1,HMOX1,HRAS,HS6ST1,HSP90AB1,KRAS,MAP2K1,MAP3K1,MAP3K7,MAPK8,NFKB1,PIK3CB,PIK3CD,PPP2CA,PRKCD,PRKCE,PRKCI,PRKD3,PTGES3,RRAS2,SOD3

Bold: significant pathways with -log(p-value) ≥ 1.30; blue: inactivated pathway with z-score ≤ -2; red: activated pathway with z-score ≥ 2; Abbreviations: Not a Number (NaN).
