# Supplementary material for: Comparison of time and dose dependent gene expression and affected pathways in primary human fibroblasts after exposure to ionizing radiation
Source: Mol Med. 2020 Sep 9;26:85. doi: 10.1186/s10020-020-00203-0 (PMC7488023; doi:10.1186/s10020-020-00203-0)
Supplement: Supplementary file 13 — Additional file 13: Supplement file 1. Settings for comparison analyses in IPA. [file 10020_2020_203_MOESM13_ESM.docx]

# SUPPLEMENT

Settings for comparison analyses in Ingenuity Pathway Analysis (IPA)

We selected the analysis of fibroblasts (or alike) in experimentally observed pathways in humans in all databases associated with IPA. Direct and indirect relationships of molecules were analyzed.

Consider only molecules and/or relationships were: (species = Human) **AND** (confidence = Experimentally Observed) **AND** (tissues/cell lines = NIH/3T3 cells OR Other Epithelial cells OR Fibroblast cell lines not otherwise specified OR Hepatocytes OR Fibroblasts OR MEF cells OR Epithelial cells not otherwise specified OR Sertoli cells OR Swiss 3T3 cells OR Keratinocytes OR Cos-7 cells OR Melanocytes OR 3T3-L1 cells OR Other Fibroblast cell lines) **AND** (molecular types = biologic drug OR chemical - endogenous mammalian OR chemical - endogenous non-mammalian OR chemical - kinase inhibitor OR chemical - other OR chemical - protease inhibitor OR chemical drug OR chemical reagent OR chemical toxicant OR complex OR cytokine OR disease OR enzyme OR function OR G-protein coupled receptor OR group OR growth factor OR ion channel OR kinase OR ligand-dependent nuclear receptor OR mature microRNA OR microRNA OR other OR peptidase OR phosphatase OR transcription regulator OR translation regulator OR transmembrane receptor OR transporter) **AND** (data sources = An Open Access Database of Genome-wide Association Results OR BIND OR BioGRID OR Catalogue Of Somatic Mutations In Cancer (COSMIC) OR Chemical Carcinogenesis Research Information System (CCRIS) OR ClinicalTrials.gov OR ClinVar OR Cognia OR DIP OR DrugBank OR Gene Ontology (GO) OR GVK Biosciences OR Hazardous Substances Data Bank (HSDB) OR HumanCyc OR Ingenuity Expert Findings OR Ingenuity ExpertAssist Findings OR IntAct OR Interactome studies OR MIPS OR miRBase OR miRecords OR Mouse Genome Database (MGD) OR Obesity Gene Map Database OR Online Mendelian Inheritance in Man (OMIM) OR TarBase OR TargetScan Human).
