# Supplementary material for: Comparison of time and dose dependent gene expression and affected pathways in primary human fibroblasts after exposure to ionizing radiation
Source: Mol Med. 2020 Sep 9;26:85. doi: 10.1186/s10020-020-00203-0 (PMC7488023; doi:10.1186/s10020-020-00203-0)

Web Figure 12: Comparison of predicted downstream diseases and functions in different data sets.

All shown results have a p-value < 0.05. Numbers given in cells are z-scores.

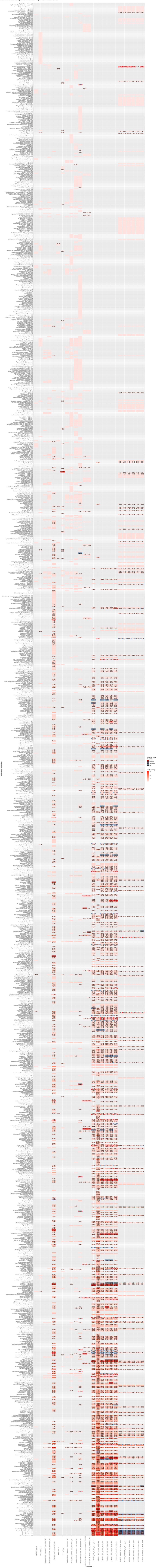

Supplement: Supplementary file 5 — Additional file 5: Web Figure 12. Comparison of predicted downstream diseases and functions in different data sets. [file 10020_2020_203_MOESM5_ESM.pdf]
